# Quick Access to Nucleobase-Modified Phosphoramidites for the Synthesis of Oligoribonucleotides Containing Post-Transcriptional Modifications and Epitranscriptomic Marks

**DOI:** 10.1021/acs.joc.2c01390

**Published:** 2022-07-20

**Authors:** Kamil Ziemkiewicz, Marcin Warminski, Radoslaw Wojcik, Joanna Kowalska, Jacek Jemielity

**Affiliations:** † Centre of New Technologies, University of Warsaw, Banacha 2c, Warsaw 02-097, Poland; ‡ Division of Biophysics, Institute of Experimental Physics, Faculty of Physics, University of Warsaw, Pasteura 5, Warsaw 02-093, Poland

## Abstract

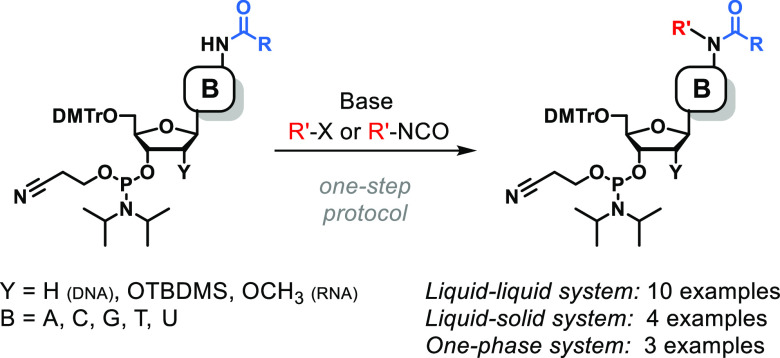

Herein, we report a straightforward one-step procedure for modifying *N*-nucleophilic groups in the nucleobases of commercially available nucleoside phosphoramidites. This method involves the deprotonation of amide groups under phase-transfer conditions and subsequent reaction with electrophilic molecules such as alkyl halides or organic isocyanates. Using this approach, we obtained 10 different classes of modified nucleoside phosphoramidites suitable for the synthesis of oligonucleotides, including several noncanonical nucleotides found in natural RNA or DNA (e.g., m^6^A, i^6^A, m^1^A, g^6^A, m^3^C, m^4^C, m^3^U, m^1^G, and m^2^G). Such modification of nucleobases is a common mechanism for post-transcriptional regulation of RNA stability and translational activity in various organisms. To better understand this process, relevant cellular recognition partners (e.g., proteins) must be identified and characterized. However, this step has been impeded by limited access to molecular tools containing such modified nucleotides.

## Introduction

The post-transcriptional modification of nucleobases is a common process in all domains of life. Noncanonical nucleotides were first observed in calf liver RNA hydrolysates in the early 1950s.^1^ To date, 143 modifications have been identified in various RNA molecules,^2^ whereas 47 modifications have been found in DNA.^3^ The chemical nature of these modifications varies from simple methyl group addition through the attachment of more complex molecules (e.g., amino acid derivatives, saccharides, and terpenes) to ring closure for tricyclic nucleobase formation.^2^ Most studies on RNA modification have focused on sequencing and mapping the whole transcriptome, which provides statistical information that can be difficult to correlate with the biological function.^4^ In some cases, such as for the most abundant *N*6-methyladenosine (m^6^A) mark, the biological effect depends on the structural context of the modification, which further complicates the task.^5,6^ Synthetic oligonucleotides with modified nucleobases have numerous applications in biological studies on natural cellular processes, such as elucidating the role of tRNA modification in codon recognition,^7,8^ characterizing the structures of nucleic acid binding proteins (e.g., epitranscriptomic readers and erasers),^9,10^ developing artificial RNA modification-specific deoxyribozymes,^11^ and creating fluorescent binding probes^12^ and isotopically labeled standards for MS analysis.^13^ However, systematic analyses of the chemical and biological properties of modified nucleic acids are hampered by limited access to nucleic acid fragments containing nucleotides with site-specific modifications. Recently, an elegant method for the ribozymatic methylation of adenosine at the *N*1 position was developed.^14^ Nonetheless, other modifications typically require traditional chemical synthesis.

The chemical synthesis of oligonucleotides is commonly achieved using the phosphoramidite method on a solid support.^15^ This efficient and inexpensive approach has been widely applied by the research and pharmaceutical communities since phosphoramidite building blocks became commercially available. However, the incorporation of nucleotides other than canonical A, C, G, T, and U usually requires the multistep synthesis of appropriate, commercially unavailable building blocks, which makes the process more laborious. The chemical properties of the nucleoside 3′-*O*-phoshoramidites and orthogonal protecting groups required for solid-phase synthesis interfere with most procedures used for nucleobase modification. As such, these modifications must be introduced early in the synthetic route, followed by base and sugar protection and phosphitylation.^16^

Notably, Kruse et al. realized the efficient and selective methylation of a fully protected 2′-*O*-methyladenosine phosphoramidite at the *N*6 position under phase-transfer conditions, providing quick access to the m^6^A_m_ building block.^17^ This approach was based on previous work by the Sekine group on the alkylation of 2′,3′,5′-*O*,*O*,*O*-tri-*tert*-butyl-dimethylsilyl (TBDMS)-protected *N*6-acyladenosine anions generated in a two-phase NaOH_aq_/CH_2_Cl_2_ system in the presence of the phase-transfer catalyst Bu_4_NBr.^18^ Silyl-protected adenosine with various *N*6-protecting groups, including acetyl (Ac), phenoxyacetyl (Pac), and 4-nitrobenzoyl amides, was shown to react selectively with active alkylating agents such as methyl, benzyl, and allyl halides. As an exception, the *N*6-benzyladenosine derivative gave a mixture of *N*6 and *N*1 alkylation products.

Inspired by these studies, we investigated the scope of electrophiles compatible with this type of reaction and attempted to apply this approach to phosphoramidites of different nucleosides. We envisage that the generalization of this synthetic method will provide easy access to oligonucleotides containing several natural or unnatural modifications and allow for the introduction of various functional groups into nucleic acid fragments. Consequently, the molecular toolbox for creating structure or activity probes, affinity resins, aptamers, ribozymes, and conjugates with cellular delivery vehicles will be expanded.^19^

## Results and Discussion

**Scheme 1:**
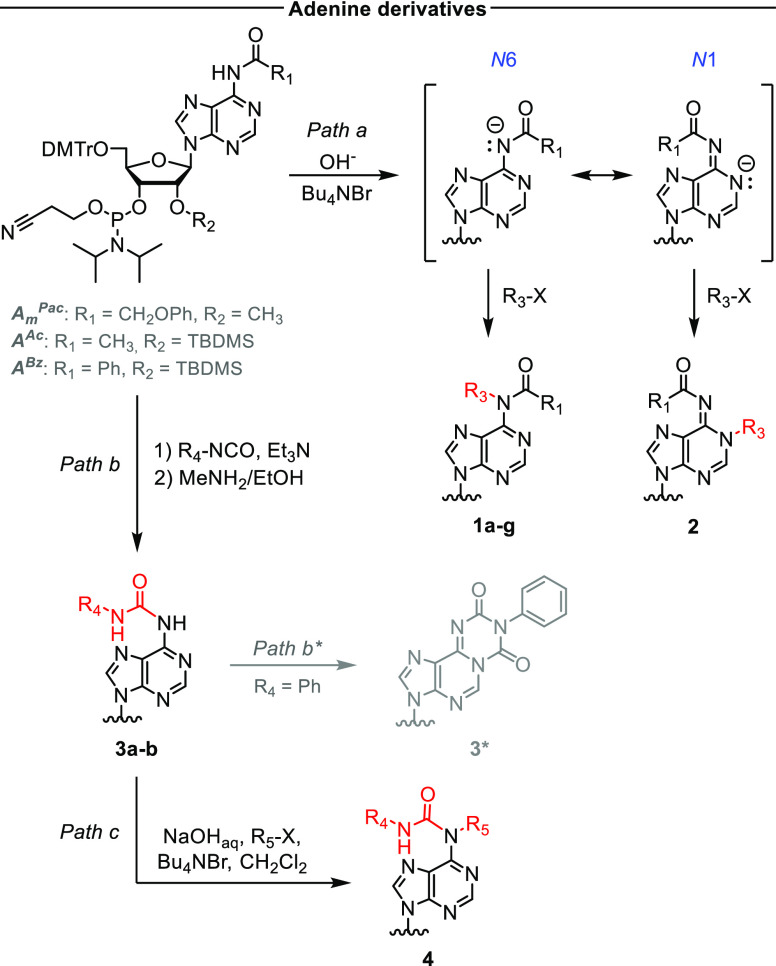
Synthesis of Base-Modified Adenosine 3′-*O*-Phosphoramidites The chemical structures of compounds **1–4** are given in Table 1.

First, we verified whether fully protected adenosine phosphoramidite could be alkylated with electrophiles other than methyl iodide. We chose commercially available *N*6-acetyl and *N*6-phenoxyacetyl phosphoramidites because these protecting groups provided the best results for silyl-protected adenosine.^18^ Active alkylating agents such as benzyl and isopentenyl bromides reacted readily with *N*6-acetyl 2′-*O*-methyladenosine and *N*6-phenoxyacetyl-2′-*O*-TBDMS-adenosine phosphoramidites in 1 M NaOH_aq_/CH_2_Cl_2_ when an equimolar amount of Bu_4_NBr was used (full conversion of the starting material in 15–30 min). In this case, the fully protected *N*6-alkyladenosine phosphoramidites were the only observable product. Catalytic amounts of Bu_4_NBr also promoted the desired reaction, albeit at much lower rates, leading to competition from partial hydrolysis of the phosphoramidite moiety. Using this procedure (Path a, Scheme 1), we obtained phosphoramidites of naturally occurring adenosine derivatives, m^6^A (**1a**) and *N*6-isopentenyladenosine (i^6^A, **1b**), as well as *N*6-benzyladenosine (Bn^6^A) (**1c**) in 59–80% yield (Table 1). Less active alkyl halides, such as 6-iodohex-1-yne, 3-bromopropylphthalimide, and 2-iodopropane, required much longer reaction times, which led to substantial hydrolysis of the phosphoramidite moiety. *N*6-Hexynyladenosine phosphoramidite (**1d**) was isolated in 56% yield, but the phthalimidopropyl and isopropyl derivatives were hydrolyzed before appreciable conversion was achieved. The conditions reported in the literature are then applicable only for modification with very reactive alkylating agents. To accelerate the formation of the desired product and limit hydrolysis, we switched to an anhydrous solid–liquid system with an organic solvent and a mixture of ground solid KOH and K_2_CO_3_ as the base.^20^ Under these conditions, the reaction rate was higher in toluene than in CH_2_Cl_2_ (complete conversion in 1 h vs 2–3 h). The optimal procedure provided amidites **1e** and **1f** in 48 and 45% yield, respectively (Table 1).

**Table 1:**
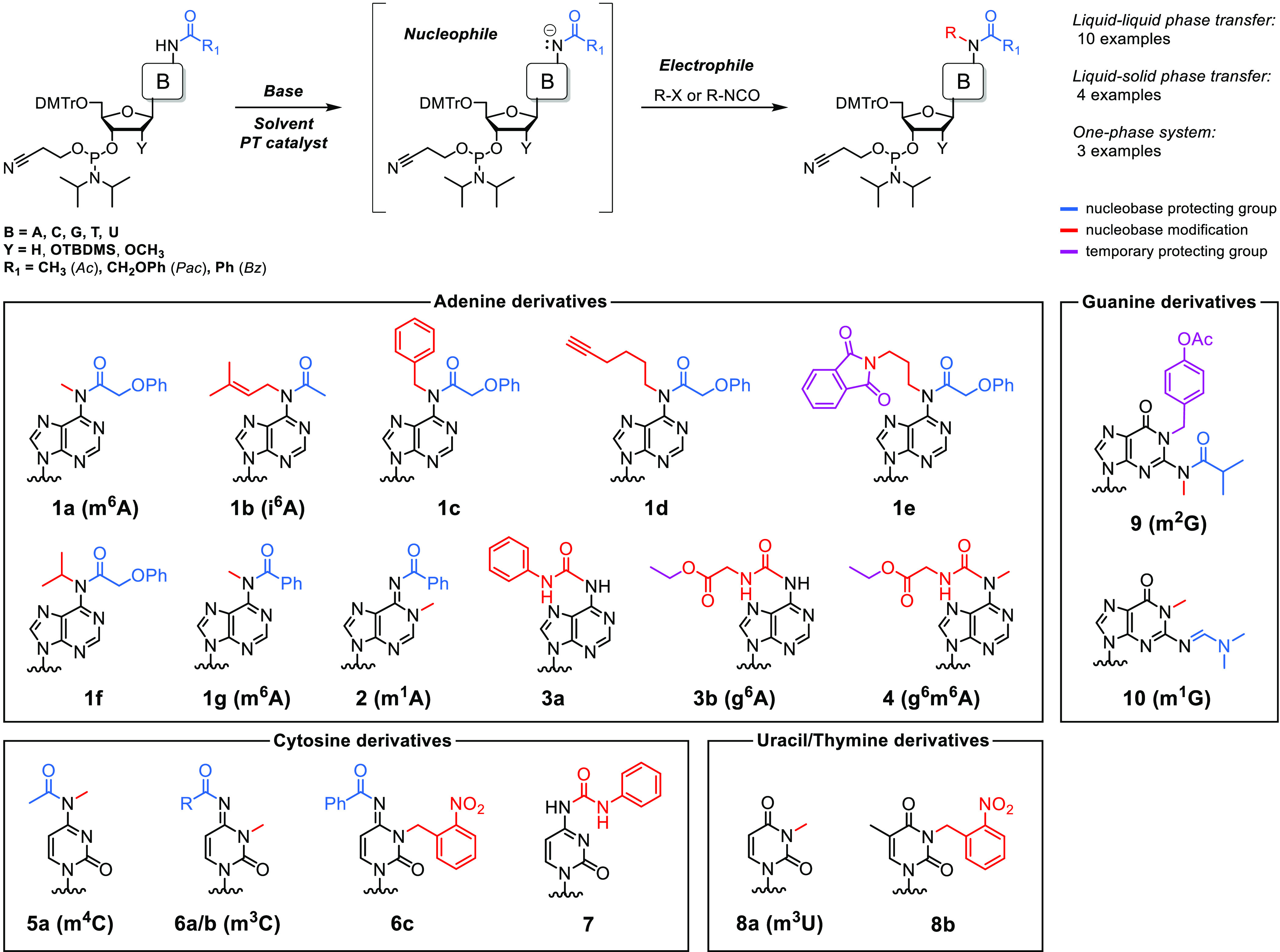
Phosphoramidites of Base-Modified Nucleosides Synthesized in this Work

product	nucleophile^a^	electrophile	base	solvent(s)	phase-transfer catalyst	yield^b^
**1a**	A_m_^Pac^	methyl iodide	1 M NaOH (aq)	CH_2_Cl_2_/H_2_O	Bu_4_NBr	79%
**1b**	A^Ac^	isopentenyl bromide	1 M NaOH (aq)	CH_2_Cl_2_/H_2_O	Bu_4_NBr	80%
**1c**	A_m_^Pac^	benzyl bromide	1 M NaOH (aq)	CH_2_Cl_2_/H_2_O	Bu_4_NBr	59%
**1d**	A_m_^Pac^	6-iodohex-1-yne	1 M NaOH (aq)	CH_2_Cl_2_/H_2_O	Bu_4_NBr	56%
**1e**	A_m_^Pac^	3-phthalimidopropyl bromide	KOH/K_2_CO_3_ (s)	toluene	Bu_4_NBr	48%
**1f**	A_m_^Pac^	2-iodopropane	KOH/K_2_CO_3_ (s)	toluene	Bu_4_NBr	45%
**1g + 2**	A^Bz^	methyl iodide	KOH/K_2_CO_3_ (s)	toluene	Bu_4_NBr	62% + 29%
**3a**	A^Ac^	phenyl isocyanate	triethylamine	CH_2_Cl_2_		57%
**3b**	A^Ac^	ethyl isocyanatoacetate	triethylamine	CH_2_Cl_2_		83%
**4**	g^6^A (**3b**)	methyl iodide	1 M NaOH (aq)	CH_2_Cl_2_/H_2_O	Bu_4_NBr	70%
**5a + 6a**	C^Ac^	methyl iodide	1 M NaOH (aq)	CH_2_Cl_2_/H_2_O	Bu_4_NBr	43% + 25%
**6b**	C^Bz^	methyl iodide	1 M NaOH (aq)	CH_2_Cl_2_/H_2_O	Bu_4_NBr	75%
**6c**	C^Bz^	2-nitrobenzyl chloride	KOH/K_2_CO_3_ (s)	toluene	Bu_4_NBr	73%
**7**	C^Ac^	phenyl isocyanate	triethylamine	CH_2_Cl_2_		42%
**8a**	U_m_	methyl iodide	1 M NaOH (aq)	CH_2_Cl_2_/H_2_O	Bu_4_NBr	89%
**8b**	T	2-nitrobenzyl chloride	KOH/K_2_CO_3_ (s)	toluene	Bu_4_NBr	71%
**9**	G^iBu^	4-(iodomethyl)phenyl acetate, methyl iodide	1 M NaOH (aq)	CH_2_Cl_2_/H_2_O	Bu_4_NBr	12%
**10**	G^dmf^	methyl iodide	1 M NaOH (aq)	CH_2_Cl_2_/H_2_O	Bu_4_NBr	82%

a The protecting group of the exocyclic amine in the nucleoside phosphoramidite is indicated by the superscript, as defined by R_1_ in the abovementioned reaction scheme; the 2′-C substituent (Y in the abovementioned reaction scheme) is −H for DNA amidites, *tert*-butyldimethylsilyloxyl (−OTBDMS) for RNA amidites, and −OCH_3_ for 2′-*O*-methylRNA amidites (denoted by a subscript “m).”

b Isolated yield (flash chromatography).

Aritomo et al. found that the phase-transfer-catalyzed (PTC) alkylation of *N*6-benzoyl-protected 2′,3′,5′-*O*,*O*,*O*-TBDMS-adenosine gave a mixture of *N*6 and *N*1 alkylation products, in contrast to *N*6-acetyl- and *N*6-phenoxyacetyl-protected compounds, which were alkylated only at the *N*6 position.^18^ We envisaged that isomeric product formation results from the mesomeric stabilization of the amide anion (Scheme 1), in which the negative charge is delocalized between two nitrogen atoms. As *N*1-methyladenosine (m^1^A) is also present in natural RNAs, we applied this finding to develop a simple synthetic route to m^1^A phosphoramidite. First, we checked whether this phenomenon was also observed for the alkylation of *N*6-benzoyl-protected adenosine phosphoramidite and, if so, whether the ratio of isomeric products depended on the reaction conditions. Indeed, the methylation of *N*6-benzoyl-protected adenosine phosphoramidite in NaOH_aq_/CH_2_Cl_2_ produced both m^6^A and m^1^A amidites in an 8:2 ratio. In contrast, in the KOH/K_2_CO_3_/toluene system, the distribution of isomeric products shifted slightly toward *N*1-substitution (∼7:3 m^6^A:m^1^A). Isomers **1h** and **2** were isolated by flash chromatography, and their structures were confirmed by NMR. Consistent with the previous reports on nucleosides alkylation, we did not observe *N*1-substitution products for either phenoxyacetyl or acetyl-protected adenosine phosphoramidites.

To expand the scope of this method, we investigated the reaction of other types of electrophilic compounds with adenosine phosphoramidite under alkaline phase-transfer conditions. First, we evaluated representative Michael acceptors, namely, acrylonitrile, methyl cinnamate, and methyl propiolate. In all cases, the reaction proceeded more slowly and was accompanied by substantial degradation of the phosphoramidite. Although the desired products were identified in the reaction mixtures by electrospray ionization mass spectrometry (ESI-MS), their isolation was impractical.

Isocyanates, which are known to react with amines to form urea derivatives, were also tested as electrophiles. Phenyl isocyanate reacted instantaneously with protected adenosine phosphoramidite under phase-transfer conditions in the presence of either aqueous NaOH or solid KOH. However, thin-layer chromatography (TLC) analysis of the reaction mixture revealed multiple unidentified products. We envisaged that reducing the nucleophile concentration by using a milder base would limit the reaction to the isocyanate addition step. Indeed, urea derivative **3a** was formed slowly when triethylamine was used as the base in a single-phase organic solvent (Path b, Scheme 1). Interestingly, for *N*6-benzoyl- and *N*6-phenoxyacetyladenosine phosphoramidites, the initial products reacted further to form the same final product, implying that the *N*6-amide bond was cleaved during the subsequent reactions. Further investigation revealed that the reaction of *N*6-acetyladenosine phosphoramidite also gave an analogous side product, although it only appeared after all the starting material was consumed (4–5 h). Mass spectrometry analysis showed that, in addition to acyl loss, a fragment with *m*/*z* = 28 was attached to the molecule, which could correspond to a carbonyl group. Under the investigated conditions, the only carbonyl group source was phenyl isocyanate, indicating that aniline was produced as a byproduct. Indeed, a peak at *m*/*z* = 94 was identified in the reaction mixture by ESI(+)-MS. A possible product is tricyclic adenosine derivative 3* (Path b*, Scheme 1),^21^ the formation of which would require the loss of the *N*6-acyl group to extend the aromatic system to the third ring. Optimized conditions with *N*6-acetyl-protected adenosine phosphoramidite provided amidite **3a** in 4 h and the product was isolated in 57% yield (Table 1).

In contrast to the reactions with phenyl isocyanate, no side products were observed in the reactions with alkyl isocyanates, which are generally weaker electrophiles. This finding paves the way for the facile and efficient synthesis of an interesting class of compounds, carbamoyladenosine derivatives, which occur naturally in tRNAs at position 37.^22^ It has been postulated that such amino acid–RNA conjugates were present in the early Earth RNA–peptide world.^23^ As an example, we reacted *N*6-acetyl-protected adenosine phosphoramidite with commercially available ethyl isocyanatoacetate and then removed the acetyl group using methylamine. The resulting *N*6-glycinylcarbamoyl-adenosine (g^6^A) phosphoramidite **3b** was isolated in 83% yield (Path b, Scheme 1). With this urea derivative in hand, we investigated selective alkylation at the *N*6 position to achieve both *N*6-carbamoylation and *N*6-methylation (e.g., m^6^t^6^A, another class of adenosine derivatives found in tRNAs).^24^ The reaction of compound **3b** with methyl iodide under phase-transfer conditions proceeded rapidly to give **4** (Path c, Scheme 1), which was isolated in 70% yield.

**Scheme 2:**
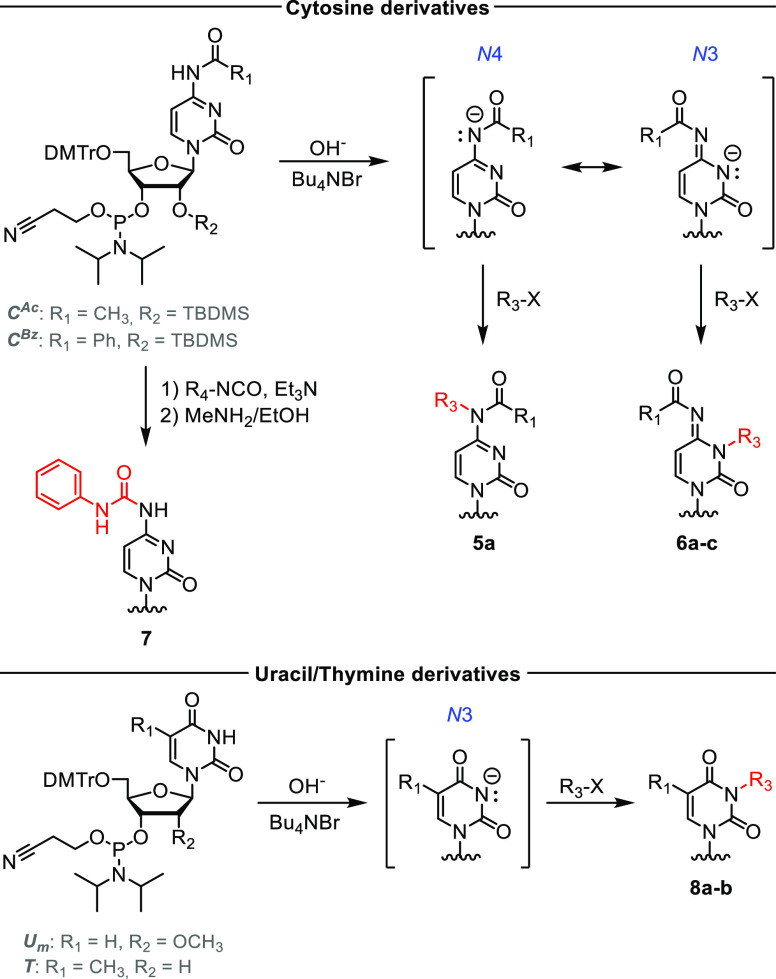
Synthesis of Base-Modified Pyrimidine 3′-*O*-Phosphoramidites The chemical structures of compounds **5–8** are given in Table 1.

Next, we examined analogous modification reactions for the phosphoramidites of another natural nucleoside, cytidine (Scheme 2). The *N*4-acetylcytidine amidite was methylated rapidly in NaOH_aq_/CH_2_Cl_2_, but *N*4-methylcytidine (m^4^C) **5a** and *N*3-methylcytidine (m^3^C) **6a** amidites were produced in a 63:37 ratio. Using the *N*4-benzoyl-protected cytidine derivative, *N*3-methylated compound **6b** was obtained as the main product (15:85 m^4^C:m^3^C), which is consistent with the findings for adenosine (*N*4 of C is equivalent to *N*6 of A and *N*3 of C is equivalent to *N*1 of A). The ratio of isomeric products was significantly affected by the polarity of the solvent in solid–liquid systems. In the dimethylformamide (DMF)/tetrahydrofuran/CH_2_Cl_2_/toluene series, solvents with a high dielectric constant (i.e., DMF) promoted *N*3-alkylation, whereas those with a low dielectric constant (i.e., toluene) promoted *N*4-alkylation. Catalysts other than Bu_4_NBr, namely, tetrabutylammonium hydrogen sulfate, benzyltriethylammonium chloride, and Aliquat 336, did not improve the reaction yield and rate. To estimate the reactivity of the nucleophiles generated from cytidine phosphoramidites, we performed reactions with less active alkyl halides, namely, 3-phthalimidopropyl bromide and 2-iodopropane. The reactivity of 3-phthalimidopropyl bromide toward cytidine phosphoramidite was comparable to that of the adenosine amidite, whereas no product was observed in the reaction with the secondary halide (2-iodopropane).

In the case of uridine, PTC deprotonation has been reported for selective alkylation at the *N*3 position.^25^ Here, we found that this methodology was also applicable to uridine 3′-*O*-phosphoramidites (Scheme 2), providing *N*3-substituted U building blocks. As an example, we chose a naturally occurring modification of uridine, *N*3,2′-*O*-dimethyluridine (m^3^U_m_), which is found in the mRNA cap-4 structure of early eukaryotes such as *Trypanosoma*.^26^ Corresponding phosphoramidite **8a** was formed in 25 min and isolated in 89% yield. Other *N*3 modifications of uridine and thymidine, except for *N*3-(3-amino-3-carboxypropyl)-uridine (acp^3^U),^27^ have limited applications in biological studies because they interfere with Watson–Crick base pairing. However, they may be useful for controlling oligonucleotide hybridization when photolabile substituents such as the 2-nitrobenzyl group are used.^28^ We obtained photoactivable derivative **8b** in 71% yield by alkylation of thymidine phosphoramidite with 2-nitrobenzyl chloride (Table 1).

**Scheme 3:**
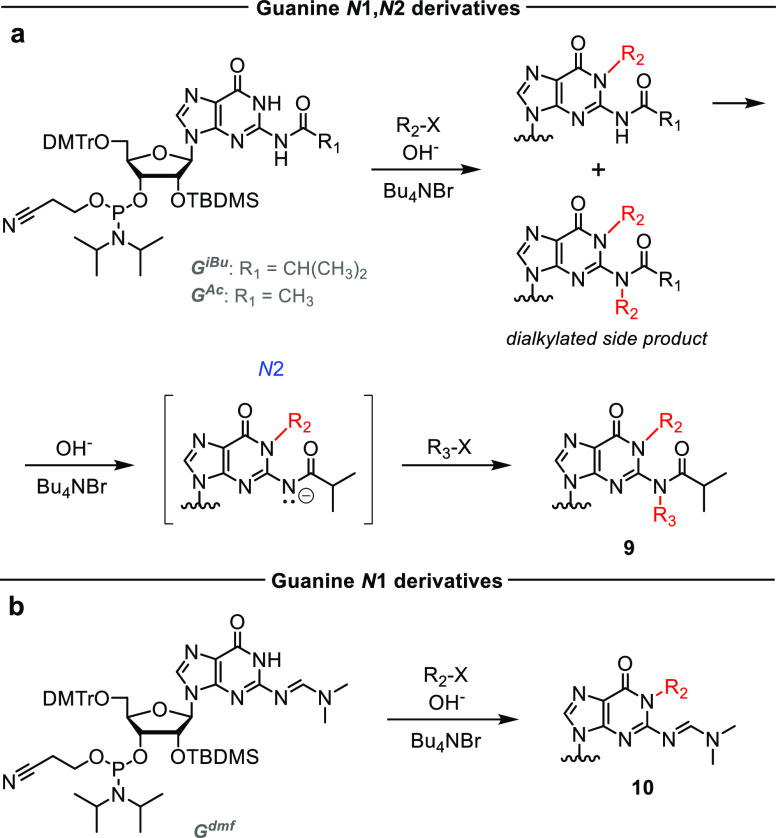
Synthesis of Base-Modified Guanosine 3′-*O*-Phosphoramidites The chemical structures of compounds **9** and **10** are given in Table 1.

The final canonical nucleoside, guanosine, requires that the exocyclic amine group is protected to create phosphoramidite building blocks. *N*2-Acylated guanosines [isobutyryl (*i*Bu), Ac, or Pac derivatives] contain two amide protons that can be abstracted by a base under phase-transfer conditions—one attached to the *N*1 atom and the other attached to *N*2. Although the *N*1 proton is more acidic than the *N*2 proton (*K*_a_ value difference of up to 10 orders of magnitude),^29^ selective methylation at the *N*1 position was challenging. An equimolar amount of MeI was insufficient for full conversion of the starting material, whereas excess MeI resulted in the formation of both mono- and dimethylated products (Scheme 3a). We envisaged that bulkier electrophiles and more hindered *N*2-protecting groups, such as isobutyryl (G^iBu^), would increase the yield of the single substitution reaction and allow asymmetric double substitution. As a proof of concept, we reacted *N*2-isobutyrylguanosine phosphoramidite with 2 equiv of 4-(iodomethyl)phenyl acetate (no reaction occurred with the corresponding chloride) and then with 5 equiv of methyl iodide. Expected *N*1-(4-acetoxybenzyl)-*N*2-methylguanosine derivative **9** was isolated from the reaction mixture in 12% yield. Thus, the use of a base-labile group for *N*1-alkylation provided easy access to oligonucleotides containing *N*2-modified guanosine. To simplify the synthesis of guanosine phosphoramidites monoalkylated selectively at the *N*1 position, we employed another commercially available guanosine amidite protected with the *N*2-[(dimethylamino)methylene] group (G^dmf^), which has only one acidic proton on the nucleobase (Scheme 3b). Methylation in NaOH_aq_/CH_2_Cl_2_ was complete in 25 min, and the desired product **10** was isolated in 82% yield.

Finally, we investigated whether the standard conditions used for oligonucleotide cleavage and deprotection were compatible with the phosphoramidite derivatives (**1–10**) obtained using our approach. Some of these compounds or their analogues were previously synthesized using a standard approach (i.e., base modification, protection, and phosphitylation), and the resulting oligonucleotides did not require any special treatment. These previously evaluated derivatives include the phosphoramidites of m^6^A,^17^*N*4-methylcytidine (m^4^C),^30^*N*3-methyl-uridine (m^3^U),^26^*N*3-(3-amino-3-carboxypropyl)uridine (acp^3^U),^27^*N*6-threonyl-carbamoyladenosine (t^6^A),^7^*N*6-glycinylcarbamoyl-adenosine (g^6^A),^23^*N*4-carbamoylcytidines,^31^ and *N*1-methylguanosine (m^1^G).^32^ For oligonucleotides containing m^1^A, milder conditions (e.g., ammonium hydroxide at 25 °C) should be used because m^1^A is known to undergo Dimroth rearrangement under basic conditions to form m^6^A.^33^ These side reactions could be at least partially limited by carefully selecting the deprotection conditions.^33−35^ For base modifications that were not previously reported (i.e., those in **1b–f**, **8c**, and **9**, as well as those in **3a**,**b**, **4**, and **7**, which contain different protecting groups), we synthesized short oligonucleotides using a solid-phase approach. To ensure that the modified monomers were stable against every reagent used, we incorporated them in the first synthetic cycle, followed by another cycle with the unmodified phosphoramidite. In the literature, there are contradictory reports on the deprotection of m^3^C containing oligonucleotides;^10a,35−37^ therefore, we also included p^m3^CpG in our tests.

As expected, simple *N*6-alkyl adenosine derivatives **1b–d** and **1f** showed properties similar to those of m^6^A and were efficiently deprotected under standard conditions [e.g., 1:1 ammonium hydroxide /methylamine (AMA) mixture at room temperature for 2 h].^38^ The phthalimide protecting group in **1e** has previously been removed from the ammonia-deprotected 2′-*O*-phthalimidopropyl oligonucleotide by additional treatment with methylamine.^39^ Here, we were able to fully deprotect the dinucleotide pA*pG prepared using amidite **1e** in a one-step procedure using AMA at 37 °C for 3 h. *N*6-Carbamoyladenosines derived from compounds **3a**,**b** and **4** were also stable under AMA treatment; however, the ethyl esters of **3b** and **4** were converted into methylamides. This issue can be addressed by using different carboxyl protecting groups (such as trimethylsilylethyl esters)^7^ or different deprotection conditions (e.g., 1 M NaOH).^40^ Deprotection of the dinucleotide containing m^3^C (prepared using phosphoramidite **6a**) with AMA (37 °C, 3 h) resulted in transamination with methylamine to produce *N*3,*N*4-dimethylcytidine derivative p^m3,4^CpG as the only product (as evidenced by MS and NMR analysis; see Supporting Information, compound **21**), which is consistent with the most recent report.^37^ However, the desired p^m3^CpG (Supporting Information, compound **19**) was efficiently prepared using aqueous ammonium hydroxide (RT, overnight) for cleavage and deprotection. Finally, we found that dinucleotide pNpG synthesized using phosphoramidite **9** (m^2^G) is deprotected readily with AMA to produce *N*1-(4-hydroxybenzyl)-*N*2-methylguanosine derivative and then, upon further incubation with AMA at 4 °C (overnight), it undergoes slow elimination of *p*-quinone methide to give U^m2^GU (compound **24**).

**Scheme 4:**
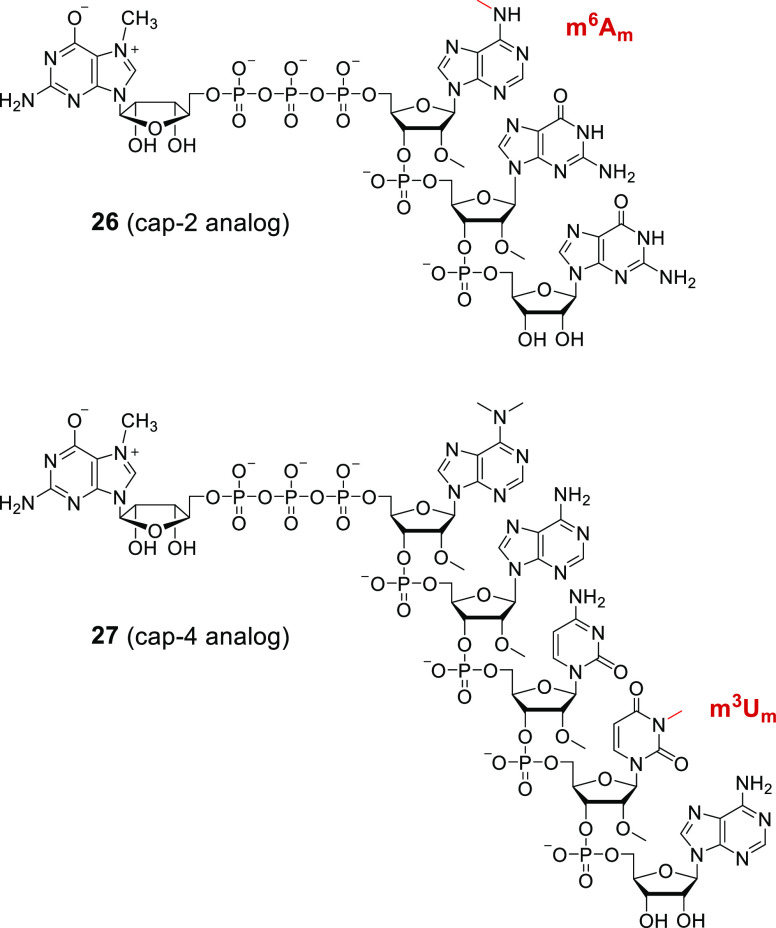
Chemical Structures of m^6^A_m_-Modified cap-2 (Compound **26**) and cap-4 (Compound **27**) Synthesized Using the Phosphoramidites Obtained in This Work

To demonstrate the potential applications of the base-modified nucleoside phosphoramidites obtained in this work, we synthesized oligonucleotide analogues of mRNA 5′ end structures, namely, m^6^A_m_-modified cap-2 found in higher eukaryotes and cap-4 found in *Trypanosoma*.^26,41^ To this end, phosphoramidites **1a** and **8a** were utilized in the solid-phase synthesis of tri- and pentanucleotide 5′-phosphates (p^m6^A_m_pG_m_pG and p^m6,6^A_m_pA_m_pC_m_p^m3^U_m_pA, respectively) and then coupled with 7-methylguanosine 5′-diphosphate using the *P*-imidazolide activation strategy in solution.^6^ The final products **26** and **27** (Scheme 4) were purified by reversed-phase high-performance liquid chromatography and their structures were confirmed by high-resolution mass spectrometry.

## Conclusions

In conclusion, we developed a one-step protocol for synthesizing nucleoside phosphoramidites with *N*-substituted nucleobases, which relies on the deprotonation of the amide moiety under phase-transfer conditions. This procedure was successfully applied to modify all five canonical nucleobases (adenine at the *N*6 and *N*1 positions, cytosine at the *N*4 and *N*3 positions, guanine at the *N*1 position, and thymine and uracil at the *N*3 position) with various alkylating agents (including methyl iodide and primary and secondary halides) in 40–89% yield, starting from commercially available phosphoramidites. Cytidine phosphoramidites were slightly less reactive in PTC alkylation than adenosine derivatives, resulting in the formation of two isomeric products. However, the product ratio was successfully shifted by changing the reaction conditions, allowing either isomer to be obtained as the major product. We also found that adenosine and cytosine phosphoramidites with *N*-protected nucleobases reacted with organic isocyanates (both alkyl and aryl) in the presence of triethylamine to form urea derivatives, which could be further alkylated under phase-transfer conditions to provide *N*-alkyl-*N*-carbamoyl derivatives. Many of the synthesized compounds (or their close structural analogues) are precursors to oligonucleotides containing natural modifications, which are very useful in biological studies on their structure and function. Our synthetic protocol is also suitable for synthesizing functionalized oligonucleotides, providing a powerful tool for obtaining molecular probes, affinity resins, and conjugates for diagnostic and therapeutic applications. This time- and cost-effective approach for phosphoramidite functionalization can also be applied to generate various modified synthetic RNA fragment libraries for high-throughput screening.

## Experimental Section

### General Information

Solvents, chemical reagents, and starting materials were acquired from commercial sources and used without further purification. Commercially available phosphoramidites were purchased from Biosearch Technologies or ChemGenes. Solid supports for oligonucleotide syntheses were purchased from GE Healthcare. DNA synthesis grade acetonitrile (<10 ppm of water) was used for the coupling reaction and for washing the solid support. All work-up and purification procedures were performed with reagent-grade solvents under an ambient atmosphere.

### Analytical and Preparative Chromatography

TLC analysis was performed on precoated silica gel 60 Å on aluminum foil (Sigma-Aldrich) and visualized under a UV lamp (254 nm). The synthesized compounds were isolated by gel chromatography using the Biotage Selekt Flash Purification System with Biotage Sfär Silica cartridges (5, 10 g). Short oligonucleotides (**11**–**26**) were purified by ion-exchange chromatography on DEAE Sephadex A-25 (HCO_3_^–^ form). After loading the column with the reaction mixture and washing it with deionized water, the products were eluted using a linear gradient of triethylammonium bicarbonate (TEAB) in water: 0–0.9 M for dinucleotides (**12**–**16** and **19–22**) and 0–1.2 M for trinucleotides (**11**, **17–18**, and **23–25**). The fractions containing the desired product were combined, concentrated under reduced pressure, and evaporated to dryness with repeated additions of 96% and then 99.8% ethanol to give a white solid of oligonucleotide triethylammonium salt. To facilitate NMR analysis, trinucleotides **11** and **17**–**26** were additionally purified by semi-preparative RP HPLC using a Gemini 5 μm NX-C18 LC column (110 Å, 150 × 10 mm, flow rate 5.0 mL/min) with linear gradient of MeCN in 0.05 M ammonium acetate buffer (pH 5.9) and UV detection at 254 nm. After repeated freeze-drying of the collected fractions, the products were isolated as ammonium salts forming white solids. Analytical RP HPLC was performed on a Gemini 3 μm NX-C18 LC column (110 Å, 150 × 4.6 mm, 3 μm, flow rate 1.0 mL/min) with linear gradient elution with 0.05 M ammonium acetate buffer pH 5.9 (buffer A) and 1:1_v/v_ methanol/buffer A (buffer B): Method A: 0–100% in 15 min; Method B: 0–100% in 7.5 min.

### Compound Characterization

NMR spectra were recorded at 25 °C with a Bruker Avance III HD spectrometer at 500.24 MHz (^1^H NMR) and 202.49 MHz (^31^P NMR) using 5 mm PABBO BB/19F-1H/D Z-GRD probe. The raw NMR data were processed using MestReNova v12.0.2-20910 Software. ^The 1H^ NMR chemical shifts were calibrated to CHCl_3_ (7.260 ppm) or D_2_O (4.790). For the calibration of ^31^P NMR chemical shifts, H_3_PO_4_ was used as an external standard. Signals were assigned based on correlation spectroscopy, heteronuclear single quantum coherence (^1^H-^13^C HSQC, ^1^H-^31^P HSQC), and optionally heteronuclear multiple bond correlation (HMBC) and 2D total correlation (^1^H-^1^H TOCSY) spectra. High-resolution mass spectra (HRMS) were recorded with a LTQ Orbitrap Velos (Thermo Scientific) spectrometer.

### Chemical Syntheses of *N*-Substituted Nucleoside Phosphoramidites

#### General Procedure A (**1a–d**, **4**, **5a**, **6a**,**b**, **8a**, **9**, and **10**)

A nucleoside phosphoramidite (1.0 equiv) and an alkyl halide (2.0–10.0 equiv) were dissolved in dichloromethane (DCM) (to obtain 0.1 M amidite, 1 volume) and mixed with 1 volume of an aqueous solution of Bu_4_NBr (0.1 M, 1.0 equiv) and NaOH (1.0 M). The reaction mixture was stirred vigorously until the starting material was fully consumed, as indicated by TLC analysis. Then, the reaction mixture was partitioned between water (10 volumes) and diethyl ether (10 volumes), and the aqueous phase was extracted with ethyl acetate (10 volumes) three times. The organic layers were combined, dried over anhydrous Na_2_SO_4_, filtered, and concentrated under reduced pressure. The residue was dissolved in DCM containing 0.5%_v/v_ triethylamine, evaporated using silica gel, loaded into a solid sample loader, and purified by flash chromatography.

#### General Procedure B (**1e–g**, **2**, **6c**, and **8b**)

To a 0.1 M solution of a nucleoside phosphoramidite (1.0 equiv) in toluene, an alkyl halide (2.0–20.0 equiv), Bu_4_NBr (1.0 equiv), and an equimolar mixture of ground solid KOH and K_2_CO_3_ (approximately 5 equiv each) were added. The reaction mixture was stirred vigorously until the starting material was fully consumed, as indicated by TLC analysis. Then, the reaction mixture was partitioned between water (10 volumes) and diethyl ether (10 volumes), and the aqueous phase was extracted with ethyl acetate (10 volumes) three times. The organic layers were combined, dried over anhydrous Na_2_SO_4_, filtered, and concentrated under reduced pressure. The residue was dissolved in DCM containing 0.5%_v/v_ triethylamine, evaporated using silica gel, loaded into a solid sample loader, and purified by flash chromatography.

#### General Procedure C (**3a**,**b** and **7**)

A nucleoside phosphoramidite (1.0 equiv), alkyl/aryl isocyanate (8.0–10.0 equiv), and trimethylamine (1.0 equiv) were dissolved in DCM (to obtain 0.1 M amidite). The reaction mixture was stirred vigorously until the starting material was fully consumed, as indicated by TLC analysis. After adding a 33% solution of methylamine in ethanol (15.0 equiv), the reaction mixture was stirred for 30 min to remove the *N*6-acyl protecting group. Then, the reaction mixture was partitioned between water (10 volumes) and diethyl ether (10 volumes), and the aqueous phase was extracted with ethyl acetate (10 volumes) three times. The organic layers were combined, dried over anhydrous Na_2_SO_4_, filtered, and concentrated under reduced pressure. The residue was dissolved in DCM containing 0.5%_v/v_ triethylamine, evaporated using silica gel, loaded into a solid sample loader, and purified by flash chromatography.

##### *N*6-Methyladenosine Phosphoramidite [5′-*O*-DMT-2′-*O*-Me-^m6^A^Pac^] (**1a**)

Compound **1a** was prepared according to procedure **A** using 1.00 g (1.09 mmol) of 5′-*O*-DMT-2′-*O*-Me-A^Pac^ phosphoramidite and 272 μL (4.36 mmol, 4 equiv) of methyl iodide. The reaction was quenched after 30 min, and the product was isolated by flash chromatography (0 → 50% ethyl acetate in *n*-hexane with 0.5%_v/v_ TEA in 30 min, 80 mL/min, Biotage Sfär HC 25 g column). The diastereomers were characterized separately and then combined to afford **1a** (804 mg, 0.863 mmol, 79%) as a white solid. Diastereomer 1: TLC (hexane/ethyl acetate 1:1): *R*_f_ = 0.30; ^1^H NMR (500 MHz, CDCl_3_, 25 °C): δ = 8.61 (s, 1H, H8), 8.26 (s, 1H, H2), 7.44 (m, 2H, *Ar*H-2,6_Ph-DMTr_), 7.35–7.16 (m, 9H, *Ar*H), 6.90 (m, 1H, *Ar*H-4_Pac_), 6.82 (m, 4H, *Ar*H-3,5_MeOPh-DMTr_), 6.71 (m, 2H, *Ar*H-2,6_Pac_), 6.16 (m, 1H, H1′), 5.14 (s, 2H, CH_2Pac_), 4.68 (m, 1H, H3′), 4.58 (m, 1H, H2′), 4.43 (m, 1H, H4′), 3.78 (s, 6H, 2 × OCH_3DMTr_), 3.75 (s, 3H, CH_3N6-Me_), 3.73–3.55 (m, 5H, OC*H_2_*CH_2_CN, 2 × CH_iPr_, H5′), 3.49 (s, 3H, 2′-*O*-CH_3_), 3.39 (dd, ^2^*J*_H,H_ = 10.7 Hz, ^3^*J*_H,H_ = 3.9 Hz, 1H, H5″), 2.38 (t, ^3^*J*_H,H_ = 6.7 Hz, 2H, OCH_2_C*H_2_*CN), 1.23–1.18 (m, 12H, CH_3iPr_) ppm; ^31^P NMR (202.5 MHz, CDCl_3_, 25 °C): δ = 151.0 (m, 1P, P) ppm; HRMS (ESI) *m*/*z* calcd for C_50_H_59_N_7_O_9_P^+^, [M + H]^+^: 932.41064, found: 932.41001; Diastereomer 2: TLC (hexane/ethyl acetate 1:1): *R*_f_ = 0.37; ^1^H NMR (500 MHz, CDCl_3_, 25 °C): δ = 8.60 (s, 1H, H8), 8.20 (s, 1H, H2), 7.42 (d, ^3^*J*_H,H_ = 7.2 Hz, 2H, *Ar*H-2,6_Ph-DMTr_), 7.32 (d, ^3^*J*_H,H_ = 8.8 Hz, 4H, *Ar*H-2,6_MeOPh-DMTr_), 7.26 (t, ^3^*J*_H,H_ = 7.2 Hz, 2H, *Ar*H-3,5_Ph-DMTr_), 7.23–7.16 (m, 3H, *Ar*H-4_Ph-DMTr_, *Ar*H-3,5_Pac_), 6.90 (t, ^3^*J*_H,H_ = 7.3 Hz, 1H, *Ar*H-4_Pac_), 6.80 (d, ^3^*J*_H,H_ = 8.8 Hz, 4H, *Ar*H-3,5_MeOPh-DMTr_), 6.71 (d, ^3^*J*_H,H_ = 7.6 Hz, 2H, *Ar*H-2,6_Pac_), 6.18 (d, ^3^*J*_H,H_ = 5.4 Hz, 1H, H1′), 5.14 (s, 2H, CH_2Pac_), 4.64 (m, 1H, H2′), 4.60 (m, 1H, H3′), 4.37 (m, 1H, H4′), 3.90 (m, 2H, OC*H_2_*CH_2_CN), 3.78 (s × 2, 6H, OCH_3DMTr_), 3.75 (s, 3H, CH_3N6-Me_), 3.61 (m, 2H, CH_iPr_), 3.53 (dd, ^2^*J*_H,H_ = 10.7 Hz, ^3^*J*_H,H_ = 3.7 Hz, 1H, H5′), 3.50 (s, 3H, 2′-*O*-CH_3_), 3.37 (dd, ^2^*J*_H,H_ = 10.7 Hz, ^3^*J*_H,H_ = 4.2 Hz, 1H, H5″), 2.64 (t, ^3^*J*_H,H_ = 6.3 Hz, 2H, OCH_2_C*H_2_*CN), 1.19 (d, ^3^*J*_H,H_ = 6.8 Hz, 6H, CH_3iPr_), 1.09 (d, ^3^*J*_H,H_ = 6.8 Hz, 6H, CH_3iPr_) ppm; ^31^P NMR (202.5 MHz, CDCl_3_, 25 °C): δ = 150.4 (m, 1P, P) ppm; HRMS (ESI) *m*/*z*: [M + H]^+^ calcd for C_50_H_59_N_7_O_9_P^+^ 932.41064, found: 932.41006; diastereomeric mixture: ^13^C{^1^H} NMR (126 MHz, CDCl_3_, 25 °C): δ = 170.7, 170.6, 158.8, 158.0, 158.0, 153.0, 152.9, 152.9, 152.8, 151.6, 151.6, 144.6, 144.5, 142.5, 142.5, 135.7, 135.6, 130.3, 130.2, 129.5, 128.4, 128.3, 128.0, 127.2, 127.2, 126.0, 126.0, 121.4, 121.4, 117.8, 117.6, 114.6, 114.6, 113.3, 87.1, 87.0, 86.9, 86.8, 84.0, 84.0, 82.6, 82.5, 82.1, 82.1, 77.4, 77.4, 77.2, 76.9, 71.3, 71.2, 70.9, 70.7, 68.9, 63.1, 62.7, 59.0, 59.0, 58.9, 58.9, 58.5, 58.5, 58.1, 58.0, 55.4, 55.4, 43.6, 43.5, 43.4, 43.3, 35.1, 24.8, 24.8, 24.7, 24.7, 24.7, 20.5, 20.5, 20.7, 20.3 ppm.

##### *N*6-Isopentenyladenosine Phosphoramidite [5′-*O*-DMT-2′-*O*-TBDMS-i^6^A^Ac^] (**1b**)

Compound **1b** was prepared according to procedure **A** using 250 mg (0.270 mmol) of 5′-*O*-DMT-2′-*O*-TBDMS-A^Ac^ phosphoramidite and 156 μL (1.35 mmol, 5.0 equiv) of isopentenyl bromide. The reaction was quenched after 35 min, and the product was isolated by flash chromatography (0 → 60% ethyl acetate in *n*-hexane with 0.5%_v/v_ TEA in 35 min, 40 mL/min, Biotage Sfär HC 10g column) to afford a mixture of diastereomers **1b** (216 mg, 0.217 mmol, 80%) as a white solid. TLC (hexane/ethyl acetate 1:1): *R*_f_ = 0.51; ^1^H NMR (500 MHz, CDCl_3_, 25 °C): δ = 8.69 (s, 1H, H8), 8.67 (s, 1H, H8), 8.30 (s, 1H, H2), 8.27 (s, 1H, H2), 7.47 (m, 4H, *Ar*H-2,6_Ph-DMTr_), 7.36 (m, 8H, *Ar*H-2,6_MeOPh-DMTr_), 7.30–7.20 (m, 6H, *Ar*H-3,4,5_Ph-DMTr_), 6.82 (m, 8H, *Ar*H-3,5_MeOPh-DMTr_), 6.13 (d, ^3^*J*_H,H_ = 6.7 Hz, 1H, H1′), 6.06 (d, ^3^*J*_H,H_ = 6.4 Hz, 1H, H1′), 5.22 (m, 2H, 2 × C=CH_N6-isopent._), 5.04 (m, 2H, 2 × H2′), 4.84 (m, 4H, 2 × CH_2N6-isopent._), 4.46 (m, 1H, H4′), 4.43–4.36 (m, 3H, 2 × H3′, H4′), 3.97 (m, 1H, OC*H_2_*CH_2_CN), 3.89 (m, 1H, OC*H_2_*CH_2_CN), 3.79 (overlapped s, 12H, 4 × OCH_3DMTr_), 3.71–3.53 (m, 8H, OC*H_2_*CH_2_CN, 2 × H5′, 4 × CH_iPr_), 3.36 (m, 1H, H5″), 3.33 (dd, ^2^*J*_H,H_ = 10.7 Hz, ^3^*J*_H,H_ = 3.8 Hz, 1H, H5″), 2.66 (m, 2H, OCH_2_C*H_2_*CN), 2.31 (m, 2H, OCH_2_C*H_2_*CN), 2.21 (s, 3H, CH_3N6-Ac_), 2.20 (s, 3H, CH_3N6-Ac_), 1.57 (s, 6H, 2 × CH_3N6-isopent._), 1.56 (s, 6H, 2 × CH_3N6-isopent._), 1.22–1.16 (m, 18H, 6 × CH_3iPr_), 1.06 (d, ^3^*J*_H,H_ = 6.8 Hz, 6H, 2 × CH_3iPr_), 0.72 (s, 18H, 2 × *t*Bu_TBDMS_), −0.03 (s, 3H, CH_3TBDMS_), −0.05 (s, 3H, CH_3TBDMS_), −0.24 (s, 6H, 2 × CH_3TBDMS_) ppm; ^13^C{^1^H} NMR (126 MHz, CDCl_3_, 25 °C): δ = 171.2, 158.7, 158.7, 153.7, 153.2, 152.2, 144.7, 144.6, 143.0, 142.9, 136.0, 135.8, 135.8, 135.6, 135.6, 130.3, 130.3, 130.2, 130.2, 128.4, 128.2, 128.1, 128.1, 128.0, 128.0, 127.1, 120.2, 117.4, 113.4, 113.3, 113.3, 88.4, 88.1, 87.0, 86.8, 84.6, 74.8, 74.8, 73.6, 73.6, 63.5, 63.3, 57.8, 57.6, 55.4, 55.4, 45.5, 45.5, 43.6, 43.5, 43.1, 43.0, 25.8, 25.7, 25.7, 24.9, 24.9, 24.8, 24.8, 24.7, 24.2, 20.6, 20.6, 20.3, 20.2, 18.1, 18.0, 18.0, −4.5, −4.6, −5.2 ppm; ^31^P NMR (202.5 MHz, CDCl_3_, 25 °C): δ = 151.2 (s, 1P, P), 149.1 (s, 1P, P) ppm; HRMS (ESI) *m*/*z*: [M + H]^+^ calcd for C_53_H_73_N_7_O_8_PSi^+^ 994.50220, found: 994.50275;

##### *N*6-Benzyladenosine Phosphoramidite [5′-*O*-DMT-2′-*O*-Me-Bn^6^A^Pac^] (**1c**)

Compound **1c** was prepared according to procedure **A** using 1.00 g (1.09 mmol) of 5′-*O*-DMT-2′-*O*-Me-A^Pac^ phosphoramidite and 162 μL (1.36 mmol, 1.25 equiv) of benzyl bromide. The reaction was quenched after 35 min, and the product was isolated by flash chromatography (0 → 50% ethyl acetate in *n*-hexane with 0.5%_v/v_ TEA in 30 min, 80 mL/min, Biotage Sfär HC 25g column) to afford a mixture of diastereomers of **1c** (646 mg, 0.640 mmol, 59%) as a white solid. TLC (hexane/ethyl acetate 1:1): *R*_f_ = 0.58; ^1^H NMR (500 MHz, CDCl_3_, 25 °C): 8.60 (s, 1H, H8), 8.58 (s, 1H, H8), 8.26 (s, 1H, H2), 8.19 (s, 1H, H2), 7.42 (m, 4H, *Ar*H-2,6_Ph-DMTr_), 7.35–7.12 (m, 28H, *Ar*H), 6.89 (m, 2H, *Ar*H), 6.81 (m, 8H, *Ar*H), 6.63 (m, 4H, *Ar*H), 6.15 (d, ^3^*J*_H,H_ = 5.2 Hz, 1H, H1′), 6.13 (d, ^3^*J*_H,H_ = 5.1 Hz, 1H, H1′), 5.65 (s, 4H, 2 × C*H*_2N6-Bn_), 5.13 (s, 4H, 2 × C*H*_2Pac_), 4.66 (m, 1H, H3′), 4.62–4.57 (m, 2H, H2′, H3′), 4.54 (m, 1H, H2′), 4.41 (m, 1H, H4′), 4.35 (m, 1H, H4′), 3.92 (m, 1H, OC*H_2_*CH_2_CN), 3.86 (m, 1H, OC*H_2_*CH_2_CN), 3.78–3.76 (overlapped s, 12H, 4 × OCH_3DMTr_), 3.73–3.55 (m, 7H, OC*H_2_*CH_2_CN, H5′, 4 × CH_iPr_), 3.52 (dd, ^2^*J*_H,H_ = 10.6 Hz, ^3^*J*_H,H_ = 3.8 Hz, H5′), 3.48 (s, 3H, CH_32′-O_), 3.47 (s, 3H, CH_32′-O_), 3.36 (m, 2H, 2 × H5″), 2.63 (t, ^3^*J*_H,H_ = 6.3 Hz, 2H, OCH_2_C*H_2_*CN), 2.37 (t, ^3^*J*_H,H_ = 6.3 Hz, 2H, OCH_2_C*H_2_*CN), 1.21–1.17 (m, 18H, 6 × CH_3iPr_), 1.08 (d, ^3^*J*_H,H_ = 6.3 Hz, 3H, CH_3iPr_) ppm; ^13^C{^1^H} NMR (126 MHz, CDCl_3_, 25 °C): δ = 170.7, 170.7, 158.8, 158.8, 157.9, 157.8, 152.9, 152.8, 152.2, 152.1, 151.8, 151.8, 144.6, 144.5, 142.6, 142.6, 137.4, 137.4, 135.7, 135.7, 135.6, 130.3, 130.3, 130.3, 129.5, 128.4, 128.4, 128.3, 128.0, 128.0, 128.0, 127.3, 127.2, 127.2, 126.6, 126.6, 121.5, 121.5, 117.8, 117.5, 117.4, 114.5, 114.5, 113.3, 87.1, 86.9, 86.9, 86.8, 84.0, 83.9, 82.5, 82.5, 71.3, 71.2, 70.7, 68.9, 68.9, 63.1, 62.6, 59.0, 58.9, 58.9, 58.9, 58.5, 58.5, 58.1, 58.0, 55.4, 55.4, 50.1, 43.6, 43.5, 43.4, 43.3, 24.8, 24.7, 24.7, 20.5, 20.5, 20.4, 20.3, 15.4.ppm; ^31^P NMR (202.5 MHz, CDCl_3_, 25 °C): δ = 151.0 (s, 1P, P), 150.4 (s, 1P, P) ppm; HRMS (ESI) *m*/*z*: [M + H]^+^ calcd for C_56_H_63_N_7_O_9_P^+^ 1008.44194, found: 1008.44298.

##### *N*6-Hexynyladenosine Phosphoramidite [5′-*O*-DMT-2′-*O*-Me-hex^6^A^Pac^] (**1d**)

Compound **1d** was prepared according to procedure **A** using 315 mg (0.343 mmol) of 5′-*O*-DMT-2′-*O*-Me-A^Pac^ phosphoramidite and 280 μL (2.06 mmol, 6 equiv) of 6-iodohex-1-yn. The reaction was quenched after 60 min, and the product was isolated by flash chromatography (0 → 50% ethyl acetate in *n*-hexane with 0.5%_v/v_ TEA in 35 min, 40 mL/min, Biotage Sfär HC 10g column) to afford a mixture of diastereomers of **1d** (192 mg, 0.192 mmol, 56%) as a white solid. TLC (hexane/ethyl acetate 1:1): *R*_f_ = 0.47; ^1^H NMR (500 MHz, CDCl_3_, 25 °C): δ = 8.63 (s, 1H, H8), 8.62 (s, 1H, H8), 8.26 (s, 1H, H2), 8.19 (s, 1H, H2), 7.46–7.14 (m, 22H, *Ar*H), 6.91–6.79 (m, 10H, *Ar*H), 6.65 (d, ^3^*J*_H,H_ = 5.4 Hz, 4H, *Ar*H), 6.18 (d, ^3^*J*_H,H_ = 5.3 Hz, 1H, H1′), 6.16 (d, ^3^*J*_H,H_ = 5.0 Hz, 1H, H1′), 5.07 (s, 4H, 2 × C*H*_2Pac_), 4.69 (m, 1H, H3′), 4.64 (m, 1H, H3′), 4.58 (m, 2H, 2 × H2′), 4.42 (m, 2H, 2 × H4′), 4.36 (m, 4H, 2 × CH_2C6-hex._), 3.93 (m, 1H, OC*H_2_*CH_2_CN), 3.86 (m, 1H, OC*H_2_*CH_2_CN), 3.78 (s, 12H, 4 × OCH_3DMTr_), 3.74–3.55 (m, 6H, OC*H_2_*CH_2_CN, 2 × H5′, 2 × CH_iPr_), 3.50 (s, 6H, 2 × CH_32′-O_), 3.50–3.46 (m, 2H, 2 × CH_iPr_), 3.38 (m, 2H, 2 × H5″), 2.64 (t, ^3^*J*_H,H_ = 6.3 Hz, 2H, OCH_2_C*H_2_*CN), 2.38 (t, ^3^*J*_H,H_ = 6.3 Hz, 2H, OCH_2_C*H_2_*CN), 2.14 (td, ^3^*J*_H,H_ = 7.0 Hz, ^4^*J*_H,H_ = 2.4 Hz, 4H, 2 × CH_2C3-hex._), 1.85 (t, ^4^*J*_H,H_ = 2.4 Hz, 2H, 2 × C≡CH_C1-hex._), 1.71 (m, 4H, 2 × CH_2C5-hex._), 1.53 (m, 4H, 2 × CH_2C4-hex._), 1.20 (m, 18H, 6 × CH_3iPr_), 1.09 (d, ^3^*J*_H,H_ = 6.8 Hz, 6H, 2 × CH_3iPr_) ppm; ^13^C{^1^H} NMR (126 MHz, CDCl_3_, 25 °C): δ = 170.4, 170.4, 158.8, 157.9, 152.9, 152.9, 152.4, 152.3, 151.8, 144.6, 142.6, 142.6, 135.7, 135.7, 135.6, 130.3, 130.3, 129.5, 128.4, 128.3, 128.0, 127.2, 126.5, 126.5, 121.4, 117.6, 114.5, 113.3, 87.0, 86.9, 86.9, 86.8, 84.3, 84.0, 84.0, 82.1, 82.0, 71.3, 71.2, 68.9, 68.5, 66.0, 62.6, 58.5, 58.5, 58.1, 57.9, 55.4, 55.4, 46.8, 43.6, 43.5, 43.4, 43.3, 27.7, 25.7, 24.8, 24.7, 24.7, 20.4, 20.3, 18.2, 15.4 ppm; ^31^P NMR (202.5 MHz, CDCl_3_, 25 °C): δ = 151.0 (s, 1P, P), 150.4 (s, 1P, P) ppm; HRMS (ESI) *m*/*z*: [M + H]^+^ calcd for C_55_H_65_N_7_O_9_P^+^ 998.45759, found: 998.45670.

##### *N*6-(3-Phthalimidopropyl)adenosine Phosphoramidite [5′-*O*-DMT-2′-*O*-Me-PhthNp^6^A^Pac^] (**1e**)

Compound **1e** was prepared according to procedure **B** using 943 mg (1.03 mmol) of 5′-*O*-DMT-2′-*O*-Me-A^Pac^ phosphoramidite and 549 mg (2.05 mmol, 2.0 equiv) of 3-phthalimidopropyl bromide. The reaction was quenched after 60 min, and the product was isolated by flash chromatography (0 → 50% ethyl acetate in *n*-hexane with 0.5%_v/v_ TEA in 35 min, 40 mL/min, Biotage Sfär HC 10g column) to afford a mixture of diastereomers of **1e** (601 mg, 0.544 mmol, 48%) as a white solid. TLC (hexane/ethyl acetate 1:1): *R*_f_ = 0.31; ^1^H NMR (500 MHz, CDCl_3_, 25 °C): δ = 8.54 (s, 1H, H8), 8.52 (s, 1H, H8), 7.89 (s, 1H, H2), 7.80 (s, 1H, H2), 7.76 (m, 4H, *Ar*H_Phth-α_), 7.62 (m, 4H, *Ar*H_Phth.β_), 7.44 (m, 4H, *Ar*H), 7.37–7.14 (m, 18H, *Ar*H), 6.89 (m, 2H, *Ar*H), 6.81 (m, 8H, *Ar*H), 6.68 (d, ^3^*J*_H,H_ = 8.2 Hz, 4H, *Ar*H), 6.08 (d, ^3^*J*_H,H_ = 5.1 Hz, 2H, 2 × H1′), 5.12 (s, 4H, 2 × C*H*_2Pac_), 4.63 (m, 2H, H3′, H2′), 4.56 (m, 2H, H3′, H2′), 4.42 (m, 5H, 2 × CH_2C3_N6-prop._, H4′), 4.35 (m, 1H, H4′), 3.89 (m, 2H, OC*H_2_*CH_2_CN), 3.77 (s, 12H, 4 × OCH_3DMTr_), 3.75 (m, 4H, 2 × CH_2C1_N6-prop._), 3.72–3.48 (m, 8H, OC*H_2_*CH_2_CN, 2 × H5′, 4 × CH_iPr_), 3.47 (s, 6H, 2 × CH_32′-O_), 3.35 (m, 2H, 2 × H5″), 2.64 (t, ^3^*J*_H,H_ = 5.9 Hz, 2H, OCH_2_C*H_2_*CN), 2.38 (t, ^3^*J*_H,H_ = 6.3 Hz, 2H, OCH_2_C*H_2_*CN), 2.11 (p, ^3^*J*_H,H_ = 6.9 Hz, 4H, 2 × CH_2C2_N6-prop_), 1.20 (m, 18H, 6 × CH_3iPr_), 1.09 (d, ^3^*J*_H,H_ = 6.7 Hz, 6H, 2 × CH_3iPr_) ppm; ^13^C{^1^H} NMR (126 MHz, CDCl_3_): δ = 170.6, 170.6, 168.4, 168.4, 158.7, 158.7, 157.9, 157.9, 152.9, 152.8, 152.0, 152.0, 151.6, 151.6, 144.7, 144.6, 142.4, 142.3, 135.7, 135.7, 135.7, 133.9, 133.9, 132.3, 130.3, 130.3, 130.2, 129.5, 128.3, 128.3, 128.0, 127.1, 127.1, 125.8, 125.7, 123.2, 121.4, 121.4, 117.8, 117.5, 114.5, 113.3, 87.0, 86.9, 86.8, 86.7, 84.0, 83.9, 82.1, 82.1, 81.8, 81.7, 71.4, 71.2, 70.8, 70.7, 69.1, 69.1, 63.2, 62.7, 59.0, 58.9, 58.9, 58.9, 58.5, 58.5, 58.1, 58.0, 55.4, 55.3, 45.2, 43.5, 43.4, 43.4, 43.3, 35.8, 35.8, 27.7, 24.8, 24.7, 24.7, 24.7, 20.5, 20.5, 20.3, 20.3 ppm; ^31^P NMR (202.5 MHz, CDCl_3_, 25 °C): δ = 151.0 (s, 1P, P), 150.4 (s, 1P, P) ppm; HRMS (ESI) *m*/*z*: [M + H]^+^ calcd for C_60_H_66_N_8_O_11_P^+^ 1105.45832, found: 1105.46053.

##### *N*6-Dsopropyladenosine Phosphoramidite [5′-*O*-DMT-2′-*O*-Me-iPr^6^A^Pac^] (**1f**)

Compound **1f** was prepared according to procedure **B** using 250 mg (0.272 mmol) of 5′-*O*-DMT-2′-*O*-Me-A^Pac^ phosphoramidite and 544 μL (5.45 mmol, 20 equiv) of 2-iodopropane. The reaction was quenched after 2.5 h, and the product was isolated by flash chromatography (0 → 100% ethyl acetate in *n*-hexane with 0.5%_v/v_ TEA in 35 min, 40 mL/min, Biotage Sfär HC 10g column) to afford a mixture of diastereomers of **1f** (117 mg, 0.122 mmol, 45%) as a white solid. TLC (hexane/ethyl acetate 1:1): *R*_f_ = 0.60; ^1^H NMR (500 MHz, CDCl_3_, 25 °C): δ = 8.74 (s, 1H, H8), 8.72 (s, 1H, H8), 8.28 (s, 1H, H2), 8.21 (s, 1H, H2), 7.43 (m, 4H, *Ar*H-2,6_Ph-DMTr_), 7.33 (m, 8H, *Ar*H-2,6_MeOPh-DMTr_), 7.29–7.20 (m, 6H, *Ar*H-3,4,5_Ph-DMTr_), 7.13 (m, 4H, *Ar*H-3,5_Ph-Pac_), 6.86 (m, 2H, *Ar*H-4_Ph-Pac_), 6.81 (m, 8H, *Ar*H-3,5_MeOPh-DMTr_), 6.56 (m, 4H, *Ar*H-2,6_Ph-Pac_), 6.17 (d, ^3^*J*_H,H_ = 5.3 Hz, 1H, H1′), 6.15 (d, ^3^*J*_H,H_ = 5.1 Hz, 1H, H1′), 4.93 (m, 2H, 2 × CH_N6_iPr_), 4.69 (m, 1H, H3′), 4.68 (m, 4H, 2 × CH_2Pac_), 4.66–4.57 (m, 3H, H3′, 2 × H2′), 4.43 (m, 1H, H4′), 4.37 (m, 1H, H4′), 3.92 (m, 1H, OC*H_2_*CH_2_CN), 3.85 (m, 1H, OC*H_2_*CH_2_CN), 3.78 (s, 3H, OCH_3DMTr_), 3.78 (s, 3H, OCH_3DMTr_), 3.77 (s, 3H, OCH_3DMTr_), 3.77 (s, 3H, OCH_3DMTr_), 3.73–3.51 (m, 8H, OC*H_2_*CH_2_CN, 2 × H5′, 4 × CH_iPr_), 3.50 (s, 3H, CH_32′-O_), 3.50 (s, 3H, CH_32′-O_), 3.38 (m, 2H, 2 × H5″), 2.63 (t, ^3^*J*_H,H_ = 6.3 Hz, 2H, OCH_2_C*H_2_*CN), 2.37 (t, ^3^*J*_H,H_ = 6.2 Hz, 2H, OCH_2_C*H_2_*CN), 1.37 (m, 12H, 4 × CH_3iPr_), 1.23–1.17 (m, 18H, 6 × CH_3iPr_), 1.09 (d, ^3^*J*_H,H_ = 6.7 Hz, 6H, 2 × CH_3iPr_) ppm; ^31^P NMR (202.5 MHz, CDCl_3_, 25 °C): δ = 151.0 (s, 1P, P), 150.4 (s, 1P, P) ppm; HRMS (ESI) *m*/*z*: [M + H]^+^ calcd for C_52_H_63_N_7_O_9_P^+^ 960.44194, found: 960.44371.

##### *N*6-Methyladenosine Phosphoramidite [5′-*O*-DMT-2′-*O*-TBDMS-m^6^A^Bz^] (**1g**) and *N*1-Methyladenosine Phosphoramidite [5′-*O*-DMT-2′-*O*-TBDMS-m^1^A^Bz^] (**2**)

Compounds **1g** and **2** were prepared according to procedure **B** using 500 mg (0.506 mmol) of 5′-*O*-DMT-2′-*O*-TBDMS-A^Bz^ phosphoramidite and 315 μL (5.06 mmol, 10 equiv) of methyl iodide. The reaction was quenched after 30 min, and the products were isolated and separated by flash chromatography (0 → 50% ethyl acetate in *n*-hexane with 0.5%_v/v_ TEA in 35 min, 40 mL/min, Biotage Sfär HC 10 g column) to afford a mixture of diastereomers of **1g** (312 mg, 0.311 mmol, 62%) and a mixture of diastereomers of **2** (146 mg, 0.146 mmol, 29%) as white solids.

m^6^A^Bz^: TLC (hexane/ethyl acetate 1:1): *R*_f_ = 0.50; ^1^H NMR (500 MHz, CDCl_3_, 25 °C): δ = 8.44 (s, 1H, H8), 8.42 (s, 1H, H8), 8.14 (s, 1H, H2), 8.11 (s, 1H, H2), 7.43 (m, 8H, *Ar*H), 7.33 (m, 8H, *Ar*H-2,6_MeOPh-DMTr_), 7.24 (m, 8H, *Ar*H), 7.13 (m, 4H, *Ar*H), 6.80 (m, 8H, *Ar*H-3,5_MeOPh-DMTr_), 6.03 (d, ^3^*J*_H,H_ = 6.7 Hz, 1H, H1′), 5.97 (d, ^3^*J*_H,H_ = 6.6 Hz, 1H, H1′), 4.99 (m, 2H, 2 × H2′), 4.42 (m, 1H, H4′), 4.35 (m, 3H, 2 × H3′, H4′), 3.95 (m, 1H, OC*H_2_*CH_2_CN), 3.86 (m, 1H, OC*H_2_*CH_2_CN), 3.78 (m, 18H, 4 × OCH_3DMTr_, 2 × CH_3N6-Me_), 3.71–3.50 (m, 8H, OC*H_2_*CH_2_CN, 2 × H5′, 4 × CH_iPr_), 3.30 (m, 2H, 2 × H5″), 2.63 (m, 2H, OCH_2_C*H_2_*CN), 2.29 (m, 2H, OCH_2_C*H_2_*CN), 1.20–1.15 (m, 18H, 6 × CH_3iPr_), 1.04 (d, ^3^*J*_H,H_ = 6.8 Hz, 6H, 2 × CH_3iPr_), 0.72 (s, 18H, 2 × *t*Bu_TBDMS_), −0.07 (s, 3H, CH_3TBDMS_), −0.09 (s, 3H, CH_3TBDMS_), −0.32 (s, 3H, CH_3TBDMS_), −0.33 (s, 3H, CH_3TBDMS_) ppm; ^13^C{^1^H} NMR (126 MHz, CDCl_3_, 25 °C): δ = 172.3, 158.7, 155.1, 155.1, 152.9, 152.9, 152.0, 144.7, 144.6, 142.7, 142.6, 136.4, 135.9, 135.8, 135.7, 135.6, 130.7, 130.3, 130.2, 130.2, 130.2, 128.8, 128.3, 128.2, 128.1, 128.0, 128.0, 128.0, 127.1, 126.9, 126.8, 117.7, 117.4, 113.4, 113.3, 113.3, 88.2, 88.0, 86.9, 86.7, 84.6, 84.2, 84.2, 75.2, 75.2, 74.6, 74.5, 73.6, 73.6, 72.8, 72.7, 63.4, 63.3, 59.0, 58.9, 57.8, 57.6, 55.4, 55.4, 43.6, 43.5, 43.1, 43.0, 36.0, 25.7, 25.7, 24.9, 24.9, 24.8, 24.8, 24.8, 24.7, 24.7, 20.6, 20.6, 20.2, 20.2, 18.0, 18.0, 15.4, −4.5, −4.5, −5.1, −5.1 ppm; ^31^P NMR (202.5 MHz, CDCl_3_, 25 °C): δ = 151.2 (s, 1P, P), 149.0 (s, 1P, P) ppm; HRMS (ESI) *m*/*z* calcd for C_54_H_69_N_7_O_8_PSi^+^, [M + H]^+^: 1002.47090, found: 1002.47145.

m^1^A^Bz^: TLC (hexane/ethyl acetate 1:1): *R*_f_ = 0.22; ^1^H NMR (500 MHz, CDCl_3_, 25 °C): δ = 8.16 (m, 4H, *Ar*H-2,6_Bz_), 7.85 (s, 1H, H8), 7.83 (s, 1H, H8), 7.82 (s, 1H, H2), 7.80 (s, 1H, H2), 7.50–7.48 (m, 10H, 2 × *Ar*H-3,4,5_Bz_, 2 × *Ar*H-2,6_Ph-DMTr_), 7.32 (m, 8H, 2 × *Ar*H-2,6_MeOPh-DMTr_), 7.26–7.16 (m, 6H, 2 × *Ar*H-3,4,5_Ph-DMTr_), 6.79 (m, 8H, 2 × *Ar*H-3,5_MeOPh-DMTr_), 5.91 (d, ^3^*J*_H,H_ = 6.1 Hz, 1H, H1′), 5.84 (d, ^3^*J*_H,H_ = 6.0 Hz, 1H, H1′), 4.85 (m, 1H, H2′), 4.81 (m, 1H, H2′), 4.38 (m, 1H, H4′), 4.28 (m, 3H, 2 × H3′, H4′), 3.88 (m, 2H, OC*H_2_*CH_2_CN), 3.77 (s, 6H, 2 × OCH_3DMTr_), 3.76 (s, 6H, 2 × OCH_3DMTr_), 3.71 (s, 3H, CH_3N1-Me_), 3.71 (s, 3H, CH_3N1-Me_), 3.64–3.40 (m, 8H, OC*H_2_*CH_2_CN, 2 × H5′, 4 × CH_iPr_), 3.22 (m, 2H, 2 × H5″), 2.63 (t, ^3^*J*_H,H_ = 6.5 Hz, 2H, OCH_2_C*H_2_*CN), 2.25 (m, 2H, OCH_2_C*H_2_*CN), 1.18–1.12 (m, 18H, 6 × CH_3iPr_), 1.00 (d, ^3^*J*_H,H_ = 6.7 Hz, 6H, 2 × CH_3iPr_), 0.79 (s, 9H, *t*Bu_TBDMS_), 0.79 (s, 9H, *t*Bu_TBDMS_), −0.01 (s, 3H, CH_3TBDMS_), −0.02 (s, 3H, CH_3TBDMS_), −0.14 (s, 3H, CH_3TBDMS_), −0.14 (s, 3H, CH_3TBDMS_) ppm; ^13^C{^1^H} NMR (126 MHz, CDCl_3_, 25 °C): δ = 177.2, 177.1, 171.3, 158.7, 158.6, 147.8, 147.7, 146.8, 146.7, 145.8, 144.8, 144.7, 139.0, 138.7, 136.0, 136.0, 135.9, 135.9, 135.7, 135.7, 131.9, 130.3, 130.3, 130.2, 129.9, 129.9, 128.3, 128.2, 128.1, 128.0, 128.0, 127.0, 122.9, 122.7, 117.7, 117.4, 113.3, 113.3, 113.3, 88.5, 88.1, 86.7, 86.5, 84.1, 83.7, 83.6, 75.6, 74.8, 74.7, 73.3, 73.2, 72.8, 72.7, 63.5, 63.4, 60.5, 59.0, 58.8, 57.8, 57.7, 55.4, 55.4, 43.6, 43.5, 43.1, 43.0, 37.0, 25.8, 25.8, 24.9, 24.8, 24.8, 24.8, 24.7, 24.7, 24.6, 21.2, 20.5, 20.5, 20.2, 20.1, 18.1, 18.0, 14.3, −4.5, −4.5, −4.5, −4.6, −4.8, −4.9 ppm; ^31^P NMR (202.5 MHz, CDCl_3_, 25 °C): δ = 150.9 (s, 1P, P), 149.2 (s, 1P, P) ppm; HRMS (ESI) *m*/*z*: [M + H]^+^ calcd for C_54_H_69_N_7_O_8_PSi^+^ 1002.47090, found: 1002.47093.

##### *N*6-(*N*-Phenylcarbamoyl)adenosine Phosphoramidite [5′-*O*-DMT-2′-*O*-TBDMS-PhNHCO^6^A] (**3a**)

Compound **3a** was prepared according to procedure **C** using 250 mg (0.270 mmol) of 5′-*O*-DMT-2′-*O*-TBDMS-A^Ac^ phosphoramidite and 293 μL (2.70 mmol, 10 equiv) of phenyl isocyanate. After 3.5 h, the 33% solution of methylamine in ethanol (0.54 mL) was added. The product was isolated by flash chromatography (0 → 60% ethyl acetate in *n*-hexane with 0.5%_v/v_ TEA in 60 min, 40 mL/min, Biotage Sfär HC 10g column) to afford a mixture of diastereomers of **3a** (154 mg, 0.154 mmol, 57%) as a white solid. TLC (ethyl acetate): *R*_f_ = 0.77; ^1^H NMR (500 MHz, CDCl_3_, 25 °C): δ = 11.73 (s, 1H, NH_PhNHCO_), 11.72 (s, 1H, NH_PhNHCO_), 8.51 (s, 1H, H8), 8.51 (s, 1H, H8), 8.22 (s, 1H, H2), 8.20 (s, 1H, H2), 8.04 (s, 1H, NH_N6_), 8.02 (s, 1H, NH_N6_), 7.64 (m, 2H, *Ar*H-2,6_PhNHCO_), 7.49 (m, 4H, *Ar*H-2,6_Ph-DMTr_), 7.40–7.33 (m, 12H, *Ar*H-2,6_MeOPh-DMTr_, *Ar*H-3,5_PhNHCO_), 7.33–7.22 (m, 6H, *Ar*H-3,4,5_Ph-DMTr_), 7.12 (m, 2H, *Ar*H-4_PhNHCO_), 6.83 (m, 8H, *Ar*H-3,5_MeOPh-DMTr_), 6.09 (d, ^3^*J*_H,H_ = 6.3 Hz, 1H, H1′), 6.03 (d, ^3^*J*_H,H_ = 6.1 Hz, 1H, H1′), 5.05 (m, 2H, 2 × H2′), 4.45 (m, 1H, H4′), 4.43–4.38 (m, 2H, 2 × H3′), 4.36 (m, 1H, H4′), 3.96 (m, 1H, OC*H_2_*CH_2_CN), 3.88 (m, 1H, OC*H_2_*CH_2_CN), 3.79 (overlapped s, 12H, 4 × OCH_3DMTr_), 3.70–3.52 (m, 8H, OC*H_2_*CH_2_CN, 2 × H5′, 4 × CH_iPr_), 3.35 (m, 1H, H5″), 3.32 (dd, ^2^*J*_H,H_ = 10.7 Hz, ^3^*J*_H,H_ = 3.8 Hz, 1H, H5″), 2.65 (m, 2H, OCH_2_C*H_2_*CN), 2.30 (m, 2H, OCH_2_C*H_2_*CN), 1.22–1.16 (m, 18H, 6 × CH_3iPr_), 1.05 (d, ^3^*J*_H,H_ = 6.8 Hz, 6H, 2 × CH_3iPr_), 0.77 (s, 18H, 2 × *t*Bu_TBDMS_), 0.00 (s, 3H, CH_3TBDMS_), −0.03 (s, 3H, CH_3TBDMS_), −0.18 (s, 3H, CH_3TBDMS_), −0.19 (s, 3H, CH_3TBDMS_) ppm; ^31^P NMR (202.5 MHz, CDCl_3_, 25 °C): δ = 151.0 (s, 1P, P), 149.2 (s, 1P, P) ppm; HRMS (ESI) *m*/*z*: [M + H]^+^ calcd for C_53_H_68_N_8_O_8_PSi^+^ 1003.46615, found: 1003.46713;

##### *N*6-Glycinylcarbamoyladenosine Phosphoramidite [5′-*O*-DMT-2′-*O*-TBDMS-g^6^A] (**3b**)

Compound **3b** was prepared according to procedure **C** using 500 mg (0.540 mmol) of 5′-*O*-DMT-2′-*O*-TBDMS-A^Ac^ phosphoramidite and 726 μL (6.47 mmol, 12 equiv) of ethyl isocyanatoacetate. After 7.5 h, the 33% solution of methylamine in ethanol (1.0 mL) was added. The product was isolated by flash chromatography (0 → 60% ethyl acetate in *n*-hexane with 0.5%_v/v_ TEA in 35 min, 40 mL/min, Biotage Sfär HC 10g column) to afford mixture of diastereomers of **3b** (452 mg, 0.446 mmol, 83%) as a white solid. TLC (hexane/ethyl acetate 1:1): *R*_f_ = 0.27; ^1^H NMR (500 MHz, CDCl_3_, 25 °C): δ = 9.93 (m, 2H, NH_Gly_), 8.46 (s, 1H, H8), 8.45 (s, 1H, H8), 8.18 (s, 1H, H2), 8.15 (s, 1H, H2), 7.96 (s, 2H, 2 × NH-6), 7.47 (m, 4H, *Ar*H-2,6_Ph-DMTr_), 7.36 (m, 8H, *Ar*H-2,6_MeOPh-DMTr_), 7.28 (m, 4H, *Ar*H-3,5_Ph-DMTr_), 7.23 (m, 2H, *Ar*H-4_Ph-DMTr_), 6.81 (m, 8H, *Ar*H-3,5_MeOPh-DMTr_), 6.06 (d, ^3^*J*_H,H_ = 6.4 Hz, 1H, H1′), 6.01 (d, ^3^*J*_H,H_ = 6.1 Hz, 1H, H1′), 5.04 (m, 2H, 2 × H2′), 4.43 (m, 1H, H4′), 4.38 (m, 3H, 2 × H3′, H4′), 4.26 (q, ^3^*J*_H,H_ = 7.1 Hz, 4H, CH_2Et-Gly_), 4.21 (m, 4H, CH_2Gly-α_), 3.95 (m, 1H, OC*H_2_*CH_2_CN), 3.87 (m, 1H, OC*H_2_*CH_2_CN), 3.79 (overlapped s, 12H, 4 × OCH_3DMTr_), 3.70–3.52 (m, 8H, OC*H_2_*CH_2_CN, 2 × H5′, 4 × CH_iPr_), 3.33 (m, 1H, H5″), 3.31 (m, 1H, H5′′), 2.65 (m, 2H, OCH_2_C*H_2_*CN), 2.30 (m, 2H, OCH_2_C*H_2_*CN), 1.31 (t, ^3^*J*_H,H_ = 7.1 Hz, 6H, CH_3Et-Gly_), 1.21–1.15 (m, 18H, 6 × CH_3iPr_), 1.05 (d, ^3^*J*_H,H_ = 6.9 Hz, 6H, 2 × CH_3iPr_), 0.76 (s, 18H, 2 × *t*Bu_TBDMS_), −0.02 (s, 3H, CH_3TBDMS_), −0.05 (s, 3H, CH_3TBDMS_), −0.21 (s, 3H, CH_3TBDMS_), −0.22 (s, 3H, CH_3TBDMS_) ppm; ^13^C{^1^H} NMR (126 MHz, CDCl_3_, 25 °C): δ = 171.1, 170.2, 169.4, 158.7, 157.3, 154.0, 153.9, 151.3, 150.6, 150.1, 144.7, 144.6, 141.8, 141.7, 135.9, 135.8, 135.6, 135.6, 130.3, 130.3, 130.2, 128.4, 128.3, 128.1, 128.0, 127.1, 121.0, 121.0, 117.7, 117.4, 113.3, 113.3, 113.3, 88.6, 88.3, 86.9, 86.8, 84.4, 74.8, 74.8, 73.5, 73.4, 63.3, 61.6, 61.5, 57.8, 57.7, 55.4, 55.4, 43.6, 43.5, 43.1, 43.0, 42.4, 42.3, 25.8, 25.7, 24.9, 24.9, 24.8, 24.8, 20.2, 20.2, 18.1, 18.0, 14.3, 14.3, −4.6, −4.6, −5.0 ppm; ^31^P NMR (202.5 MHz, CDCl_3_, 25 °C): δ = 151.0 (s, 1P, P), 149.1 (s, 1P, P) ppm; HRMS (ESI) *m*/*z*: [M + H]^+^ calcd for C_51_H_70_N_8_O_10_PSi^+^ 1013.47163, found: 1013.47238.

##### *N*6-Glycinylcarbamoyl-*N*6-Methyladenosine Phosphoramidite [5′-*O*-DMT-2′-*O*-TBDMS-g^6^m^6^A] (**4**)

Compound **4** was prepared according to procedure **A** using 200 mg (0.197 mmol) of 5′-*O*-DMT-2′-*O*-TBDMS-g^6^A phosphoramidite (**3b**) and 123 μL (1.97 mmol, 10 equiv) of methyl iodide. The reaction was quenched after 30 min, and the product was isolated by flash chromatography (0 → 60% ethyl acetate in *n*-hexane with 0.5%_v/v_ TEA in 35 min, 40 mL/min, Biotage Sfär HC 10 g column) to afford **4** (141 mg, 0.137 mmol, 70%) as a white solid. TLC (hexane/ethyl acetate 1:1): *R*_f_ = 0.49; ^1^H NMR (500 MHz, CDCl_3_, 25 °C): δ = 11.03 (m, 2H, NH_Gly_), 8.47 (s, 1H, H8), 8.46 (s, 1H, H8), 8.19 (s, 1H, H2), 8.16 (s, 1H, H2), 7.47 (m, 4H, *Ar*H-2,6_Ph-DMTr_), 7.36 (m, 8H, *Ar*H-2,6_MeOPh-DMTr_), 7.28 (m, 4H, *Ar*H-3,5_Ph-DMTr_), 7.23 (m, 2H, *Ar*H-4_Ph-DMTr_), 6.81 (m, 8H, *Ar*H-3,5_MeOPh-DMTr_), 6.12 (d, ^3^*J*_H,H_ = 6.4 Hz, 1H, H1′), 6.06 (d, ^3^*J*_H,H_ = 6.0 Hz, 1H, H1′), 5.01 (m, 2H, 2 × H2′), 4.43 (m, 1H, H4′), 4.37 (m, 3H, 2 × H3′, H4′), 4.25 (q, ^3^*J*_H,H_ = 7.1 Hz, 4H, CH_2Et-Gly_), 4.20 (d, ^3^*J*_H,H_ = 6.4 Hz, 4H, CH_2Gly-α_), 4.01 (s, 3H, CH_3N6-Me_), 4.00 (s, 3H, CH_3N6-Me_), 3.96 (m, 1H, OC*H_2_*CH_2_CN), 3.87 (m, 1H, OC*H_2_*CH_2_CN), 3.79 (overlapped s, 12H, 4 × OCH_3DMTr_), 3.71–3.52 (m, 8H, OC*H_2_*CH_2_CN, 2 × H5′, 4 × CH_iPr_), 3.30 (m, 2H, 2 × H5″), 2.65 (m, 2H, OCH_2_C*H_2_*CN), 2.29 (m, 2H, OCH_2_C*H_2_*CN), 1.31 (t, ^3^*J*_H,H_ = 7.1 Hz, 6H, CH_3Et-Gly_), 1.20–1.15 (m, 18H, 6 × CH_3iPr_), 1.03 (d, ^3^*J*_H,H_ = 6.8 Hz, 6H, 2 × CH_3iPr_), 0.77 (s, 18H, 2 × *t*Bu_TBDMS_), −0.01 (s, 3H, CH_3TBDMS_), −0.04 (s, 3H, CH_3TBDMS_), −0.17 (s, 3H, CH_3TBDMS_), −0.19 (s, 3H, CH_3TBDMS_) ppm; ^31^P NMR (202.5 MHz, CDCl_3_, 25 °C): δ = 151.0 (s, 1P, P), 149.1 (s, 1P, P) ppm; HRMS (ESI) *m*/*z*: [M + H]^+^ calcd for C_52_H_72_N_8_O_10_PSi^+^ 1027.48728, found: 1027.48746;

##### *N*4-Methylcytidine Phosphoramidite [5′-*O*-DMT-2′-*O*-TBDMS-m^4^C^Ac^] (**5a**) and *N*3-Methylcytidine Phosphoramidite [5′-*O*-DMT-2′-*O*-TBDMS-m^3^C^Ac^] (**6a**)

Compounds **5a** and **6a** were prepared according to procedure **A** using 250 mg (0.277 mmol) of 5′-*O*-DMT-2′-*O*-TBDMS-C^Ac^ phosphoramidite and 173 μL (2.77 mmol, 10.0 equiv) of methyl iodide. The reaction was quenched after 25 min, and the products were isolated and separated by flash chromatography (0 → 100% ethyl acetate in *n*-hexane with 0.5%_v/v_ TEA in 30 min, 40 mL/min, Biotage Sfär HC 10g column) to afford mixture of diastereomers of **5a** (108 mg, 0.118 mmol, 43%) and mixture of diastereomers of **6a** (64 mg, 0.070 mmol, 25%) as white solids. m^4^C^Ac^ (**5a**): TLC (ethyl acetate): *R*_f_ = 0.48, 0.44; ^1^H NMR (500 MHz, CDCl_3_, 25 °C): δ = 8.51 (d, ^3^*J*_H,H_ = 7.6 Hz, 1H, H6), 8.42 (d, ^3^*J*_H,H_ = 7.6 Hz, 1H, H6), 7.46 (m, 2H, *Ar*H-2,6_Ph-DMTr_), 7.41 (m, 2H, *Ar*H-2,6_Ph-DMTr_), 7.35 (m, 4H, *Ar*H-2,6_MeOPh-DMTr_), 7.32–7.23 (m, 10H, *Ar*H-3,4,5_Ph-DMTr,_*Ar*H-2,6_MeOPh-DMTr_), 6.85 (m, 8H, *Ar*H-3,5_MeOPh-DMTr_), 6.53 (d, ^3^*J*_H,H_ = 7.6 Hz, 1H, H5), 6.36 (d, ^3^*J*_H,H_ = 7.6 Hz, 1H, H5), 5.88 (d, ^3^*J*_H,H_ = 1.7 Hz, 1H, H1′), 5.79 (s, 1H, H1′), 4.36 (m, 4H, 2 × H2′, 2 × H4′), 4.29 (m, 2H, 2 × H3′), 3.84 (m, 1H, OC*H_2_*CH_2_CN), 3.81 (s, 6H, 2 × OCH_3DMTr_), 3.80 (s, 6H, 2 × OCH_3DMTr_), 3.76–3.63 (m, 4H, 2 × H5′, 2 × OC*H_2_*CH_2_CN), 3.61–3.42 (m, 7H, OC*H_2_*CH_2_CN, 4 × CH_iPr_, 2 × H5″), 3.40 (s, 3H, CH_3N4-Me_), 3.38 (s, 3H, CH_3N4-Me_), 2.57 (t, ^3^*J*_H,H_ = 6.3 Hz, 2H, OCH_2_C*H_2_*CN), 2.39 (m, 2H, OCH_2_C*H_2_*CN), 2.39 (s, 3H, CH_3N4-Ac_), 2.38 (s, 3H, CH_3N4-Ac_), 1.15–1.08 (m, 18H, 6 × CH_3iPr_), 0.96 (d, ^3^*J*_H,H_ = 6.8 Hz, 6H, 2 × CH_3iPr_), 0.92 (s, 9H, *t*Bu_TBDMS_), 0.91 (s, 9H, *t*Bu_TBDMS_), 0.29 (s, 6H, 2 × CH_3TBDMS_), 0.16 (s, 6H, 2 × CH_3TBDMS_) ppm; ^31^P NMR (202.5 MHz, CDCl_3_, 25 °C): δ = 150.6 (s, 1P, P), 148.8 (s, 1P, P) ppm; HRMS (ESI) *m*/*z*: [M + H]^+^ calcd for C_48_H_66_N_5_O_9_PSi 916.44402, found: 916.44443; m^3^C^Ac^ (**6a**): TLC (ethyl acetate): *R*_f_ = 0.65; ^1^H NMR (500 MHz, CDCl_3_, 25 °C): δ = 7.81 (d, ^3^*J*_H,H_ = 8.2 Hz, 1H, H6), 7.72 (d, ^3^*J*_H,H_ = 8.2 Hz, 1H, H6), 7.41 (m, 2H, *Ar*H-2,6_Ph-DMTr_), 7.36 (m, 2H, *Ar*H-2,6_Ph-DMTr_), 7.33–7.24 (m, 16H, *Ar*H-3,4,5_Ph-DMTr,_*Ar*H-2,6_MeOPh-DMTr_), 6.84 (m, 8H, *Ar*H-3,5_MeOPh-DMTr_), 5.96 (d, ^3^*J*_H,H_ = 4.1 Hz, 1H, H1′), 5.88 (d, ^3^*J*_H,H_ = 2.7 Hz, 1H, H1′), 5.81 (d, ^3^*J*_H,H_ = 8.2 Hz, 1H, H5), 5.75 (d, ^3^*J*_H,H_ = 8.2 Hz, 1H, H5), 4.34 (m, 1H, H2′), 4.31–4.25 (m, 4H, H2′, 2 × H3′, H4′), 4.22 (m, 1H, H4′), 3.92 (m, 1H, OC*H_2_*CH_2_CN), 3.81 (s, 6H, 2 × OCH_3DMTr_), 3.81 (s, 6H, 2 × OCH_3DMTr_), 3.78 (m, 1H, OC*H_2_*CH_2_CN), 3.69 (m, 1H, OC*H_2_*CH_2_CN), 3.64–3.51 (m, 7H, 2 × H5′, OC*H_2_*CH_2_CN, 4 × CH_iPr_), 3.42–3.36 (m, 2H, 2 × H5″), 3.36 (s, 3H, CH_3N3-Me_), 3.35 (s, 3H, CH_3N3-Me_), 2.64 (m, 2H, OCH_2_C*H_2_*CN), 2.39 (m, 2H, OCH_2_C*H_2_*CN), 2.19 (s, 3H, CH_3N4-Ac_), 2.18 (s, 3H, CH_3N4-Ac_), 1.15 (d, ^3^*J*_H,H_ = 6.7 Hz, 18H, 6 × CH_3iPr_), 0.99 (d, ^3^*J*_H,H_ = 6.8 Hz, 6H, 2 × CH_3iPr_), 0.90 (s, 9H, *t*Bu_TBDMS_), 0.89 (s, 9H, *t*Bu_TBDMS_), 0.16 (s, 6H, CH_3TBDMS_), 0.15 (s, 6H, CH_3TBDMS_), 0.14 (s, 3H, CH_3TBDMS_), 0.12 (s, 3H, CH_3TBDMS_) ppm; ^31^P NMR (202.5 MHz, CDCl_3_, 25 °C): δ = 150.0 (s, 1P, P), 149.7 (s, 1P, P) ppm; HRMS (ESI) *m*/*z*: [M + H]^+^ calcd for C_48_H_67_N_5_O_9_PSi^+^ 916.44402, found: 916.44456.

##### *N*3-Methylcytidine Phosphoramidite (5′-*O*-DMT-2′-*O*-TBDMS-m^3^C^Bz^) (**6b**)

Compound **6b** was prepared according to procedure **A** using 500 mg (0.519 mmol) of 5′-*O*-DMT-2′-*O*-TBDMS-C^Bz^ phosphoramidite and 65 μL (1.04 mmol, 2.0 equiv) of methyl iodide. The reaction was quenched after 25 min, and the product was isolated and separated by flash chromatography (0 → 100% ethyl acetate in *n*-hexane with 0.5%_v/v_ TEA in 30 min, 40 mL/min, Biotage Sfär HC 10 g column) to afford mixture of diastereomers of **6b** (380 mg, 0.389 mmol, 75%) as white solids. TLC (ethyl acetate): *R*_f_ = 0.73; ^1^H NMR (500 MHz, CDCl_3_, 25 °C): δ = 8.13 (m, 4H, 2 × *Ar*H-2,6_Ph-Bz_), 7.88 (d, ^3^*J*_H,H_ = 8.2 Hz, 1H, H6), 7.78 (d, ^3^*J*_H,H_ = 8.2 Hz, 1H, H6), 7.52 (m, 2H, 2 × *A*rH-4_Ph-Bz_), 7.43 (m, 4H, 2 × *Ar*H-3,5_Ph-Bz_), 7.39 (m, 2H, *Ar*H-2,6_Ph-DMTr_), 7.35 (m, 2H, *Ar*H-2,6_Ph-DMTr_), 7.31–7.23 (m, 14H, *Ar*H-3,5_Ph-DMTr,_*Ar*H-2,6_MeOPh-DMTr_), 7.20 (m, 2H, *Ar*H-4_Ph-DMTr_), 6.82 (m, 8H, *Ar*H-3,5_MeOPh-DMTr_), 6.10 (d, ^3^*J*_H,H_ = 8.2 Hz, 1H, H5), 6.03 (d, ^3^*J*_H,H_ = 8.2 Hz, 1H, H5), 6.00 (d, ^3^*J*_H,H_ = 4.1 Hz, 1H, H1′), 5.91 (d, ^3^*J*_H,H_ = 3.0 Hz, 1H, H1′), 4.37 (m, 1H, H2′), 4.34–4.25 (m, 4H, H2′, 2 × H3′, H4′), 4.24 (m, 1H, H4′), 3.93 (m, 1H, OC*H_2_*CH_2_CN), 3.80–3.77 (overlapped, 13H, OC*H_2_*CH_2_CN, 4 × OCH_3DMTr_), 3.70 (m, 1H, OC*H_2_*CH_2_CN), 3.62 (dd,^2^*J*_H,H_ = 11.1 Hz, ^3^*J*_H,H_ = 1.2 Hz, 1H, H5′), 3.60–3.51 (m, 12H, OC*H_2_*CH_2_CN, 4 × CH_iPr_, 2 × CH_3N3-Me_, H5′), 3.40 (dd,^2^*J*_H,H_ = 11.1 Hz, ^3^*J*_H,H_ = 2.1 Hz, 1H, H5″), 3.37 (dd,^2^*J*_H,H_ = 11.1 Hz, ^3^*J*_H,H_ = 2.7 Hz, 1H, H5″), 2.64 (m, 2H, OCH_2_C*H_2_*CN), 2.38 (m, 2H, OCH_2_C*H_2_*CN), 1.17–1.13 (m, 18H, 6 × CH_3iPr_), 0.99 (d, ^3^*J*_H,H_ = 6.8 Hz, 6H, 2 × CH_3iPr_), 0.91 (s, 9H, *t*Bu_TBDMS_), 0.90 (s, 9H, *t*Bu_TBDMS_), 0.17 (s, 6H, 2 × CH_3TBDMS_), 0.15 (s, 3H, CH_3TBDMS_), 0.13 (s, 3H, CH_3TBDMS_) ppm; ^31^P NMR (202.5 MHz, CDCl_3_, 25 °C): δ = 150.0 (s, 1P, P), 149.7 (s, 1P, P) ppm; HRMS (ESI) *m*/*z*: [M + H]^+^ calcd for C_53_H_69_N_5_O_9_PSi^+^ 978.45967, found: 978.46048;

##### *N*3-(2-Nitrobenzyl)cytidine Phosphoramidite [5′-*O*-DMT-2′-*O*-TBDMS-(2-NO_2_-Bn)^3^C^Bz^] (**6c**)

Compound **6c** was prepared according to procedure **B** using 250 mg (0.259 mmol) of 5′-*O*-DMT-2′-*O*-TBDMS-C^Bz^ phosphoramidite and 445 mg (2.59 mmol, 10 equiv) of 2-nitrobenzyl chloride. The reaction was quenched after 30 min, and the product was isolated by flash chromatography (0 → 50% ethyl acetate in *n*-hexane with 0.5%_v/v_ TEA in 30 min, 40 mL/min, Biotage Sfär HC 10 g column) to afford a mixture of diastereomers of **6c** (207 mg, 0.188 mmol, 73%) as a pale yellow solid. TLC (ethyl acetate): *R*_f_ = 0.70; ^1^H NMR (500 MHz, CDCl_3_, 25 °C): δ = 8.09 (m, 1H, *Ar*H), 8.08 (m, 1H, *Ar*H), 7.94 (d, ^3^*J*_H,H_ = 8.3 Hz, 1H, H6_C_), 7.85 (d, ^3^*J*_H,H_ = 8.2 Hz, 1H, H6_C_), 7.79 (m, 4H, *Ar*H), 7.56 (m, 2H, *Ar*H), 7.48–7.19 (m, 28H, *Ar*H), 6.83 (m, 8H, 2 × *Ar*H-3,5_MeOPh-DMTr_), 6.16 (d, ^3^*J*_H,H_ = 8.3 Hz, 1H, H5_C_), 6.09 (d, ^3^*J*_H,H_ = 8.2 Hz, 1H, H5_C_), 6.01 (d, ^3^*J*_H,H_ = 4.2 Hz, 1H, H1′), 5.94 (d, ^3^*J*_H,H_ = 3.2 Hz, 1H, H1′), 5.76 (m, 2H, CH_2nBn_), 5.75 (m, 2H, CH_2nBn_), 4.43 (m, 1H, H2′), 4.38 (m, 1H, H2′), 4.37–4.33 (m, 2H, 2 × H3′), 4.32 (m, 1H, H4′), 4.24 (m, 1H, H4′), 3.93 (m, 1H, OC*H_2_*CH_2_CN), 3.79 (m, 1H, OC*H_2_*CH_2_CN), 3.79 (s, 6H, 2 × OCH_3DMTr_), 3.78 (s, 6H, 2 × OCH_3DMTr_), 3.73 (m, 1H, OC*H_2_*CH_2_CN), 3.65–3.51 (m, 8H, OC*H_2_*CH_2_CN, 2 × H5′, 4 × CH_iPr_), 3.42 (m, 1H, H5″), 3.40 (m, 1H, H5′′), 2.64 (m, 2H, OCH_2_C*H_2_*CN), 2.40 (m, 2H, OCH_2_C*H_2_*CN), 1.17–1.13 (m, 18H, 6 × CH_3iPr_), 1.01 (d, ^3^*J*_H,H_ = 6.8 Hz, 6H, 2 × CH_3iPr_), 0.90 (s, 9H, *t*Bu_TBDMS_), 0.88 (s, 9H, *t*Bu_TBDMS_), 0.14 (s, 3H, CH_3TBDMS_), 0.14 (s, 3H, CH_3TBDMS_), 0.13 (s, 3H, CH_3TBDMS_), 0.12 (s, 3H, CH_3TBDMS_) ppm; ^31^P NMR (202.5 MHz, CDCl_3_, 25 °C): δ = 150.8 (s, 1P, P), 150.8 (s, 1P, P) ppm; HRMS (ESI) *m*/*z*: [M + H]^+^ calcd for C_59_H_72_N_6_O_11_PSi^+^ 1099.47605, found: 1099.47702.

##### *N*6-(*N*-Phenylcarbamoyl)cytidine Phosphoramidite [5′-*O*-DMT-2′-*O*-TBDMS-PhNHCO^4^C] (**7**)

Compound **7** was prepared according to procedure **C** using 260 mg (0.288 mmol) of 5′-*O*-DMT-2′-*O*-TBDMS-C^Ac^ phosphoramidite and 250 μL (2.30 mmol, 8 equiv) of phenyl isocyanate. After 35 min, the 33% solution of methylamine in ethanol (0.52 mL) was added. The product was isolated by flash chromatography (0 → 75% ethyl acetate in *n*-hexane with 0.5%_v/v_ TEA in 40 min, 40 mL/min, Biotage Sfär HC 10g column) to afford a mixture of diastereomers of **7** (119 mg, 0.122 mmol, 42%) as a white solid. TLC (ethyl acetate): *R*_f_ = 0.68, 0.59; ^1^H NMR (500 MHz, CDCl_3_, 25 °C): δ = 11.49 (m, 2H, NH_N4/PhNHCO_), 10.79 (m, 2H, NH_N4/PhNHCO_), 8.53 (m, 1H, H6), 8.39 (m, 1H, H6), 7.64 (m, 4H, *Ar*H), 7.45 (m, 2H, *Ar*H), 7.40 (m, 2H *Ar*H), 7.37–7.24 (m, 19H, *Ar*H, H5), 7.21 (m, 1H, H5), 7.05 (m, 2H, *Ar*H), 6.86 (m, 8H, *Ar*H-3,5_MeOPh-DMTr_), 6.17 (s, 1H, H1′), 6.08 (s, 1H, H1′), 4.40 (m, 1H, H2′), 4.38–4.30 (m, 5H, H2′, 2 × H3′, 2 × H4′), 3.90 (m, 1H, OC*H_2_*CH_2_CN), 3.80 (overlapped s, 6H, 2 × OCH_3DMTr_), 3.79 (s, 6H, 2 × OCH_3DMTr_), 3.78 (m, 1H, OC*H_2_*CH_2_CN) 3.74–3.64 (m, 3H, 2 × H5′, OC*H_2_*CH_2_CN), 3.63–3.52 (m, 5H, OC*H_2_*CH_2_CN, 4 × CH_iPr_), 3.46 (m, 2H, 2 × H5″), 2.60 (m, 2H, OCH_2_C*H_2_*CN), 2.41 (t, ^3^*J*_H,H_ = 6.5 Hz, 2H, OCH_2_C*H_2_*CN), 1.18–1.13 (m, 18H, 6 × CH_3iPr_), 1.00 (d, ^3^*J*_H,H_ = 6.7 Hz, 6H, 2 × CH_3iPr_), 0.90 (s, 9H, *t*Bu_TBDMS_), 0.89 (s, 9H, *t*Bu_TBDMS_), 0.17 (s, 6H, 2 × CH_3TBDMS_), 0.13 (s, 3H, CH_3TBDMS_), 0.10 (s, 3H, CH_3TBDMS_) ppm; ^13^C{^1^H} NMR (126 MHz, CDCl_3_, 25 °C): δ = 158.9, 158.9, 139.4, 138.5, 135.4, 135.2, 130.4, 130.3, 129.3, 128.7, 128.6, 128.2, 128.1, 127.4, 127.4, 123.9, 120.8, 120.0, 117.7, 117.5, 114.2, 113.5, 113.4, 87.5, 87.4, 55.4, 55.4, 43.5, 43.4, 43.3, 43.2, 34.0, 32.1, 29.8, 29.8, 29.8, 29.7, 29.5, 29.3, 29.1, 25.9, 25.9, 25.0, 25.0, 24.9, 24.8, 24.8, 24.7, 24.7, 22.8, 20.6, 20.6, 20.4, 20.3, 18.2, 14.3, −4.2, −4.2, −4.7, −4.8 ppm; ^31^P NMR (202.5 MHz, CDCl_3_, 25 °C): δ = 150.3 (s, 1P, P), 149.9 (s, 1P, P) ppm; HRMS (ESI) *m*/*z*: [M + H]^+^ calcd for C_52_H_68_N_6_O_9_PSi^+^ 979.45492, found: 979.45708;

##### *N*3-Methyluridine Phosphoramidite [5′-*O*-DMT-2′-*O*-Me-m^3^U_m_] (**8a**)

Compound **8a** was prepared according to procedure **A** using 250 mg (0.329 mmol) of 5′-*O*-DMT-2′-*O*-Me-U phosphoramidite and 205 μL (3.29 mmol, 10 equiv) of methyl iodide. The reaction was quenched after 25 min, and the product was isolated by flash chromatography (0 → 100% ethyl acetate in *n*-hexane with 0.5%_v/v_ TEA in 30 min, 40 mL/min, Biotage Sfär HC 10 g column) to afford a mixture of diastereomers of **8a** (227 mg, 0.293 mmol, 89%) as a white solid. TLC (ethyl acetate): *R*_f_ = 0.60; ^1^H NMR (500 MHz, CDCl_3_, 25 °C): δ = 8.05 (d, ^3^*J*_H,H_ = 8.1 Hz, 1H, H6_U_), 7.95 (d, ^3^*J*_H,H_ = 8.1 Hz, 1H, H6_U_), 7.41 (m, 2H, *Ar*H_Ph-DMTr_), 7.36 (m, 2H, *Ar*H_Ph-DMTr_), 7.33–7.23 (m, 14H, *Ar*H), 6.84 (m, 8H, 2 × *Ar*H-3,5_MeOPh-DMTr_), 6.04 (d, ^3^*J*_H,H_ = 2.7 Hz, 1H, H1′), 5.99 (d, ^3^*J*_H,H_ = 1.8 Hz, 1H, H1′), 5.32 (d, ^3^*J*_H,H_ = 8.1 Hz, 1H, H5_U_), 5.29 (d, ^3^*J*_H,H_ = 8.1 Hz, 1H, H5_U_), 4.61 (m, 1H, H3′), 4.46 (m, 1H, H3′), 4.24 (m, 1H, H4′), 4.21 (m, 1H, H4′), 3.92 (m, 1H, H2′), 3.88 (m, 2H, H2′, OC*H_2_*CH_2_CN), 3.83 (m, 1H, OC*H_2_*CH_2_CN), 3.80 (s, 3H, OCH_3DMTr_), 3.80 (s, 3H, OCH_3DMTr_), 3.79 (s, 3H, OCH_3DMTr_), 3.79 (s, 3H, OCH_3DMTr_), 3.68–3.41 (m, 10H, OC*H_2_*CH_2_CN, 2 × H5′, 2 × H5′, 4 × CH_iPr_), 3.60 (s, 3H, CH_3N3-Me_), 3.60 (s, 3H, CH_3N3-Me_), 3.32 (s, 6H, 2 × CH_32′-O_), 2.64 (m, 2H, OCH_2_C*H_2_*CN), 2.40 (t, ^3^*J*_H,H_ = 6.2 Hz, 2H, OCH_2_C*H_2_*CN), 1.19 (d, ^3^*J*_H,H_ = 6.7 Hz, 6H, CH_3iPr_), 1.19 (d, ^3^*J*_H,H_ = 6.7 Hz, 6H, CH_3iPr_), 1.16 (d, ^3^*J*_H,H_ = 6.8 Hz, 6H, CH_3iPr_), 1.03 (d, ^3^*J*_H,H_ = 6.8 Hz, 6H, CH_3iPr_) ppm; ^13^C{^1^H} NMR (126 MHz, CDCl_3_, 25 °C): δ = 163.1, 163.1, 158.9, 158.9, 158.9, 151.2, 151.2, 144.4, 144.3, 137.8, 137.8, 135.4, 135.3, 135.2, 135.1, 130.4, 130.4, 128.5, 128.4, 128.1, 127.4, 127.4, 117.8, 117.6, 113.4, 113.3, 101.7, 101.6, 88.4, 88.4, 87.3, 87.1, 83.9, 83.8, 83.2, 83.2, 82.4, 82.3, 82.1, 82.1, 69.9, 69.8, 69.7, 69.7, 61.5, 60.8, 58.8, 58.8, 58.8, 58.7, 58.4, 58.4, 58.3, 58.1, 55.4, 55.4, 43.5, 43.4, 43.4, 43.3, 29.8, 27.7, 27.6, 24.8, 24.8, 24.8, 24.7, 24.7, 24.6, 20.5, 20.5, 20.4, 20.4 ppm; ^31^P NMR (202.5 MHz, CDCl_3_, 25 °C): δ = 150.7 (s, 1P, P), 150.2 (s, 1P, P) ppm; HRMS (ESI) *m*/*z*: [M + H]^+^ calcd for C_41_H_52_N_4_O_9_P^+^ 775.34664, found: 775.34746.

##### *N*3-(2-Nitrobenzyl)thymidine Phosphoramidite [5′-*O*-DMT-(2-NO_2_-Bn)^3^T] (**8b**)

Compound **8b** was prepared according to procedure **B** using 250 mg (0.336 mmol) of 5′-*O*-DMT-T phosphoramidite and 577 mg (3.36 mmol, 10 equiv) of 2-nitrobenzyl chloride. The reaction was quenched after 35 min, and the product was isolated by flash chromatography (0 → 100% ethyl acetate in *n*-hexane with 0.5%_v/v_ TEA in 35 min, 40 mL/min, Biotage Sfär HC 10 g column) to afford a mixture of diastereomers of **8c** (211 mg, 0.240 mmol, 71%) as a pale yellow solid. TLC (ethyl acetate): *R*_f_ = 0.67; ^1^H NMR (500 MHz, CDCl_3_, 25 °C): δ = 8.04 (d, ^3^*J*_H,H_ = 1.1 Hz, 1H, *Ar*H-3_nBn_), 8.02 (d, ^3^*J*_H,H_ = 1.2 Hz, 1H, *Ar*H-3_nBn_), 7.73 (d, ^3^*J*_H,H_ = 1.1 Hz, 1H, H6_T_), 7.68 (d, ^3^*J*_H,H_ = 1.1 Hz, 1H, H6_T_), 7.52 (m, 2H, 2 × *Ar*H-5_nBn_), 7.43–7.37 (m, 6H, 2 × *Ar*H-4_nBn_, 2 × *Ar*H-2,6_Ph-DMTr_), 7.33–7.27 (m, 12H, 2 × *Ar*H-2,6_MeOPh-DMTr_, 2 × *Ar*H-3,5_Ph-DMTr_), 7.27–7.20 (m, 4H, 2 × ArH-6_nBn_, 2 × *Ar*H-4_Ph-DMTr_), 6.84 (m, 8H, 2 × *Ar*H-3,5_MeOPh-DMTr_), 6.41 (m, 2H, 2 × H1′), 5.52 (m, 4H, 2 × CH_2nBn_), 4.66 (m, 2H, 2 × H3′), 4.18 (m, 1H, H4′), 4.14 (m, 1H, H4′), 3.80 (s, 6H, 2 × OCH_3DMTr_), 3.79 (s, 6H, 2 × OCH_3DMTr_), 3.77 (m, 2H, OC*H_2_*CH_2_CN), 3.68–3.45 (m, 8H, OC*H_2_*CH_2_CN, 2 × H5′, 4 × CH_iPr_), 3.34 (m, 2H, 2 × H5″), 2.61 (t, ^3^*J*_H,H_ = 6.2 Hz, 2H, OCH_2_C*H_2_*CN), 2.56 (ddd, ^2^*J*_H,H_ = 13.4 Hz, ^3^*J*_H,H_ = 5.8 Hz, ^3^*J*_H,H_ = 2.4 Hz, 1H, H2′), 2.49 (ddd, ^2^*J*_H,H_ = 13.4 Hz, ^3^*J*_H,H_ = 5.8 Hz, ^3^*J*_H,H_ = 2.9 Hz, 1H, H2′), 2.41 (t, ^3^*J*_H,H_ = 6.4 Hz, 2H, OCH_2_C*H_2_*CN), 2.35 (m, 2H, 2 × H2″), 1.48 (s, 6H, 2 × CH_3T_), 1.18–1.13 (m, 18H, 6 × CH_3iPr_), 1.05 (d, ^3^*J*_H,H_ = 6.8 Hz, 6H, 2 × CH_3iPr_) ppm; ^13^C{^1^H} NMR (126 MHz, CDCl_3_, 25 °C): δ = 163.5, 158.9, 158.9, 151.0, 151.0, 148.9, 144.4, 144.4, 135.5, 135.5, 135.4, 135.4, 134.5, 134.4, 133.6, 133.6, 132.5, 130.3, 130.3, 130.3, 128.4, 128.3, 128.1, 128.1, 128.1, 127.3, 127.3, 125.1, 125.1, 117.7, 117.5, 113.4, 113.4, 110.6, 110.6, 87.1, 87.1, 85.9, 85.9, 85.7, 85.6, 74.1, 74.0, 73.6, 73.5, 63.4, 63.2, 58.4, 58.3, 58.1, 55.4, 55.4, 43.5, 43.4, 43.4, 43.3, 41.8, 40.3, 40.3, 29.8, 24.8, 24.7, 24.7, 24.6, 24.6, 20.6, 20.5, 20.4, 20.3, 12.6, 12.6 ppm; ^31^P NMR (202.5 MHz, CDCl_3_, 25 °C): δ = 148.9 (s, 1P, P), 148.5 (s, 1P, P) ppm; HRMS (ESI) *m*/*z*: [M + H]^+^ calcd for C_47_H_55_N_5_O_10_P^+^ 880.36811, found: 880.36806.

##### *N*1-(4-*O*-Acetyl)benzyl-*N*2-methylguanosine Phosphoramidite [5′-*O*-DMT-2′-*O*-TBDMS-(4-OAc)Bn^1^m^2^G^iBu^] (**9**)

Compound **9** was prepared according to procedure **A** using 1.00 g (1.03 mmol) of 5′-*O*-DMT-2′-*O*-TBDMS-G^iBu^ phosphoramidite and 569 mg (2.06 mmol, 2.0 equiv) of (iodomethyl)phenyl acetate. After 2.5 h, the aqueous fraction was removed and 320 μL (5.15 mmol, 5 equiv) of methyl iodide and a fresh aqueous solution of NaOH with Bu_4_NBr (1.0 equiv) was added to the organic phase. The reaction was quenched after 1 h, and the product was isolated by flash chromatography (0 → 50% ethyl acetate in *n*-hexane with 0.5%_v/v_ TEA in 60 min, 40 mL/min, Biotage Sfär HC 10g column) and additionally purified by the second column chromatography (0 → 100% DCM in *n*-hexane with 0.5%_v/v_ TEA in 30 min, 40 mL/min, Biotage Sfär HC 10 g column) to afford a mixture of diastereomers of **9** (140 mg, 0.124 mmol, 12%) as a white solid. TLC (ethyl acetate): *R*_f_ = 0.68; ^1^H NMR (500 MHz, CDCl_3_, 25 °C): δ = 8.21 (s, 1H, H8), 8.17 (s, 1H, H8), 7.52 (m, 4H, ArH_N1_AcOBn_), 7.44 (m, 4H, *Ar*H-2,6_Ph-DMTr_), 7.34 (m, 8H, *Ar*H-2,6_MeOPh-DMTr_), 7.32–7.20 (m, 6H, *Ar*H-3,4,5_Ph-DMTr_), 7.09 (m, 4H, *Ar*H_N1_AcOBn_), 6.83 (m, 8H, *Ar*H-3,5_MeOPh-DMTr_), 6.11 (d, ^3^*J*_H,H_ = 6.7 Hz, 1H, H1′), 6.09 (d, ^3^*J*_H,H_ = 6.9 Hz, 1H, H1′), 5.61 (m, 4H, CH_2N1_AcOBn_), 4.82 (dd, ^3^*J*_H,H_ = 6.6 Hz, ^3^*J*_H,H_ = 4.6 Hz, 1H, H2′), 4.74 (dd, ^3^*J*_H,H_ = 6.8 Hz, ^3^*J*_H,H_ = 5.0 Hz, 1H, H2′), 4.40 (m, 1H, H4′), 4.38 (m, 1H, H3′), 4.32 (m, 2H, H3′, H4′), 3.92 (m, 1H, OC*H_2_*CH_2_CN), 3.86 (m, 1H, OC*H_2_*CH_2_CN), 3.79–3.77 (overlapped s, 12H, 4 × OCH_3DMTr_), 3.69–3.50 (m, 7H, OC*H_2_*CH_2_CN, H5′, 4 × CH_iPr_), 3.45 (m, 1H, H5′), 3.40–3.36 (overlapped, 4H, CH_3N2-Me_, H5″), 3.35–3.28 (m, 3H, H5′′, 2 × CH_iBu_), 3.26 (dd, ^2^*J*_H,H_ = 10.6 Hz, ^3^*J*_H,H_ = 4.0 Hz, 1H, H5″), 3.12 (s, 3H, CH_3G-dmf_), 3.11 (s, 6H, 2 × CH_3G-dmf_), 2.61 (m, 2H, OCH_2_C*H_2_*CN), 2.29 (s, 6H, 2 × CH_3AcOBn_) 2.28 (m, 2H, OCH_2_C*H_2_*CN), 1.21–1.12 (m, 30H, 6 × CH_3iPr_, 4 × CH_3iBu_), 1.05 (d, ^3^*J*_H,H_ = 6.8 Hz, 6H, 2 × CH_3iPr_), 0.75 (s, 9H, *t*Bu_TBDMS_), 0.72 (s, 9H, *t*Bu_TBDMS_), 0.01 (s, 3H, CH_3TBDMS_), −0.01 (s, 3H, CH_3TBDMS_), −0.18 (s, 3H, CH_3TBDMS_), −0.21 (s, 3H, CH_3TBDMS_) ppm; ^13^C{^1^H} NMR (126 MHz, CDCl_3_, 25 °C): δ = 169.5, 160.3, 158.8, 130.3, 130.2, 130.1, 129.4, 129.4, 129.2, 128.9, 128.8, 128.3, 128.2, 128.2, 128.2, 128.1, 127.3, 127.2, 121.9, 121.4, 113.5, 113.5, 113.4, 113.4, 68.2, 68.2, 55.4, 55.4, 43.6, 43.5, 25.7, 25.6, 24.9, 24.8, 24.8, 24.8, 21.3, 21.3, 20.3, 20.3, 20.3, 20.2, 18.1, 18.0, −4.5, −4.5, −5.0, −5.1 ppm; ^31^P NMR (202.5 MHz, CDCl_3_, 25 °C): δ = 151.6 (s, 1P, P), 148.8 (s, 1P, P) ppm; HRMS (ESI) *m*/*z*: [M + H]^+^ calcd for C_60_H_79_N_7_O_11_PSi^+^ 1132.53390, found: 1132.53482.

##### *N*1-Methylguanosine Phosphoramidite [5′-*O*-DMT-2′-*O*-TBDMS-m^1^G^dmf^] (**10**)

Compound **10** was prepared according to procedure **A** using 250 mg (0.262 mmol) of 5′-*O*-DMT-2′-*O*-TBDMS-G^dmf^ phosphoramidite and 163 μL (2.62 mmol, 10 equiv) of methyl iodide. The reaction was quenched after 25 min, and the product was isolated by flash chromatography (0 → 100% ethyl acetate in *n*-hexane with 0.5%_v/v_ TEA in 30 min, 40 mL/min, Biotage Sfär HC 10 g column) to afford a mixture of diastereomers of **10** (208 mg, 0.215 mmol, 82%) as a white solid. TLC (ethyl acetate): *R*_f_ = 0.34; ^1^H NMR (500 MHz, CDCl_3_, 25 °C): δ = 8.52 (s, 1H, N=C*H-*N_G-dmf_), 8.47 (s, 1H, N=C*H-*N_G-dmf_), 7.86 (s, 1H, H8), 7.82 (s, 1H, H8), 7.44 (m, 4H, *Ar*H-2,6_Ph-DMTr_), 7.33 (m, 8H, *Ar*H-2,6_MeOPh-DMTr_), 7.28 (m, 4H, *Ar*H-3,5_Ph-DMTr_), 7.22 (m, 2H, *Ar*H-4_Ph-DMTr_), 6.81 (m, 8H, *Ar*H-3,5_MeOPh-DMTr_), 6.02 (d, ^3^*J*_H,H_ = 6.3 Hz, 1H, H1′), 5.96 (d, ^3^*J*_H,H_ = 6.0 Hz, 1H, H1′), 4.71 (dd, ^3^*J*_H,H_ = 6.3 Hz, ^3^*J*_H,H_ = 4.7 Hz, 1H, H2′), 4.67 (dd, ^3^*J*_H,H_ = 6.0 Hz, ^3^*J*_H,H_ = 4.9 Hz, 1H, H2′), 4.37 (m, 1H, H4′), 4.34 (m, 1H, H3′), 4.28 (m, 2H, H3′, H4′), 3.90 (m, 2H, OC*H_2_*CH_2_CN), 3.79–3.77 (overlapped s, 12H, 4 × OCH_3DMTr_), 3.67 (s, 6H, 2 × CH_3N1-Me_), 3.66–3.50 (m, 7H, OC*H_2_*CH_2_CN, H5′, 4 × CH_iPr_), 3.42 (dd, ^2^*J*_H,H_ = 10.6 Hz, ^3^*J*_H,H_ = 2.6 Hz, 1H, H5′), 3.31 (dd, ^2^*J*_H,H_ = 10.6 Hz, ^3^*J*_H,H_ = 3.8 Hz, 1H, H5″), 3.26 (dd, ^2^*J*_H,H_ = 10.6 Hz, ^3^*J*_H,H_ = 4.0 Hz, 1H, H5″), 3.12 (s, 3H, CH_3G-dmf_), 3.11 (s, 6H, 2 × CH_3G-dmf_), 3.02 (s, 3H, CH_3G-dmf_), 2.65 (m, 2H, OCH_2_C*H_2_*CN), 2.29 (m, 2H, OCH_2_C*H_2_*CN), 1.19–1.14 (m, 18H, 6 × CH_3iPr_), 1.01 (d, ^3^*J*_H,H_ = 6.8 Hz, 6H, 2 × CH_3iPr_), 0.81 (s, 9H, *t*Bu_TBDMS_), 0.79 (s, 9H, *t*Bu_TBDMS_), 0.01 (s, 3H, CH_3TBDMS_), 0.00 (s, 3H, CH_3TBDMS_), −0.12 (s, 3H, CH_3TBDMS_), −0.14 (s, 3H, CH_3TBDMS_) ppm; ^13^C{^1^H} NMR (126 MHz, CDCl_3_, 25 °C): δ = 158.7, 158.7, 157.5, 157.2, 157.1, 148.5, 148.3, 144.6, 144.5, 136.0, 135.8, 135.8, 135.6, 135.5, 130.3, 130.2, 130.1, 128.3, 128.2, 128.1, 128.1, 127.1, 120.2, 120.0, 117.6, 117.4, 113.4, 113.4, 113.4, 86.9, 86.7, 86.7, 86.4, 83.5, 83.5, 83.4, 76.6, 75.7, 75.7, 73.5, 73.4, 63.8, 63.5, 59.0, 58.9, 57.9, 57.7, 55.4, 55.4, 43.6, 43.5, 43.1, 43.0, 41.2, 41.0, 35.3, 35.2, 34.0, 30.1, 30.0, 29.8, 29.3, 29.1, 25.8, 25.8, 24.9, 24.8, 24.8, 24.7, 20.5, 20.4, 20.2, 20.2, 18.1, 18.1, 14.3, −4.5, −4.6, −4.8, −4.9 ppm; ^31^P NMR (202.5 MHz, CDCl_3_, 25 °C): δ = 150.7 (s, 1P, P), 149.6 (s, 1P, P) ppm; HRMS (ESI) *m*/*z*: [M + H]^+^ calcd for C_50_H_70_N_8_O_8_PSi^+^ 969.48180, found: 969.48308.

### Solid-Phase Synthesis of Oligonucleotides

#### General Procedure

Solid-phase syntheses of short oligonucleotides were performed in a 10 mL syringe equipped with frit and loaded with polystyrene support [ribo U 300 PrimerSupport 5G (298 μmol/g, GE Healthcare), ribo G 300 PrimerSupport 5G (308 μmol/g, GE Healthcare), or dC 350 PrimerSupport 5G (360 μmol/g, GE Healthcare)]. The typical synthesis scale was 15 μmol (based on the support loading provided by the manufacturer), but it could be easily scaled-up to ca. 200 μmol using this setup. The detritylation step was performed by passing 5 mL of 3% (v/v) trichloroacetic acid in DCM through the column. The solid support was washed with 5 mL of DNA synthesis grade acetonitrile (<10 ppm of H_2_O) and dried in a vacuum desiccator. In the coupling step, a 0.3 M solution of an appropriate phosphoramidite (3.0 equivalents) in anhydrous acetonitrile and a 1.5 volume of 0.3 M BTT Activator were shaken with the support for 30 min. Then the support was washed with 5 mL of acetonitrile and the phosphite triester was oxidized by passing 1.5 mL of 0.05 M iodine in pyridine/water 9:1_v/v_. To prepare the dinucleotide 5′-phosphates, the bis(2-cyanoethyl)-*N*,*N*-diisopropylphosphoramidite (3.0 equivalents, 0.3 M in acetonitrile + 1.5 volume of 0.3 M BTT Activator) was used in the last cycle and the detritylation step was omitted. After the last cycle of the synthesis, 2-cyanoethyl groups were removed by passing 5 mL of 20%_v/v_ solution of diethylamine in acetonitrile. The support was dried in a vacuum desiccator and transferred to a 50 mL polypropylene tube, and the oligonucleotide was cleaved from the support using AMA (1 mL, 1:1_v/v_ mixture of 33% ammonium hydroxide and 40% methylamine in water for 3 h at 37 °C (Eppendorf ThermoMixer C, 1000 rpm)***. The suspension was filtered, washed with water, evaporated to dryness, redissolved in water, and freeze-dried. The residue was dissolved in 20 μL of DMSO, followed by the addition of triethylamine (33 μL) and triethylammonium trihydrofluoride (TEA·3HF, 20 μL), and the resulting mixture was shaken for 3 h at 65 °C (Eppendorf ThermoMixer C, 1000 rpm). The reaction was quenched by addition of 0.05 M NaHCO_3_ in water (ca. 20 mL), and the pH was adjusted to 6–7 if necessary. A sample of the product for compound characterization was isolated by ion-exchange chromatography on DEAE Sephadex using a linear gradient of TEAB: 0–0.9 M for the dinucleotides and 0–1.2 M for the trinucleotides and evaporated to dryness with ethanol to give a white solid.

*** Oligonucleotide **14** (prepared using phosphoramidite **1e**) was treated with AMA for 4 h at 37 °C (Eppendorf ThermoMixer C, 1000 rpm) to ensure complete aminolysis of phthalimide moiety. Oligonucleotide **24** prepared using phosphoramidite **9** (m^2^G) was deprotected with AMA for 3 h at 37 °C (Eppendorf ThermoMixer C, 1000 rpm) and then left at 4 °C overnight for complete elimination of 4-hydroxybenzyl substituent. Oligonucleotide **19** containing m^3^C was cleaved from the solid support and deprotected using 30–33% aqueous ammonium hydroxide to avoid *N*4-transamination with methylamine deprotection with AMA produced dinucleotide **21** (*N*3,*N*4-dimethylcytidine derivative p(m_2_^3,4^C)pG) as the only product.

##### U^i6^AU (**11**)

^1^H NMR (500 MHz, D_2_O, 25 °C): δ = 8.38 (s, 1H, H8_A2_), 8.22 (s, 1H, H2_A2_), 7.77 (d, ^3^*J*_H,H_ = 8.2 Hz, 1H, H6_U_), 7.76 (d, ^3^*J*_H,H_ = 8.1 Hz, 1H, H6_U_), 6.10 (d, ^3^*J* = 3.6 Hz, 1H, H1′_A2_), 5.83 (d, ^3^*J*_H,H_ = 4.0 Hz, 1H, H1′_U3_), 5.70 (m, 2H, H5_U_, H1′_U1_), 5.66 (d, ^3^*J*_H,H_ = 8.1 Hz, 1H, H5_U_), 5.41 (m, 1H, C*H*=C_N6-isopent_), 4.80 (m, overlapped with HDO, 2H, H2′_A2_, H3′_A2_), 4.54 (m, 1H, H4′_A2_), 4.48 (m, 1H, H3′_U1_), 4.35–4.28 (m, 4H, H2′_U1_, H3′_U3_, H5′_A2_, H5′_U3_), 4.25–4.24 (m, 2H, H4′_U3_, H2′_U3_), 4.22–4.10 (m, 5H, H4′_U1_, H5″_A2_, H5″_U3_, CH_2N6-isopent_), 3.82–3.73 (m, 2H, H5′_U1_, H5″_U1_), 3.20 (q, ^3^*J*_H,H_ = 7.3 Hz, 12H, CH_2TEAH+_), 1.76 (m, 6H, 2 × CH_3N6-isopent_), 1.28 (t, ^3^*J*_H,H_ = 7.3 Hz, 18H, CH_3TEAH+_) ppm; ^31^P NMR (202.5 MHz, D_2_O, 25 °C): δ *=* 0.17 (s, 1P,P_A-U_), 0.12 (s, 1P,P_U-A_); HRMS (ESI) *m*/*z*: [M + H]^+^ calcd for C_33_H_42_N_9_O_20_P_2_^–^ 946.20268, found: 946.20454; physical description: white amorphous solid.

##### p^Bn6^A_m_pG (**12**)

^1^H NMR (500 MHz, D_2_O, 25 °C): δ = 8.40 (s, 1H, H8_A_), 8.13 (s, 1H, H2_A_), 7.93 (s, 1H, H8_G_), 7.41–7.35 (m, 4H, *Ar*H_Bn_), 7.31 (m, 1H, *Ar*H-4_Bn_), 6.10 (d, ^3^*J*_H,H_ = 5.2 Hz, 1H, H1′_A_), 5.83 (d, ^3^*J*_H,H_ = 5.2 Hz, 1H, H1′_G_), 4.92 (m, 1H, H3′_A_), 4.82 (m, overlapped with HDO, 2H, CH_2Bn_), 4.73 (m, 1H, H2′_G_), 4.50–4.44 (m, 3H, H3′_G_, H2′_A_, H4′_A_), 4.34 (m, 1H, H4′_G_), 4.21 (m, 2H, H5′_G_, H5″_G_), 4.07 (m, 2H, H5′_A_, H5″_A_), 3.49 (s, 3H, CH_32′-O-Me_), 3.19 (q, ^3^*J*_H,H_ = 7.3 Hz, 6H, CH_2TEAH+_), 1.27 (t, ^3^*J*_H,H_ = 7.3 Hz, 9H, CH_3TEAH+_) ppm; ^31^P NMR (202.5 MHz, D_2_O, 25 °C): δ = 1.10 (s, 1P, P_5′_), 0.05 (s, 1P, P_A-G_) ppm; HRMS (ESI) *m*/*z* calcd for C_28_H_33_N_10_O_14_P_2_^–^ [M-H]^−^: 795.1658, found: 795.16658; physical description: white amorphous solid.

##### p^hex6^A_m_pG (**13**)

^1^H NMR (500 MHz, D_2_O, 25 °C): δ = 8.38 (s, 1H, H8_A_), 8.14 (s, 1H, H2_A_), 7.91 (s, 1H, H8_G_), 6.09 (d, ^3^*J*_H,H_ = 5.0 Hz, 1H, H1′_A_), 5.82 (d, ^3^*J*_H,H_ = 5.1 Hz, 1H, H1′_G_), 4.92 (m, 1H, H3′_A_), 4.72 (m, 1H, H2′_G_), 4.50–4.43 (m, 3H, H3′_G_, H2′_A_, H4′_A_), 4.34 (m, 1H, H4′_G_), 4.21 (m, 2H, H5′_G_, H5″_G_), 4.07 (m, 2H, H5′_A_, H5″_A_), 3.59 (m, 2H, CH_2_-6_hex_), 3.50 (s, 3H, CH_32′-O-Me_), 3.20 (q, ^3^*J*_H,H_ = 7.3 Hz, 6H, CH_2TEAH+_), 2.33 (t, ^4^*J*_H,H_ = 2.6 Hz, 1H, H1_hex_), 2.27 (td, ^3^*J*_H,H_ = 7.0 Hz, ^4^*J*_H,H_ = 2.6 Hz, CH_2_-3_hex_), 1.80 (m, 2H, CH_2_-5_hex_), 1.64 (m, 2H, CH_2_-4_hex_), 1.28 (t, ^3^*J*_H,H_ = 7.3 Hz, 9H, CH_3TEAH+_) ppm; ^31^P NMR (202.5 MHz, D_2_O, 25 °C): δ = 1.24 (s, 1P, P_5′_), 0.04 (s, 1P, P_A-G_) ppm; HRMS (ESI) *m*/*z* calcd For C_27_H_35_N_10_O_14_P_2_^–^ [M-H]^−:^ 785.1815, found: 785.18225; physical description: white amorphous solid.

##### p^ap6^A_m_pApG (**14**)

^1^H NMR (500 MHz, D_2_O, 25 °C): δ = 8.38 (s, 1H, H8_A1_), 8.14 (s, 1H, H8_A2_), 8.06 (s, 1H, H_purine_), 7.90 (s, 1H, H_purine_), 7.80 (s, 1H, H_purine_), 6.03 (d, ^3^*J*_H,H_ = 3.9 Hz, 1H, H1′_A1_), 5.88 (d, ^3^*J*_H,H_ = 3.1 Hz, 1H, H1′_A2_), 5.71 (d, ^3^*J*_H,H_ = 5.4 Hz, 1H, H1′_G3_), 4.88 (m, 1H, H3′_A1_), 4.74 (m, 1H, H3′_A2_), 4.57 (m, 3H, H2′_A1_, H2′_A2_, H2′_G3_), 4.52 (m, 1H, H4′_A2_), 4.48 (m, 1H, H4′_A1_), 4.39 (m, 1H, H3′_G3_), 4.31–4.26 (m, 3H, H4′_G3_, H5′_A2_, H5′_G3_), 4.25–4.08 (m, 4H, H5″_A2_, H5′_A1_, H5″_G3_, H5″_A1_), 3.61–3.51 (m, 2H, CH_2aminopropyl_), 3.58 (s, 3H, CH_32′-O-Me_), 3.07 (s, 2H, CH_2aminopropyl_), 2.00 (m, 2H, CH_2aminopropyl_) ppm; ^31^P NMR (202.5 MHz, D_2_O, 25 °C): δ = 1.96 (s, 1P, P_5′_), 0.14 (s, 1P, P_A2-G3_), −0.24 (s, 1P, P_A1-A2_) ppm; HRMS (ESI) *m*/*z* calcd for C_34_H_46_N_16_O_20_P_3_^–^, [M-H]^−:^ 1091.22926, found: 1091.23012; physical description: white amorphous solid.

##### p^iPr6^A_m_pG (**15**)

^1^H NMR (500 MHz, D_2_O, 25 °C): δ = 8.39 (s, 1H, H8_A_), 8.16 (s, 1H, H2_A_), 7.93 (s, 1H, H8_G_), 6.09 (d, ^3^*J*_H,H_ = 5.3 Hz, 1H, H1′_A_), 5.85 (d, ^3^*J*_H,H_ = 5.3 Hz, 1H, H1′_G_), 4.93 (m, 1H, H3′_A_), 4.76 (m, overlapped with HDO, H2′_G_), 4.50–4.45 (m, 3H, H2′_A_, H3′_G_, H4′_A_), 4.35 (m, 1H, H4′_G_), 4.24–4.18 (m, H5′_G_, H5″_G_), 4.12–4.01 (m, 2H, H5′_A_, H5″_A_), 3.49–3.45 (m, 4H, CH_iPr_, CH_32′-O-Me_), 3.20 (q, ^3^*J*_H,H_ = 7.3 Hz, 6H, CH_2TEAH+_), 1.31 (m, 6H, 2 × CH_3 iPr_), 1.28 (t, ^3^*J*_H,H_ = 7.3 Hz, 9H, CH_3TEAH+_) ppm; ^31^P NMR (202.5 MHz, D_2_O, 25 °C): δ = 1.05 (s, 1P, P_5′_), 0.06 (s, 1P, P_A-G_) ppm; HRMS (ESI) *m*/*z* calcd for C_24_H_33_N_10_O_14_P_2_^–^, [M-H]^−:^ 747.16584, found: 747.16699; physical description: white amorphous solid.

##### p^PhNCO6^ApG (**16**)

^1^H NMR (500 MHz, D_2_O, 25 °C): δ = 8.47 (s, 1H, H8_A_), 8.43 (s, 1H, H2_A_), 7.70 (s, 1H, H8_G_), 7.28 (m, 2H, *Ar*H-2,6_PhNHCO_), 7.20 (m, 2H, *Ar*H-3,5_PhNHCO_), 6.99 (m, 1H, *Ar*H-4_PhNHCO_), 5.94 (m, 1H, H1′_A_), 5.71 (d, ^3^*J*_H,H_ = 4.2 Hz, 1H, H1′_G_), 4.78 (m, 2H, H2′_A_, H3′_A_), 4.53–4.47 (m, 2H, H2′_G_, H4′_A_), 4.40 (m, 1H, H3′_G_), 4.33–4.30 (m, 2H, H4′_G_, H5′_G_), 4.27–4.25 (m, 1H, H5′_A_), 4.17–4.12 (m, 2H, H5″_A_, H5″_G_) ppm; ^31^P NMR (202.5 MHz, D_2_O, 25 °C): δ = 1.28 (s, 1P, P_5′_), 0.05 (s, 1P, P_A-G_) ppm; HRMS (ESI) *m*/*z* calcd for C_27_H_30_N_11_O_15_P_2_^–^ [M-H]^−:^ 810.14036, found: 810.14200; physical description: white amorphous solid.

##### U^g6^AU (**17**)

^1^H NMR (500 MHz, D_2_O, 25 °C): δ = 8.61 (s, 1H, H8_A2_), 8.60 (s, 1H, H2_A2_), 7.79 (d, *J*_H,H_ = 8.1 Hz, 1H, H6_U_), 7.77 (d, ^3^*J*_H,H_ = 8.1 Hz, 1H, H6_U_), 6.21 (d, ^3^*J*_H,H_ = 3.6 Hz, 1H, H1′_A2_), 5.85 (d, ^3^*J*_H,H_ = 4.3 Hz, 1H, H1′_U3_), 5.73 (d, ^3^*J*_H,H_ = 8.1 Hz, 1H, H5_U_), 5.67 (d, ^3^*J*_H,H_ = 4.2 Hz, 1H, H1′_U1_), 5.65 (d, ^3^*J*_H,H_ = 8.1 Hz, 1H, H5_U_), 4.90–4.83 (m, 2H, H3′_A2_, H2′_A2_), 4.57 (m, 1H, H4′_A2_), 4.49 (m, 1H, H3′_U1_), 4.39–4.33 (m, 1H, H5′_A2_), 4.33–4.29 (m, 3H, H2′_U1_, H5′_U3_, H3′_U3_), 4.27–4.24 (m, 2H, H2′_U3_, H4′_U3_), 4.23–4.17 (m, 2H, H4′_U1_, H5″_A2_), 4.16–4.12 (m, 1H, H5″_U3_), 4.10 (s, 2H, CH_2Gly-α_) 3.78–3.75 (m, 2H, H5′_U1_, H5″_U1_), 3.20 (q, ^3^*J*_H,H_ = 7.3 Hz, 6H, CH_2TEAH+_), 2.79 (s, 3H, CH_3CONHCH3_), 1.28 (t, ^3^*J*_H,H_ = 7.3 Hz, 9H, CH_3TEAH+_) ppm; ^31^P NMR (202.5 MHz, D_2_O, 25 °C): δ = 0.18 (s, 1P,P_A-U_), 0.05 (s, 1P, P_U-A_); HRMS (ESI) *m*/*z* calcd for C_32_H_40_N_11_O_22_P_2_^–^ [M-H]^−:^ 992.18301, found: 992.18614; physical description: white amorphous solid.

##### U^g6m6^AU (**18**)

^1^H NMR (500 MHz, D_2_O, 25 °C): δ = 8.66 (s, 1H, H8_A2_), 8.65 (s, 1H, H2_A2_), 7.80 (d, ^3^*J*_H–H_ = 8.1 Hz, 1H, H6_U_), 7.77 (d, ^3^*J*_H–H_ = 8.1 Hz, 1H, H6_U_), 6.24 (d, ^3^*J*_H–H_ = 3.5 Hz, 1H, H1′_A2_), 5.84 (d, ^3^*J*_H–H_ = 4.2 Hz, 1H, H1′_U3_), 5.74 (d, ^3^*J*_H–H_ = 8.1 Hz, 1H, H5_U_), 5.68 (d, ^3^*J*_H–H_ = 4.3 Hz, 1H, H1′_U1_), 5.63 (d, ^3^*J*_H–H_ = 8.1 Hz, 1H, H5_U_), 4.91–4.87 (m, 1H, H3′_A2_), 4.86–4.84 (m, 1H, H2′_A2_), 4.57 (m, 1H, H4′_A2_), 4.50 (m, 1H, H3′_U1_), 4.39–4.35 (m, 2H, H2′_U1_, H5′_A2_), 4.34–4.29 (m, 2H, H3′_U3_, H5′_U3_), 4.28–4.19 (m, 3H, H4′_U3_, H2′_U3_, H5″_A2_), 4.20–4.12 (m, 2H, H4′_U1_, H5″_U3_), 4.04 (s, 2H, CH_2Gly-α_), 3.76 (m, 2H, H5′_U1_, H5″_U1_), 3.72 (s, 3H, CH_3N6-Me_), 3.21 (q, ^3^*J*_H,H_ = 7.3 Hz, 6H, CH_2TEAH+_), 2.78 (s, 3H, CH_3CONHCH3_), 1.28 (t, ^3^*J*_H,H_ = 7.3 Hz, 9H, CH_3TEAH+_) ppm; ^31^P NMR (202.5 MHz, D_2_O, 25 °C): δ = 0.16 (s, 1P,P_A-U_), 0.05 (s, 1P, P_U-A_); HRMS (ESI) *m*/*z*: [M + H]^+^ calcd for C_33_H_42_N_11_O_22_P_2_^–^ 1006.19866, found: 1006.20085; physical description: white amorphous solid.

##### p^m3^CpG (**19**)

^1^H NMR (500 MHz, D_2_O, 25 °C): δ = 8.13 (d, ^3^*J*_H,H_ = 8.1 Hz, 1H, H6_C_), 8.02 (s, 1H, H8_G_), 6.31 (d, ^3^*J*_H,H_ = 8.0 Hz, 1H, H5_C_), 5.88 (d, ^3^*J*_H,H_ = 5.9 Hz, 1H, H1′_G_), 5.81 (d, ^3^*J*_H,H_ = 3.9 Hz, 1H, H1′_C_), 4.82 (m, overlapped with HDO, 1H, H2′_G_), 4.62 (m, 1H, H3′_C_), 4.49 (dd, ^3^*J*_H,H_ = 5.3 Hz, ^3^*J*_H,H_ = 3.7 Hz, 1H, H3′_G_), 4.39 (m, 1H, H4′_C_), 4.36–4.30 (m, 2H, H2′_C_, H4′_G_), 4.24–4.22 (m, 1H, H5′_G_), 4.19–4.09 (m, 2H, H5′_C_, H5″_G_), 4.09–4.02 (m, 1H, H5″_C_), 3.45 (s, 3H, CH_3N3-Me_), 3.20 (q, ^3^*J*_H,H_ = 7.3 Hz, 6H, CH_2TEAH+_), 1.28 (t, ^3^*J*_H,H_ = 7.3 Hz, 9H, CH_3TEAH+_) ppm; ^31^P NMR (202.5 MHz, D_2_O, 25 °C): δ = 0.90 (s, 1P, P_5′_), 0.19 (s, 1P, P_C-G_) ppm; HRMS (ESI) *m*/*z*: [M + H]^+^ calcd for C_20_H_27_N_8_O_15_P_2_^–^ 681.10766, found: 681.10921; physical description: white amorphous solid.

##### p^m4^CpG (**20**)

^1^H NMR (500 MHz, D_2_O, 25 °C): δ = 8.04 (s, 1H, H8_G_), 7.94 (d, ^3^*J*_H,H_ = 7.6 Hz, 1H, H6_C_), 5.94 (d, ^3^*J*_H,H_ = 3.8 Hz, 1H, H1′_C_), 5.88–5.85 (m, 2H, H1′_G_, H5_C_), 4.75–4.73 (m, 1H, H2′_G_), 4.62 (m, 1H, H3′_C_), 4.54 (m, 1H, H3′_G_), 4.41 (m, 1H, H2′_C_), 4.35 (m, 2H, H4′_G_, H4′_C_), 4.24–4.16 (m, 2H, H5′_G_, H5″_G_), 4.12–4.08 (m, 1H, H5′_C_), 3.98–3.94 (m, 1H, H5″_C_), 3.20 (q, ^3^*J*_H,H_ = 7.3 Hz, 6H, CH_2TEAH+_), 2.84 (s, 3H, CH_3*N*4-Me_), 1.28 (t, ^3^*J*_H,H_ = 7.3 Hz, 9H, CH_3TEAH+_) ppm; ^1^P NMR (202.5 MHz, D_2_O, 25 °C): δ = 4.37 (s, 1P, P_5′_), 0.33 (s, 1P, P_C-G_) ppm; HRMS (ESI) *m*/*z*: [M + H]^+^ calcd for C_20_H_27_N_8_O_15_P_2_^–^ 681.10766, found: 681.10877; physical description: white amorphous solid.

##### p^m^_2_^3,4^CpG (**21**)

^1^H NMR (500 MHz, D_2_O, 25 °C): δ = 8.01 (s, 1H, H8_G_), 7.94 (d, ^3^*J*_H,H_ = 8.1 Hz, 1H, H6_C_), 5.92 (d, ^3^*J*_H,H_ = 8.1 Hz, 1H, H5_C_), 5.91 (d, ^3^*J*_H,H_ = 3.7 Hz, 1H, H1′_C_), 5.87 (d, ^3^*J*_H,H_ = 5.8 Hz, 1H, H1′_G_), 4.82 (m, 1H, H2′_G_), 4.73 (m, 1H, H3′_C_), 4.50 (dd, ^3^*J*_H,H_ = 5.2, 3.7 Hz, 1H, H3′_G_), 4.35–4.32 (m, 2H, H4′_C_, H4′_G_), 4.18–4.16 (m, 2H, H5′_G_, H5″_G_), 4.12–4.07 (m, 2H, H2′_C_, H5′_C_), 4.06–4.02 (m, 1H, H5″_C_), 3.52 (s, 3H, CH_3N3-Me_), 3.20 (m, 9H, CH_3N4-Me_, 3 × CH_2TEAH+_), 1.28 (t, ^3^*J*_H,H_ = 7.3 Hz, 9H, CH_3TEAH+_) ppm; ^1^P NMR (202.5 MHz, D_2_O, 25 °C): δ = 1.01 (s, 1P, P_5′_), −0.02 (s, 1P, P_C-G_); HRMS (ESI) *m*/*z*: [M + H]^+^ calcd for C_21_H_29_N_8_O_15_P_2_^–^ 695.12331, found: 695.12428; physical description: white amorphous solid.

##### p^m3^U_m_pG (**22**)

^1^H NMR (500 MHz, D_2_O, 25 °C): δ = 8.31 (d, ^3^*J*_H,H_ = 8.2 Hz, 1H, H6_U_), 8.00 (s, 1H, H8_G_), 6.44 (d, ^3^*J*_H,H_ = 8.2 Hz, 1H, H5_U_), 5.86 (d, ^3^*J*_H,H_ = 6.2 Hz, 1H, H1′_G_), 5.79 (d, ^3^*J*_H,H_ = 3.3 Hz, 1H, H1′_U_), 4.81 (m, 1H, H2′_G_), 4.64 (m, 1H, H3′_U_), 4.49 (dd, ^3^*J*_H,H_ = 5.2, ^3^*J*_H,H_ = 3.4 Hz, 1H, H3′_G_), 4.38–4.41 (m, 2H, H2′_U_, H4′_U_), 4.33 (m, 1H, H4′_G_), 4.25–4.22 (m, 1H, H5′_G_), 4.20–4.13 (m, 2H, H5′_U_, H5″_G_), 4.11–4.01 (m, 1H, H5″_U_), 3.42 (s, 3H, CH_32′-OMe_), 3.20 (q, ^3^*J*_H,H_ = 7.3 Hz, 6H, CH_2TEAH+_), 3.15 (s, 3H, CH_3*N*3-Me_), 1.28 (t, ^3^*J*_H,H_ = 7.3 Hz, 9H, CH_3TEAH+_) ppm; ^31^P NMR (202.5 MHz, D_2_O, 25 °C): δ = 1.52 (s, 1P, P_5′_), 0.21 (s, 1P, P_U-G_) ppm; HRMS (ESI) *m*/*z*: [M + H]^+^ calcd for C_21_H_28_N_7_O_16_P_2_^–^ 696.10732, found: 696.10792; physical description: white amorphous solid.

##### d(G^2nBn3^TC) (**23**)

^1^H NMR (500 MHz, D_2_O, 25 °C): δ = 8.01 (m, 1H, *Ar*H-3_nBn_), 7.94 (s, 1H, H8_G_), 7.90 (d, ^3^*J*_H,H_ = 7.7 Hz, 1H, H6_C_), 7.74 (d, ^4^*J*_H,H_ = 0.9 Hz, 1H, H6_T_), 7.38 (m, 2H, *Ar*H-4_nBn_, *Ar*H-5_nBn_), 7.03 (m, 1H, *Ar*H6-_nBn_), 6.27 (dd, ^3^*J*_H,H_ = 7.9 Hz, ^3^*J*_H,H_ = 6.2 Hz, 1H, H1′_T_), 6.15 (m, 2H, H1′_C_, H1′_G_), 6.06 (d, ^3^*J*_H,H_ = 7.7 Hz, 1H, H5_C_), 5.33 (m, 2H, CH_2nBnT_), 4.91 (m, 2H, H3′_G_, H3′_T_), 4.52 (m, 1H, H3′_C_), 4.37 (m, 1H, H4′_T_), 4.30 (m, 1H, H4′_G_), 4.24 (ddd, ^2^*J*_H,H_ = 11.4 Hz, ^3^*J*_H,P_ = 4.4 Hz, ^3^*J*_H,H_ = 2.2 Hz, 1H, H5′_T_), 4.18–4.10 (m, 3H, H5″_T_, H4′_C_, H5′_C_), 4.08 (m, 1H, H5″_C_), 3.80 (m, 2H, H5′_G_, H5″_G_), 3.20 (q, ^3^*J*_H,H_ = 7.3 Hz, 6H, CH_2TEAH+_), 2.75–2.63 (m, 2H, H2′_G_, H2″_G_), 2.55 (ddd, ^2^*J*_H,H_ = 13.9 Hz, ^3^*J*_H,H_ = 6.0 Hz, ^3^*J*_H,H_ = 2.6 Hz, 1H, H2′_T_), 2.43–2.34 (m, 2H, H2″_T_, H2′_C_), 2.22 (m, 1H, H2″_C_), 1.89 (d,^4^*J*_H,H_ = 0.9 Hz, 3H, CH_35-T_), 1.28 (t, ^3^*J*_H,H_ = 7.3 Hz, 9H, CH_3TEAH+_) ppm; ^31^P NMR (202.5 MHz, D_2_O, 25 °C): δ = −0.06 (s, 1P, P_T-C_), −0.13 (s, 1P, P_G-T_) ppm; HRMS (ESI) *m*/*z*: [M + H]^+^ calcd for C_36_H_42_N_11_O_19_P_2_^–^ 994.21391, found: 994.21533; physical description: pale yellow amorphous solid.

##### U^m2^GU (**24**)

^1^H NMR (500 MHz, D_2_O, 25 °C): δ = 8.10 (s, 1H, H8_G_), 7.85 (d, ^3^*J*_H,H_ = 8.2 Hz, 1H, H6_U_), 7.77 (d, ^3^*J*_H,H_ = 8.1 Hz, 1H, H6_U_), 5.98 (d, ^3^*J*_H,H_ = 3.6 Hz, 1H, H1′_G2_), 5.90 (d, ^3^*J*_H,H_ = 4.2 Hz, 1H, H1′_U3_), 5.80–5.78 (m, 2H, H1′_U1_, H5_U_), 5.76 (d, ^3^*J*_H,H_ = 8.1 Hz, 1H, H5_U_), 4.94–4.88 (m, 2H, H2′_G2_, H3′_G2_), 4.52–4.47 (m, 2H, H4′_G2_, H3′_U1_), 4.34–4.28 (m, 5H, H2′_U1_, H2′_U3_, H3′_U3_, H5′_G2_, H5′_U3_), 4.26 (m, 1H, H4′_U3_), 4.24–4.21 (m, 1H, H5_G2_), 4.19–4.13 (m, 2H, H4′_U1_, H5_U3_), 3.69 (dd, ^2^*J*_H,H_ = 12.9, ^3^*J*_H,H_ = 3.8 Hz, 1H, H5′_U1_), 3.60 (dd, ^2^*J*_H,H_ = 12.9, ^3^*J*_H,H_ = 2.8 Hz, 1H, H5″_U1_), 3.21 (q, ^3^*J*_H,H_ = 7.3 Hz, 6H, CH_2TEAH+_), 2.97 (s, 3H, CH_3N2-Me_) 1.28 (t, ^3^*J*_H,H_ = 7.3 Hz, 9H, CH_3TEAH+_) ppm; ^31^P NMR (202.5 MHz, D_2_O, 25 °C): δ = −0.76 (s, 2P, P_U-G_, P_G-U_) ppm; HRMS (ESI) *m*/*z*: [M + H]^+^ calcd for. C_29_H_36_N_9_O_21_P_2_^–^ 908.15065, found: 908.15258; physical description: white amorphous solid.

##### U^m1^GU (**25**)

^1^H NMR (500 MHz, D_2_O, 25 °C): δ = 8.03 (s, 1H, H8_G_), 7.85 (d, ^3^*J* = 8.1 Hz, 1H, H6_U_), 7.78 (d, ^3^*J*_H,H_ = 8.1 Hz, 1H, H6_U_), 5.91 (m, 2H, H1′_U3_, H1′_G2_), 5.75 (d, ^3^*J*_H,H_ = 4.2 Hz, 1H, H1′_U1_), 5.73 (d, ^3^*J*_H,H_ = 8.1 Hz, 1H, H5_U_), 5.72 (d, ^3^*J*_H,H_ = 8.1 Hz, 1H, H5_U_), 4.85 (m, 1H, H3′_G2_), 4.76 (m, overlapped with HDO, 1H, overlapped, H2′_G2_), 4.51–4.45 (m, 2H, H3′_U1_, H4′_G2_), 4.35 (m, 1H, H2′_U1_), 4.32–4.30 (m, 2H, H3′_U3_, H5′_G2_), 4.29–4.26 (m, 3H, H2′_U3_, H4′_U3_, H5′_U3_), 4.22–4.19 (m, 2H, H4′_U1_, H5″_G2_), 4.17–4.12 (m, 1H, H5″_U3_), 3.74 (d, ^3^*J*_H,H_ = 3.3 Hz, 2H, H5′_U1_, H5″_U1_), 3.43 (s, 3H, CH_3N1-Me_), 3.20 (q, ^3^*J*_H,H_ = 7.3 Hz, 6H, CH_2TEAH+_), 1.28 (t, ^3^*J*_H,H_ = 7.3 Hz, 9H, CH_3TEAH+_) ppm; ^31^P NMR (202.5 MHz, D_2_O, 25 °C): δ = 0.22 (s, 1P, P_G-U_), 0.12 (s, 1P, P_U-G_) ppm; HRMS (ESI) *m*/*z*: [M + H]^+^ calcd for. C_29_H_36_N_9_O_21_P_2_^–^ 908.15065, found: 908.15245; physical description: white amorphous solid.

#### Synthesis of cap-2 and cap-4

The p^m6^A_m_pG_m_pG and p^m6,6^A_m_pA_m_pC_m_p^m3^U_m_pA oligonucleotides were synthesized and isolated as triethylammonium salts according to the procedure described above.

##### Cap-2: m^7^Gppp^m6^A_m_pG_m_pG (**26**)

Triethylammonium salt of p^m6^A_m_pG_m_pG (520 mOD, 13.3 μmol) was dissolved in DMSO (265 μL), and *P*-imidazolide of *N*^7^-methylguanosine 5′-diphosphate [m^7^GDP-Im]^42^ (16.7 mg, 26.5 μmol) and anhydrous ZnCl_2_ (36.1 mg, 265 μmol) were added. The mixture was stirred for ca. 48 h, and the reaction was quenched by addition of 6.2 mL of aqueous solution of EDTA (20 mg/mL) and NaHCO_3_ (10 mg/mL). The product was isolated by ion-exchange chromatography on DEAE Sephadex (gradient elution 0–1.2 M TEAB) and purified by semi-preparative RP HPLC (gradient elution 0–15% acetonitrile in 0.05 M ammonium acetate buffer pH 5.9) to afford—after evaporation and repeated freeze-drying from water—ammonium salt of trinucleotide **26** m^7^Gppp^m6^A_m_pG_m_pG (13.4 mg, 370 mOD, 8.39 μmol, 63%) as a white amorphous solid. HRMS (ESI) *m*/*z*: [M + H]^+^ calcd for C_44_H_58_N_20_O_31_P_5_^–^ 1517.22704, found: 1517.22838.

##### Cap-4: m^7^Gppp^m6,6^A_m_pA_m_pC_m_p^m3^U_m_pA (**27**)

Triethylammonium salt of p^m6,6^A_m_pA_m_pC_m_p^m3^U_m_pA (206 mOD, 3.3 μmol) was dissolved in anhydrous DMF (132 μL) followed by the addition of imidazole (14.4 mg, 211 μmol), triethylamine (11 μL, 79 μmol), 2,2′-dithiodipyridine (17.4 mg, 79 μmol), and triphenylphospine (20.7 mg,79 μmol). After 5 h, the product was precipitated with a cold solution of NaClO_4_ (16.2 mg, 132 μmol) in acetonitrile (1.32 mL). The precipitate was centrifuged (6000 rpm, 6 min) in a 50 mL conical tube at 4 °C, washed with cold acetonitrile by centrifugation 3 times, and dried under reduced pressure. Thus obtained *P*-imidazolide was mixed with 7-methylguanosine 5′-diphosphate (30 mg, 33.0 μmol) in anhydrous DMSO (440 μL), followed by the addition of anhydrous ZnCl_2_ (72 mg, 528 μmol). The mixture was stirred for ca. 14 h, and the reaction was quenched by addition of 8.5 mL of aqueous solution of EDTA (20 mg/mL) and NaHCO_3_ (10 mg/mL). The product was isolated by ion-exchange chromatography on DEAE Sephadex using a linear gradient of TEAB (0–1.2 M) and purified by semi-preparative RP HPLC (gradient elution 0–15% acetonitrile in 0.05 M ammonium acetate buffer pH 5.9) to afford—after evaporation and repeated freeze-drying from water—ammonium salt of **27** m^7^Gppp^m6,6^A_m_pA_m_pCp^m3^U_m_pA (5.67 mg, 115 mOD, 1.88 μmol, 57%) as a white amorphous solid. HRMS (ESI) *m*/*z*: [M + H]^+^ calcd for C_66_H_89_N_25_O_44_P_7_^–^ 2152.36640, found: 2152.36410;

## Supporting Information Available

NMR and HRMS spectra and HPLC profiles of oligonucleotides (PDF)
