# Supplementary material for: Quick Access to Nucleobase-Modified Phosphoramidites for the Synthesis of Oligoribonucleotides Containing Post-Transcriptional Modifications and Epitranscriptomic Marks
Source: J Org Chem. 2022 Jul 20;87(15):10333–48. doi: 10.1021/acs.joc.2c01390 (PMC9361293; doi:10.1021/acs.joc.2c01390)

Quick Access to Nucleobase-Modified Phosphoramidites for the Synthesis of Oligoribonucleotides Containing Post-Transcriptional Modifications and Epitranscriptomic Marks

Kamil Ziemkiewicz[a], Marcin Warminski[b*], Radoslaw Wojcik[a], Joanna Kowalska[b], and Jacek Jemielity[a*]

[a] Centre of New Technologies, University of Warsaw, Banacha 2c, 02-097 Warsaw, Poland; E-mail: j.jemielity@cent.uw.edu.pl

[b] Division of Biophysics, Institute of Experimental Physics, Faculty of Physics, University of Warsaw, Pasteura 5, 02-093 Warsaw, Poland;
E-mail: warminski.marcin@uw.edu.pl

Supporting Information

Compounds characterization **S3**

(1a) N6 -methyladenosine phosphoramidite (5′-O-DMT-2′-O-Me-m^{6}A^{Pac}) S3

(1b) N6-Isopentenyladenosine phosphoramidite (5′-O-DMT-2′-O-TBDMS-i^{6}A^{Ac}) S15

(1c) N6-benzyladenosine phosphoramidite (5′-O-DMT-2′-O-Me-Bn^{6}A^{Pac}) S22

(1d) N6-hexynyladenosine phosphoramidite (5′-O-DMT-2′-O-Me-hex^{6}A^{Pac}) S30

(1e) N6-(3-phthalimidopropyl)adenosine phosphoramidite (5′-O-DMT-2′-O-Me-PhthNp^{6}A^{Pac}) S37

(1f) N6-isopropyladenosine phosphoramidite (5′-O-DMT-2′-O-Me-iPr^{6}A^{Pac}) S44

(1g) N6-methyladenosine phosphoramidite (5′-O-DMT-2′-O-TBDMS-m^{6}A^{Bz}) S50

(2) N1-methyladenosine phosphoramidite (5′-O-DMT-2′-O-TBDMS-^{m1}A^{Bz}) S57

(3a) N6-(N-phenylcarbamoyl)adenosine phosphoramidite (5′-O-DMT-2′-O-TBDMS-PhNHCO^{6}A) S64

(3b) N6-glycinylcarbamoyladenosine phosphoramidite (5′-O-DMT-2′-O-TBDMS-g^{6}A) S71

(4) N6-glycinylcarbamoyl-N6-methyladenosine phosphoramidite (5′-O-DMT-2′-O-TBDMS-g^{6}m^{6}A) S78

(5a) N4-methylcytidine phosphoramidite (5′-O-DMT-2′-O-TBDMS-m^{4}C^{Ac})....S85
(6a) N3-methylcytidine phosphoramidite (5′-O-DMT-2′-O-TBDMS-m^{3}C^{Ac})....S91
(6b) N3-methylcytidine phosphoramidite (5′-O-DMT-2′-O-TBDMS-m^{3}C^{Bz})....S97
(6c) N3-(2-nitrobenzyl)cytidine phosphoramidite (5′-O-DMT-2′-O-TBDMS-nb^{3}C^{Bz})....S103
(7) N6-(N-phenylcarbamoyl)cytidine phosphoramidite (5′-O-DMT-2′-O-TBDMS-PhNHCO4C)....S109
(8a) N3-methyluridine phosphoramidite (5′-O-DMT-2′-O-Me-m^{3}U$_{m}$)....S116
(8b) N3-(2-nitrobenzyl)thymidine phosphoramidite (5′-O-DMT- nb^{3}T)....S123
(9) N1-(4-O-acetyl)benzyl-N2-methylguanosine phosphoramidite (5′-O-DMT-2′-O-TBDMS-(4-OAc)Bn^{1}m^{2}G^{iBu})....S130
(10) N1-methylguanosine phosphoramidite (5′-O-DMT-2′-O-TBDMS-m^{1}G^{dmf})....S138
(11) Ui^{6}AU....S146
(12) p^{Bn6}A$_{m}$p....S153
(13) p^{hex6}A$_{m}$pG....S159
(14) p^{ap6}A$_{m}$pApG....S166
(15) p^{iPr6}A$_{m}$pG....S173
(16) p^{PhNCO6}ApG....S180
(17) U^{g6}AU....S187
(18) U^{g6m6}AU....S194
(19) p^{m3}CpG....S201
(20) p^{m4}CpG....S208
(21) p^{m3,4}CpG....S215
(22) p^{m3}U$_{m}$pG....S222
(23) G^{2nBn3}TC....S229
(24) U^{m2}GU....S236
(25) U^{m1}GU....S243
(26) m^{7}Gppp^{m6}A$_{m}$pG$_{m}$pG....S250
(27) m^{7}Gppp^{m6,6}A$_{m}$pA$_{m}$pC$_{m}$p^{m3}U^{m}pA....S252

Compounds characterization

(1a) *N6* -methyladenosine phosphoramidite (5′-*O*-DMT-2′-*O*-Me-m^{6}A^{Pac})	
**MS (+) ESI**<br>(Calc. $[M+H]^+$ $C_{50}H_{59}N_7O_9P^+$, 932.41064) *Diastereomer 1*	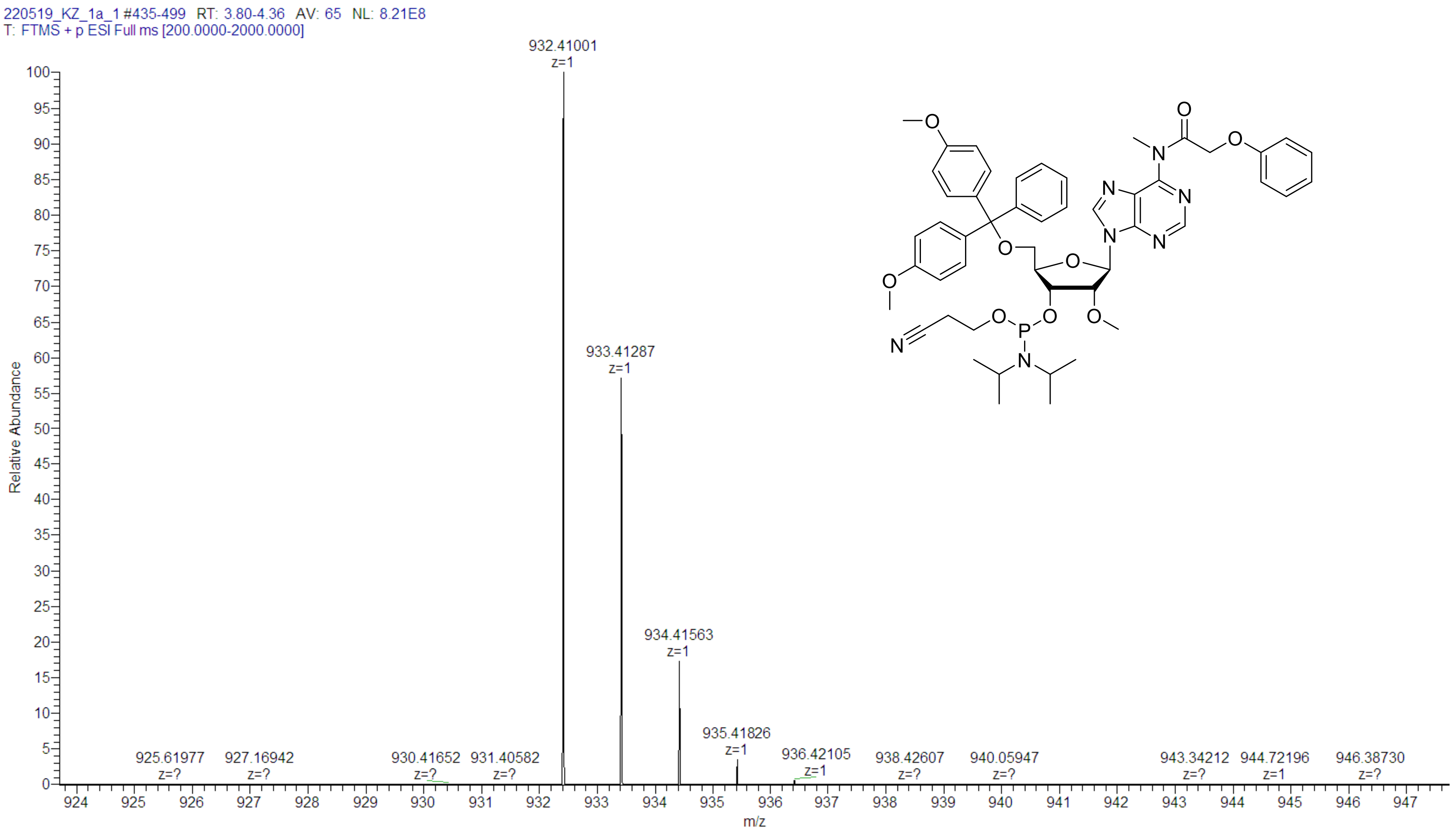

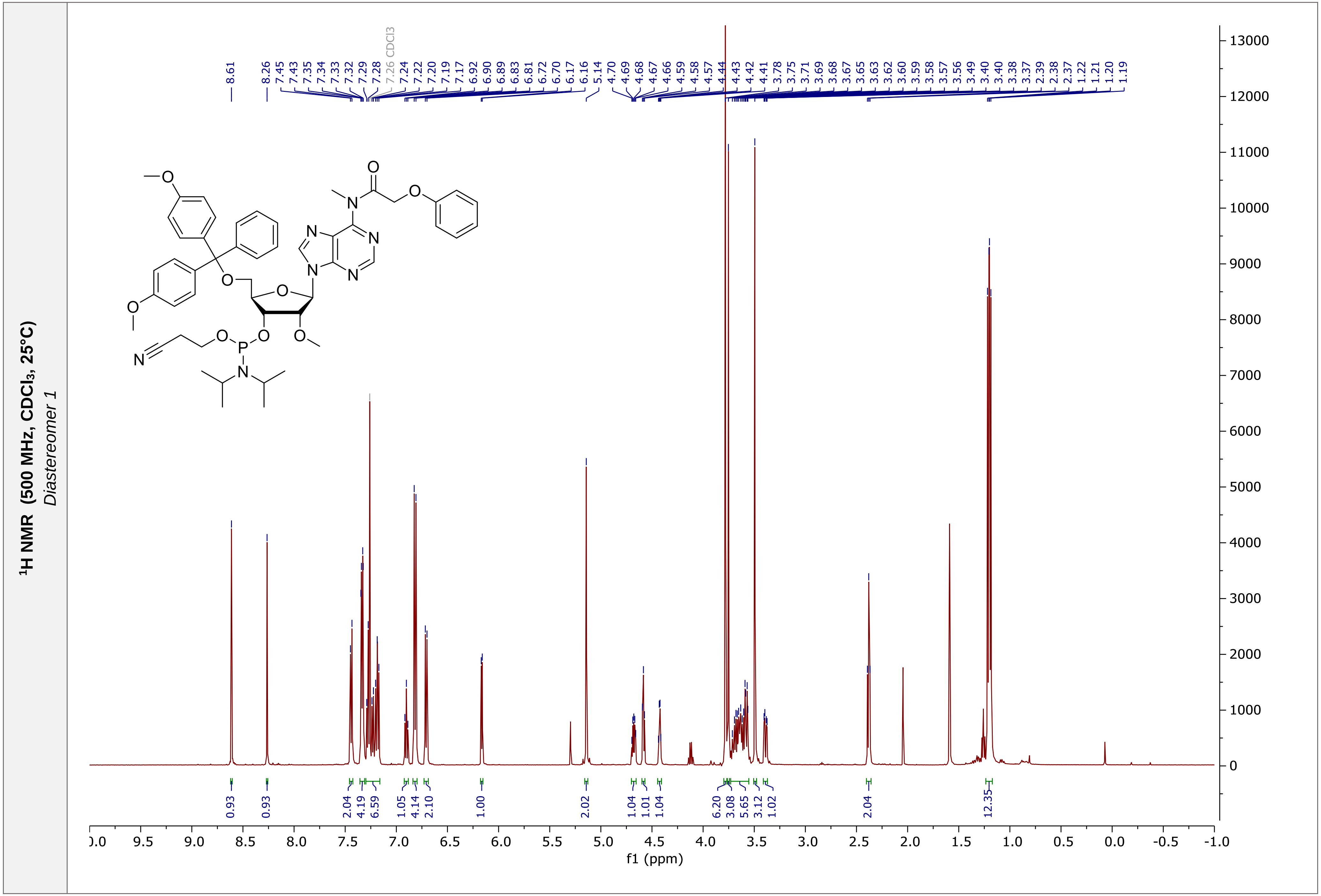
^{1}H NMR (500 MHz, CDCl$_3$, 25°C)
Diastereomer 1
f1 (ppm)

^{31}**P NMR (202.5 MHz, CDCl$_3$, 25°C)**

Diastereomer 1

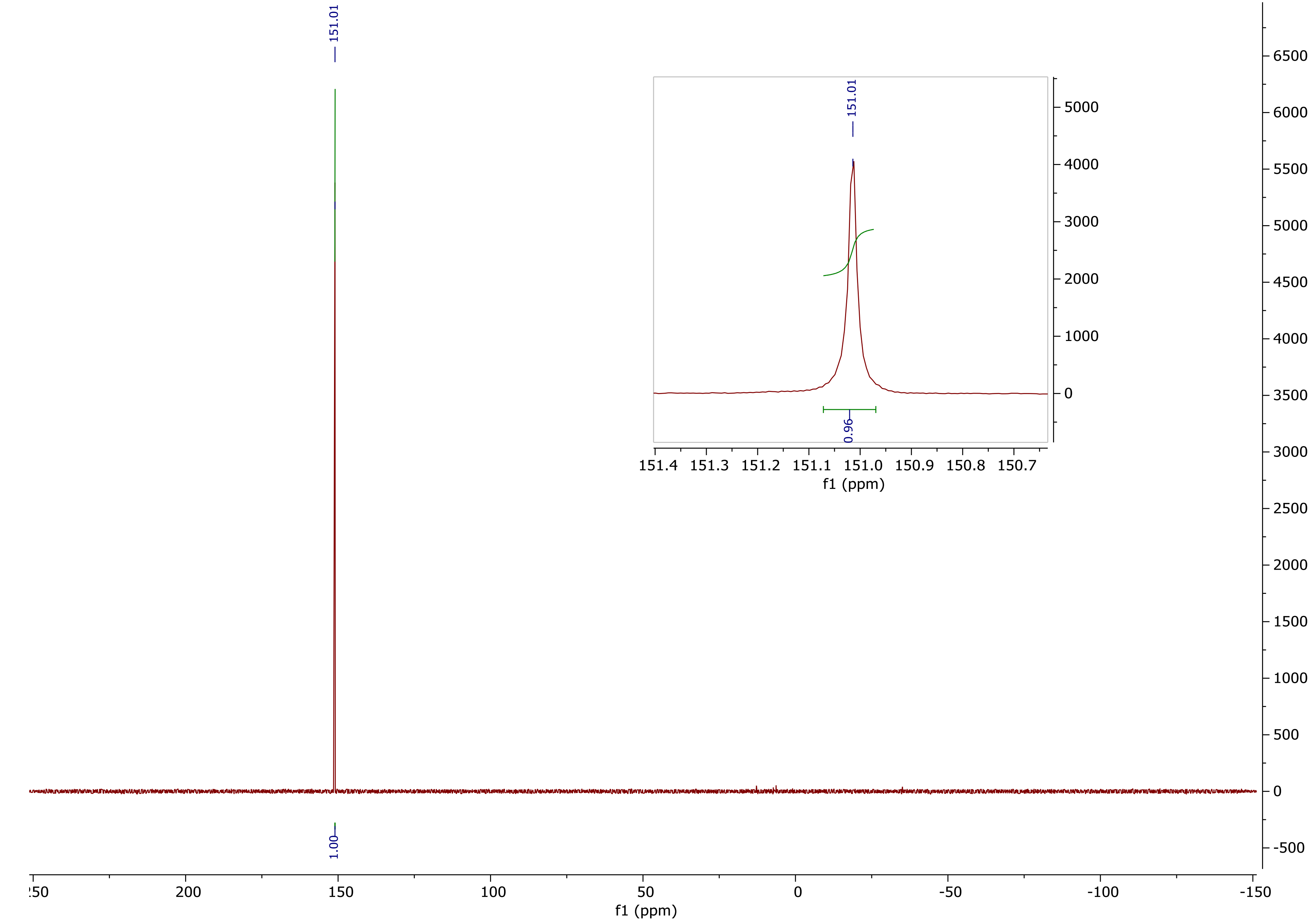

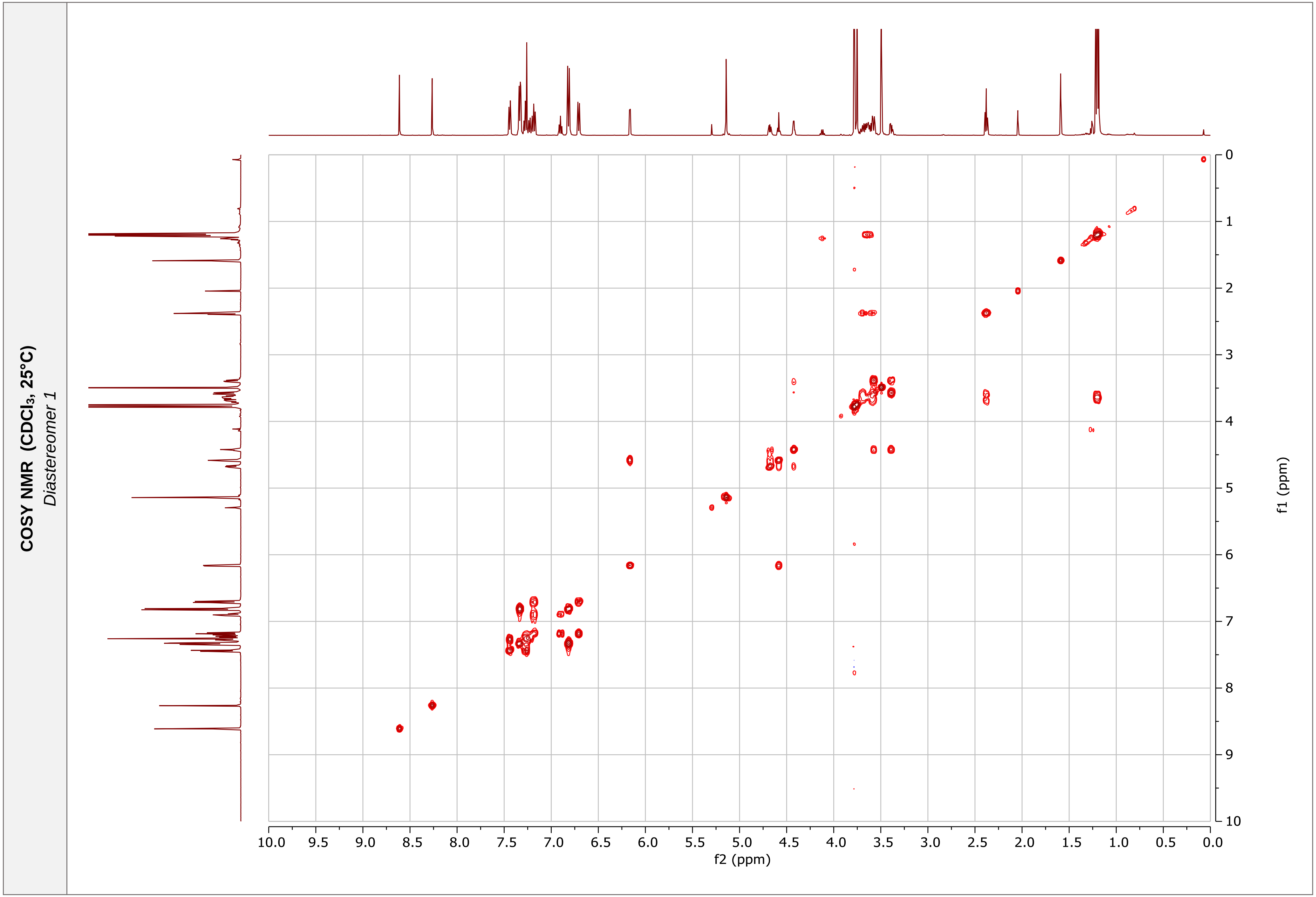
COSY NMR (CDCl$_3$, 25°C)
Diastereomer 1
f2 (ppm)
f1 (ppm)
10.0 9.5 9.0 8.5 8.0 7.5 7.0 6.5 6.0 5.5 5.0 4.5 4.0 3.5 3.0 2.5 2.0 1.5 1.0 0.5 0.0
0 1 2 3 4 5 6 7 8 9 10

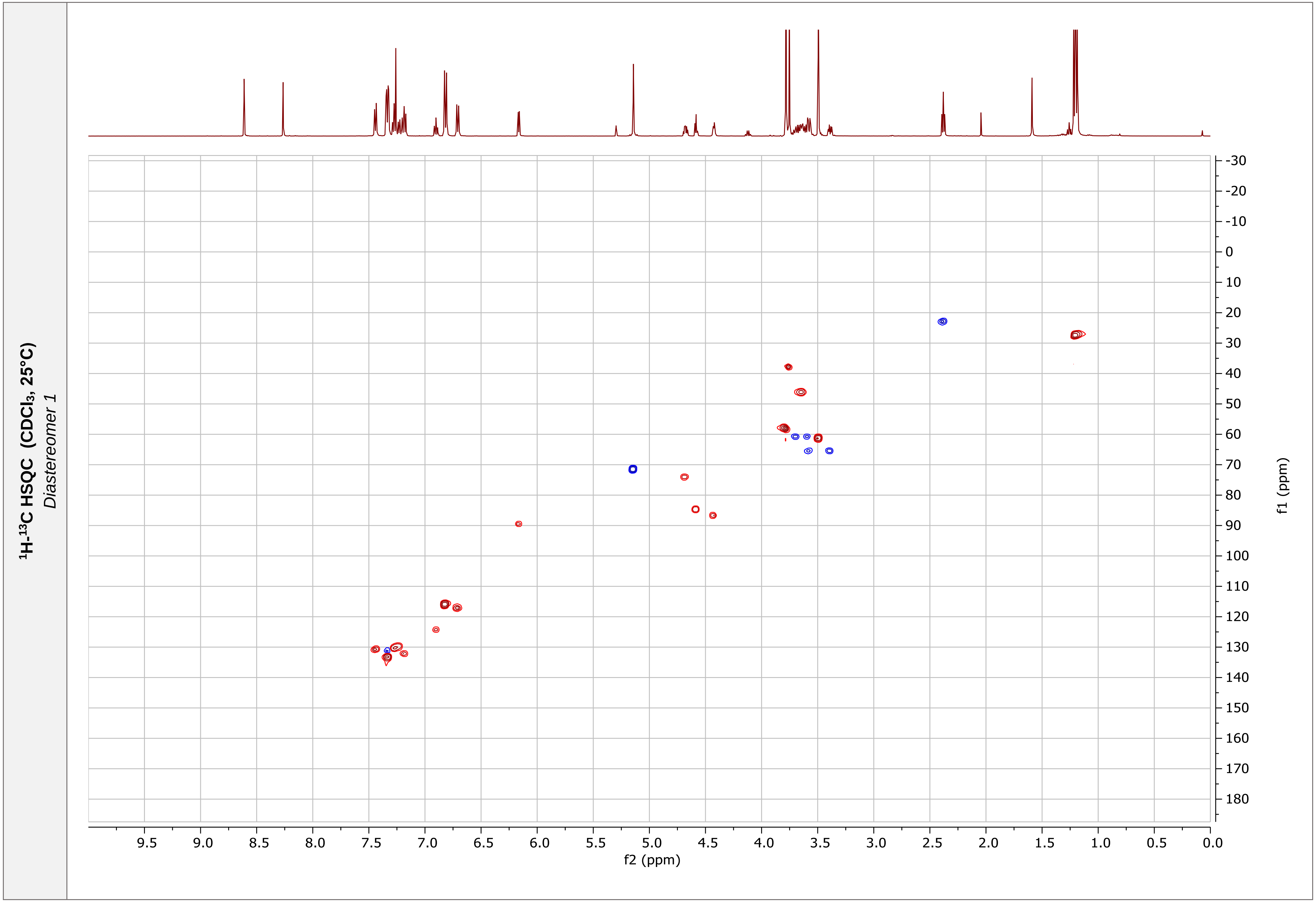

^{1}H-^{13}C HSQC (CDCl$_3$, 25°C)
Diastereomer 1
f2 (ppm)
f1 (ppm)

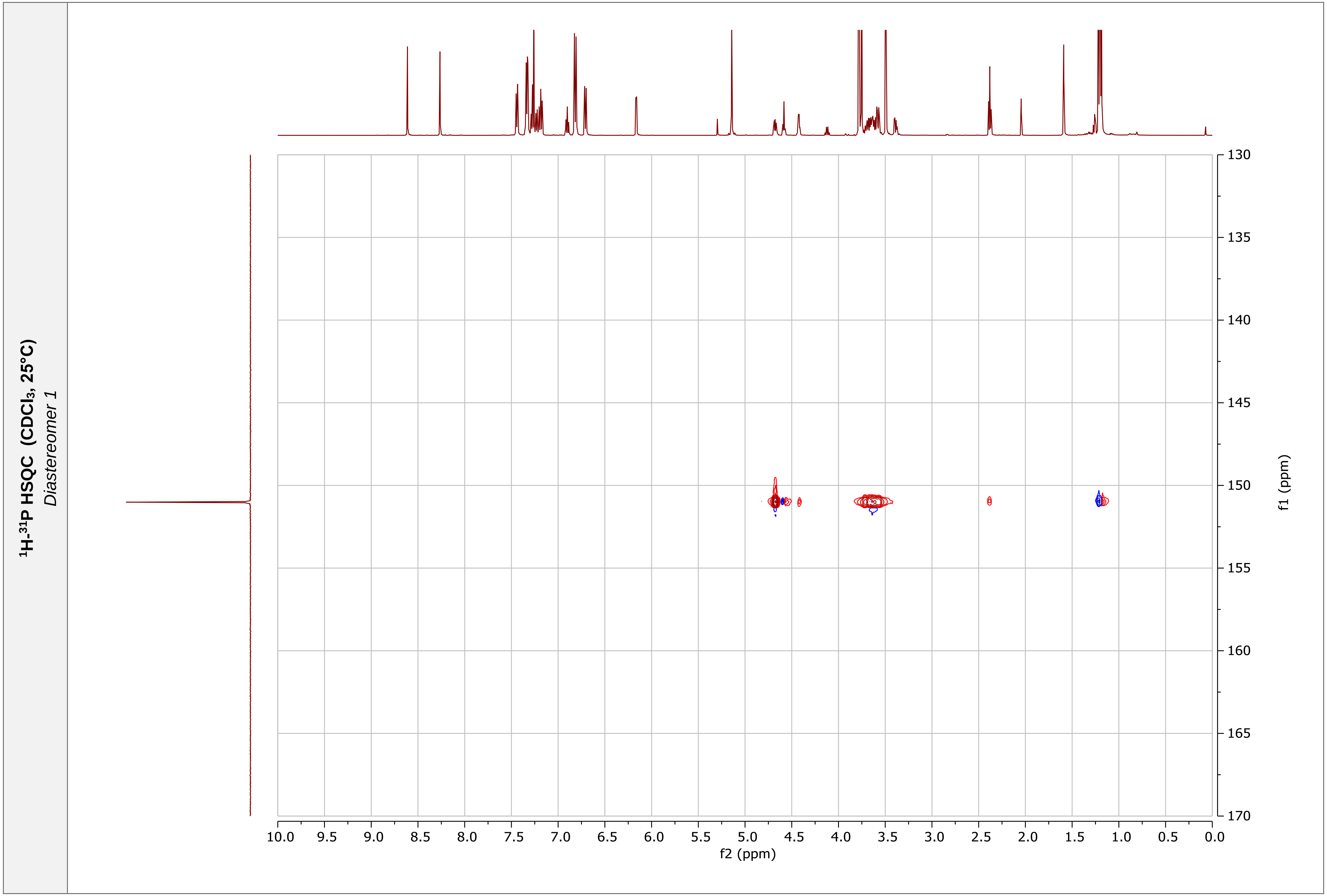
1H-31P HSQC (CDCl3, 25°C)
Diastereomer 1
f2 (ppm)
f1 (ppm)

MS (+) ESI

(Calc. $[M+H]^+$ $C_{50}H_{59}N_7O_9P^+$, 932.41064) *Diastereomer 2*

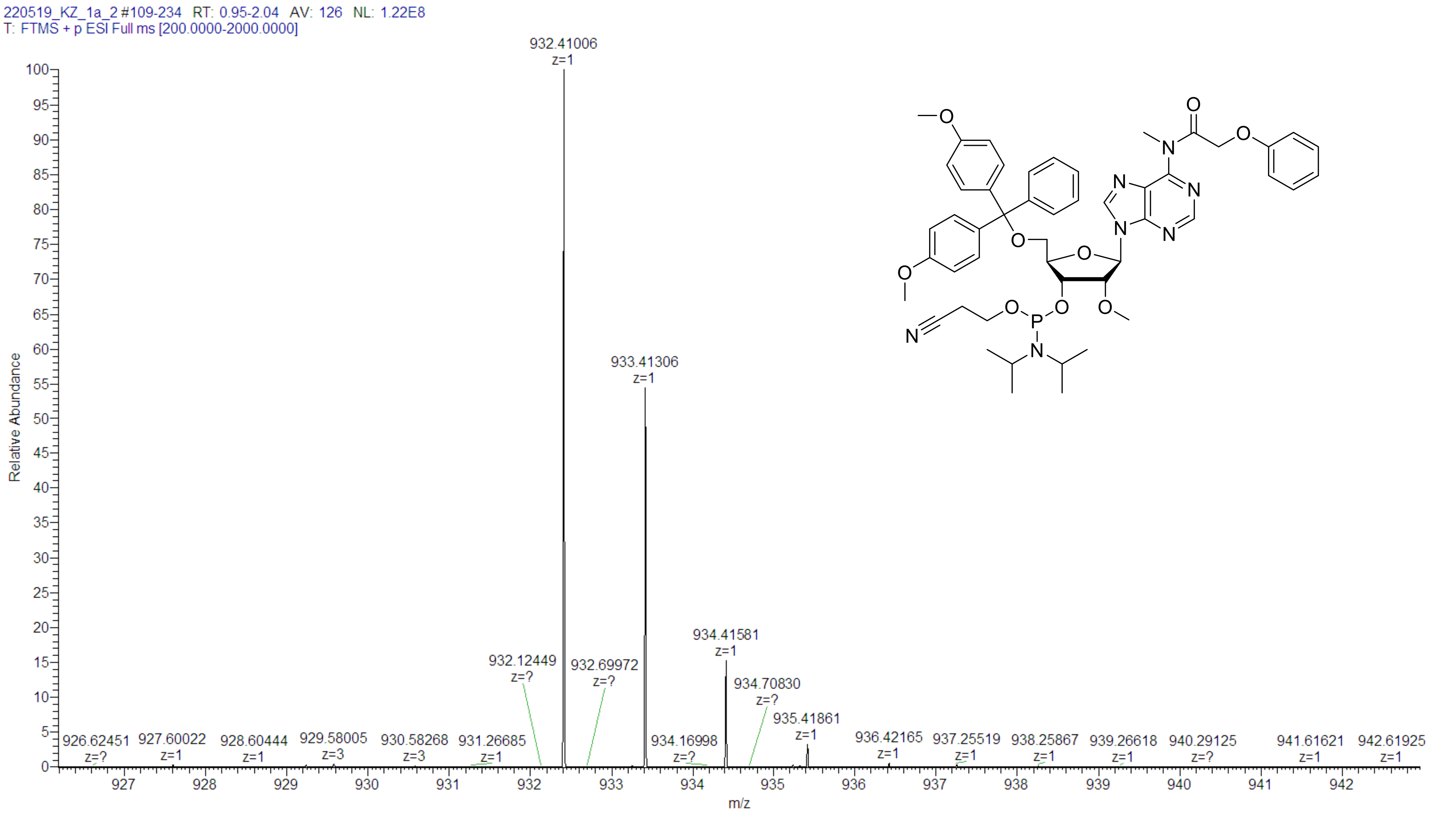

^{1}H NMR (500 MHz, $CDCl_3$, 25°C)

Diastereomer 2

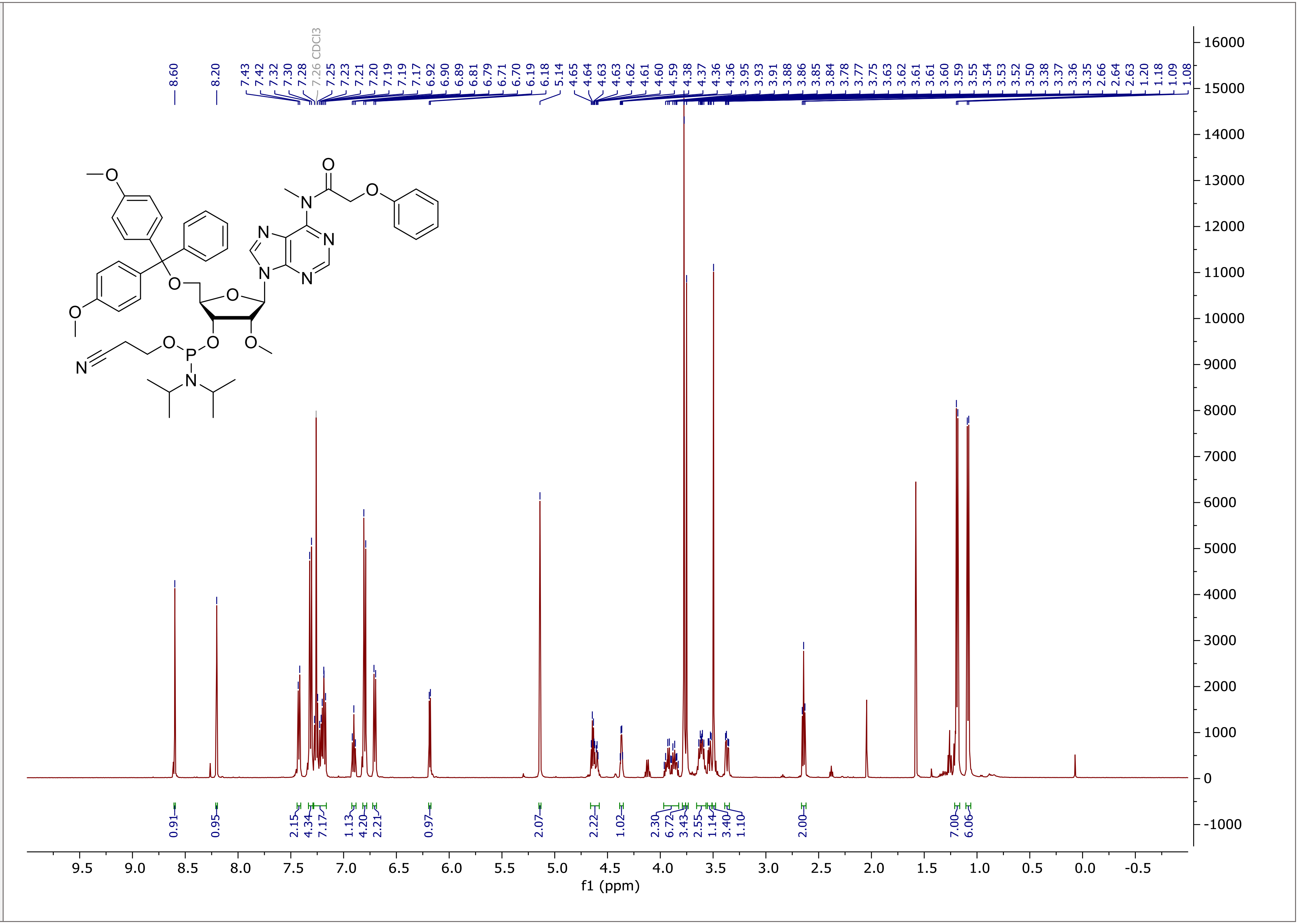

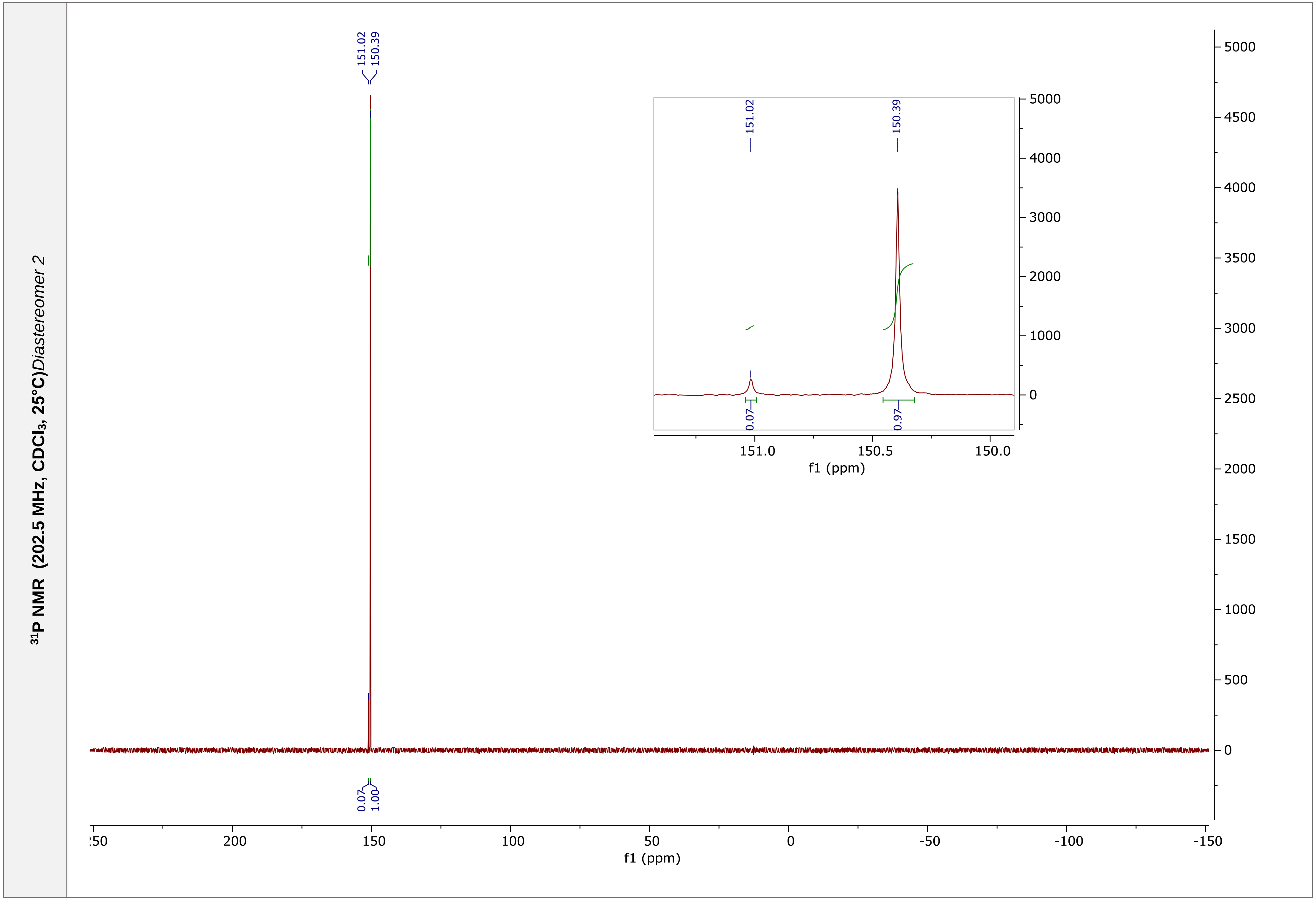

^{31}P NMR (202.5 MHz, CDCl$_3$, 25°C) *Diastereomer 2*
151.02
150.39
0.07
1.00
0.97
f1 (ppm)

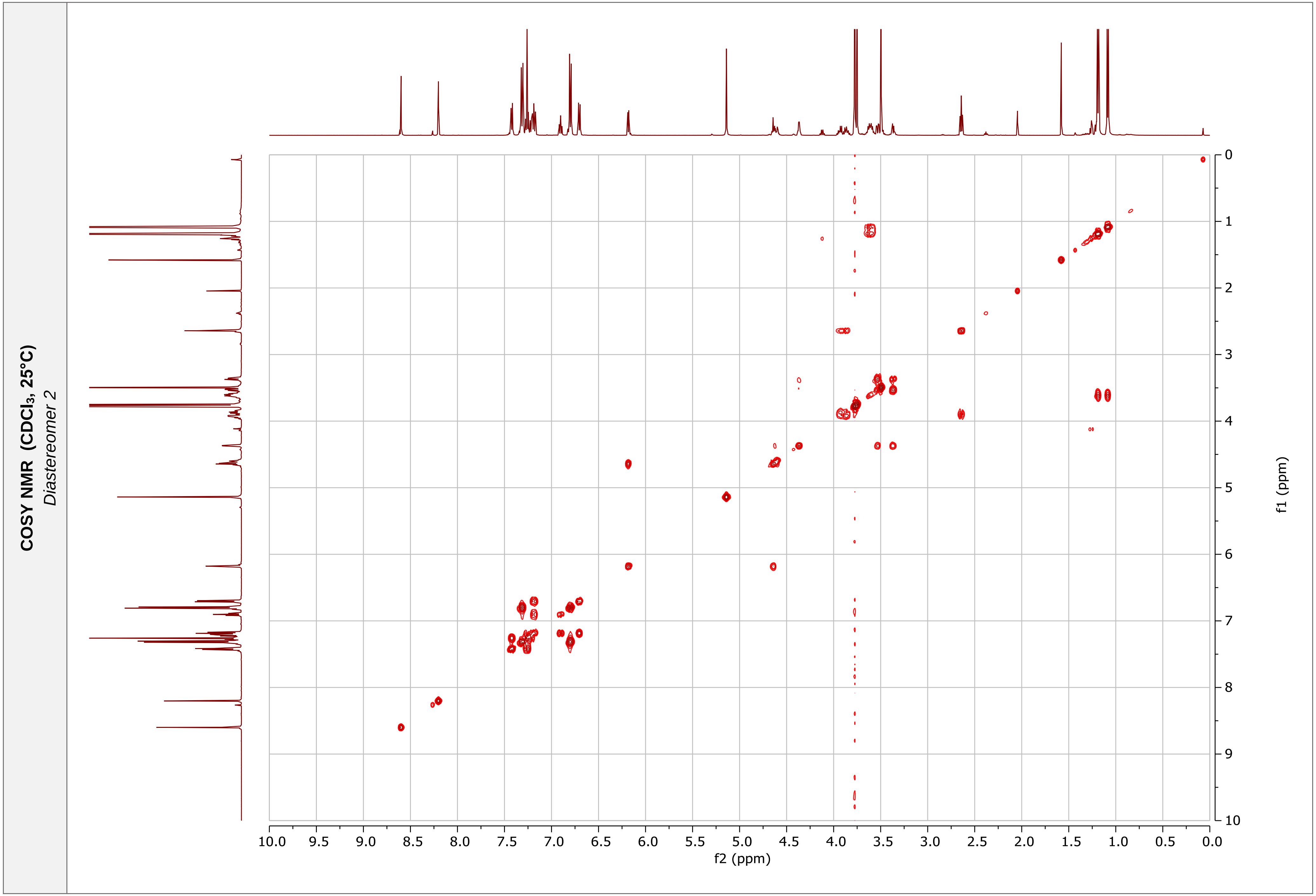

COSY NMR (CDCl$_3$, 25°C)
Diastereomer 2
f2 (ppm)
f1 (ppm)

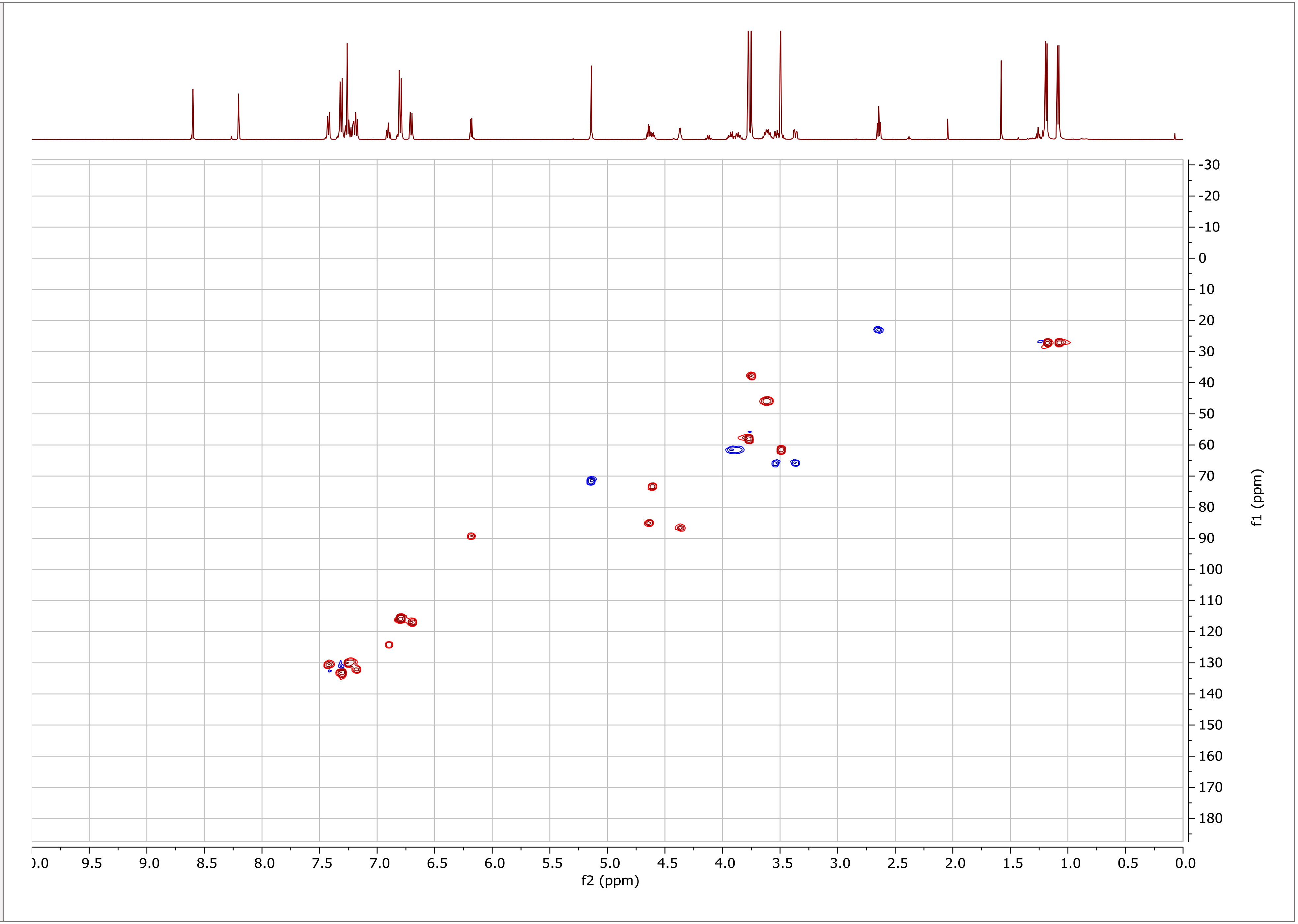

^{1}H-^{13}C HSQC ($CDCl_3$, 25°C)

Diastereomer 2

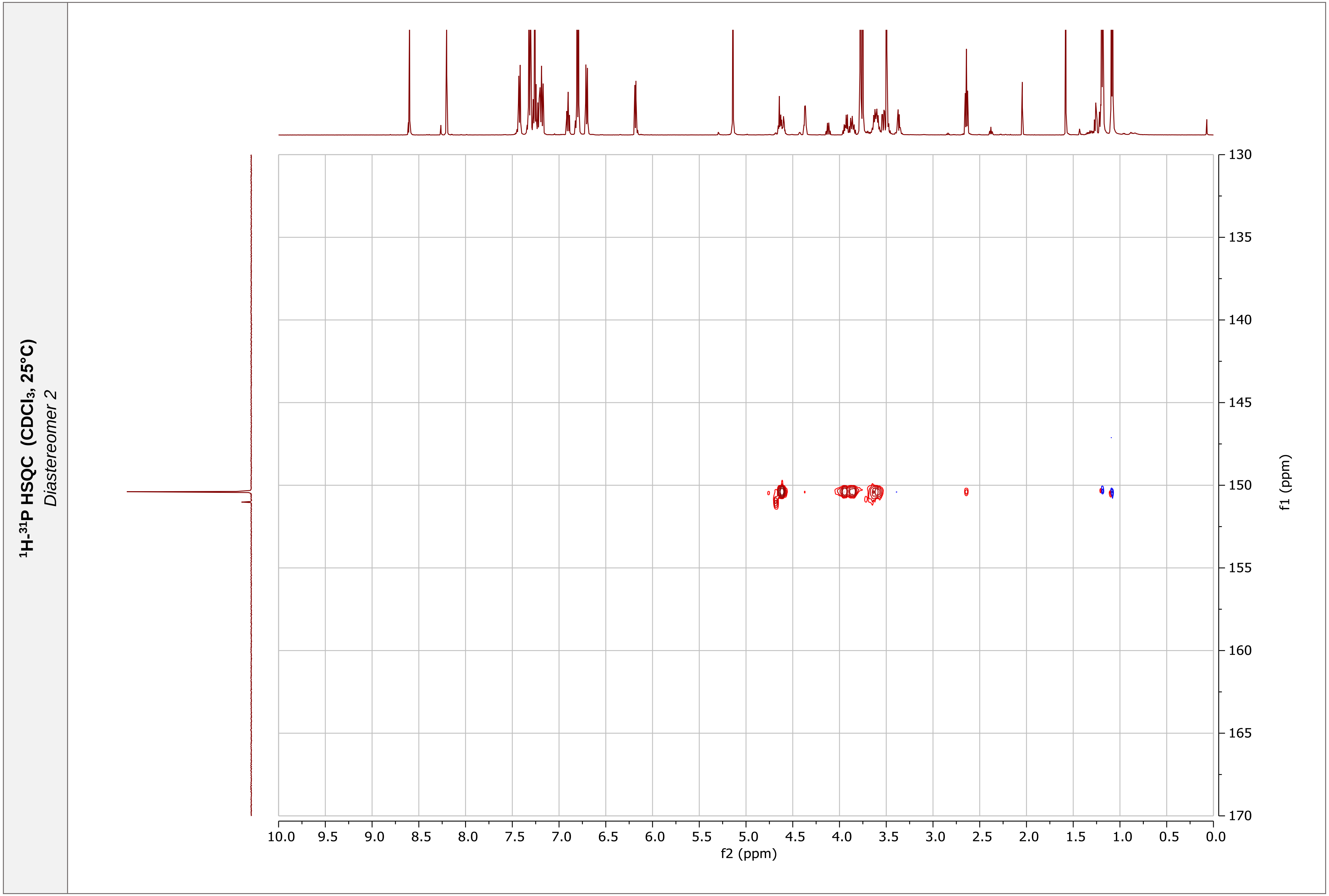
^{1}H-^{31}P HSQC (CDCl$_3$, 25°C)
Diastereomer 2
f2 (ppm)
f1 (ppm)

(1b) *N*6-Isopentenyladenosine phosphoramidite (5′-*O*-DMT-2′-*O*-TBDMS-i^6A^{Ac})

MS (+) ESI

(Calc. $[M+H]^+$ $C_{53}H_{73}N_7O_8PSi^+$ 994.50220)

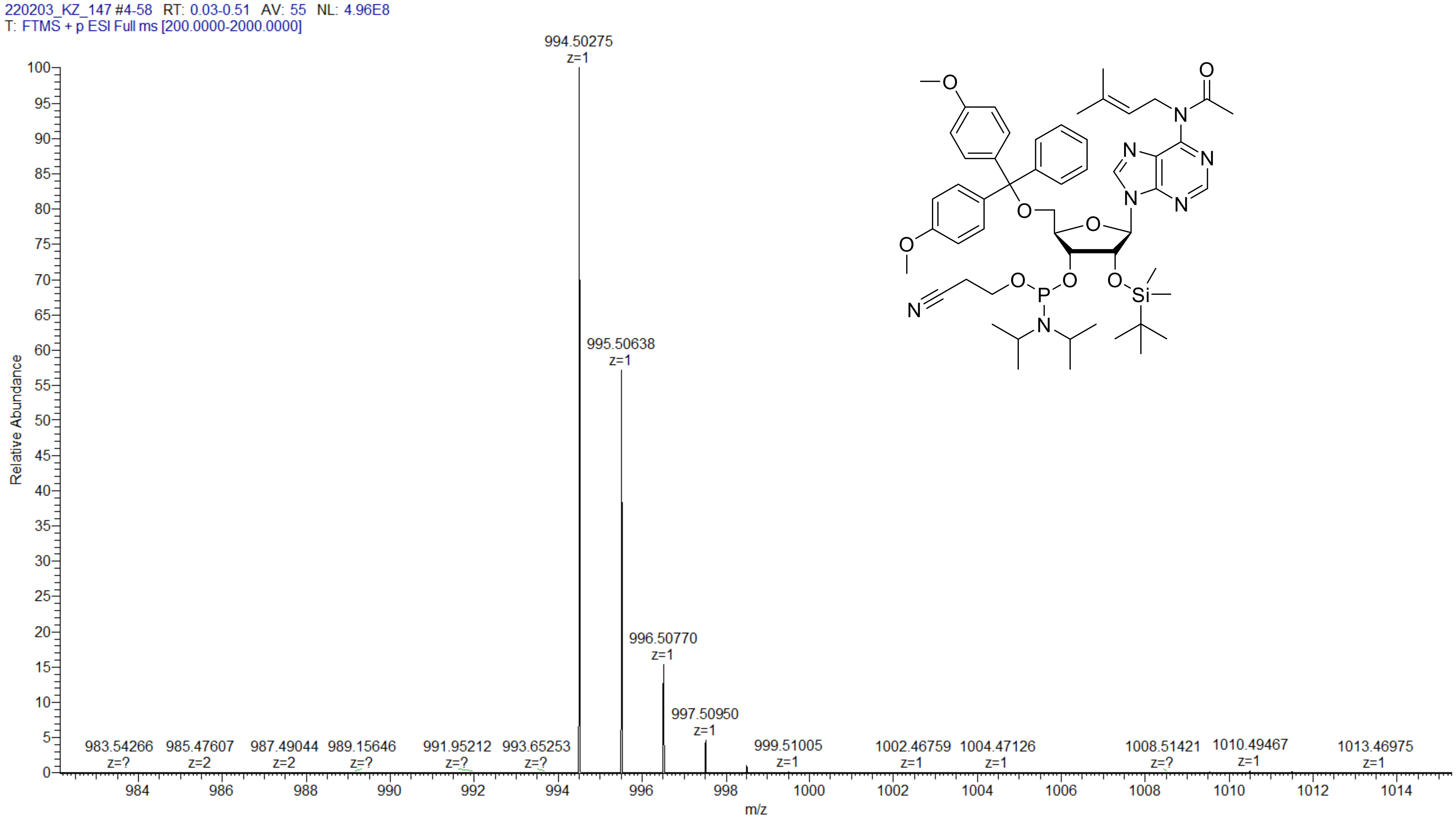

^{1}H NMR (500 MHz, $CDCl_3$, 25°C)

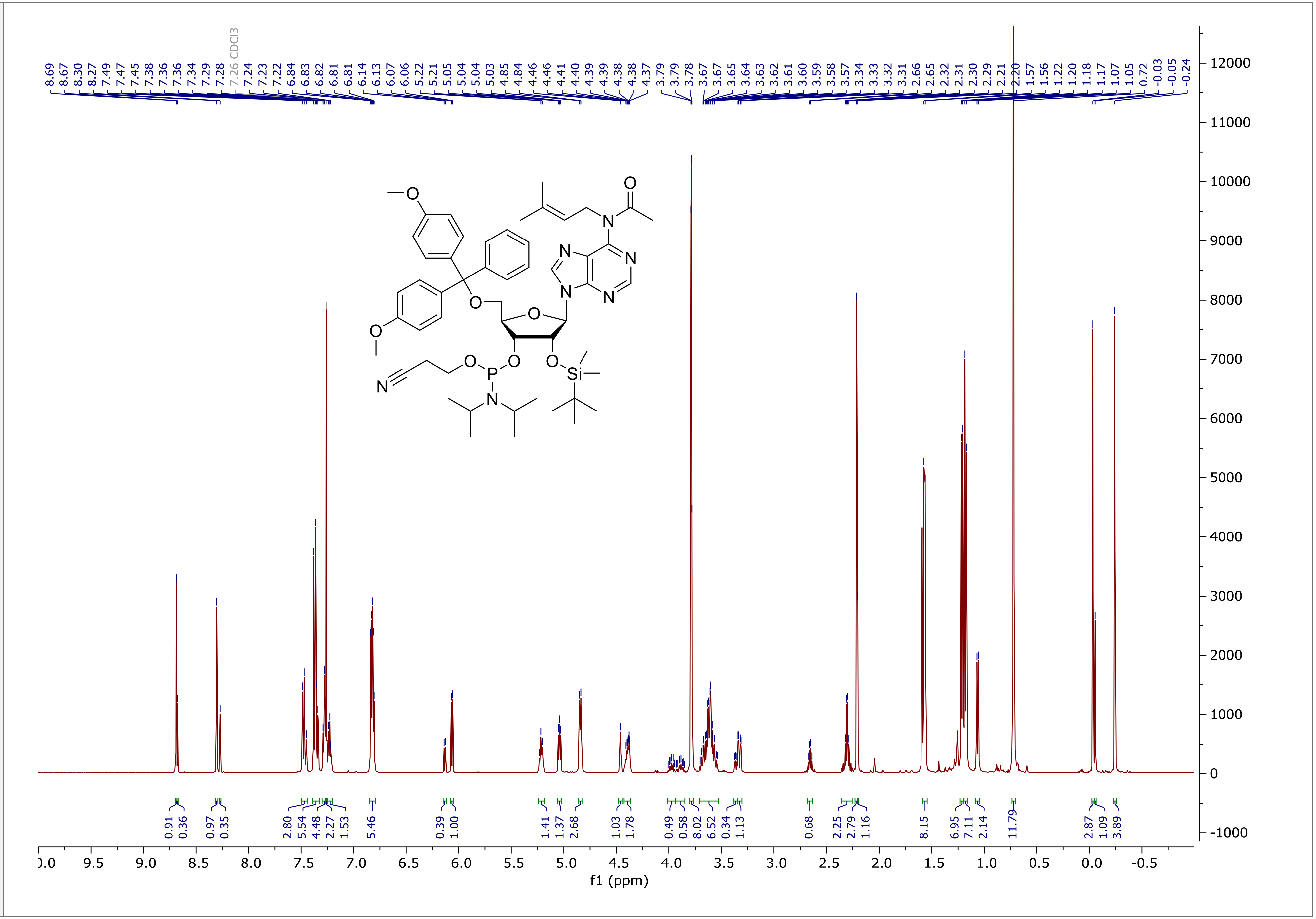

$^{13}C\{^{1}H\}$ NMR (126 MHz, $CDCl_3$, 25°C)

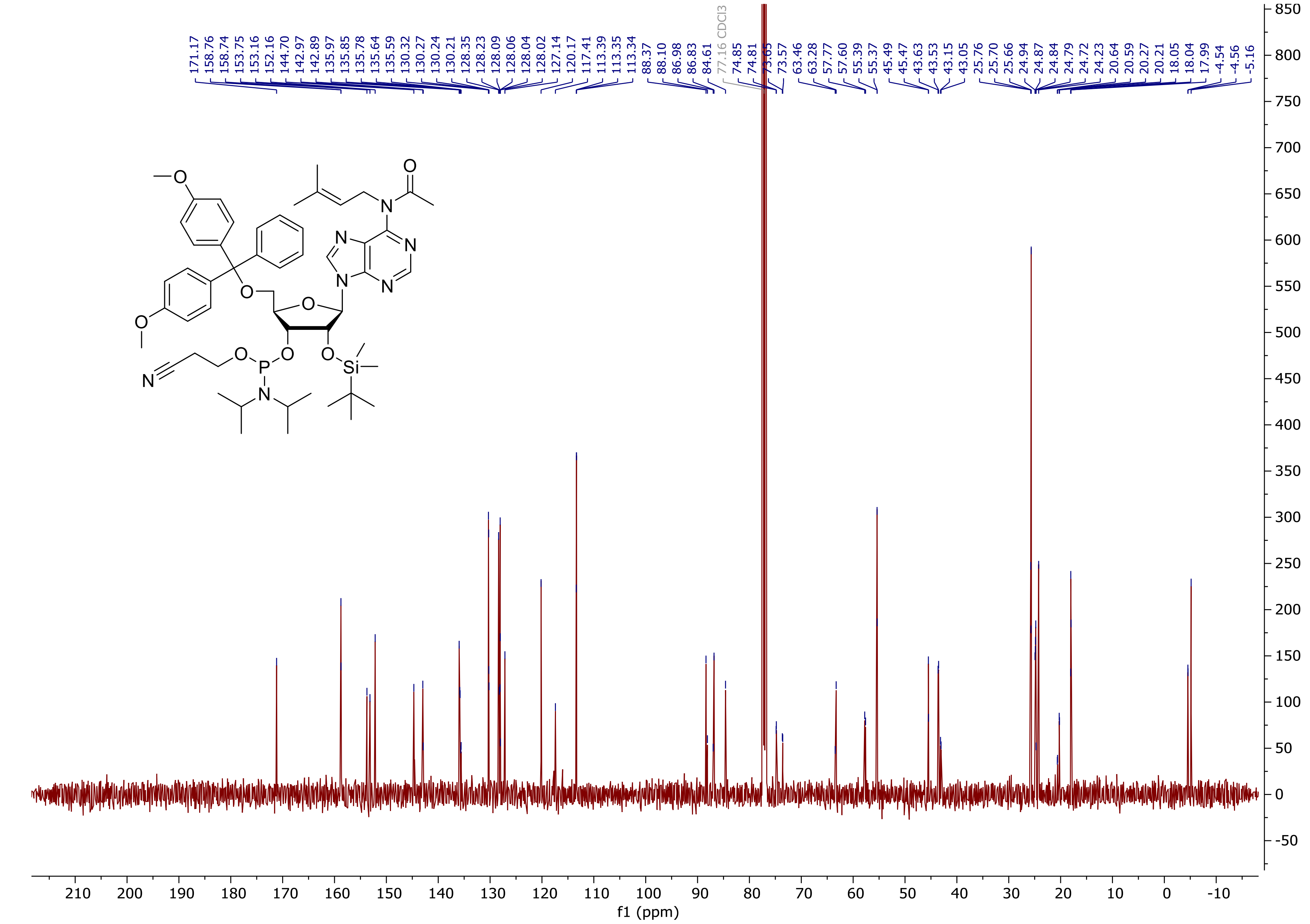

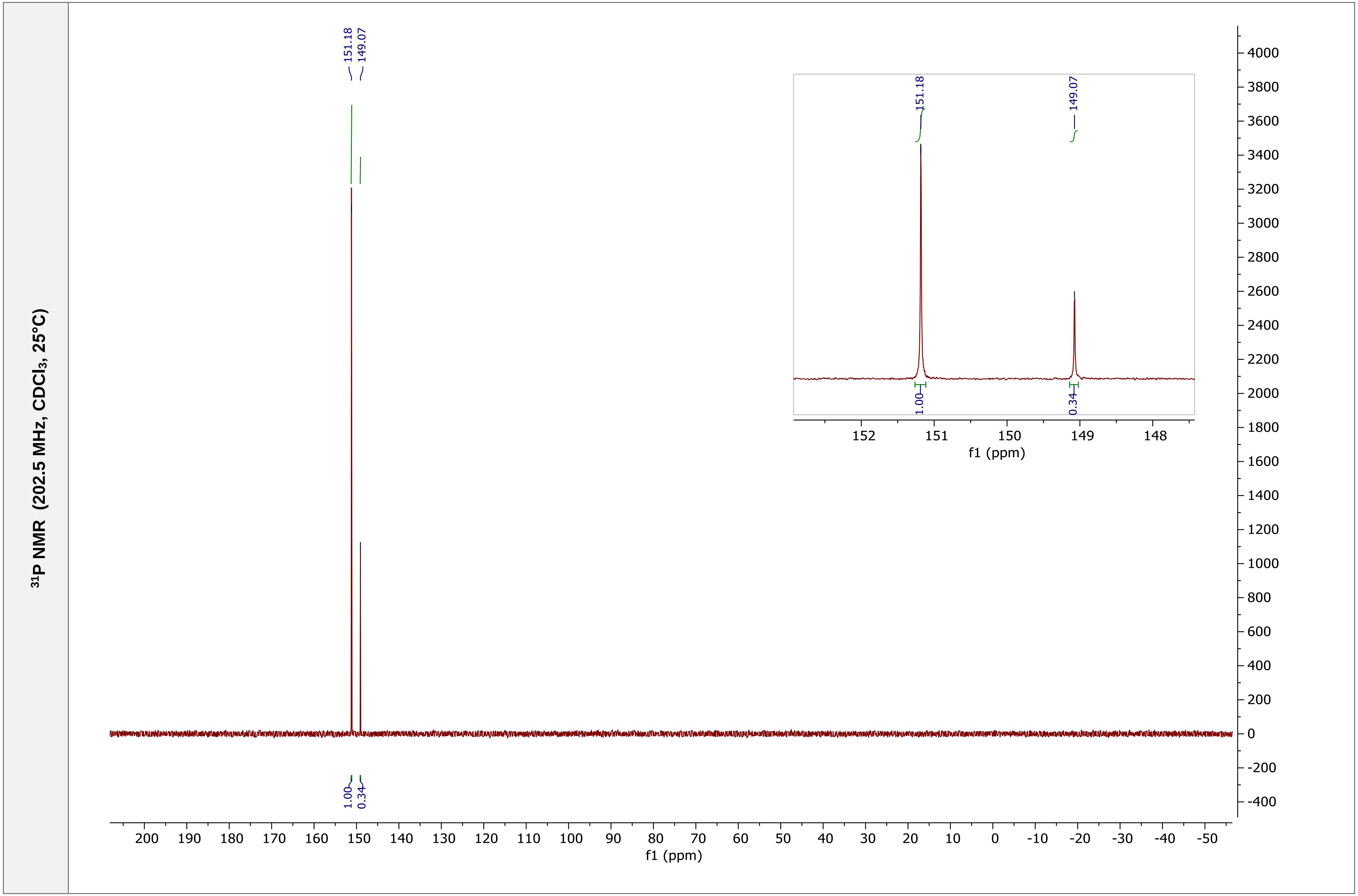
31P NMR (202.5 MHz, CDCl3, 25°C)
151.18
149.07
1.00
0.34
f1 (ppm)

COSY NMR (CDCl$_3$, 25°C)

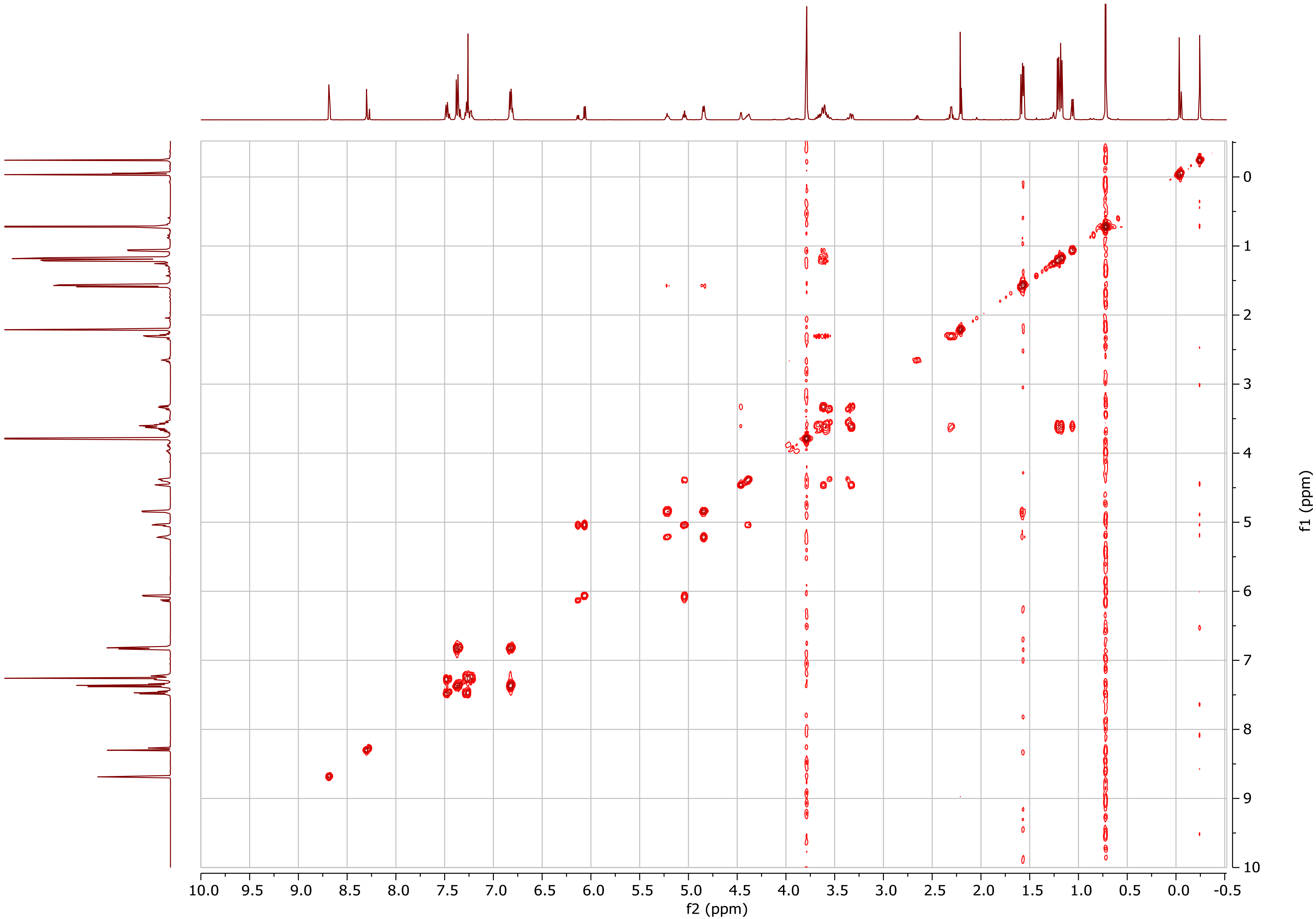

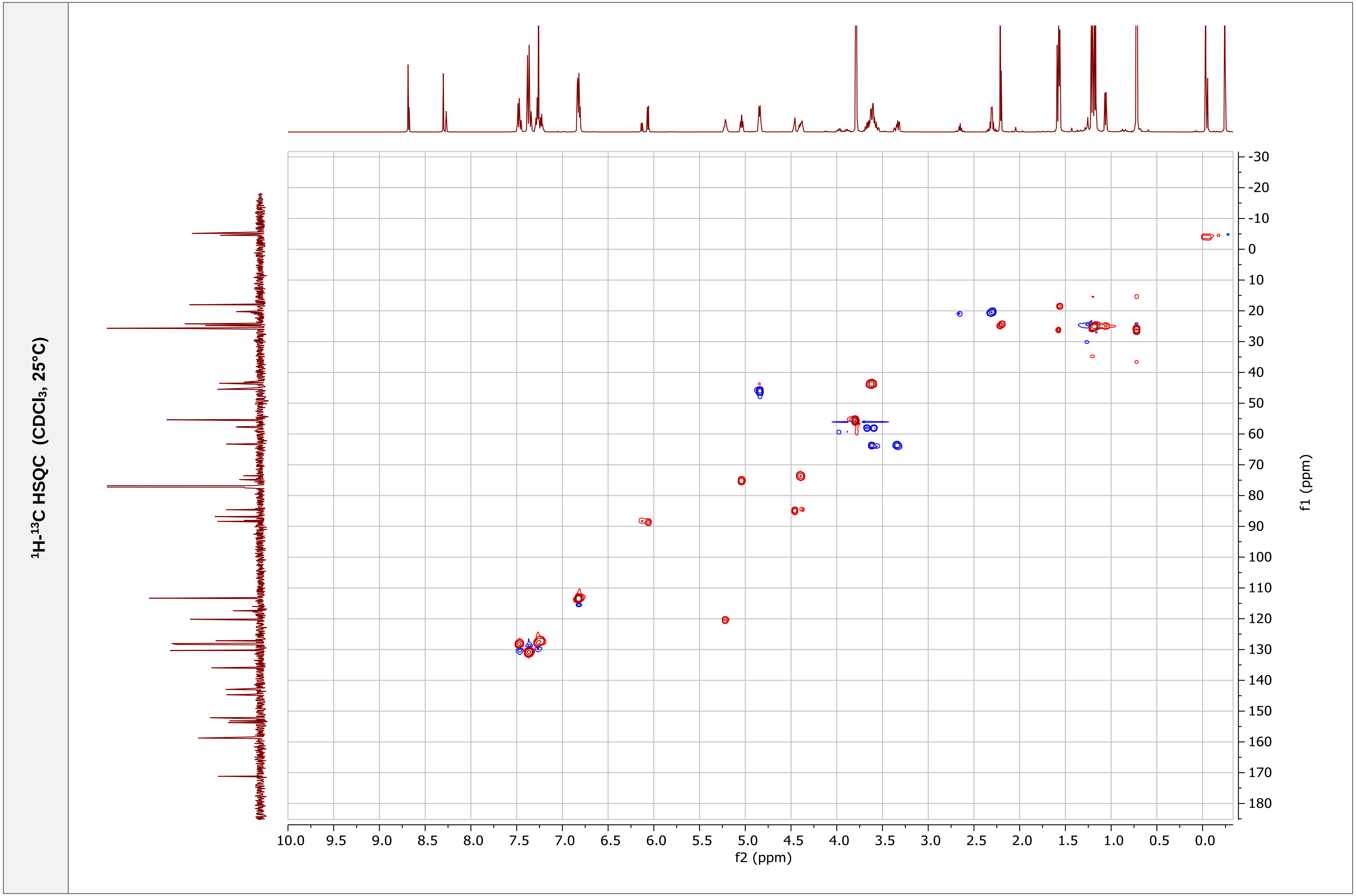
^1H-^{13}C HSQC (CDCl$_3$, 25°C)
f2 (ppm)
f1 (ppm)
10.0 9.5 9.0 8.5 8.0 7.5 7.0 6.5 6.0 5.5 5.0 4.5 4.0 3.5 3.0 2.5 2.0 1.5 1.0 0.5 0.0
-30 -20 -10 0 10 20 30 40 50 60 70 80 90 100 110 120 130 140 150 160 170 180

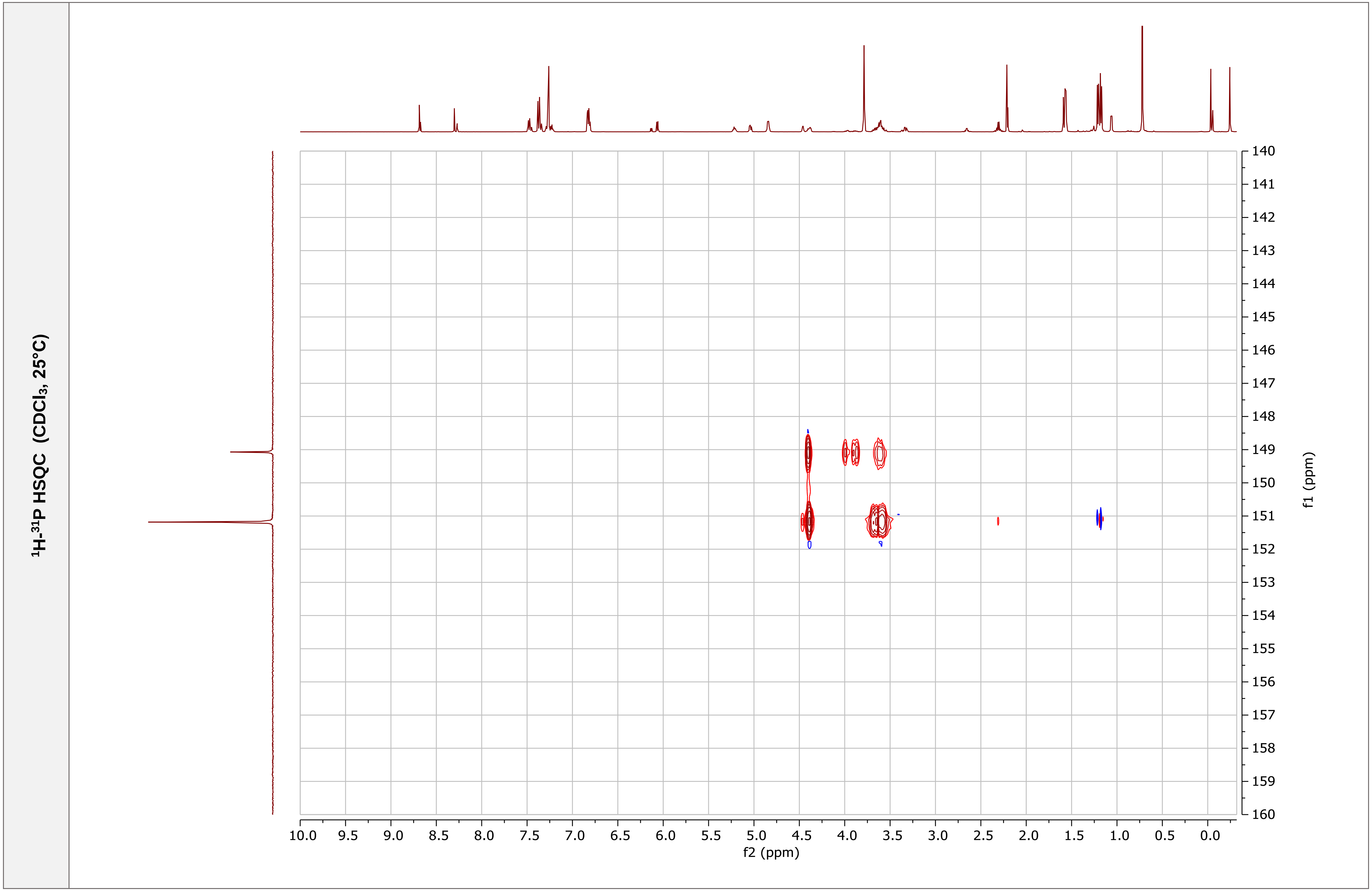
^{1}H-^{31}P HSQC (CDCl$_3$, 25°C)
f2 (ppm)
f1 (ppm)
10.0 9.5 9.0 8.5 8.0 7.5 7.0 6.5 6.0 5.5 5.0 4.5 4.0 3.5 3.0 2.5 2.0 1.5 1.0 0.5 0.0
140 141 142 143 144 145 146 147 148 149 150 151 152 153 154 155 156 157 158 159 160

(1c) *N*6-benzyladenosine phosphoramidite (5′-*O*-DMT-2′-*O*-Me-Bn^{6}A^{Pac})

MS (+) ESI

(Calc. [M+H]$^{+}$ $C_{56}H_{63}N_{7}O_{9}P^{+}$ 1008.44194)

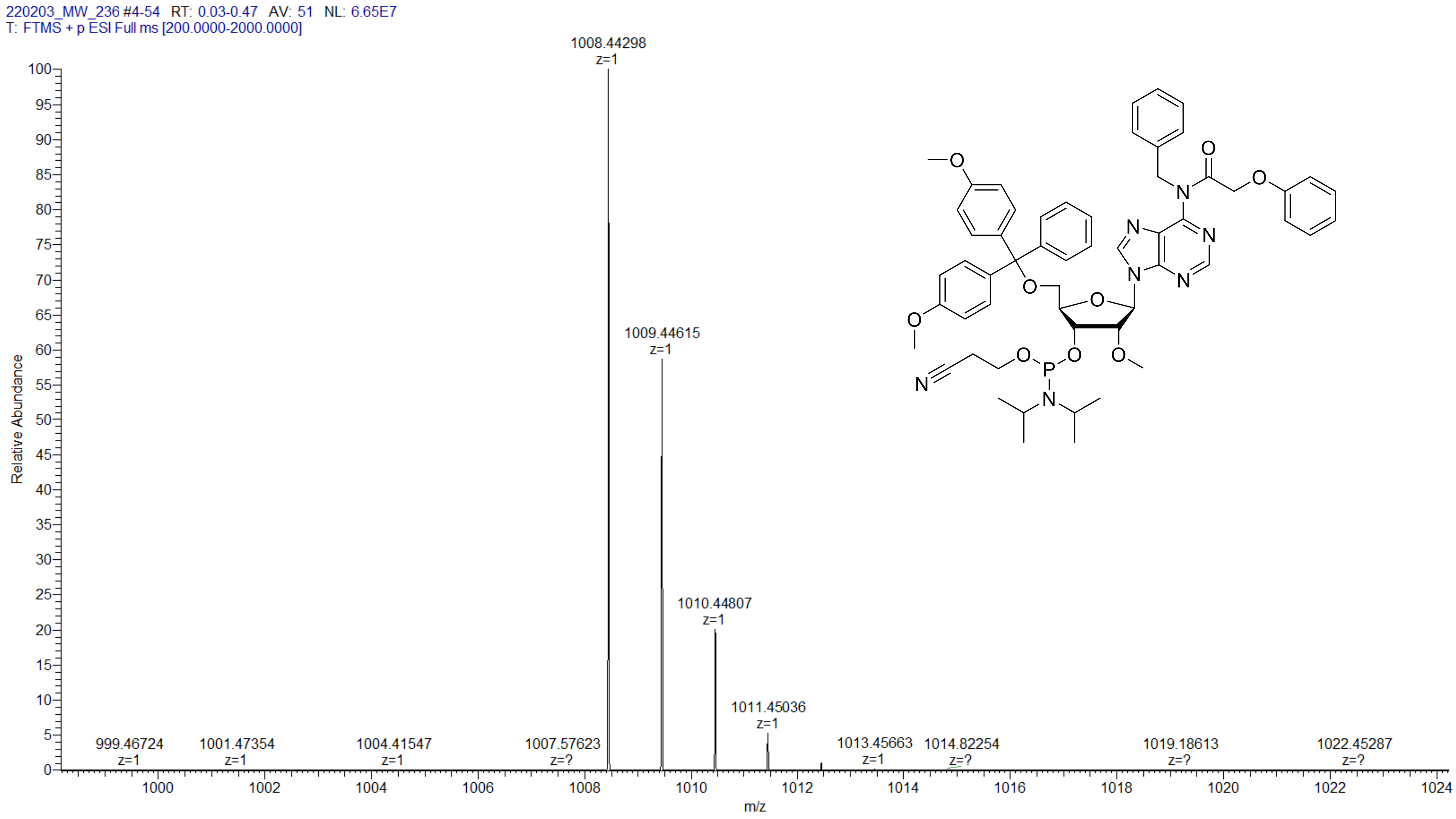

^{1}H NMR (500 MHz, $CDCl_3$, 25°C)

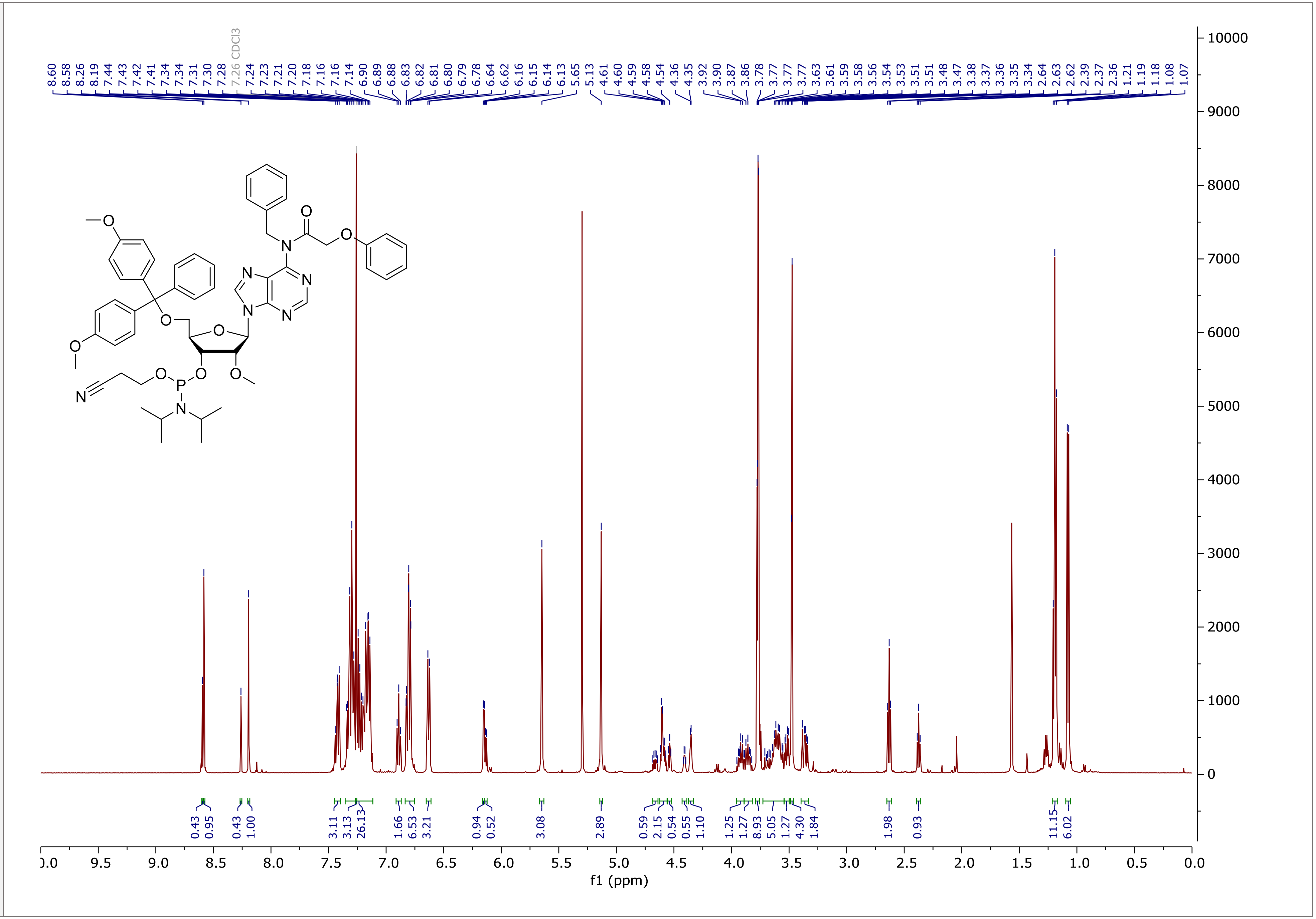

$^{13}C\{^{1}H\}$ NMR (126 MHz, $CDCl_3$, 25°C)

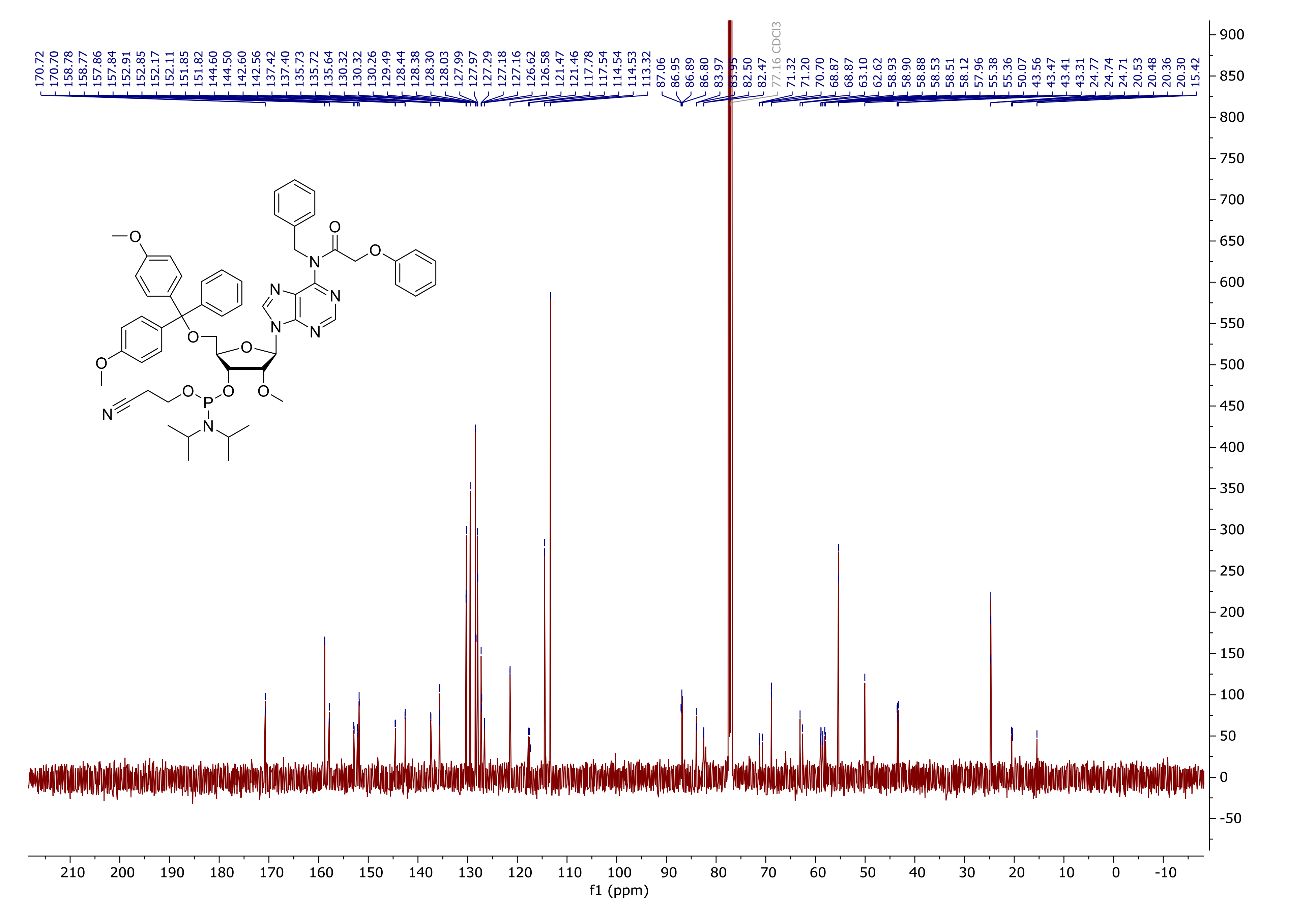

^{31}P NMR (202.5 MHz, $CDCl_3$, 25°C)

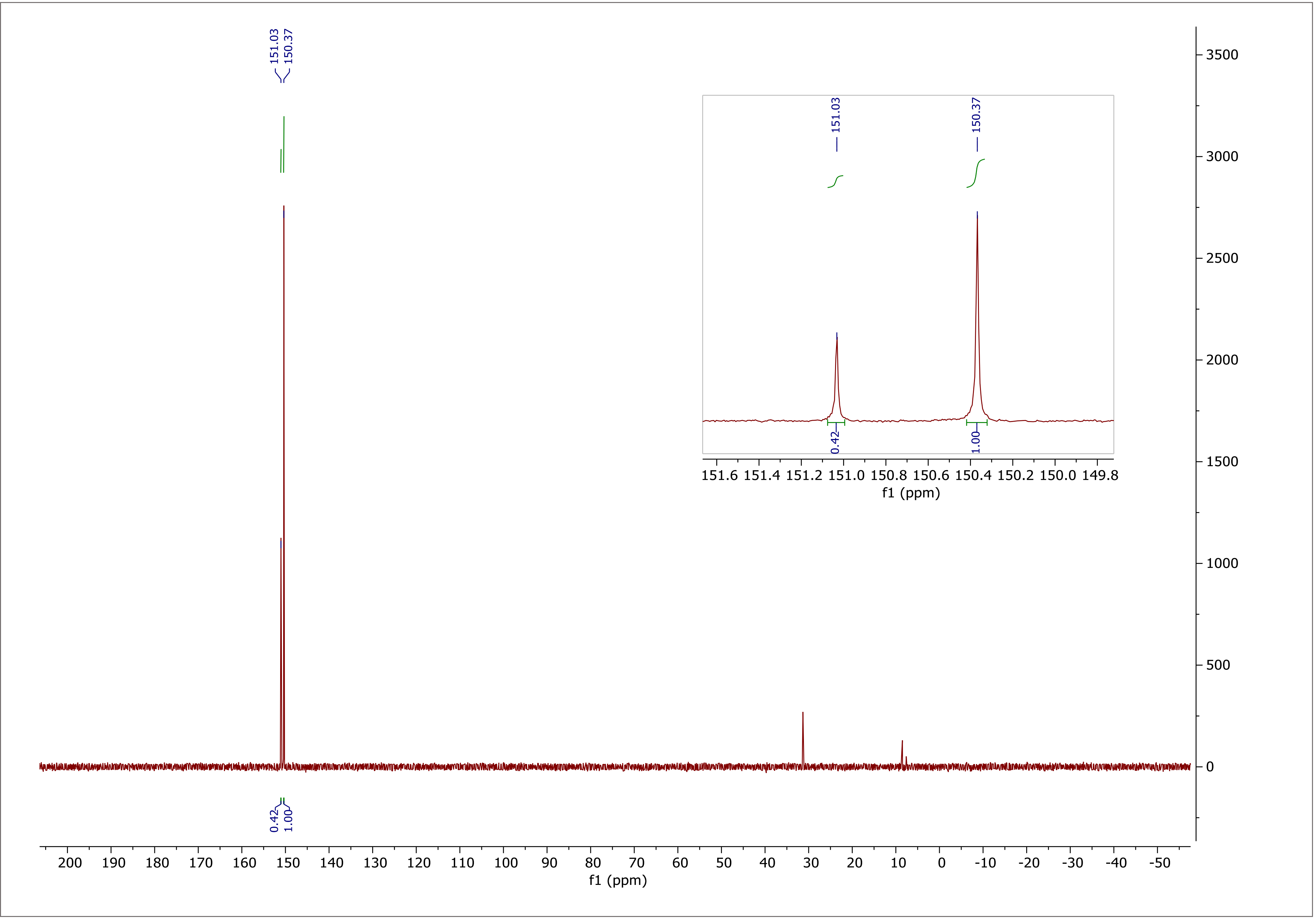

COSY NMR (CDCl$_3$, 25°C)

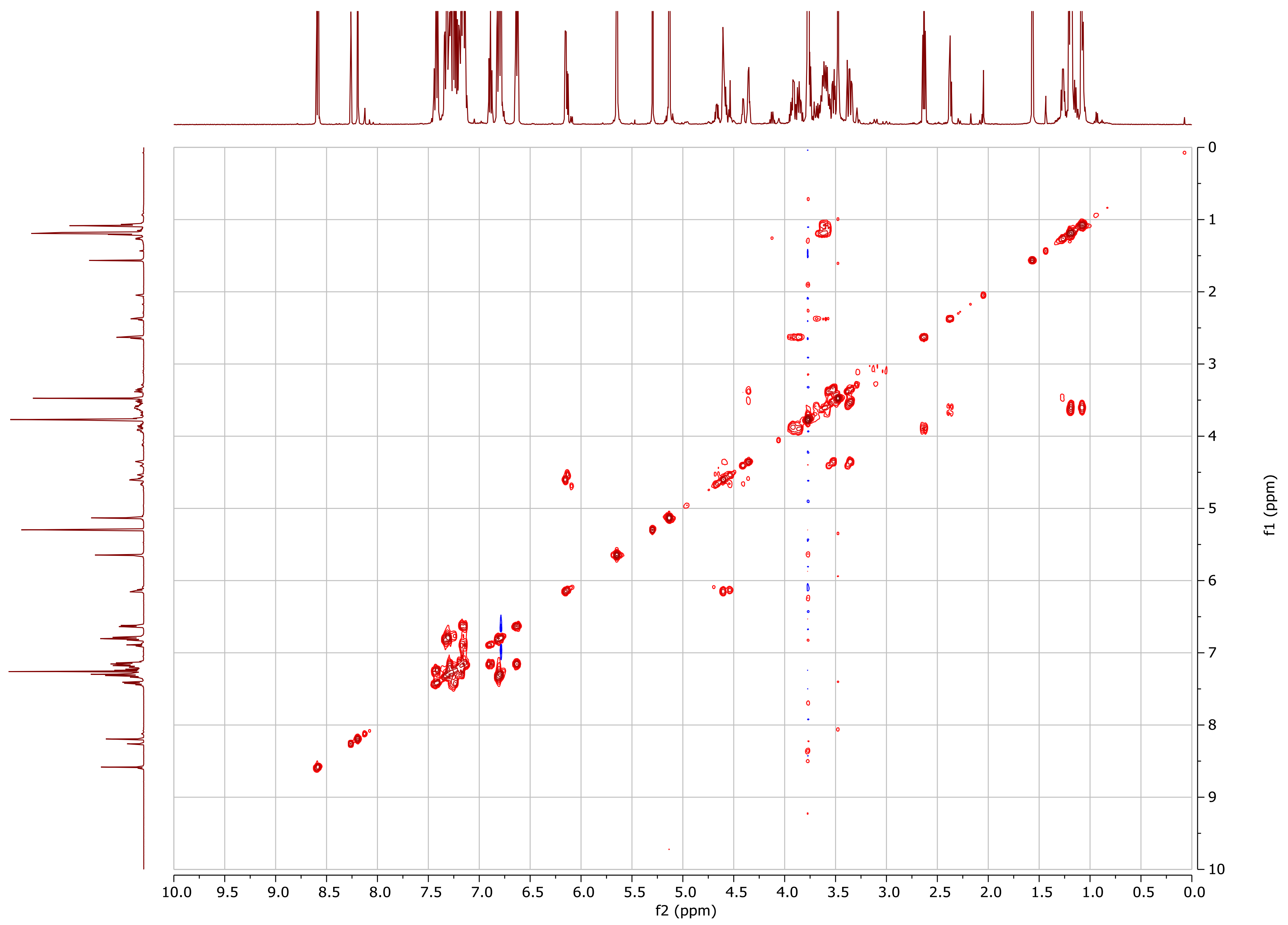

^{1}H-^{13}C HSQC (CDCl$_3$, 25°C)

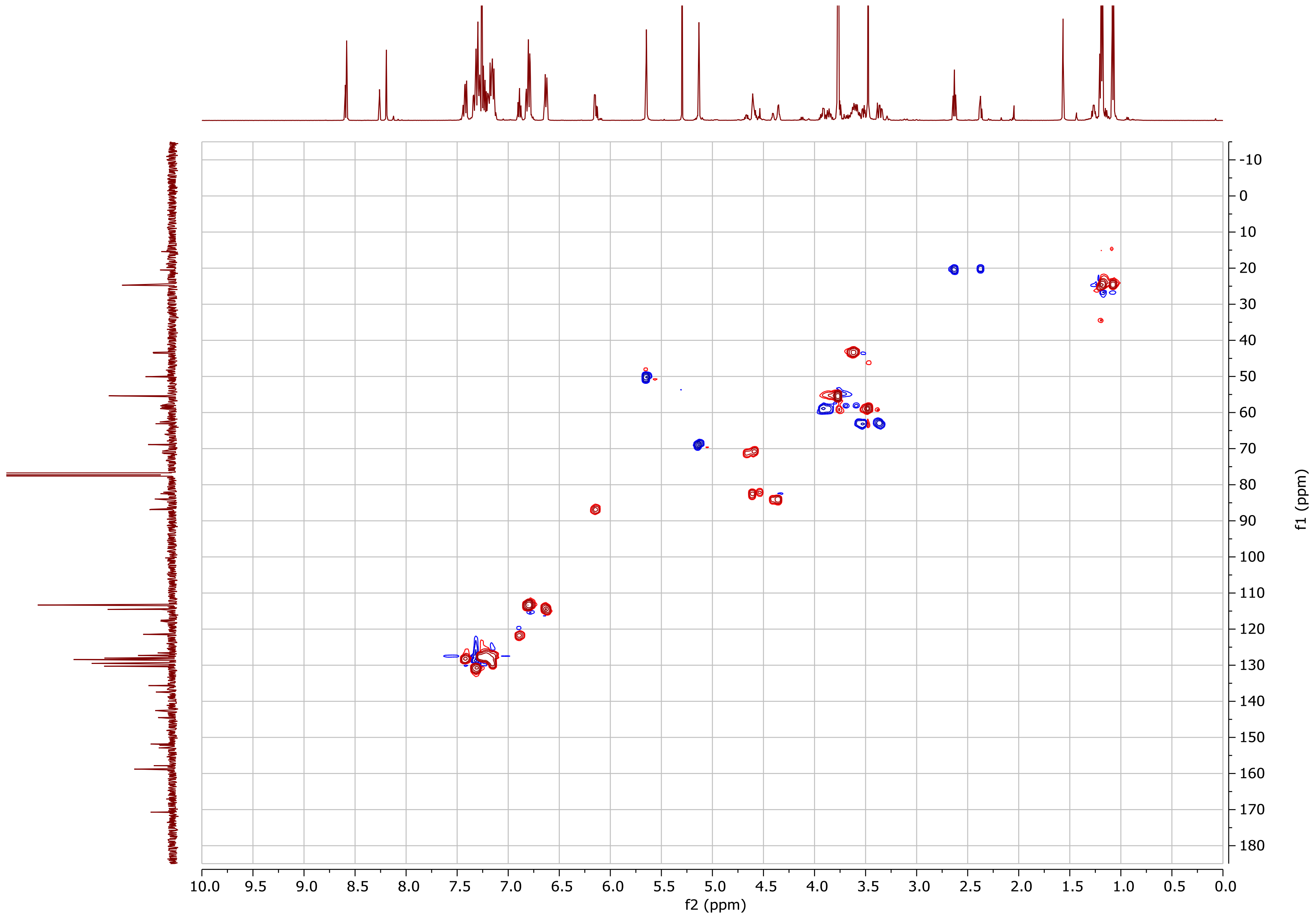

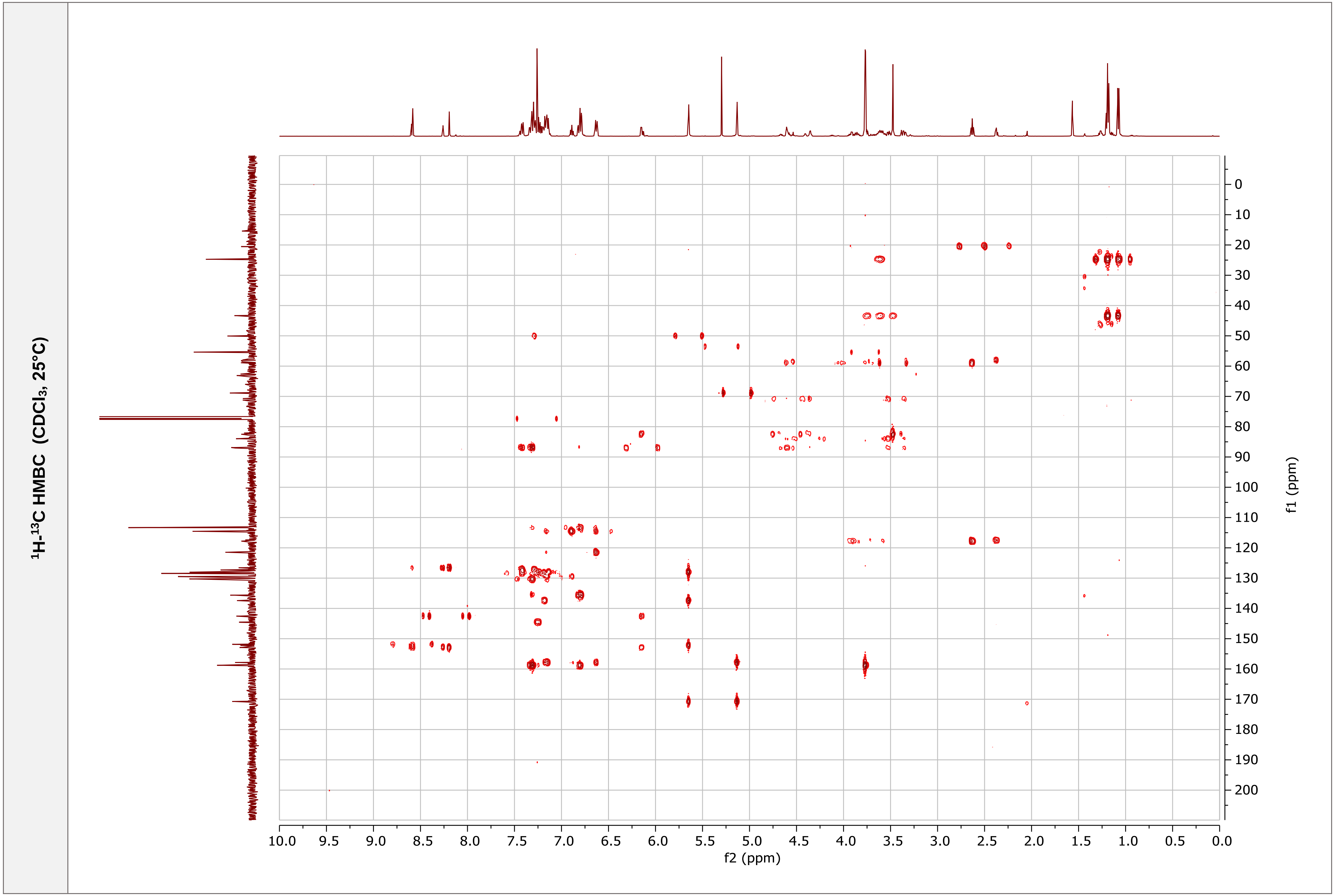

^{1}H-^{13}C HMBC (CDCl$_3$, 25°C)
f2 (ppm)
f1 (ppm)

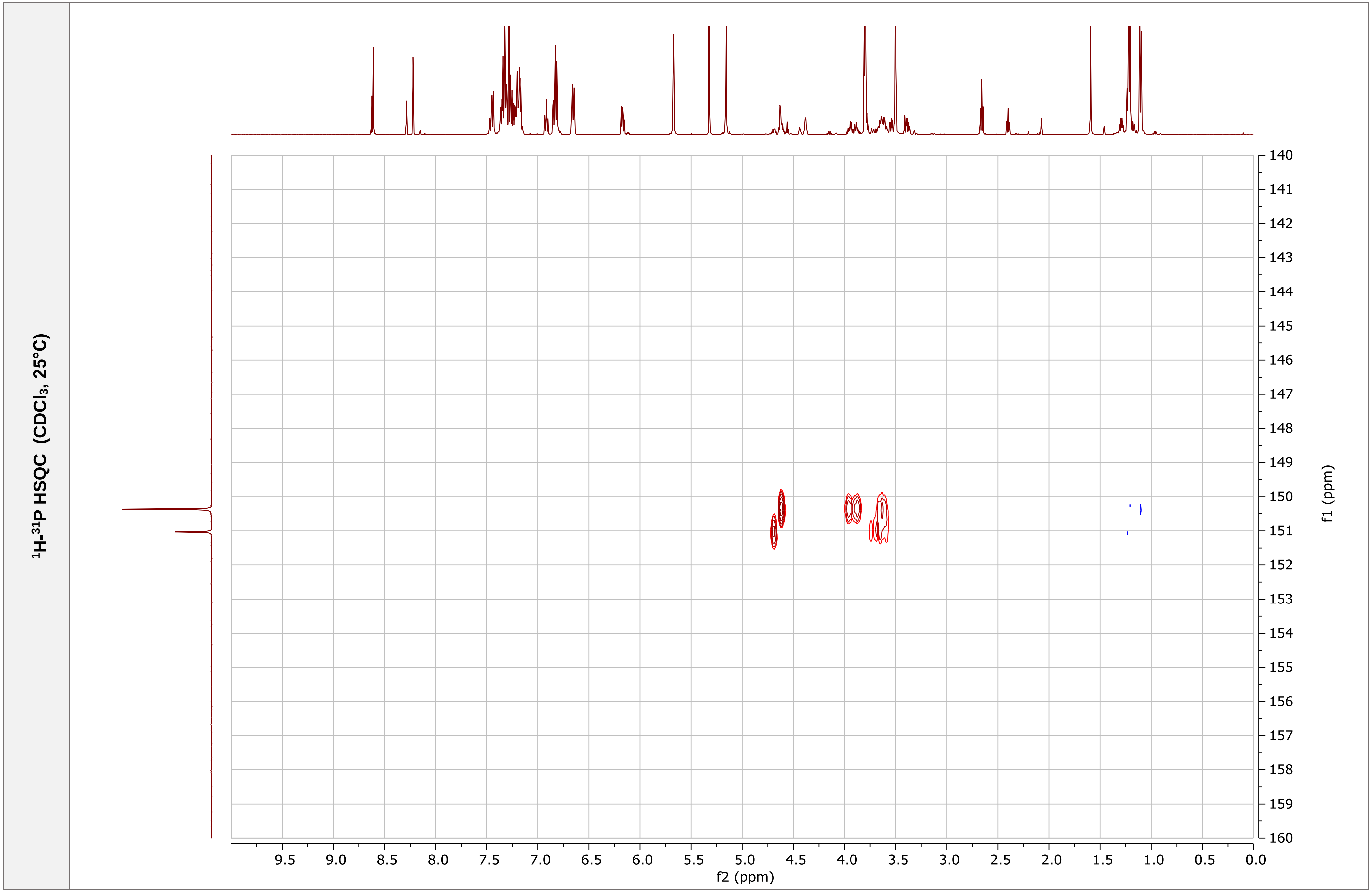

^{1}H-^{31}P HSQC (CDCl$_3$, 25°C)
f2 (ppm)
f1 (ppm)

(1d) *N*6-hexynyladenosine phosphoramidite (5′-*O*-DMT-2′-*O*-Me-hex^{6}A^{Pac})

MS (+) ESI
(Calc. $[M+H]^{+}$ $C_{55}H_{65}N_{7}O_{9}P^{+}$ 998.45759)

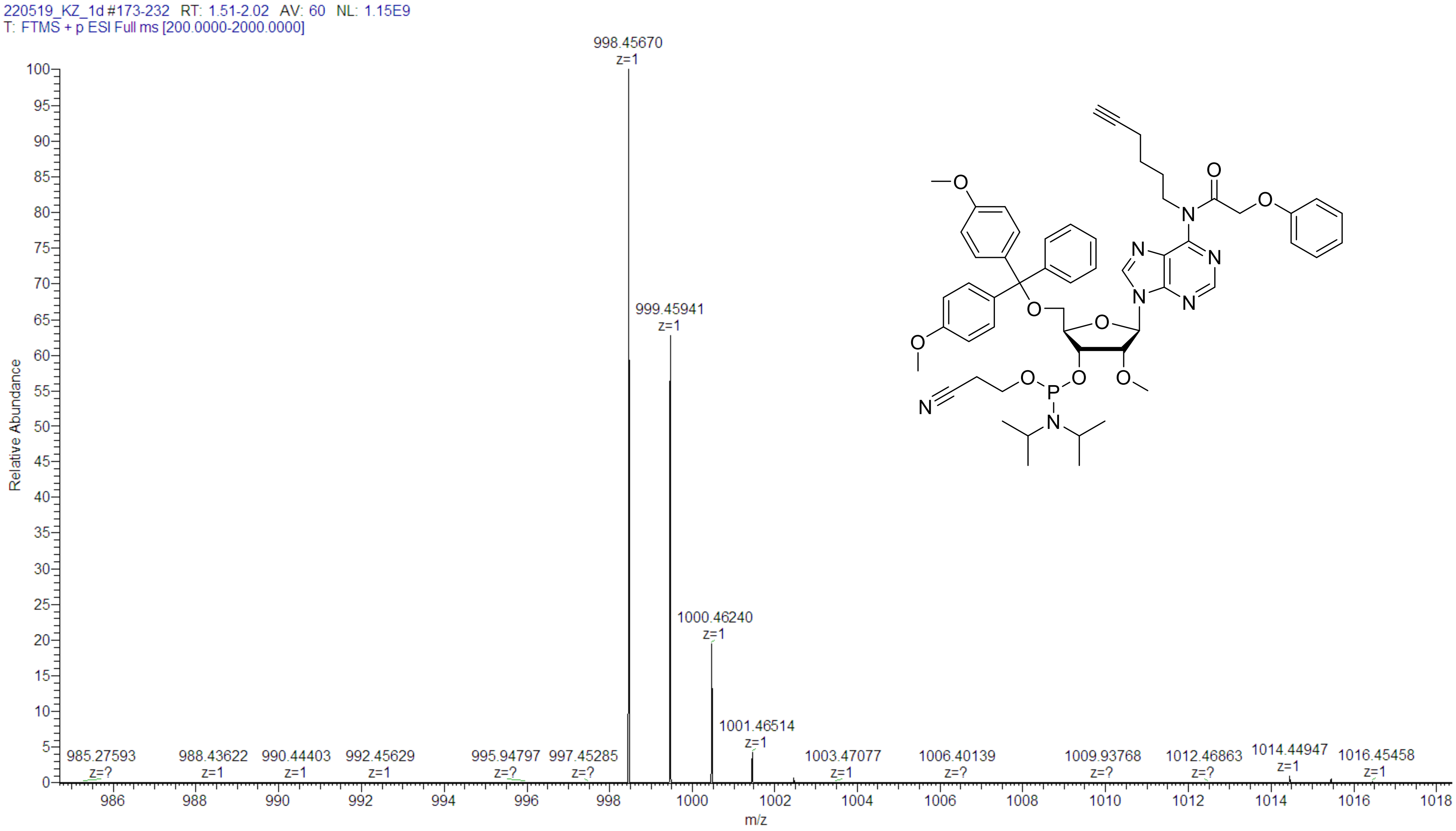

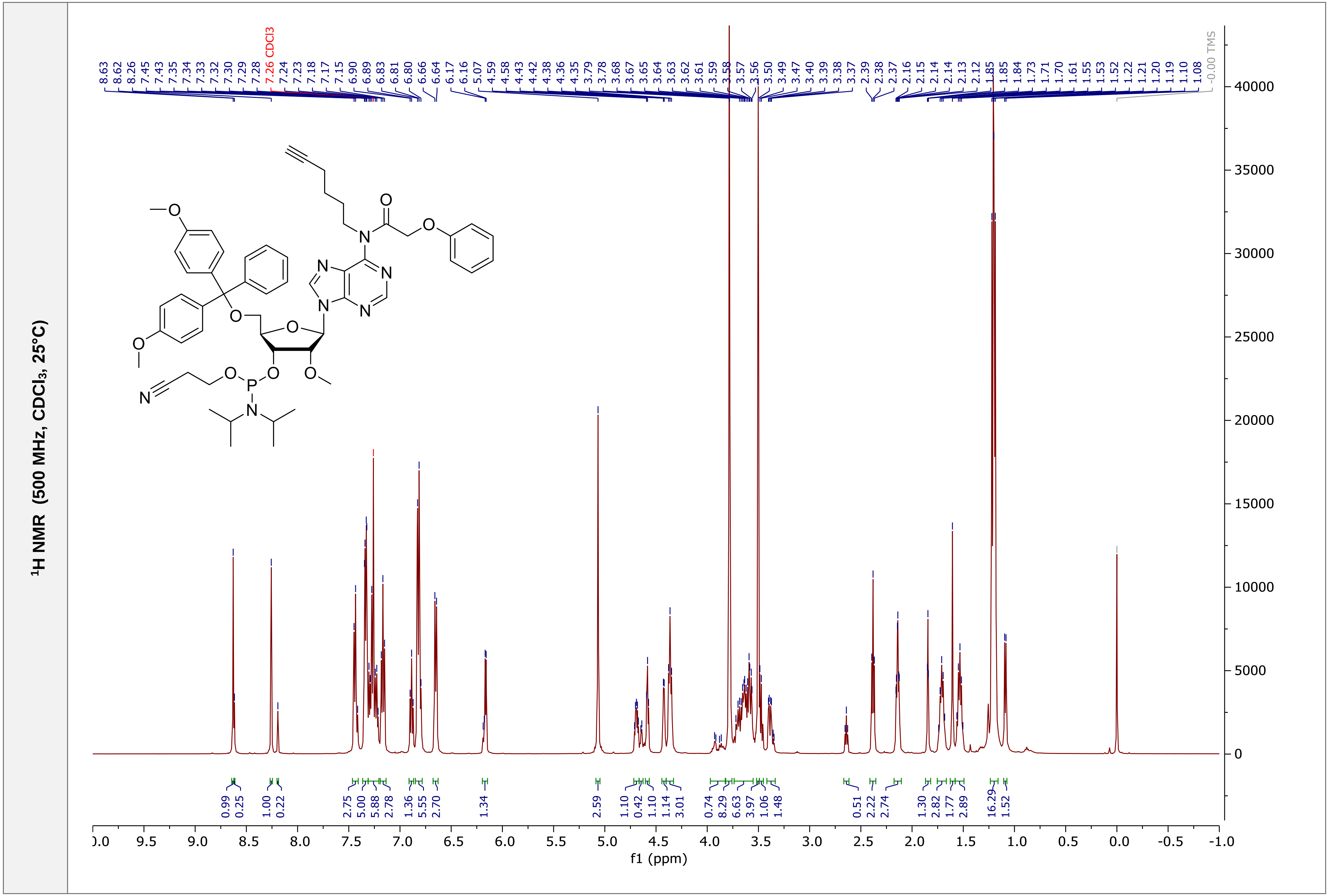

^1H NMR (500 MHz, $CDCl_3$, 25°C)
7.26 CDCl3
-0.00 TMS
f1 (ppm)

$^{13}C\{^{1}H\}$ NMR (126 MHz, $CDCl_3$, 25°C)

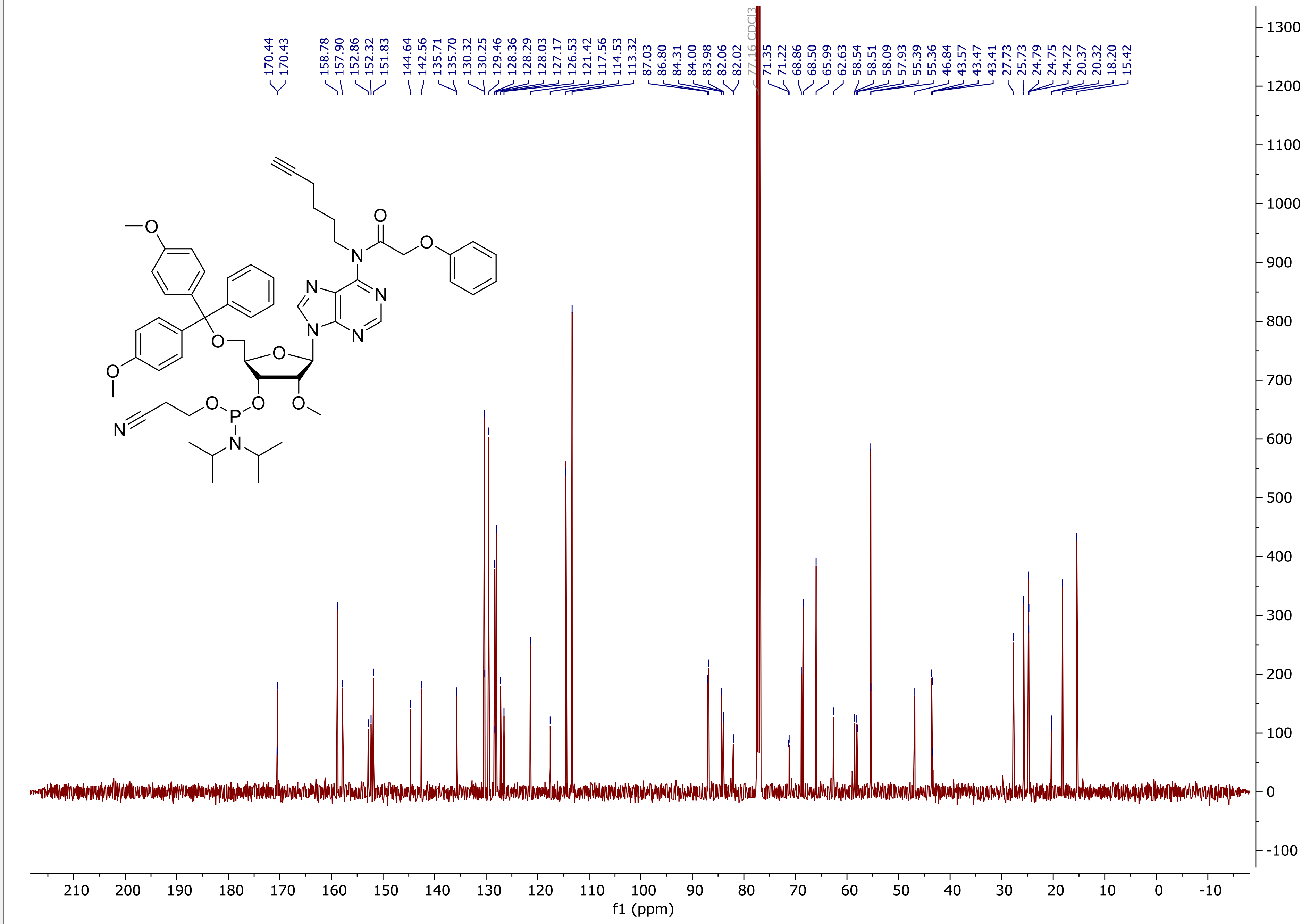

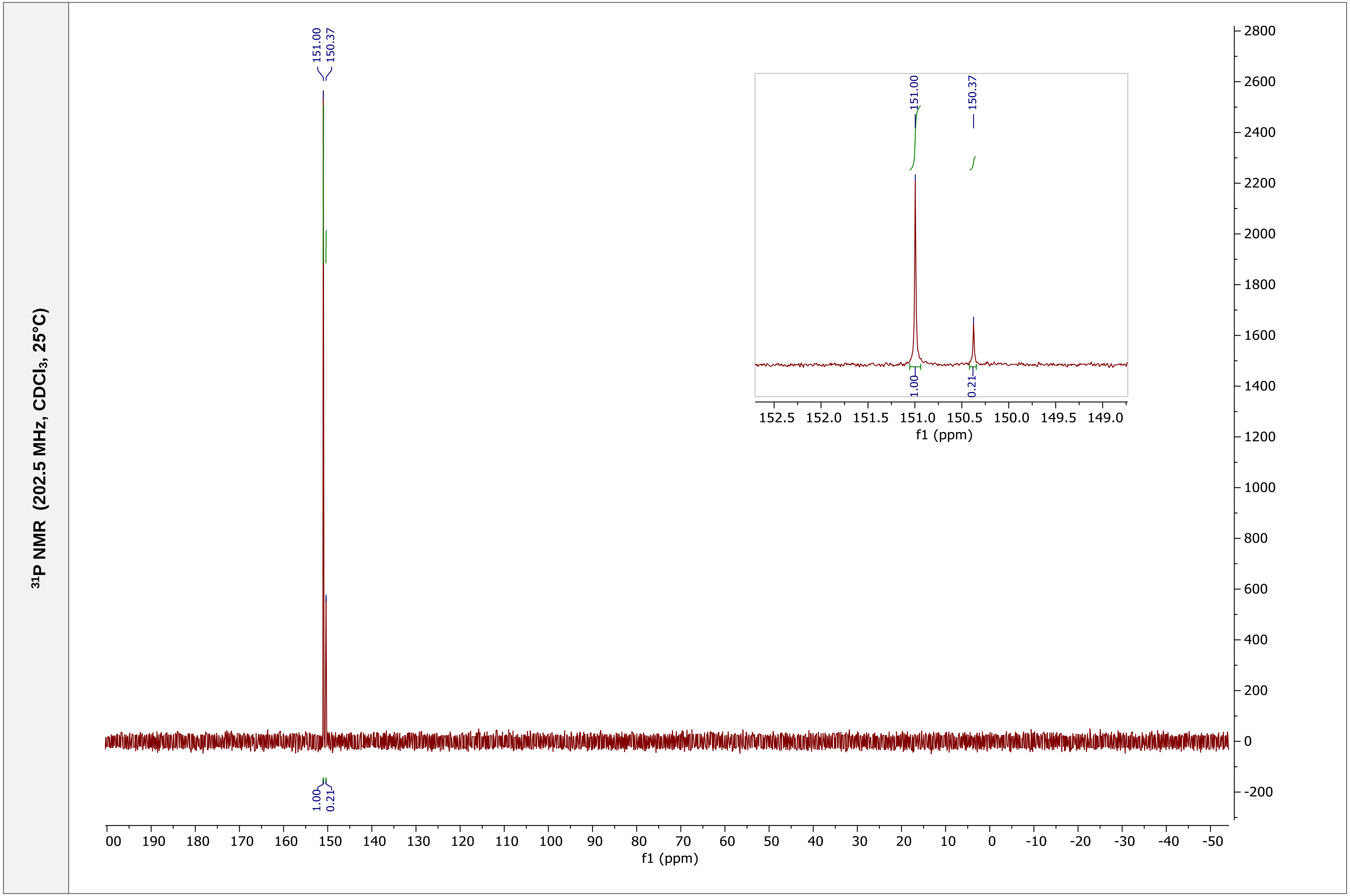

^{31}P NMR (202.5 MHz, CDCl$_3$, 25°C)
151.00
150.37
1.00
0.21
f1 (ppm)

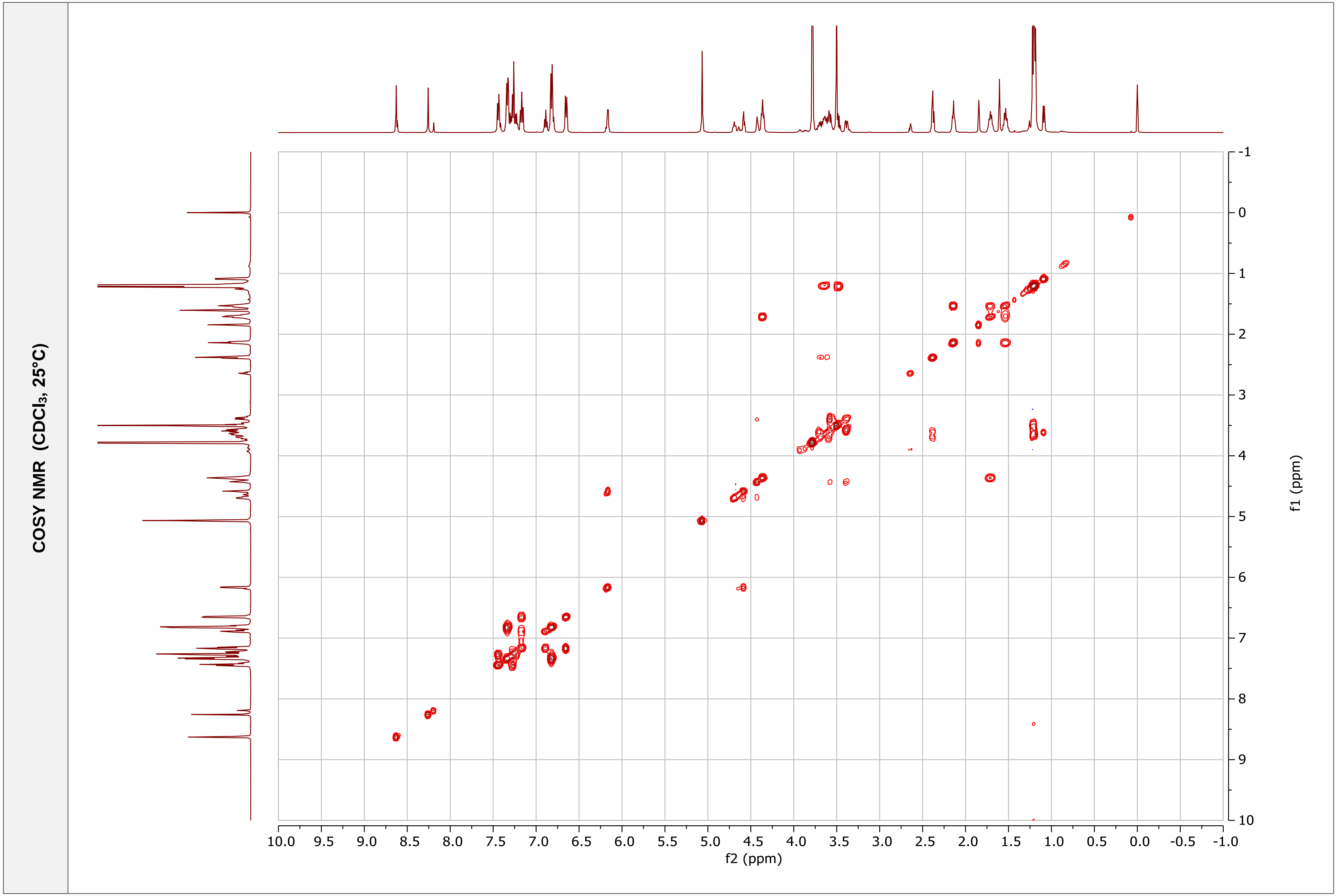
COSY NMR (CDCl$_3$, 25°C)
f2 (ppm)
f1 (ppm)

^{1}H-^{13}C HSQC (CDCl$_3$, 25°C)

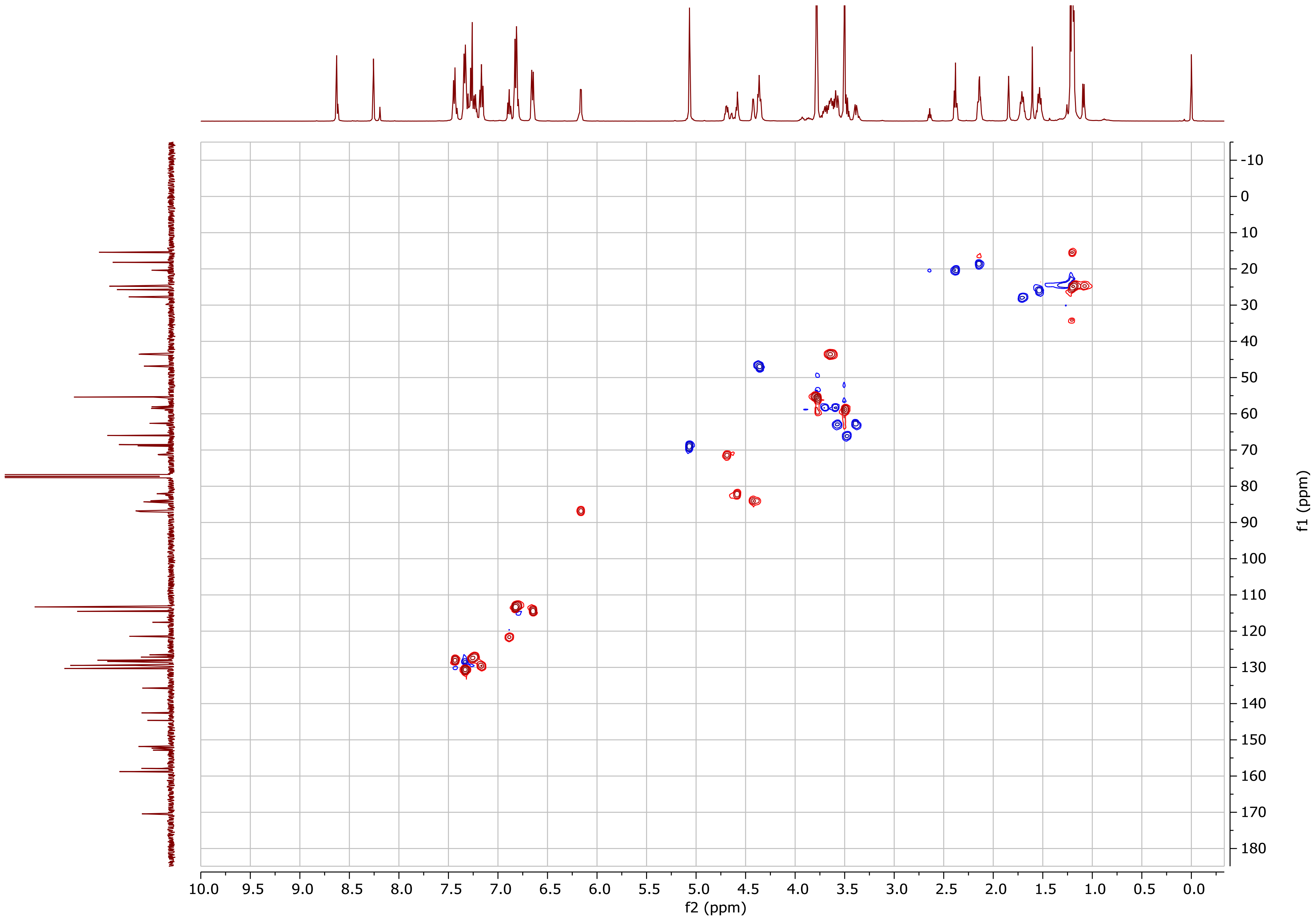

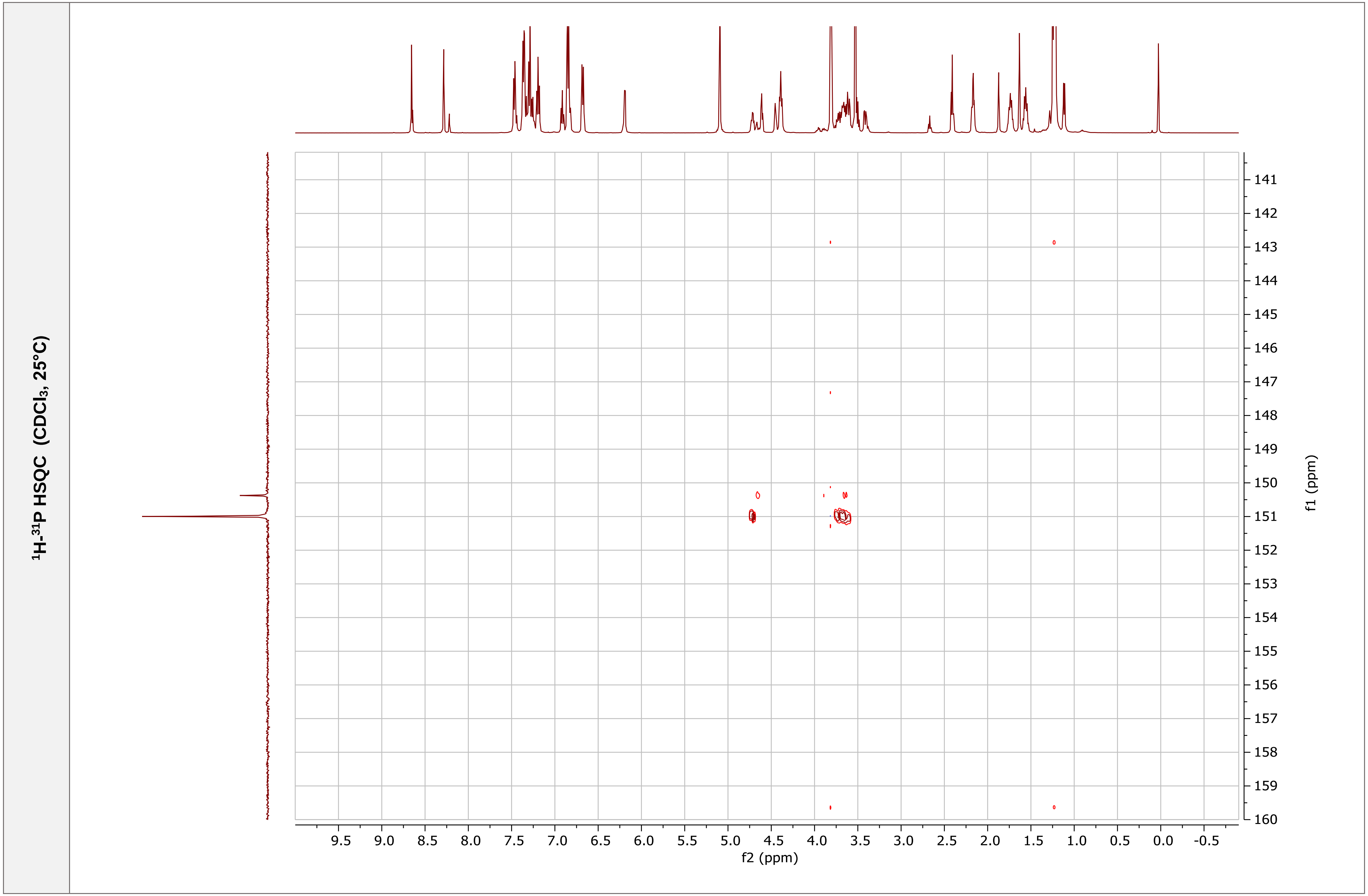

1H-31P HSQC (CDCl3, 25°C)
f2 (ppm)
f1 (ppm)

(1e) *N*6-(3-phthalimidopropyl)adenosine phosphoramidite (5′-*O*-DMT-2′-*O*-Me-PhthNp^{6}A^{Pac})

MS (+) ESI

(Calc. $[M+H]^+$ $C_{60}H_{66}N_8O_{11}P^+$ 1105.45832)

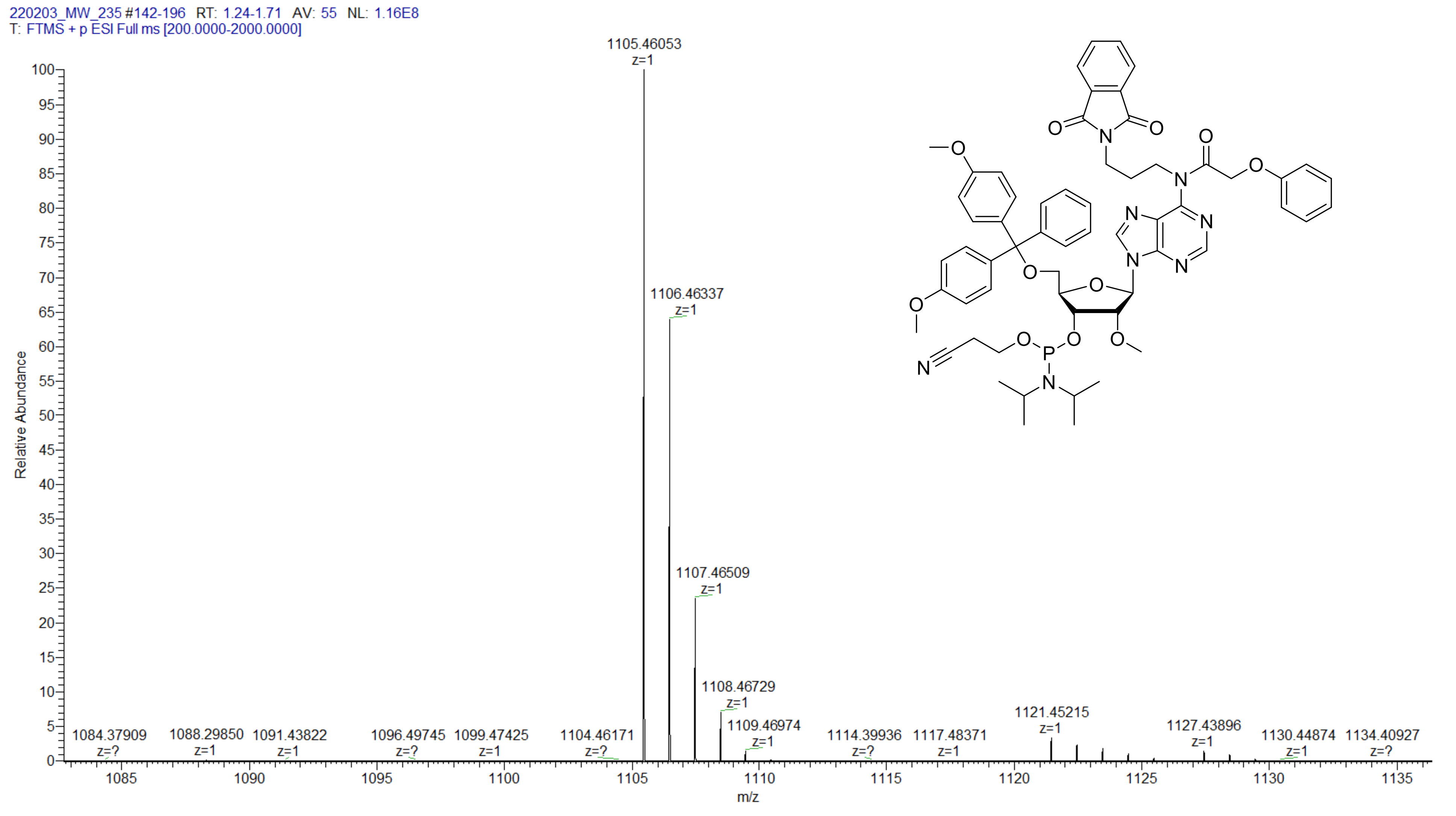

^{1}H NMR (500 MHz, CDCl$_3$, 25°C)

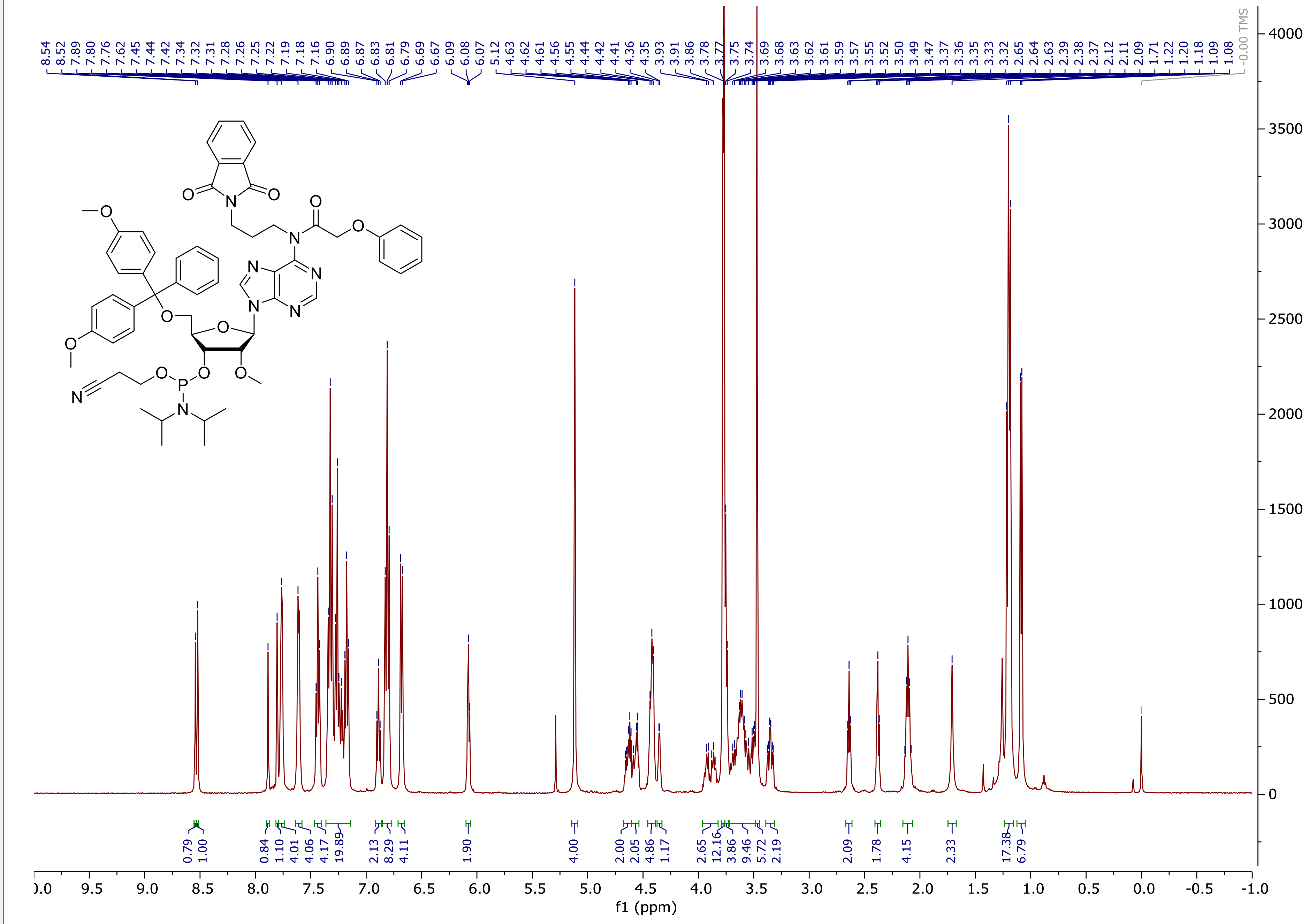

$^{13}C\{^1H\}$ NMR (126 MHz, $CDCl_3$, 25°C)

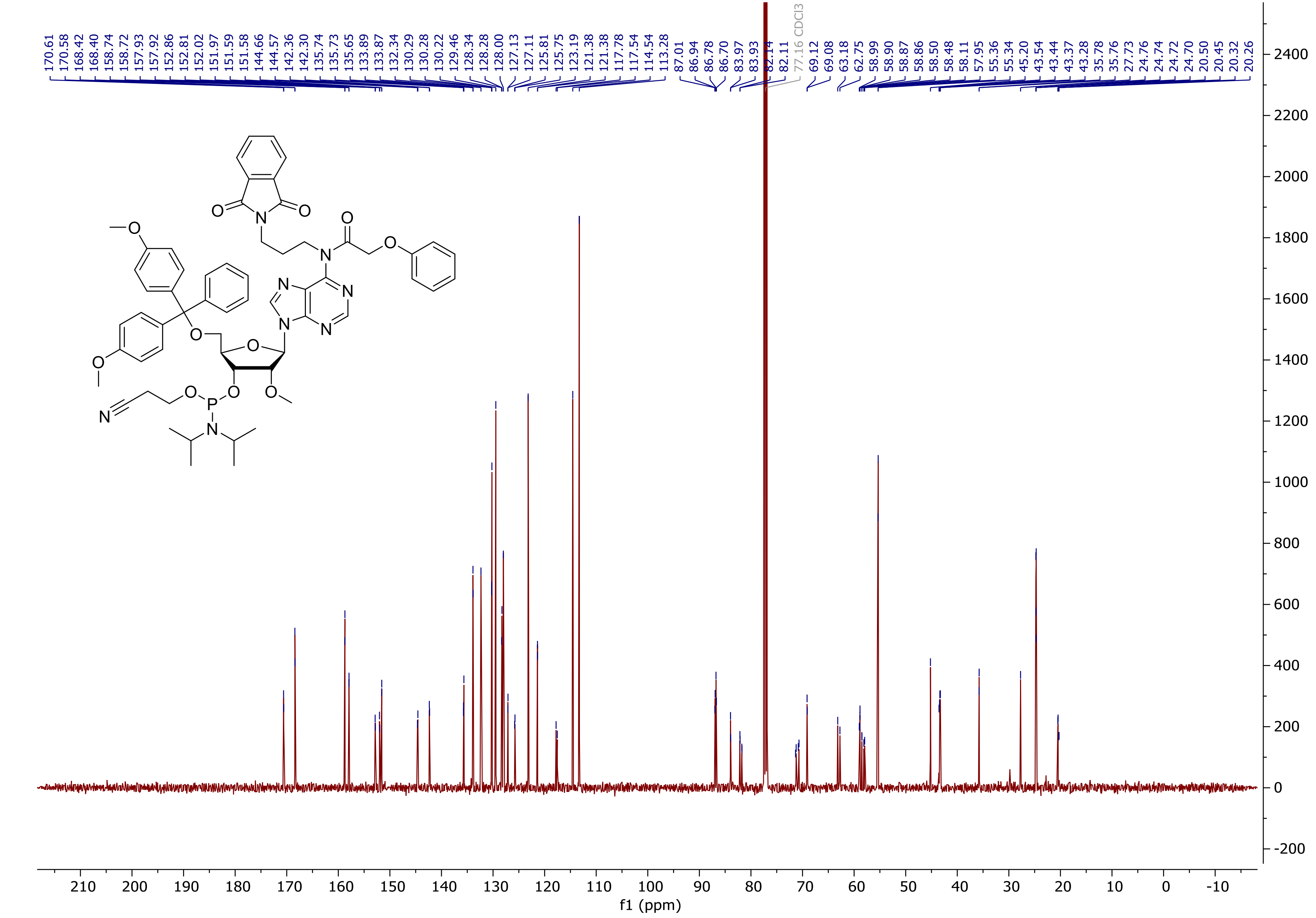

^{1}P NMR (202.5 MHz, $CDCl_3$, 25°C)

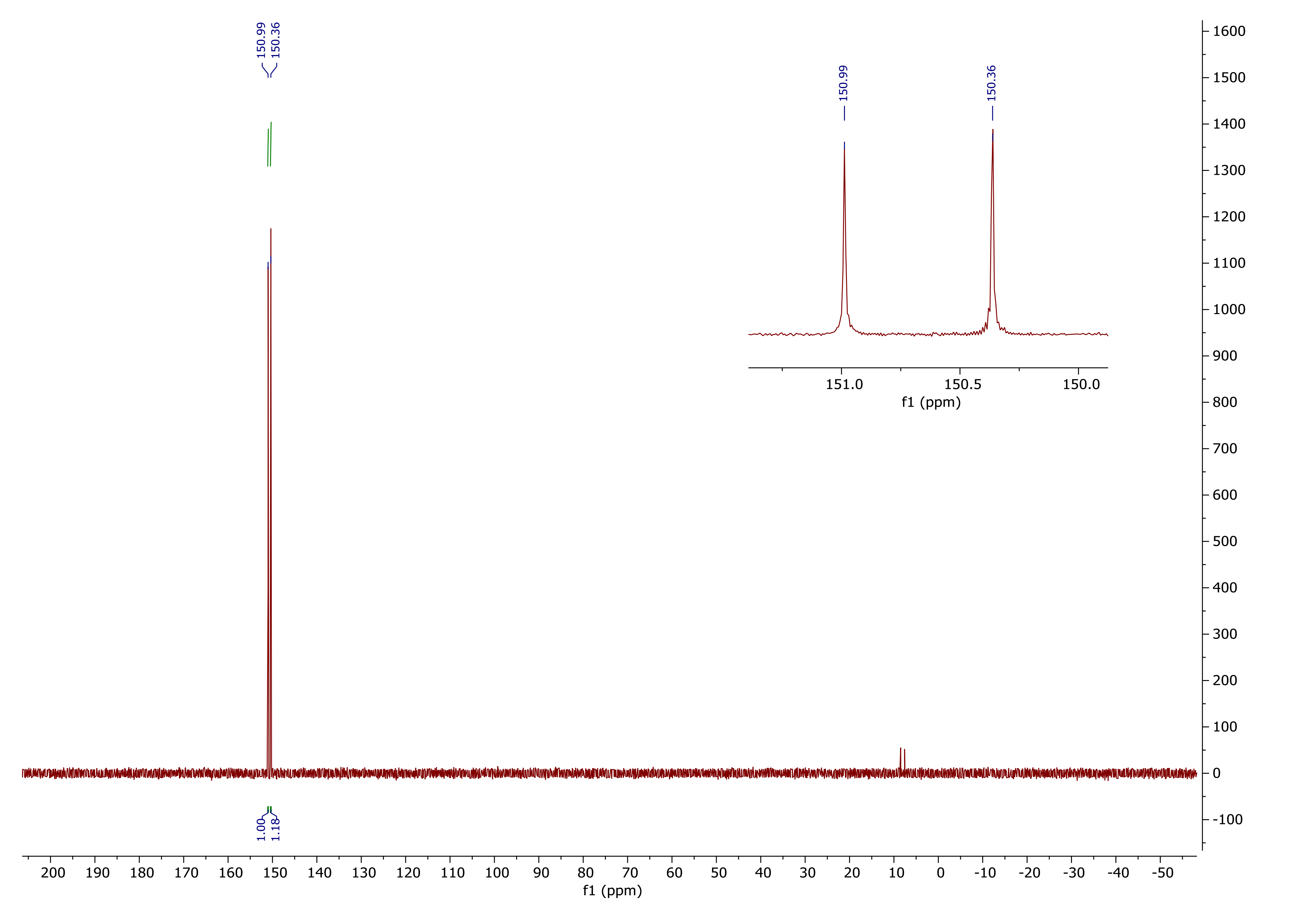

COSY NMR (CDCl$_3$, 25°C)

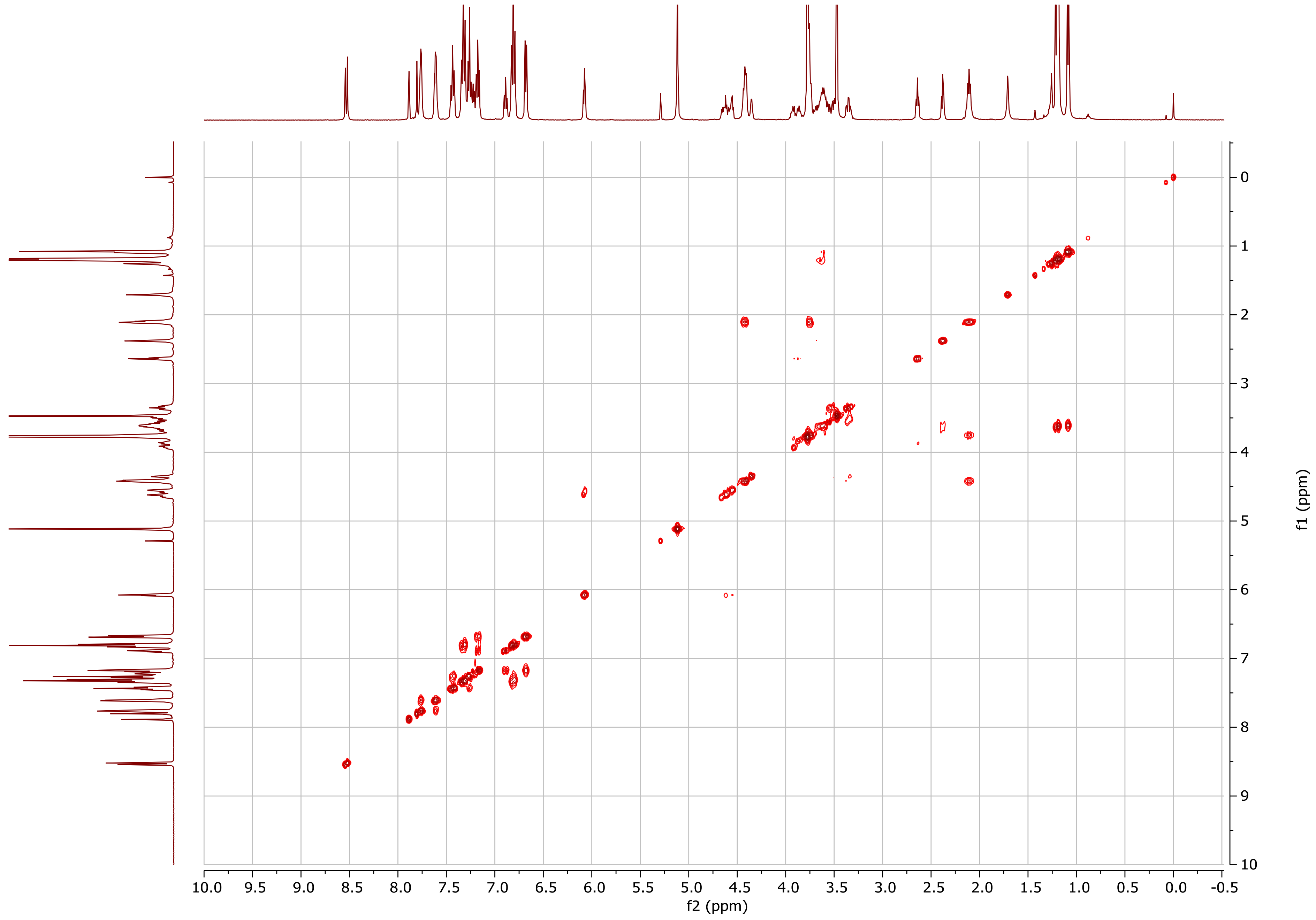

^{1}H-^{13}C HSQC ($CDCl_3$, 25°C)

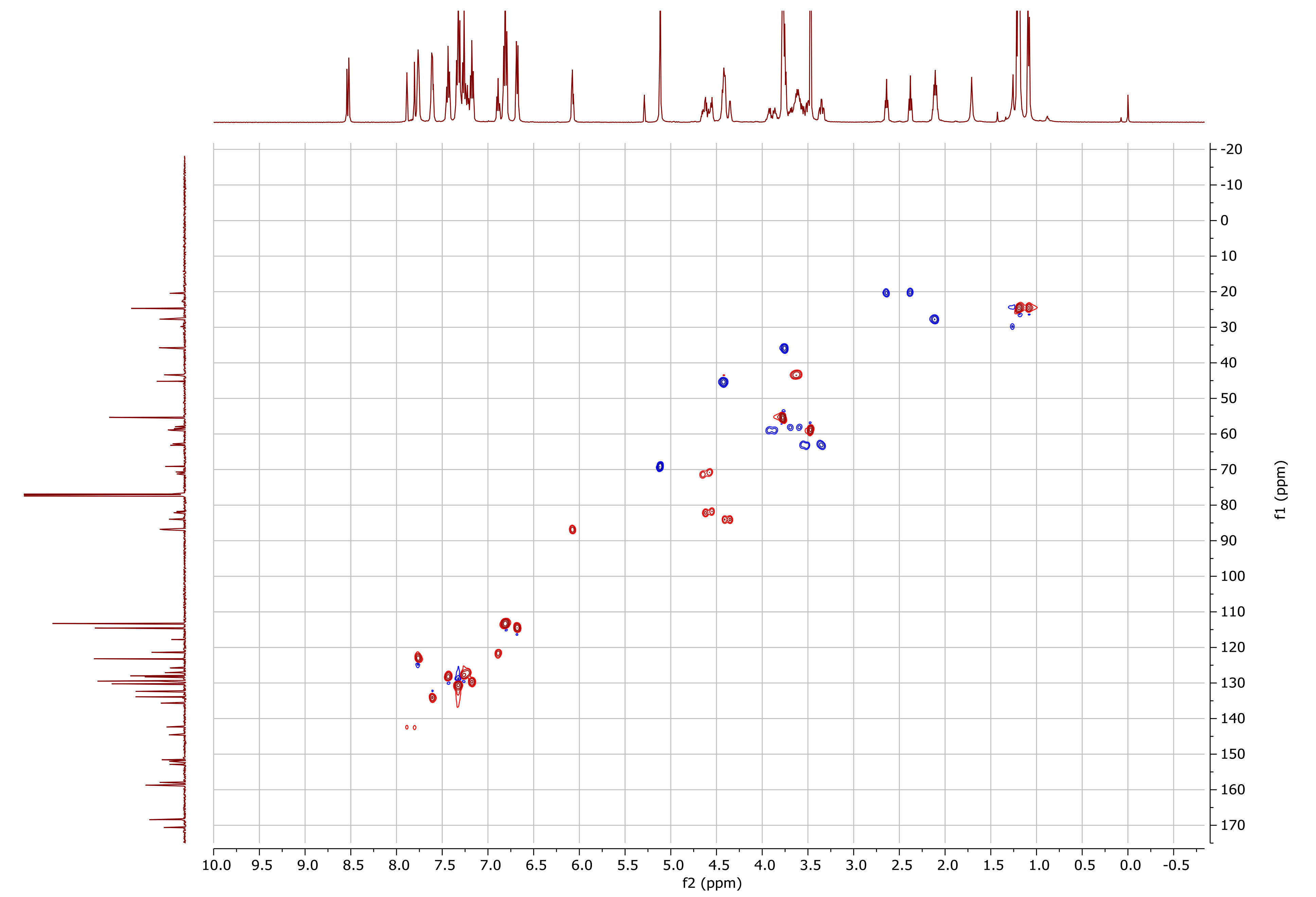

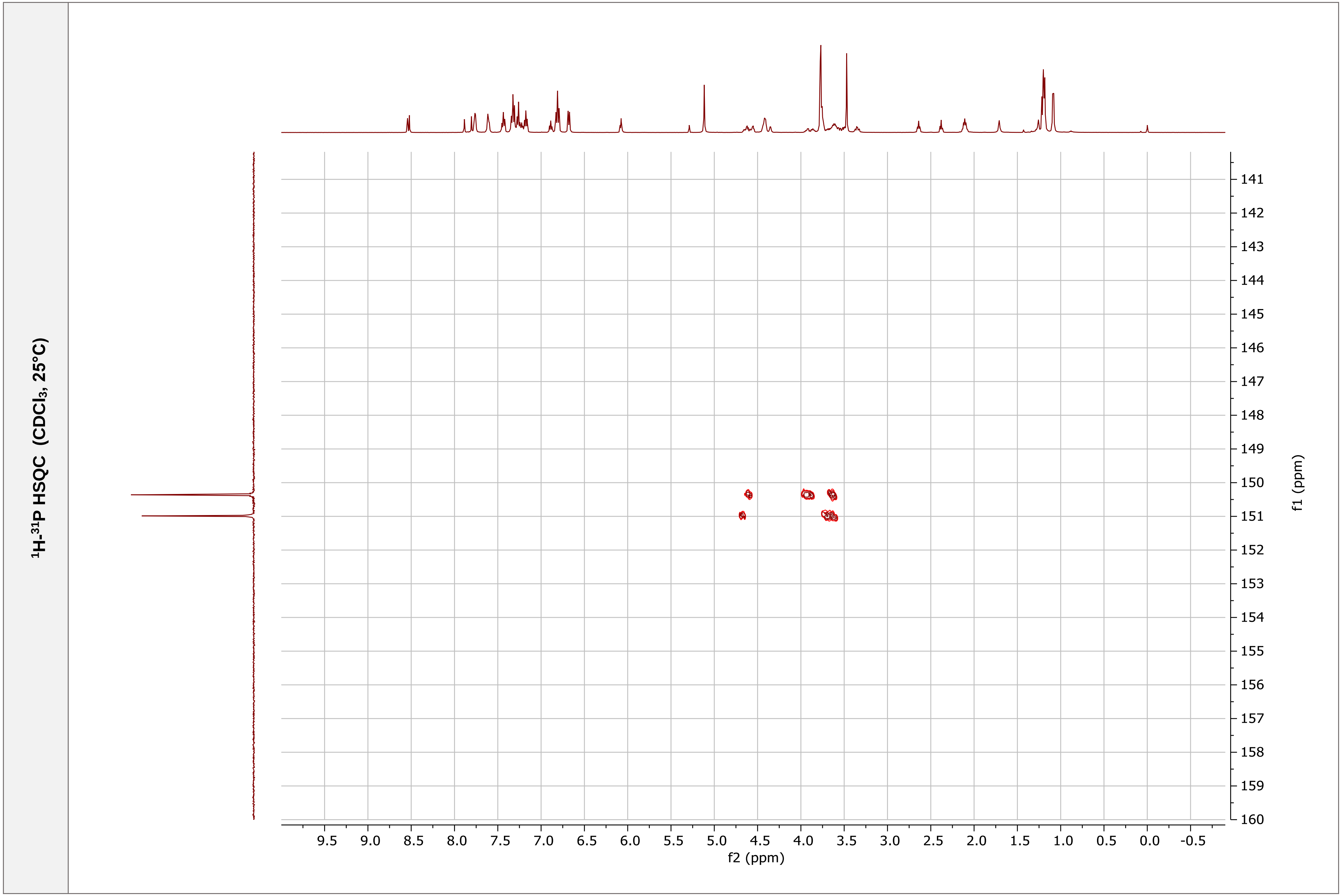

^{1}H-^{31}P HSQC (CDCl$_3$, 25°C)
f2 (ppm)
f1 (ppm)
9.5 9.0 8.5 8.0 7.5 7.0 6.5 6.0 5.5 5.0 4.5 4.0 3.5 3.0 2.5 2.0 1.5 1.0 0.5 0.0 -0.5
141 142 143 144 145 146 147 148 149 150 151 152 153 154 155 156 157 158 159 160

(1f) *N*6-isopropyladenosine phosphoramidite (5′-*O*-DMT-2′-*O*-Me-iPr^{6}A^{Pac})

MS (+) ESI

(Calc. $[M+H]^+$ $C_{52}H_{63}N_7O_9P^+$ 960.44194)

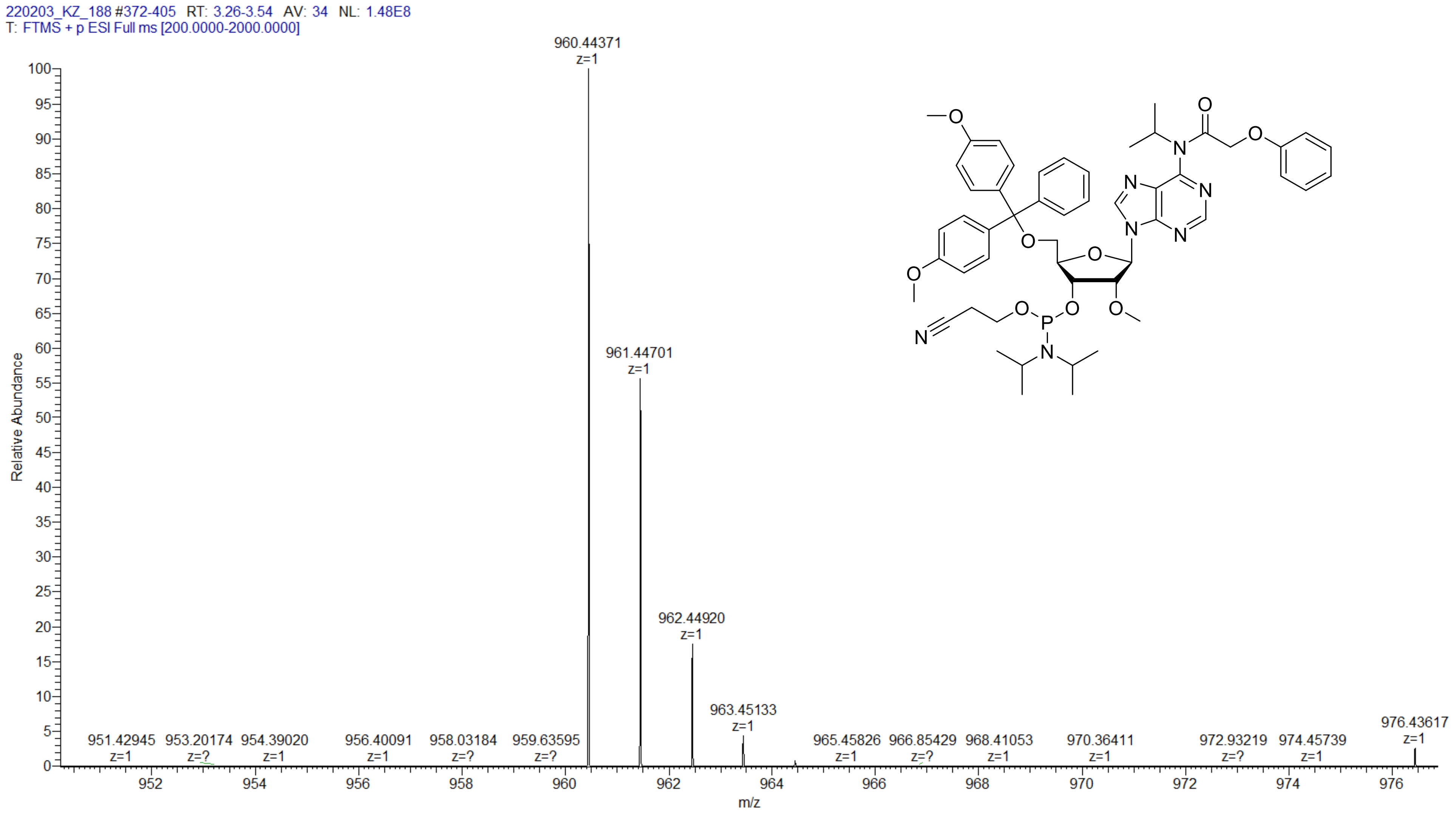

¹H NMR (500 MHz, CDCl$_3$, 25°C)

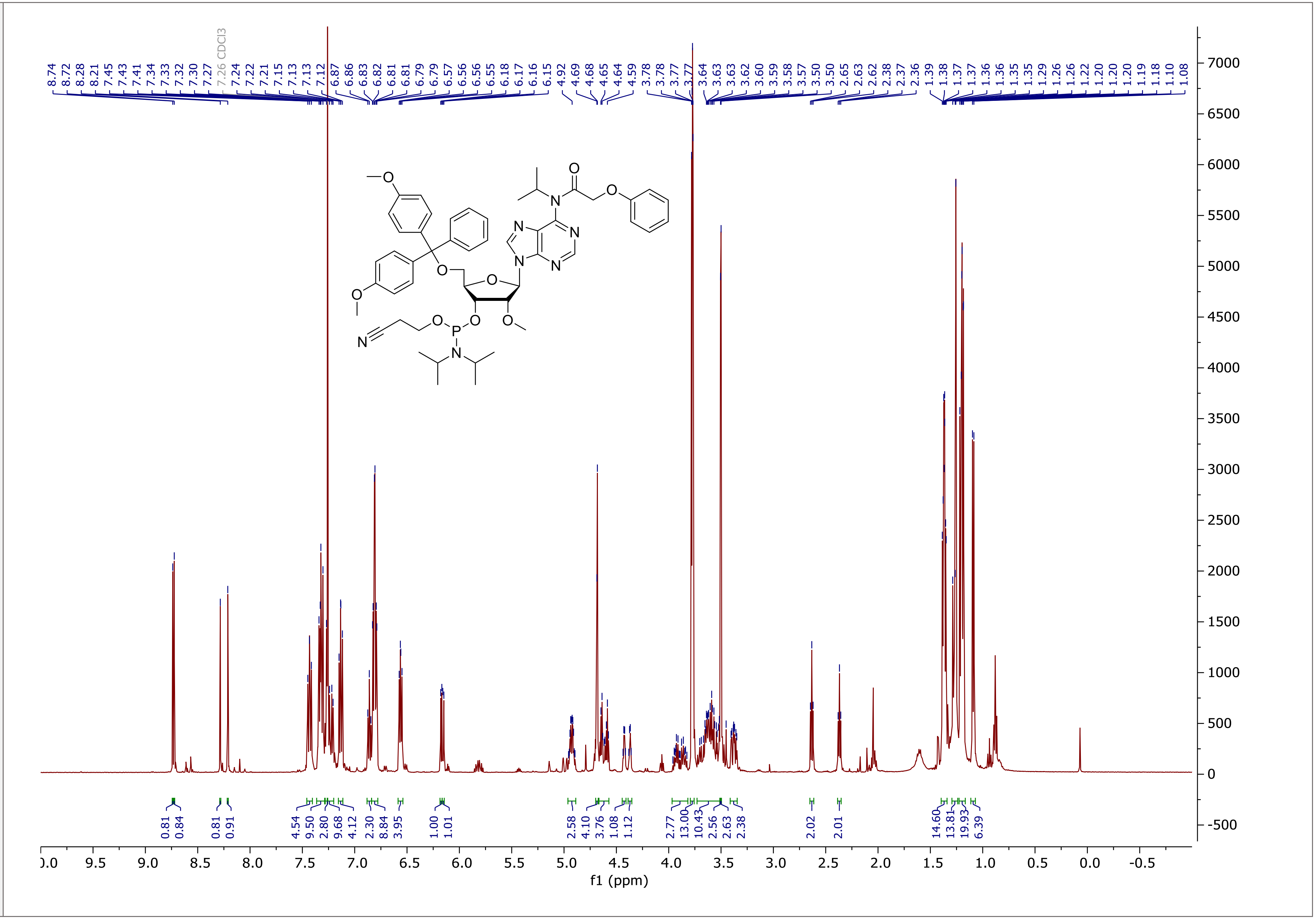

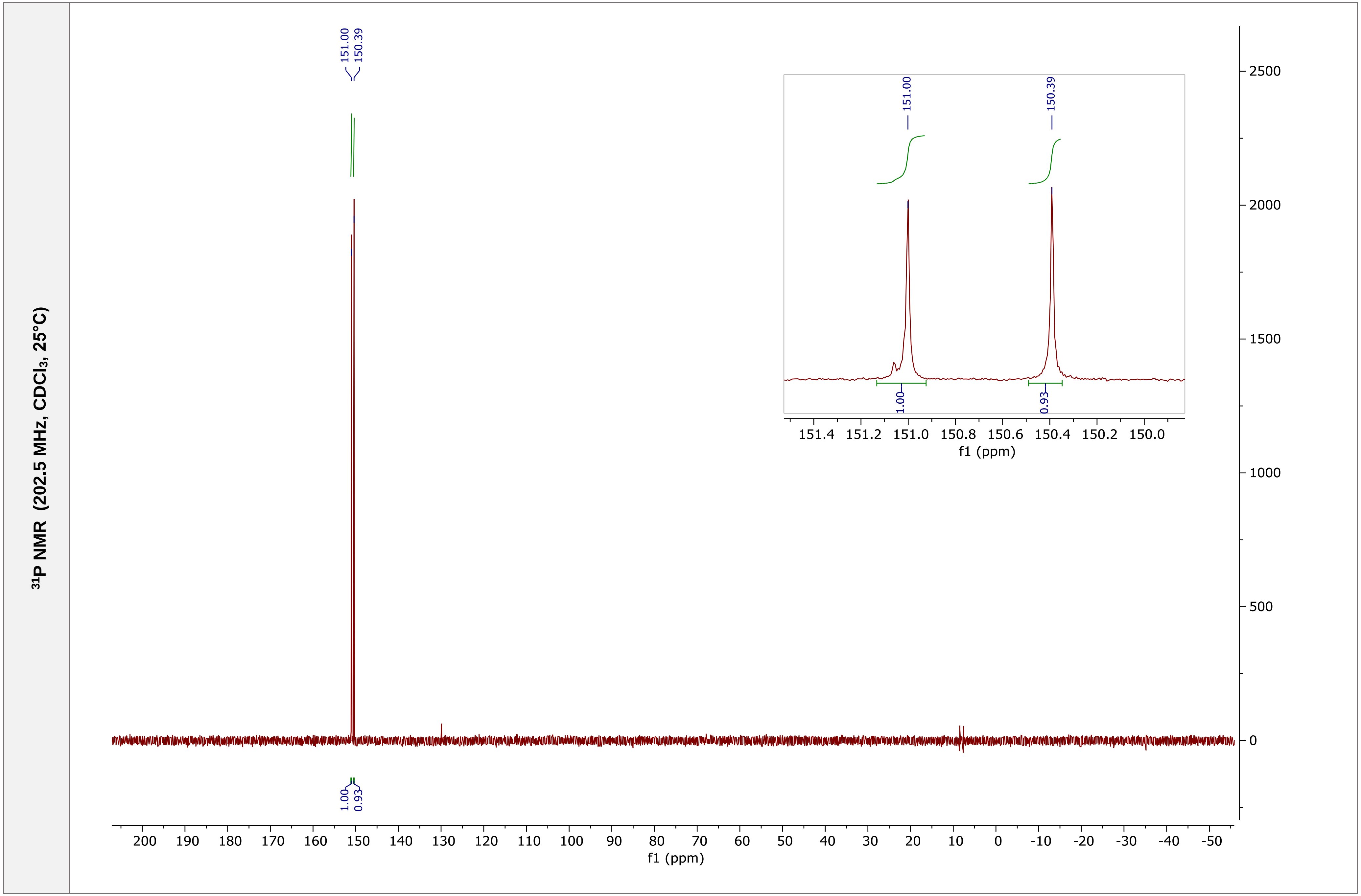

^{31}P NMR (202.5 MHz, $CDCl_3$, 25°C)
151.00
150.39
1.00
0.93
f1 (ppm)

COSY NMR (CDCl$_3$, 25°C)

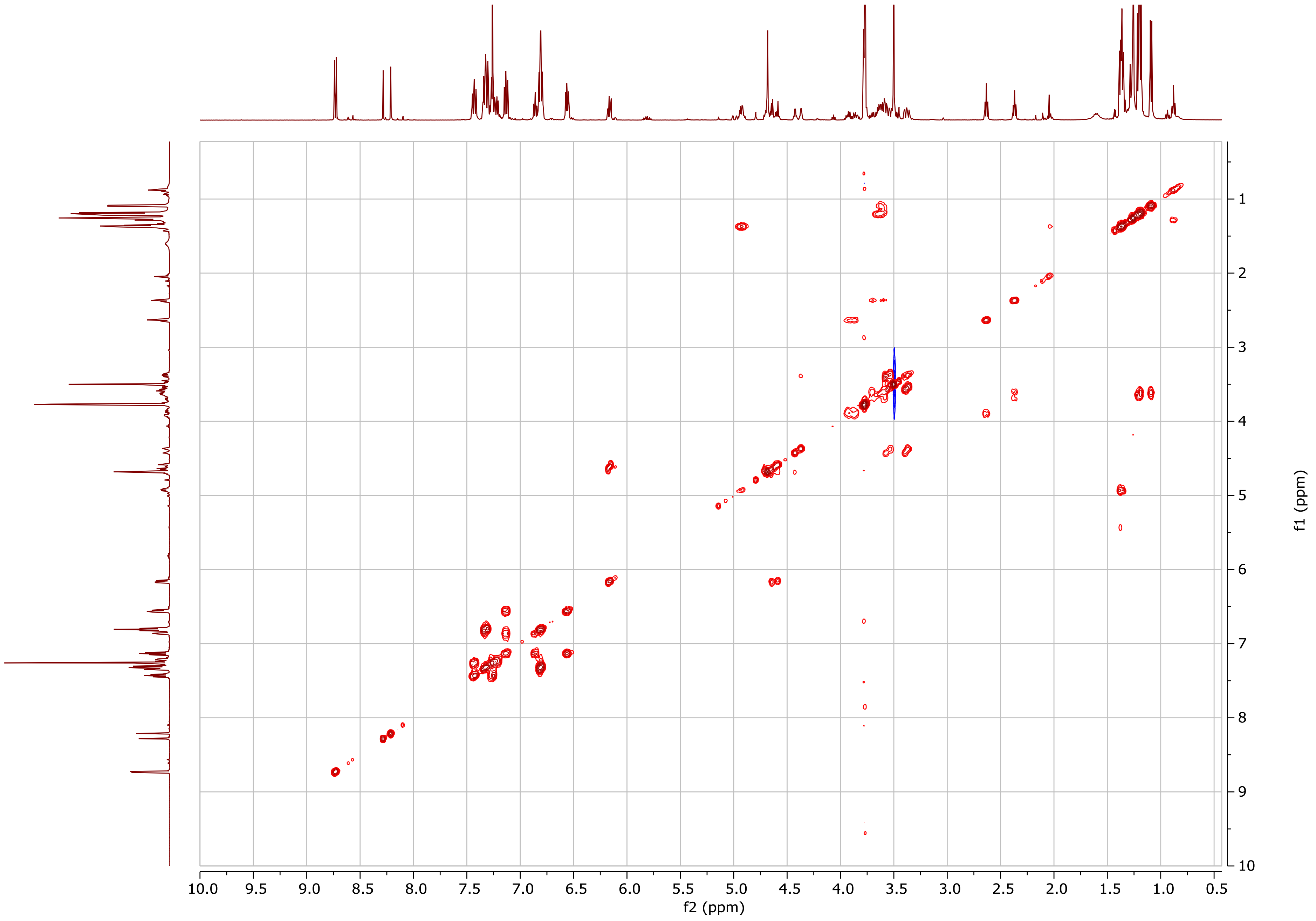

^{1}H-^{13}C HSQC (CDCl$_3$, 25°C)

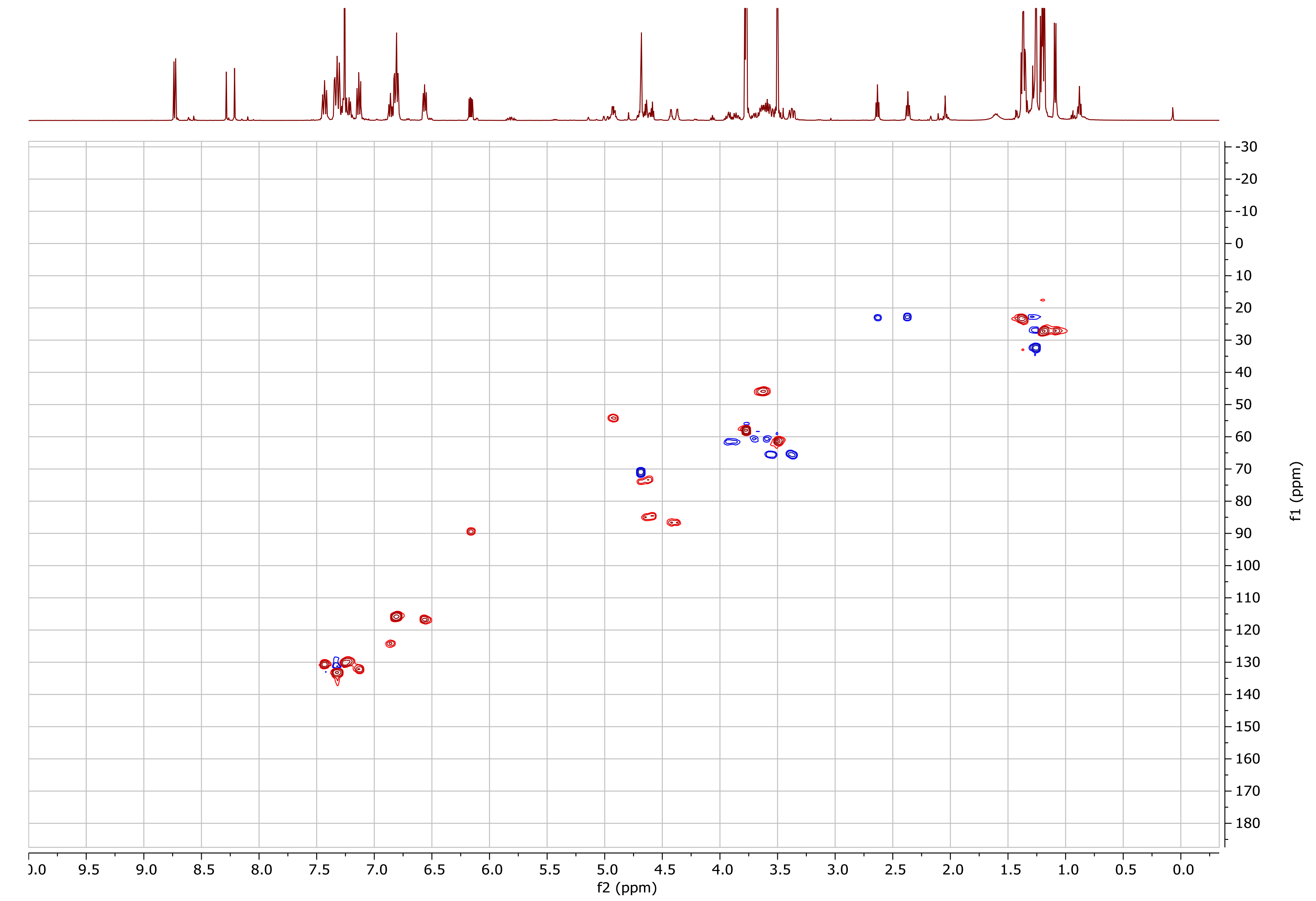

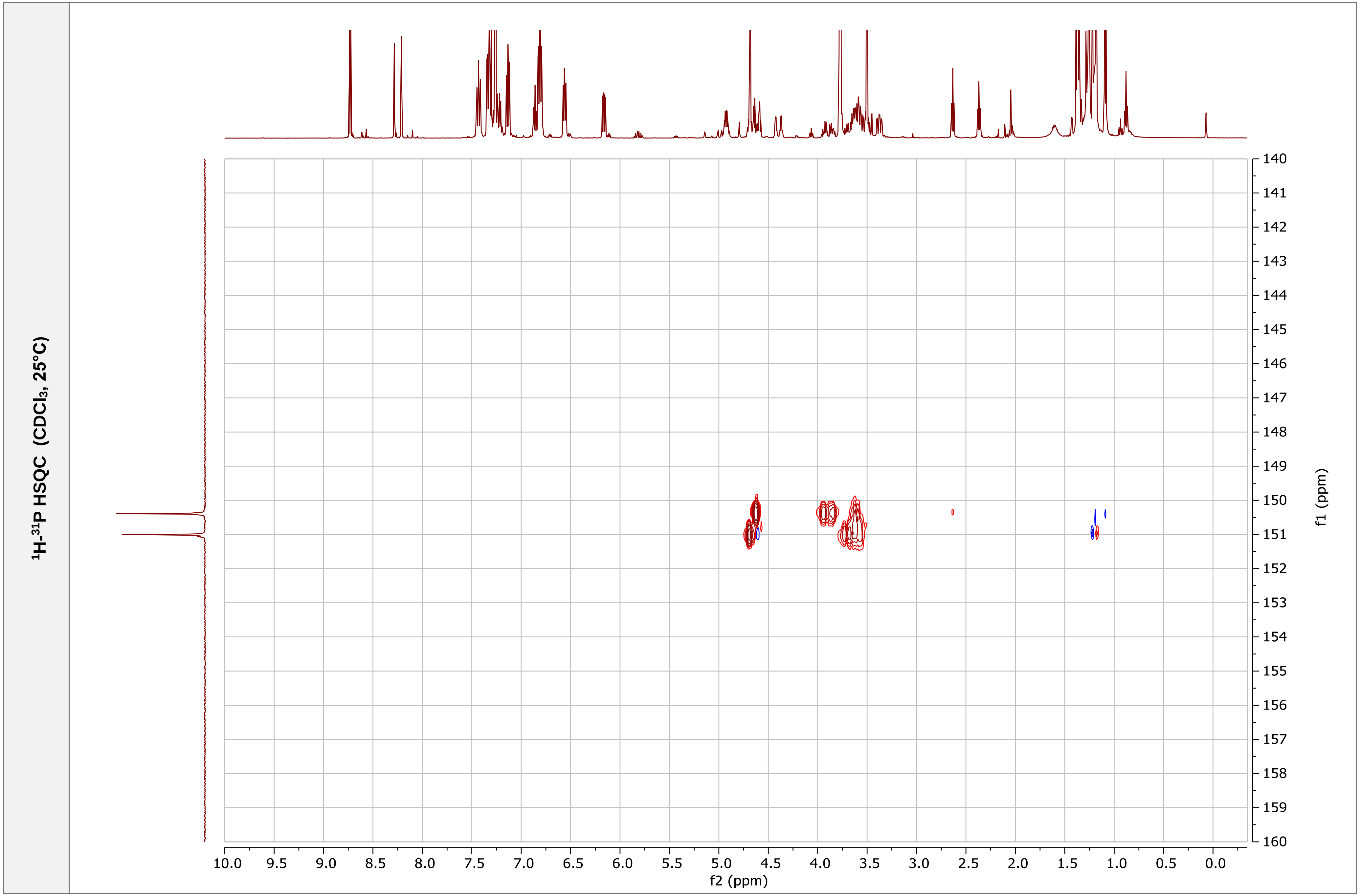

¹H-³¹P HSQC (CDCl₃, 25°C)
f2 (ppm)
f1 (ppm)

(1g) *N*6-methyladenosine phosphoramidite (5′-*O*-DMT-2′-*O*-TBDMS-m^{6}A^{Bz})

MS (+) ESI

(Calc. [M+H]$^+$ $C_{54}H_{69}N_7O_8PSi^+$ 1002.47090)

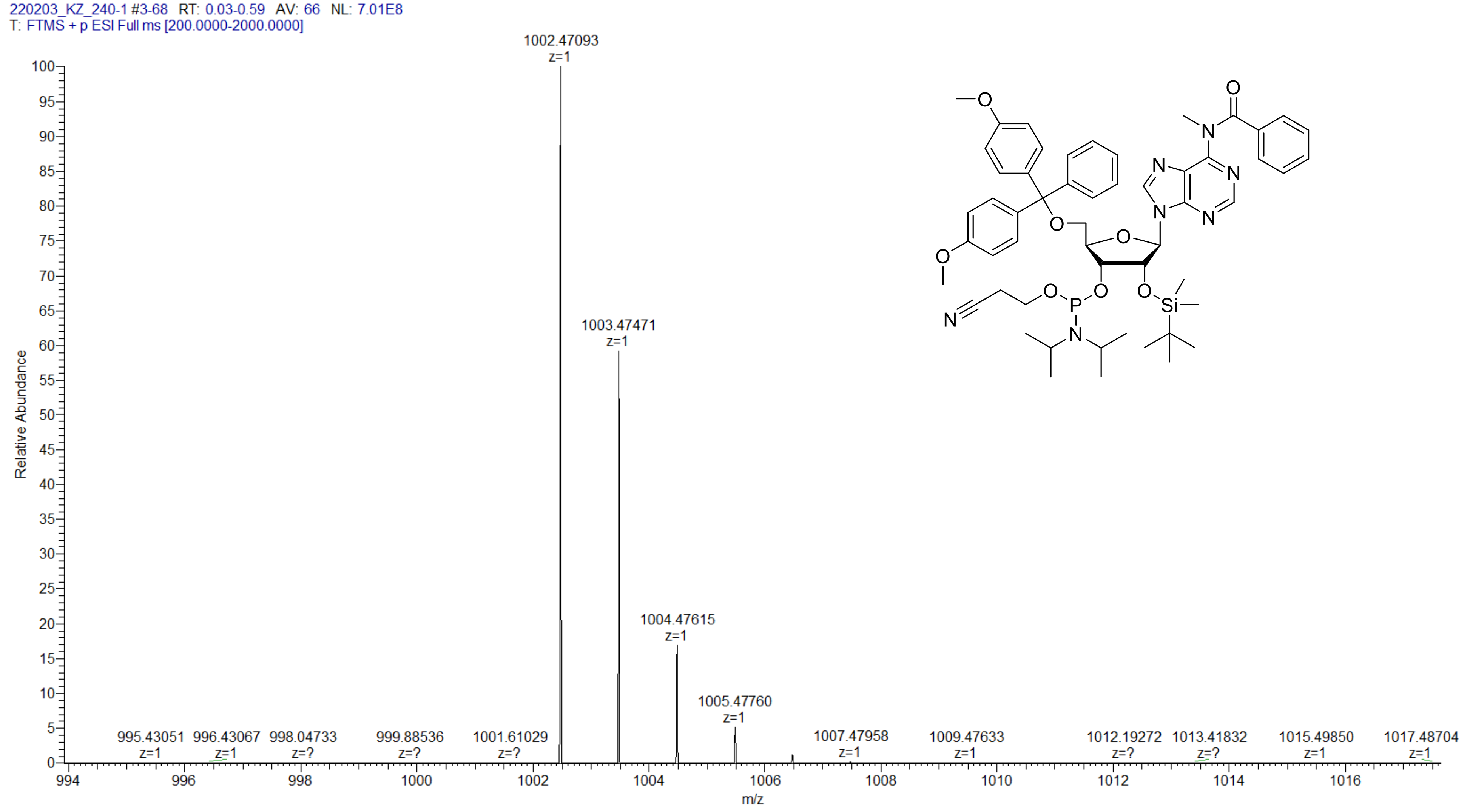

^1H NMR (500 MHz, $CDCl_3$, 25°C)

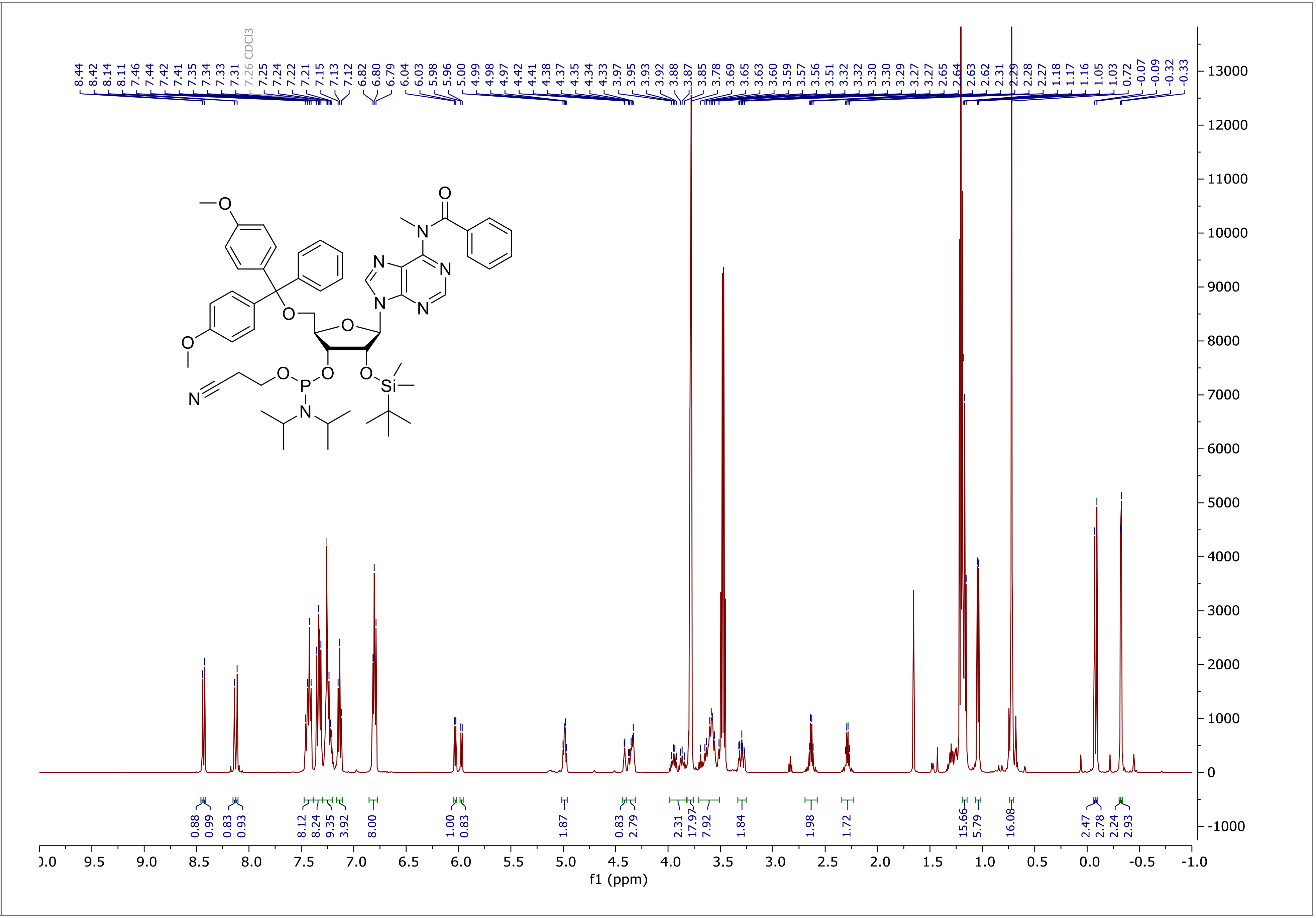

$^{13}C\{^{1}H\}$ NMR (126 MHz, $CDCl_3$, 25°C)

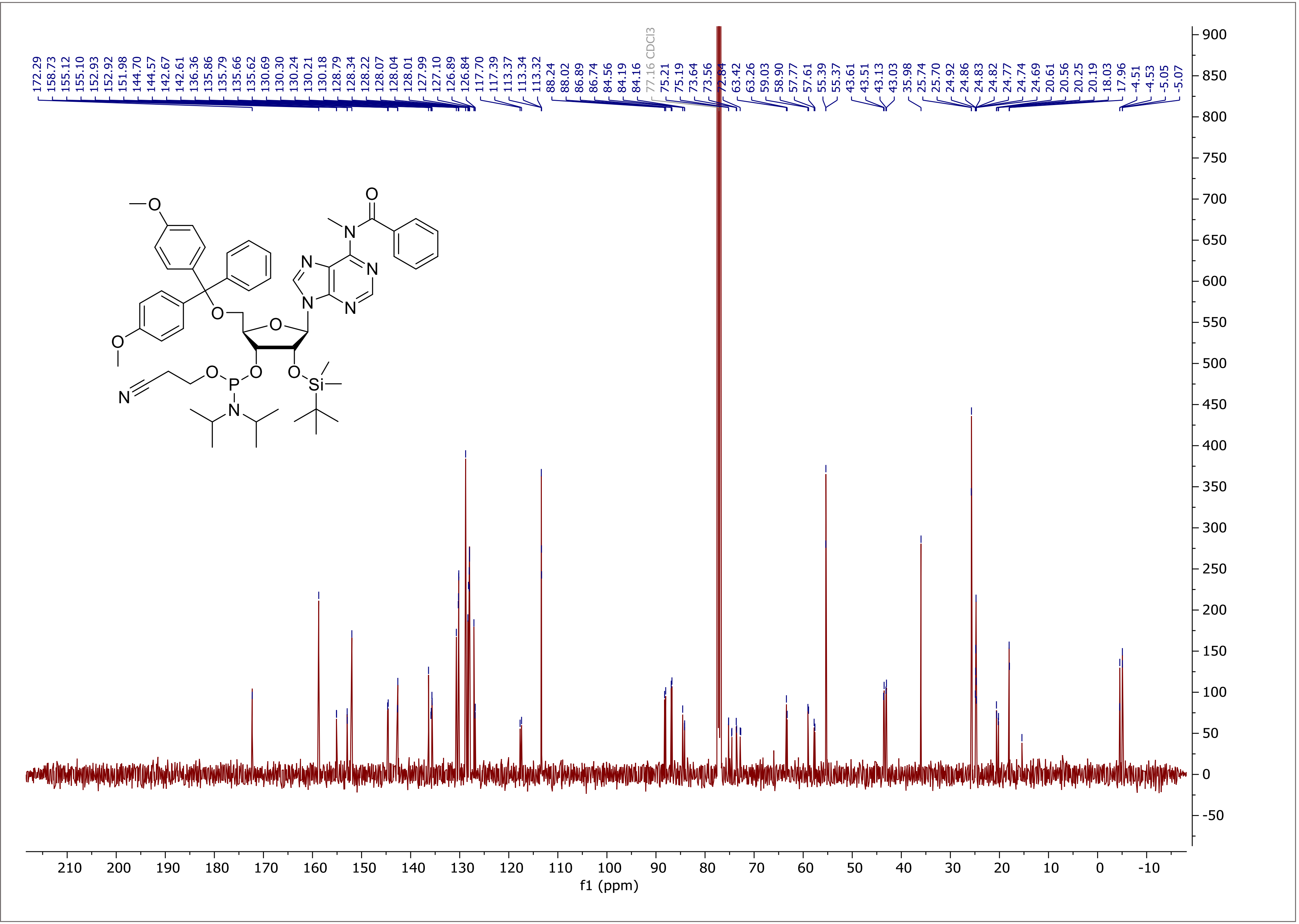

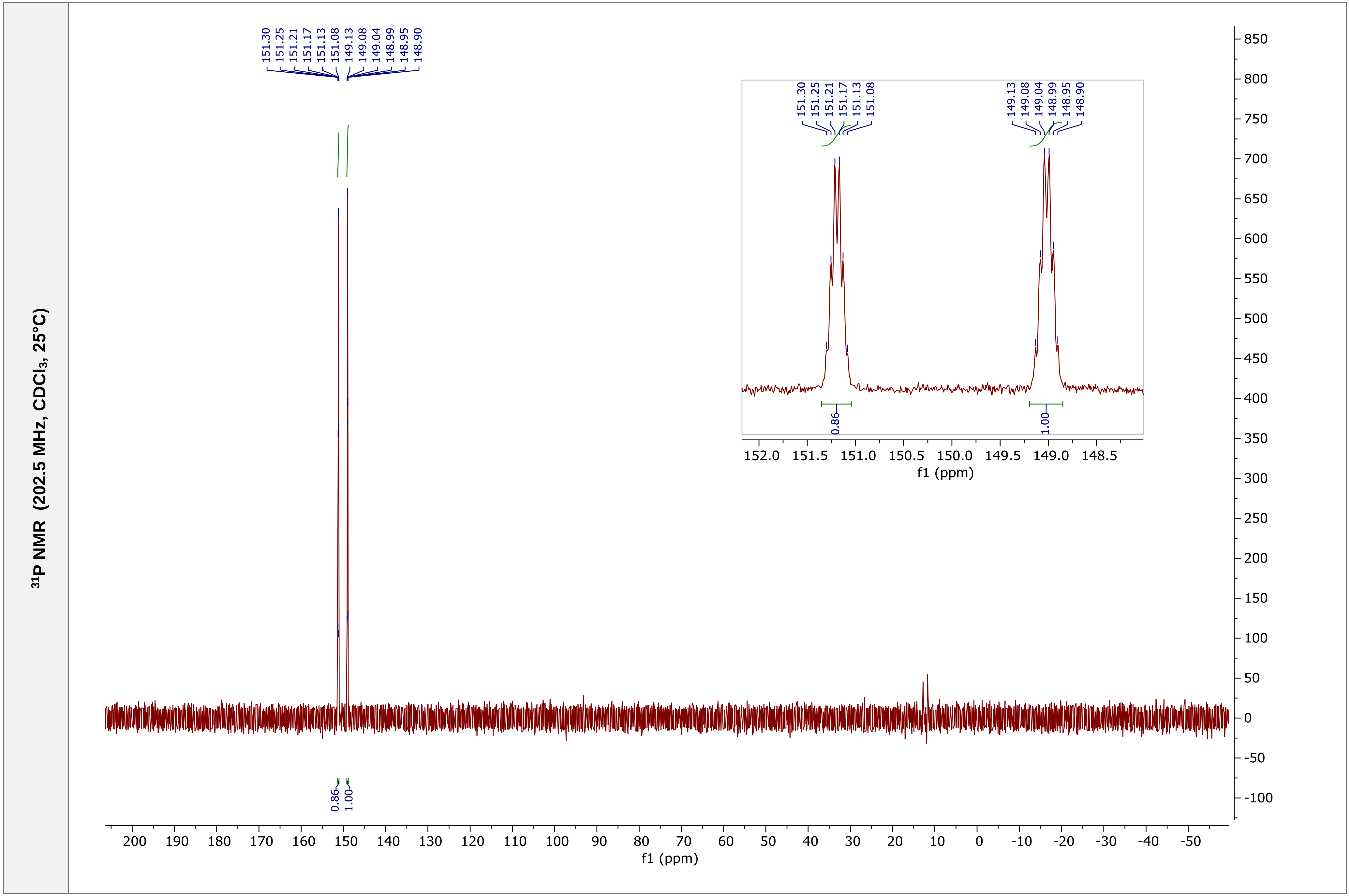
^{31}P NMR (202.5 MHz, CDCl$_3$, 25°C)
151.30
151.25
151.21
151.17
151.13
151.08
149.13
149.08
149.04
148.99
148.95
148.90
0.86
1.00
f1 (ppm)

COSY NMR (CDCl₃, 25°C)

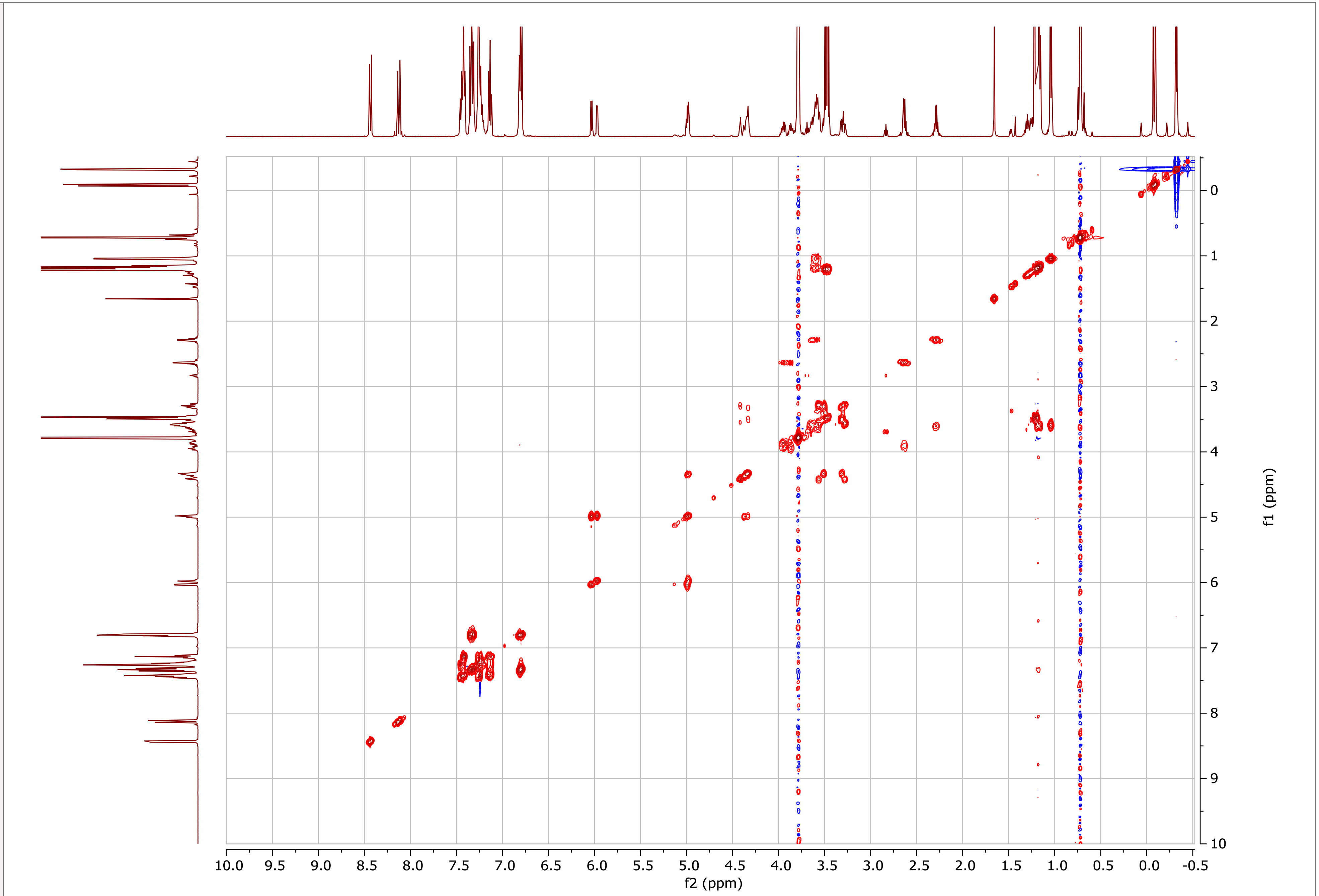

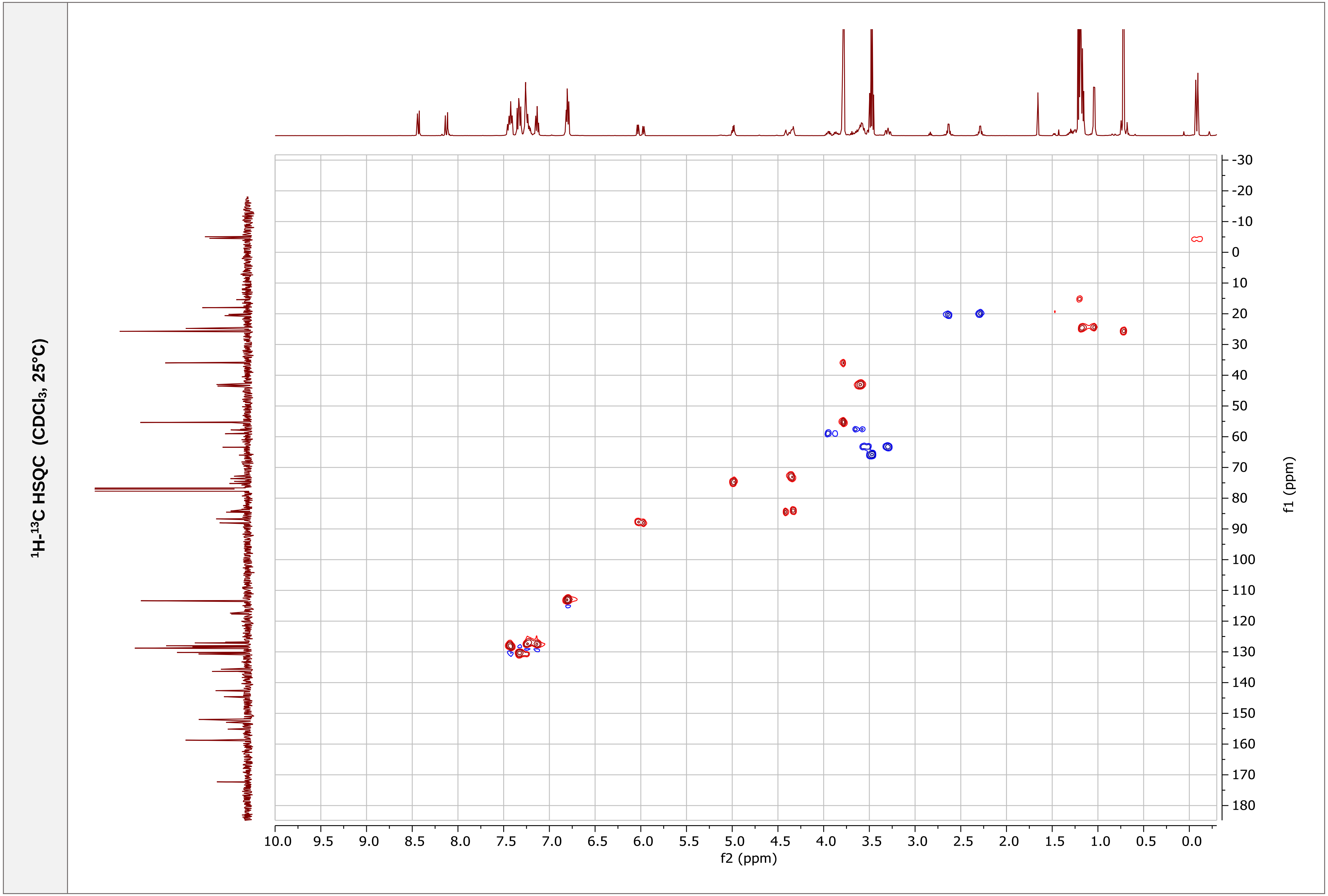

^1H-^{13}C HSQC (CDCl$_3$, 25°C)
f2 (ppm)
f1 (ppm)

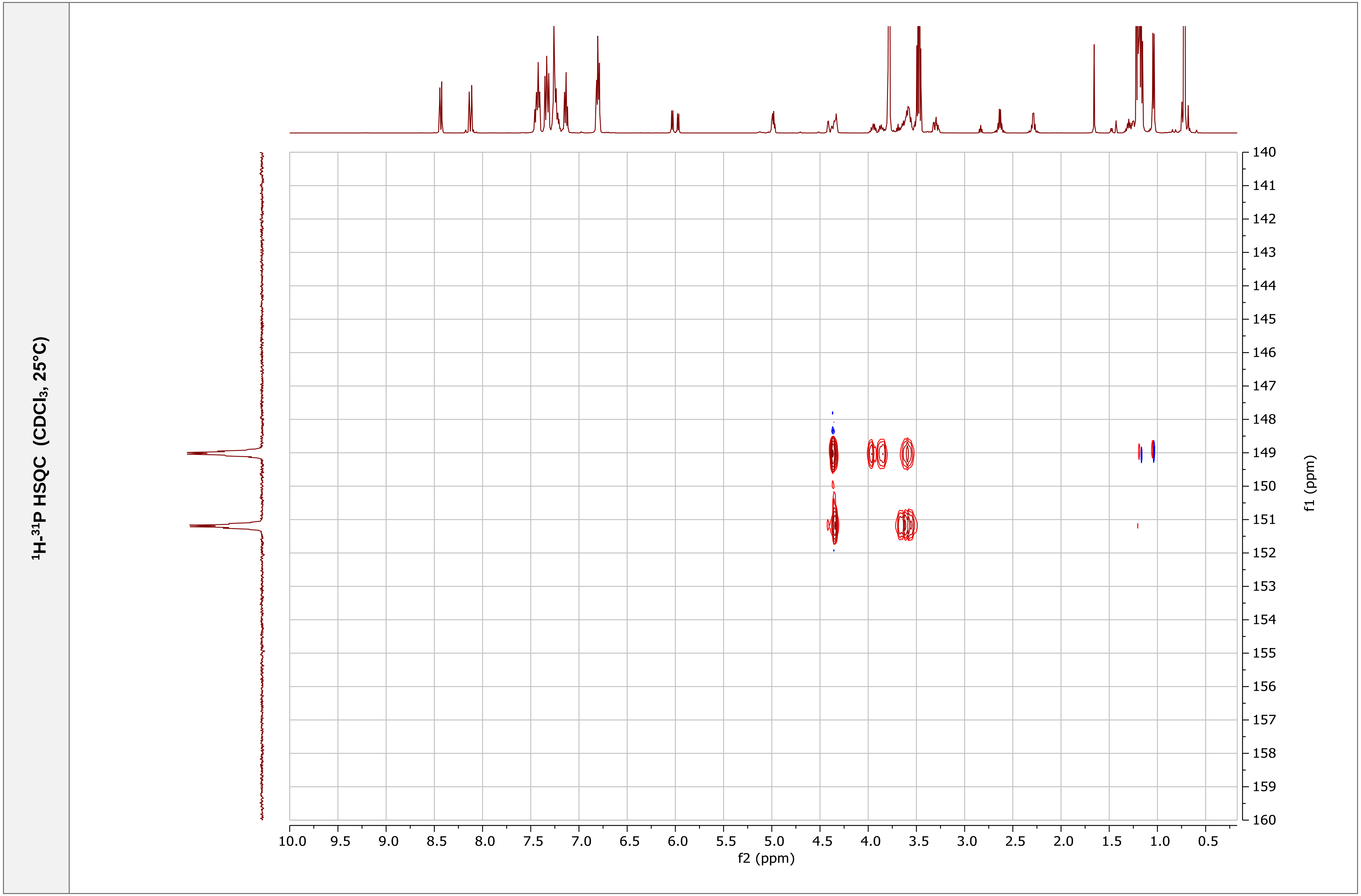

^{1}H-^{31}P HSQC (CDCl$_3$, 25°C)
f2 (ppm)
f1 (ppm)

(2) N1-methyladenosine phosphoramidite (5′-*O*-DMT-2′-*O*-TBDMS-$^{m1}A^{Bz}$)

MS (+) ESI

(Calc. $[M+H]^+$ $C_{54}H_{69}N_7O_8PSi^+$ 1002.47090)

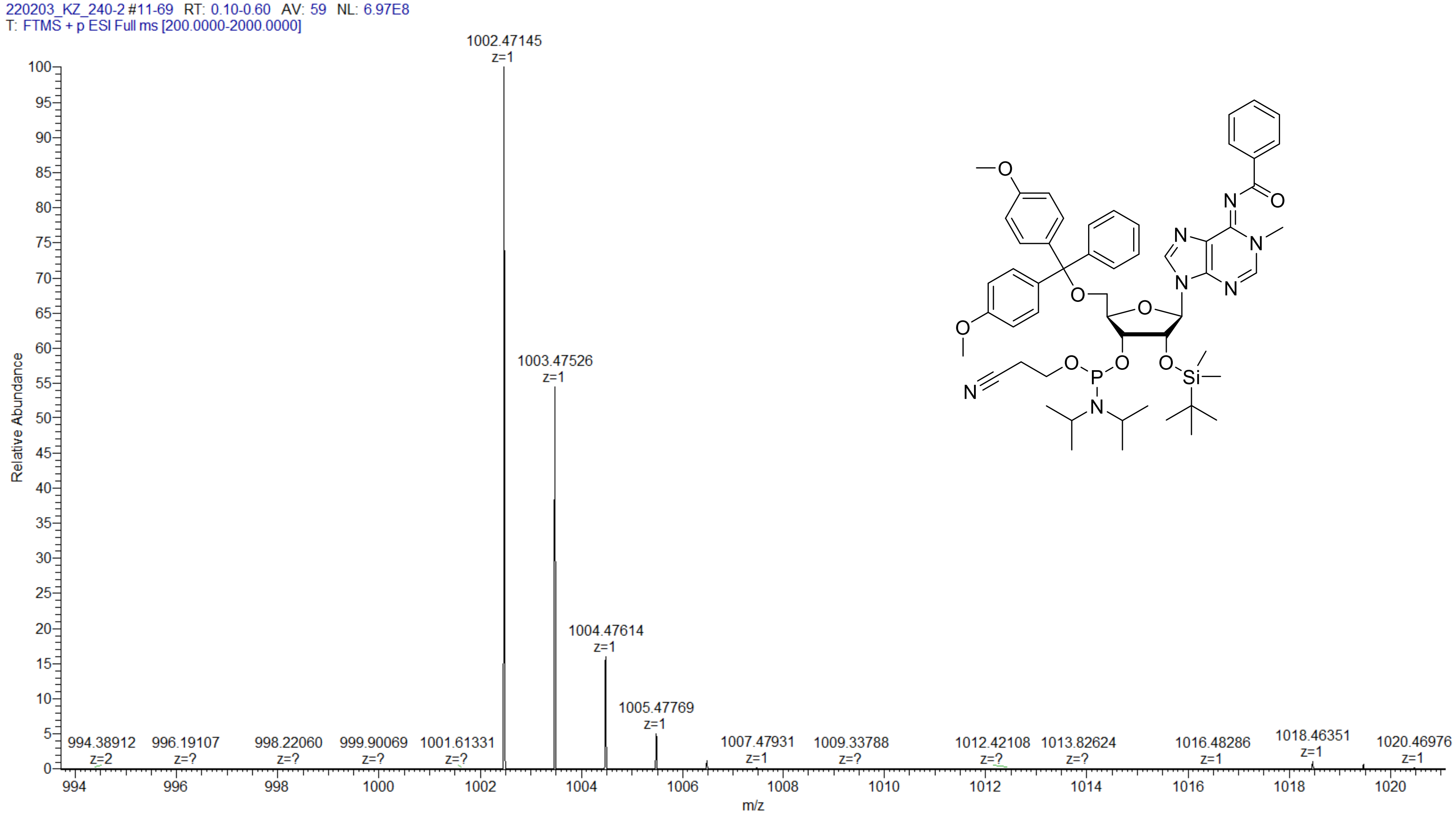

^1H NMR (500 MHz, $CDCl_3$, 25°C)

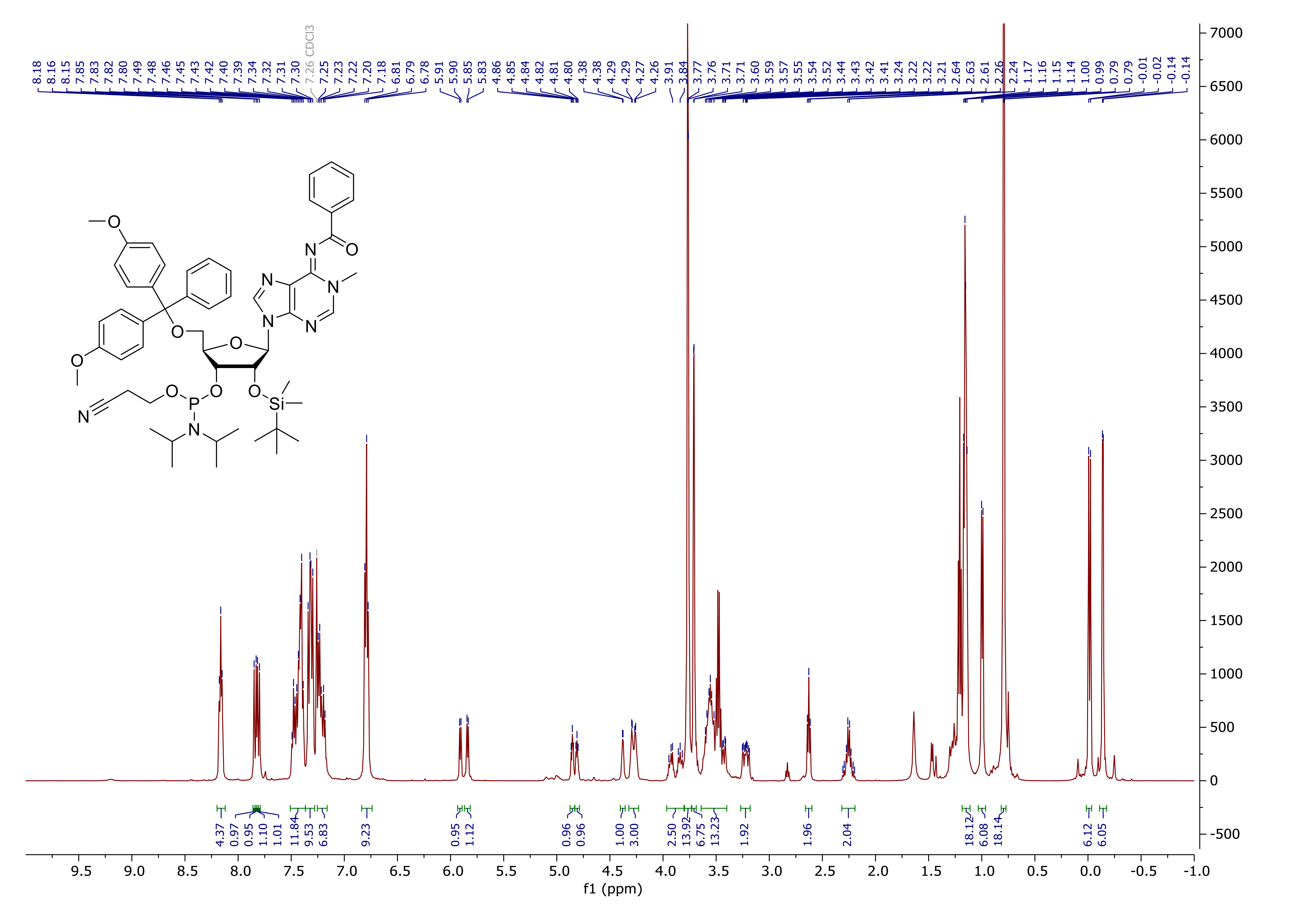

$^{13}C\{^{1}H\}$ NMR (126 MHz, $CDCl_3$, 25°C)

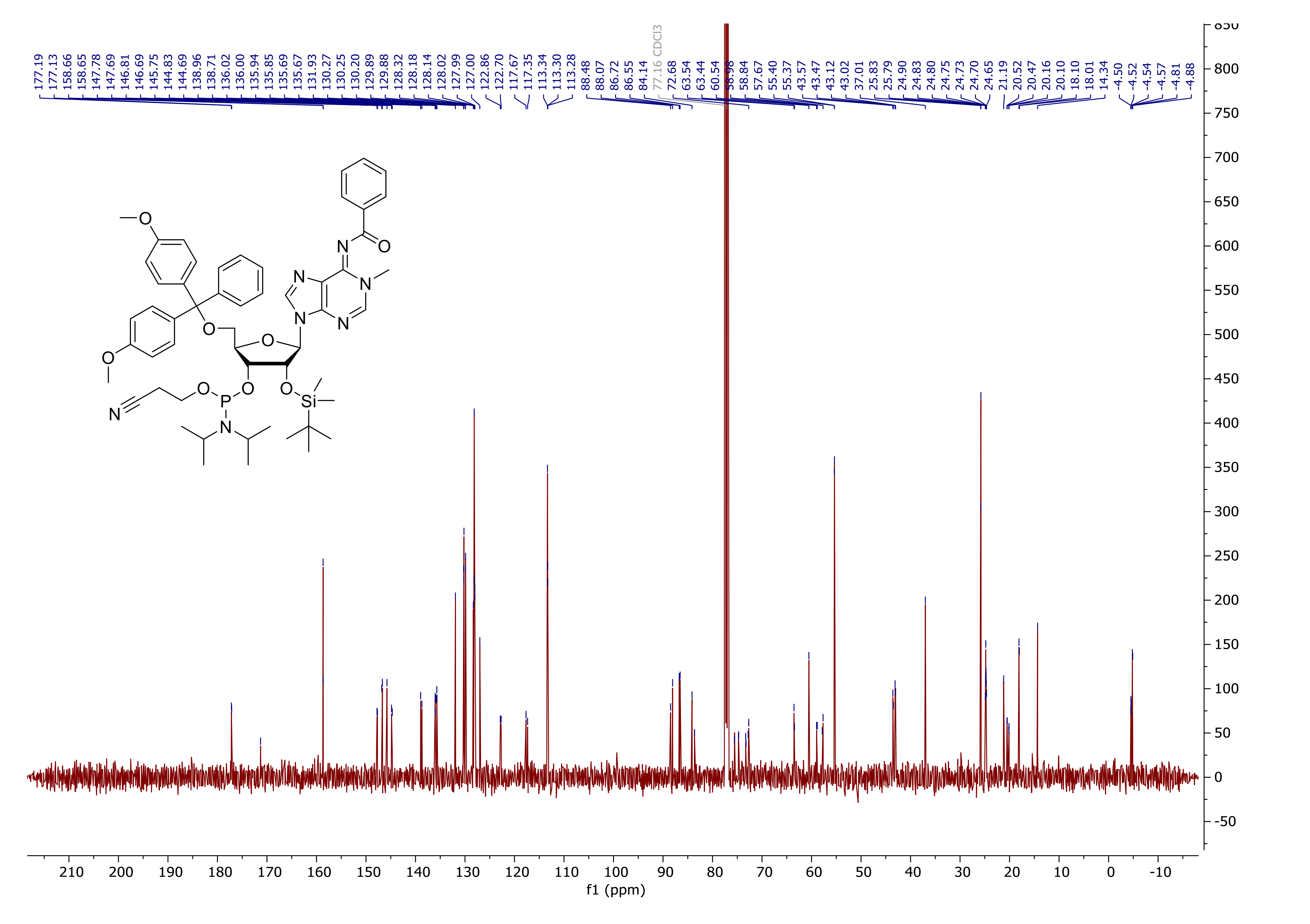

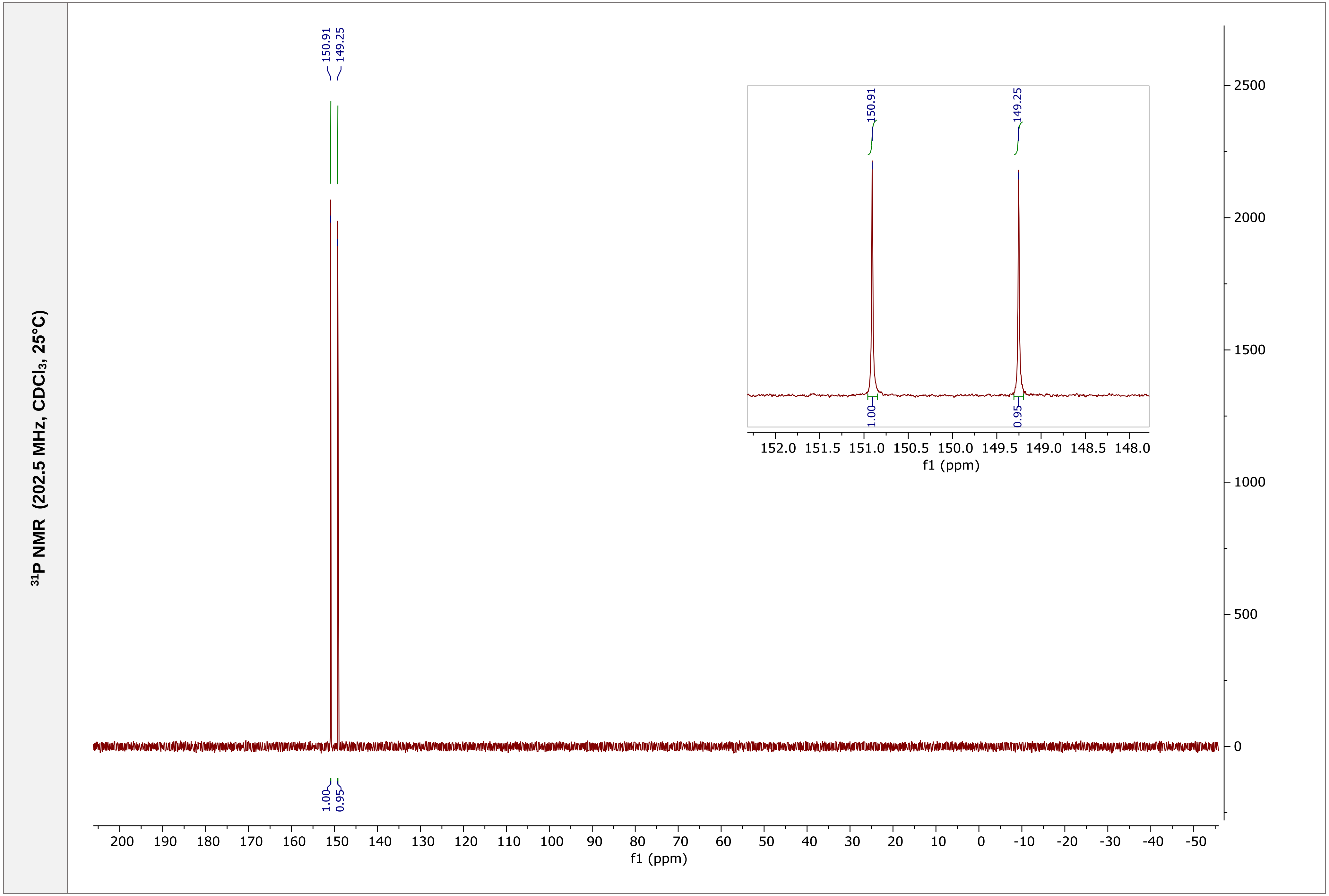

^{31}P NMR (202.5 MHz, CDCl$_3$, 25°C)
150.91
149.25
1.00
0.95
f1 (ppm)

COSY NMR (CDCl$_3$, 25°C)

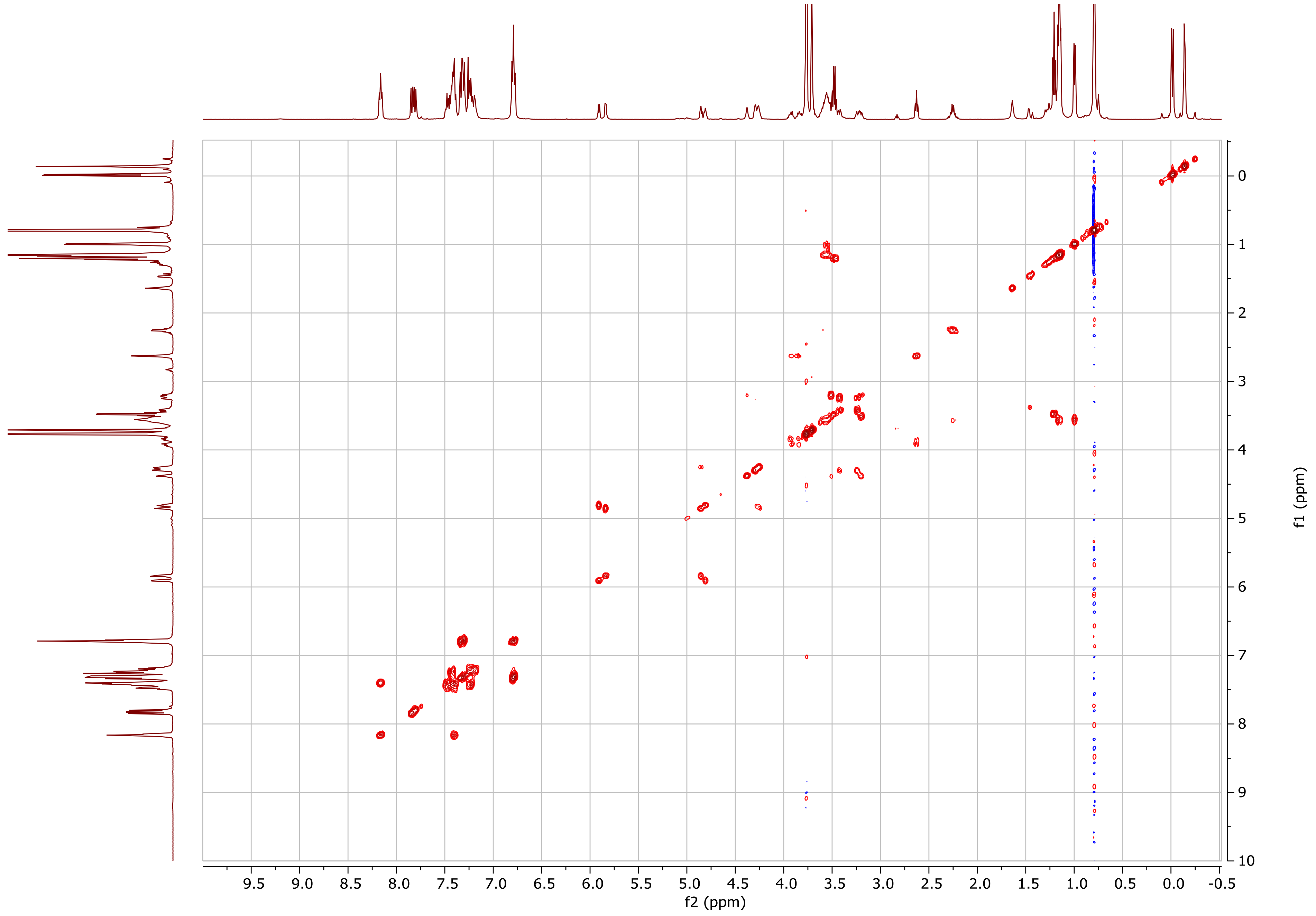

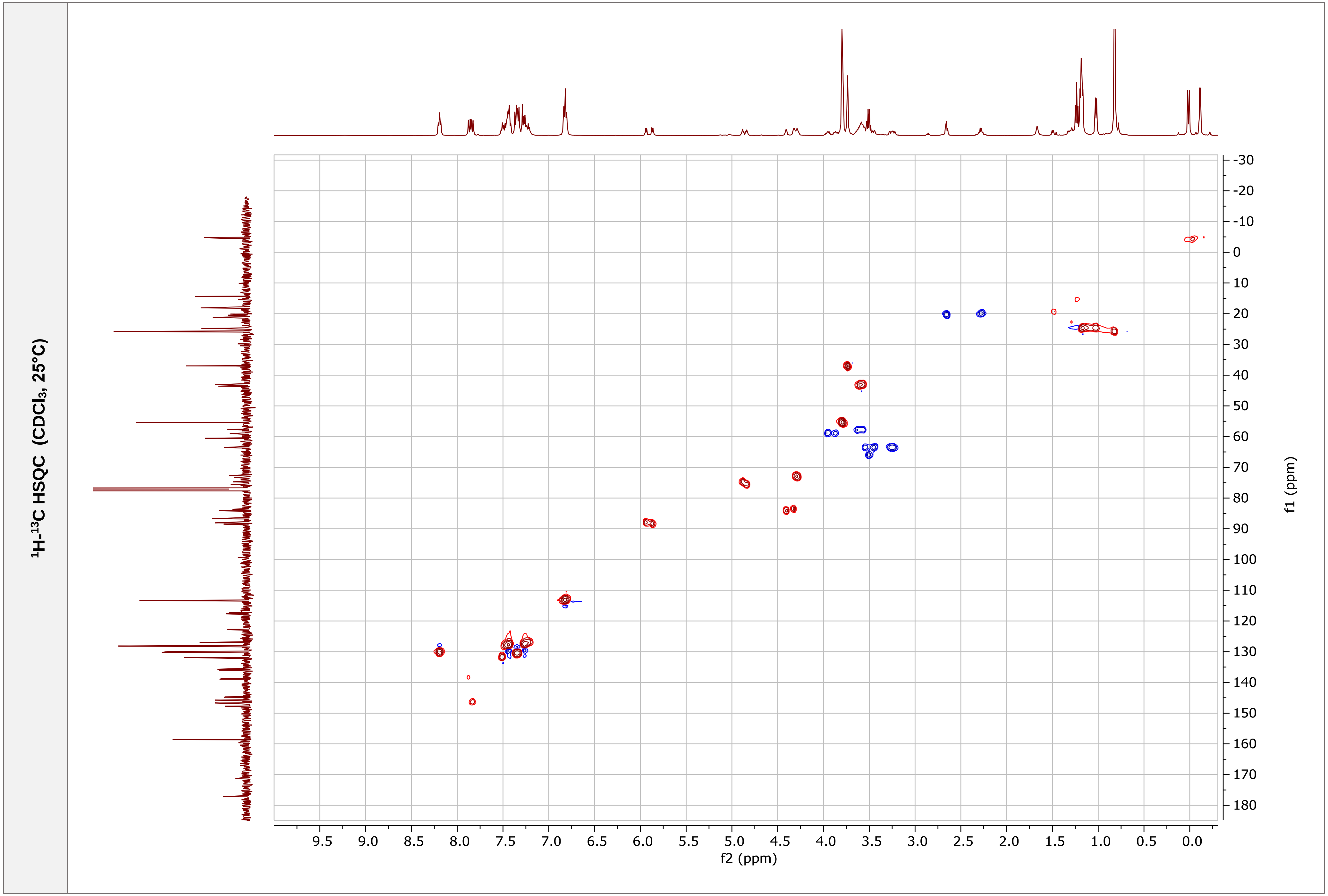
^{1}H-^{13}C HSQC (CDCl$_3$, 25°C)
f2 (ppm)
f1 (ppm)

^1H-^{31}P HSQC (CDCl$_3$, 25°C)

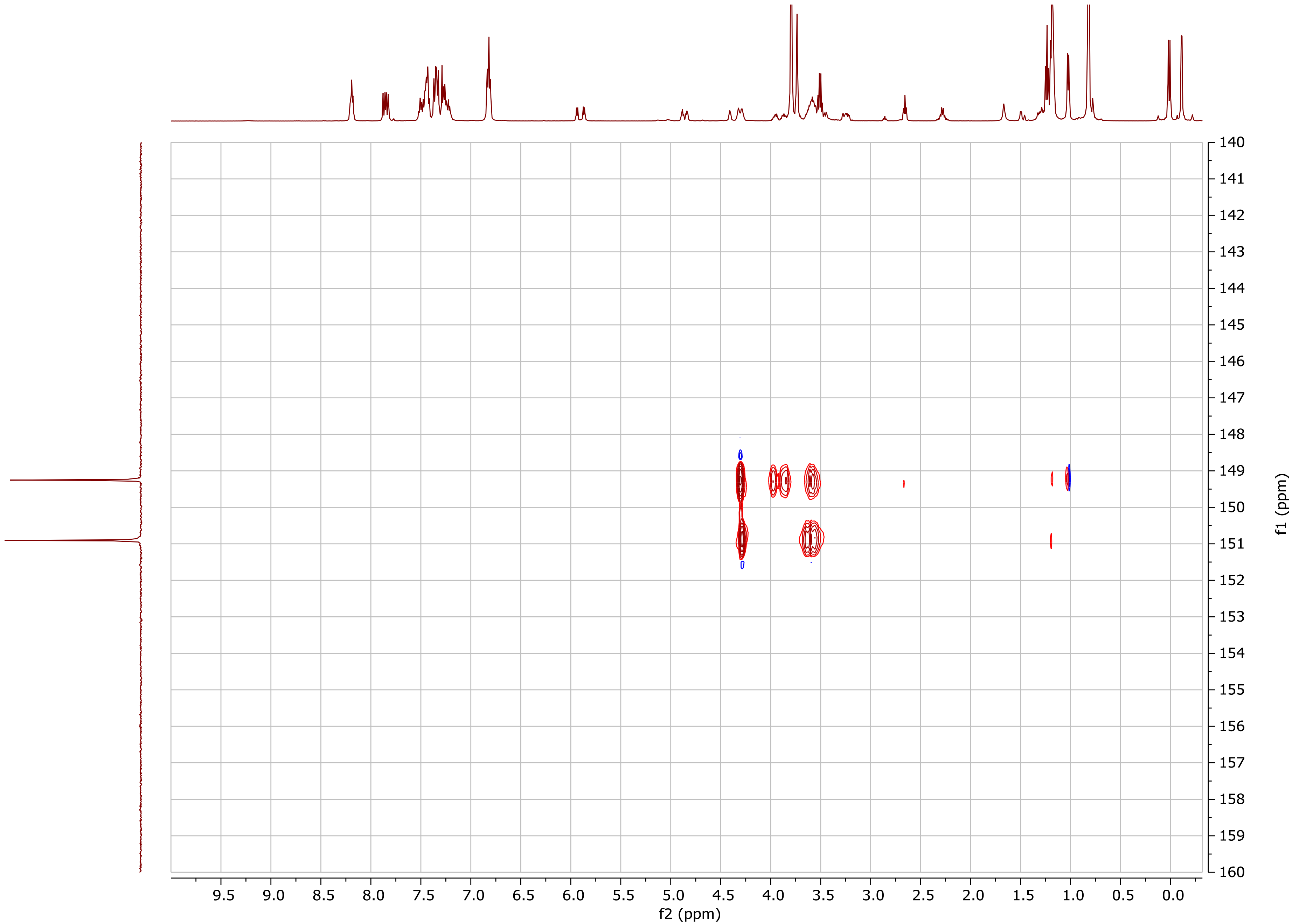

(3a) *N*6-(*N*-phenylcarbamoyl)adenosine phosphoramidite (5′-*O*-DMT-2′-O-TBDMS-PhNHCO^{6}A)

MS (+) ESI
(Calc. $[M+H]^+$ $C_{53}H_{68}N_8O_8PSi^+$ 1003.46615)

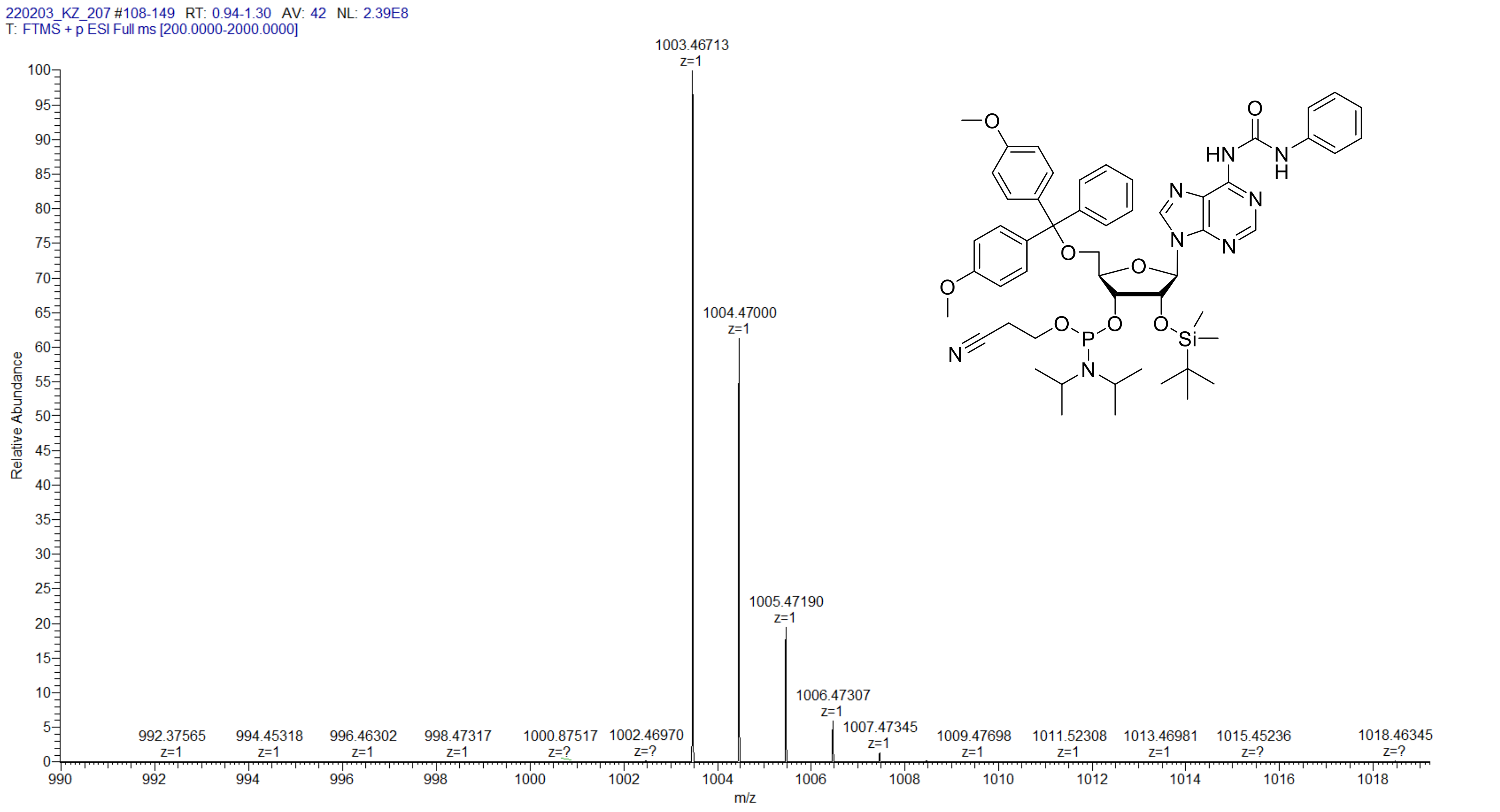

^1H NMR (500 MHz, $CDCl_3$, 25°C)

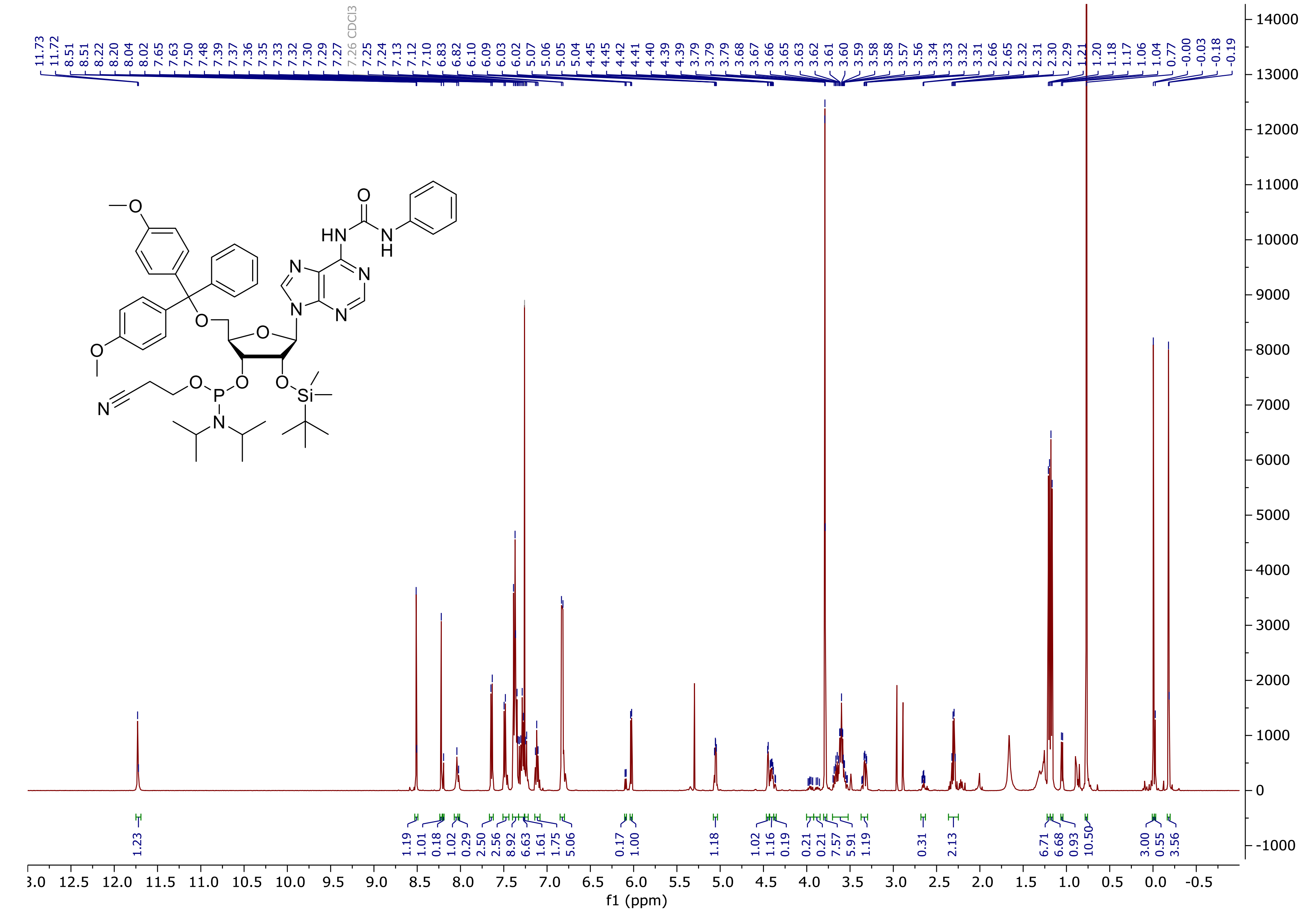

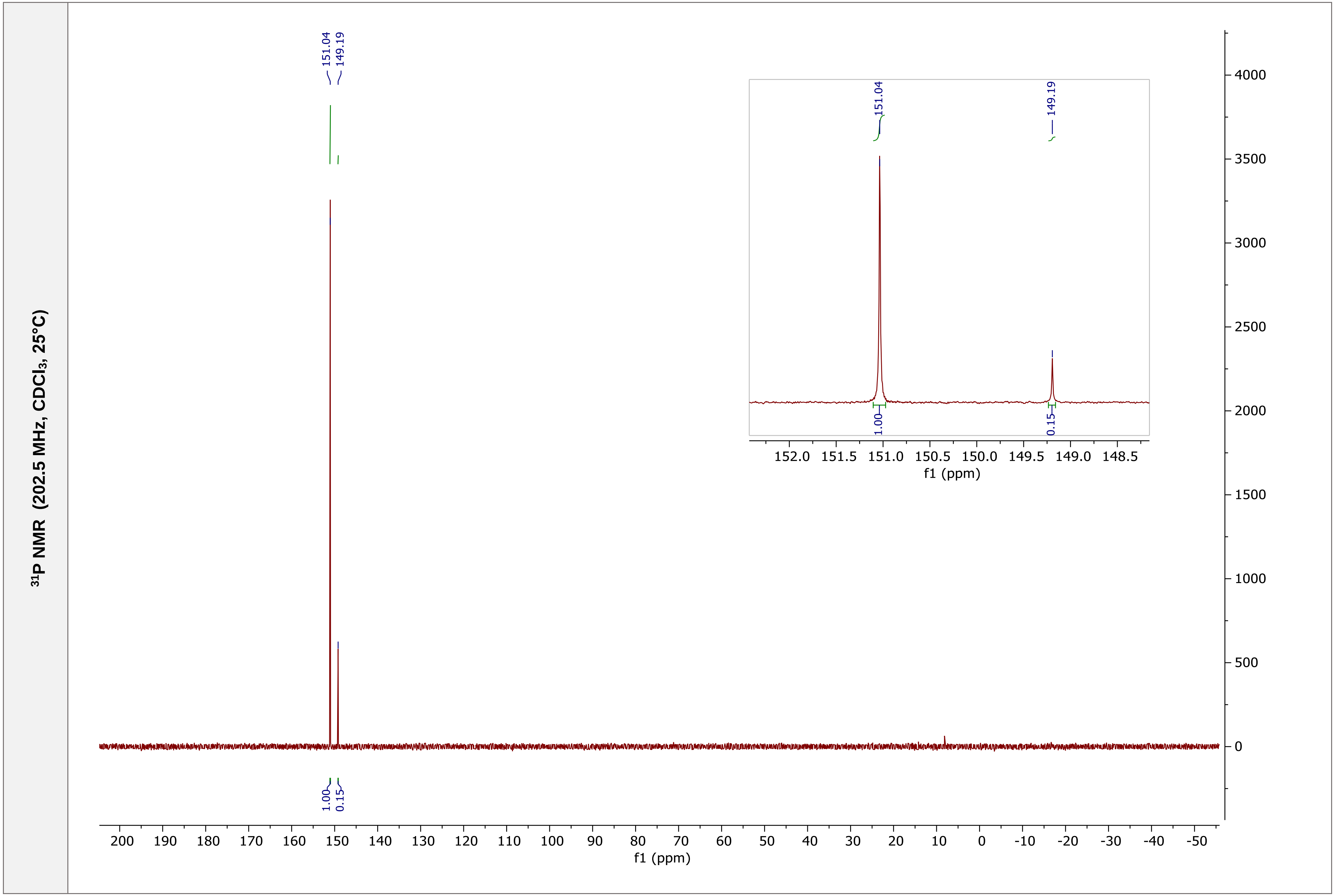

³¹P NMR (202.5 MHz, CDCl₃, 25°C)
151.04
149.19
1.00
0.15
f1 (ppm)

COSY NMR (CDCl$_3$, 25°C)

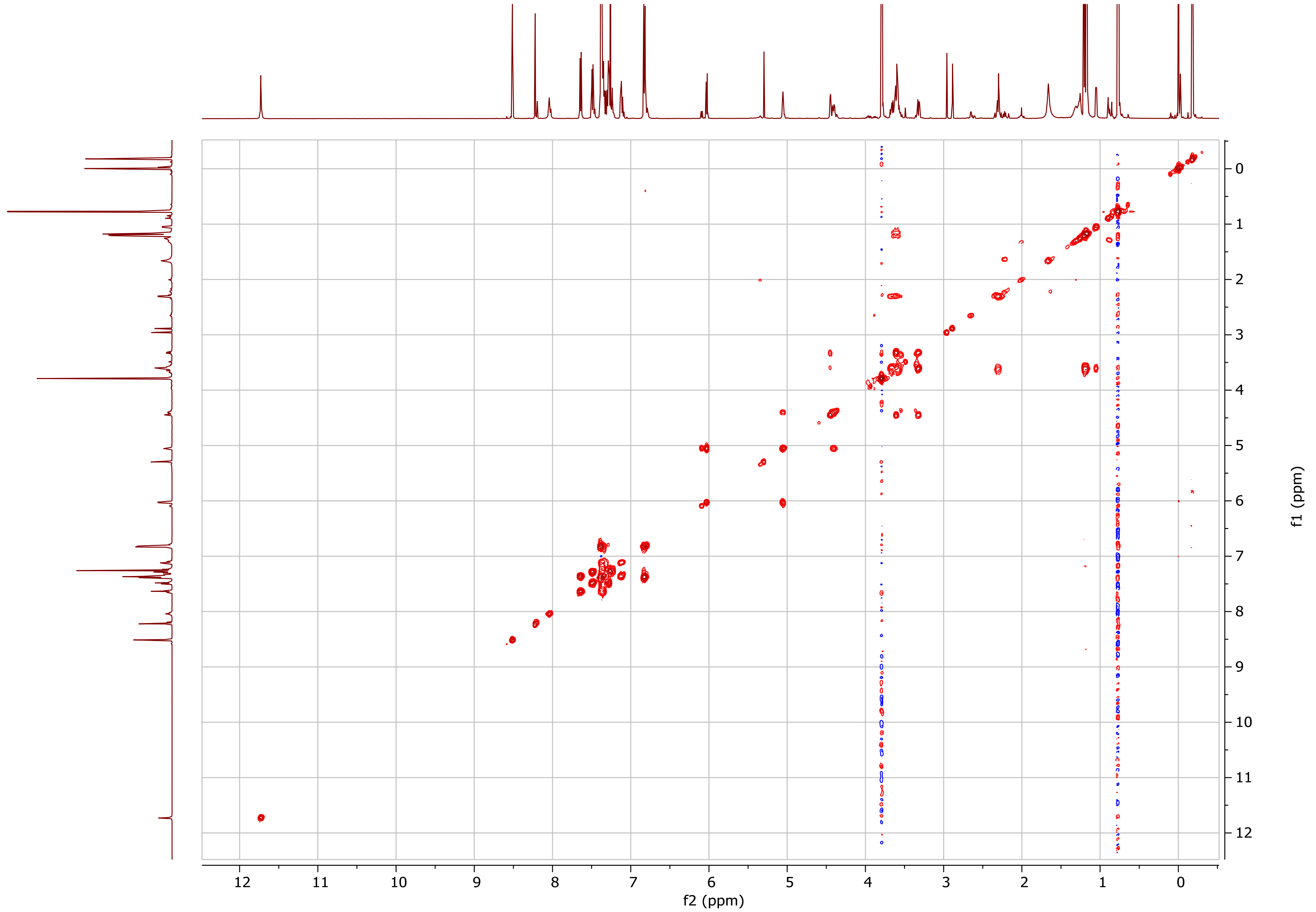

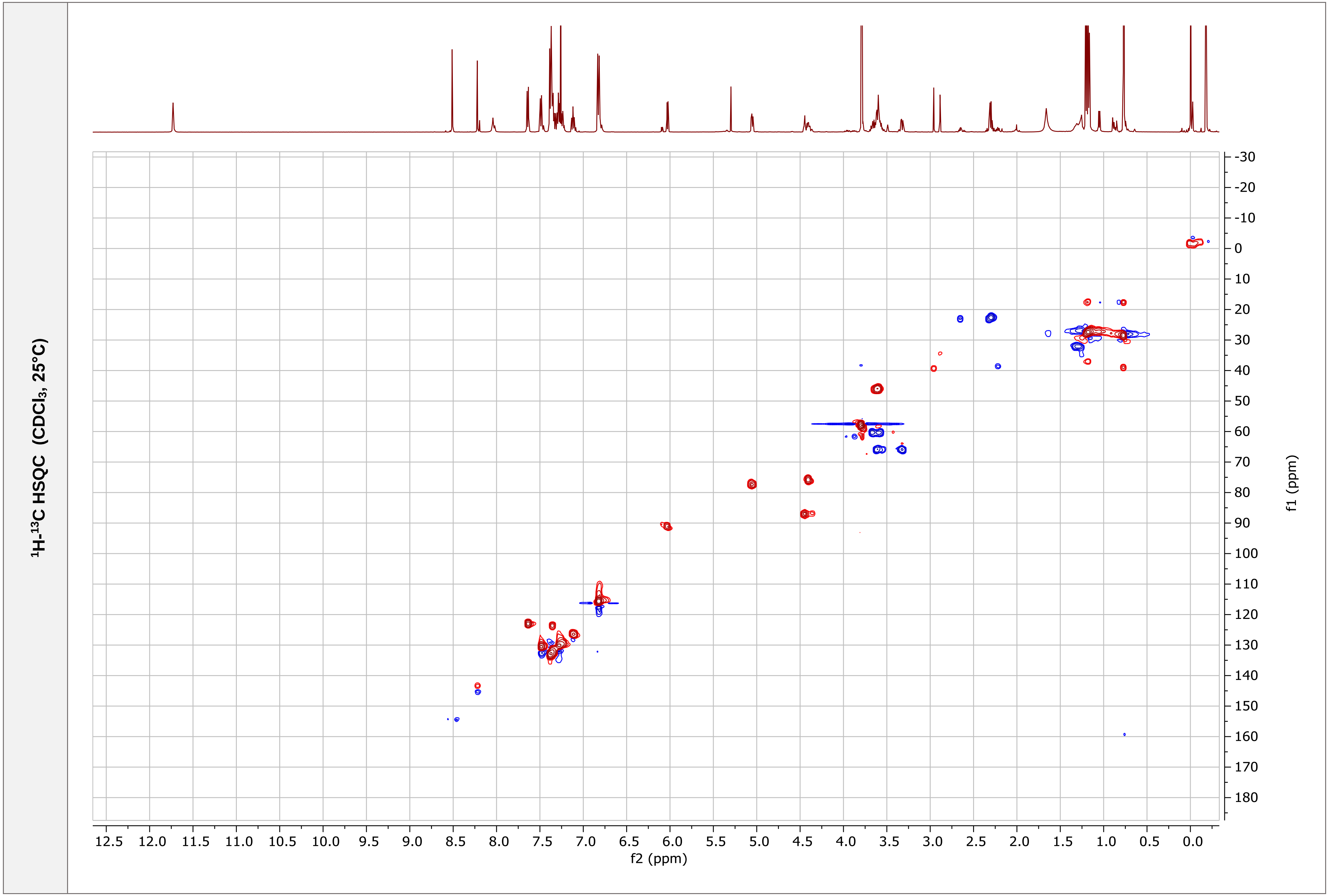
^{1}H-^{13}C HSQC (CDCl$_3$, 25°C)
f2 (ppm)
f1 (ppm)

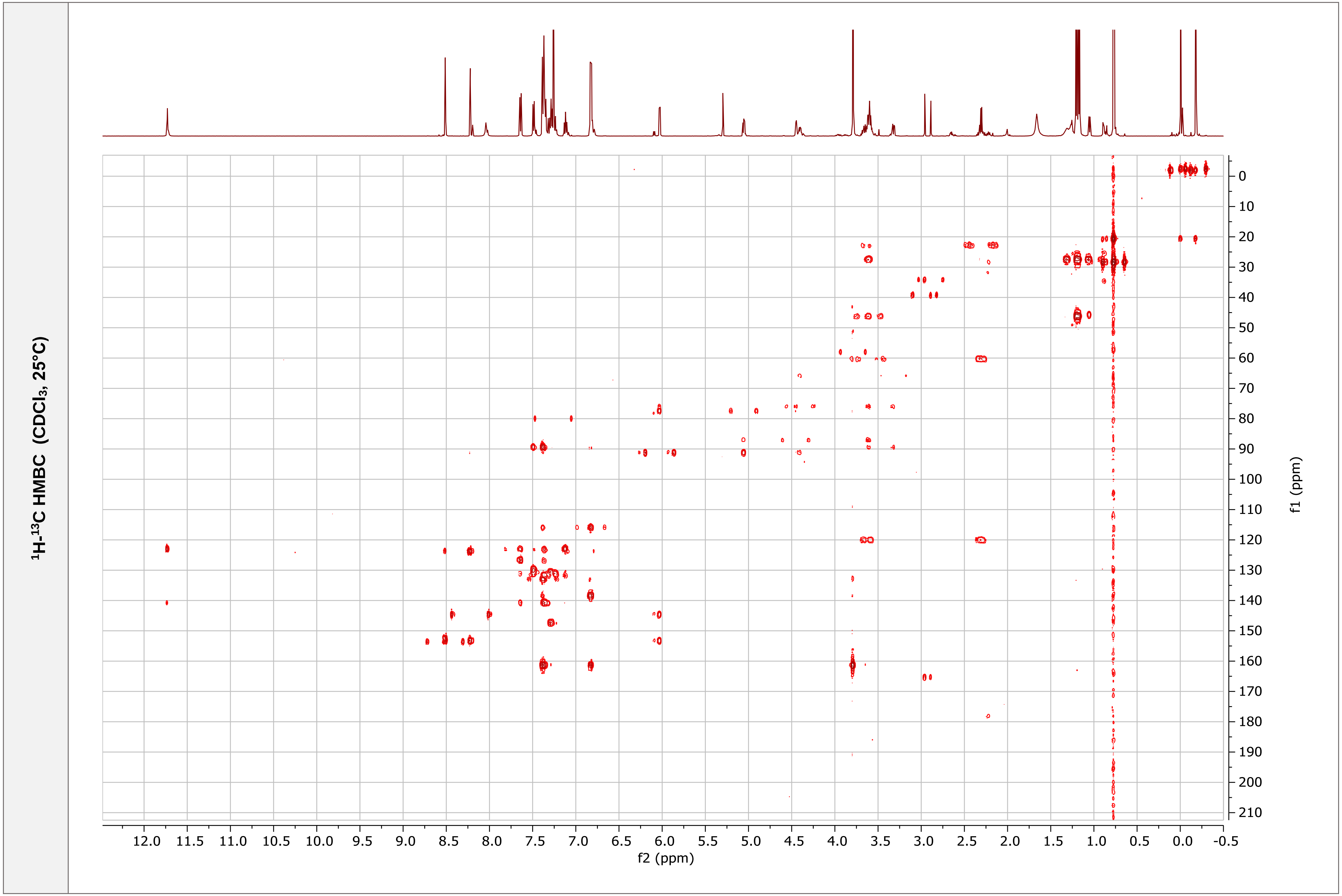
^1H-^{13}C HMBC (CDCl$_3$, 25°C)
f2 (ppm)
f1 (ppm)

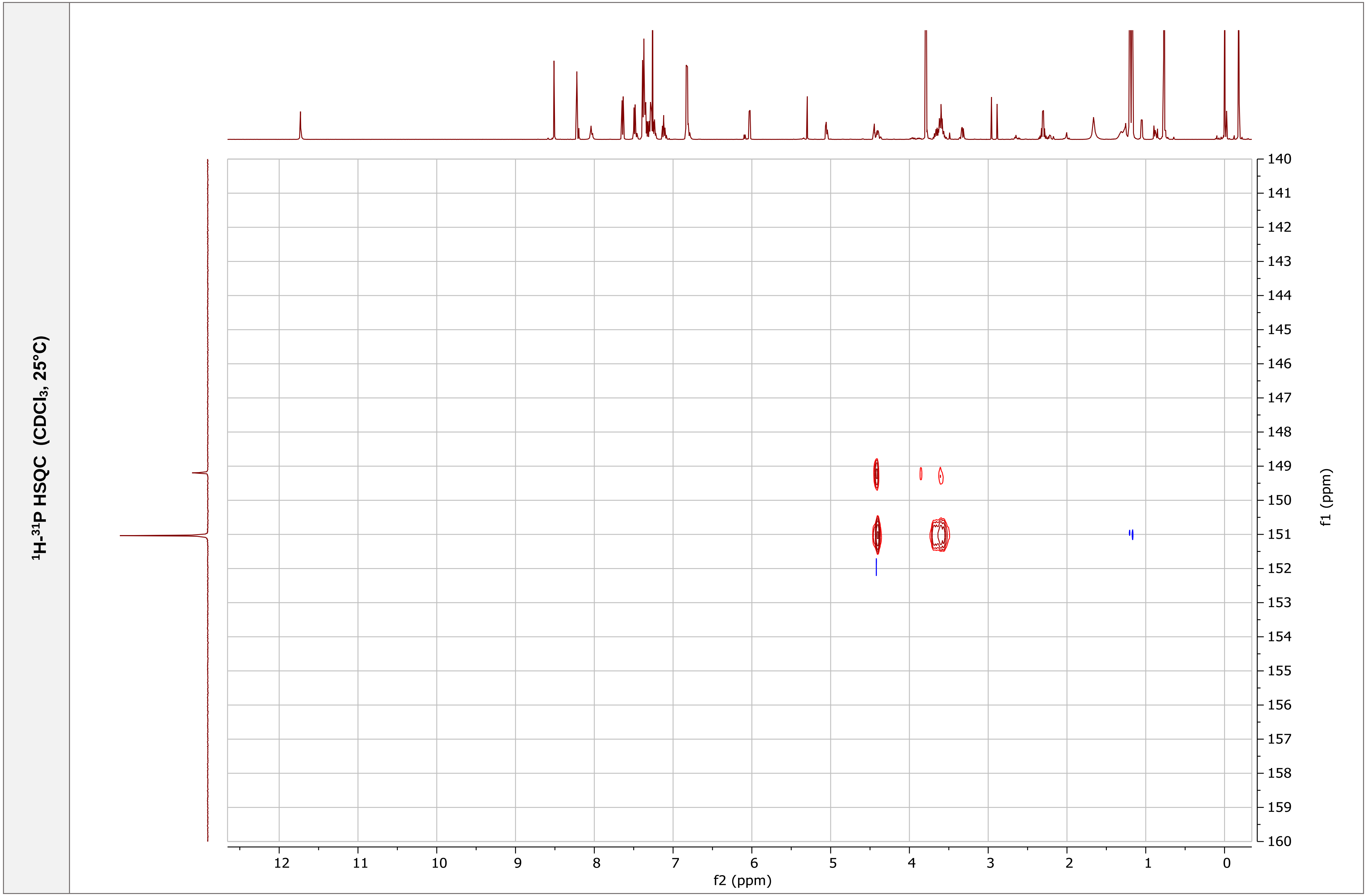

^{1}H-^{31}P HSQC (CDCl$_3$, 25°C)
f1 (ppm)
f2 (ppm)

(3b) *N*6-glycinylcarbamoyladenosine phosphoramidite (5′-*O*-DMT-2′-*O*-TBDMS-g^{6}A)

MS (+) ESI
(Calc. $[M+H]^+$ $C_{51}H_{70}N_8O_{10}PSi^+$ 1013.47163)

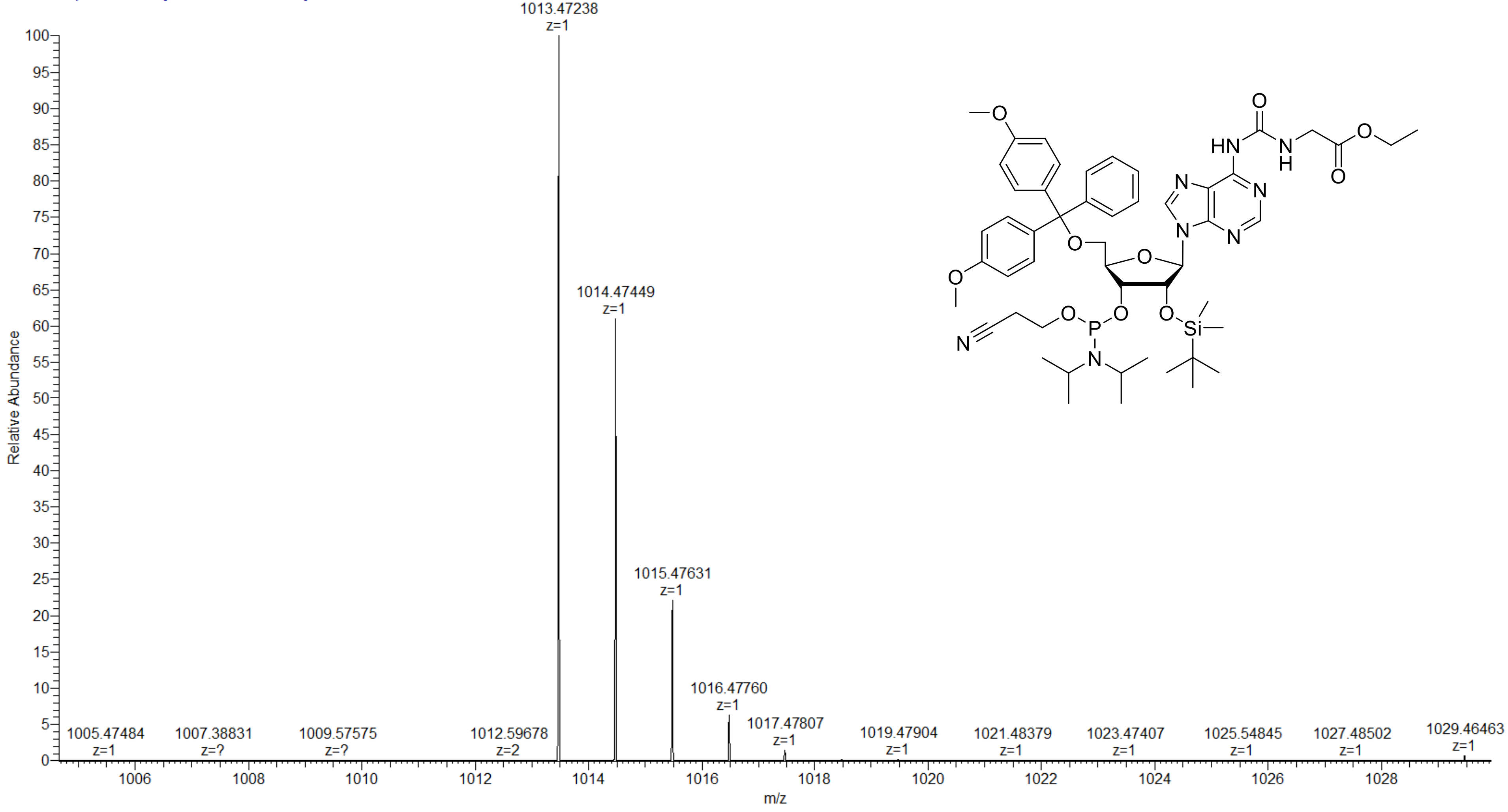

^{1}H NMR (500 MHz, CDCl$_3$, 25°C)

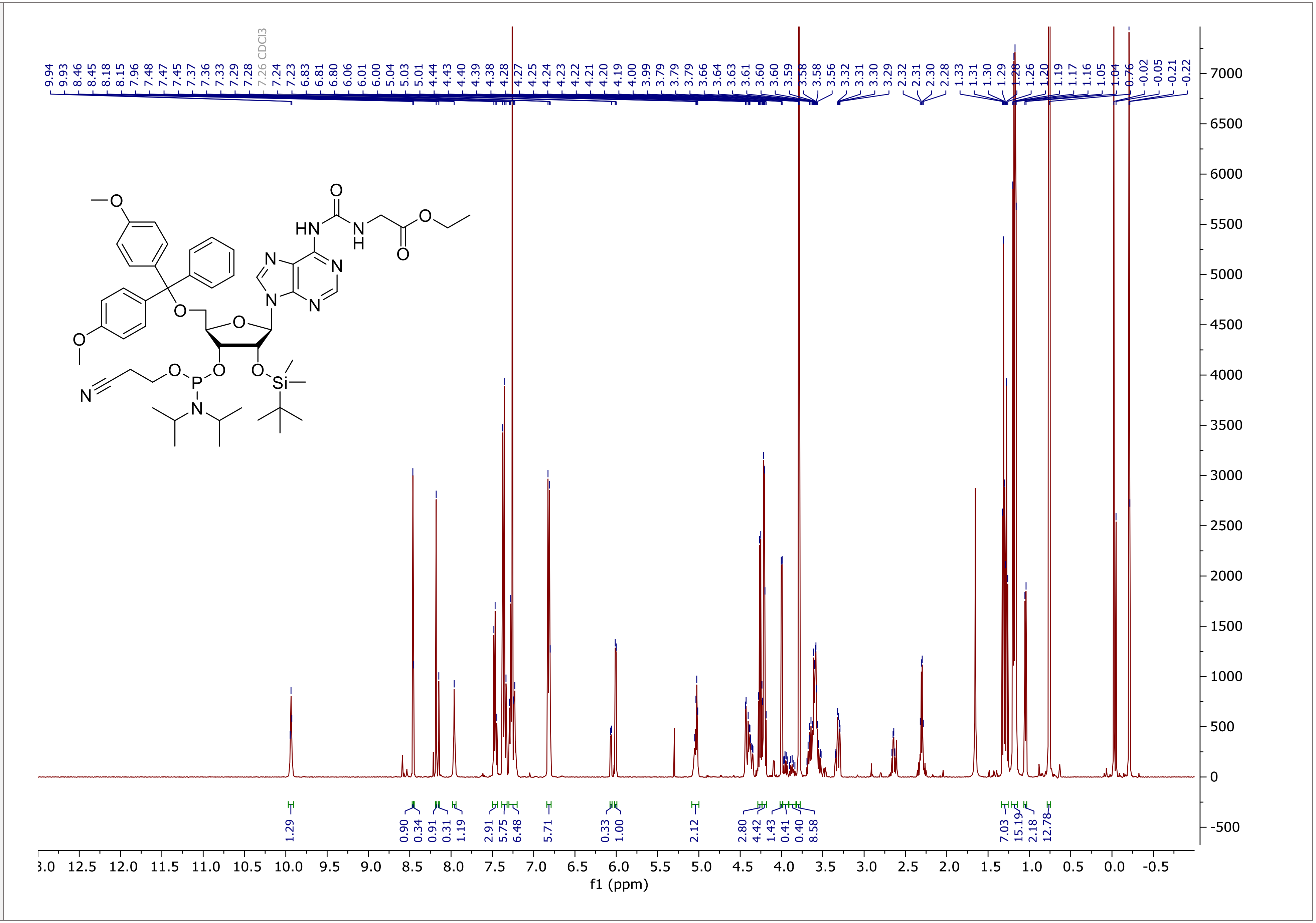

$^{13}C\{^1H\}$ NMR (126 MHz, $CDCl_3$, 25°C)

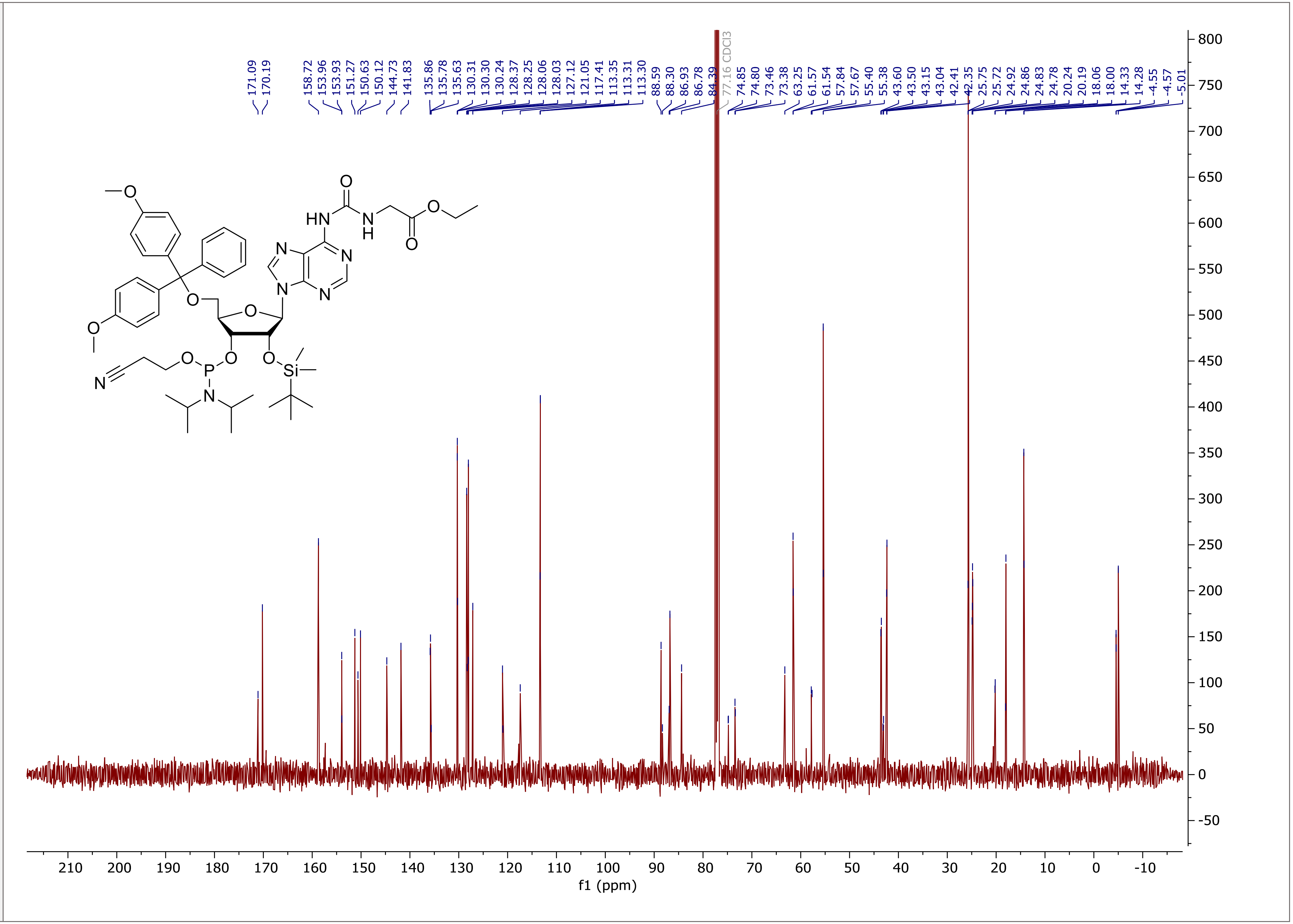

^{31}P NMR (202.5 MHz, CDCl$_3$, 25°C)

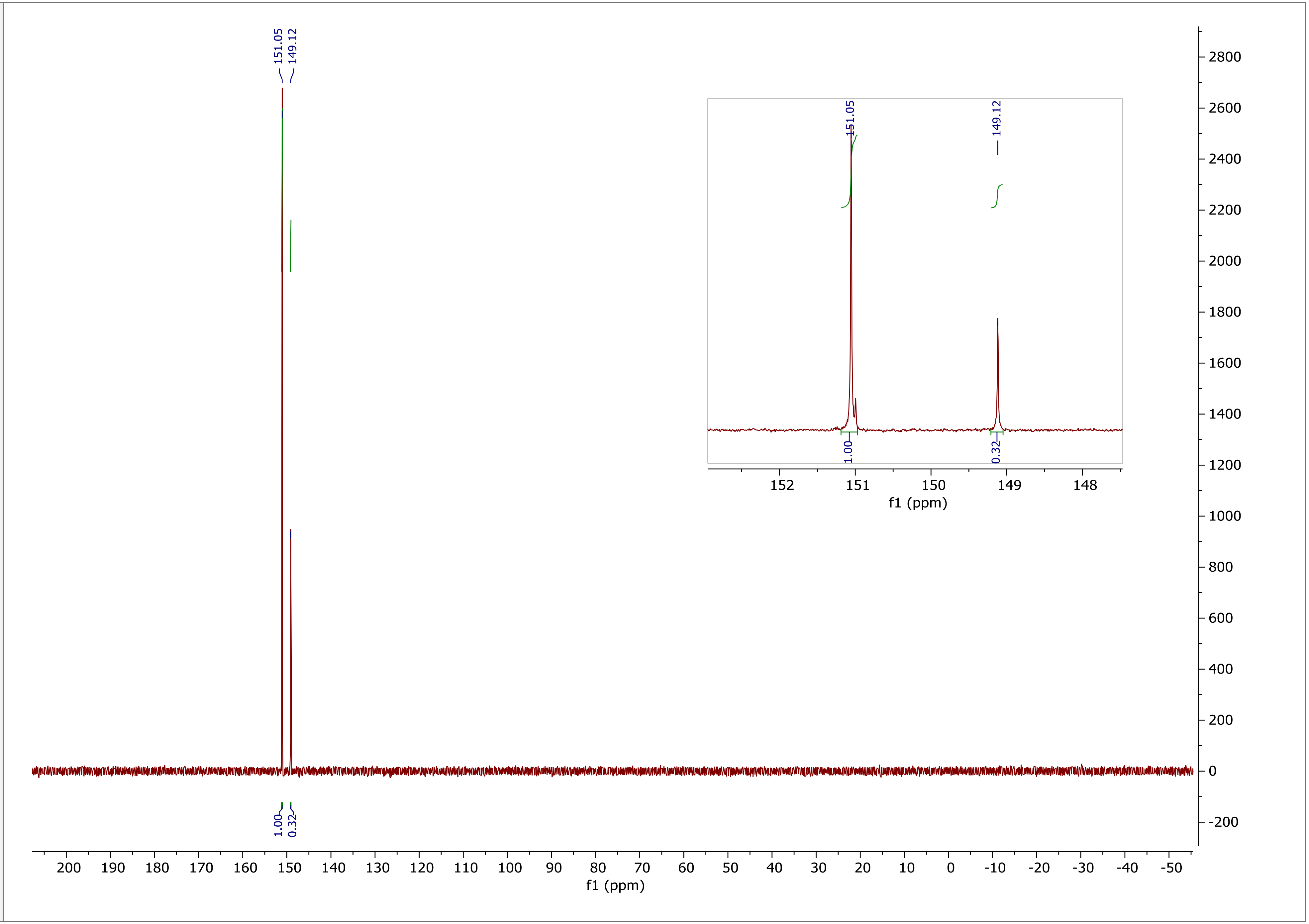

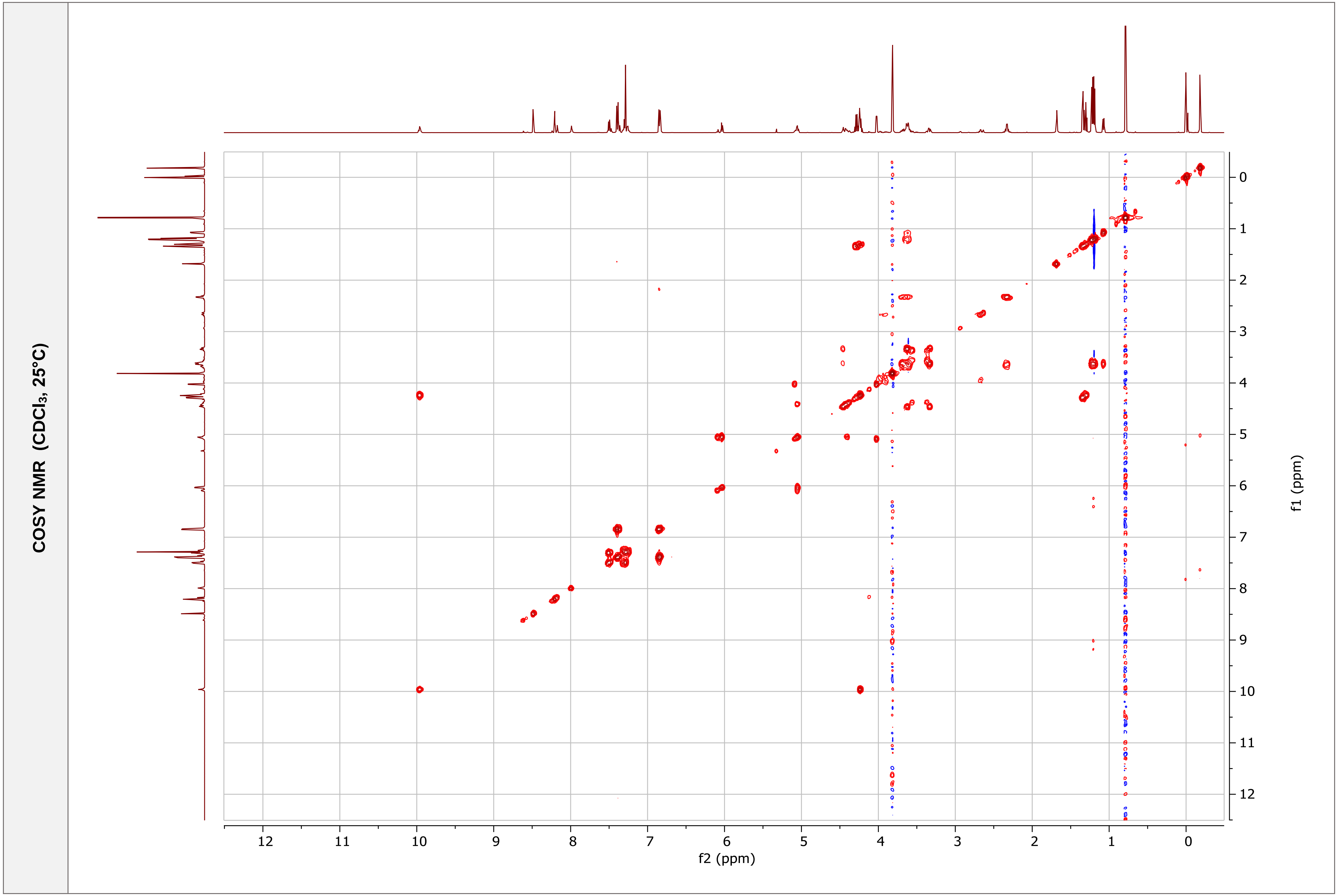
COSY NMR (CDCl$_3$, 25°C)
12
11
10
9
8
7
6
5
4
3
2
1
0
f2 (ppm)
f1 (ppm)

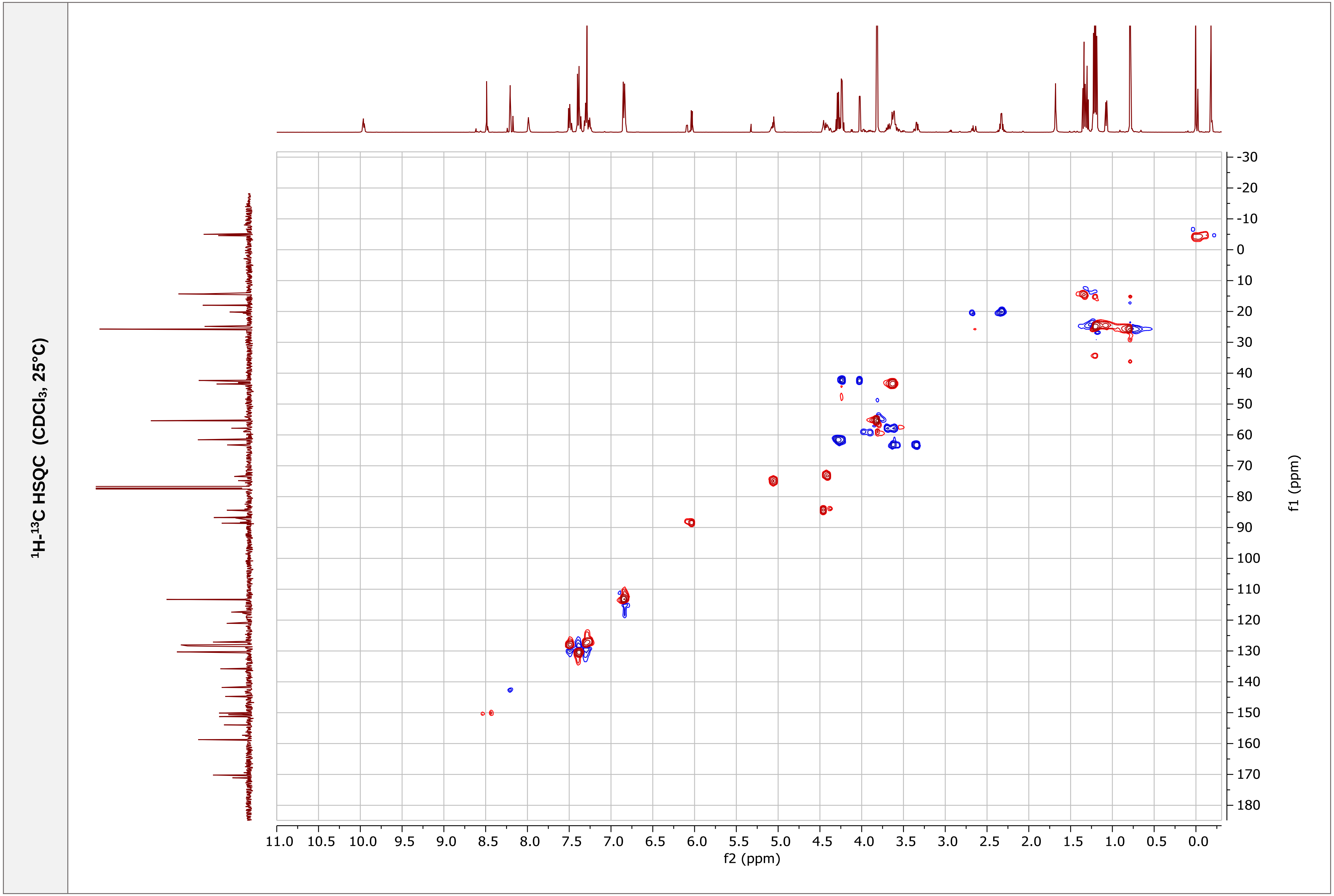
¹H-¹³C HSQC (CDCl₃, 25°C)
f2 (ppm)
f1 (ppm)

^1H-^{13}C HMBC (CDCl$_3$, 25°C)

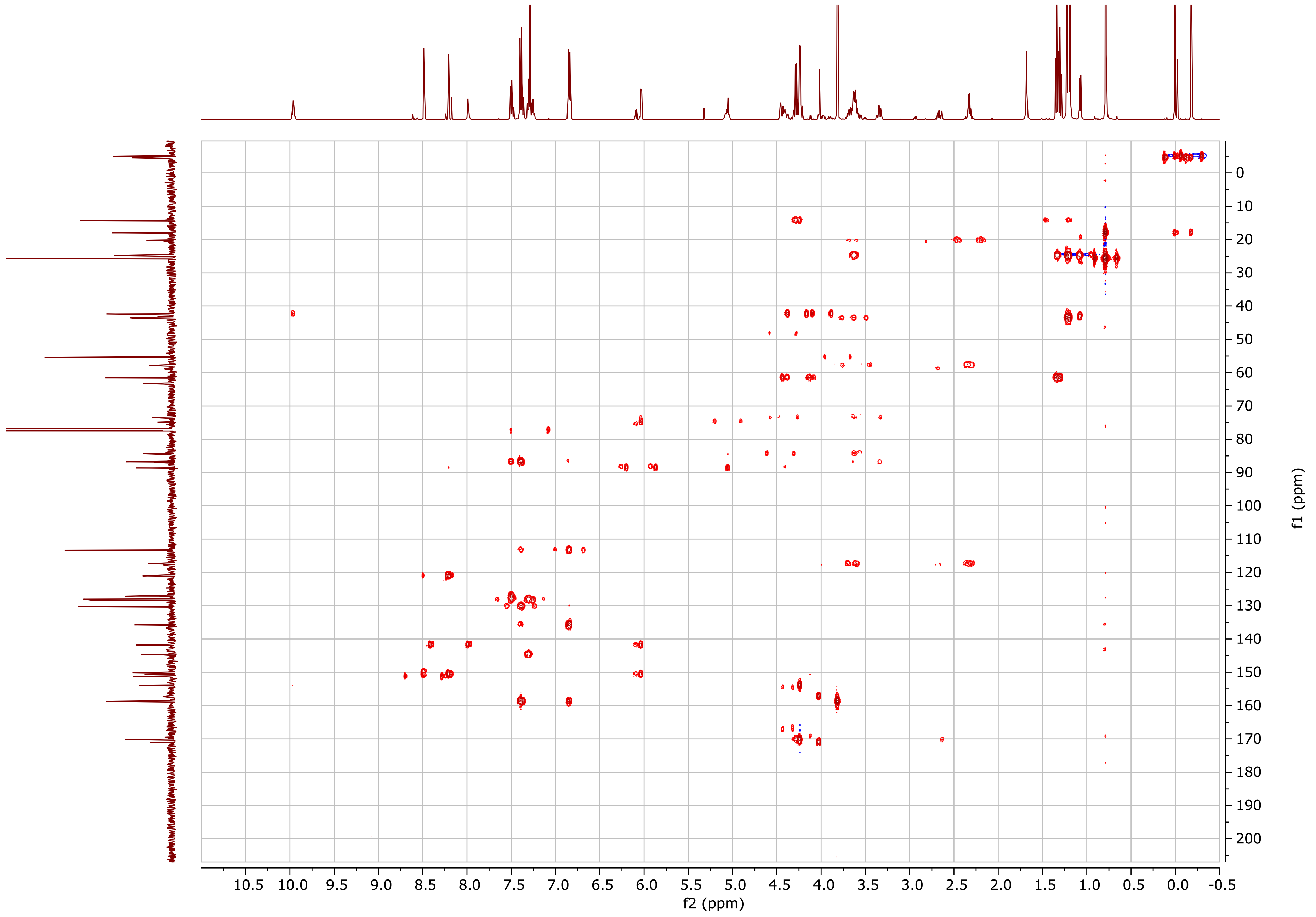

(4) *N*6-glycinylcarbamoyl-*N*6-methyladenosine phosphoramidite (5′-*O*-DMT-2′-*O*-TBDMS-g^{6}m^{6}A)

MS (+) ESI

(Calc. $[M+H]^+$ $C_{52}H_{72}N_8O_{10}PSi^+$ 1027.48728)

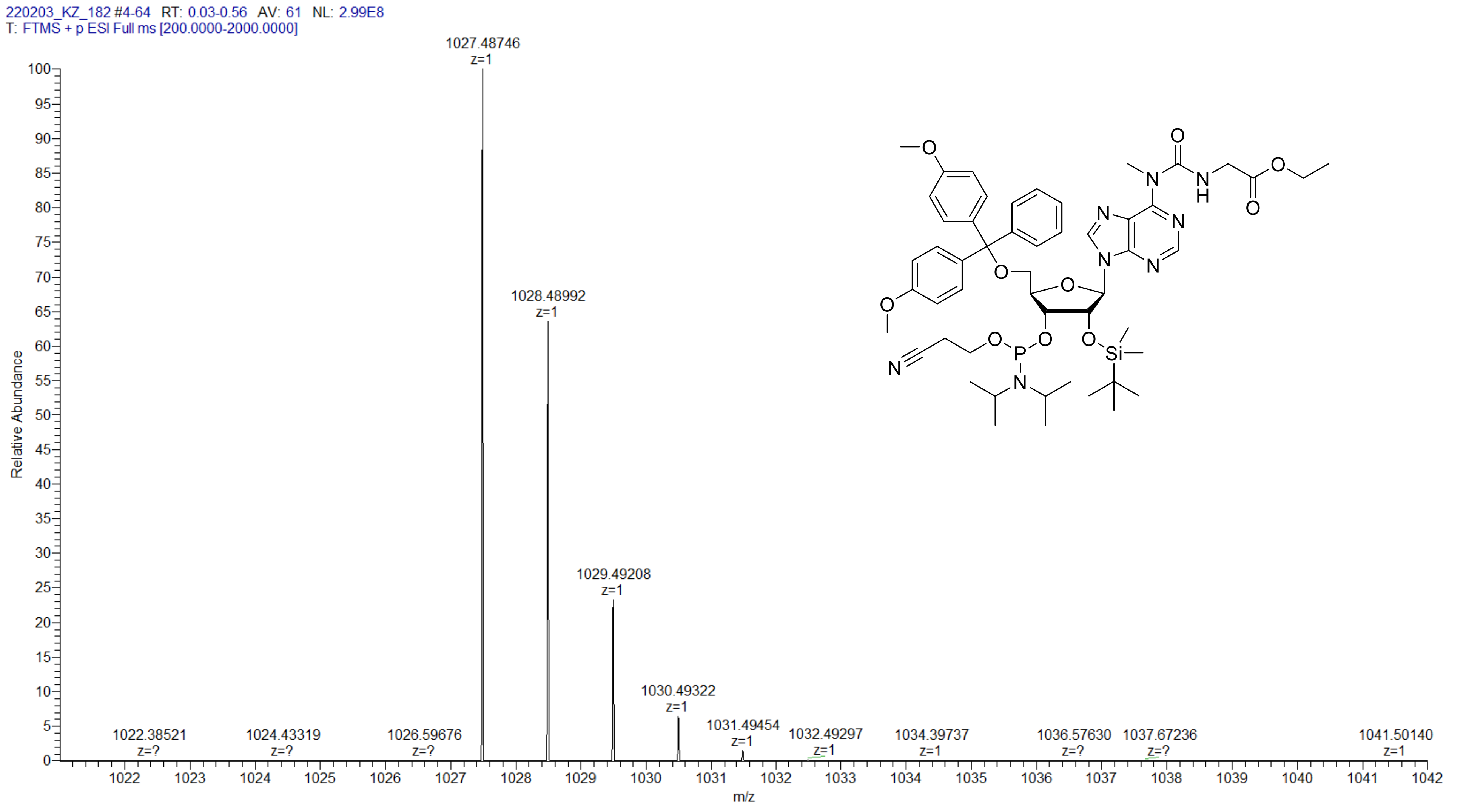

^1H NMR (500 MHz, $CDCl_3$, 25°C)

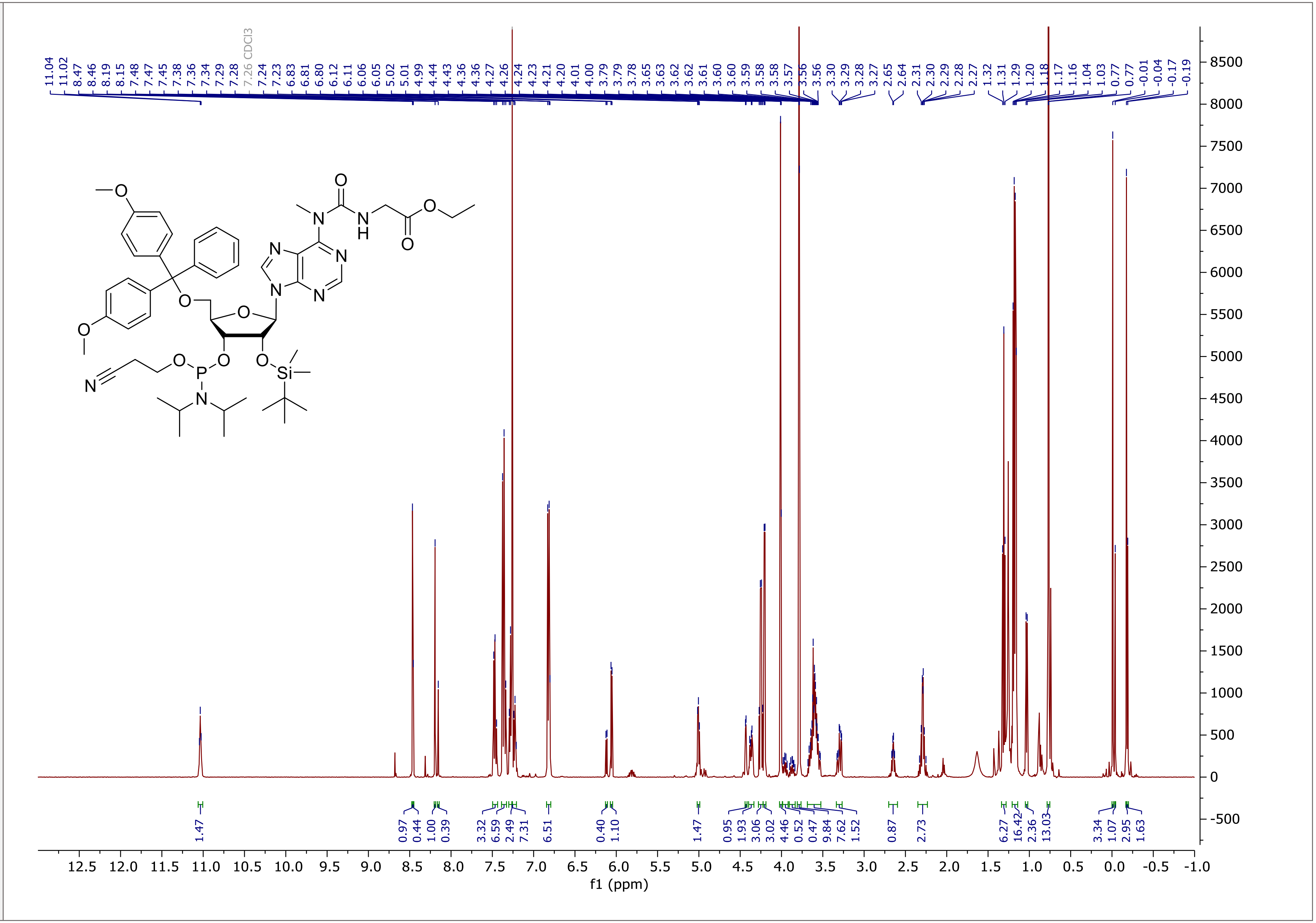

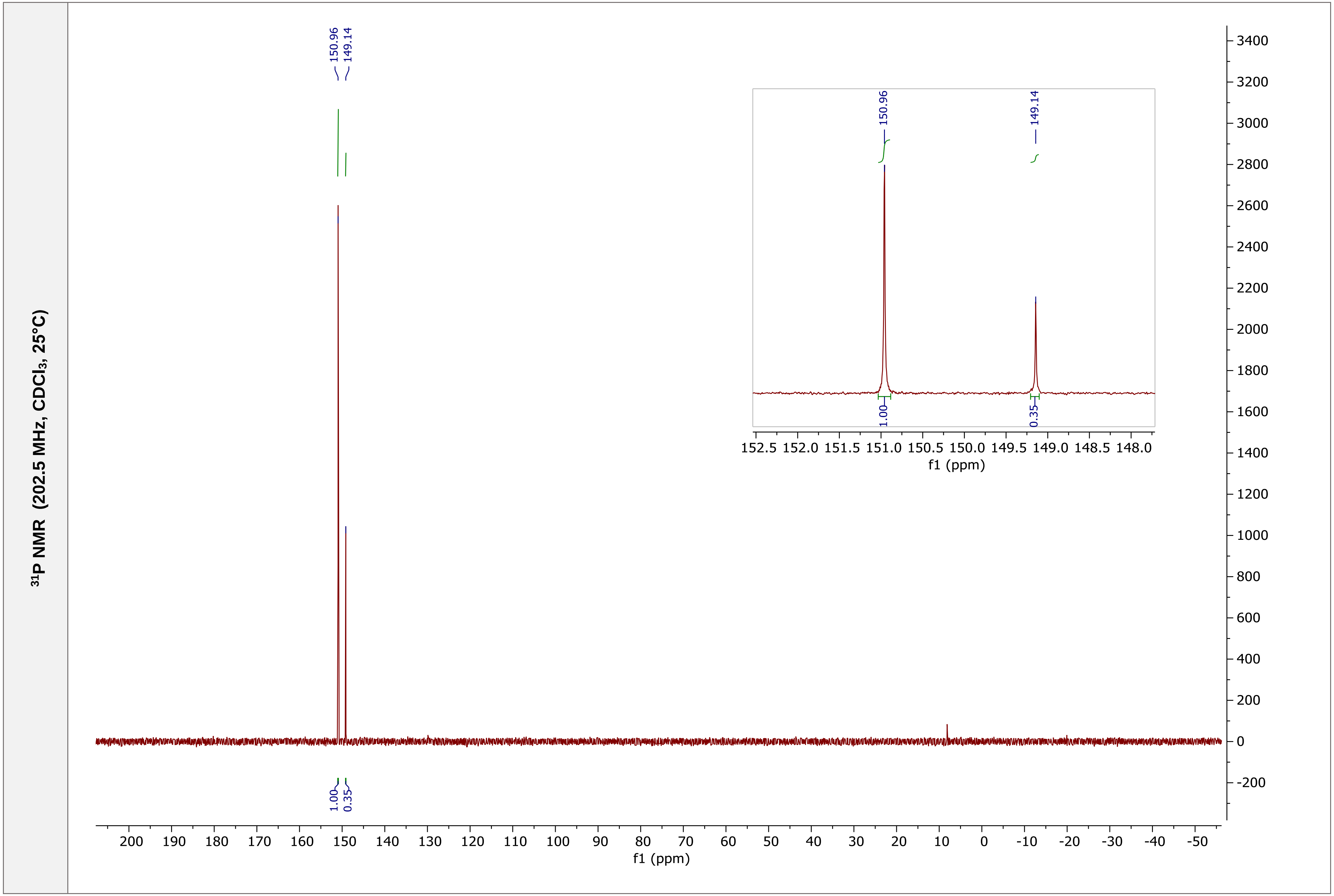

31P NMR (202.5 MHz, CDCl3, 25°C)
150.96
149.14
1.00
0.35
f1 (ppm)

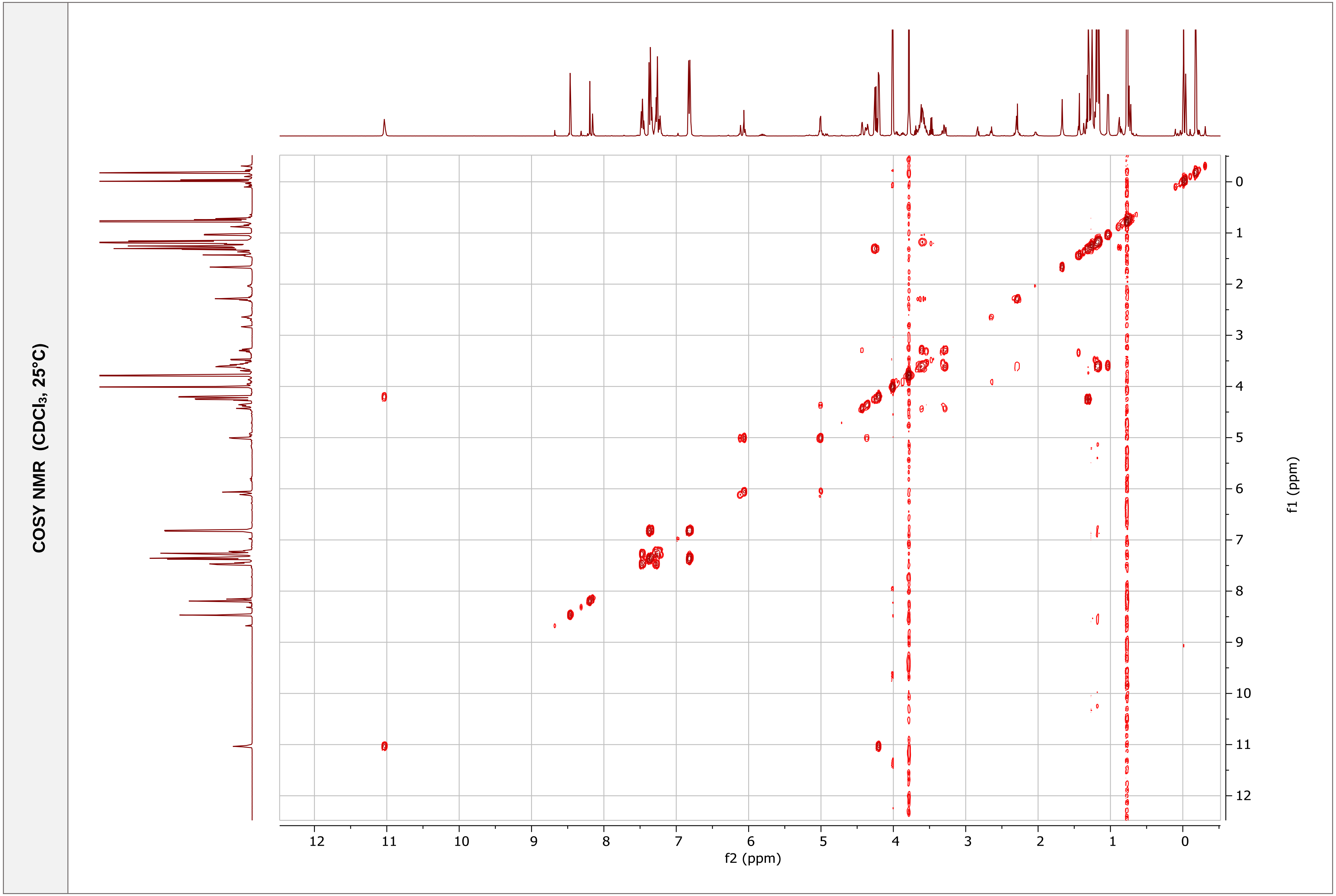
COSY NMR (CDCl3, 25°C)
f2 (ppm)
f1 (ppm)
12
11
10
9
8
7
6
5
4
3
2
1
0

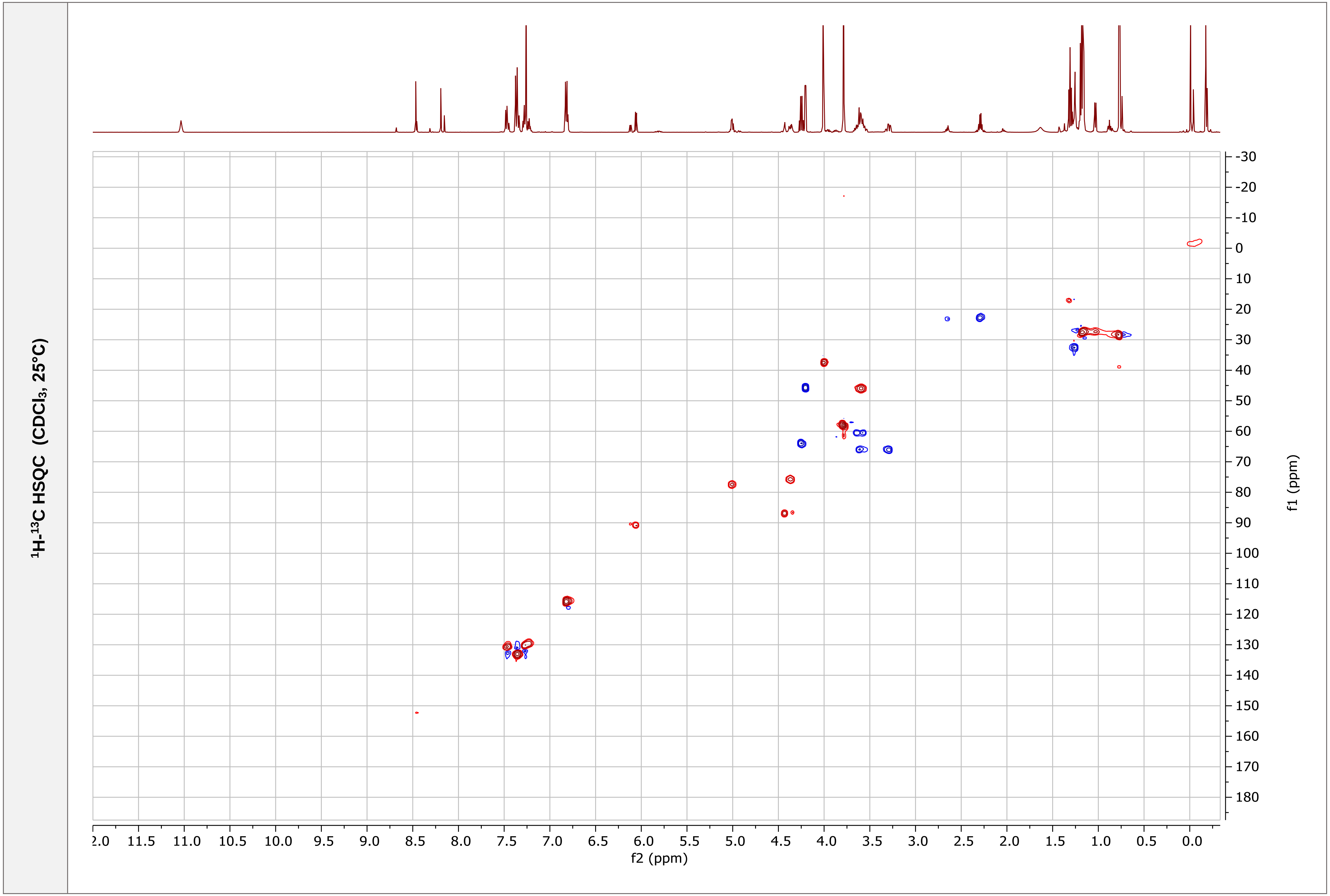
^{1}H-^{13}C HSQC (CDCl$_3$, 25°C)
f2 (ppm)
f1 (ppm)

^1H-^{13}C HMBC (CDCl$_3$, 25°C)

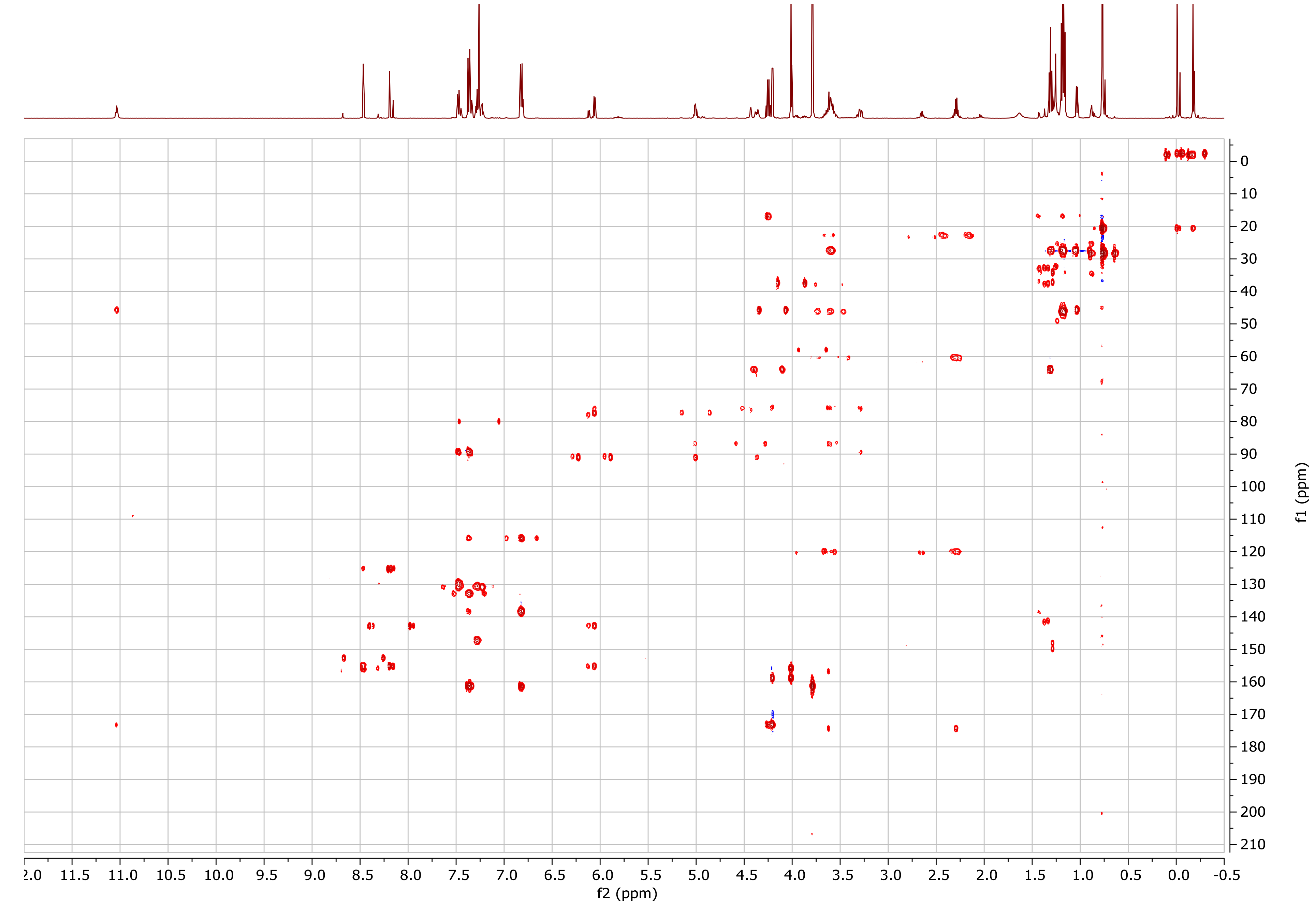

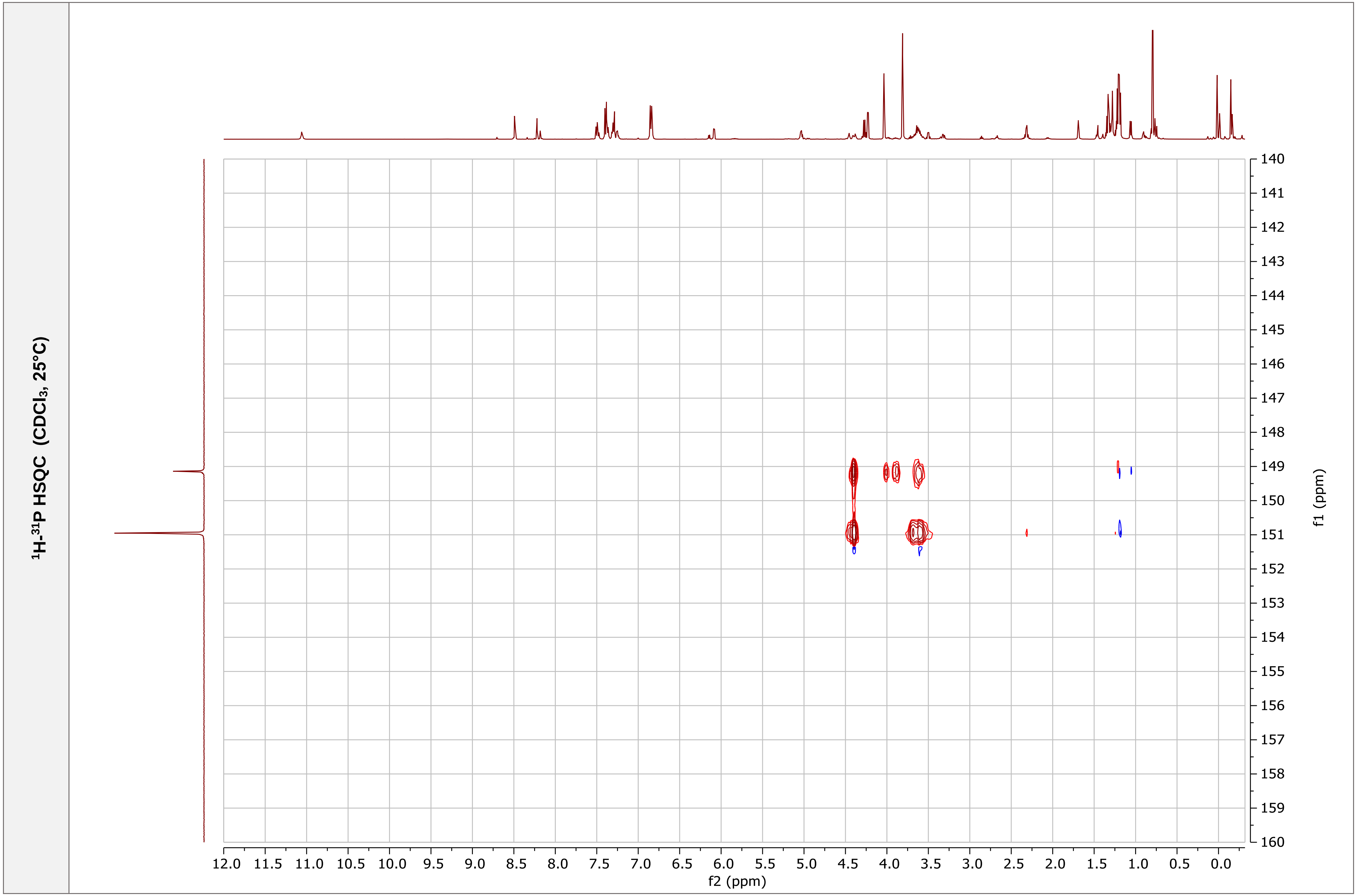

^1H-^{31}P HSQC (CDCl$_3$, 25°C)
f2 (ppm)
f1 (ppm)

(5a) *N*4-methylcytidine phosphoramidite (5′-*O*-DMT-2′-*O*-TBDMS-m^4C^{Ac})

MS (+) ESI
(Calc. $[M+H]^+$ $C_{48}H_{67}N_5O_9PSi^+$ 916.44402)

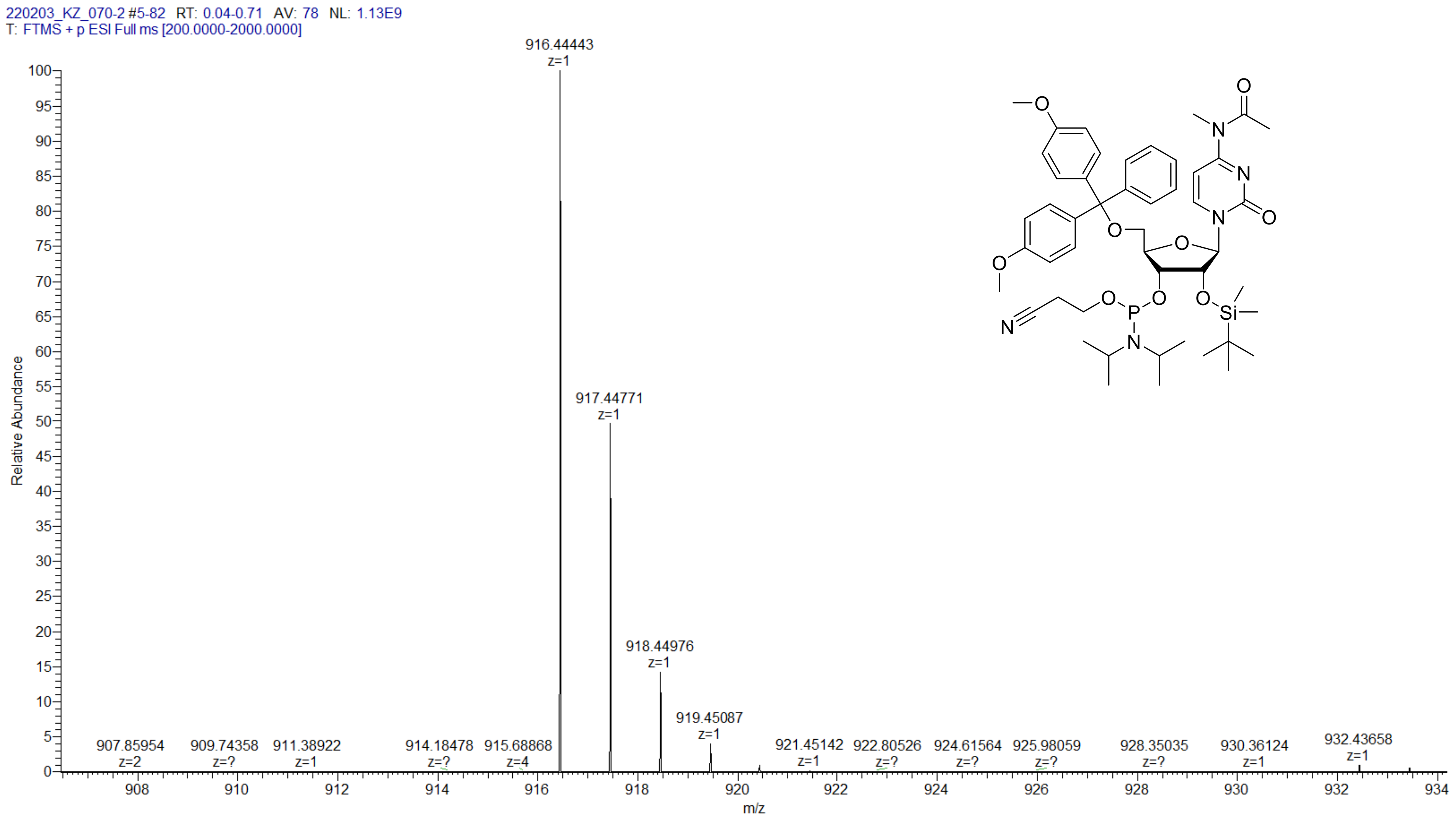

^1H NMR (500 MHz, $CDCl_3$, 25°C)

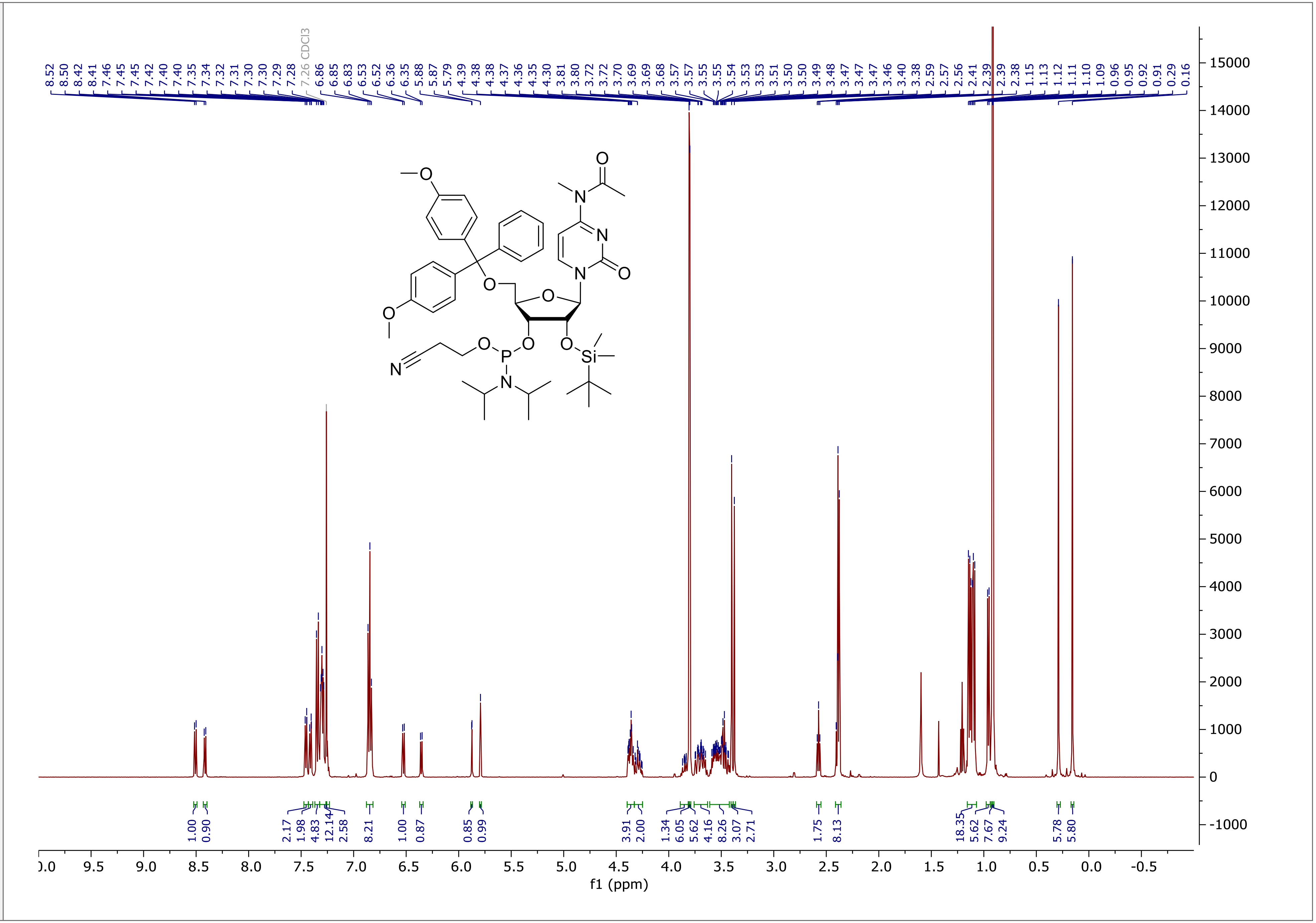

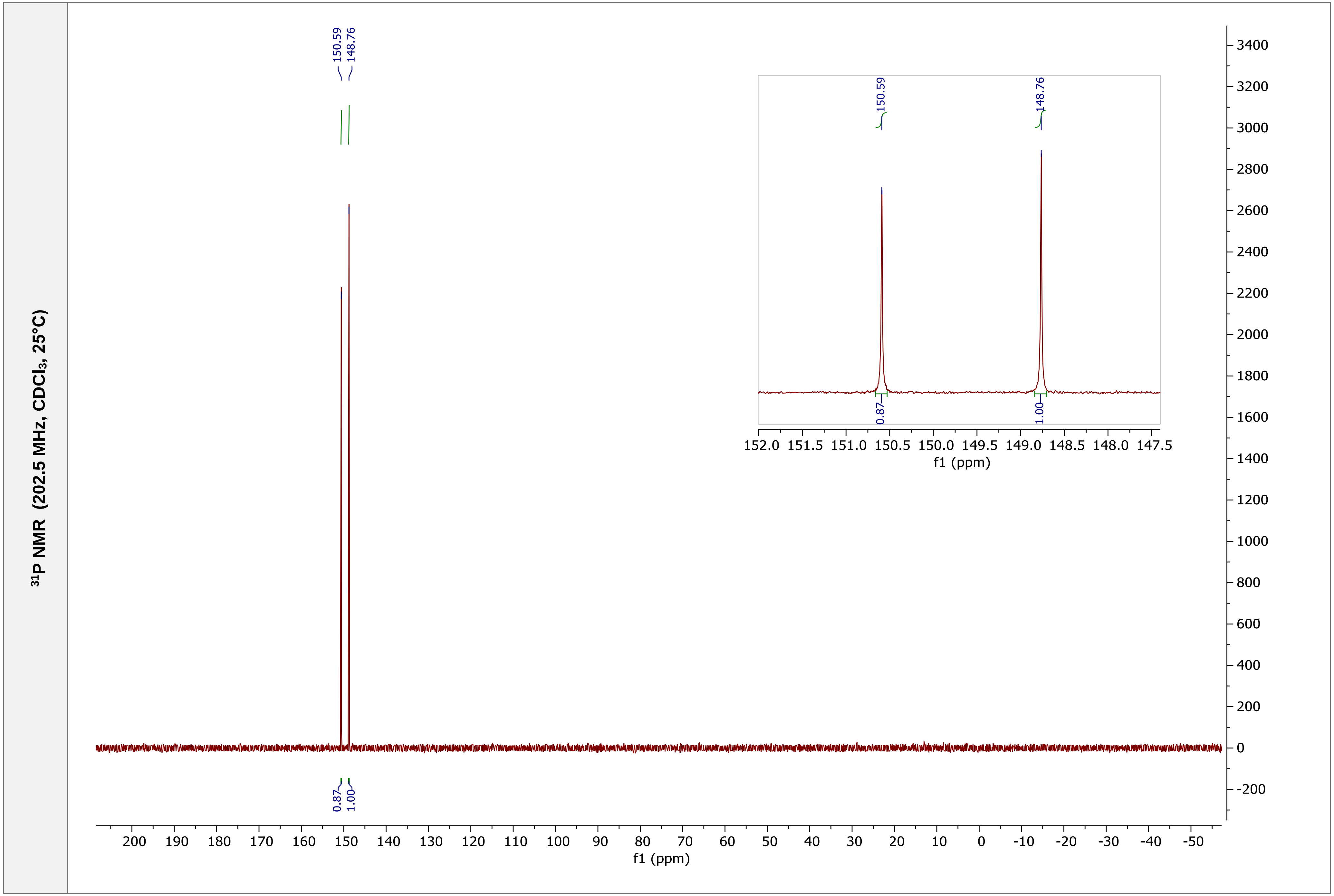

^{31}P NMR (202.5 MHz, CDCl$_3$, 25°C)
150.59
148.76
0.87
1.00
f1 (ppm)

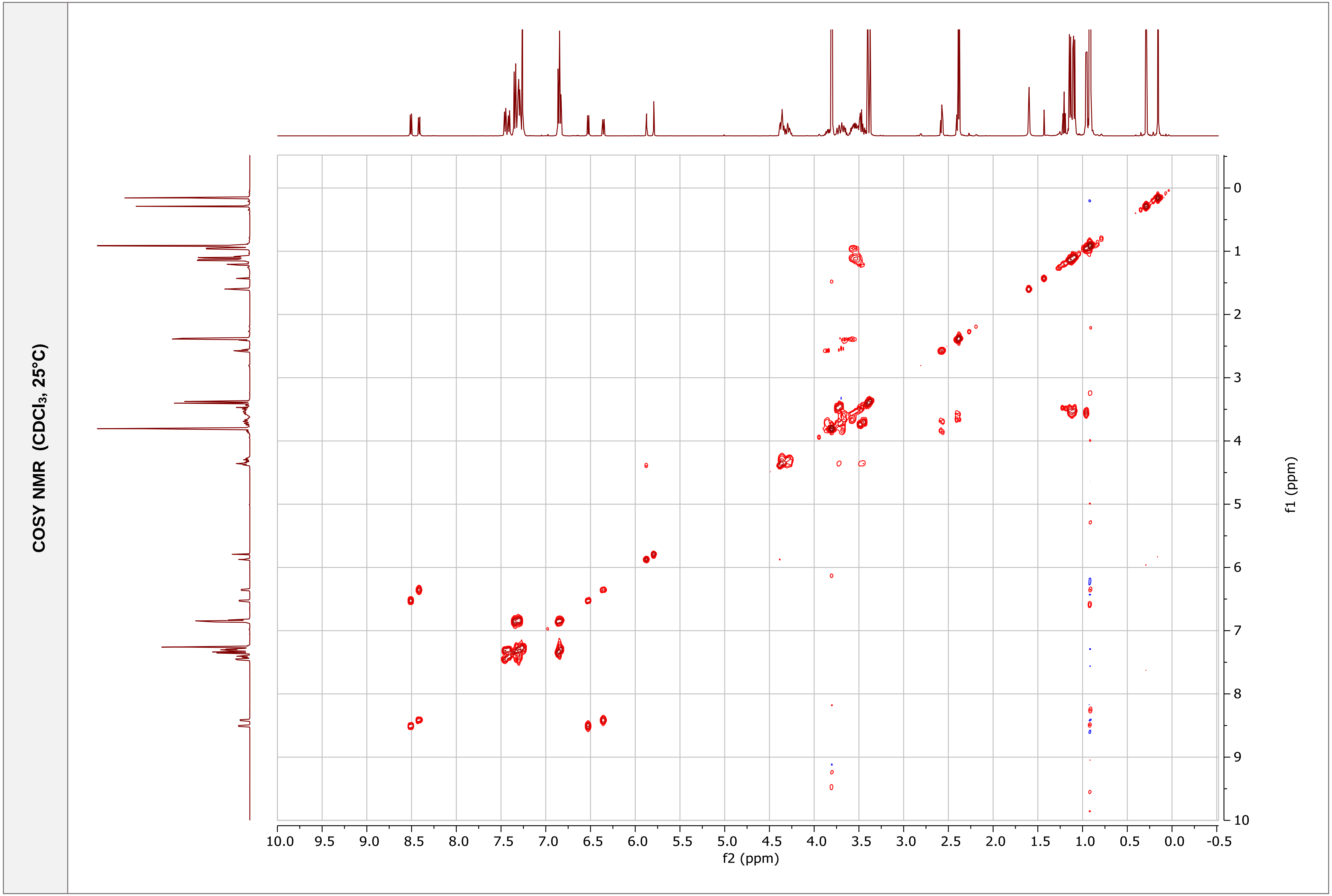
COSY NMR (CDCl$_3$, 25°C)
f2 (ppm)
f1 (ppm)

^{1}H-^{13}C HSQC (CDCl$_3$, 25°C)

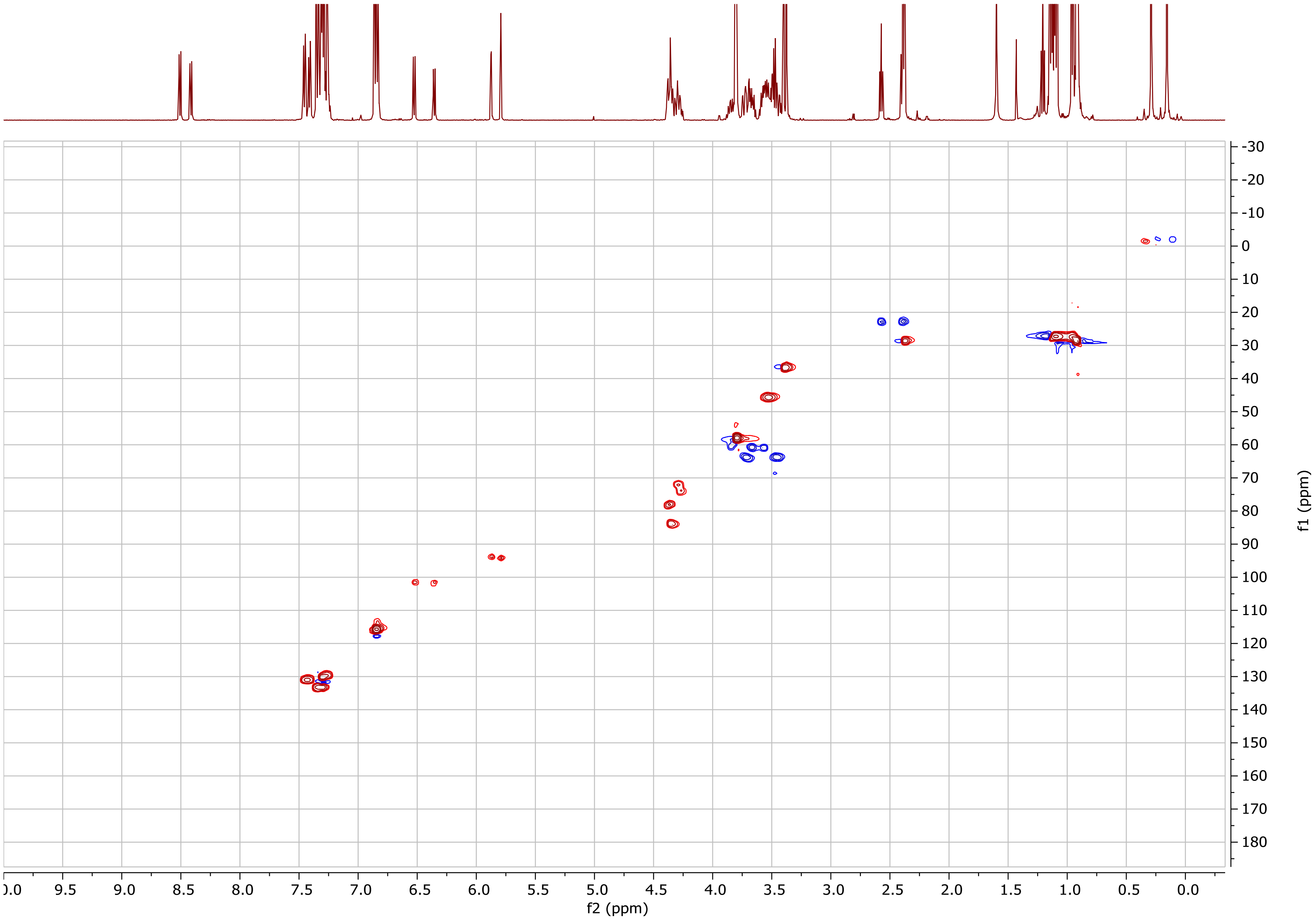

^{1}H-^{31}P HSQC (CDCl$_3$, 25°C)

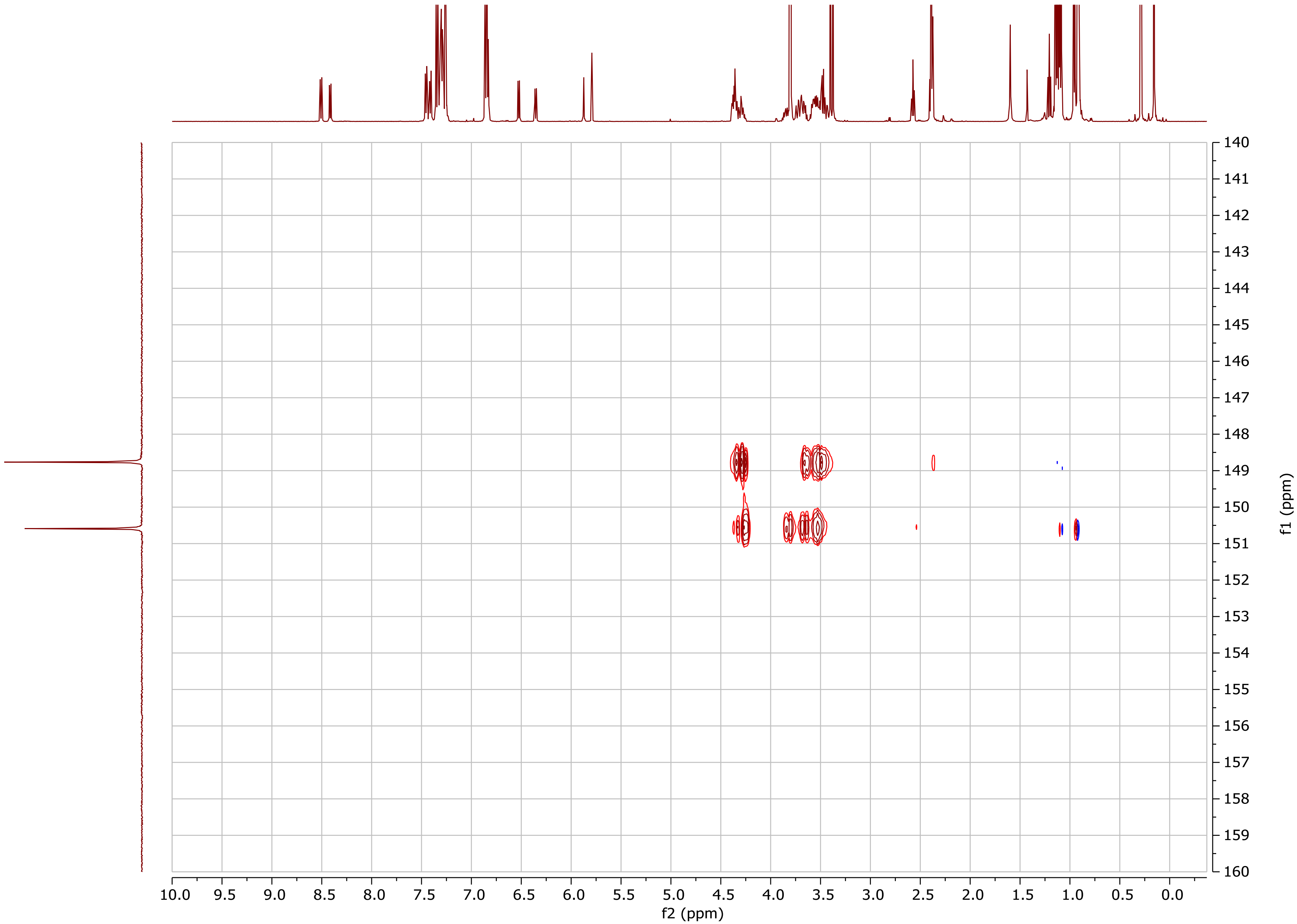

(6a) *N*3-methylcytidine phosphoramidite (5′-*O*-DMT-2′-*O*-TBDMS-m^{3}C^{Ac})

MS (+) ESI

(Calc. $[M+H]^+$ $C_{48}H_{67}N_5O_9PSi^+$ 916.44402)

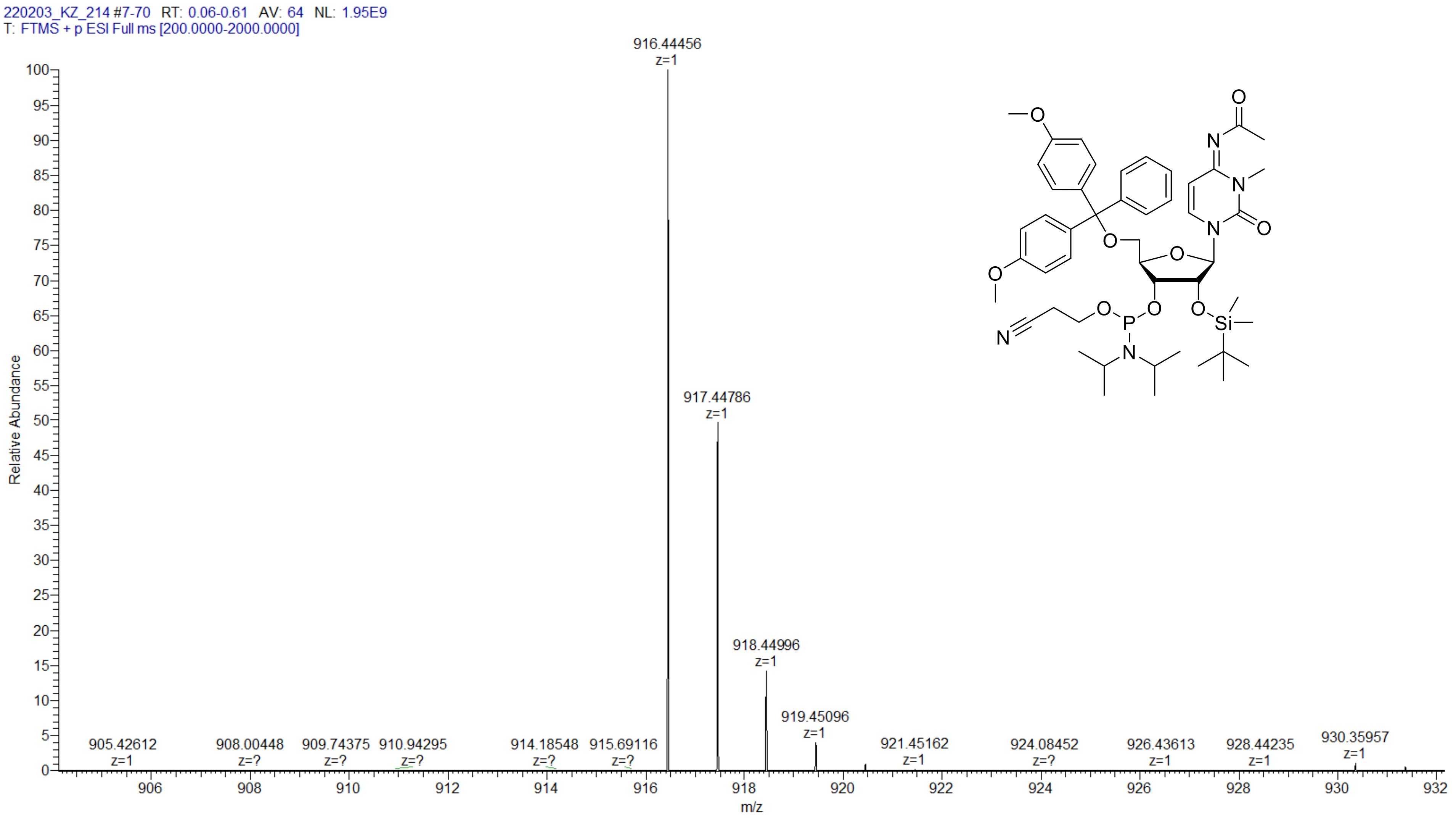

^{1}H NMR (500 MHz, $CDCl_3$, 25°C)

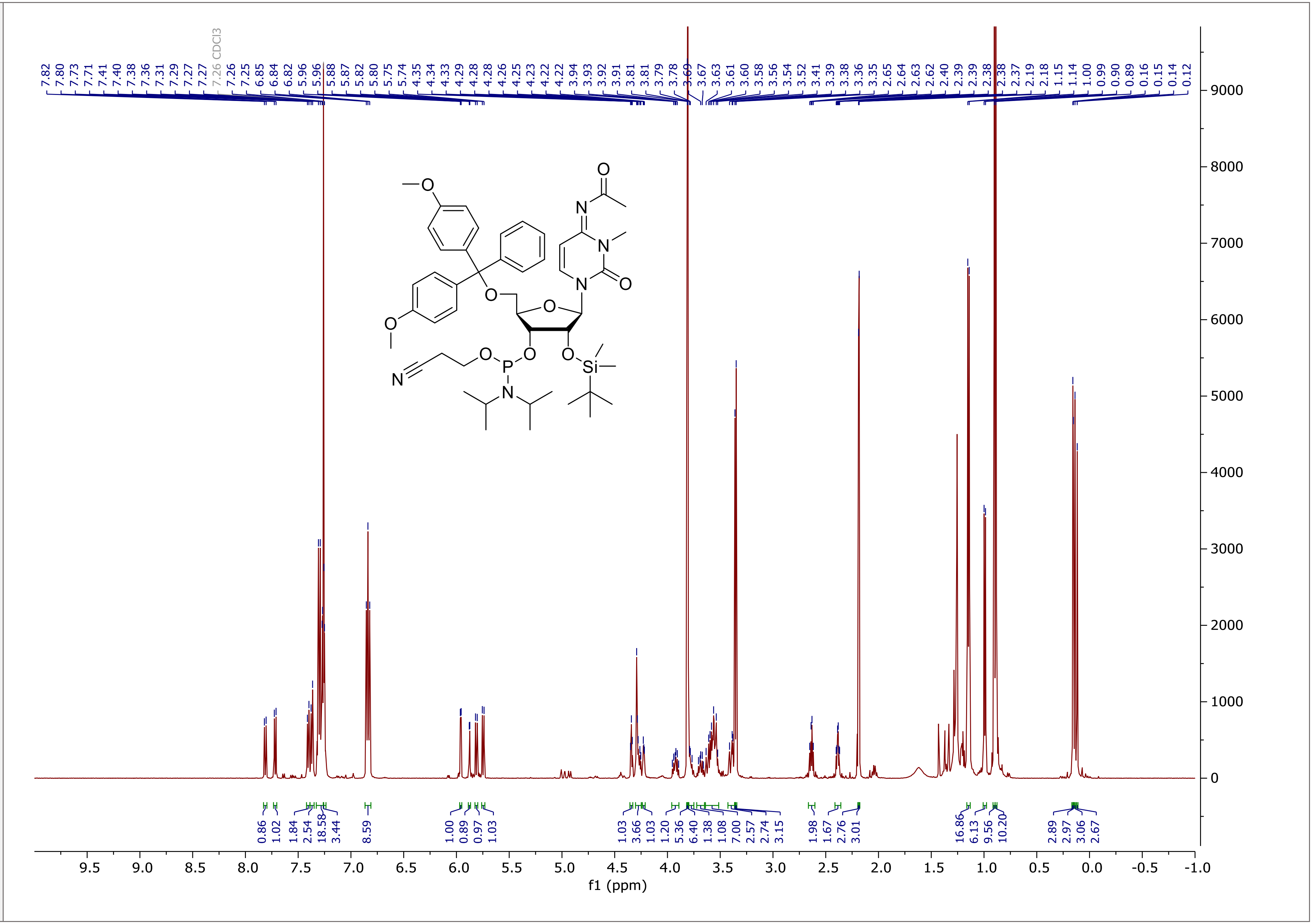

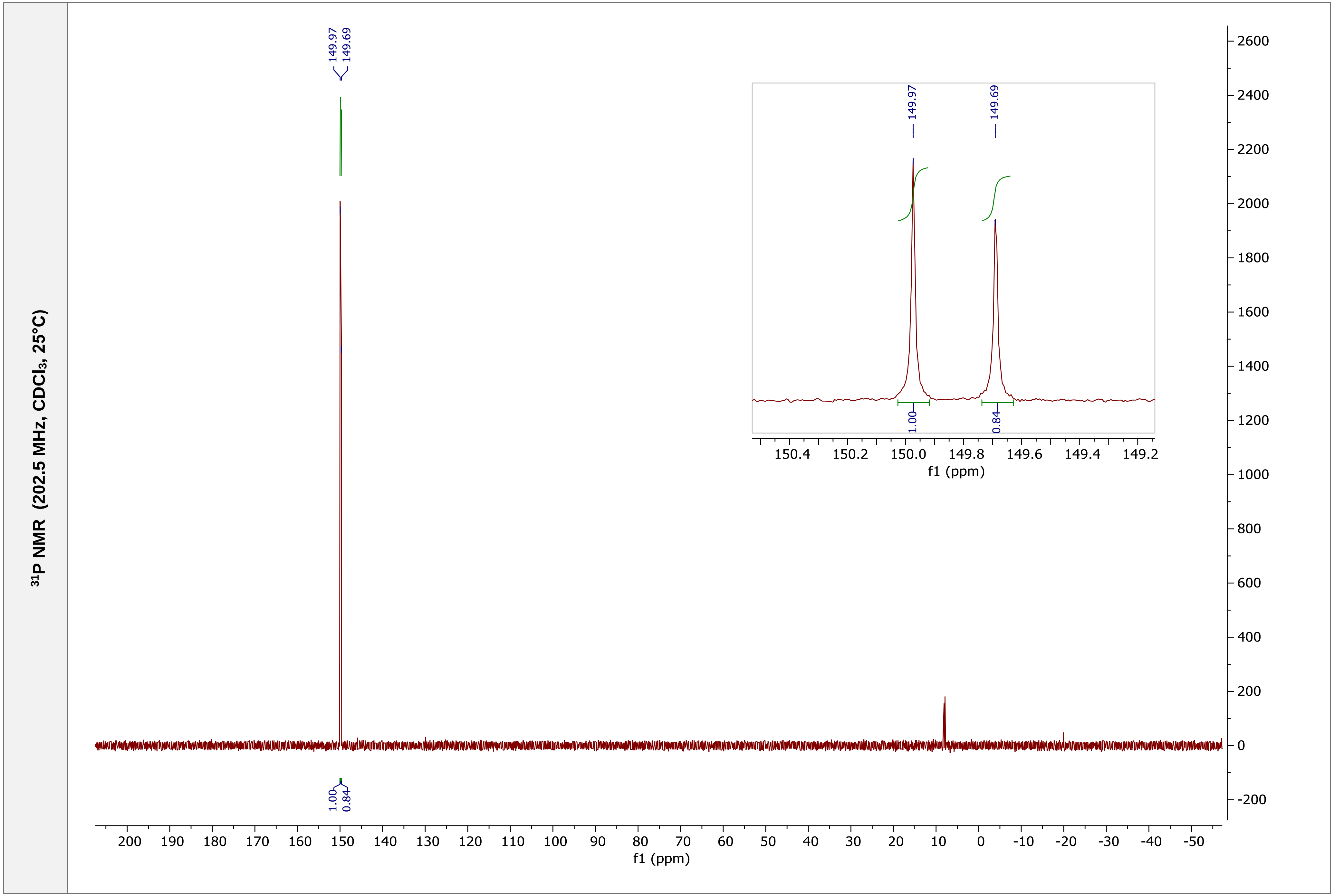

^{31}P NMR (202.5 MHz, CDCl$_3$, 25°C)
149.97
149.69
1.00
0.84
f1 (ppm)

COSY NMR (CDCl$_3$, 25°C)

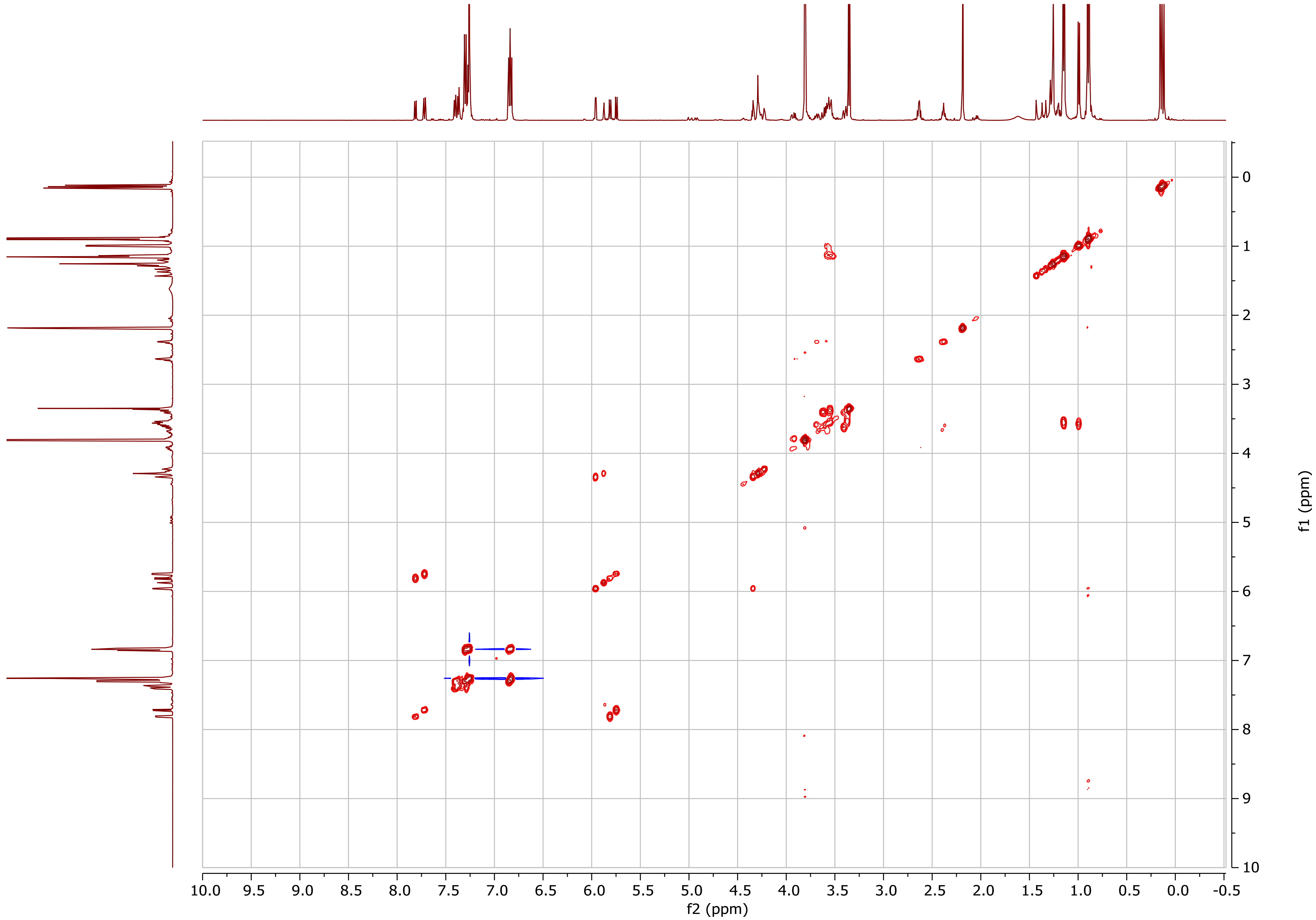

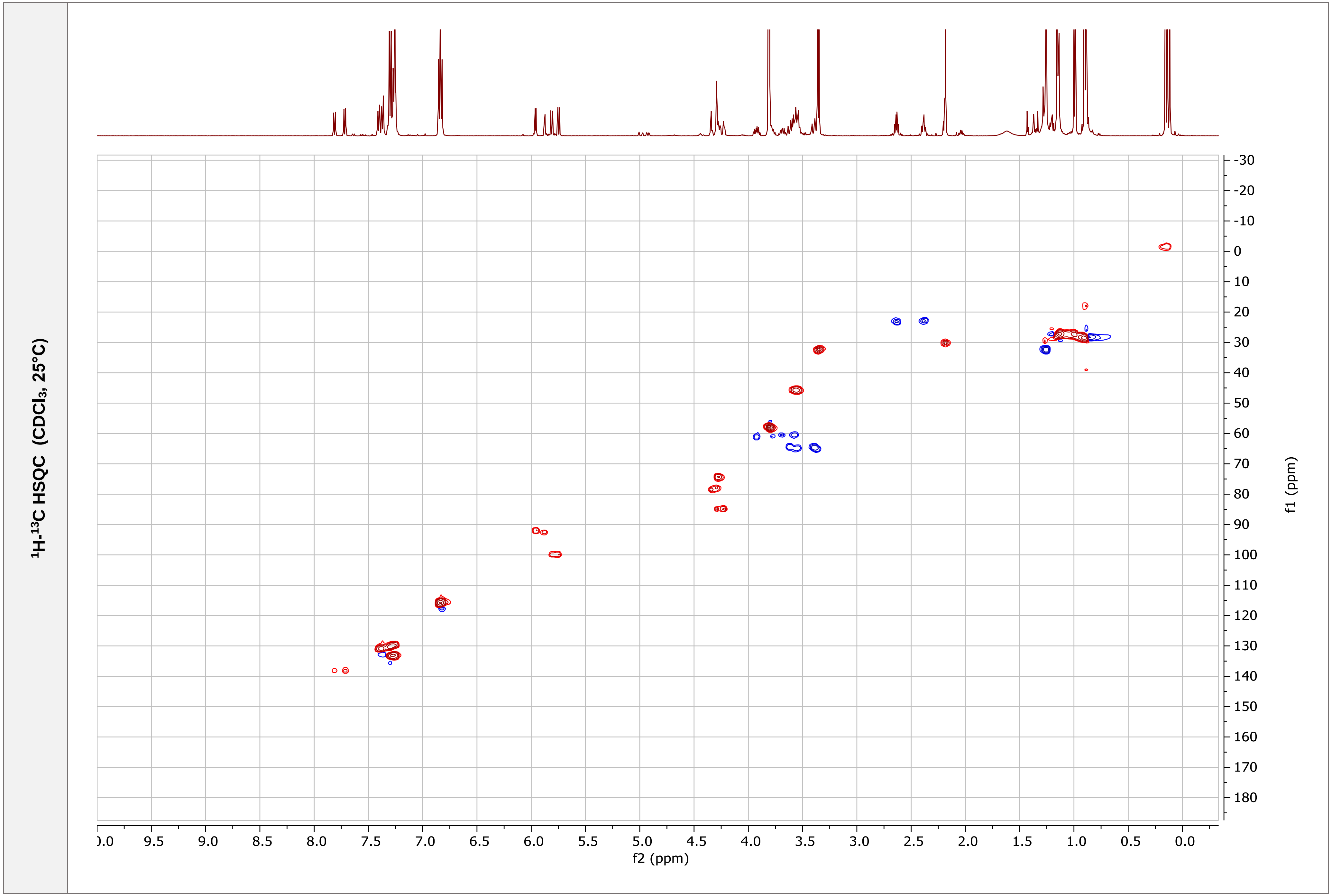

^{1}H-^{13}C HSQC (CDCl$_3$, 25°C)
f2 (ppm)
f1 (ppm)

^{1}H-^{31}P HSQC (CDCl$_3$, 25°C)

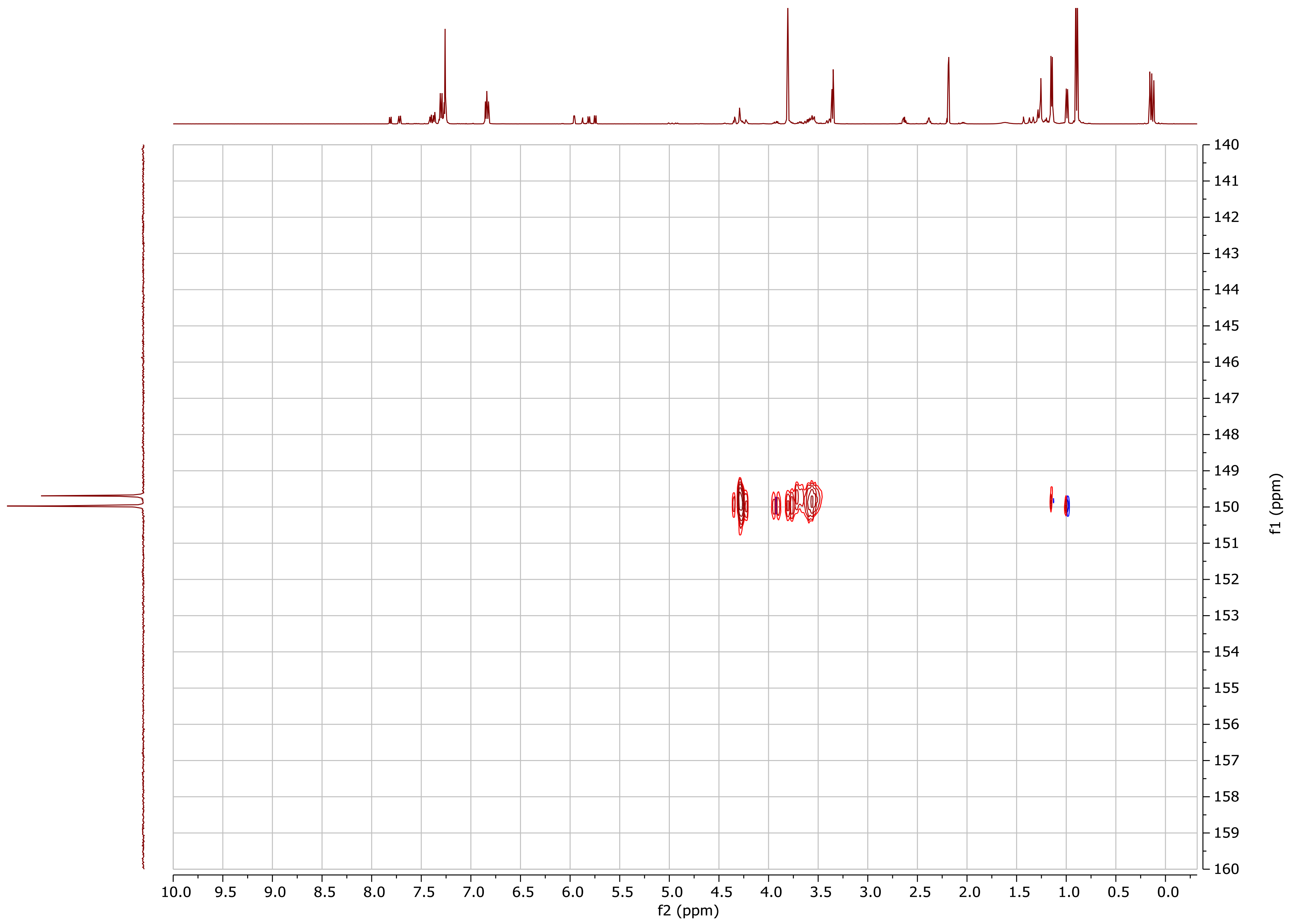

(6b) *N*3-methylcytidine phosphoramidite (5′-*O*-DMT-2′-*O*-TBDMS-m^{3}C^{Bz})

MS (+) ESI

(Calc. [M+H]$^+$ $C_{53}H_{69}N_5O_9PSi^+$ 978.45967)

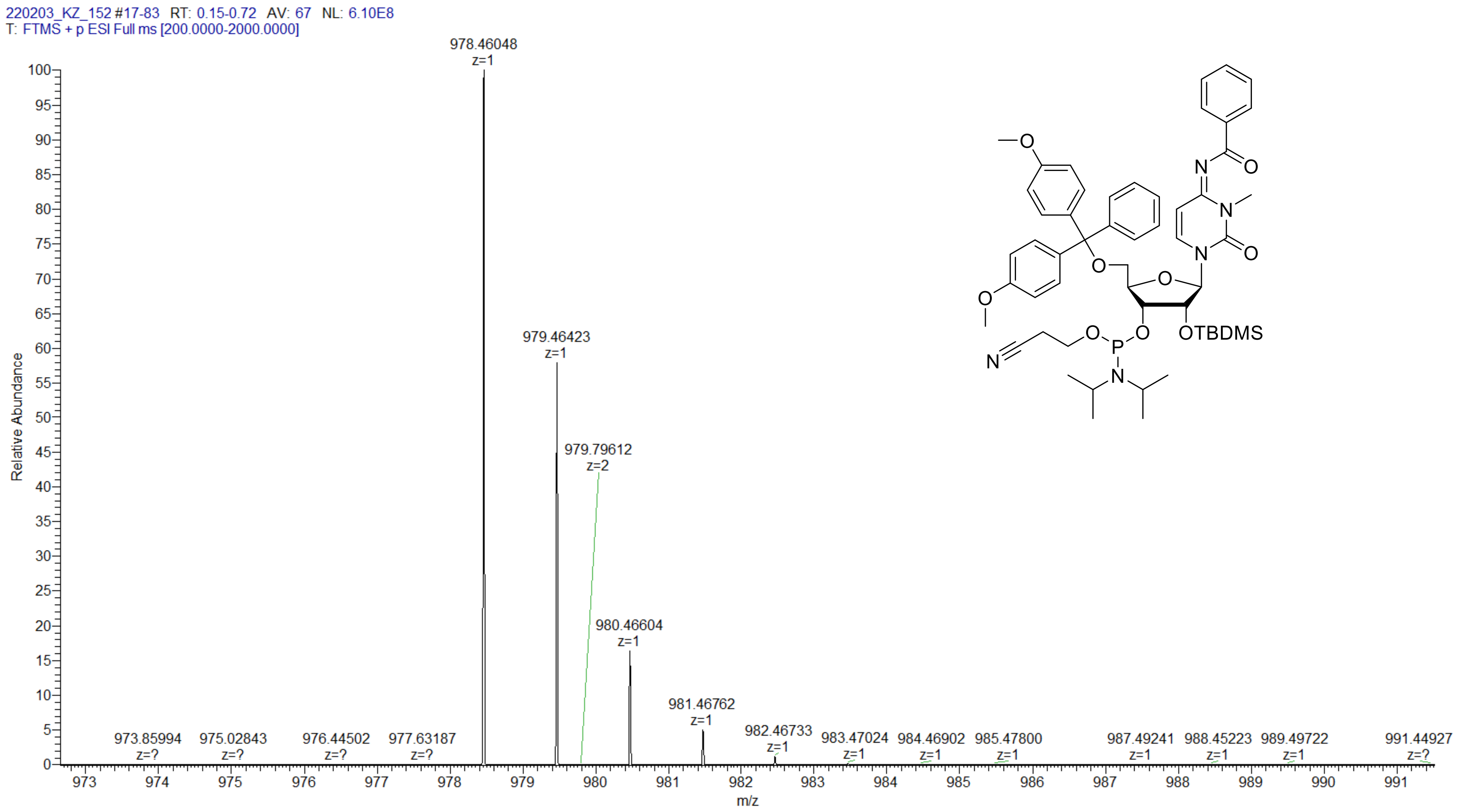

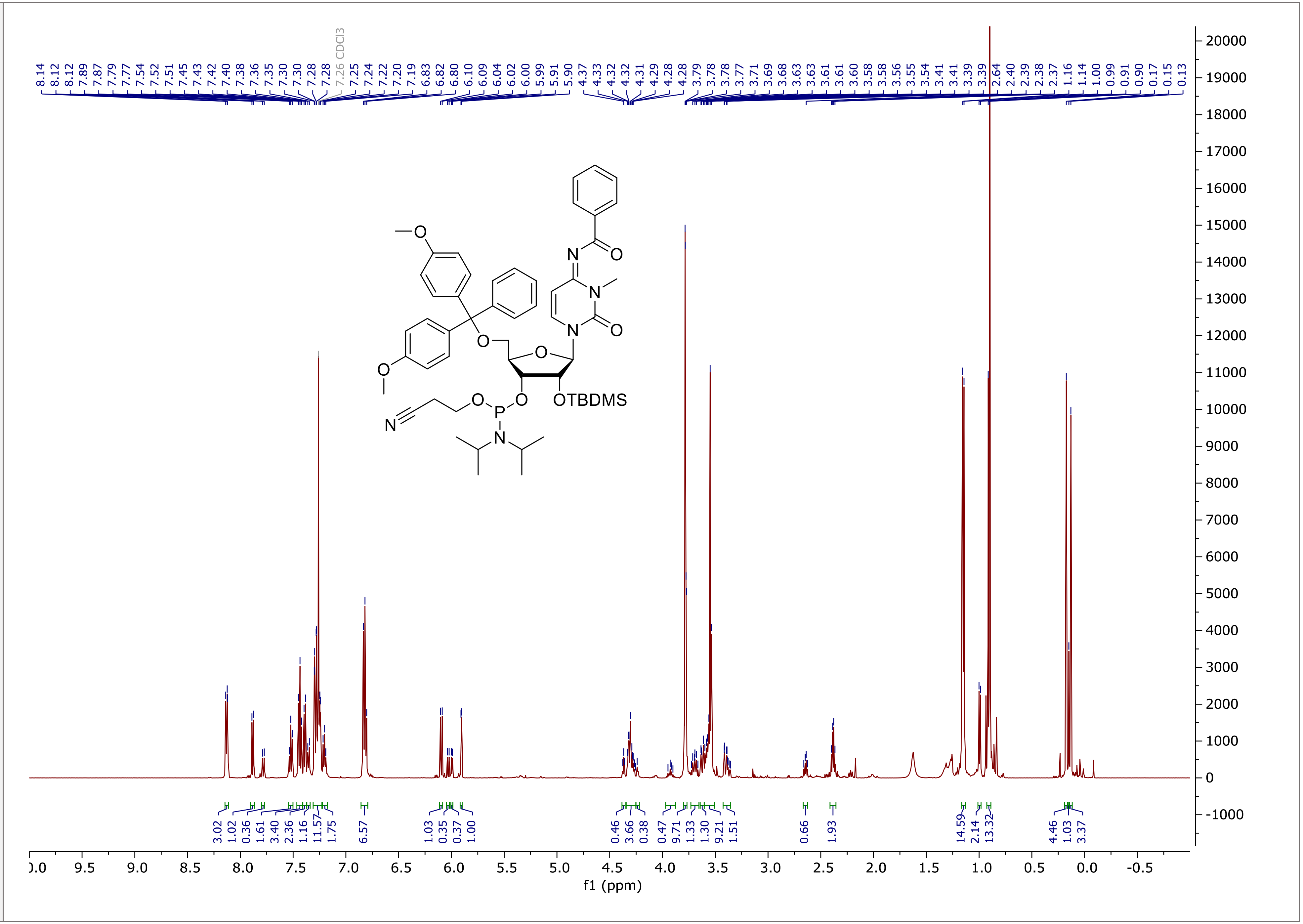

^{1}H NMR (500 MHz, $CDCl_3$, 25°C)

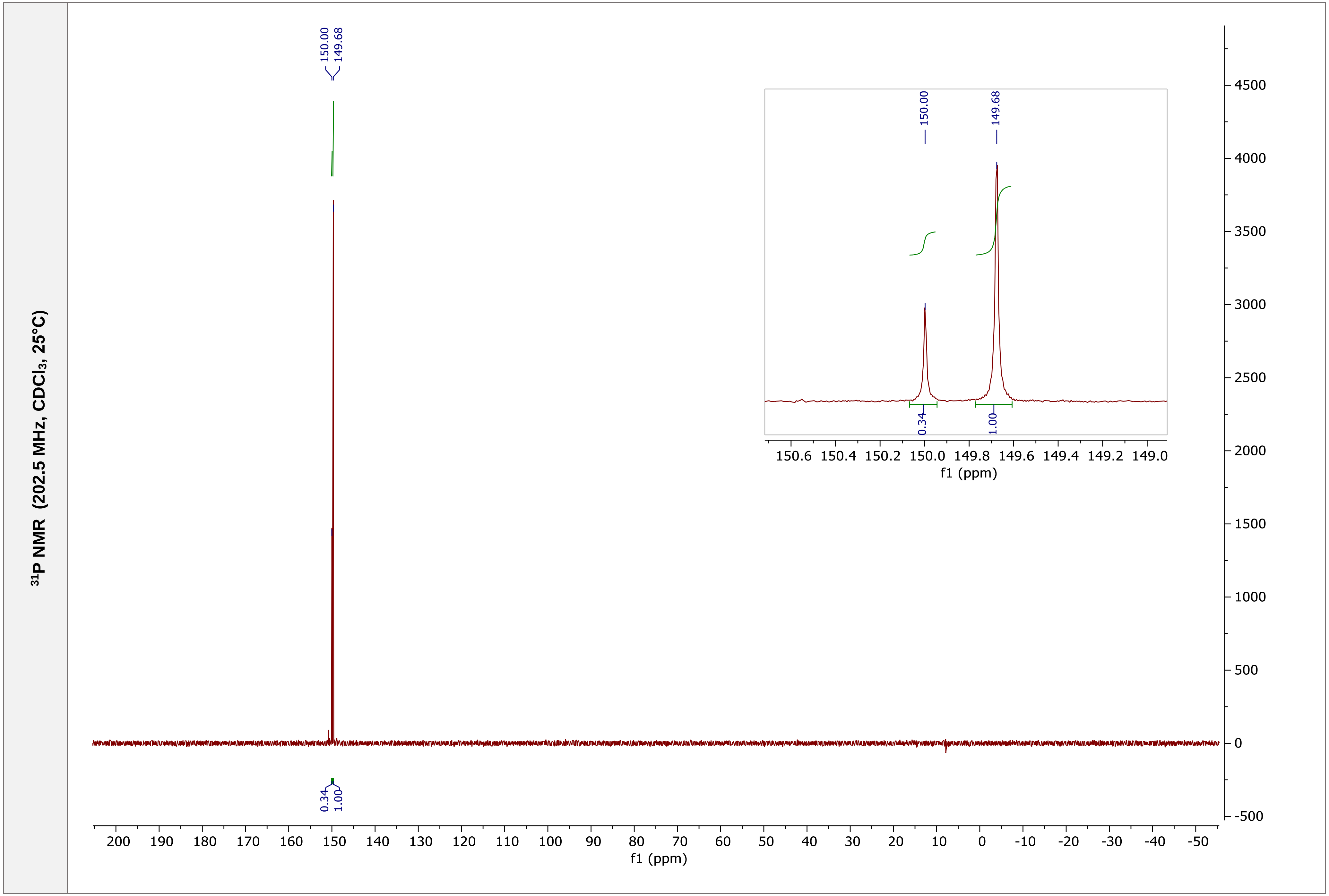

^{31}P NMR (202.5 MHz, $CDCl_3$, 25°C)
150.00
149.68
0.34
1.00
f1 (ppm)

COSY NMR (CDCl$_3$, 25°C)

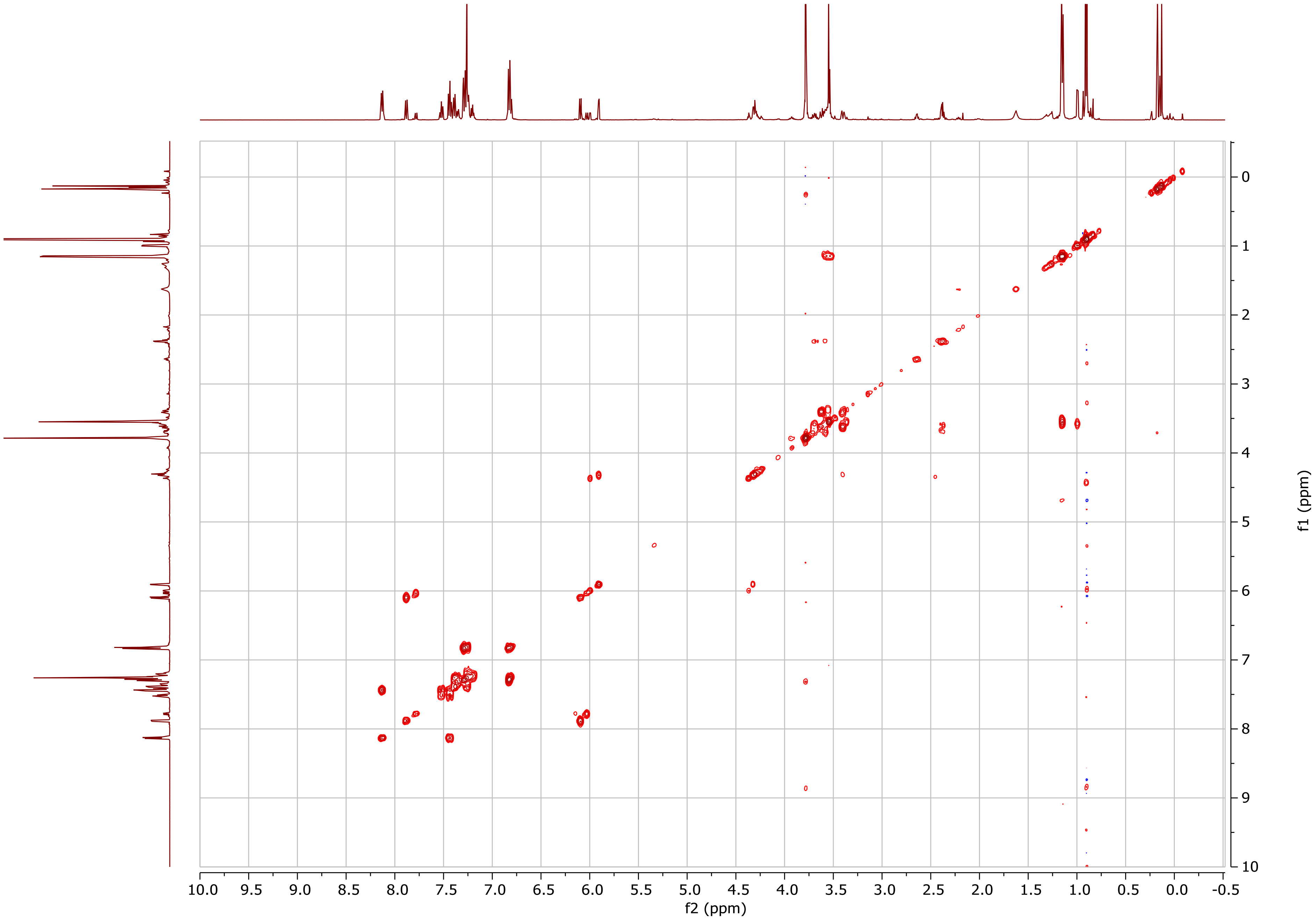

^{1}H-^{13}C HSQC (CDCl$_3$, 25°C)

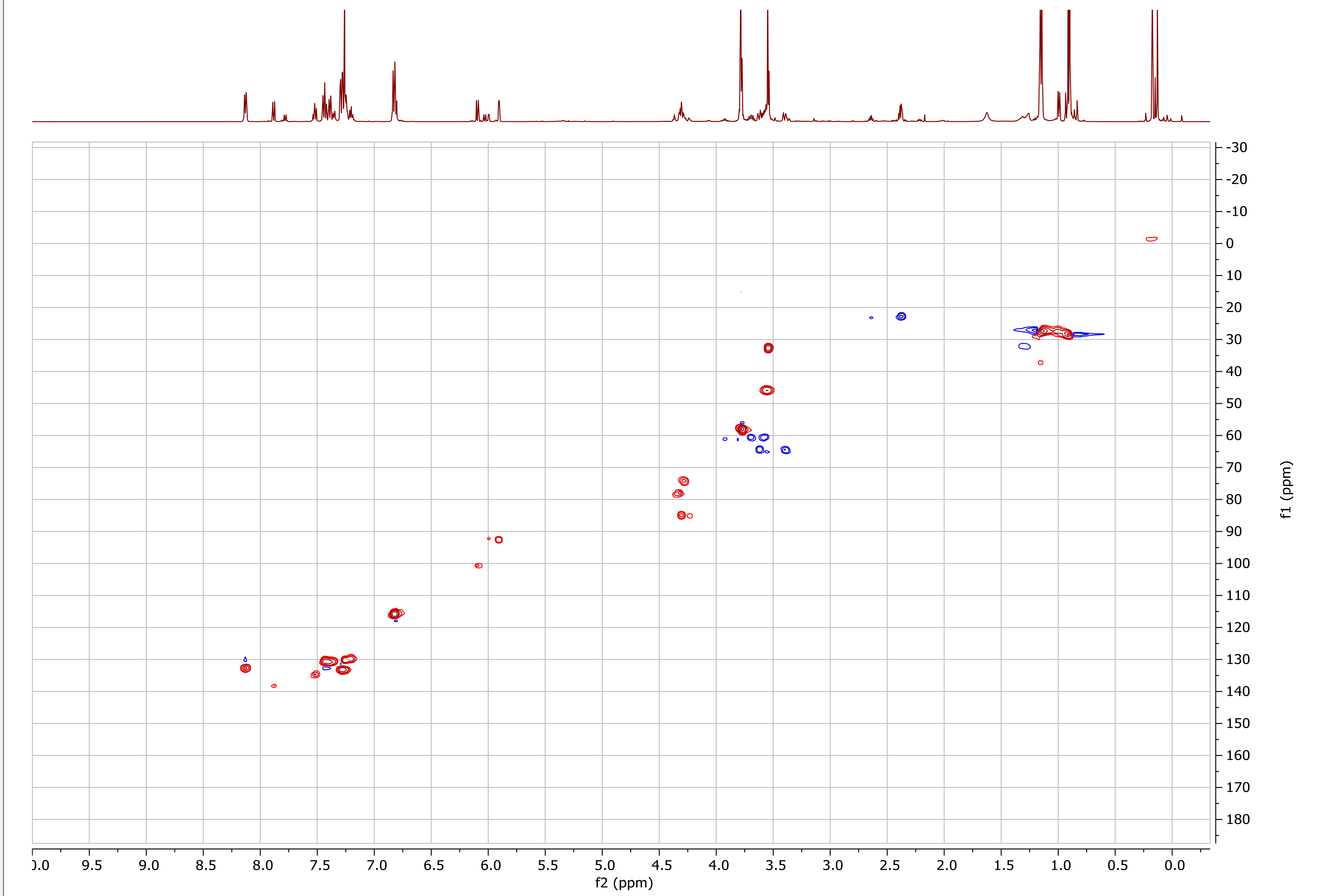

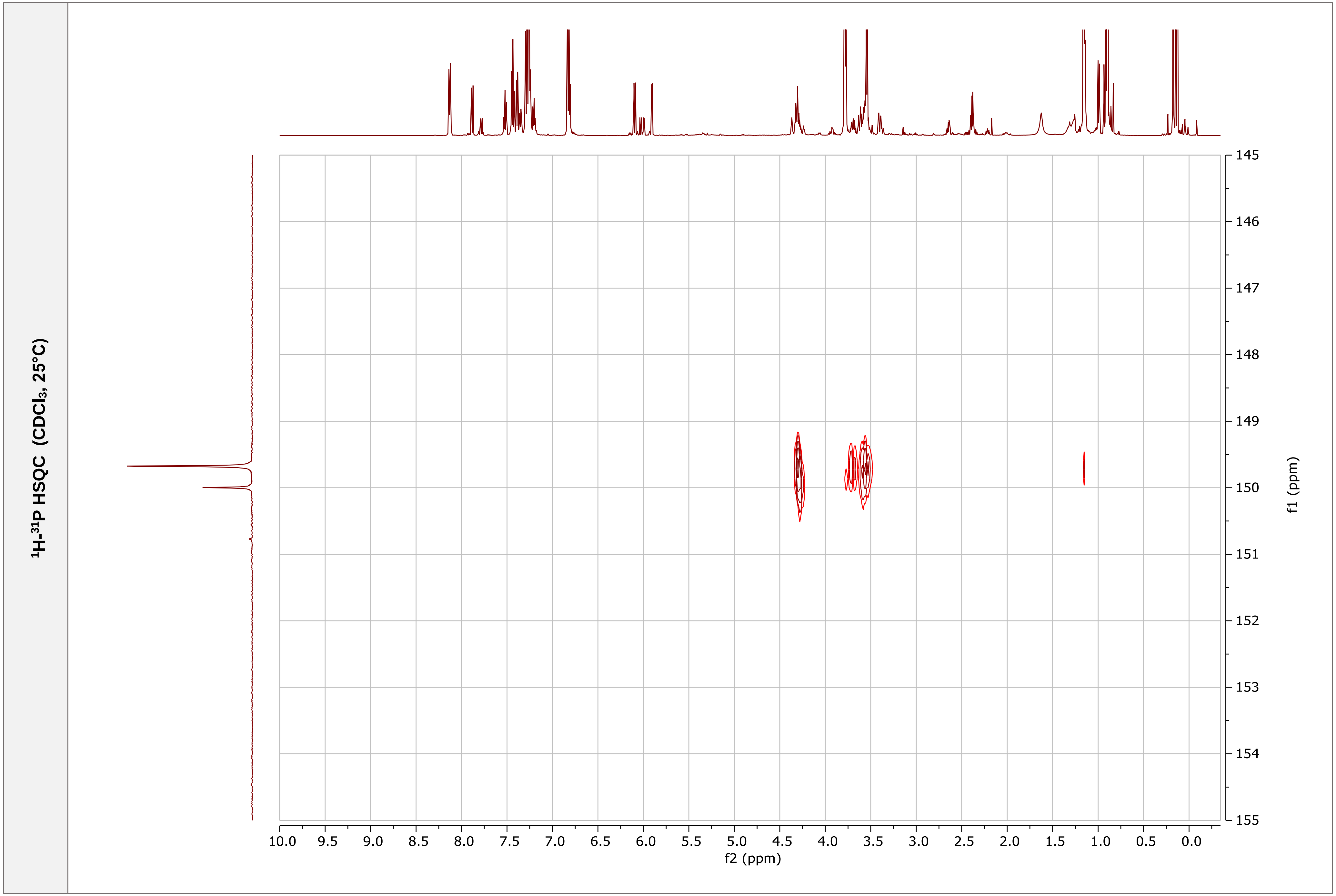

¹H-³¹P HSQC (CDCl₃, 25°C)
f2 (ppm)
f1 (ppm)

(6c) *N*3-(2-nitrobenzyl)cytidine phosphoramidite (5′-*O*-DMT-2′-*O*-TBDMS-nb^{3}C^{Bz})

MS (+) ESI
(Calc. [M+H]$^+$ $C_{59}H_{72}N_6O_{11}PSi^+$ 1099.47605)

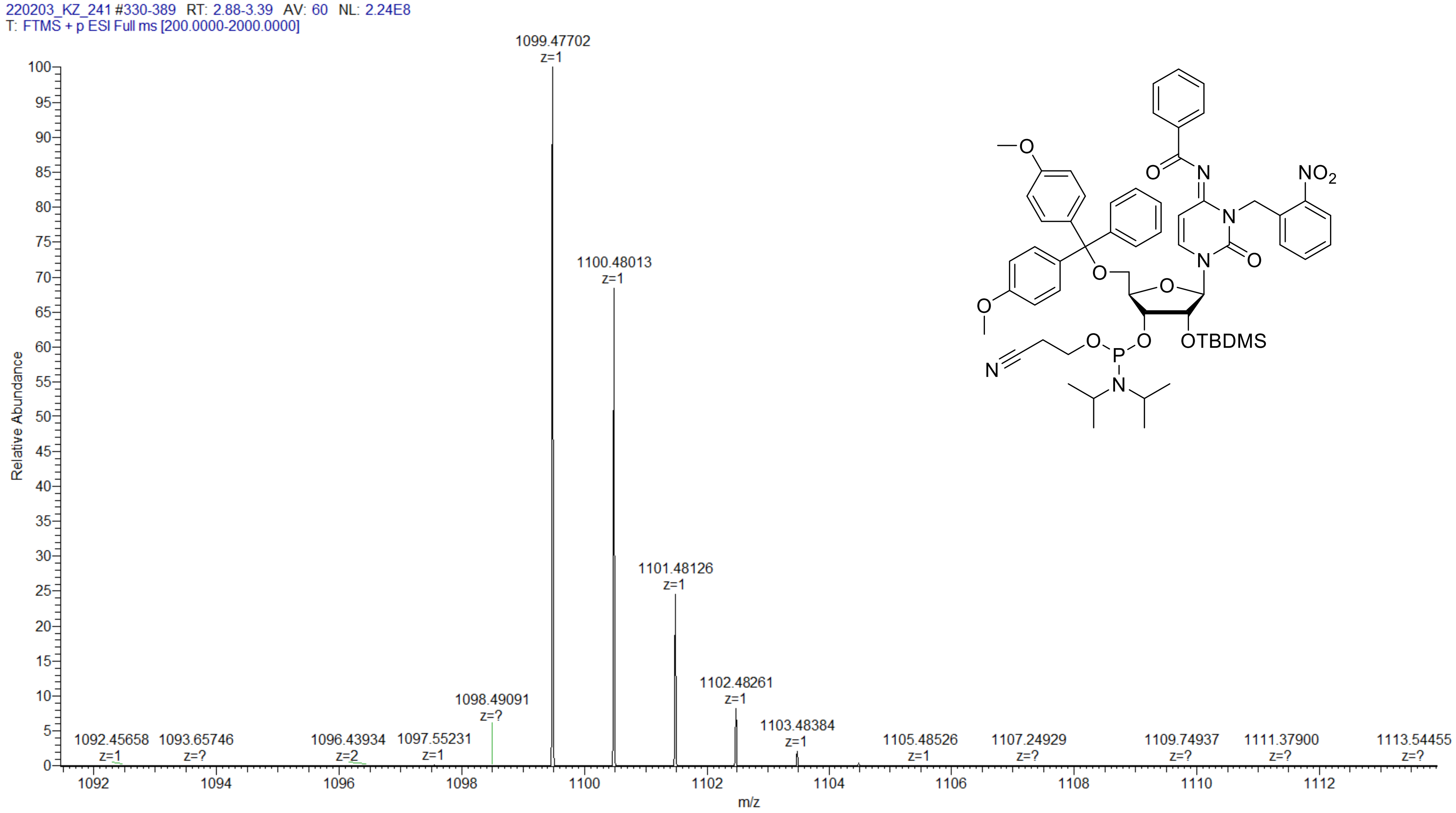

^{1}H NMR (500 MHz, $CDCl_3$, 25°C)

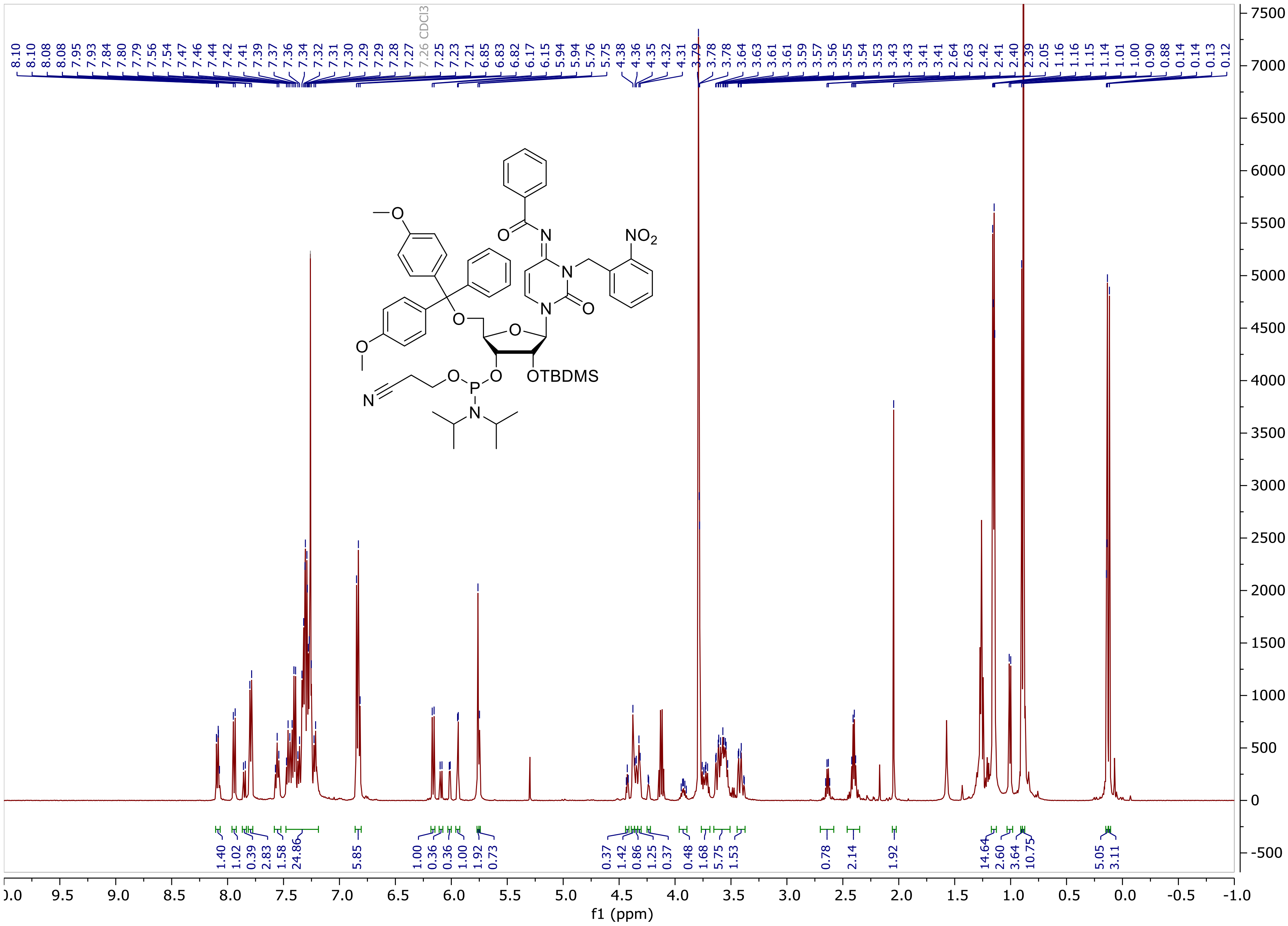

^{31}P NMR (202.5 MHz, $CDCl_3$, 25°C)

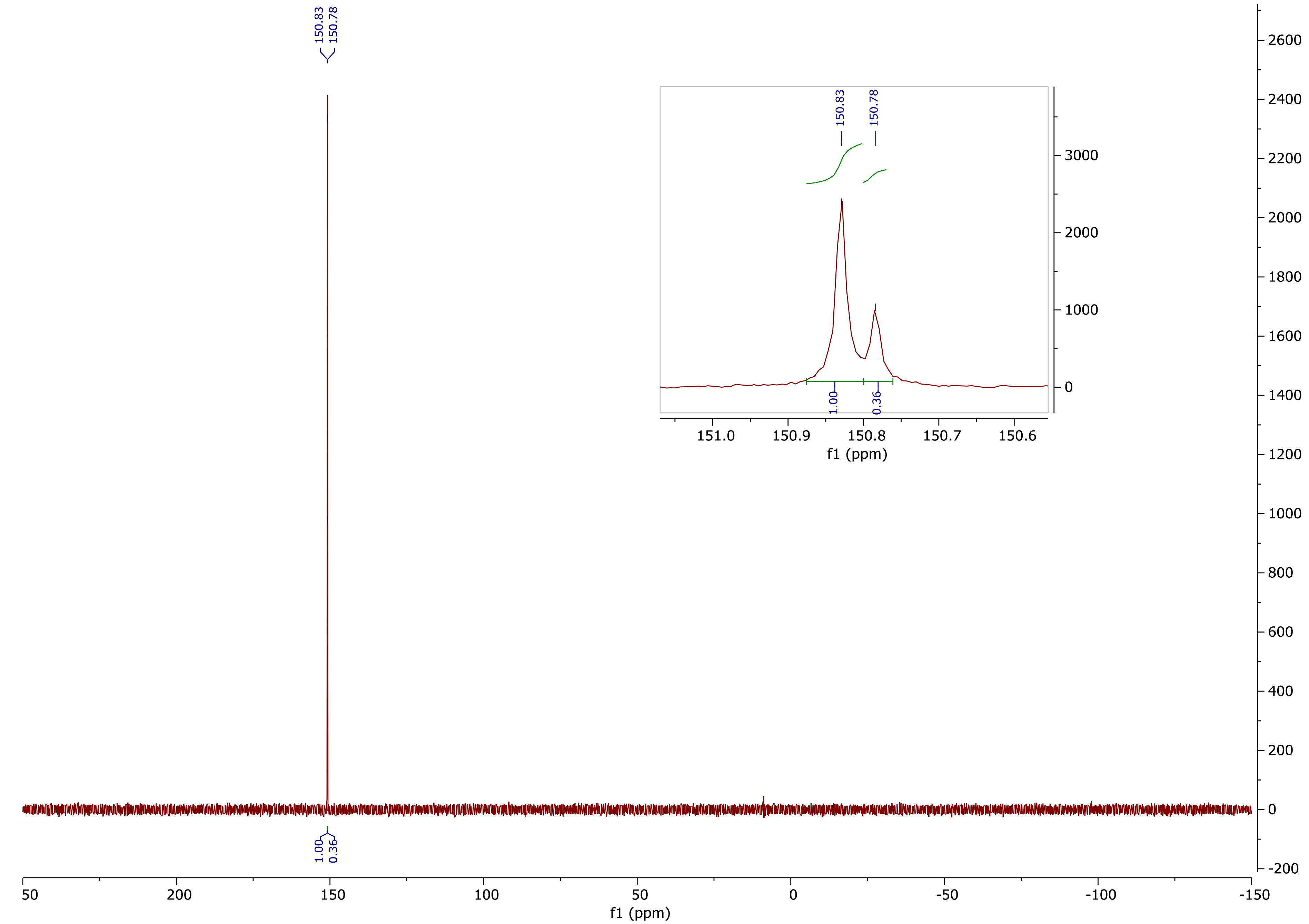

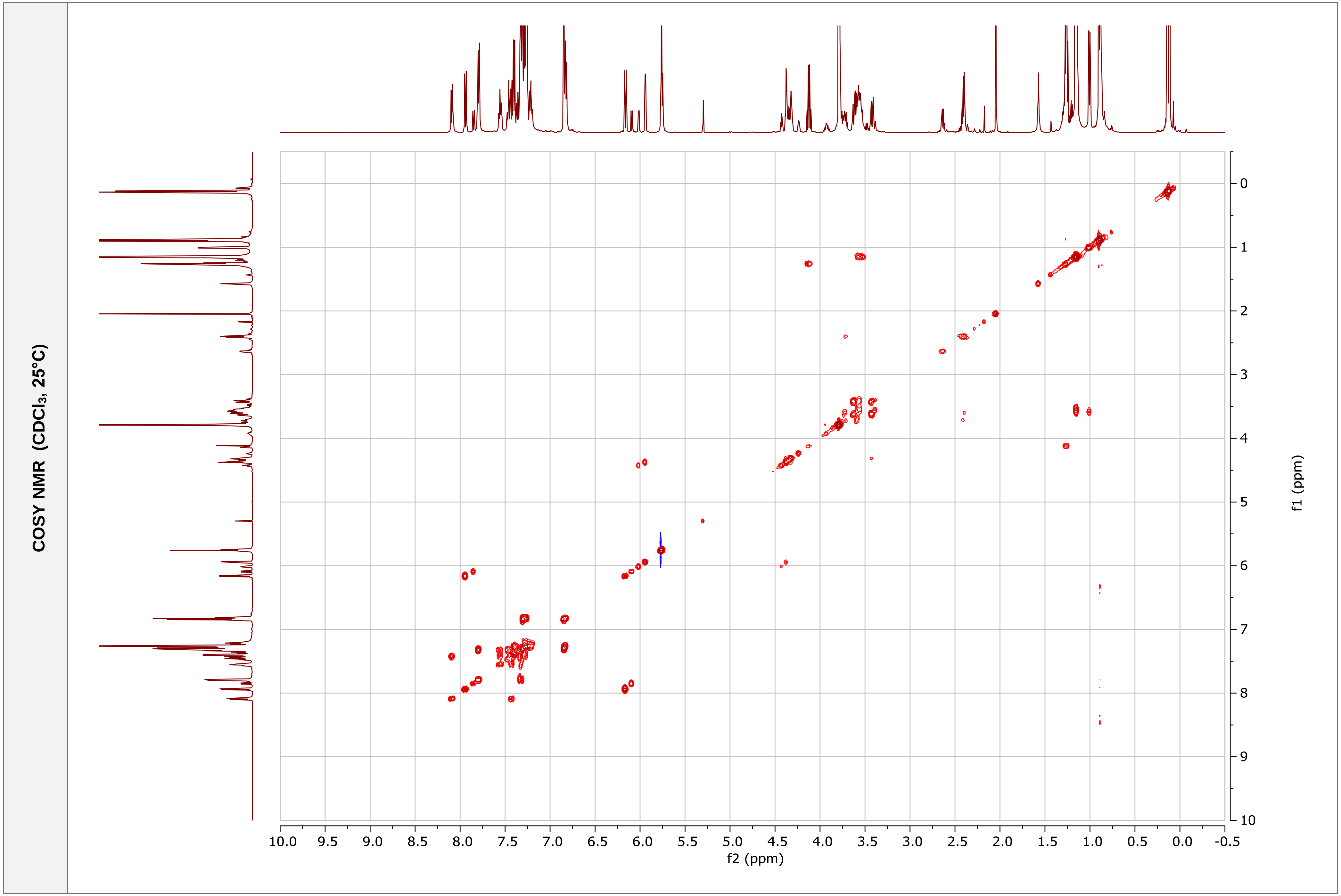
COSY NMR (CDCl$_3$, 25°C)
f2 (ppm)
f1 (ppm)
10.0 9.5 9.0 8.5 8.0 7.5 7.0 6.5 6.0 5.5 5.0 4.5 4.0 3.5 3.0 2.5 2.0 1.5 1.0 0.5 0.0 -0.5
0 1 2 3 4 5 6 7 8 9 10

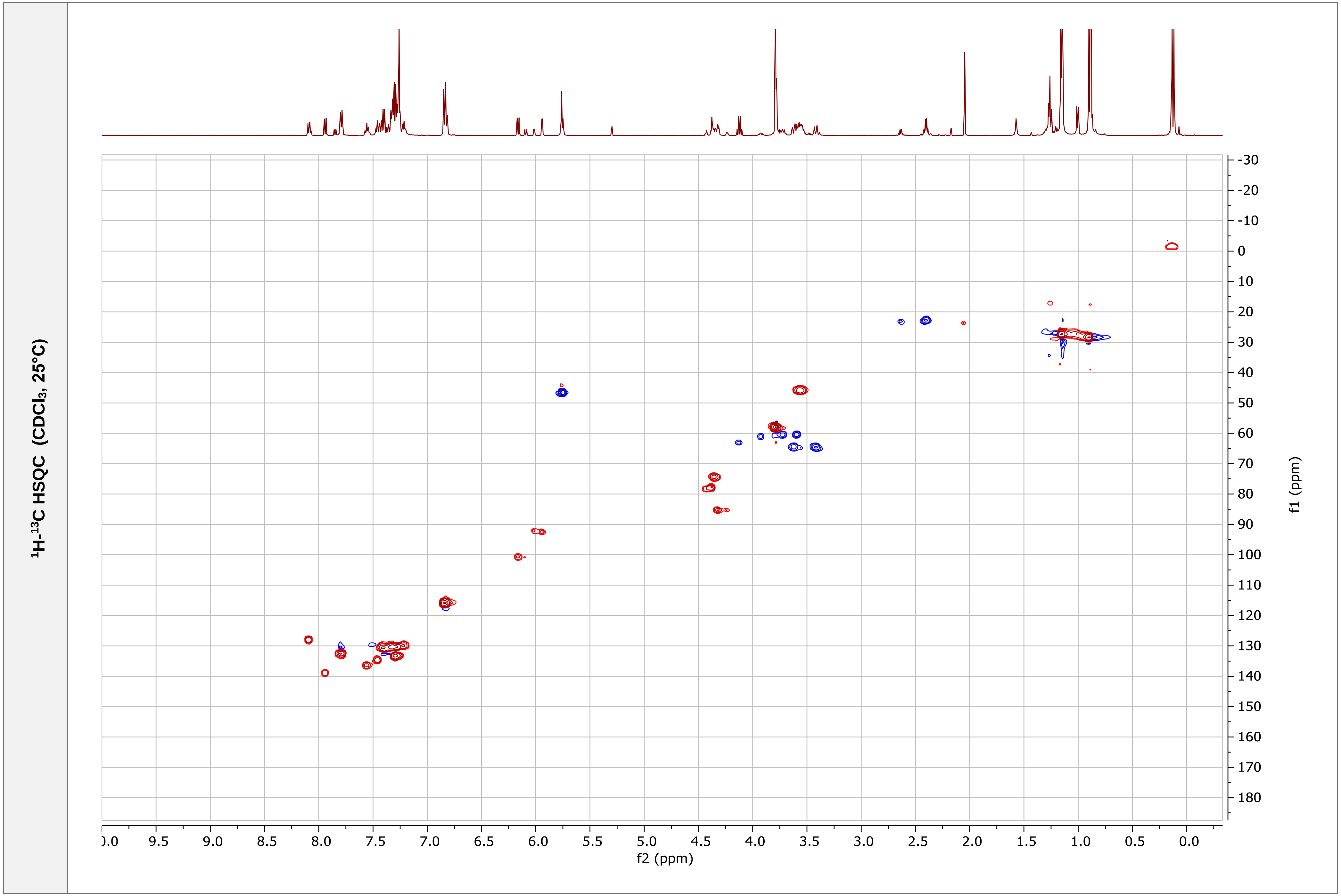

^{1}H-^{13}C HSQC (CDCl$_3$, 25°C)
f2 (ppm)
f1 (ppm)

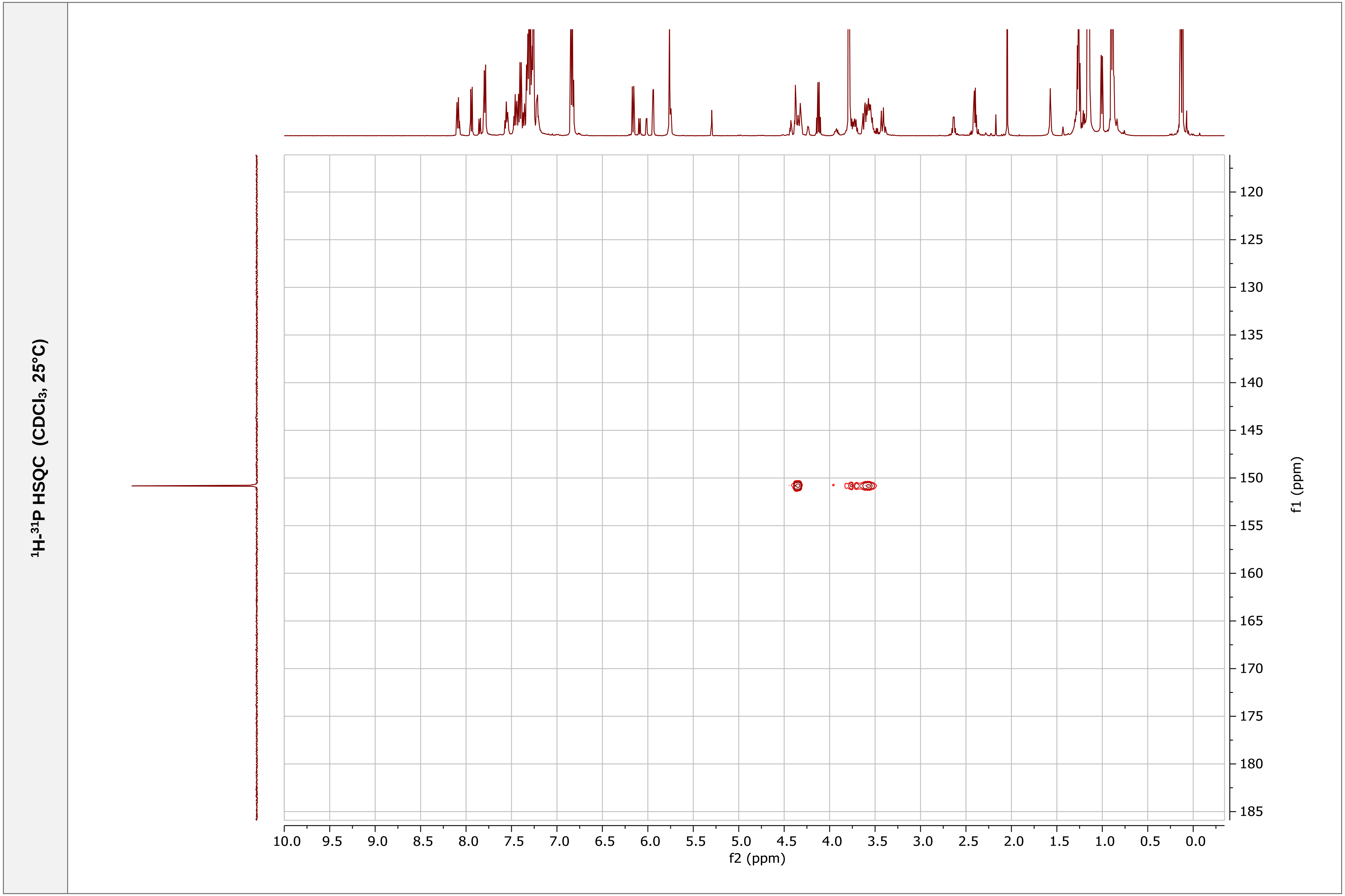
1H-31P HSQC (CDCl3, 25°C)
f2 (ppm)
f1 (ppm)

(7) *N*6-(*N*-phenylcarbamoyl)cytidine phosphoramidite (5′-*O*-DMT-2′-*O*-TBDMS-PhNHCO4C)

MS (+) ESI
(Calc. $[M+H]^+$ $C_{52}H_{68}N_6O_9PSi^+$ 979.45492)

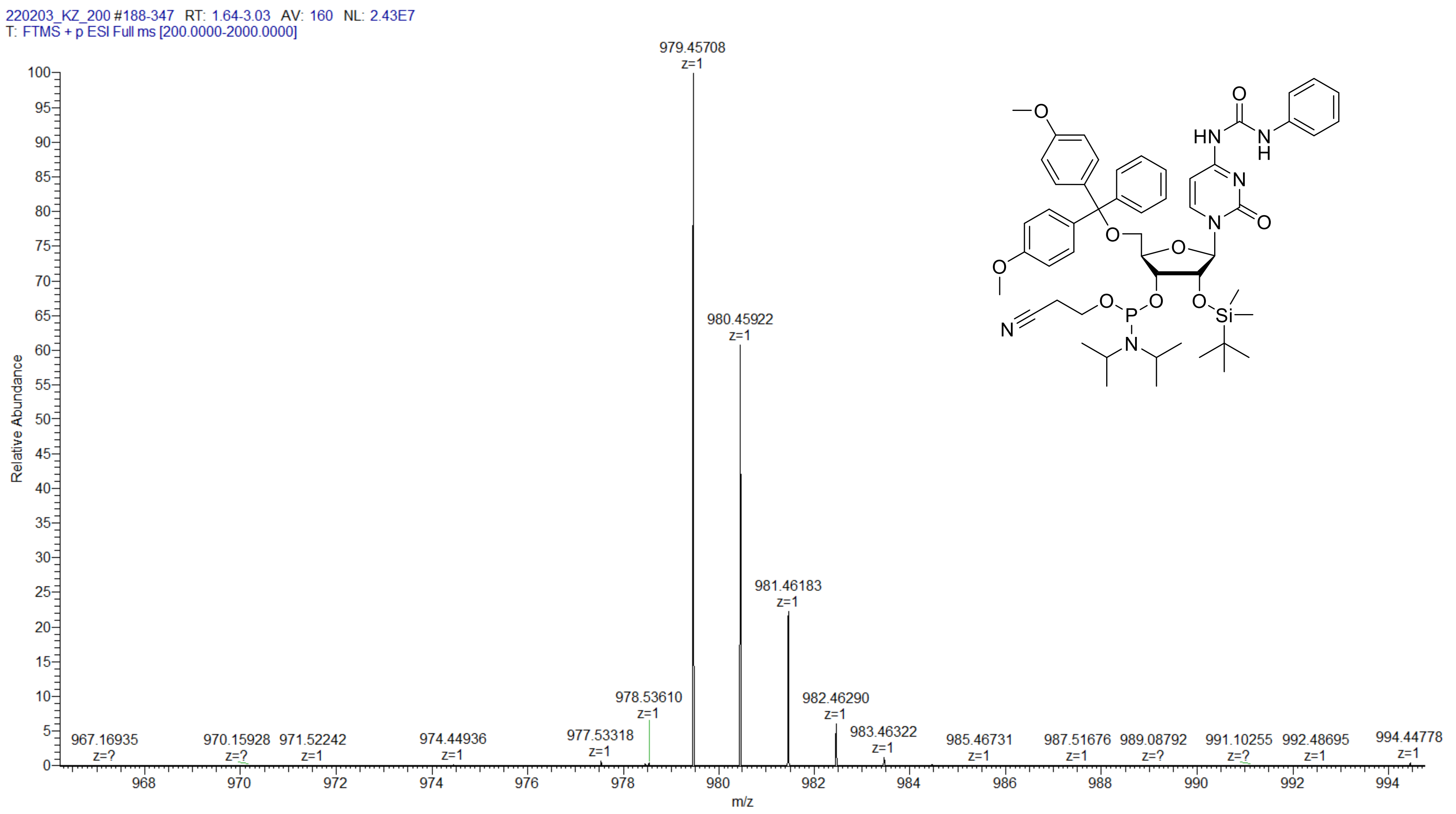

^{1}H NMR (500 MHz, $CDCl_3$, 25°C)

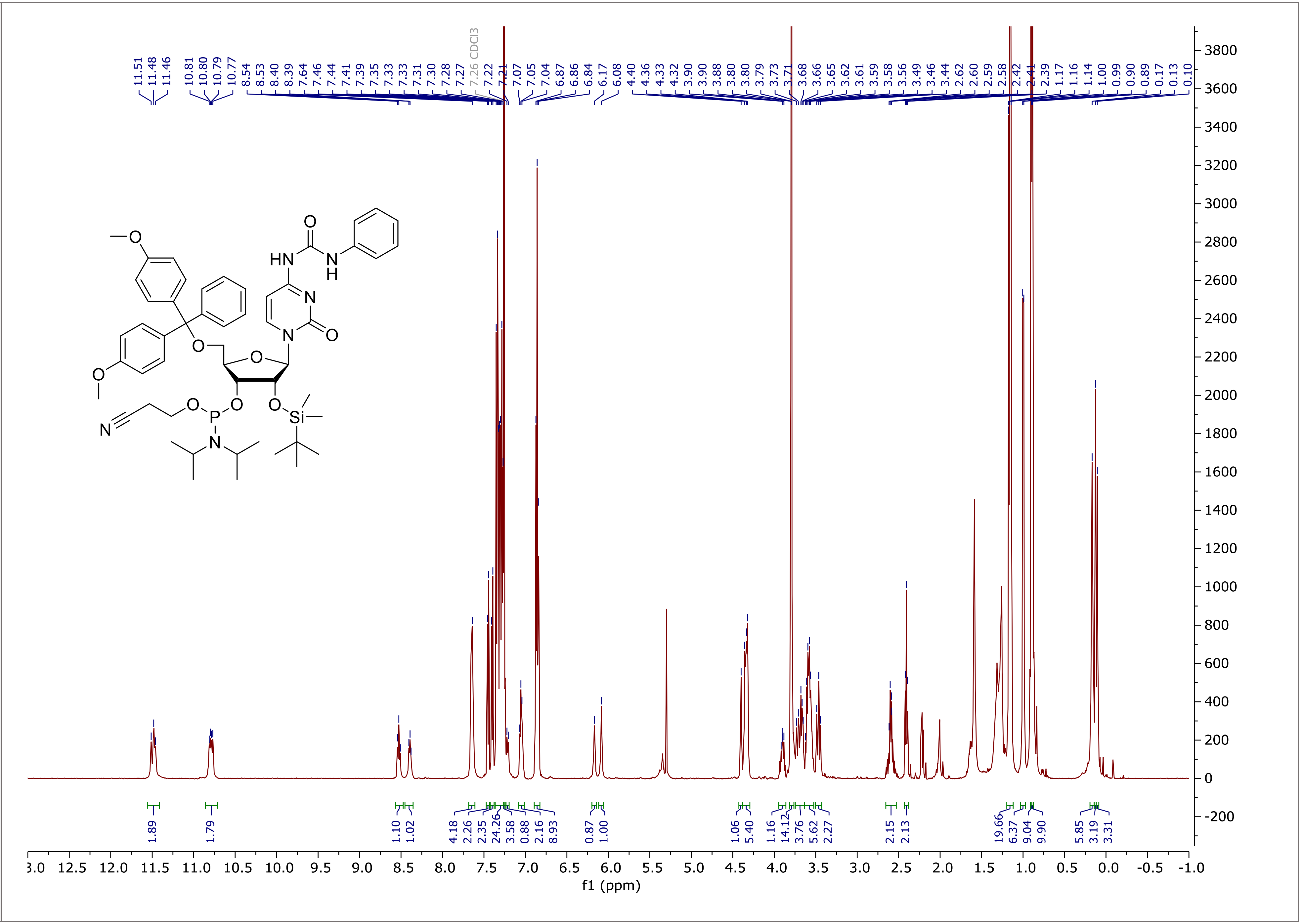

$^{13}C\{^{1}H\}$ NMR (126 MHz, $CDCl_3$, 25°C)

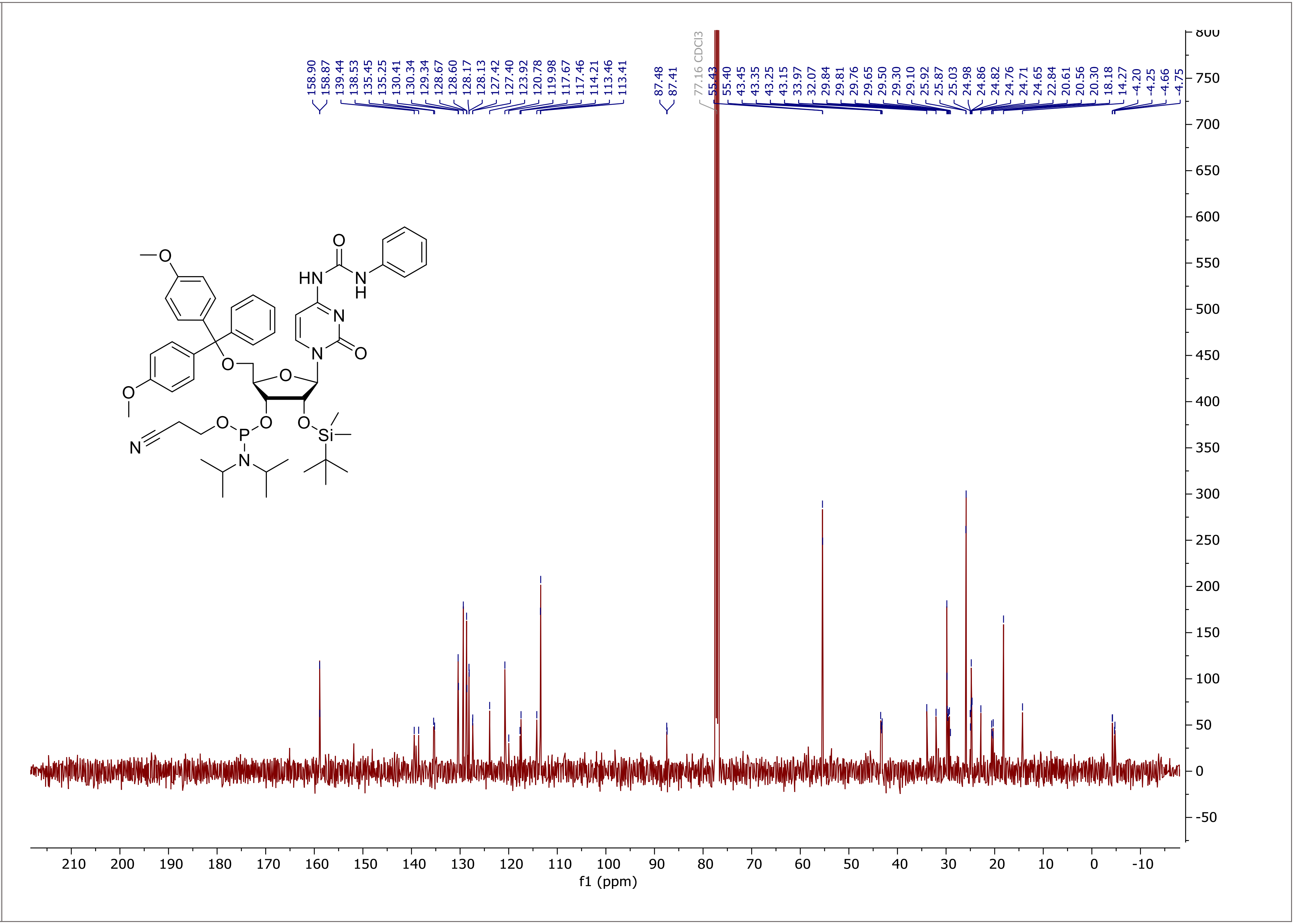

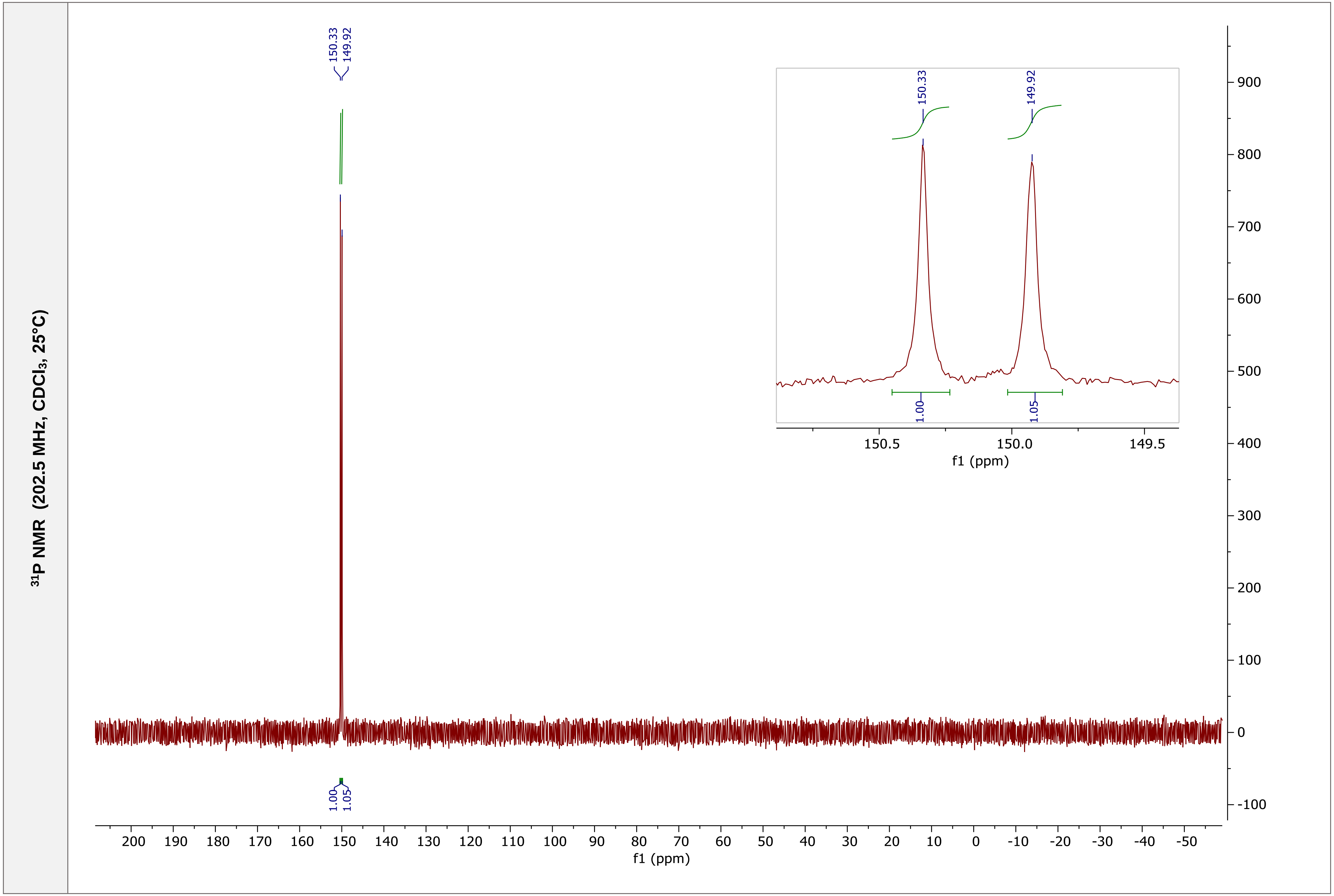
^{31}P NMR (202.5 MHz, $CDCl_3$, 25°C)
150.33
149.92
1.00
1.05
f1 (ppm)
150.5
150.0
149.5
200
190
180
170
160
150
140
130
120
110
100
90
80
70
60
50
40
30
20
10
0
-10
-20
-30
-40
-50
900
800
700
600
500
400
300
200
100
-100

COSY NMR (CDCl$_3$, 25°C)

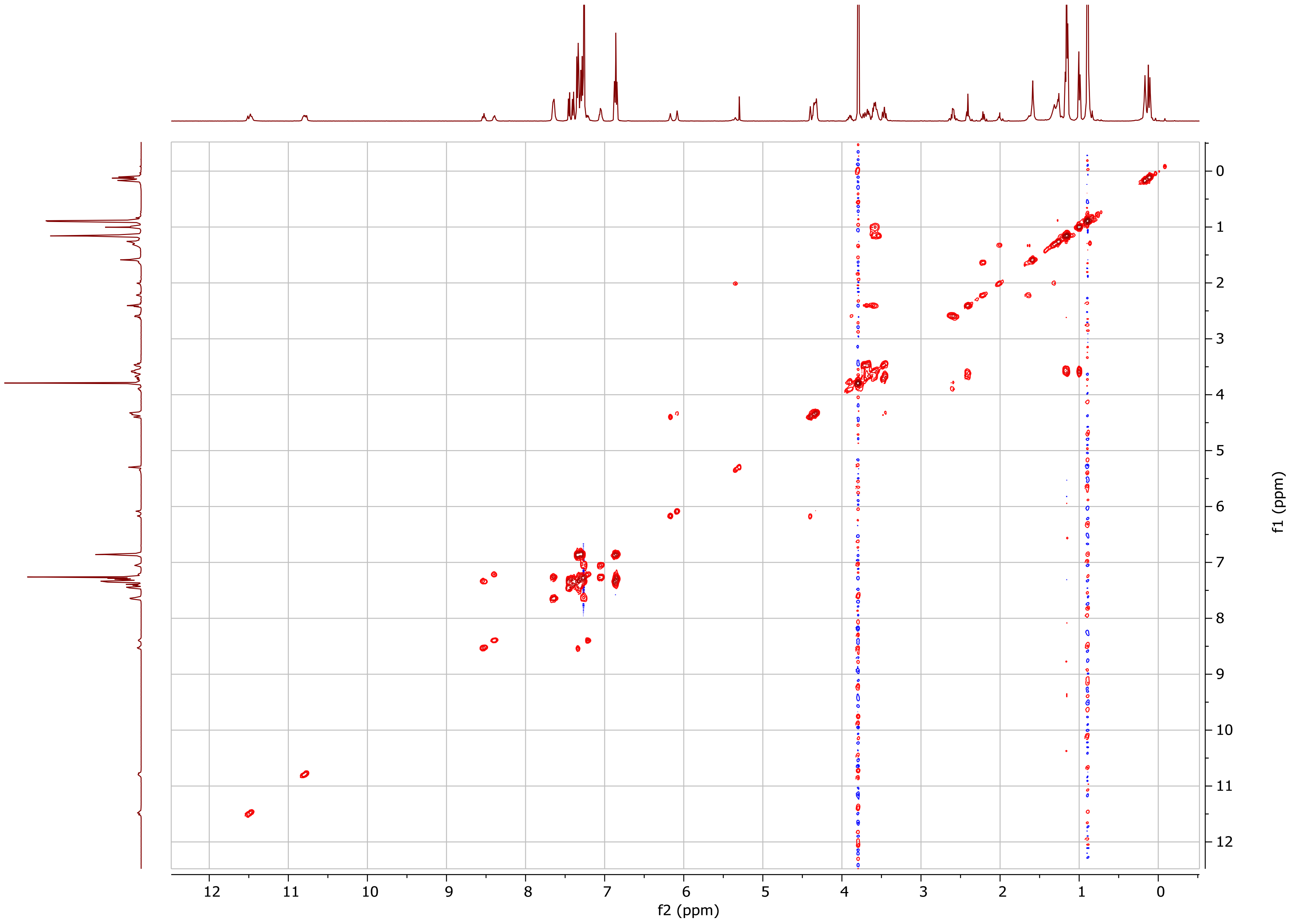

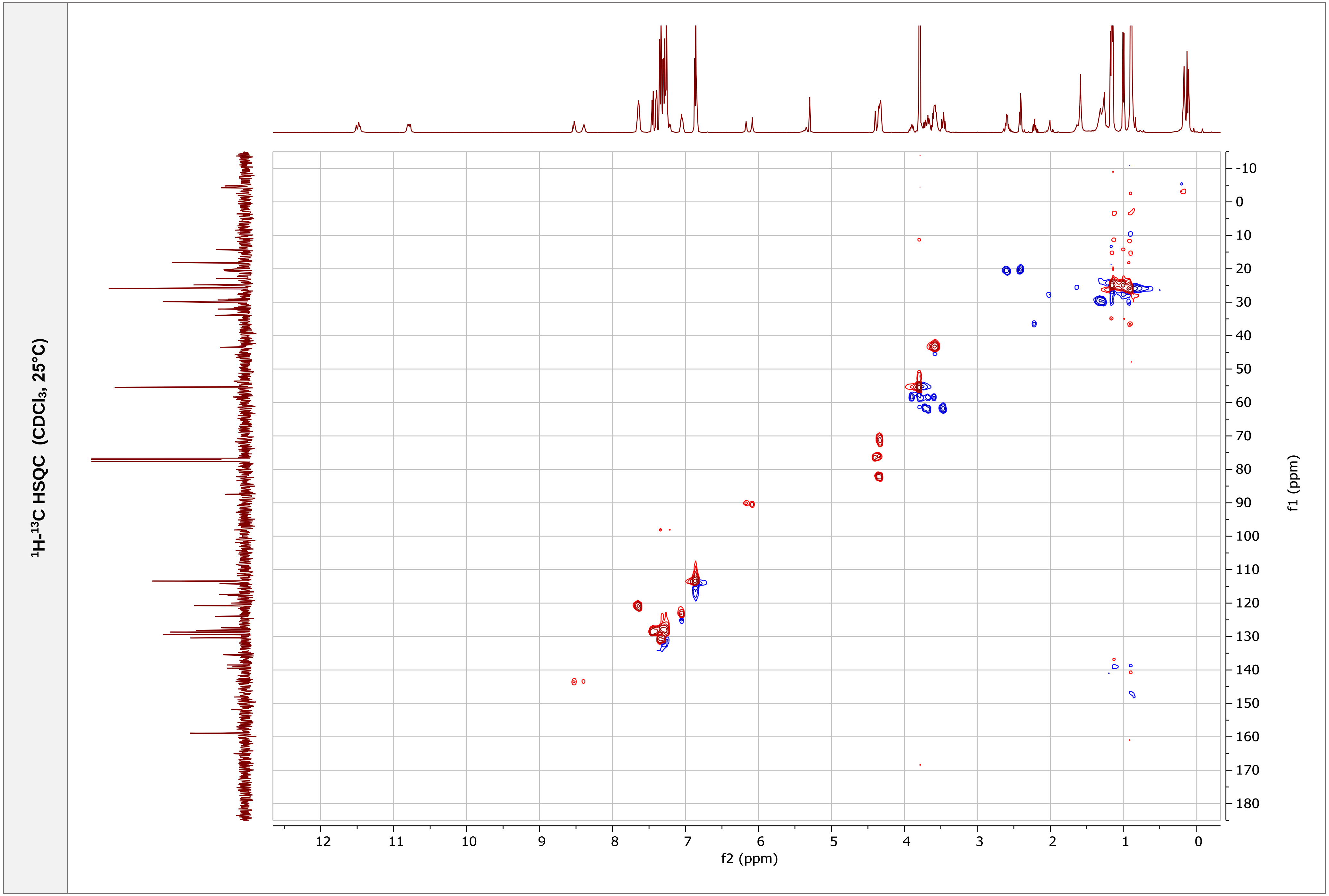

^{1}H-^{13}C HSQC (CDCl$_3$, 25°C)
f2 (ppm)
f1 (ppm)

^{1}H-^{31}P HSQC (CDCl$_3$, 25°C)

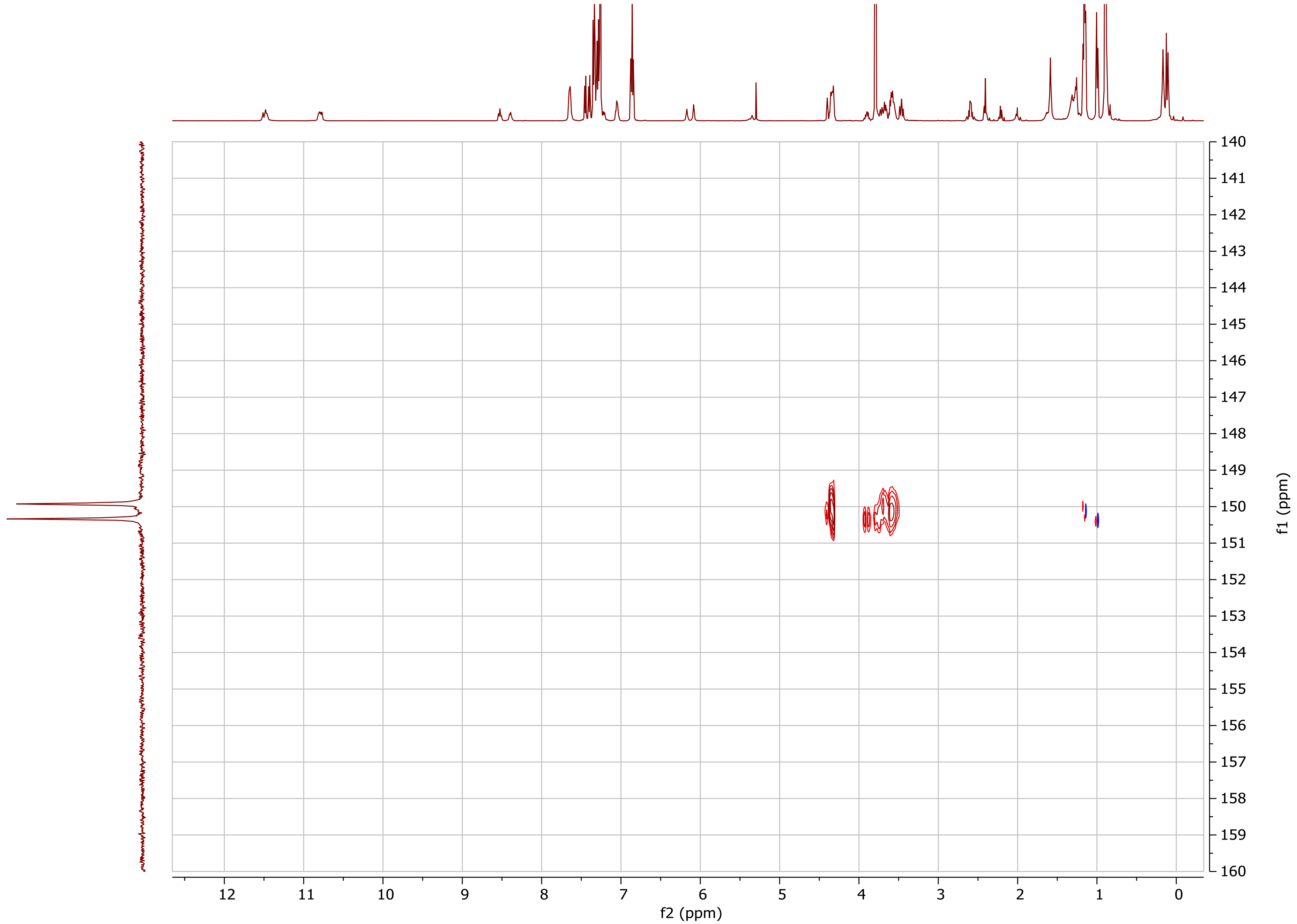

(8a) *N*3-methyluridine phosphoramidite (5′-*O*-DMT-2′-*O*-Me-m^3U_m)

MS (+) ESI
(Calc. $[M+H]^+$ $C_{41}H_{52}N_4O_9P^+$ 775.34664)

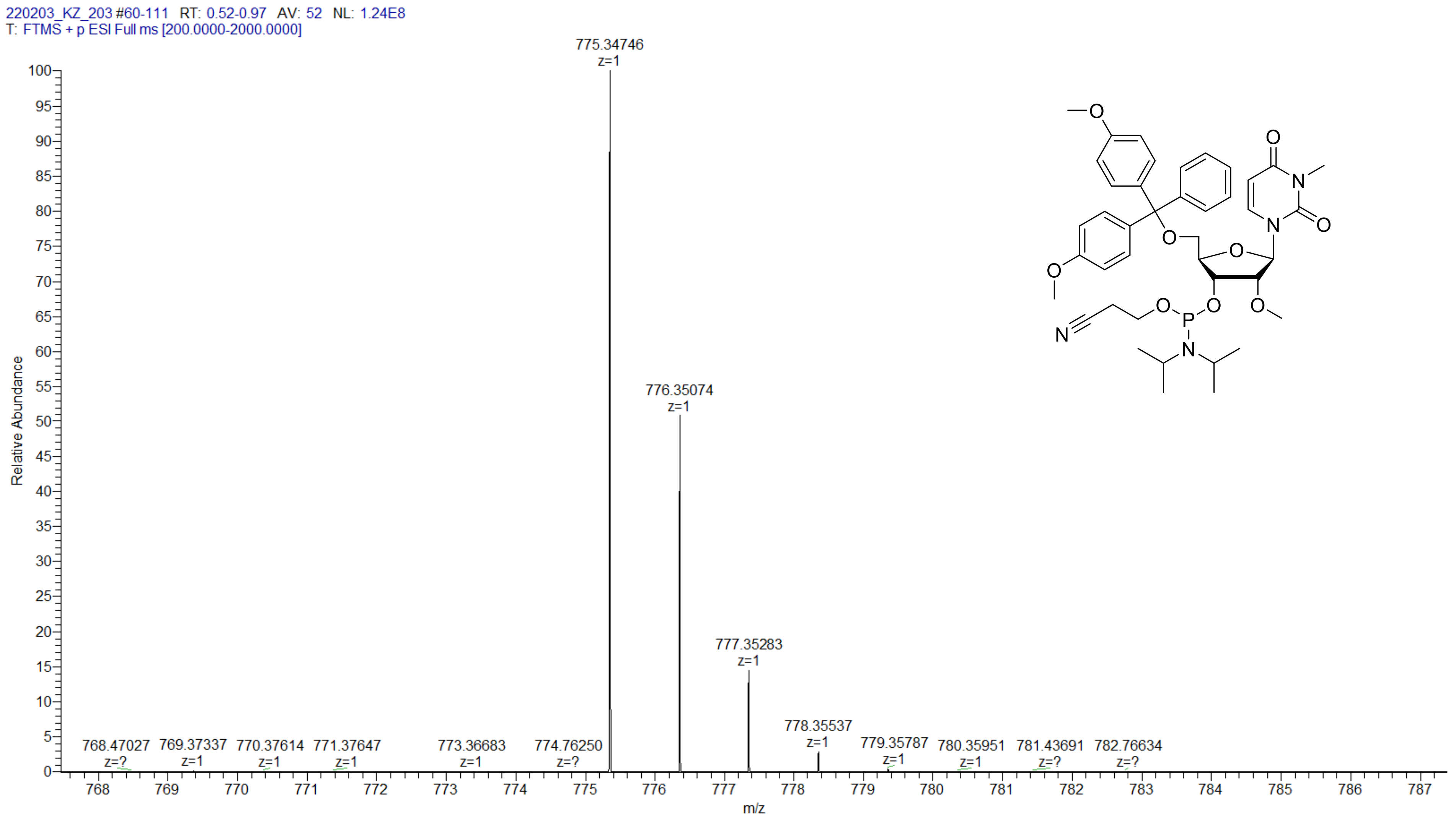

^{1}H NMR (500 MHz, $CDCl_3$, 25°C)

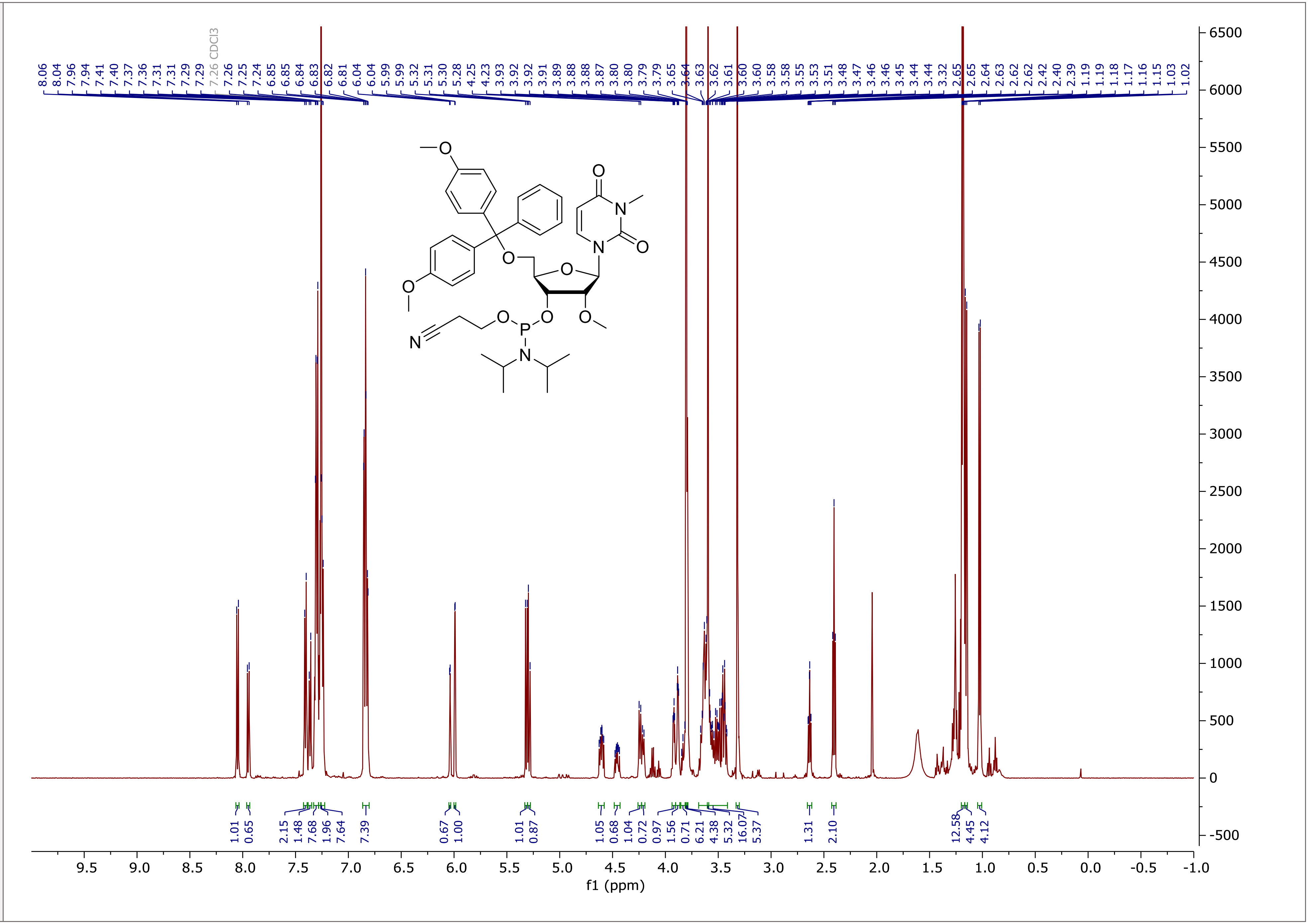

$^{13}C\{^1H\}$ NMR (126 MHz, $CDCl_3$, 25°C)

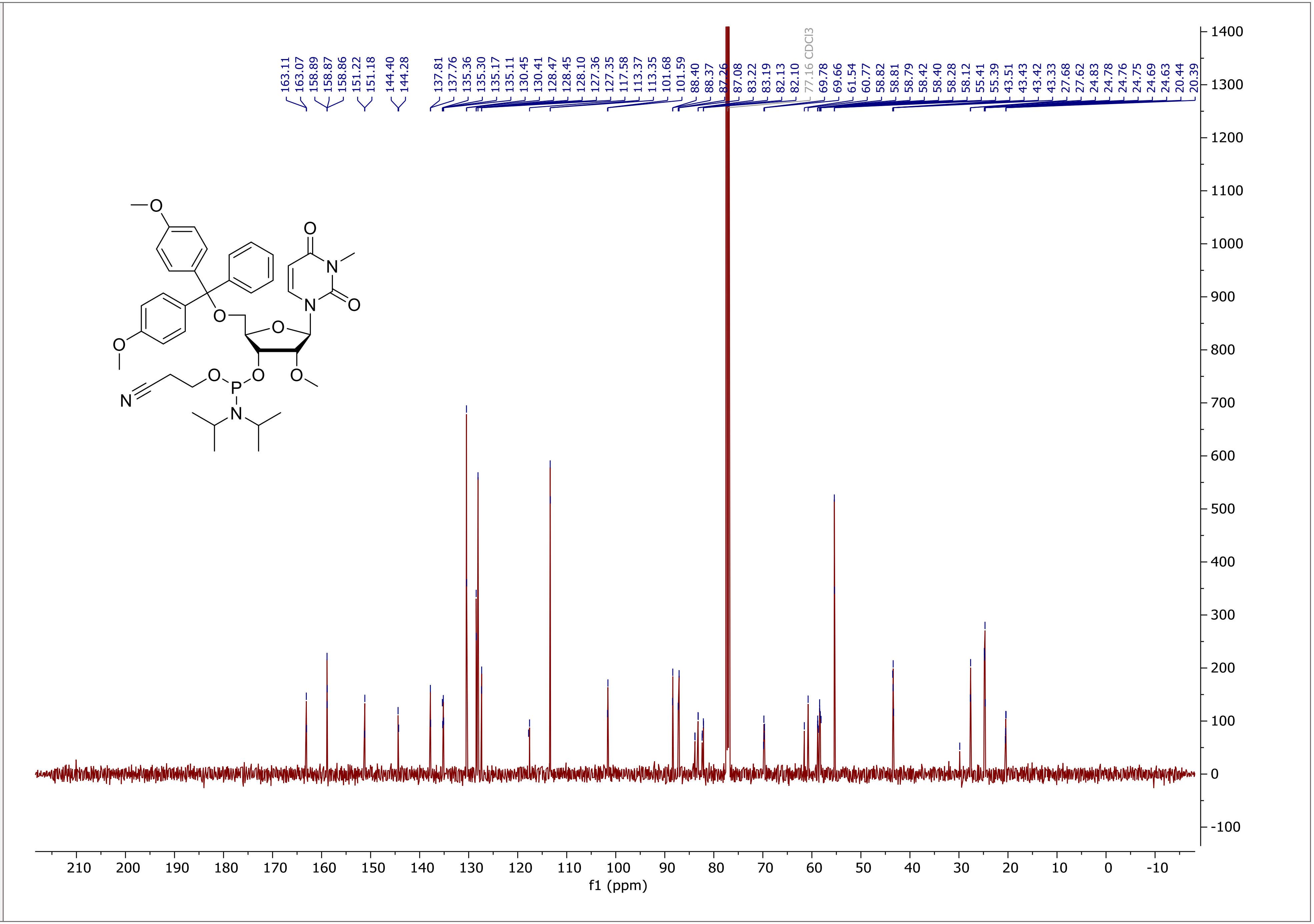

^{31}P NMR (202.5 MHz, $CDCl_3$, 25°C)

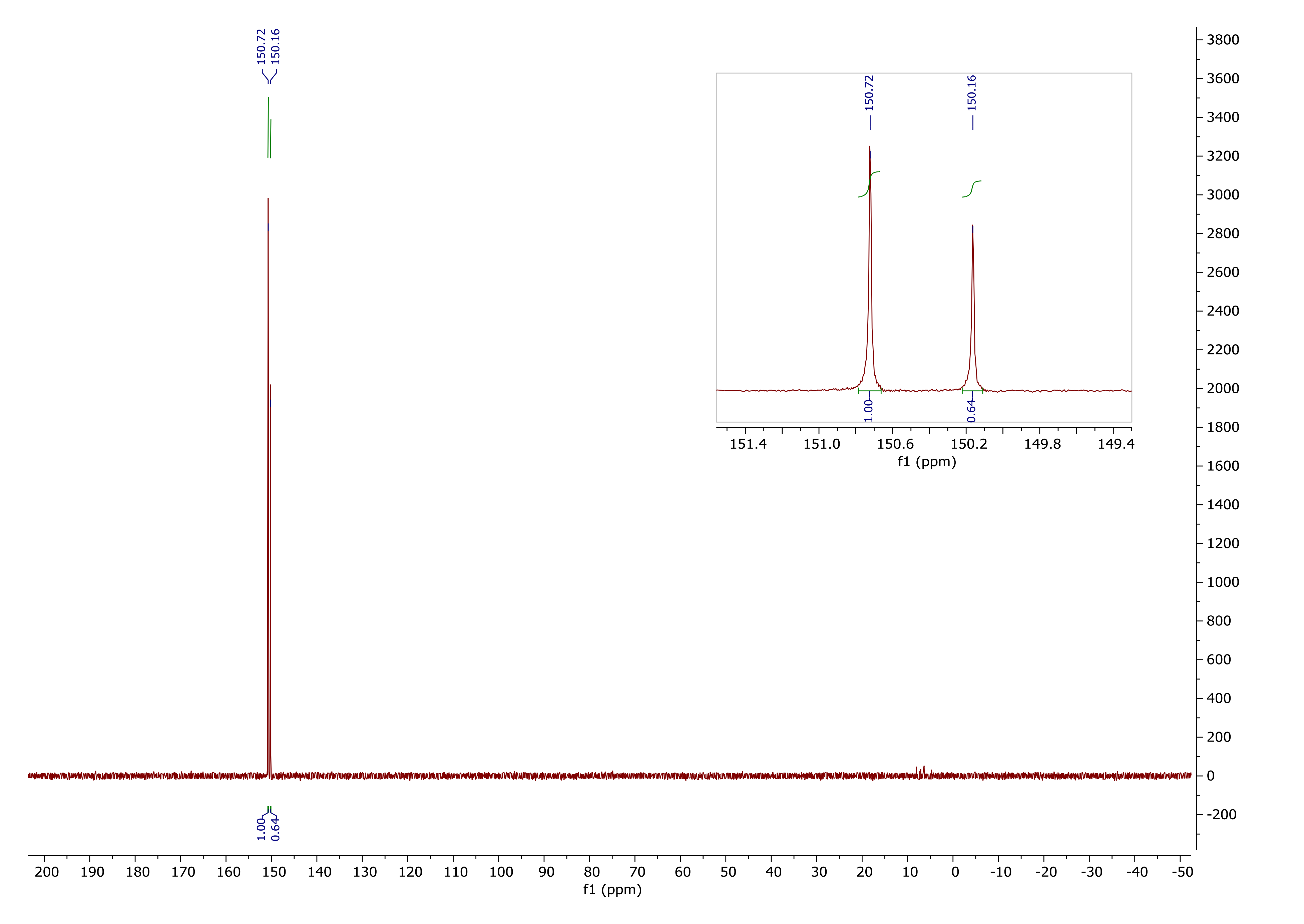

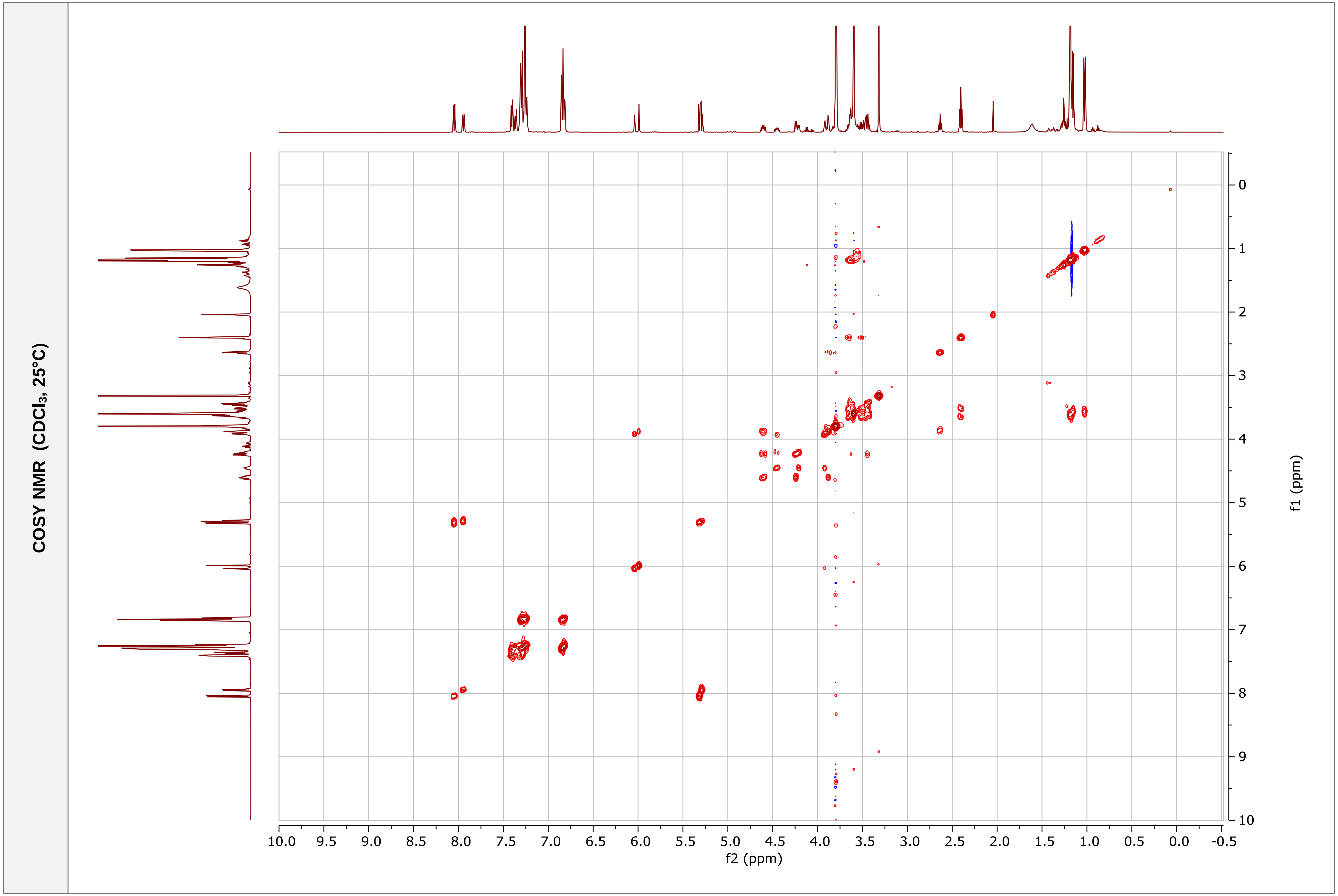
COSY NMR (CDCl3, 25°C)
f2 (ppm)
f1 (ppm)

^{1}H-^{13}C HSQC (CDCl$_3$, 25°C)

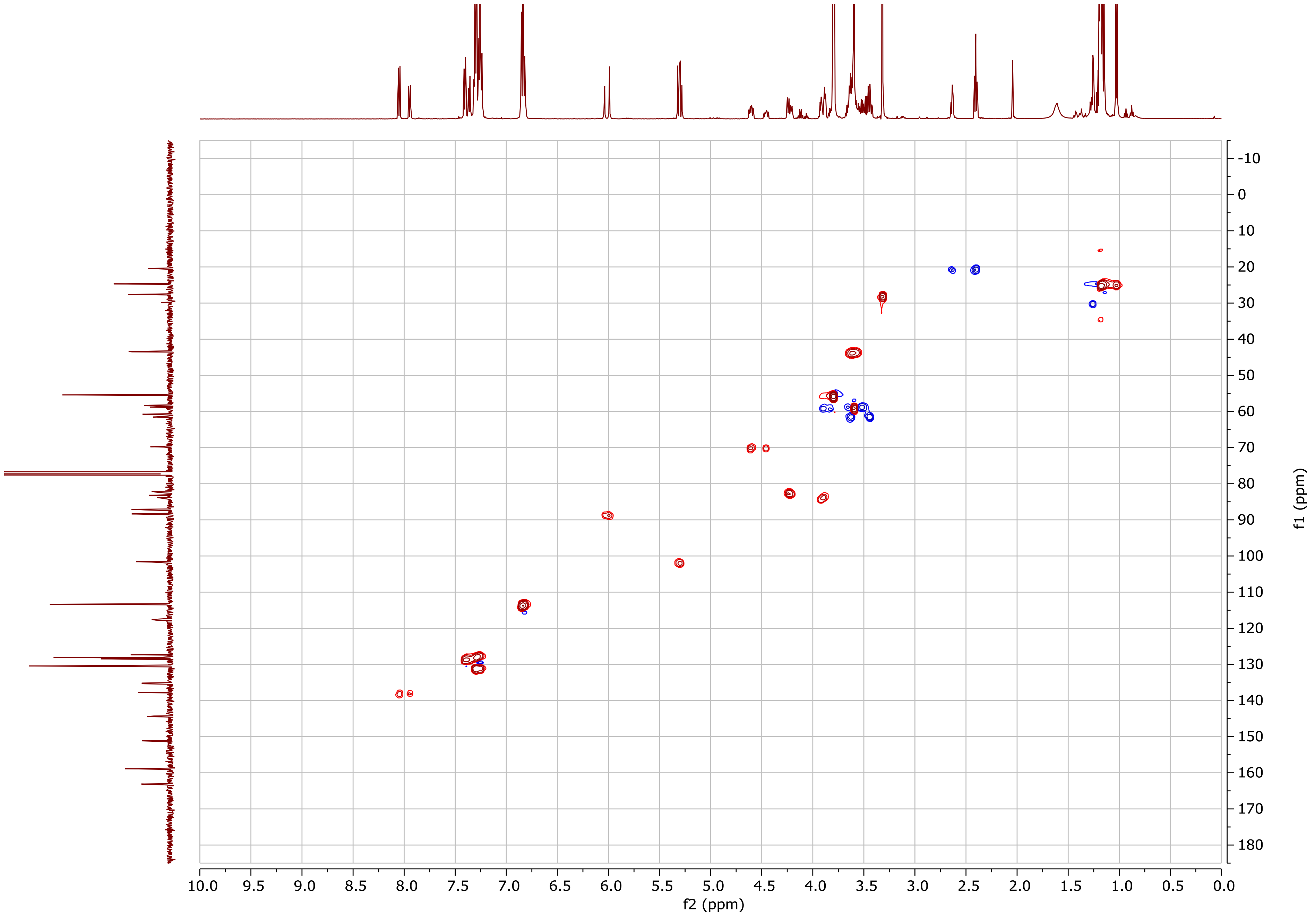

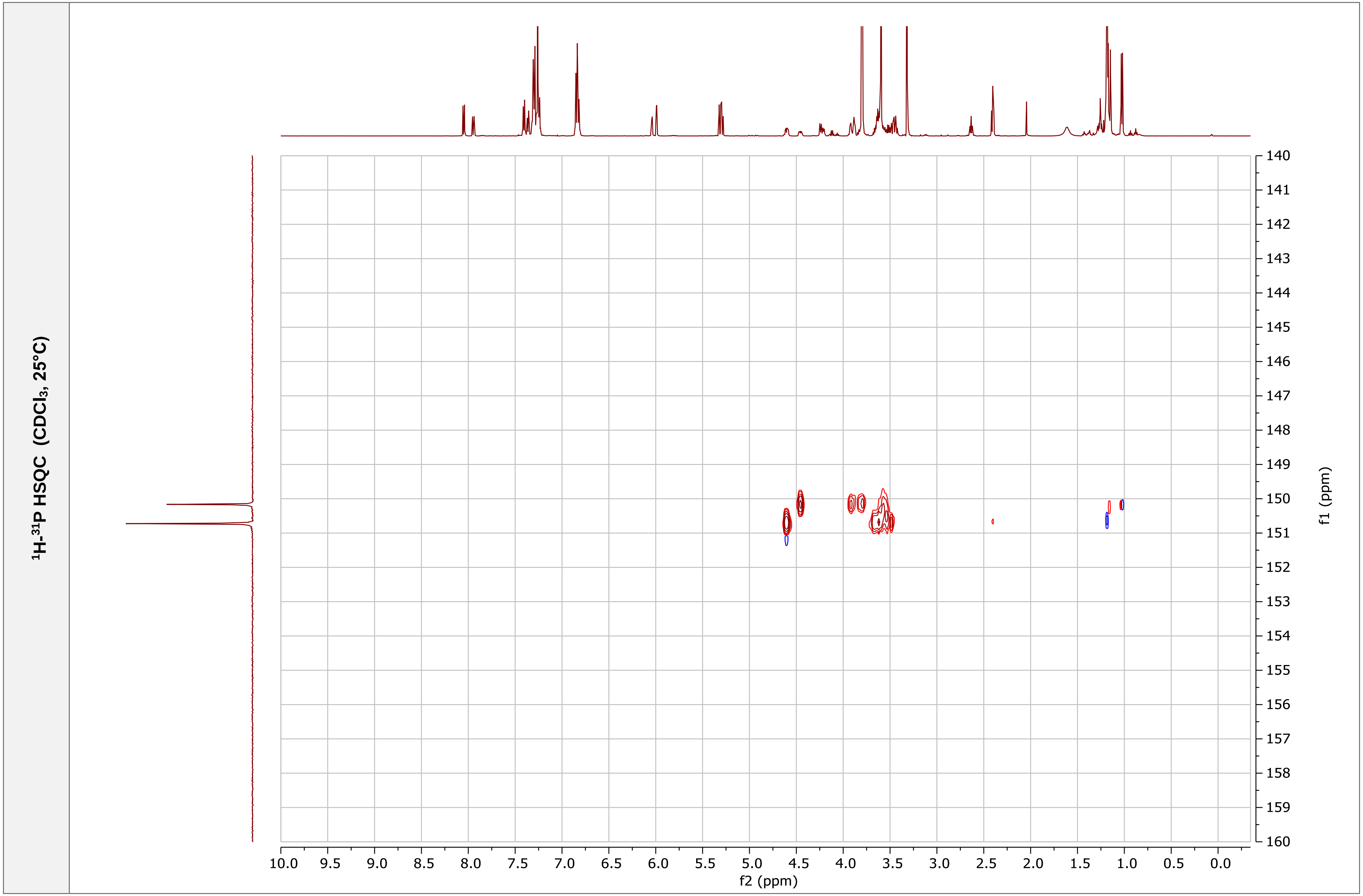
^{1}H-^{31}P HSQC (CDCl$_3$, 25°C)
f2 (ppm)
f1 (ppm)

(8b) *N*3-(2-nitrobenzyl)thymidine phosphoramidite (5′-*O*-DMT- nb^{3}T)

MS (+) ESI

(Calc. $[M+H]^+$ $C_{47}H_{55}N_5O_{10}P^+$ 880.36811)

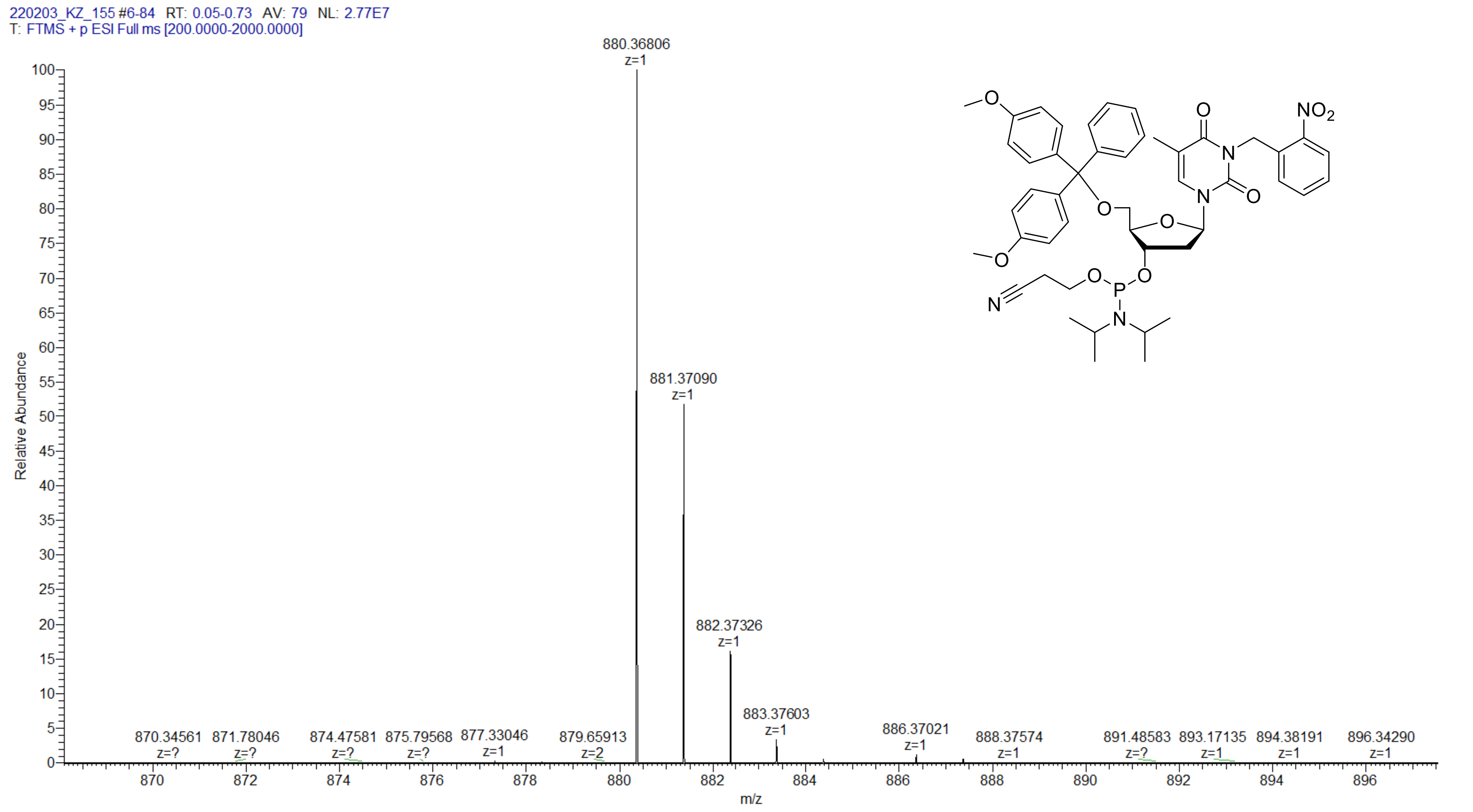

^{1}H NMR (500 MHz, CDCl$_3$, 25°C)

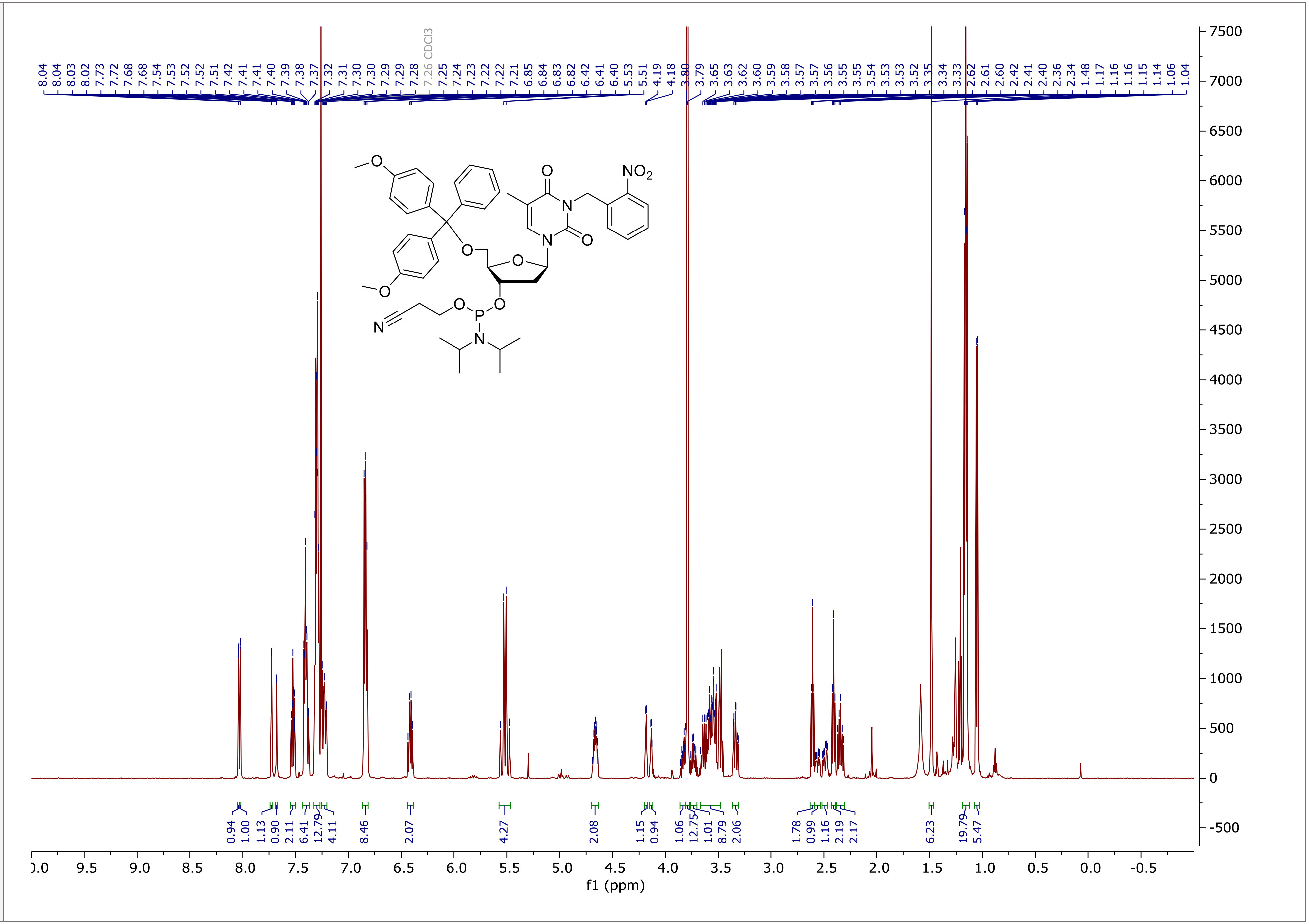

$^{13}C\{^1H\}$ NMR (126 MHz, $CDCl_3$, 25°C)

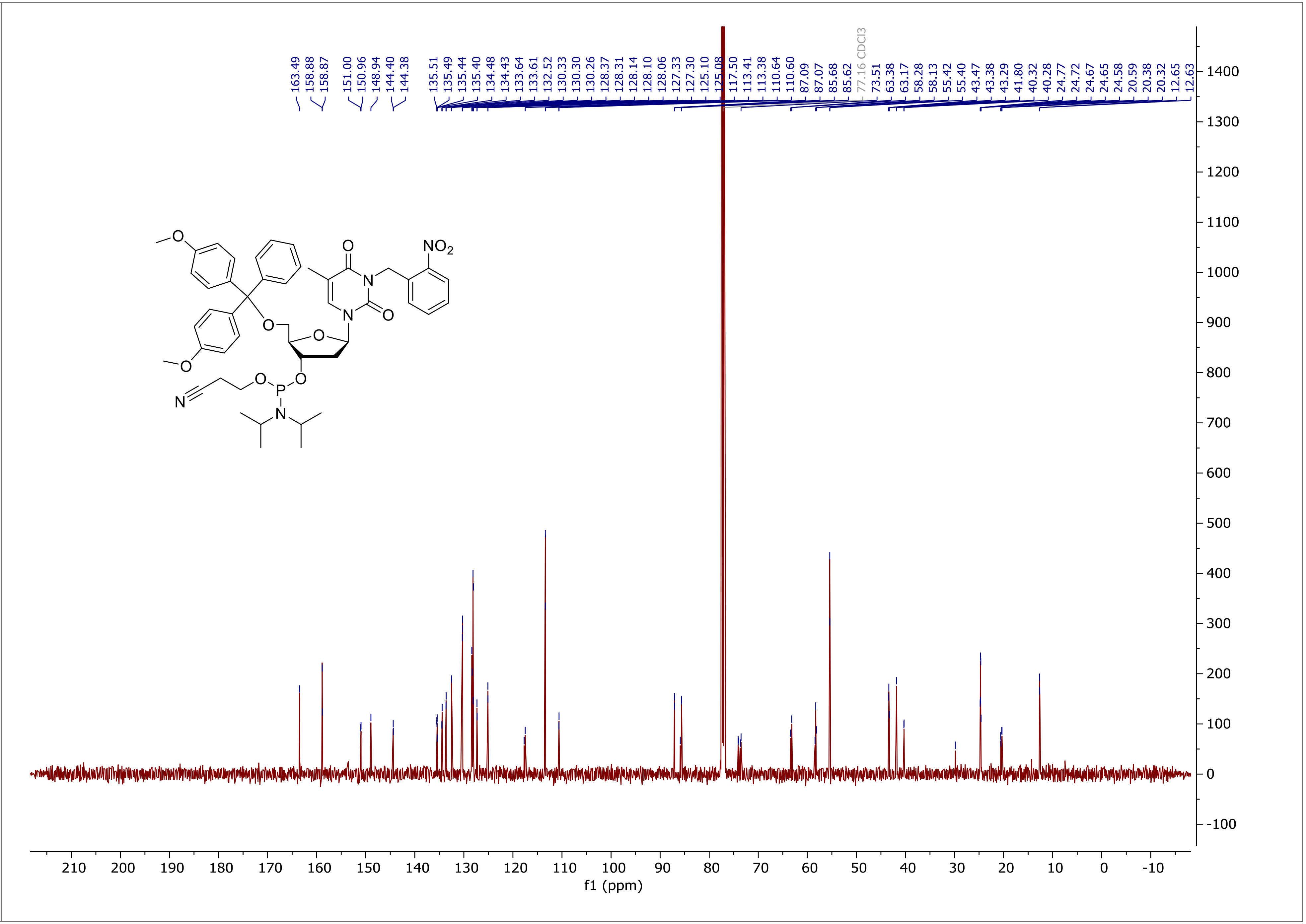

^{1}P NMR (202.5 MHz, $CDCl_3$, 25°C)

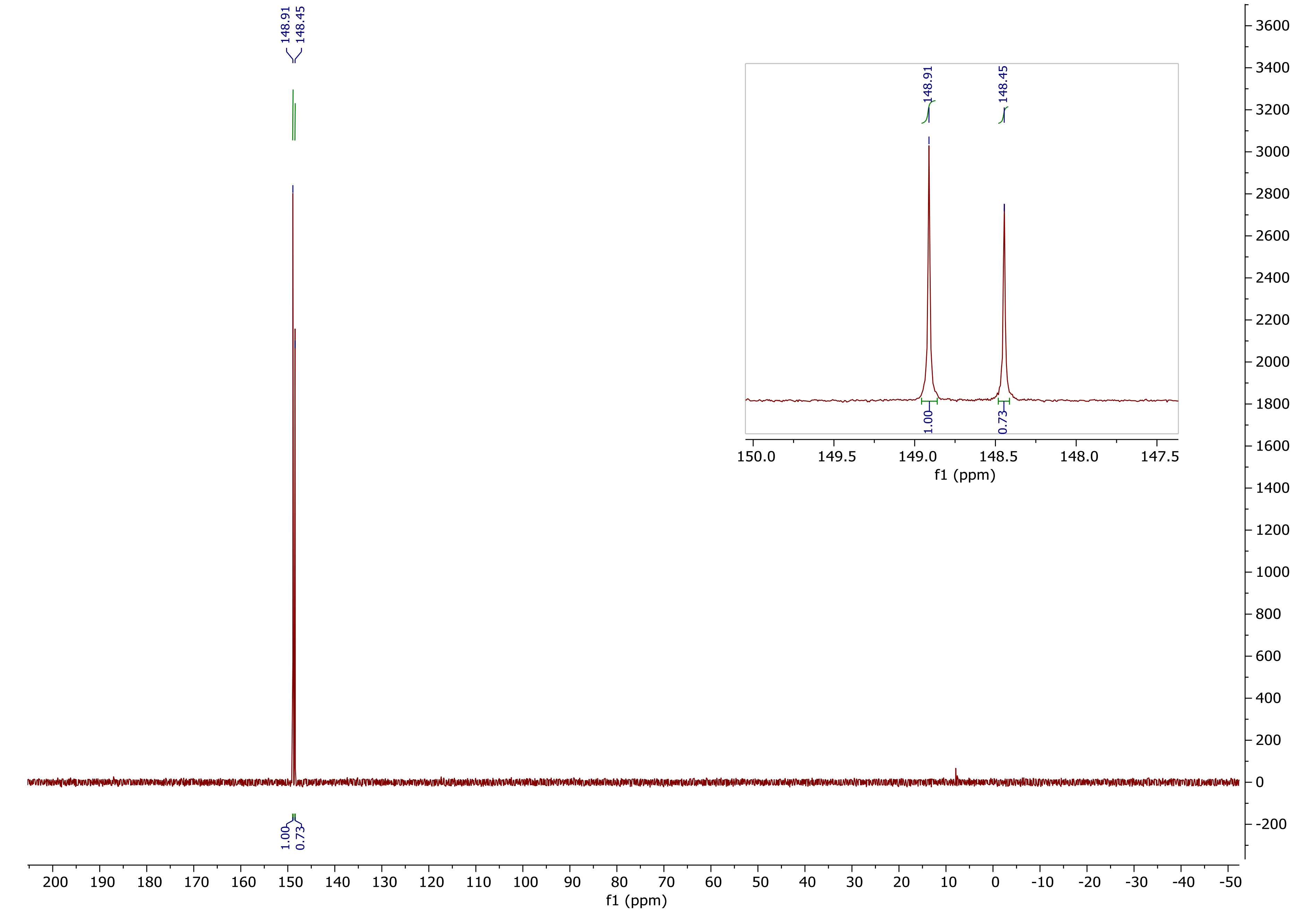

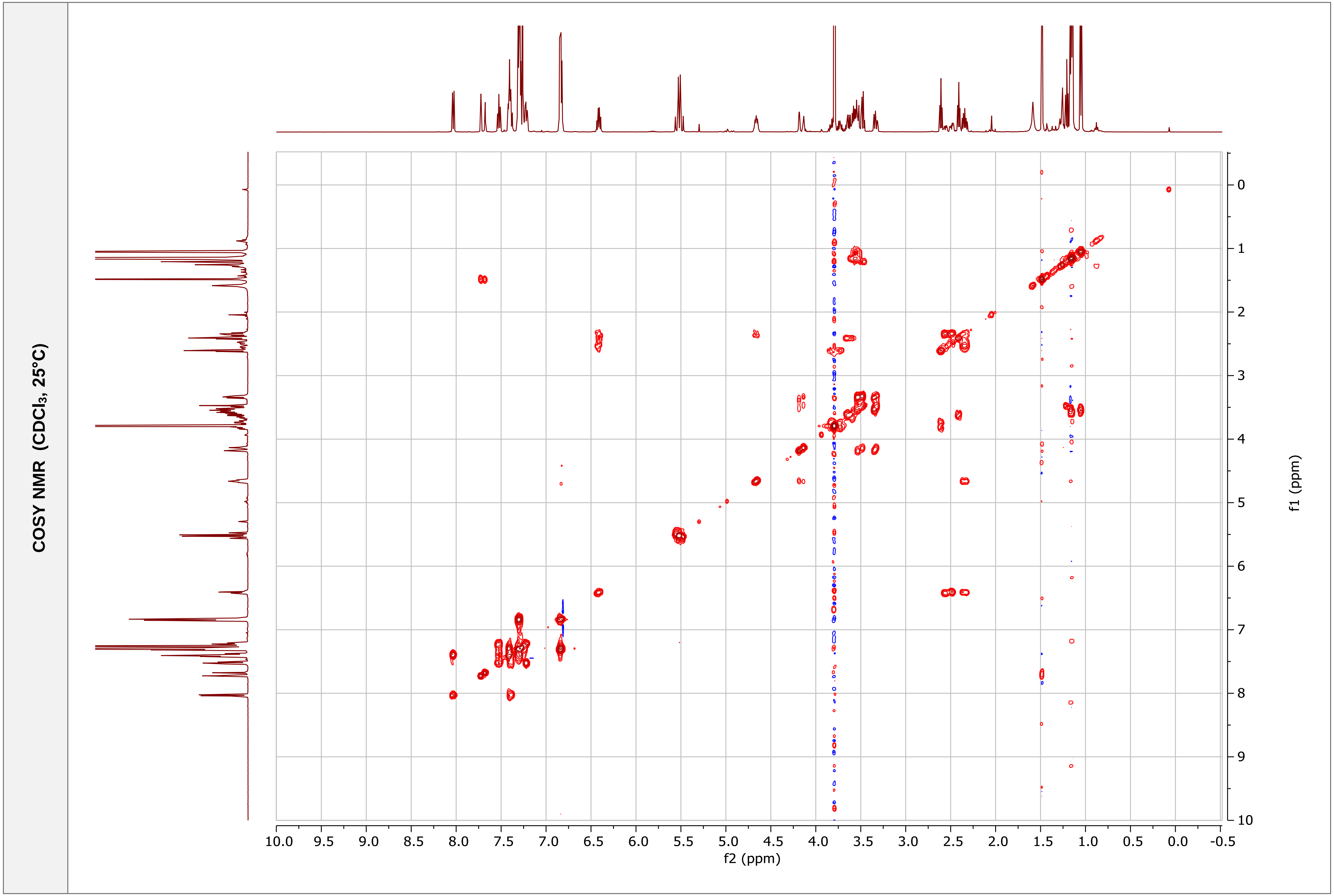

COSY NMR (CDCl$_3$, 25°C)
f2 (ppm)
f1 (ppm)

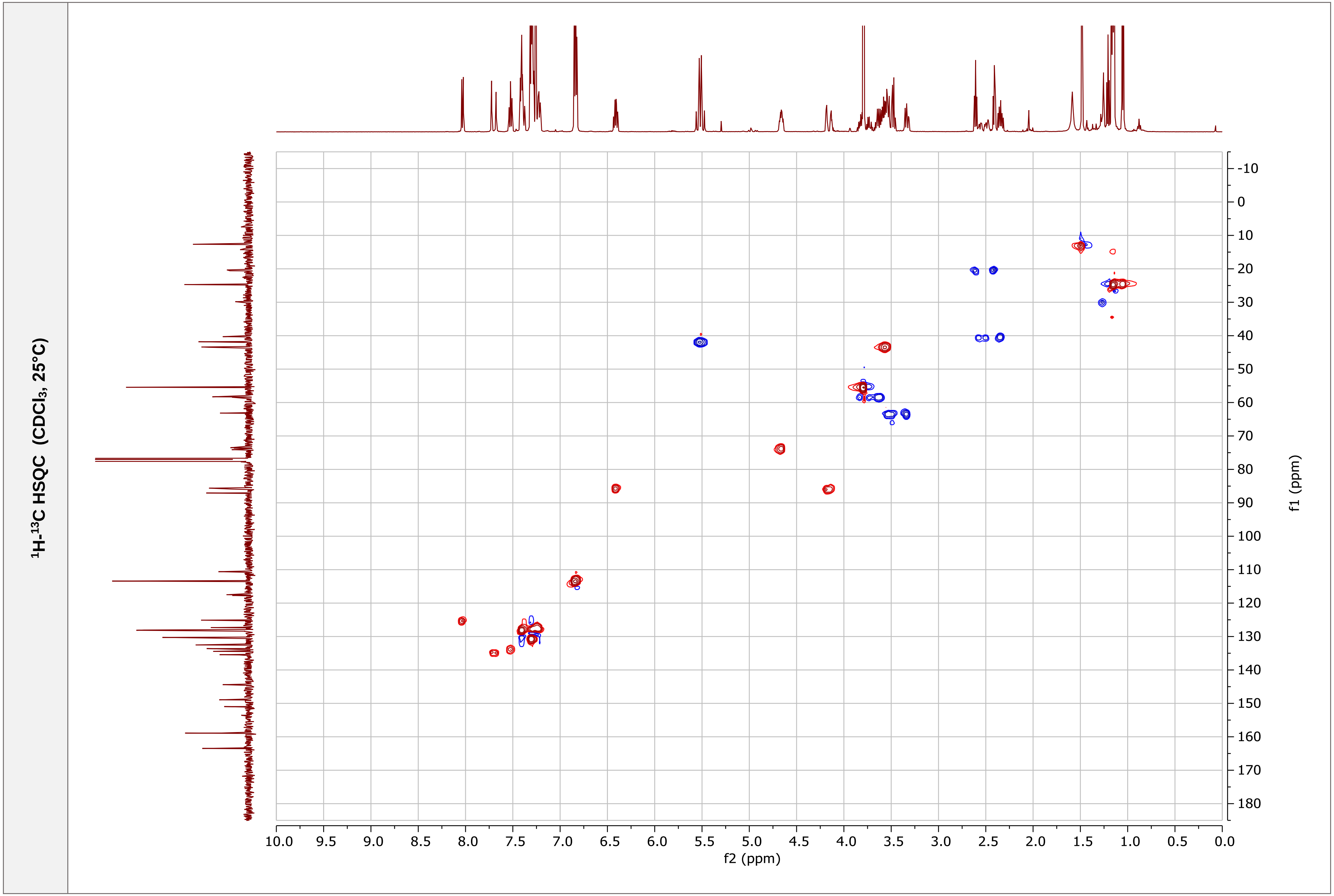

^{1}H-^{13}C HSQC (CDCl$_3$, 25°C)
f2 (ppm)
f1 (ppm)

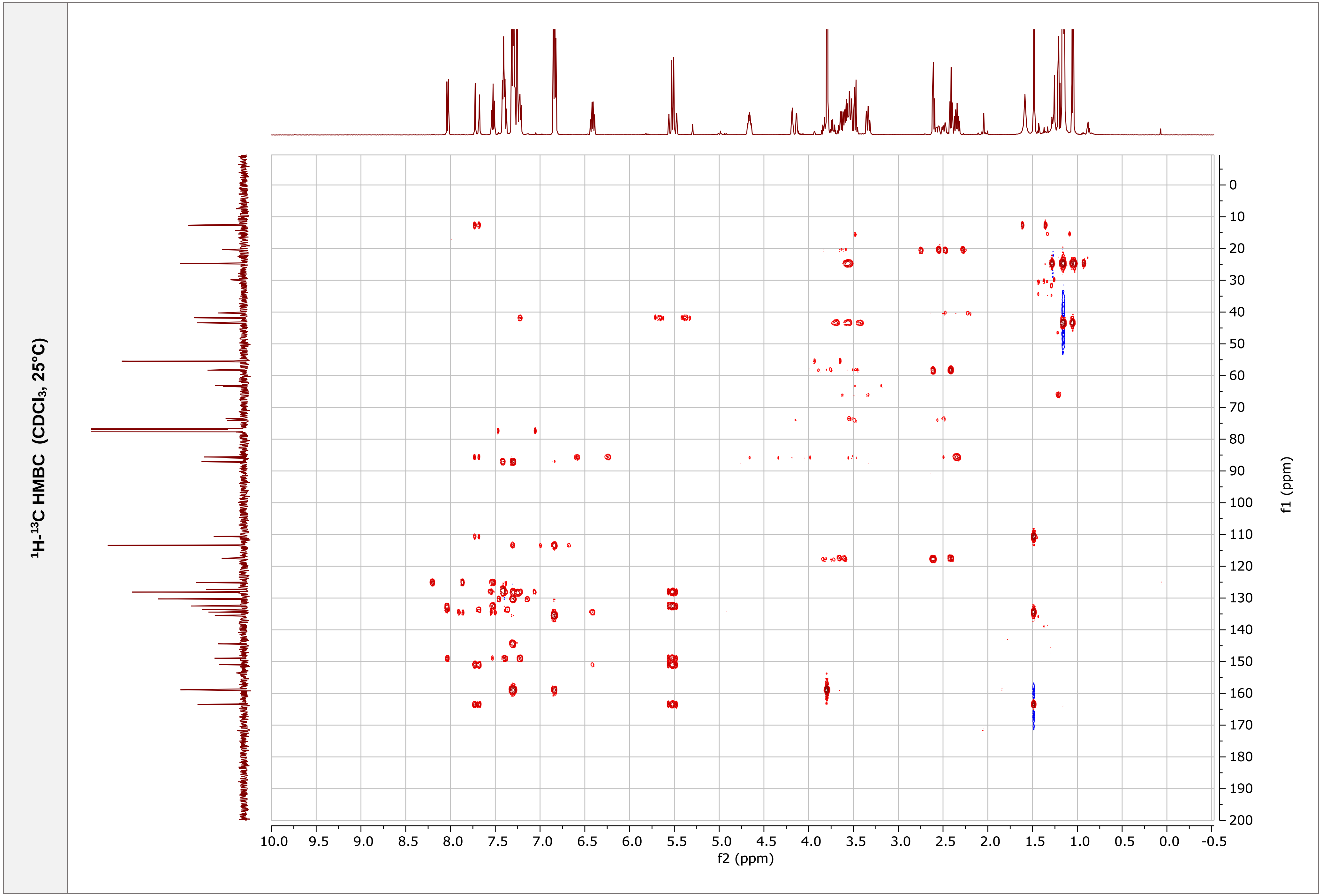

^{1}H-^{13}C HMBC (CDCl$_3$, 25°C)
f2 (ppm)
f1 (ppm)

(9) *N*1-(4-*O*-acetyl)benzyl-*N*2-methylguanosine phosphoramidite (5′-*O*-DMT-2′-*O*-TBDMS-(4-OAc)Bn^{1}m^{2}G^{iBu})

MS (+) ESI

(Calc. $[M+H]^+$ $C_{60}H_{79}N_7O_{11}PSI^+$ 1132.53390)

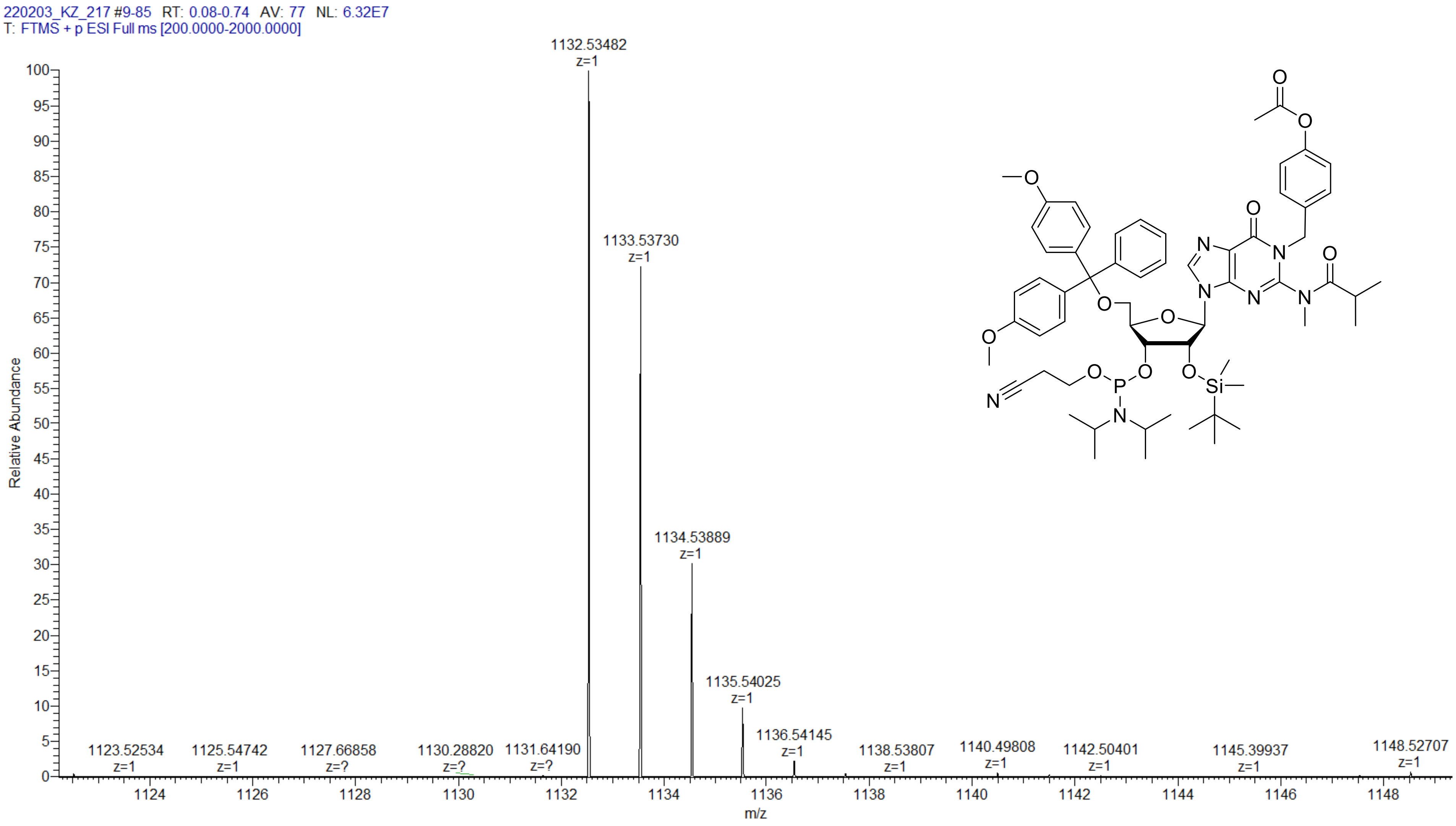

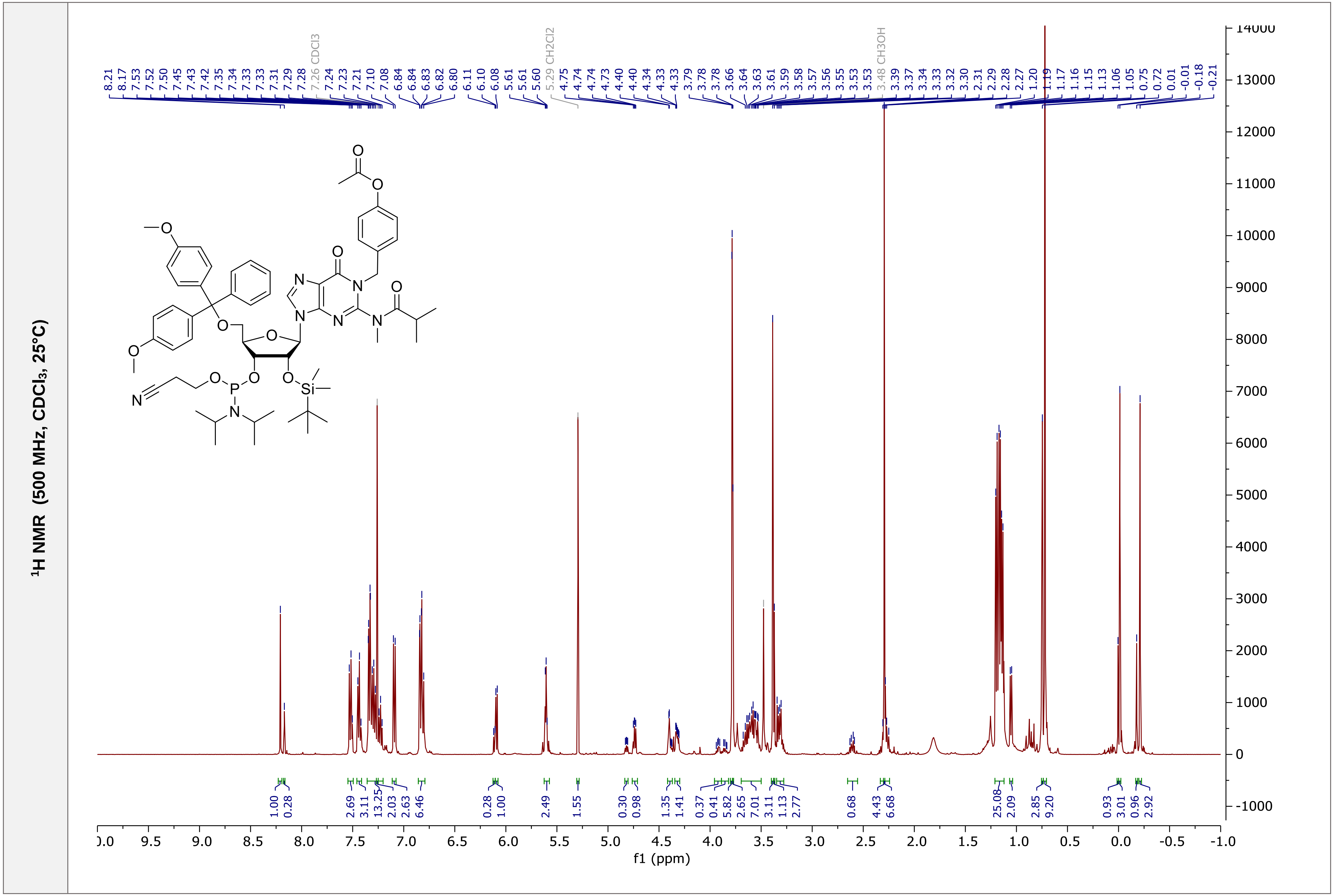

1H NMR (500 MHz, CDCl3, 25°C)
f1 (ppm)
7.26 CDCl3
5.29 CH2Cl2
3.48 CH3OH

$^{13}C\{^1H\}$ NMR (126 MHz, $CDCl_3$, 25°C)

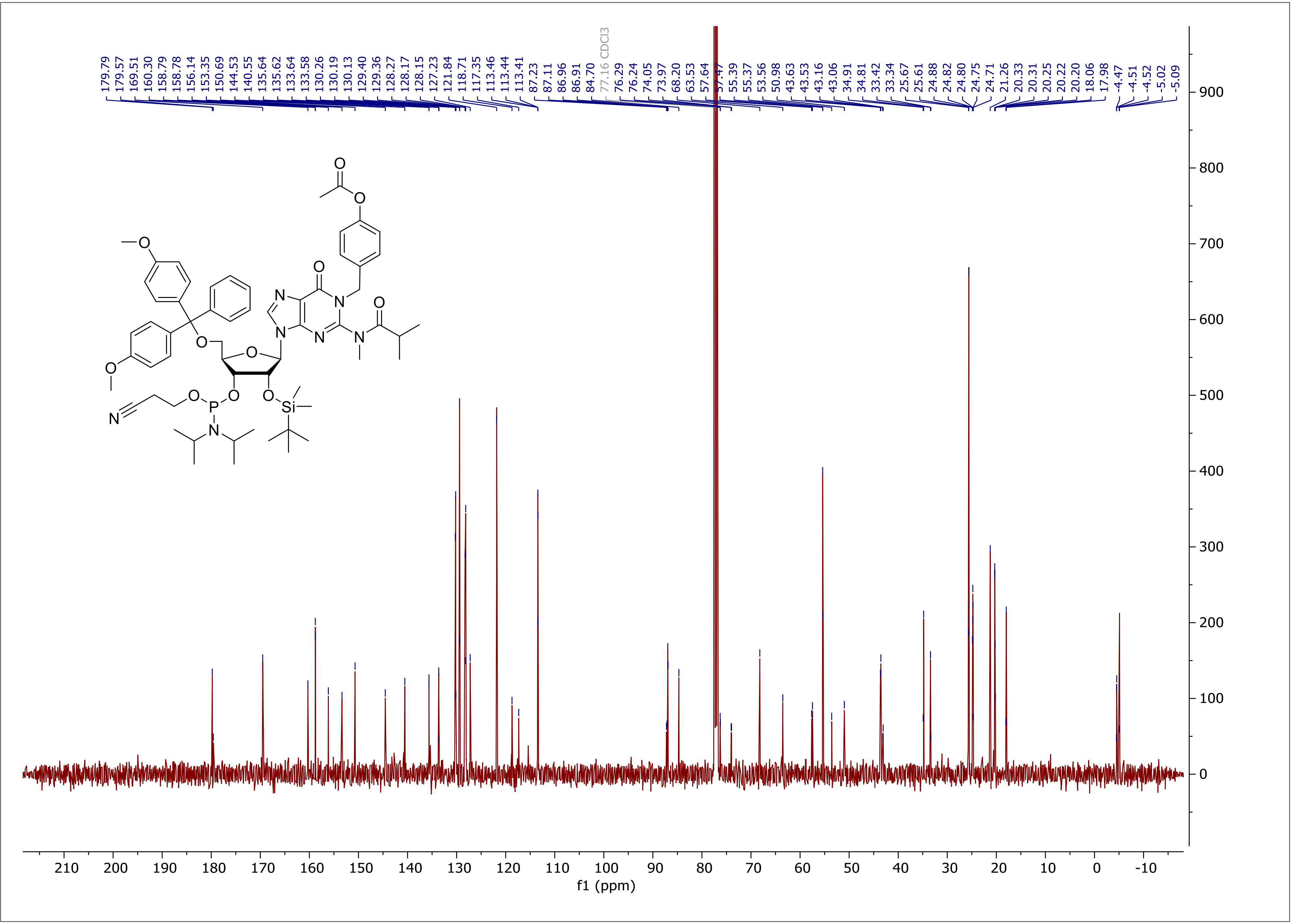

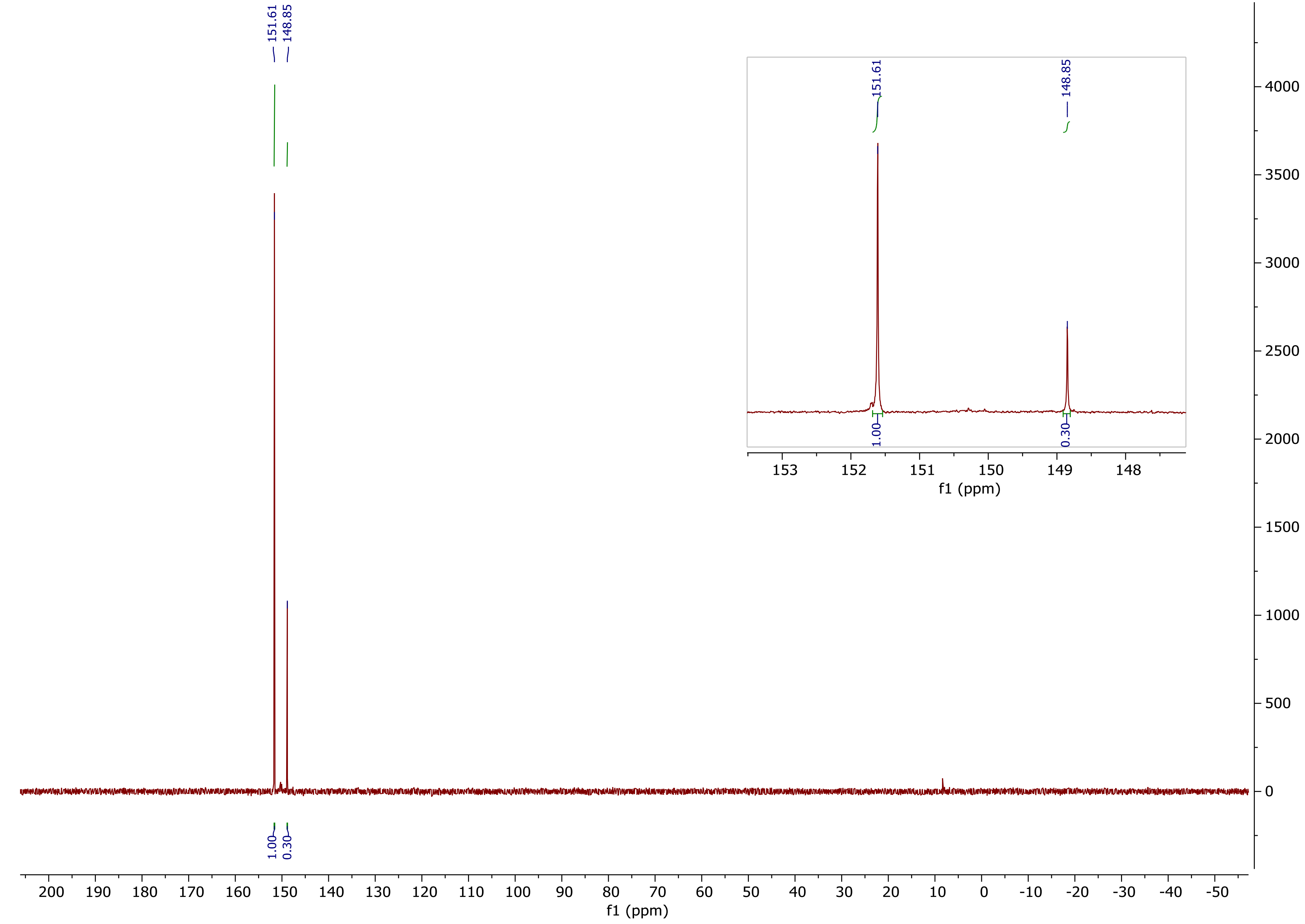

^{31}P NMR (202.5 MHz, $CDCl_3$, 25°C)

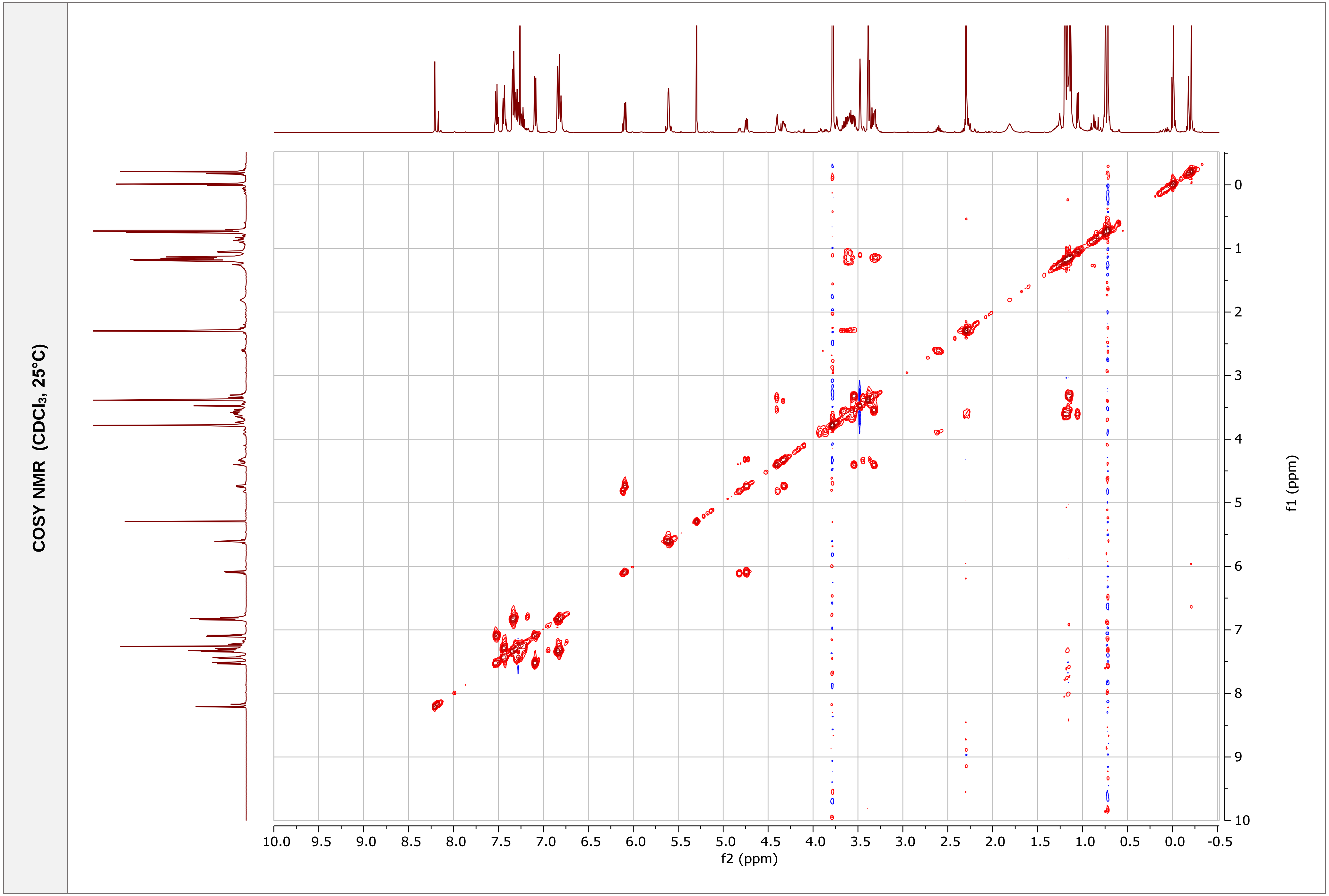
COSY NMR (CDCl$_3$, 25°C)
f2 (ppm)
f1 (ppm)

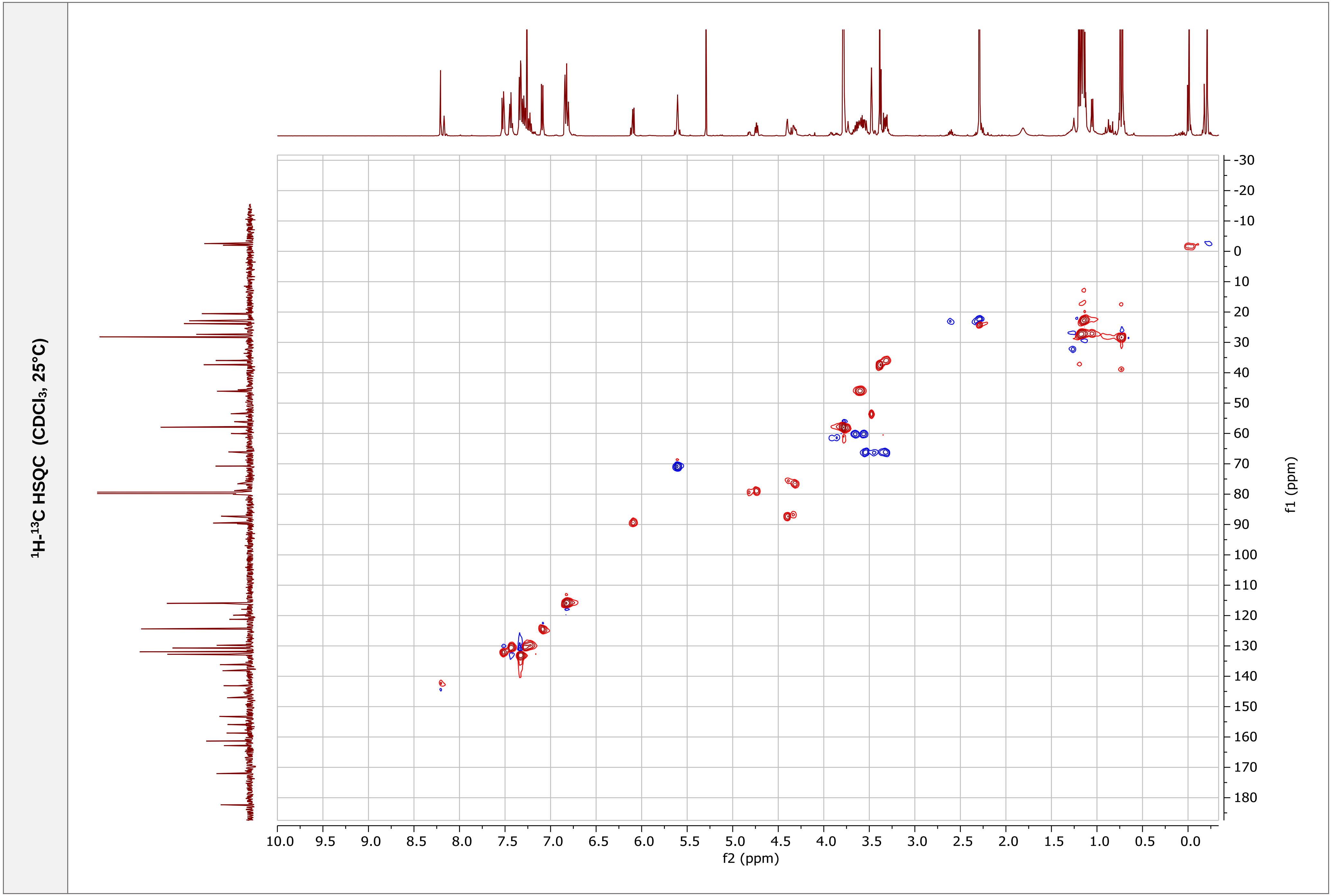

^{1}H-^{13}C HSQC (CDCl$_3$, 25°C)
f2 (ppm)
f1 (ppm)

^1H-^{13}C HMBC (CDCl$_3$, 25°C)

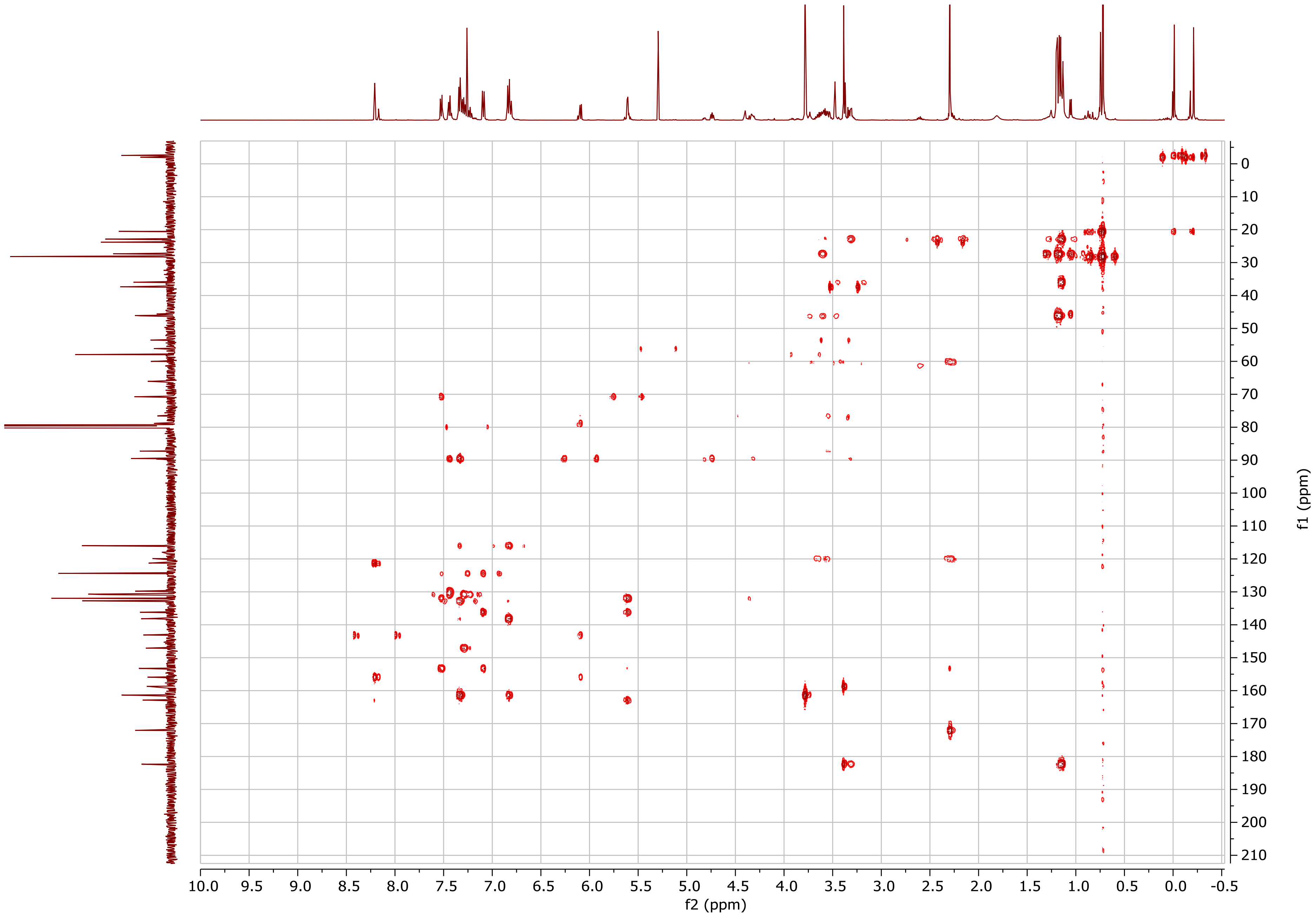

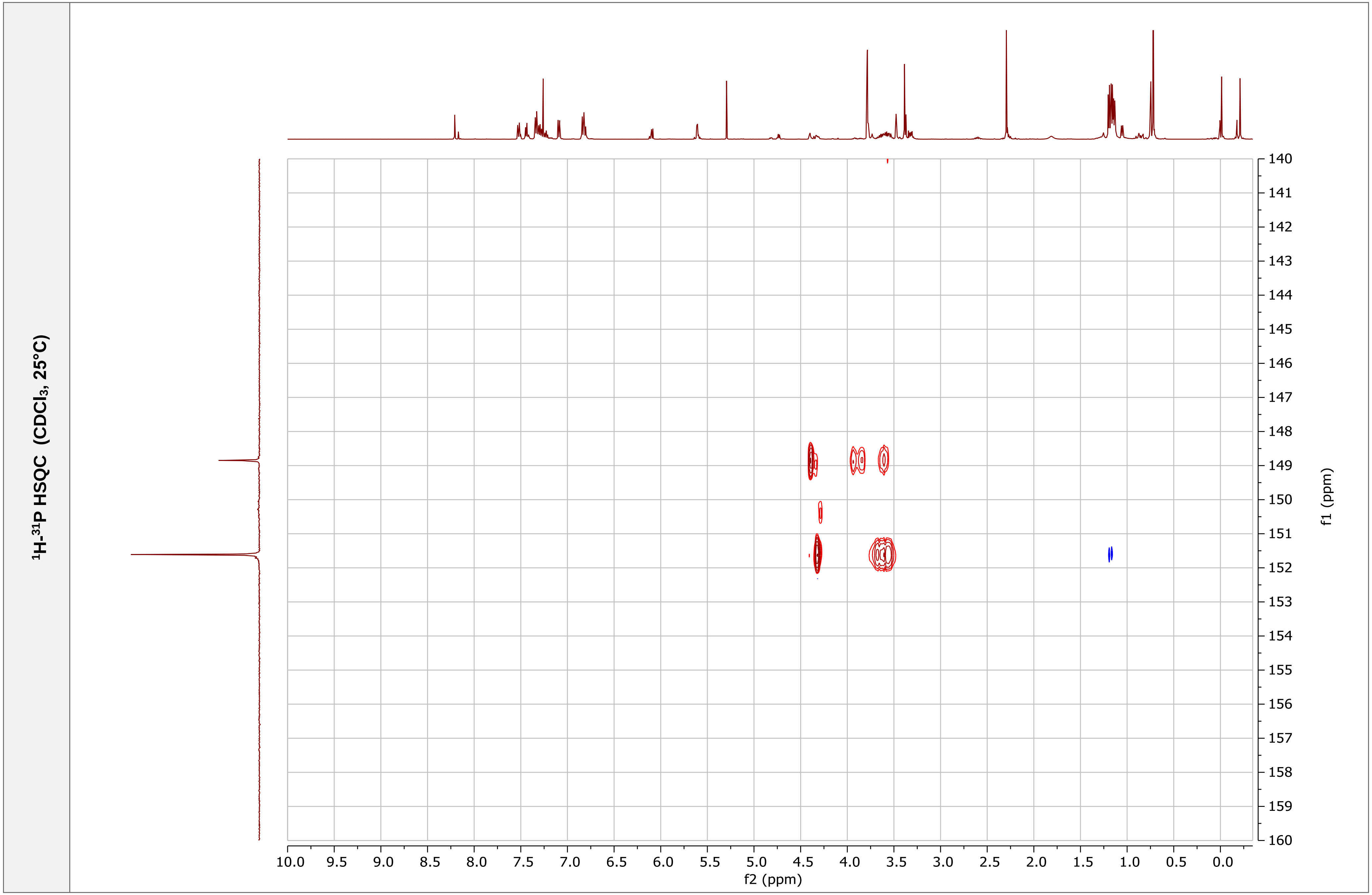

^{1}H-^{31}P HSQC (CDCl$_3$, 25°C)
f2 (ppm)
f1 (ppm)

(10) *N*1-methylguanosine phosphoramidite (5′-*O*-DMT-2′-*O*-TBDMS-m^{1}G^{dmf})

MS (+) ESI

(Calc. $[M+H]^+$ $C_{50}H_{70}N_8O_8PSi^+$ 969.48180)

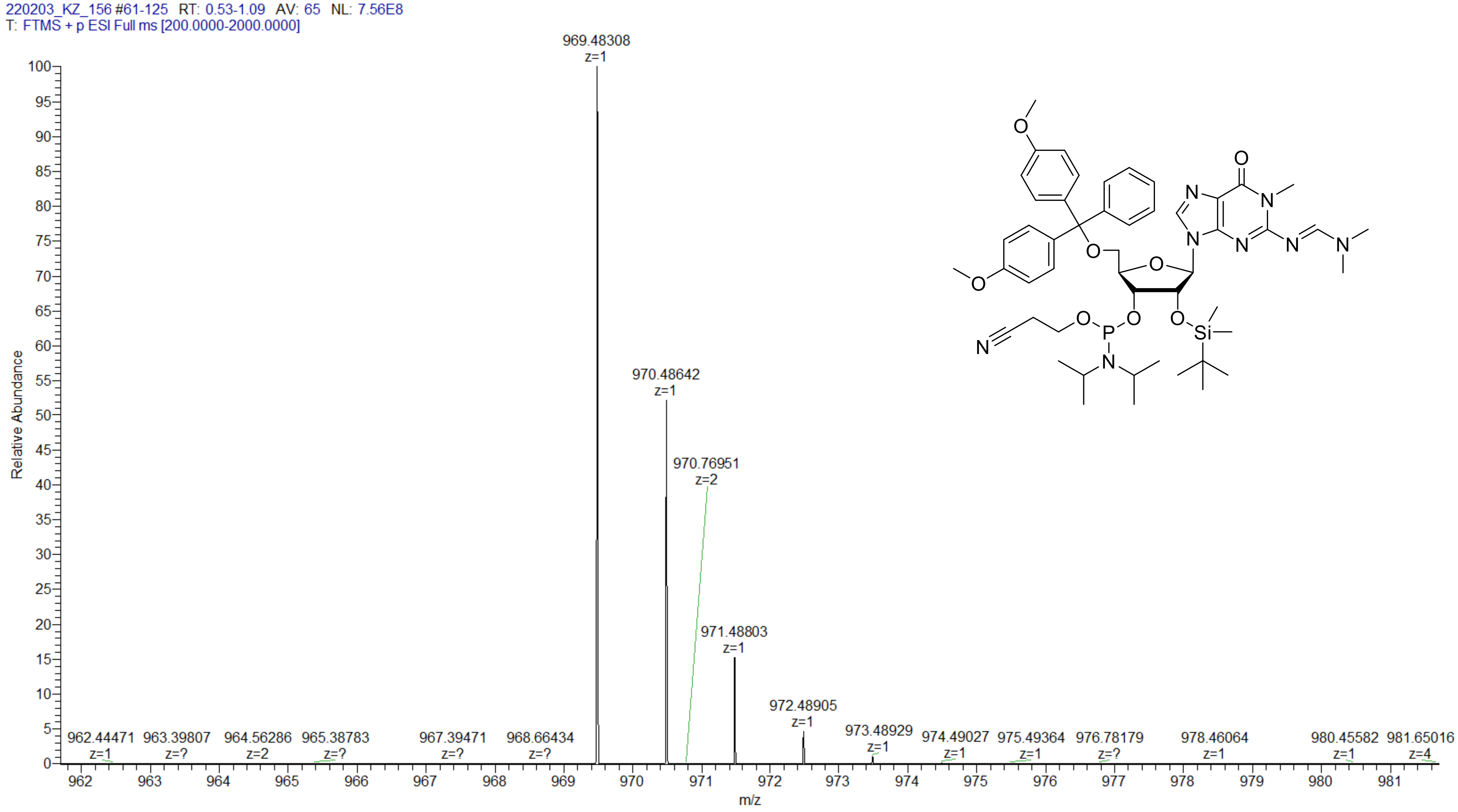

^{1}H NMR (500 MHz, $CDCl_3$, 25°C)

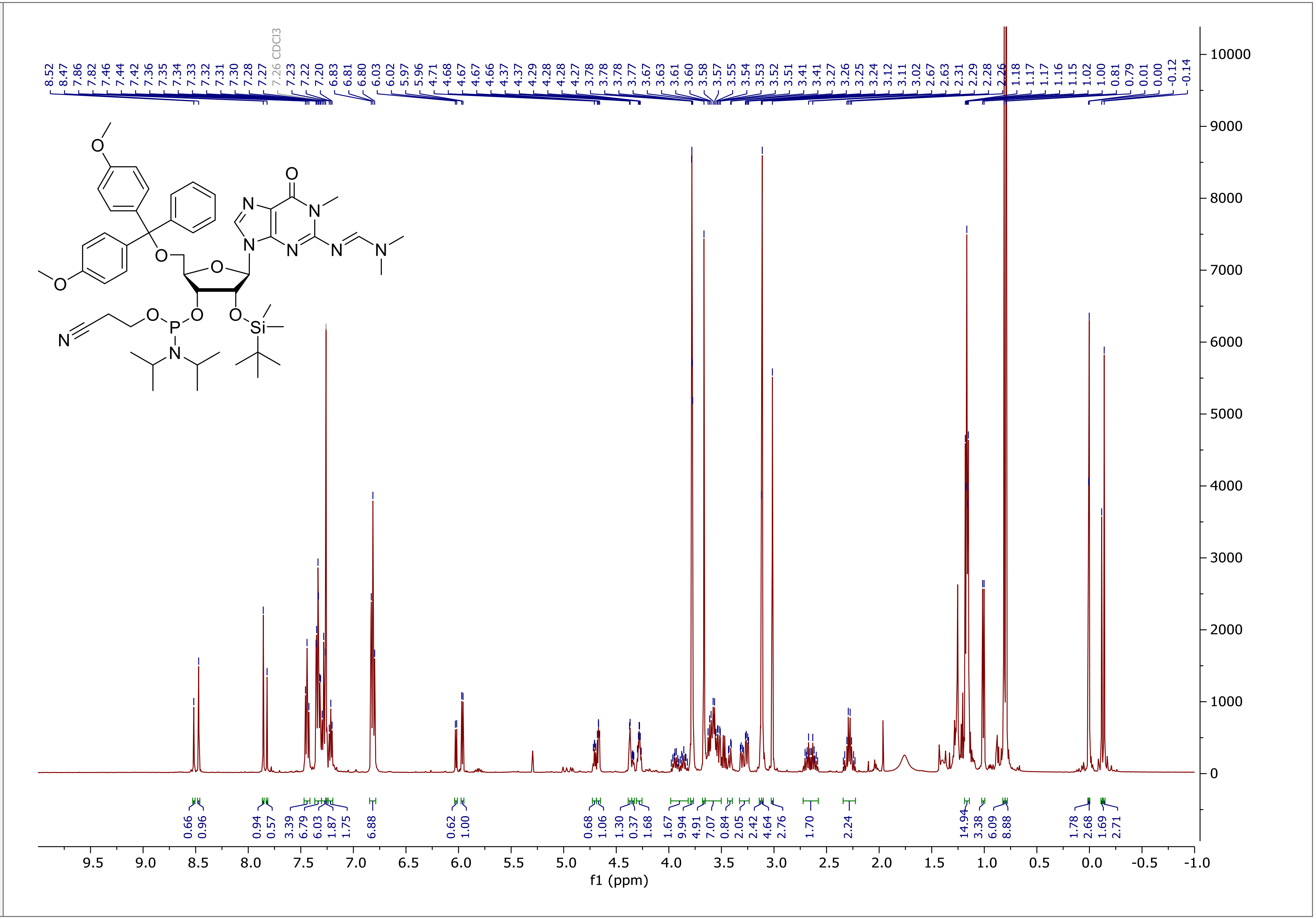

$^{13}C\{^1H\}$ NMR (126 MHz, $CDCl_3$, 25°C)

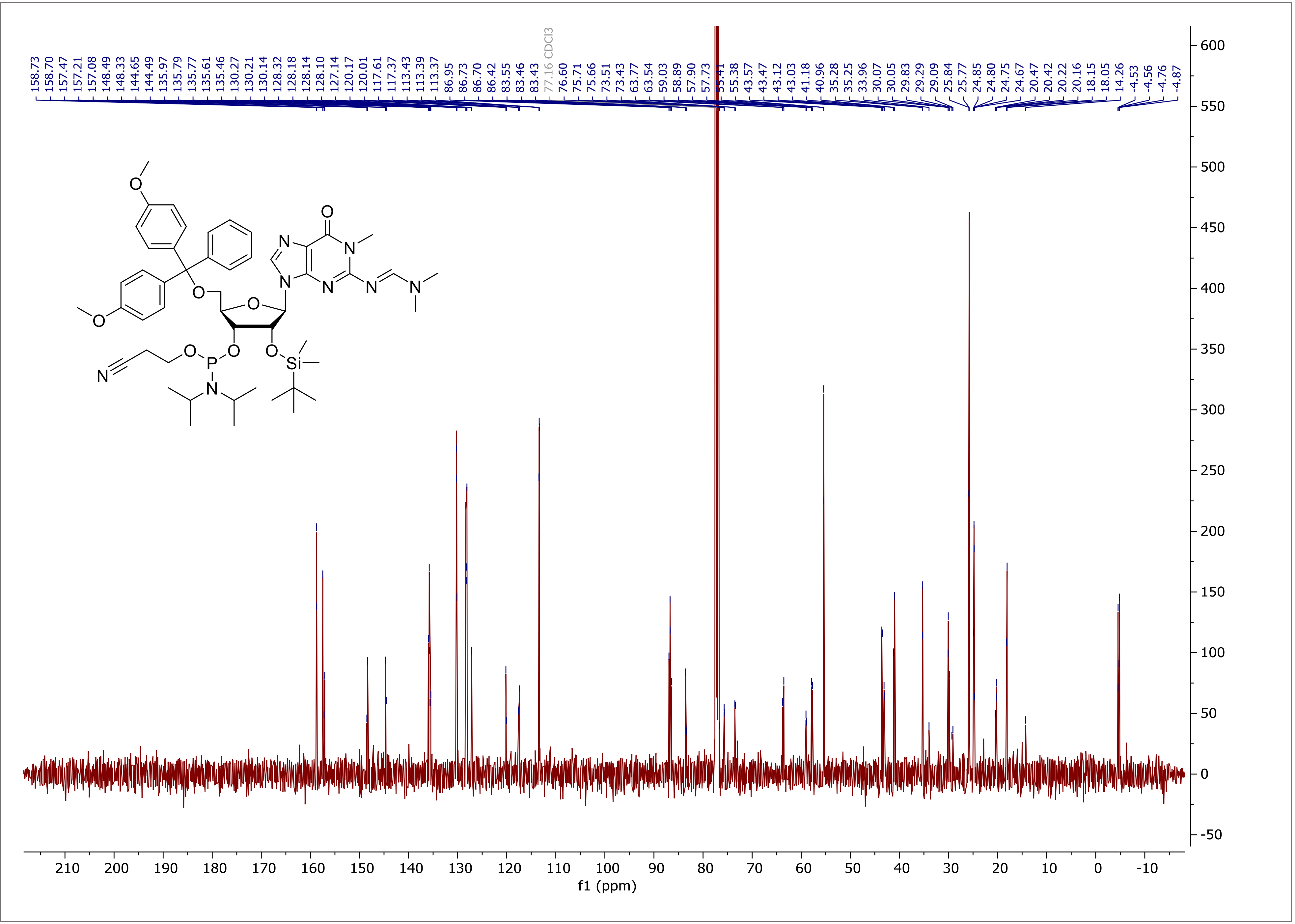

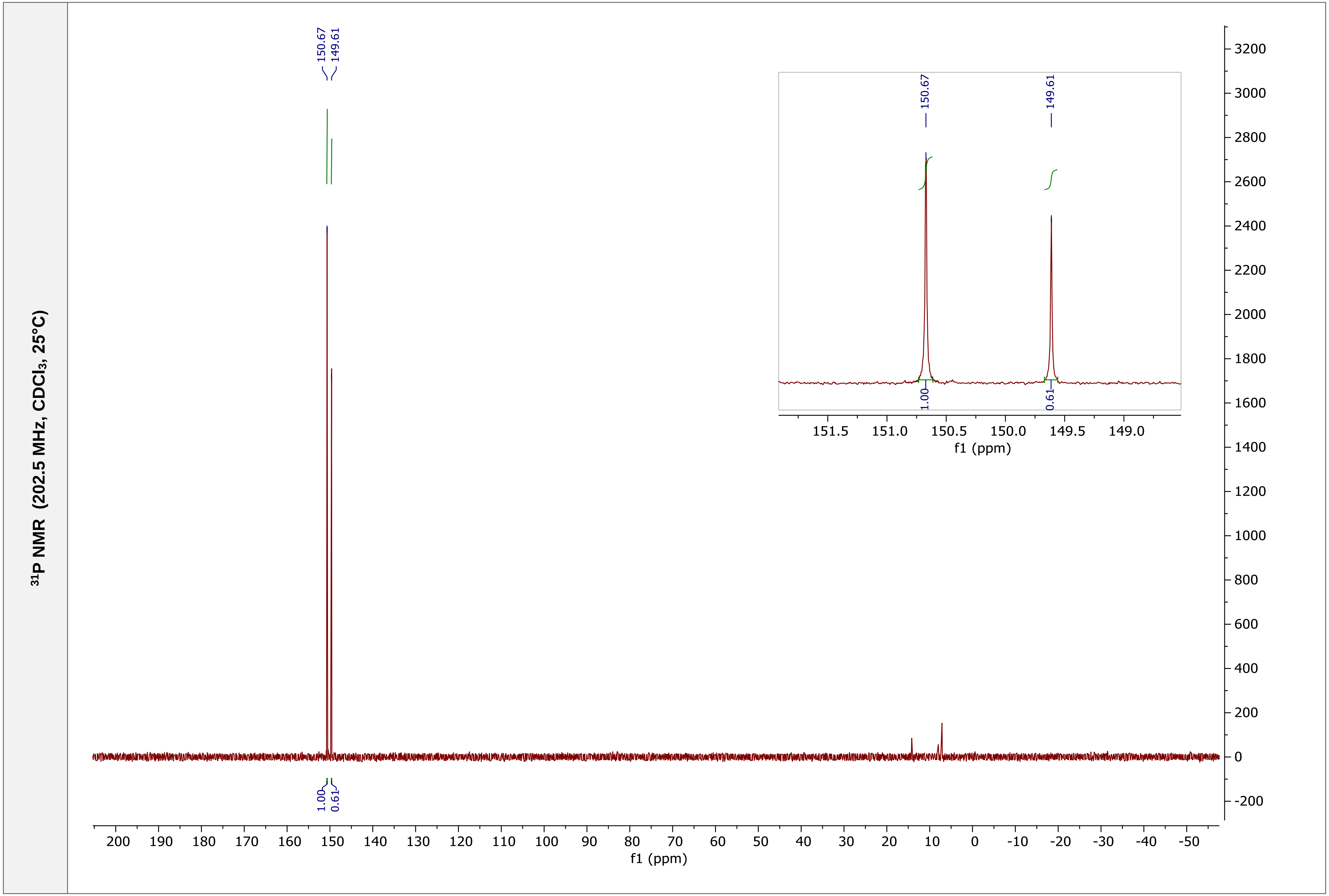

^{31}P NMR (202.5 MHz, CDCl$_3$, 25°C)
150.67
149.61
1.00
0.61
151.5
151.0
150.5
150.0
149.5
149.0
f1 (ppm)
200
190
180
170
160
150
140
130
120
110
100
90
80
70
60
50
40
30
20
10
0
-10
-20
-30
-40
-50
3200
3000
2800
2600
2400
2200
2000
1800
1600
1400
1200
1000
800
600
400
200
-200

COSY NMR (CDCl$_3$, 25°C)

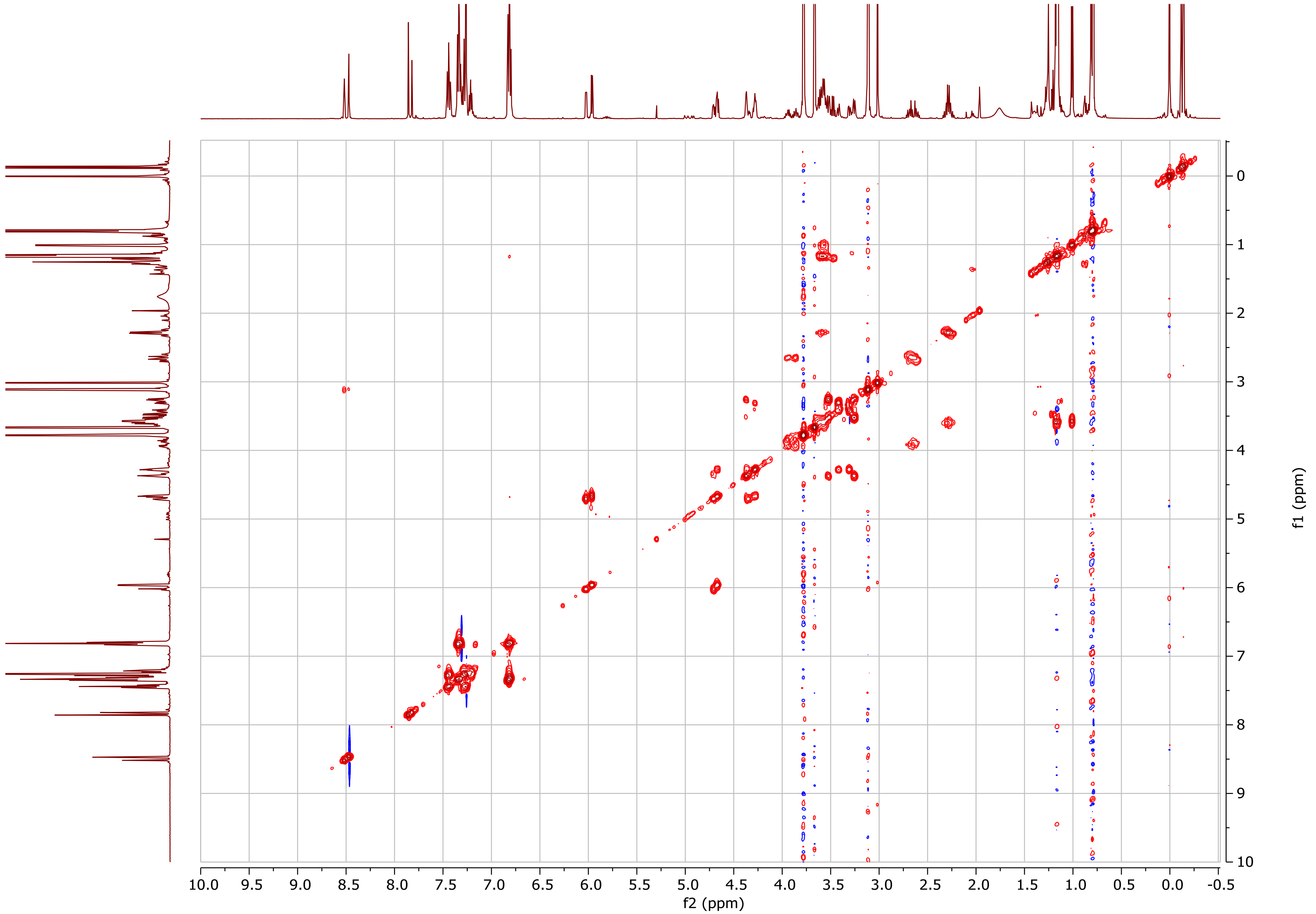

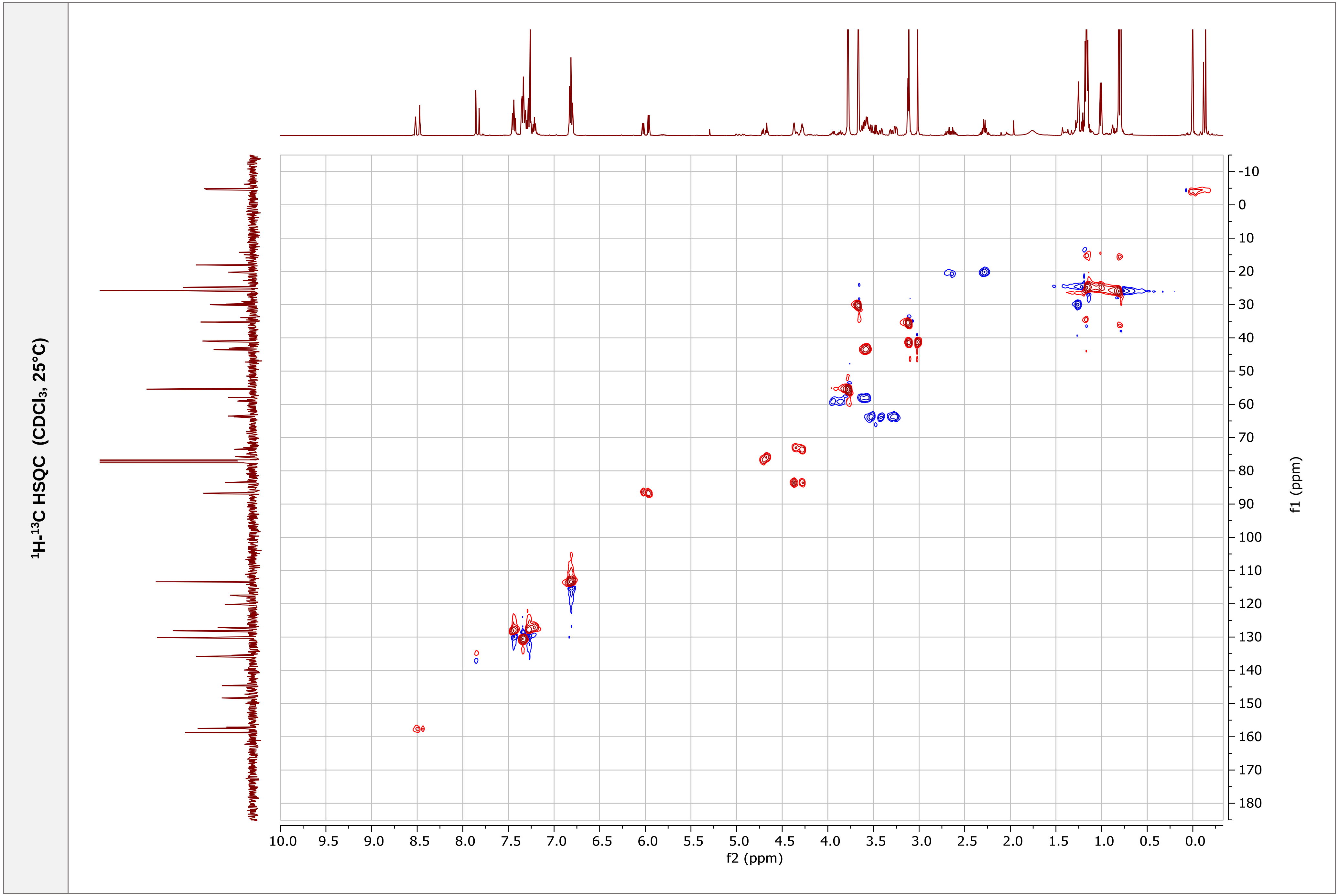

¹H-¹³C HSQC (CDCl₃, 25°C)
f2 (ppm)
f1 (ppm)

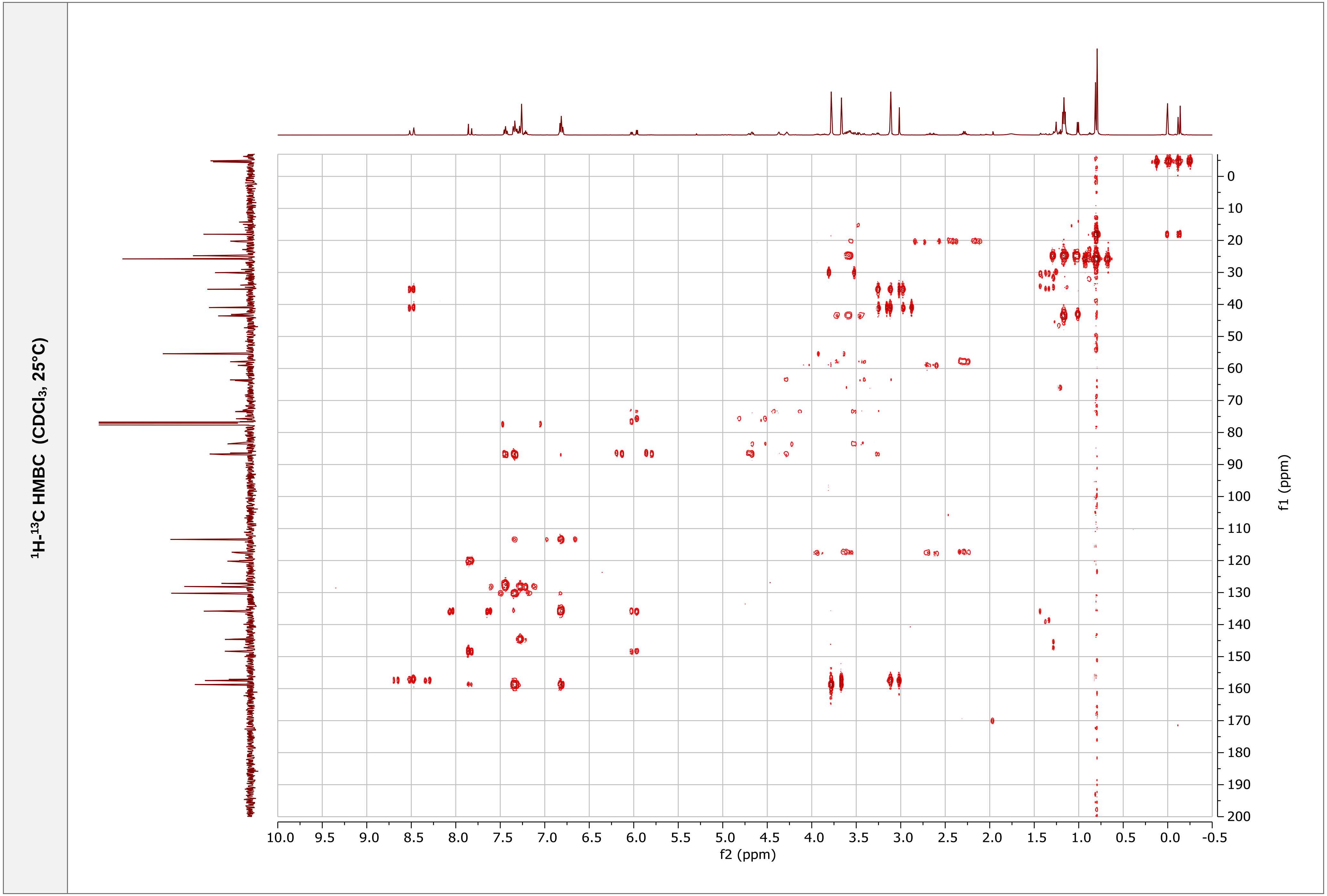

^{1}H-^{13}C HMBC (CDCl$_3$, 25°C)
f2 (ppm)
f1 (ppm)
10.0 9.5 9.0 8.5 8.0 7.5 7.0 6.5 6.0 5.5 5.0 4.5 4.0 3.5 3.0 2.5 2.0 1.5 1.0 0.5 0.0 -0.5
0 10 20 30 40 50 60 70 80 90 100 110 120 130 140 150 160 170 180 190 200

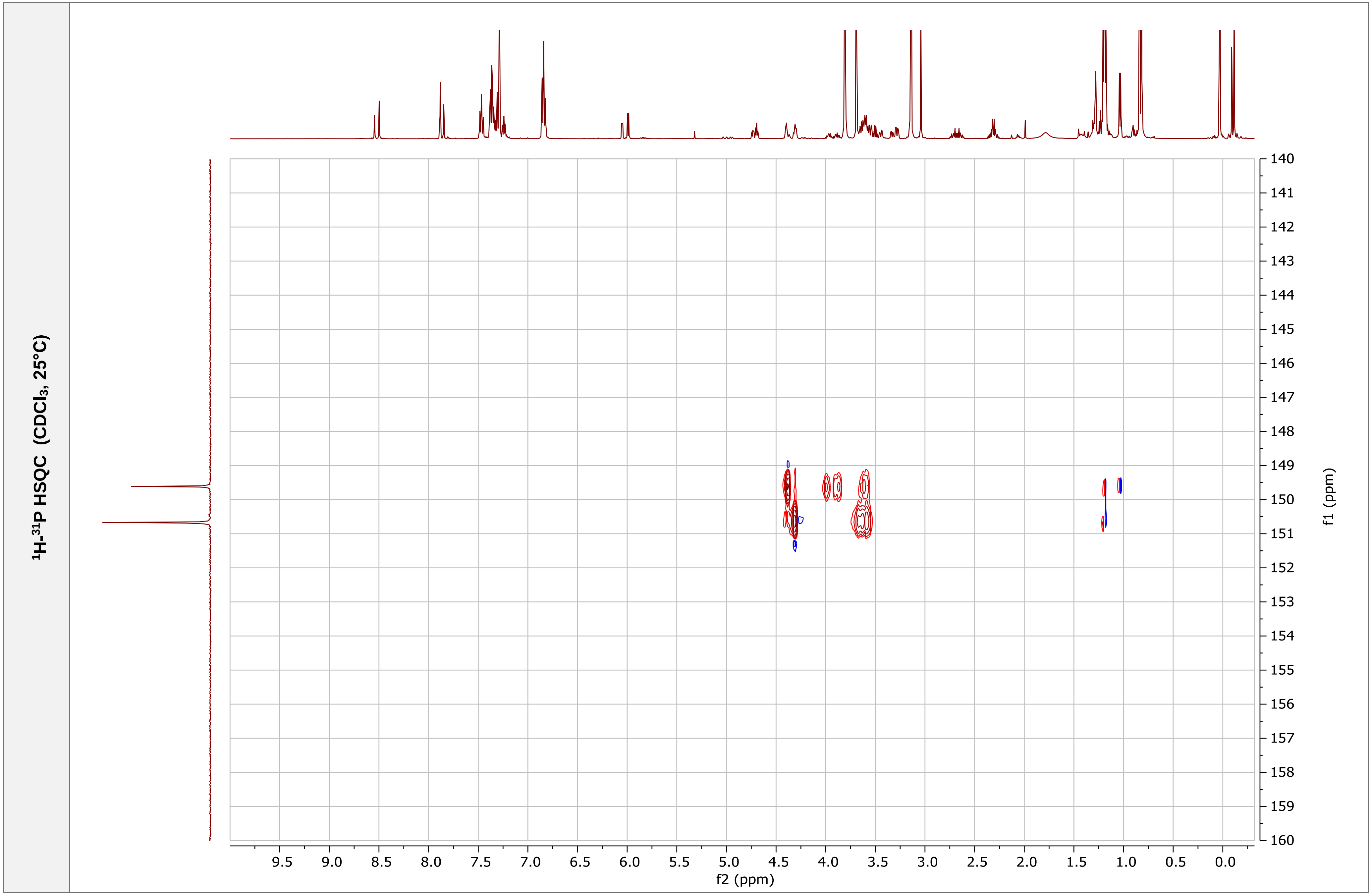
^{1}H-^{31}P HSQC (CDCl$_3$, 25°C)
f2 (ppm)
f1 (ppm)

(11) Ui^{6}AU

Chemical structure

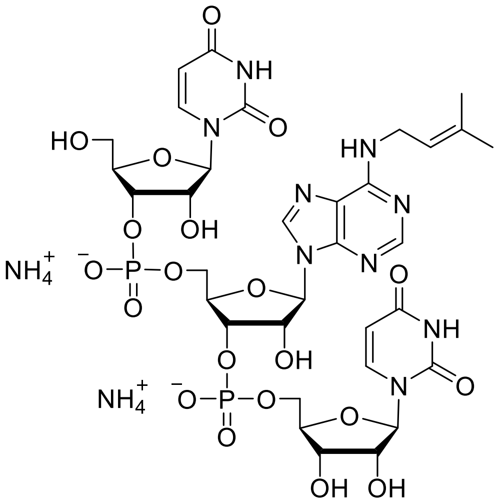

RP HPLC
Abs. @ 254 nm

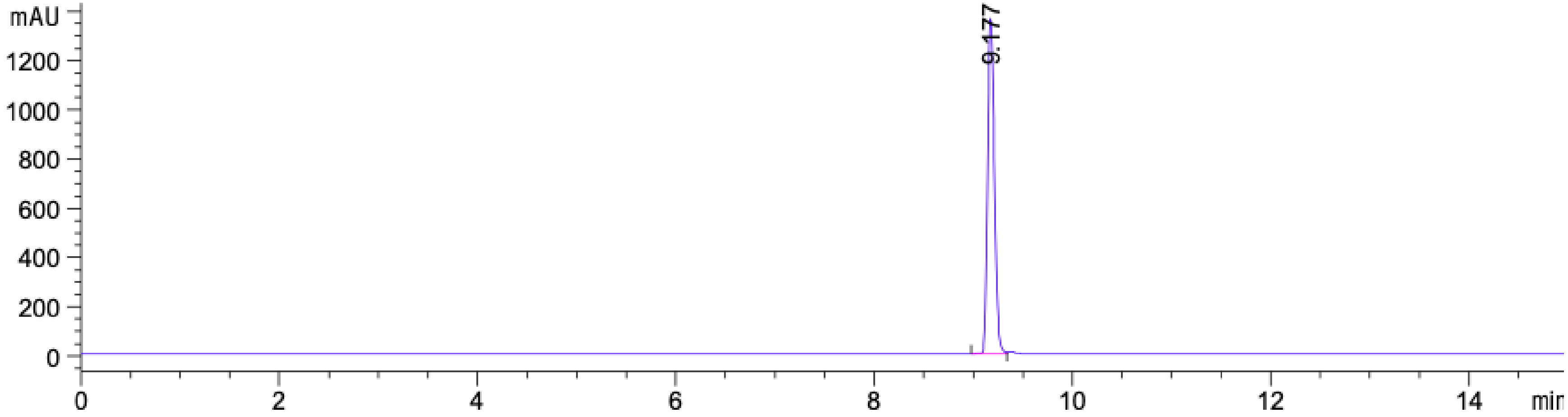

MS (-) ESI

(Calc. [M-H]$^-$ $C_{33}H_{42}N_9O_{20}P_2^-$ 946.20268)

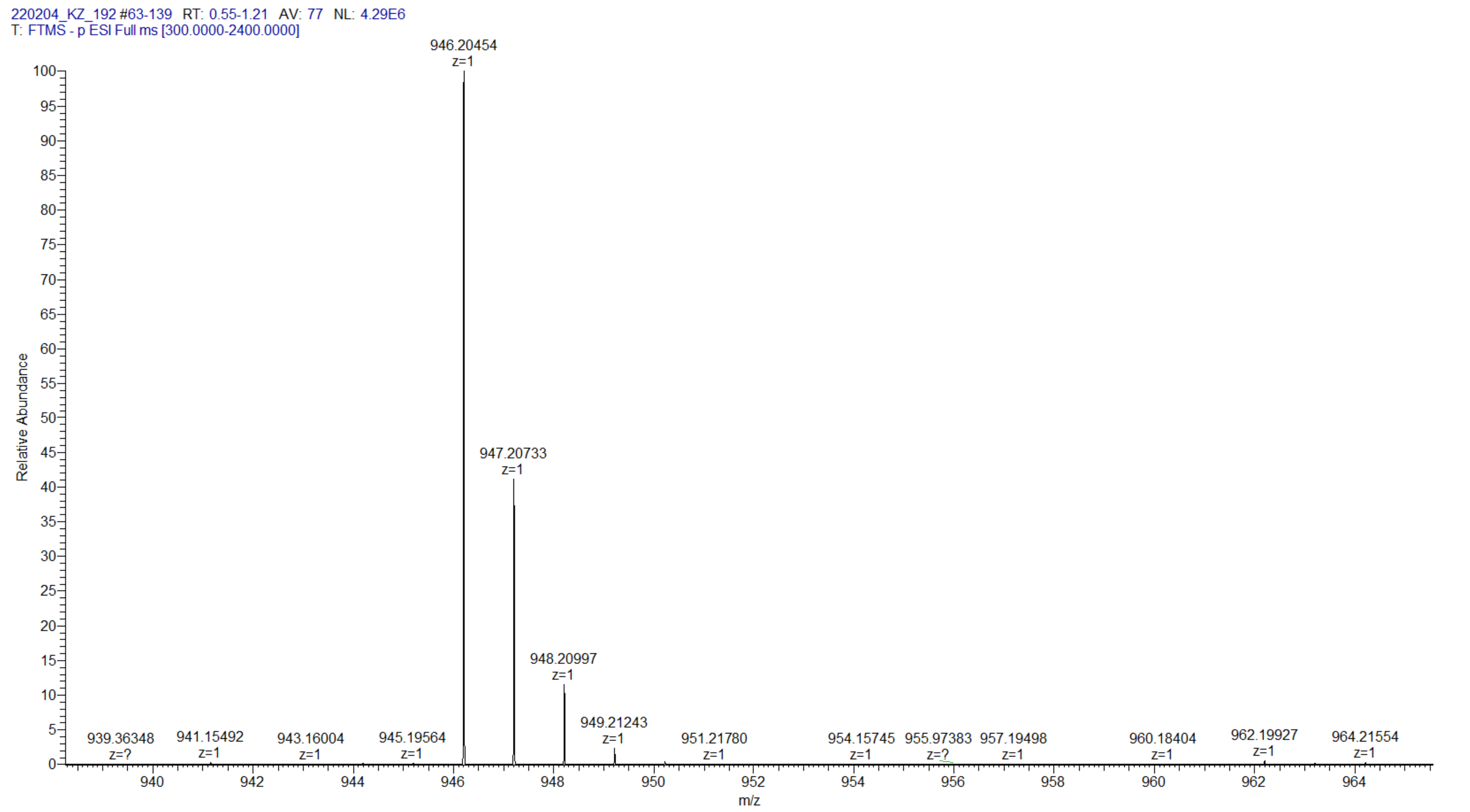

^{1}H NMR (500 MHz, D_2O, 25°C)

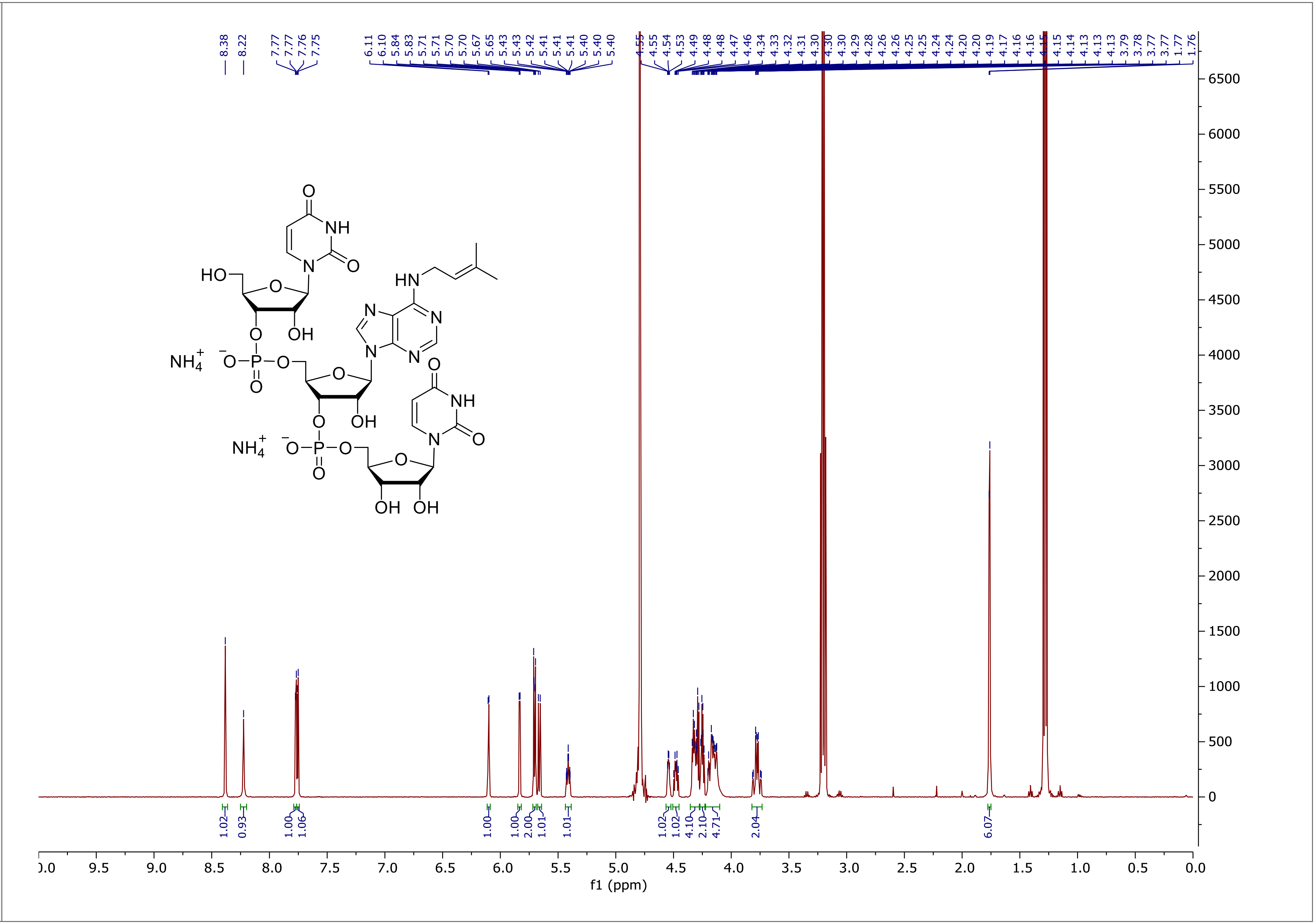

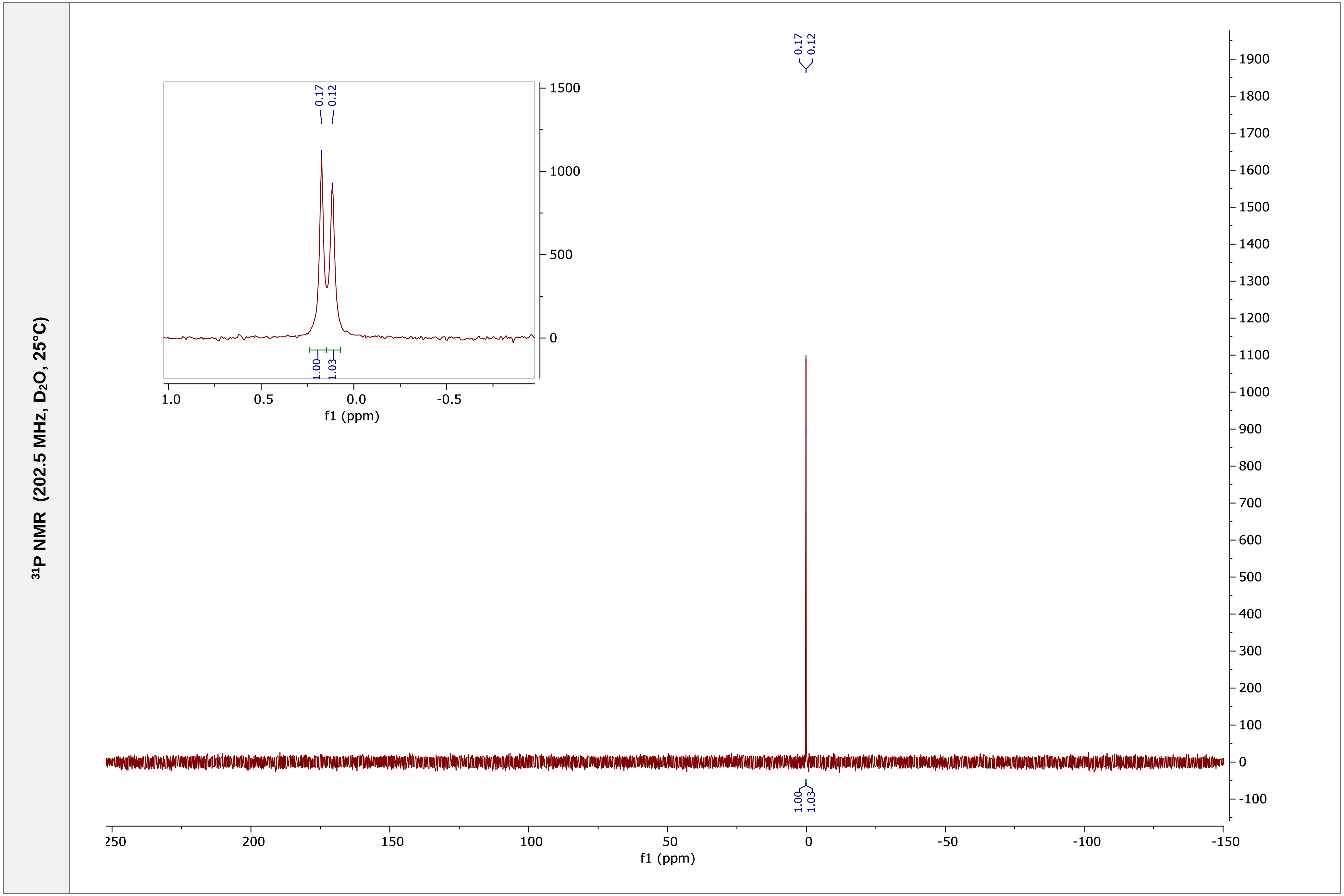

^{31}P NMR (202.5 MHz, D_2O, 25°C)
0.17
0.12
1.00
1.03
f1 (ppm)

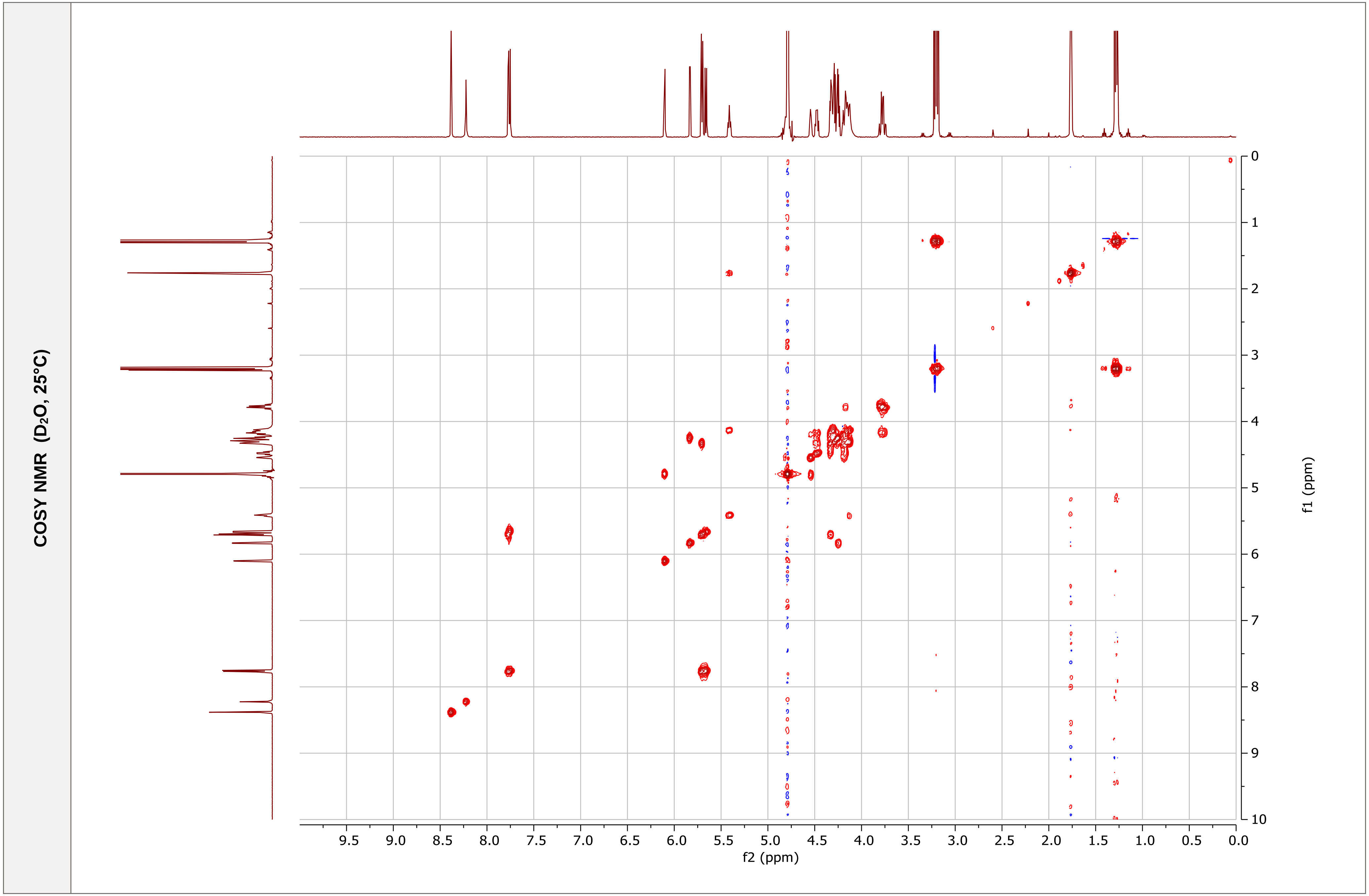

COSY NMR (D$_2$O, 25°C)
f2 (ppm)
f1 (ppm)
9.5 9.0 8.5 8.0 7.5 7.0 6.5 6.0 5.5 5.0 4.5 4.0 3.5 3.0 2.5 2.0 1.5 1.0 0.5 0.0
0 1 2 3 4 5 6 7 8 9 10

^{1}H-^{13}C HSQC (D_2O, 25°C)

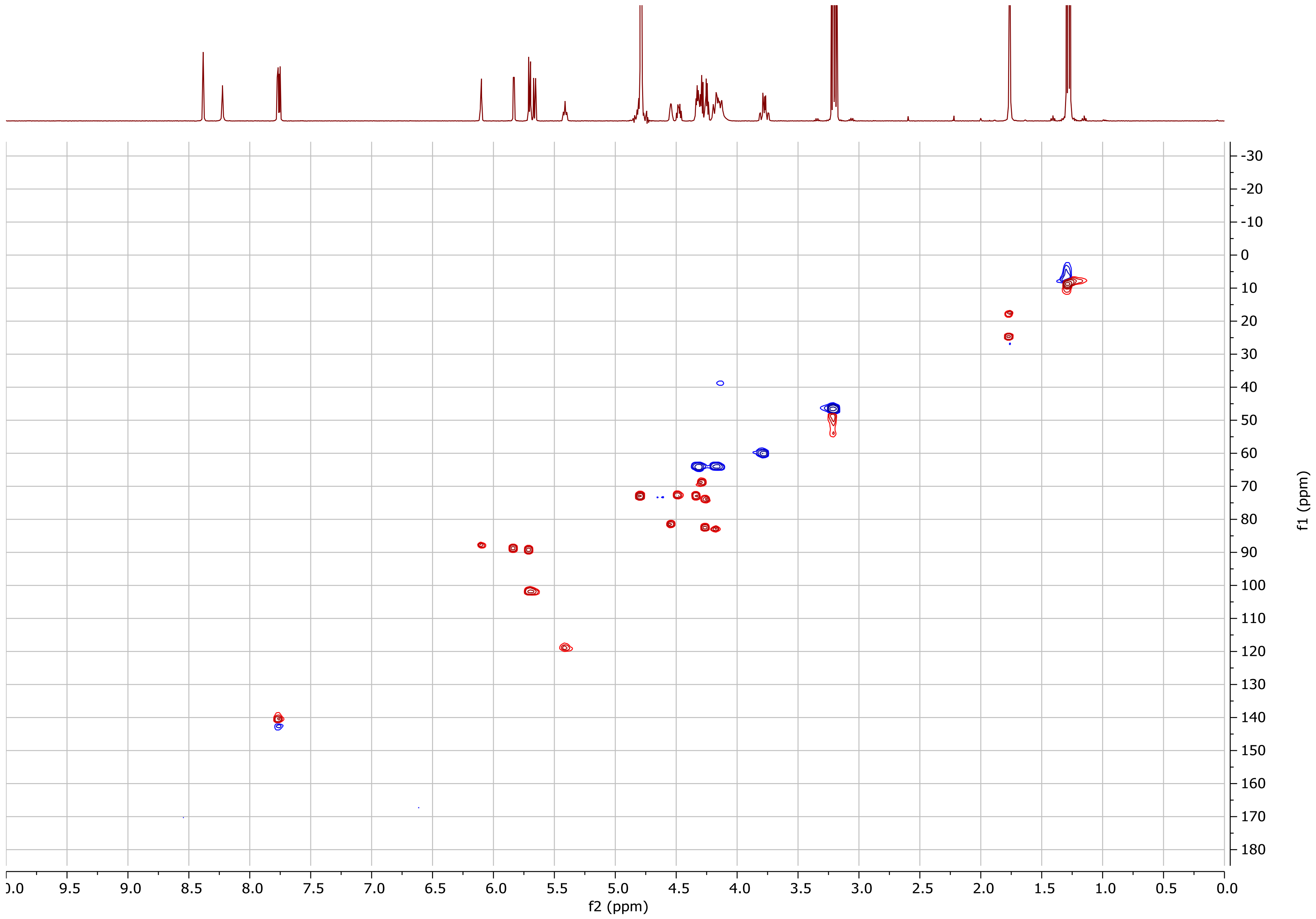

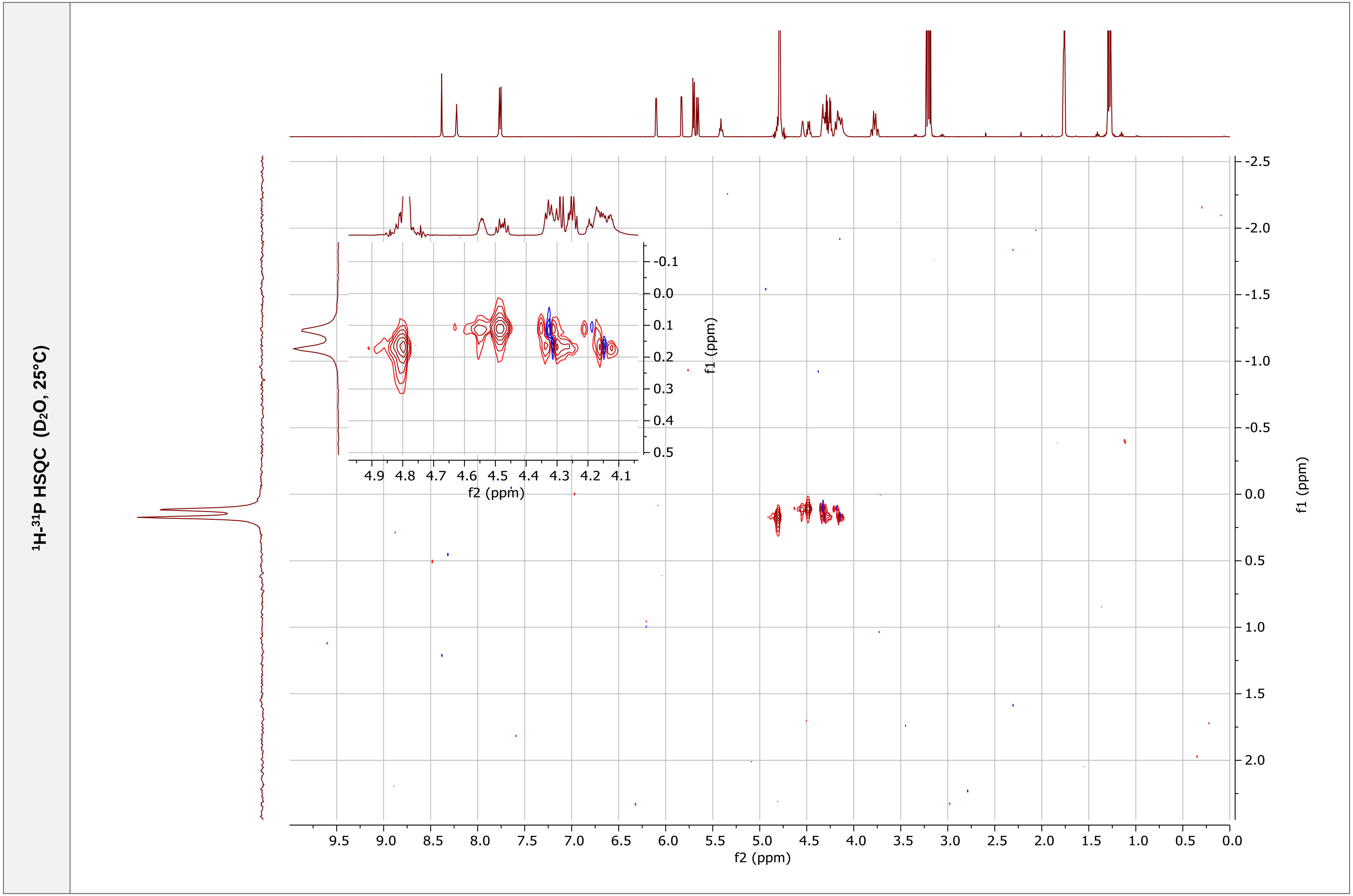
^1H-^{31}P HSQC (D$_2$O, 25°C)
f2 (ppm)
f1 (ppm)

(12) $p^{Bn6}A_mpG$

Chemical structure

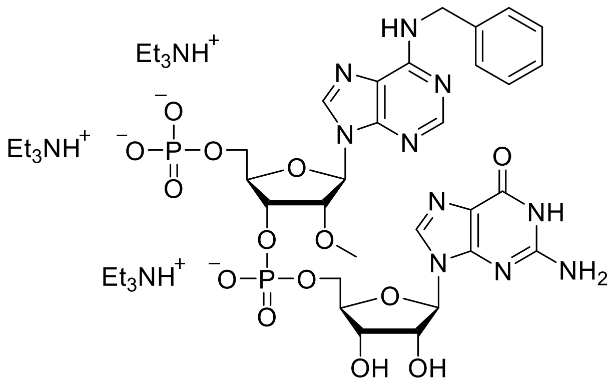

RP HPLC
Abs. @ 254 nm

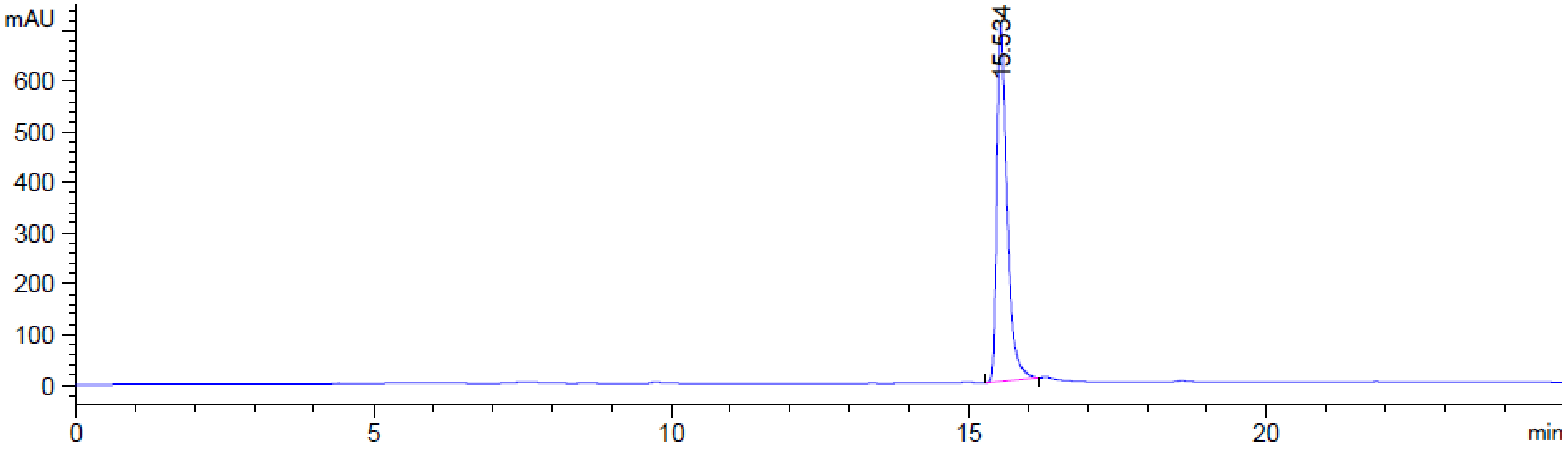

MS (-) ESI

Calc. [M-H]$^-$ $C_{28}H_{33}N_{10}O_{14}P_2^-$ 795.16584

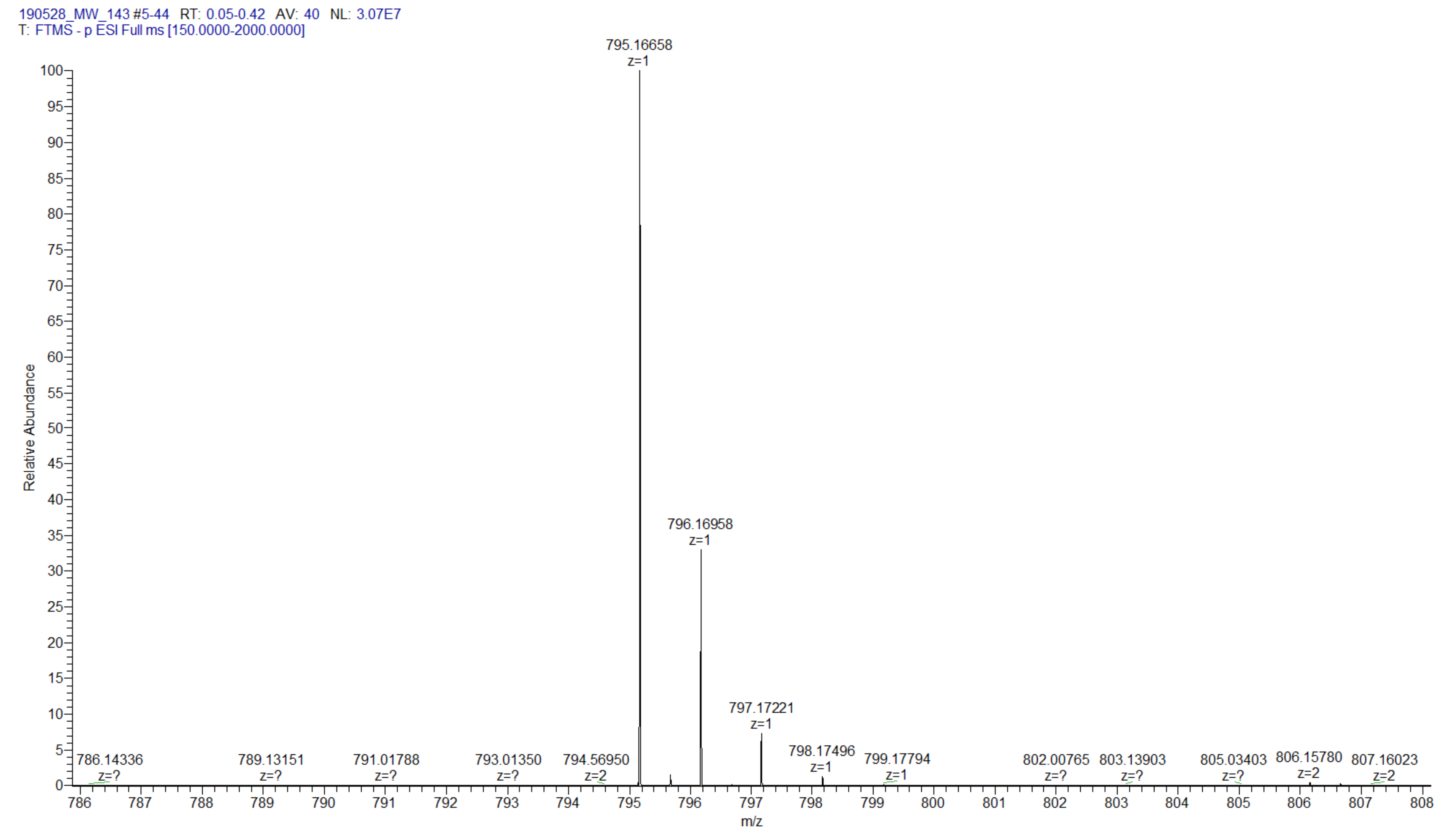

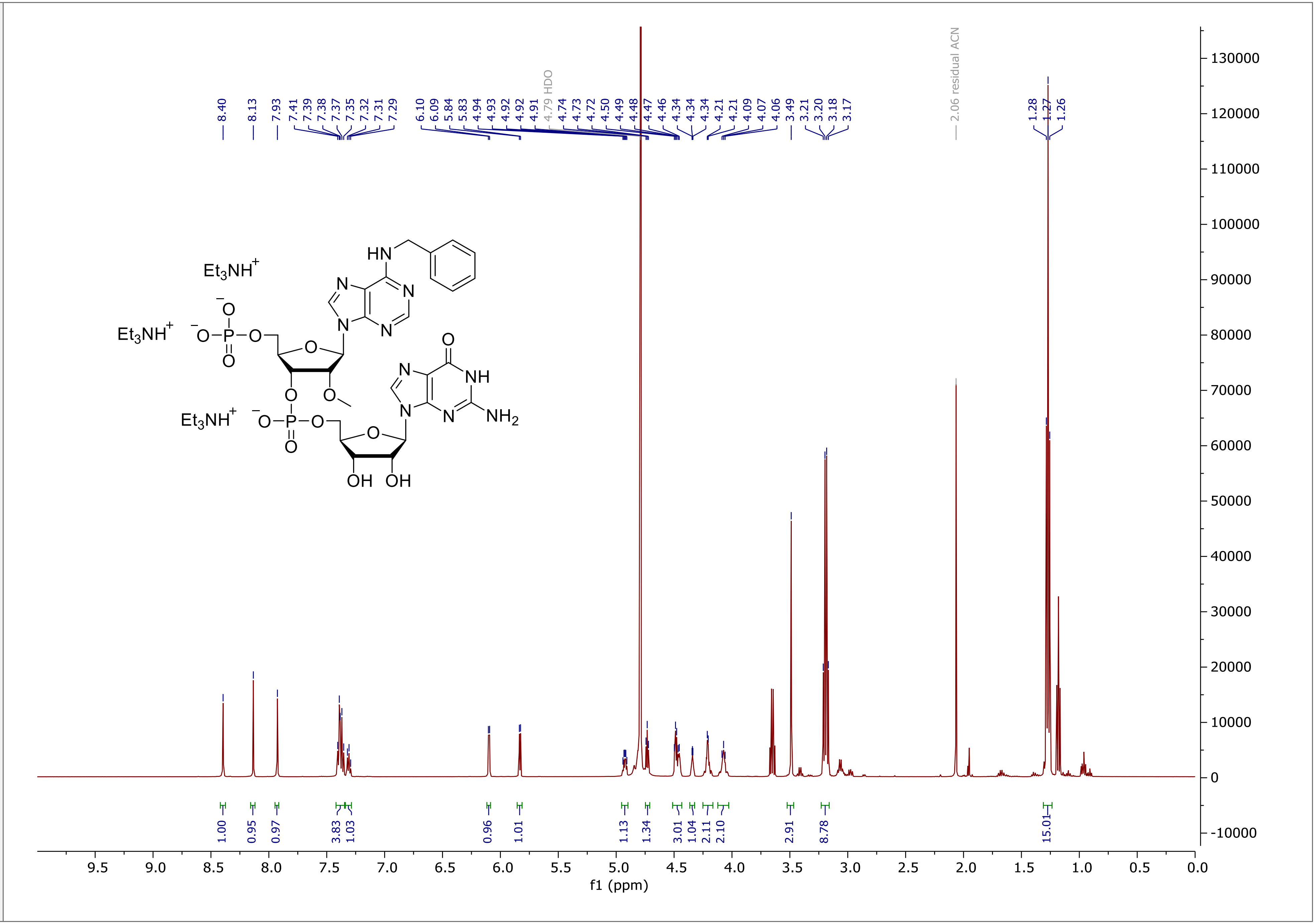

^{1}H NMR (500 MHz, D_2O, 25°C)

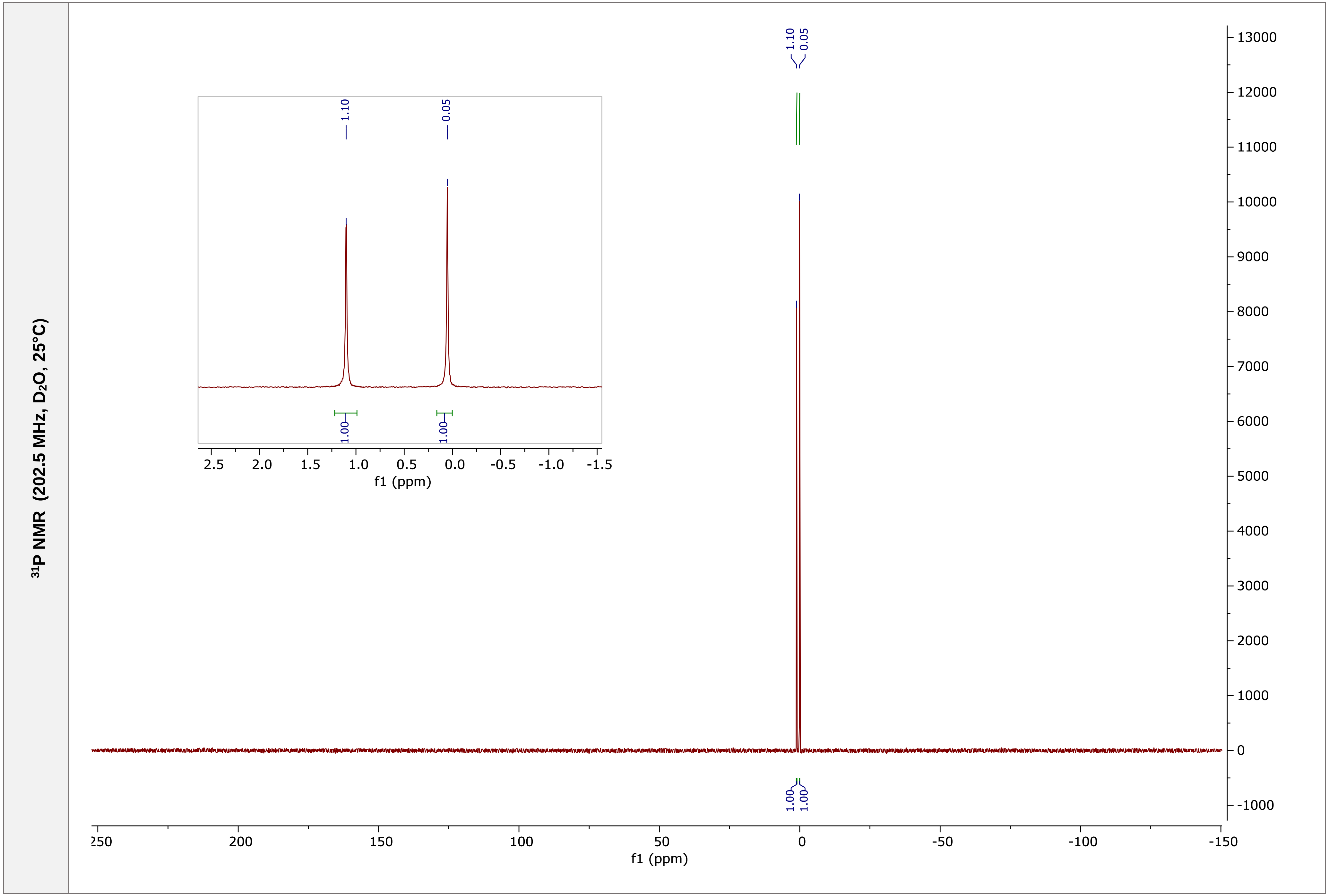

^{31}P NMR (202.5 MHz, D$_2$O, 25°C)
1.10
0.05
1.00
1.00
2.5
2.0
1.5
1.0
0.5
0.0
-0.5
-1.0
-1.5
f1 (ppm)
13000
12000
11000
10000
9000
8000
7000
6000
5000
4000
3000
2000
1000
0
-1000
250
200
150
100
50
0
-50
-100
-150
f1 (ppm)

COSY NMR (D$_2$O, 25°C)

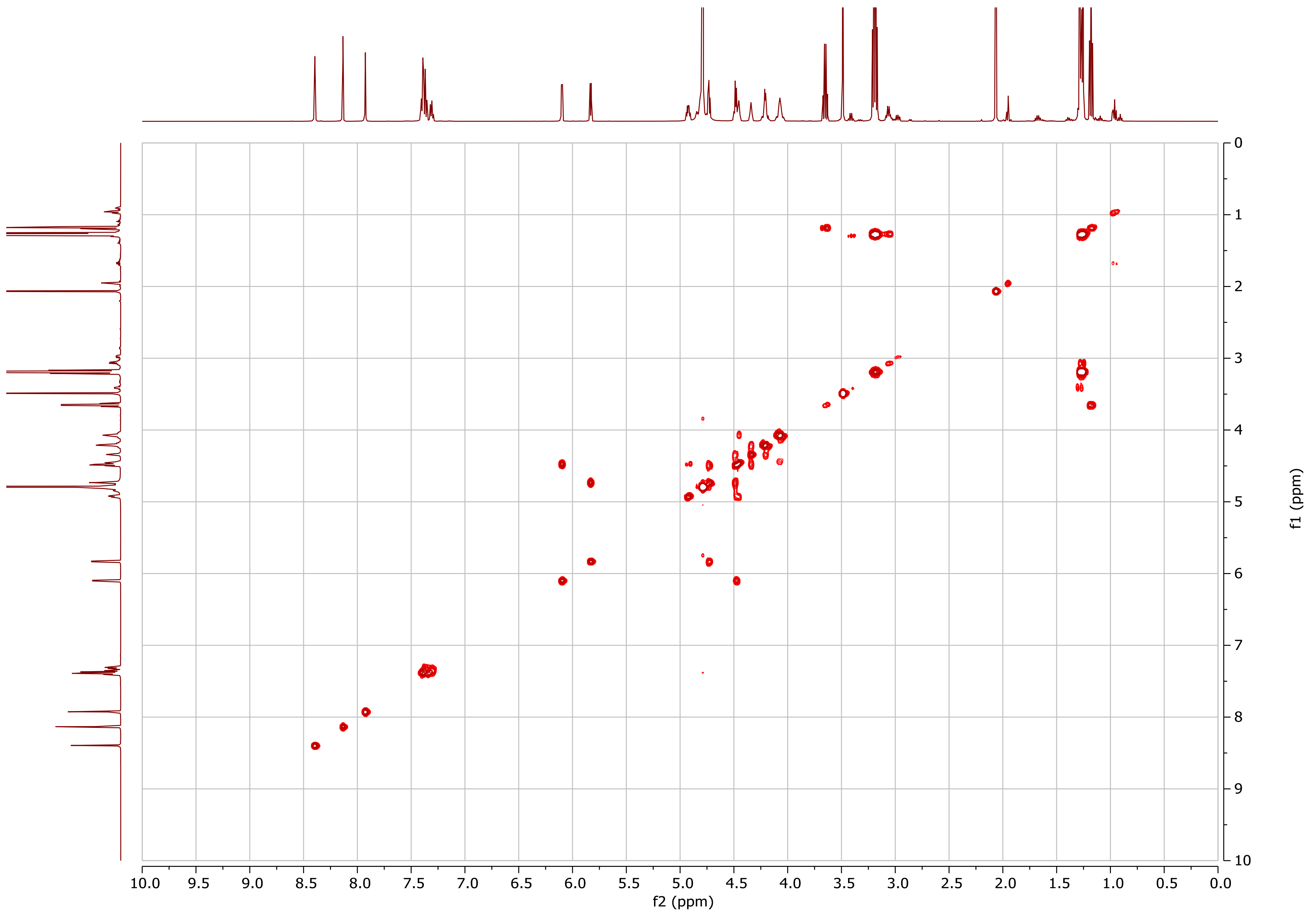

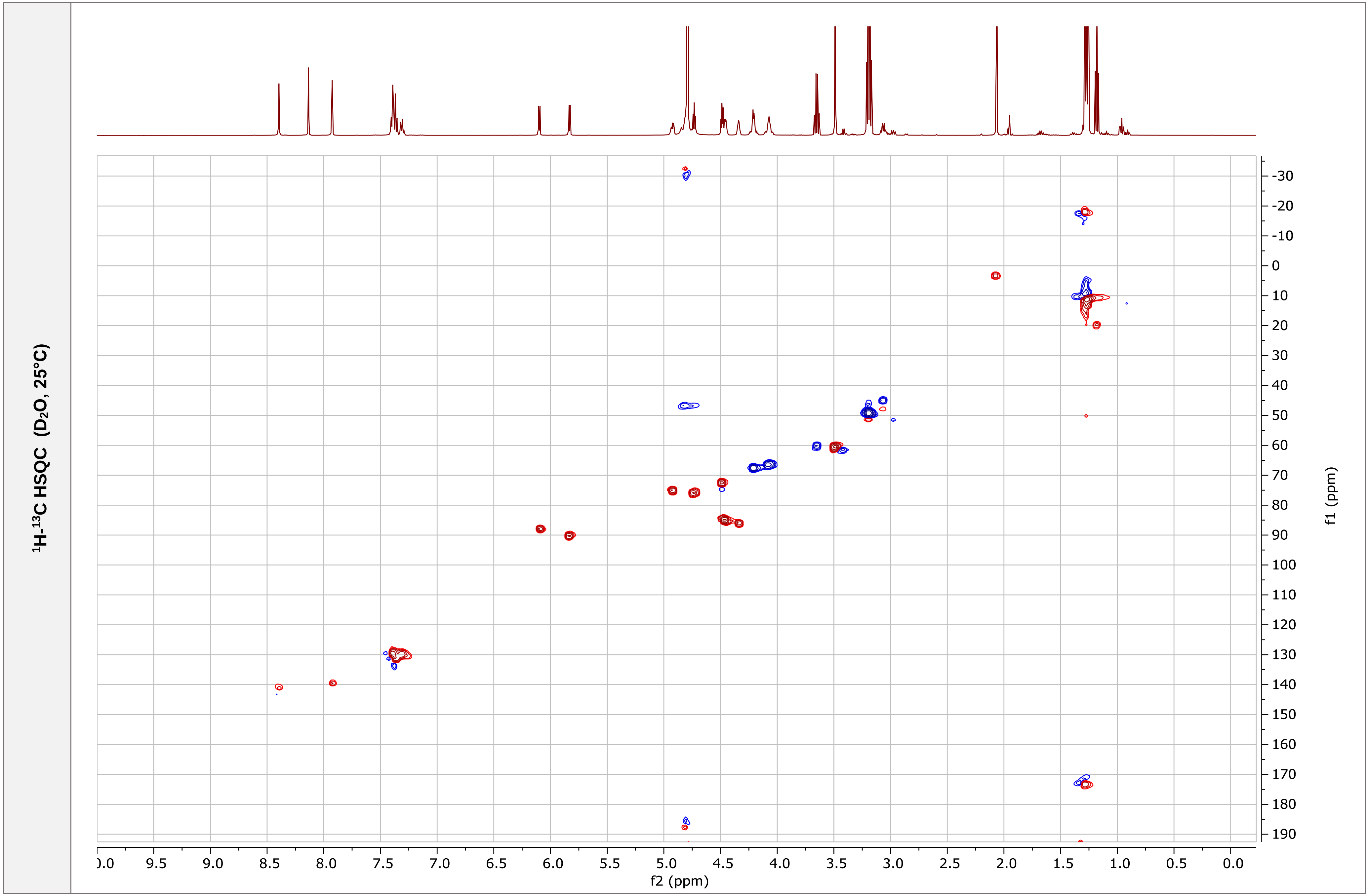

^{1}H-^{13}C HSQC (D$_2$O, 25°C)
f2 (ppm)
f1 (ppm)

(13) $p^{hex6}A_mpG$	
**Chemical structure**	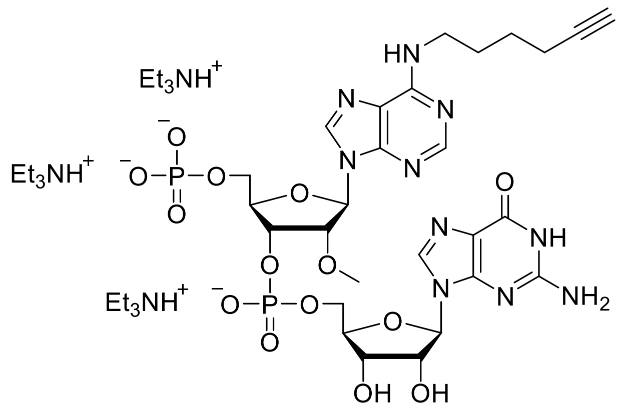
**RP HPLC** Abs. @ 254 nm	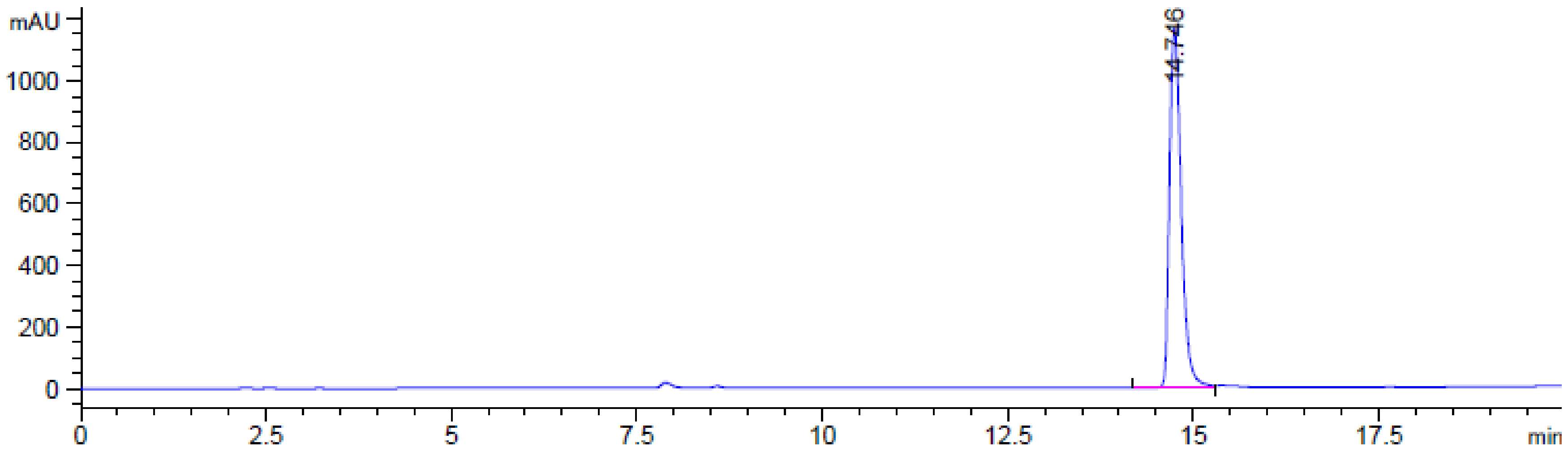

MS (-) ESI
(Calc. [M-H]$^-$ $C_{27}H_{35}N_{10}O_{14}P_2^-$ 785.18149)

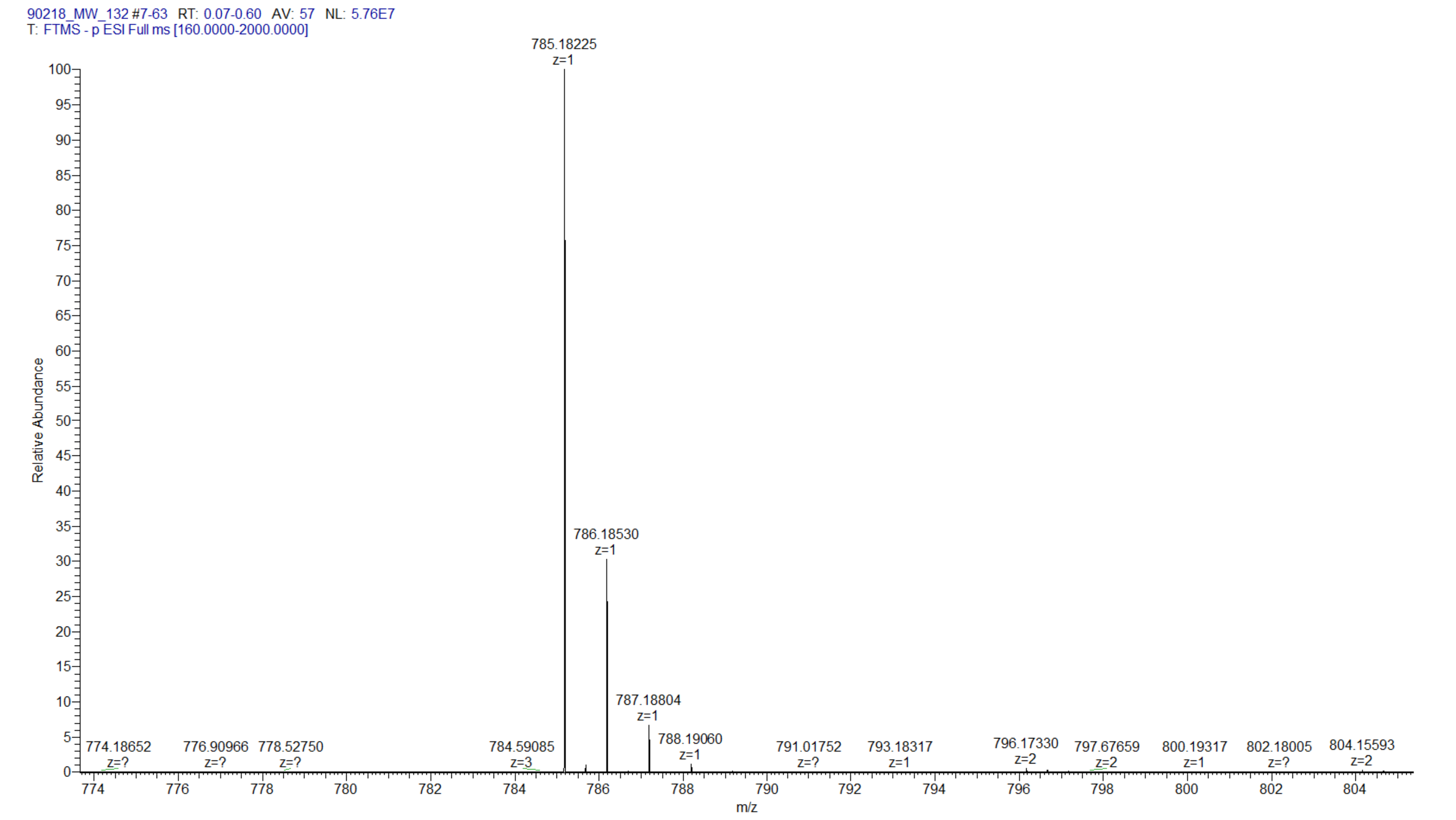

^{1}H NMR (500 MHz, D_2O, 25°C)

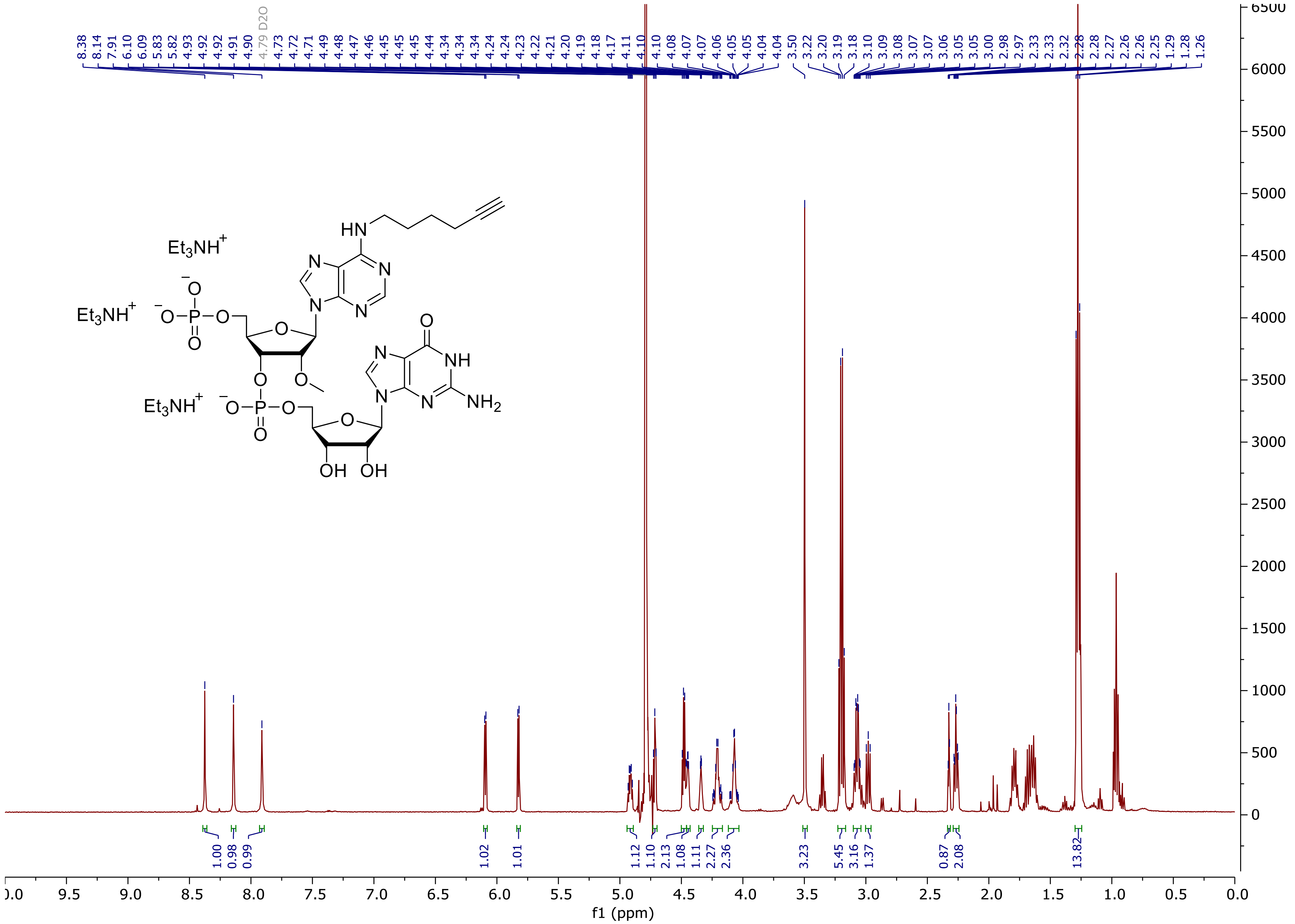

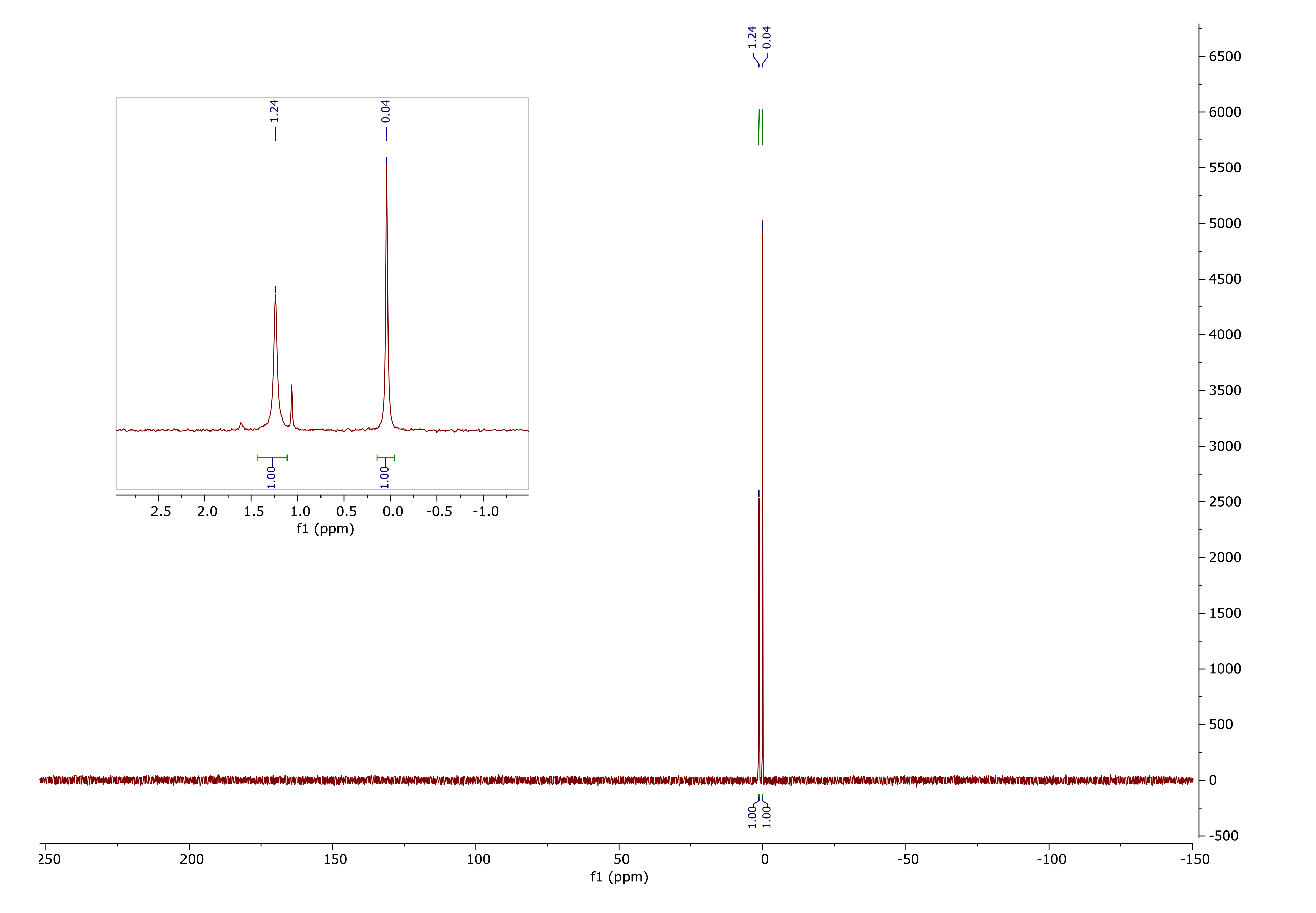

^{31}P NMR (202.5 MHz, D_2O, 25°C)

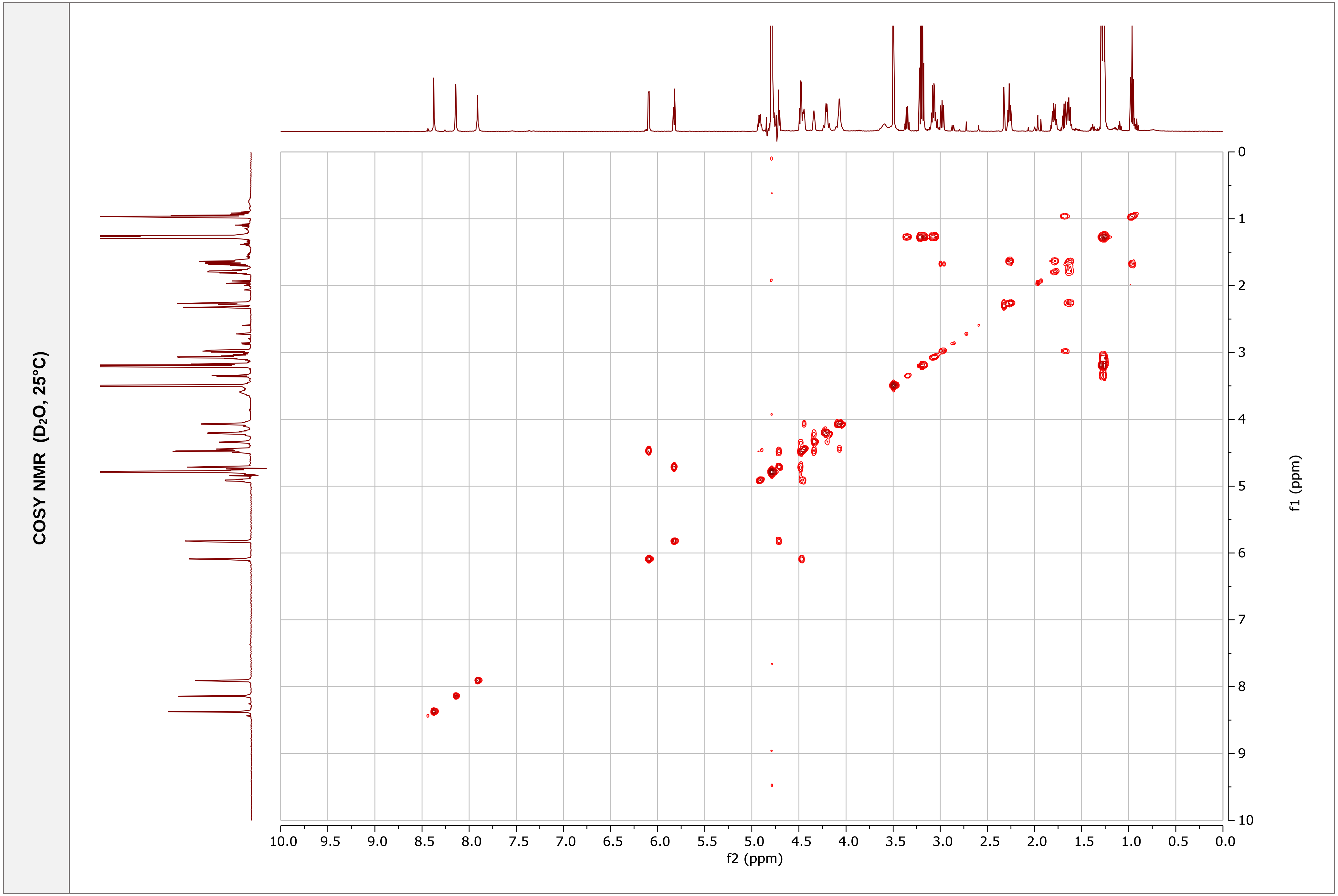
COSY NMR (D2O, 25°C)
f2 (ppm)
f1 (ppm)
10.0
9.5
9.0
8.5
8.0
7.5
7.0
6.5
6.0
5.5
5.0
4.5
4.0
3.5
3.0
2.5
2.0
1.5
1.0
0.5
0.0
0
1
2
3
4
5
6
7
8
9
10

^{1}H-^{13}C HSQC (D$_2$O, 25°C)

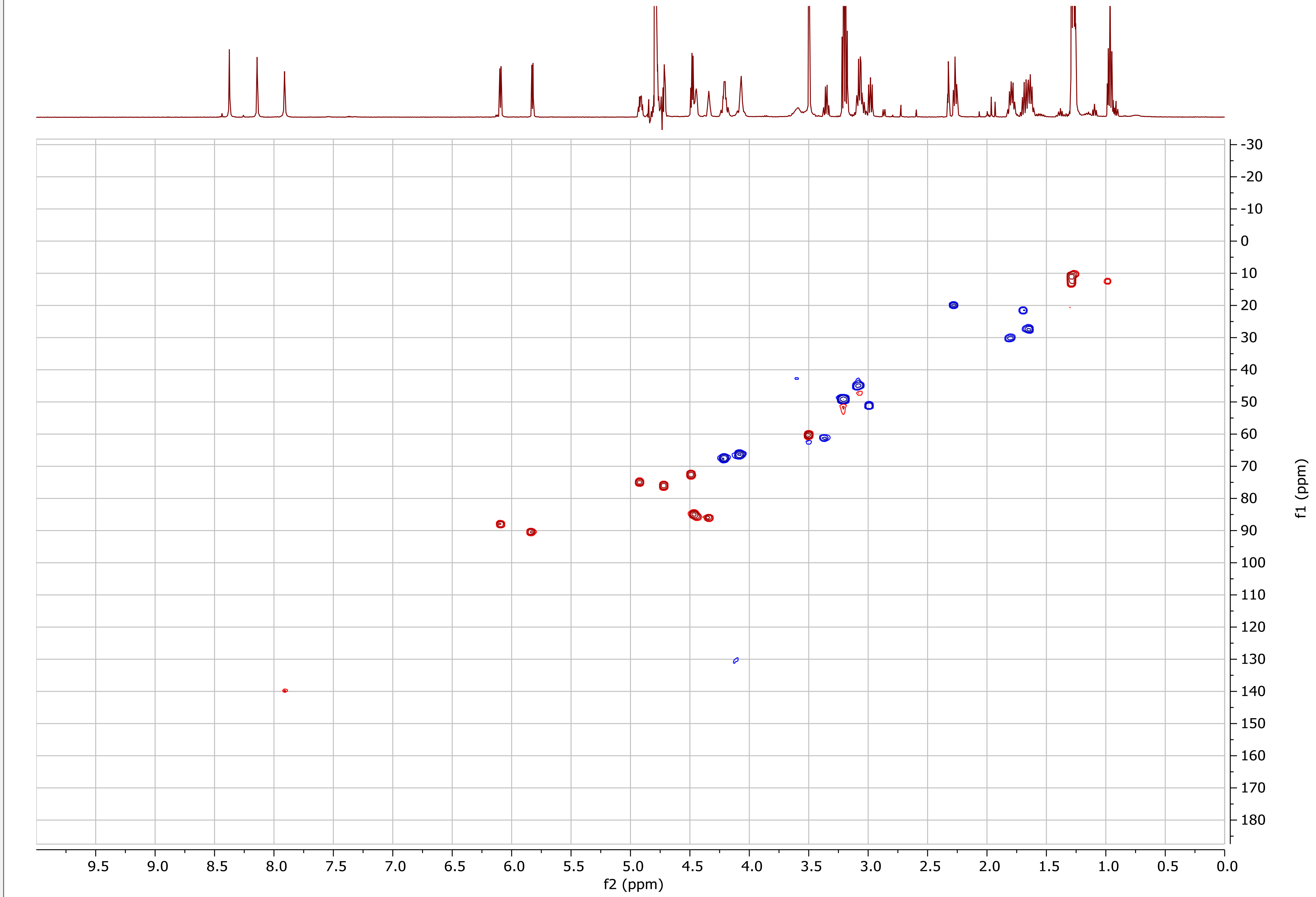

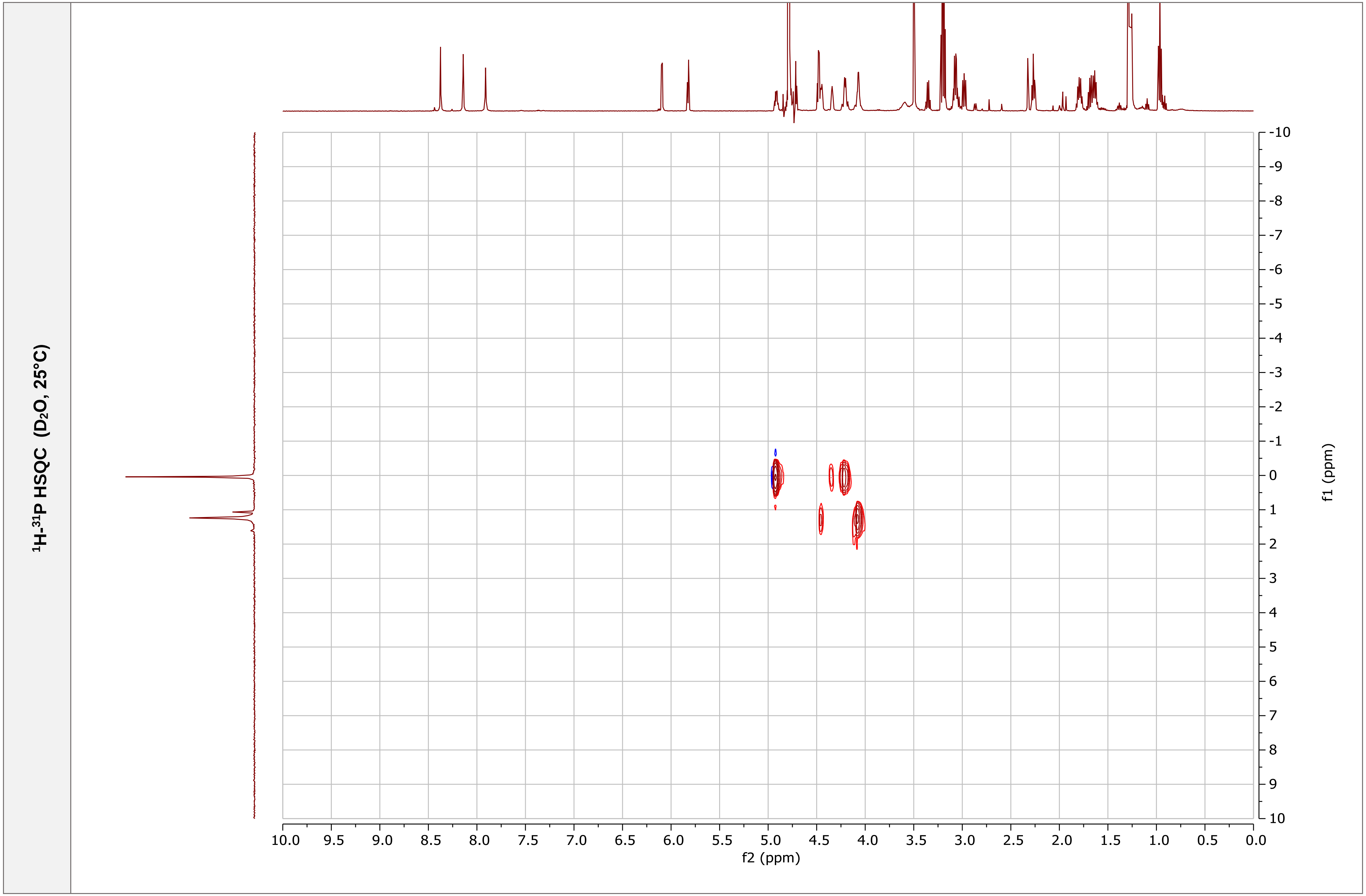

^1H-^{31}P HSQC (D$_2$O, 25°C)
f2 (ppm)
f1 (ppm)

(14) $p^{ap6}A_mpApG$

Chemical structure

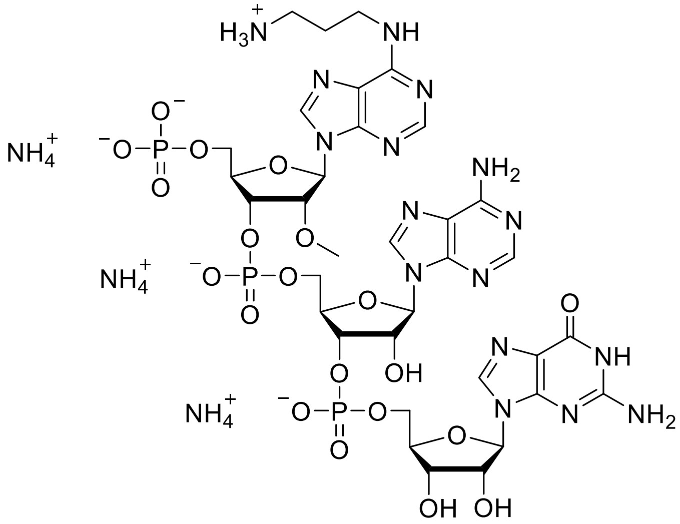

RP HPLC
Abs. @ 254 nm

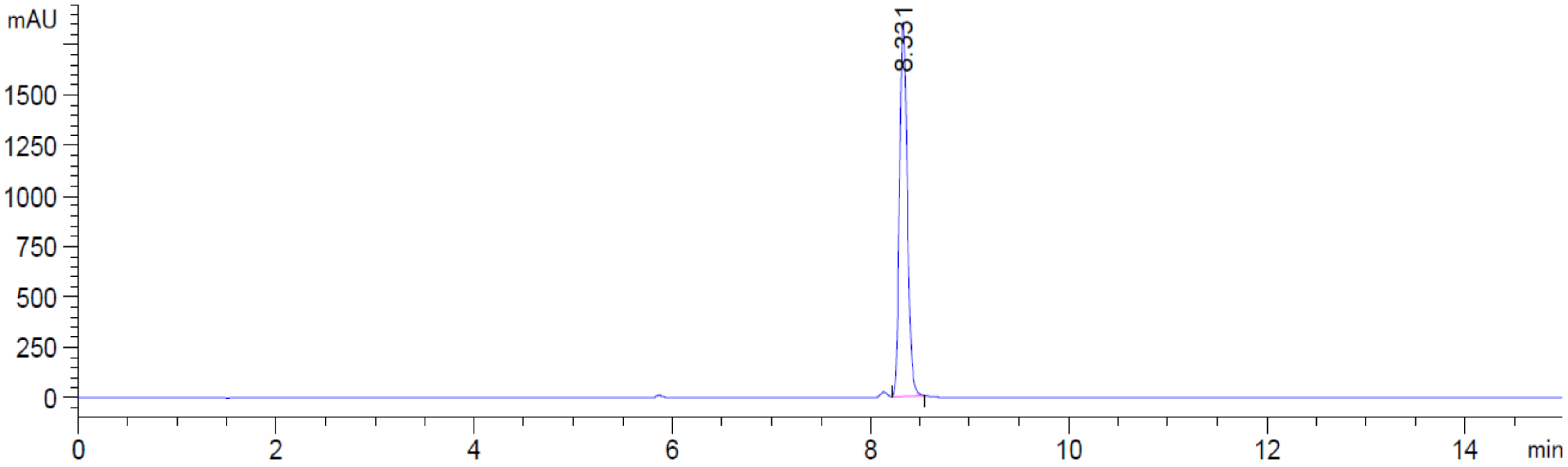

MS (-) ESI

(Calc. $[M\text{-}H]^- C_{34}H_{46}N_{16}O_{20}P_3^-$ 1091.22926)

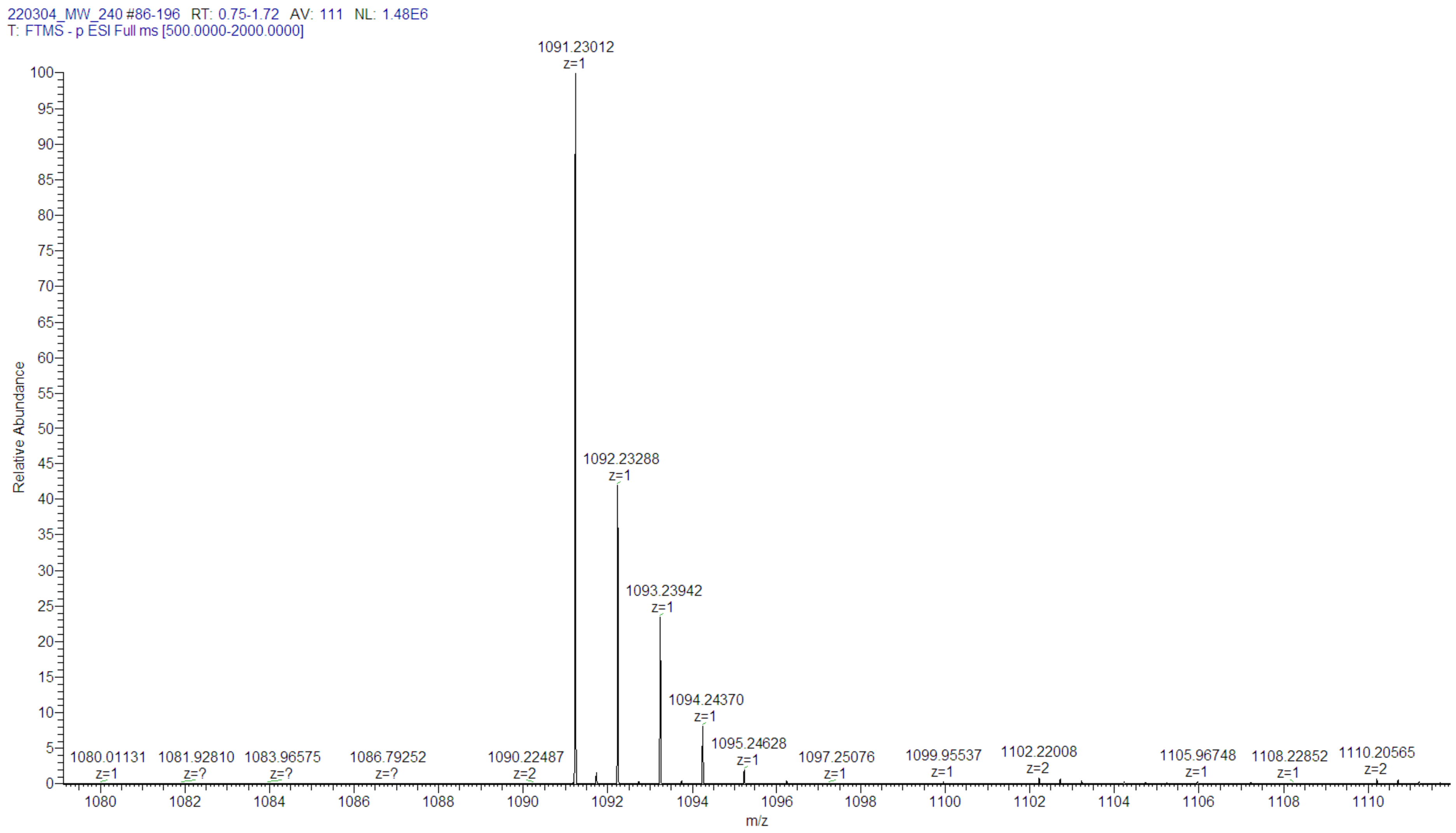

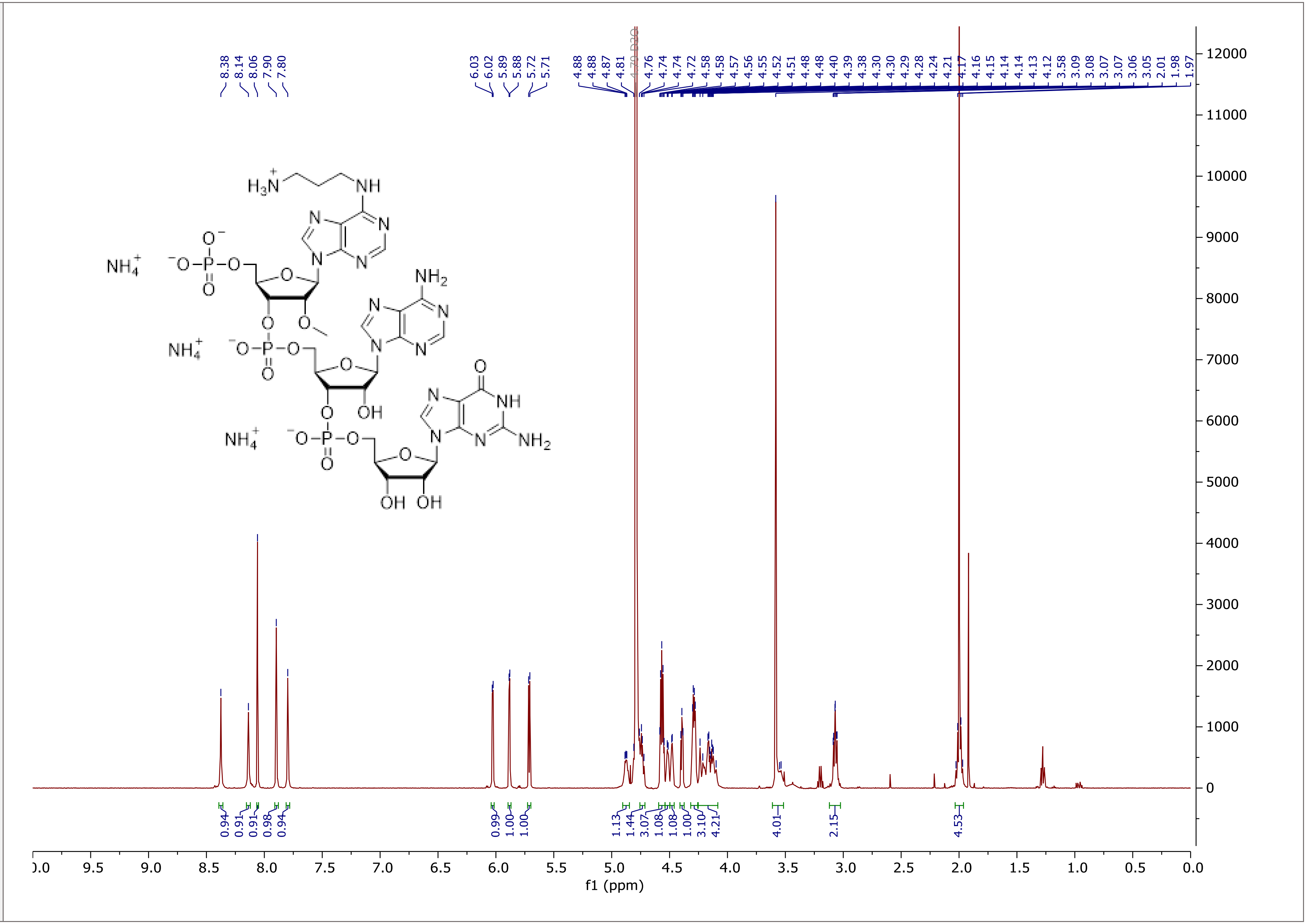

^{1}H NMR (500 MHz, D_2O, 25°C)

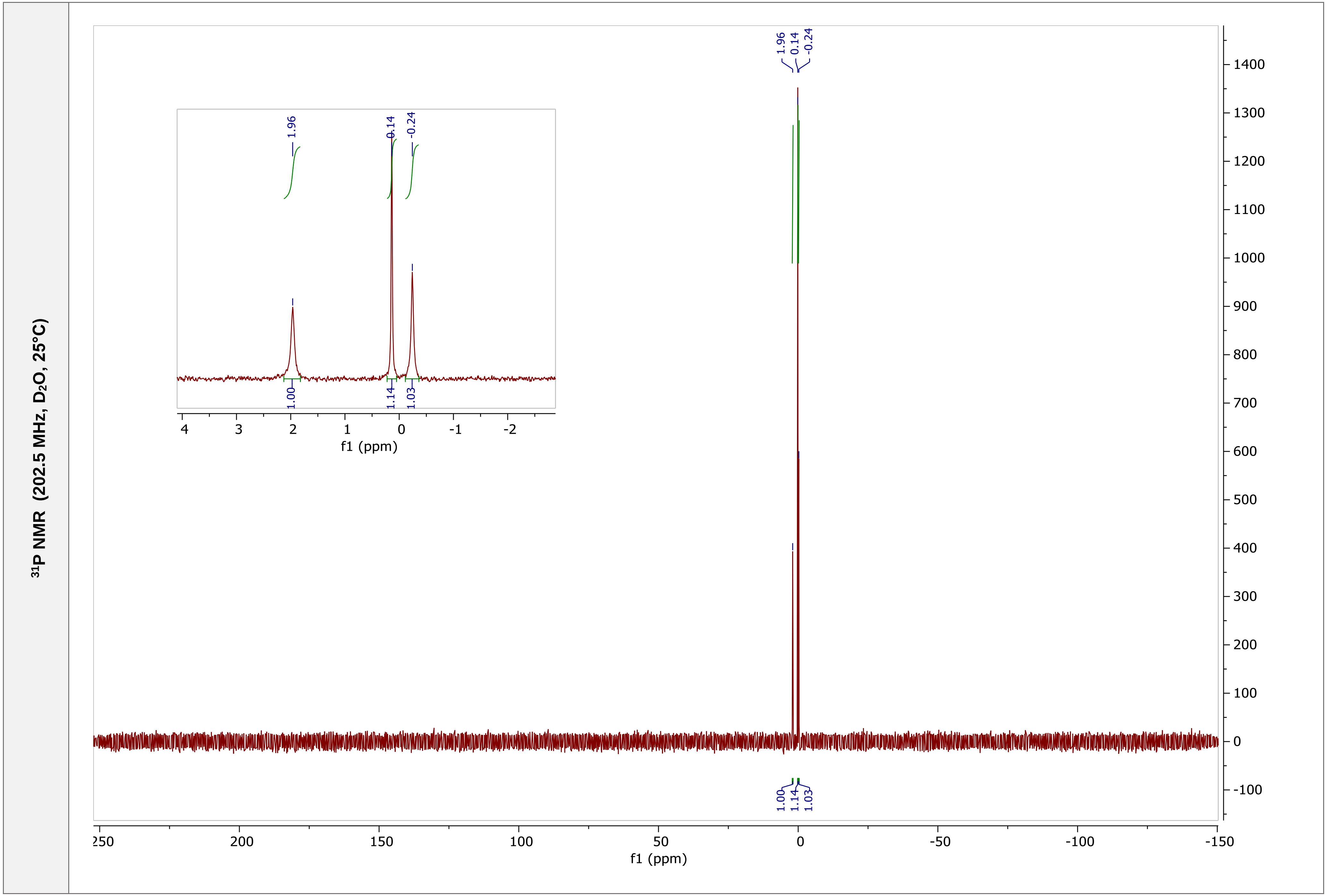

^{31}P NMR (202.5 MHz, D_2O, 25°C)
1.96
0.14
-0.24
1.00
1.14
1.03
f1 (ppm)

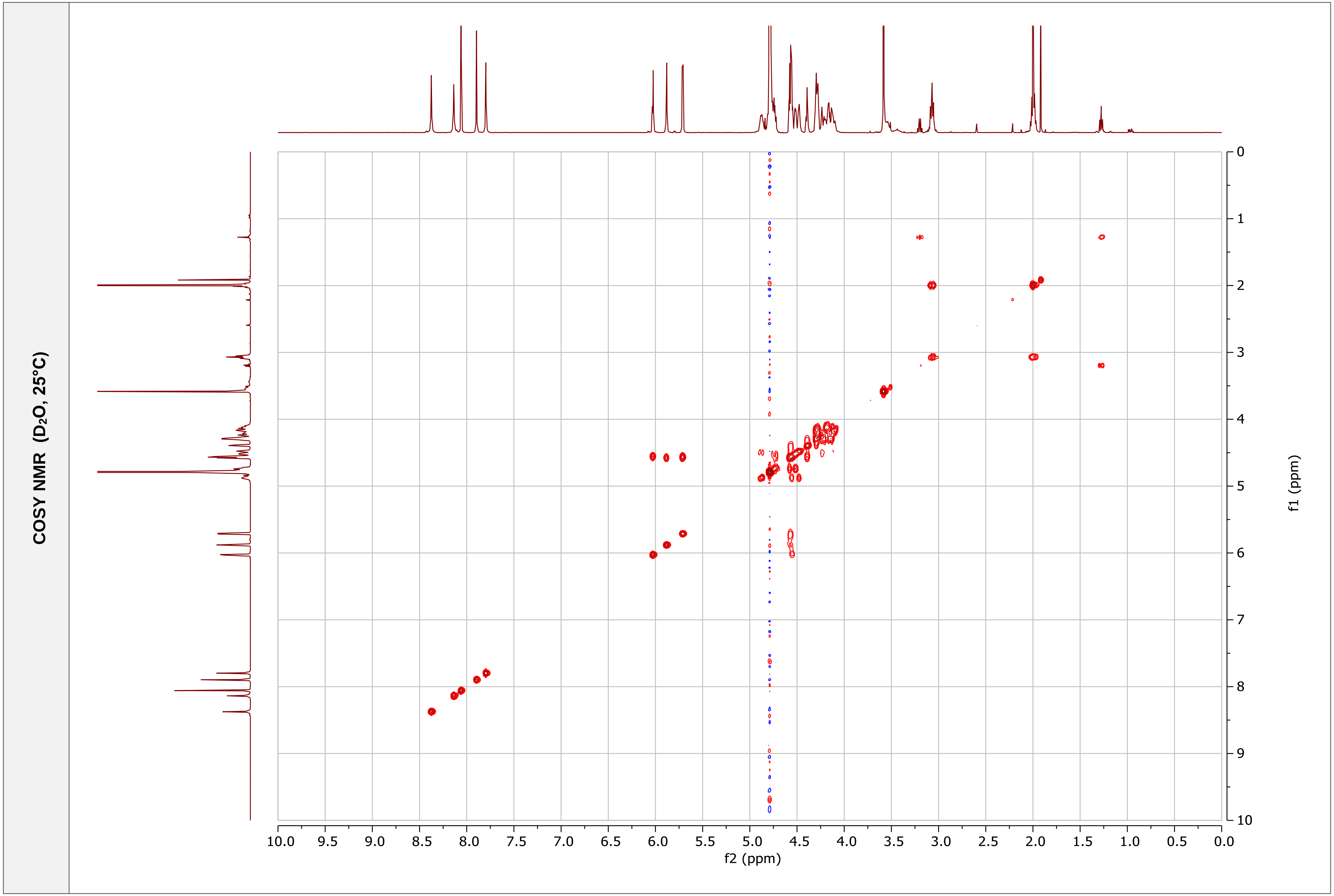

COSY NMR (D_2O, 25°C)
f2 (ppm)
f1 (ppm)

TOCSY (D$_2$O, 25°C)
(mixing time 120 ms)

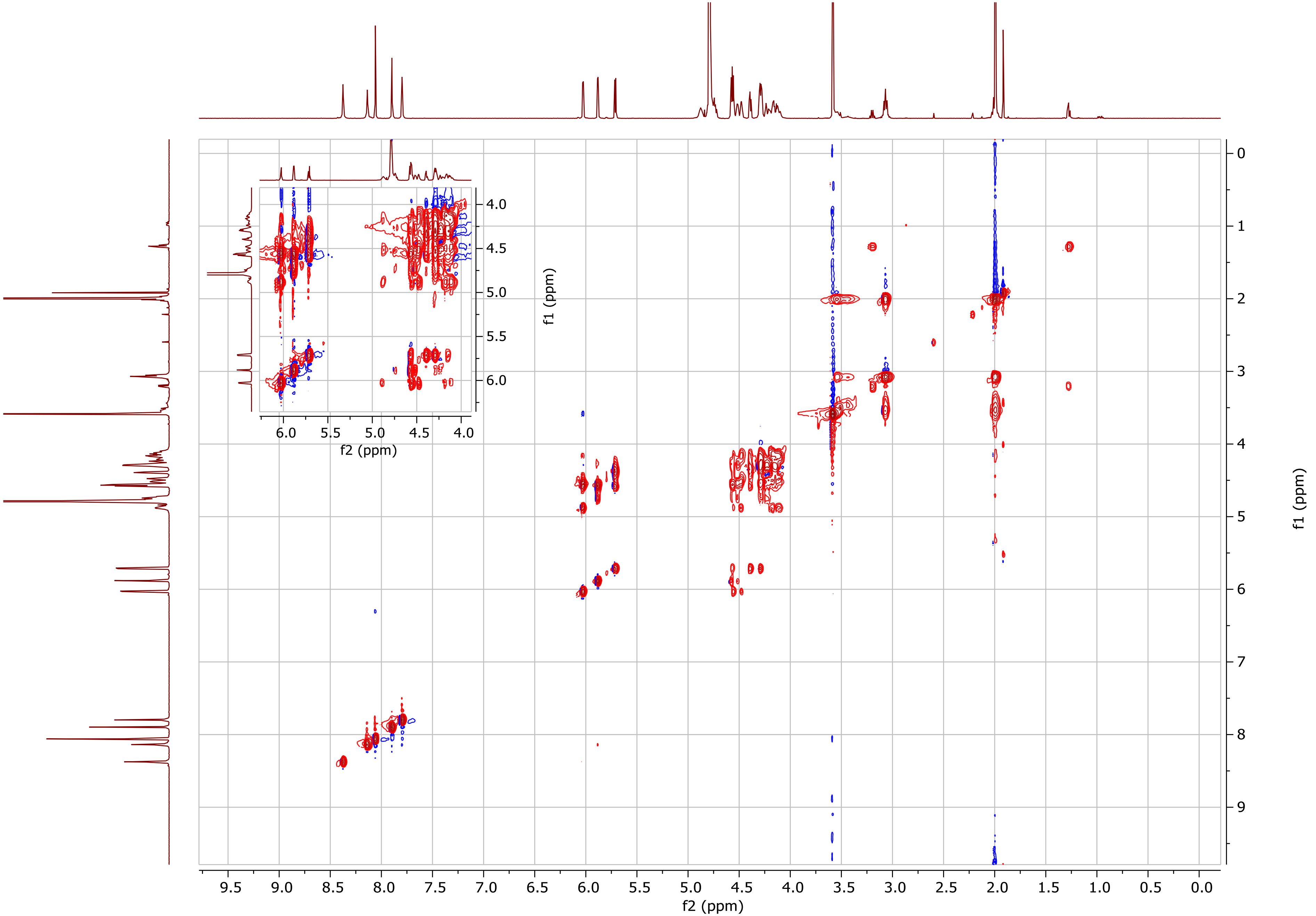

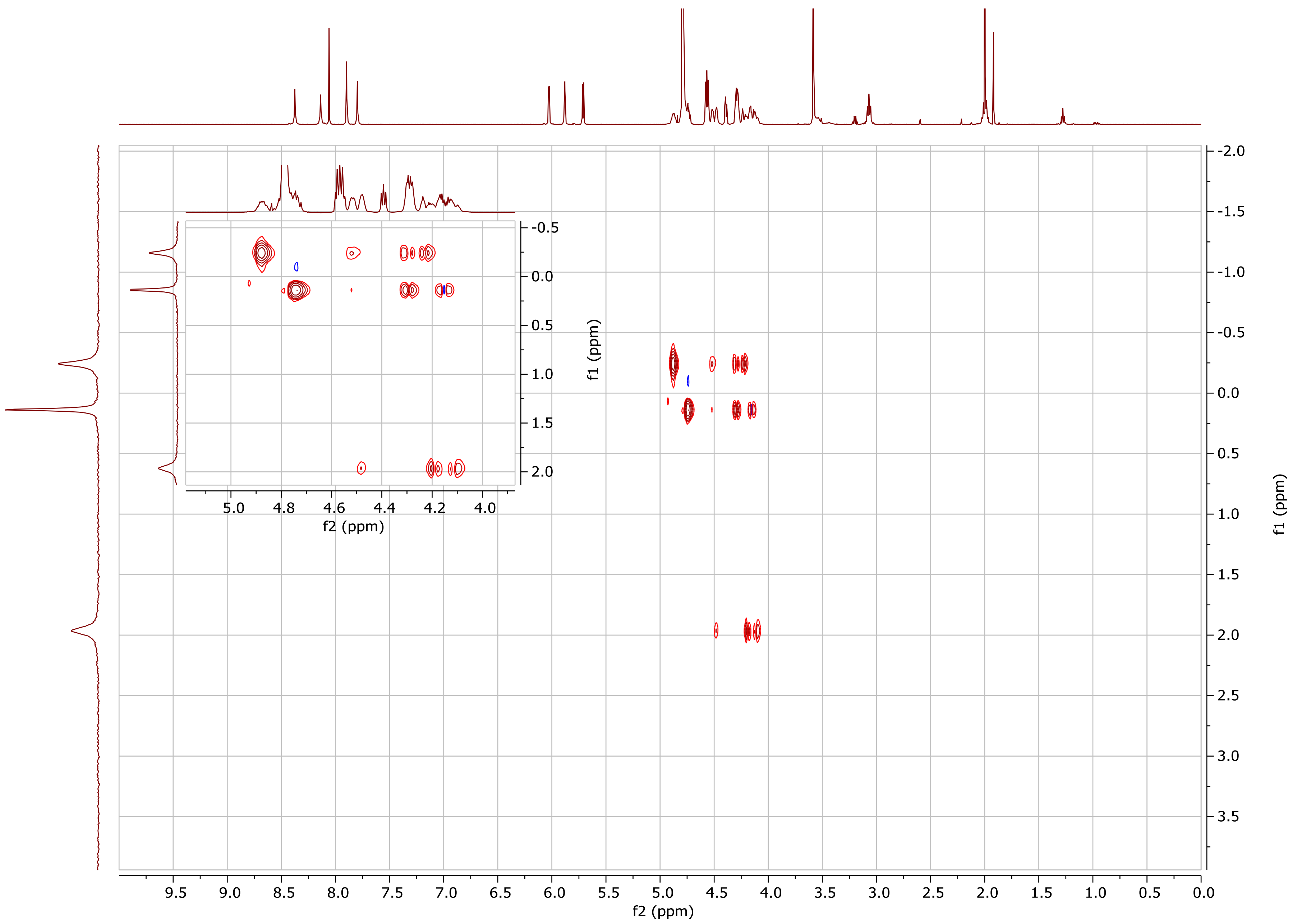

^{1}H-^{31}P HSQC (D_2O, 25°C)

(15) p^{iPr6}A$_m$pG

Chemical structure

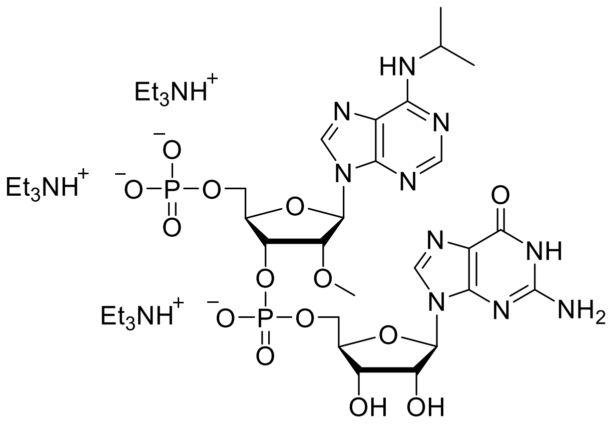

RP HPLC
Abs. @ 254 nm

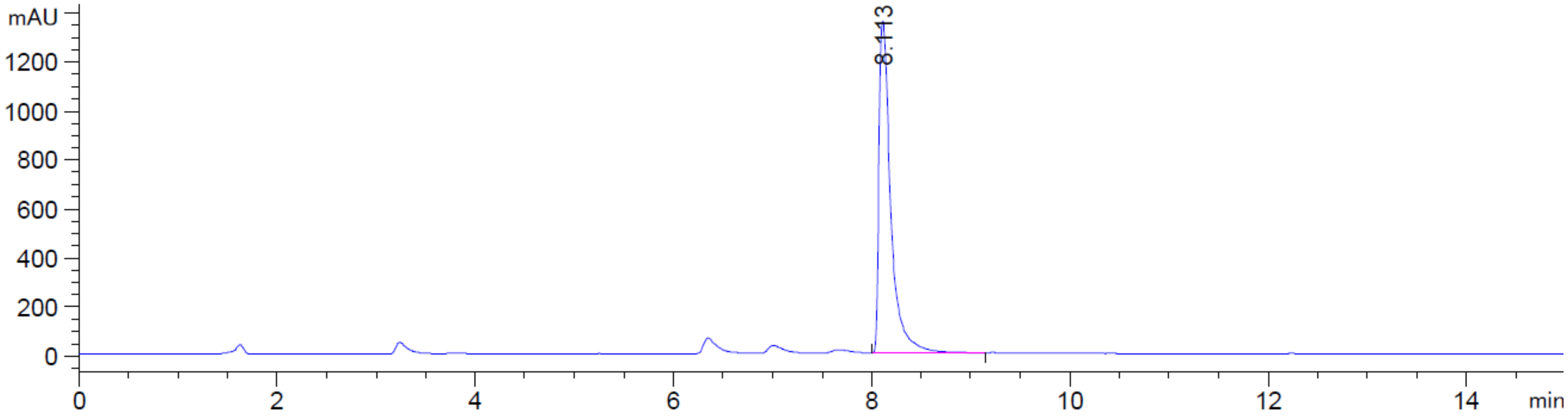

MS (-) ESI
(Calc. $[M-H]^- C_{24}H_{33}N_{10}O_{14}P_2^-$ 747.16584)

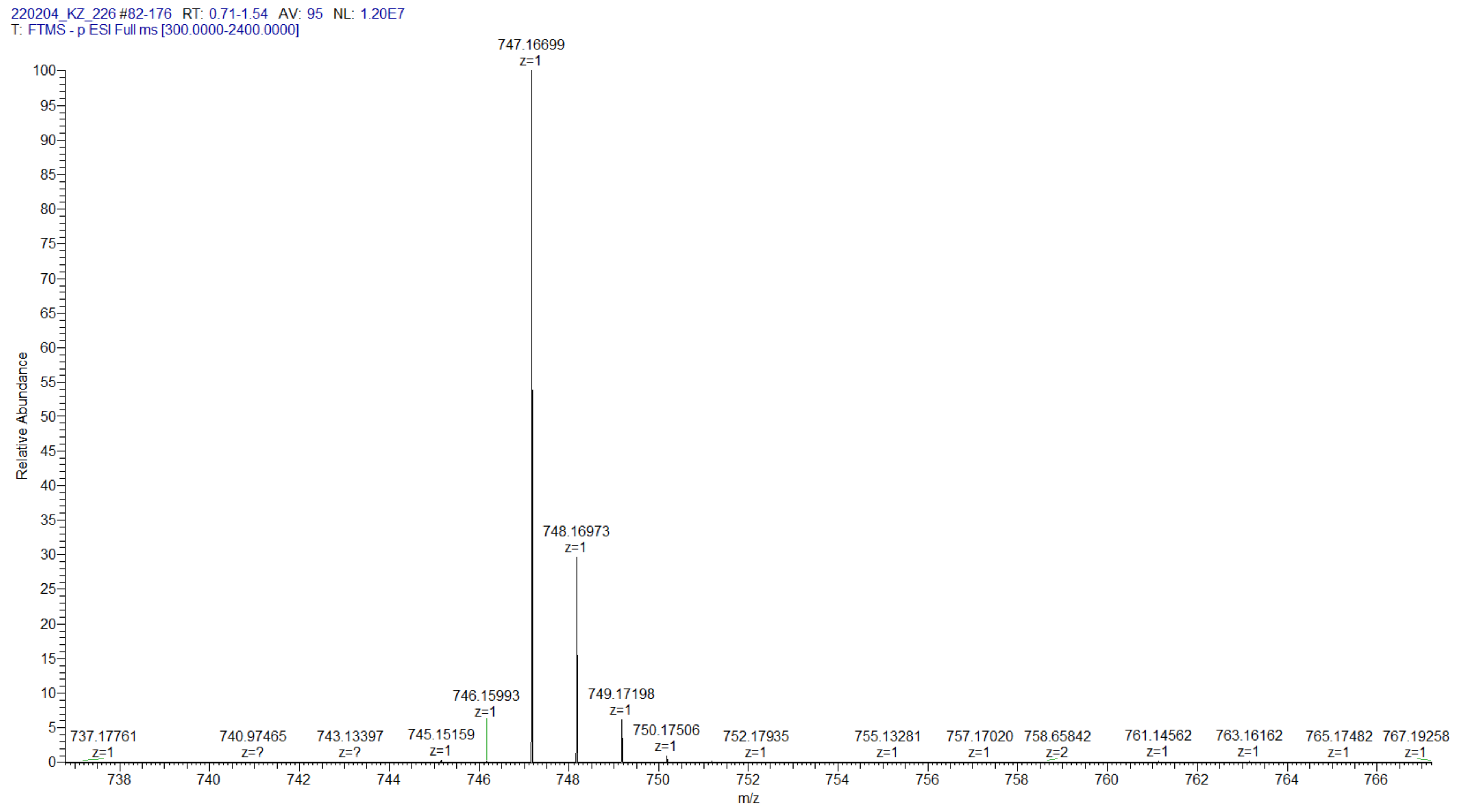

^1H NMR (500 MHz, D_2O, 25°C)

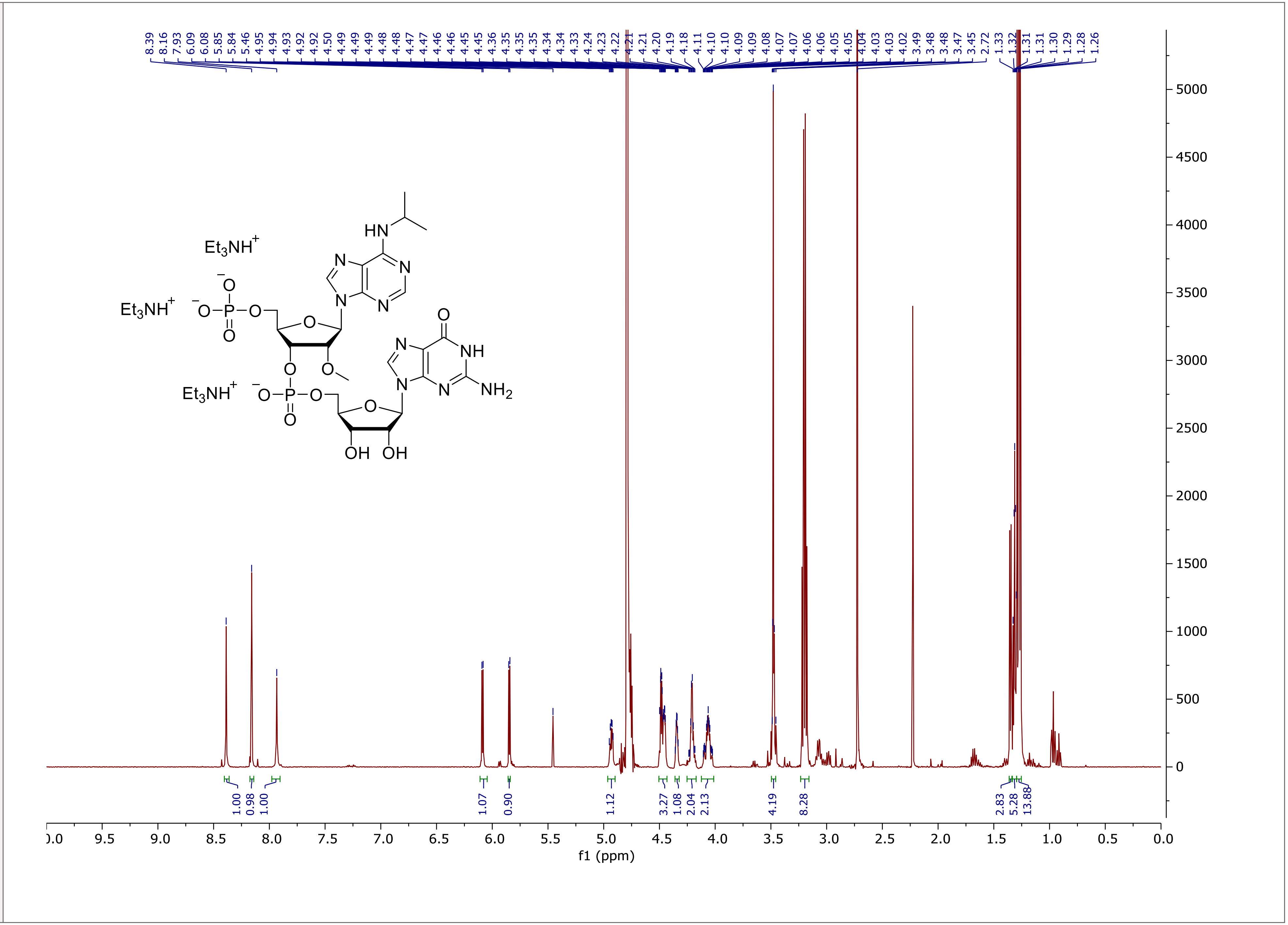

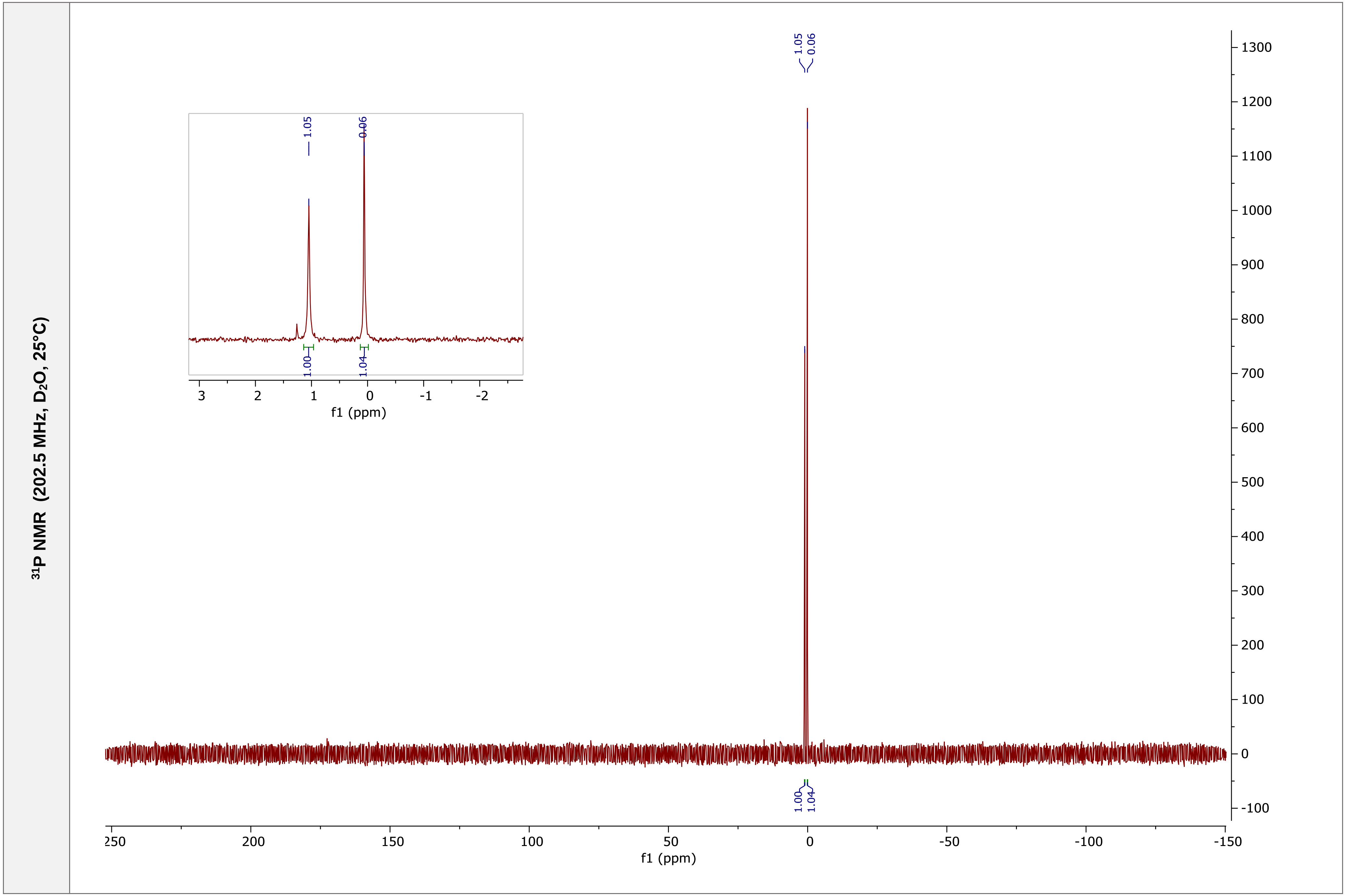

^{31}P NMR (202.5 MHz, D_2O, 25°C)
1.05
0.06
1.00
1.04
f1 (ppm)

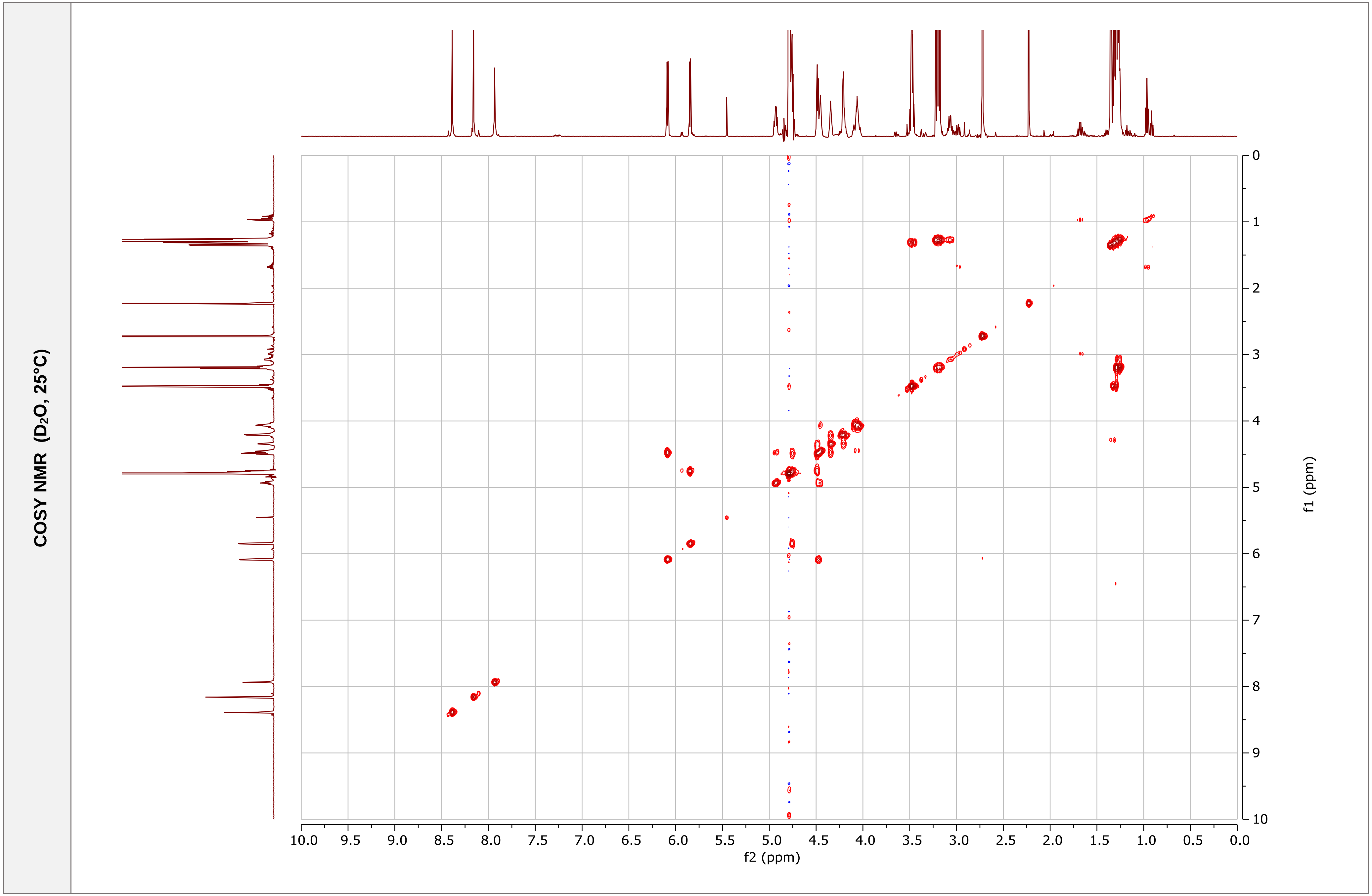

COSY NMR (D$_2$O, 25°C)
f2 (ppm)
f1 (ppm)

^{1}H-^{13}C HSQC (D$_2$O, 25°C)

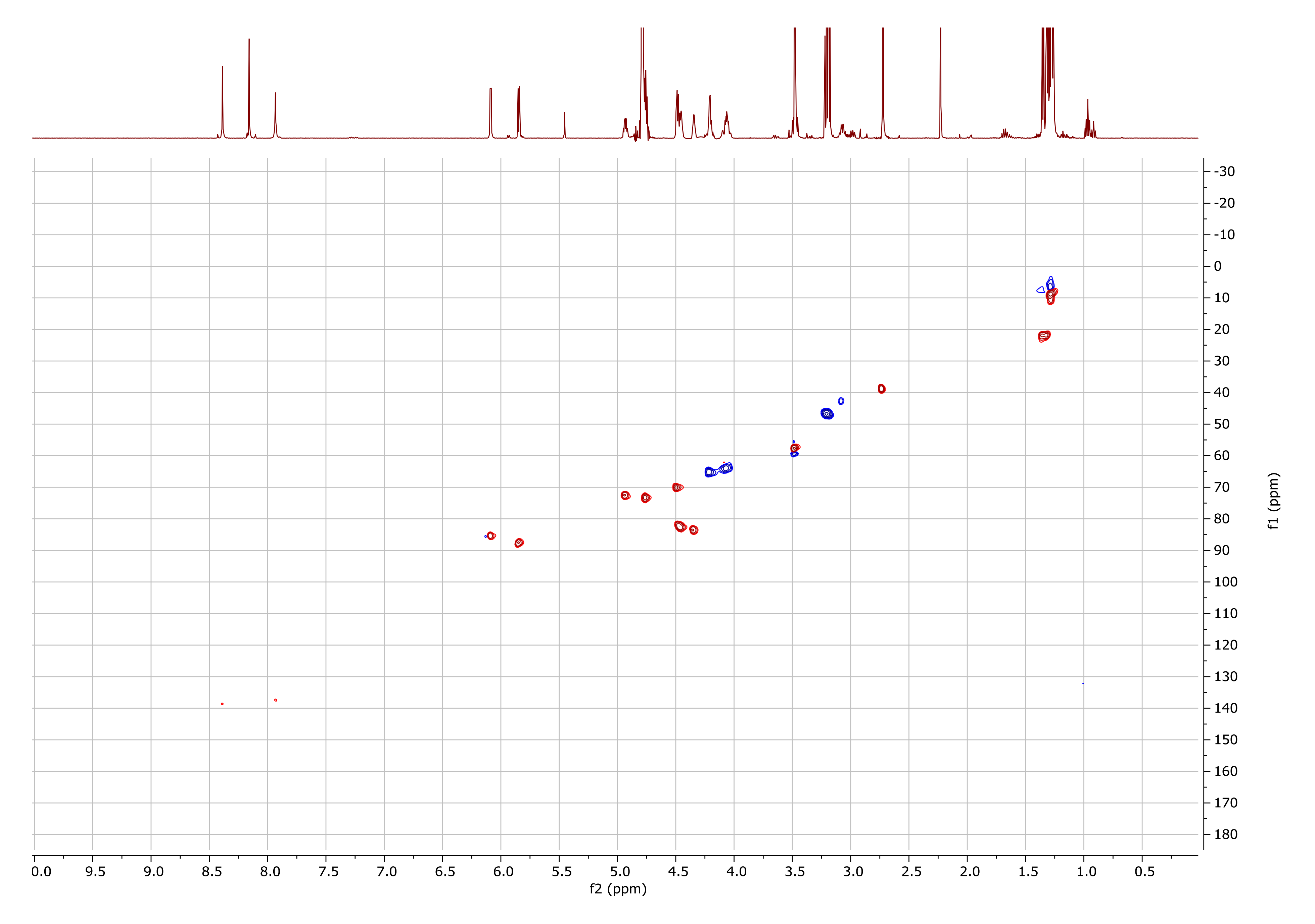

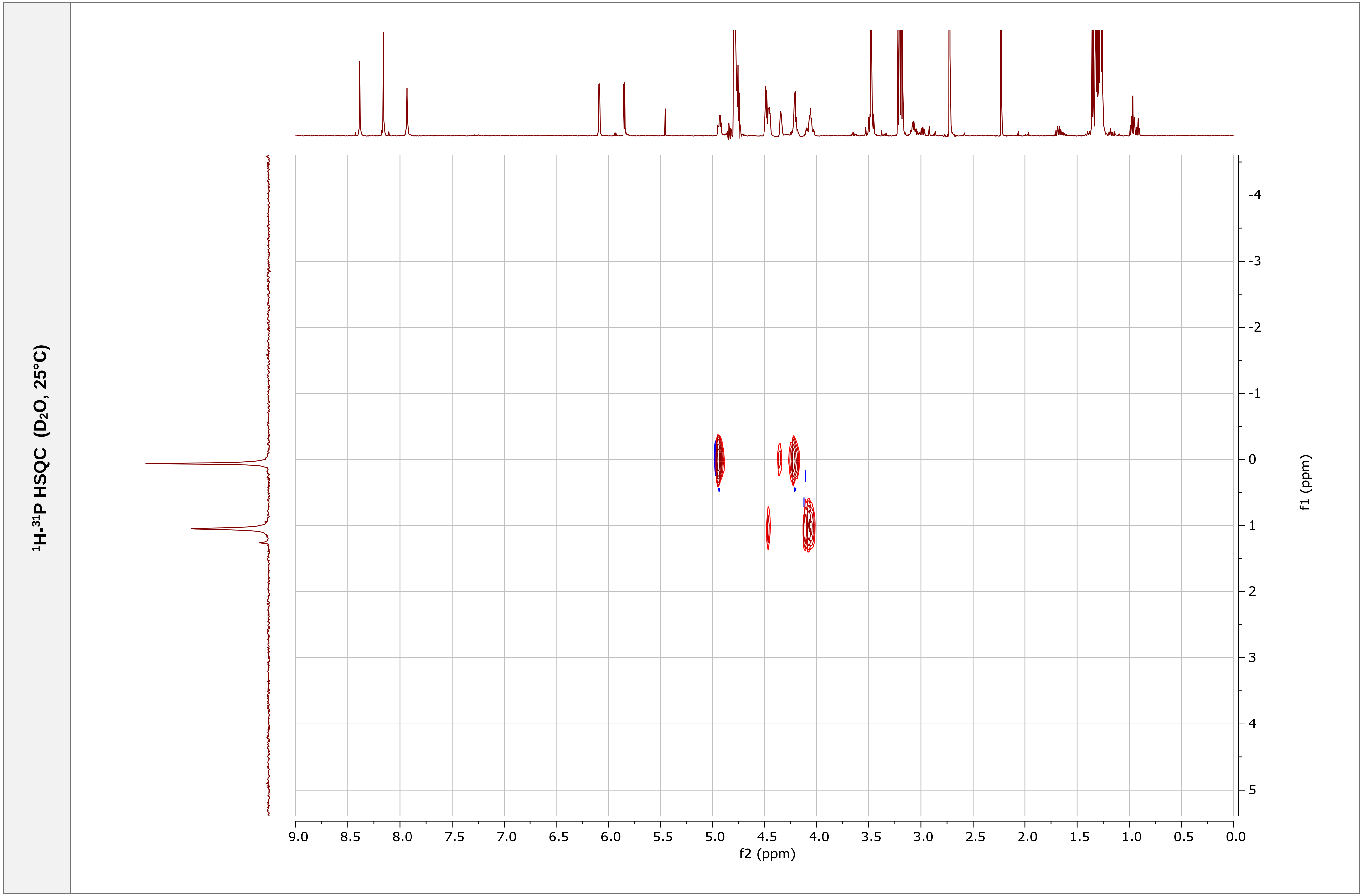

^1H-^{31}P HSQC (D$_2$O, 25°C)
f2 (ppm)
f1 (ppm)
9.0 8.5 8.0 7.5 7.0 6.5 6.0 5.5 5.0 4.5 4.0 3.5 3.0 2.5 2.0 1.5 1.0 0.5 0.0
-4 -3 -2 -1 0 1 2 3 4 5

(16) $p^{PhNCO6}ApG$	
**Chemical structure**	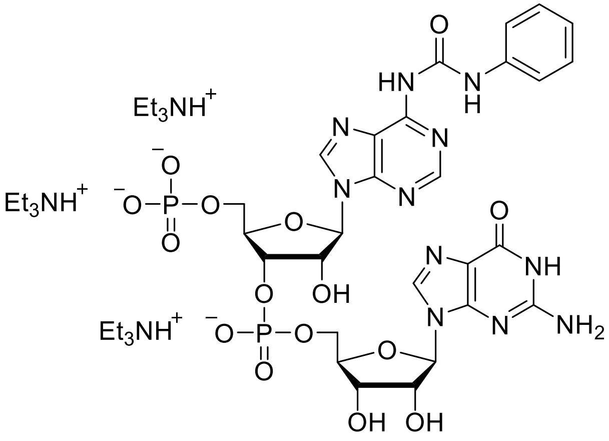
**RP HPLC** Abs. @ 254 nm	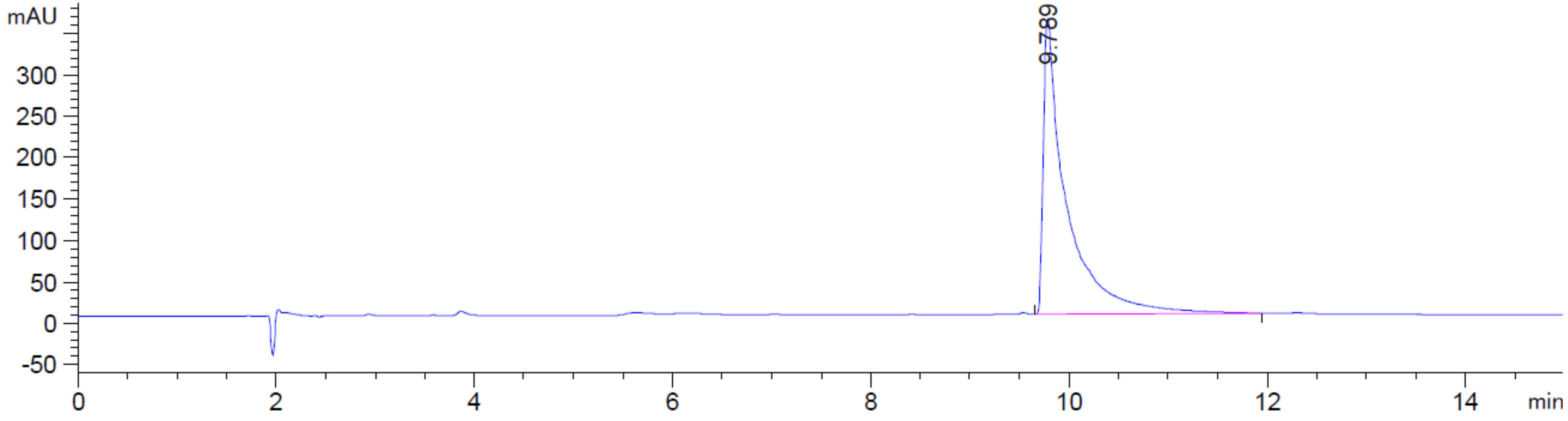

MS (-) ESI

(Calc. [M-H]$^-$ $C_{27}H_{30}N_{11}O_{15}P_2^-$ 810.14036)

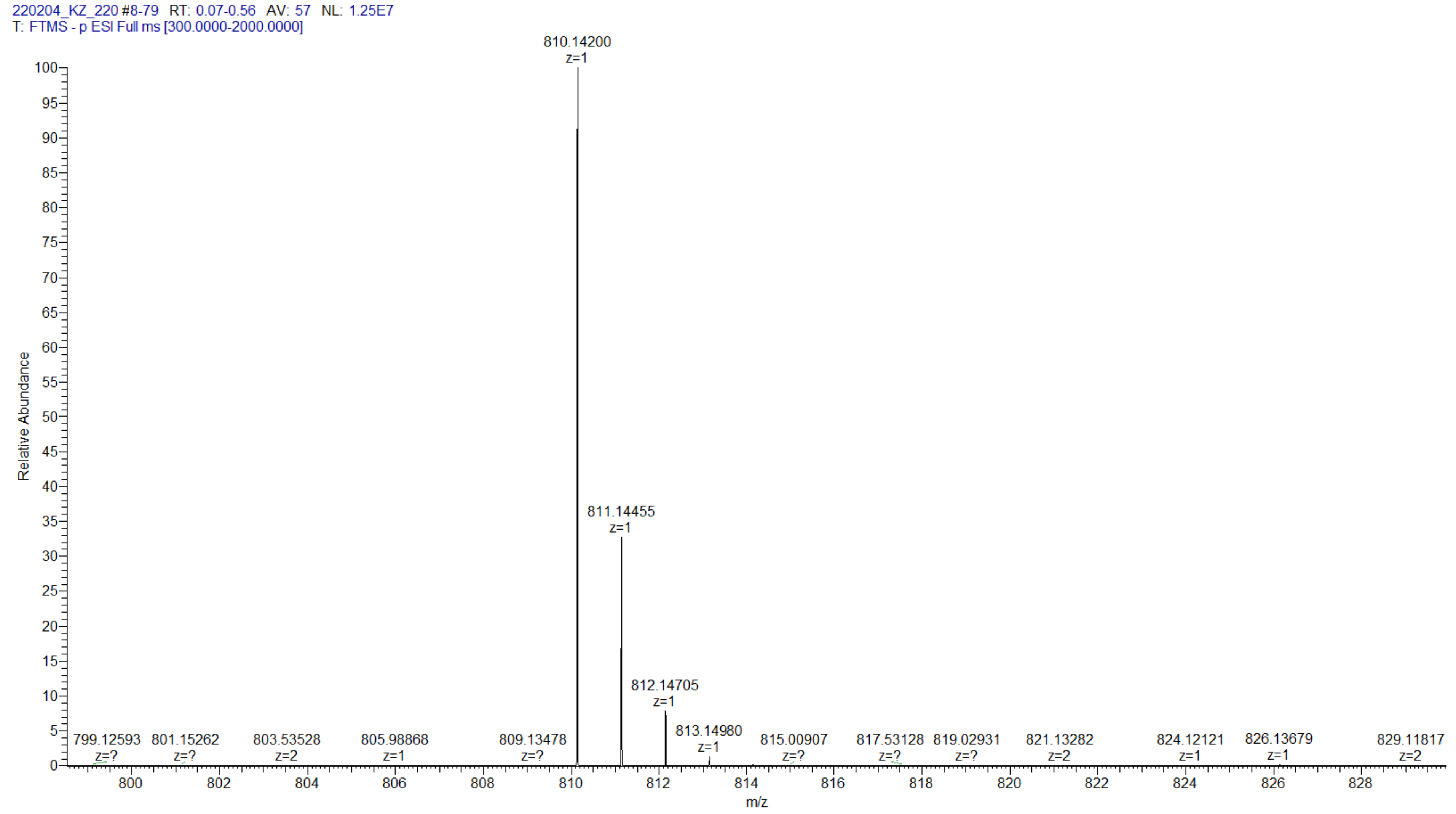

^{1}H NMR (500 MHz, D_2O, 25°C)

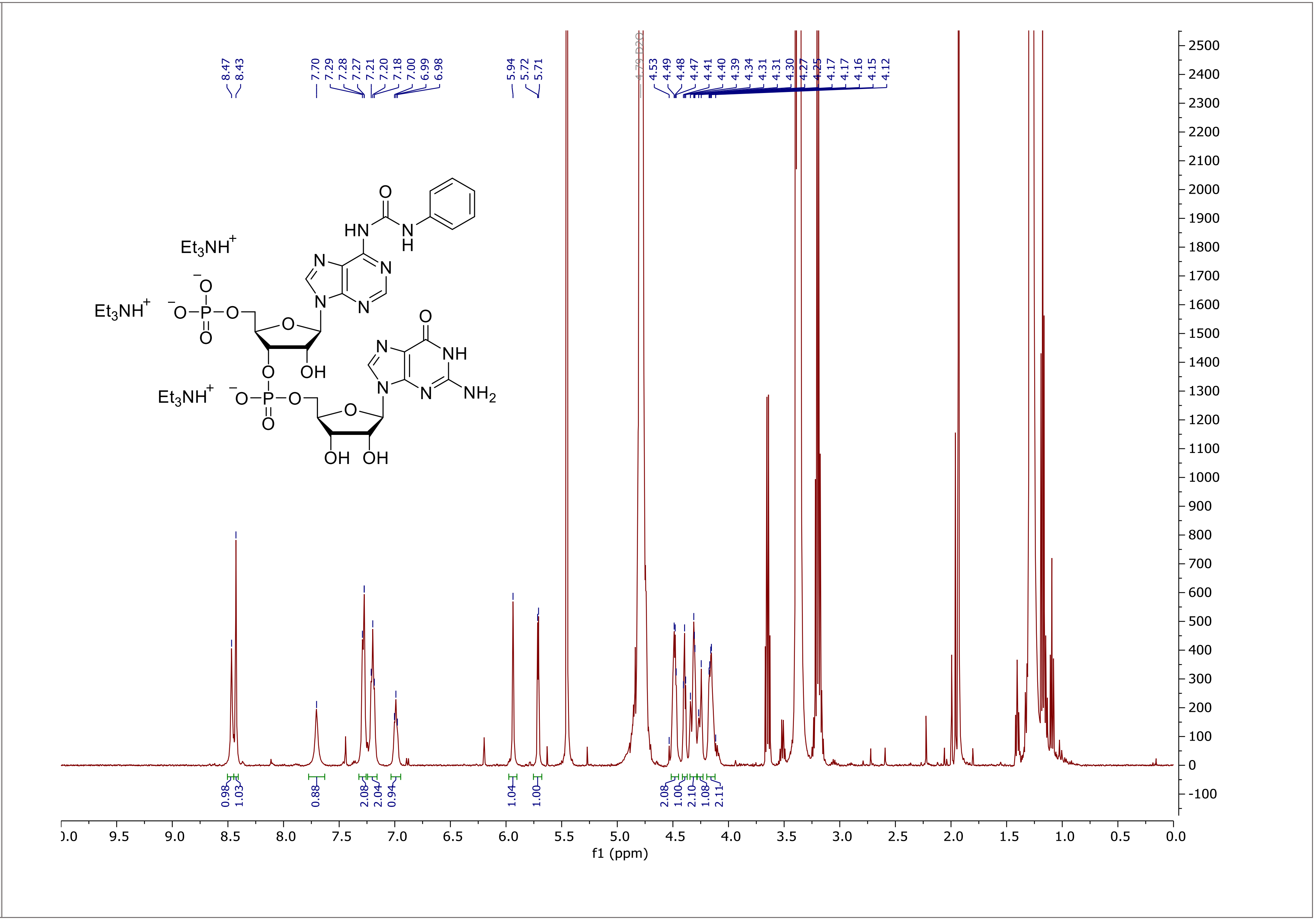

^{31}P NMR (202.5 MHz, D_2O, 25°C)

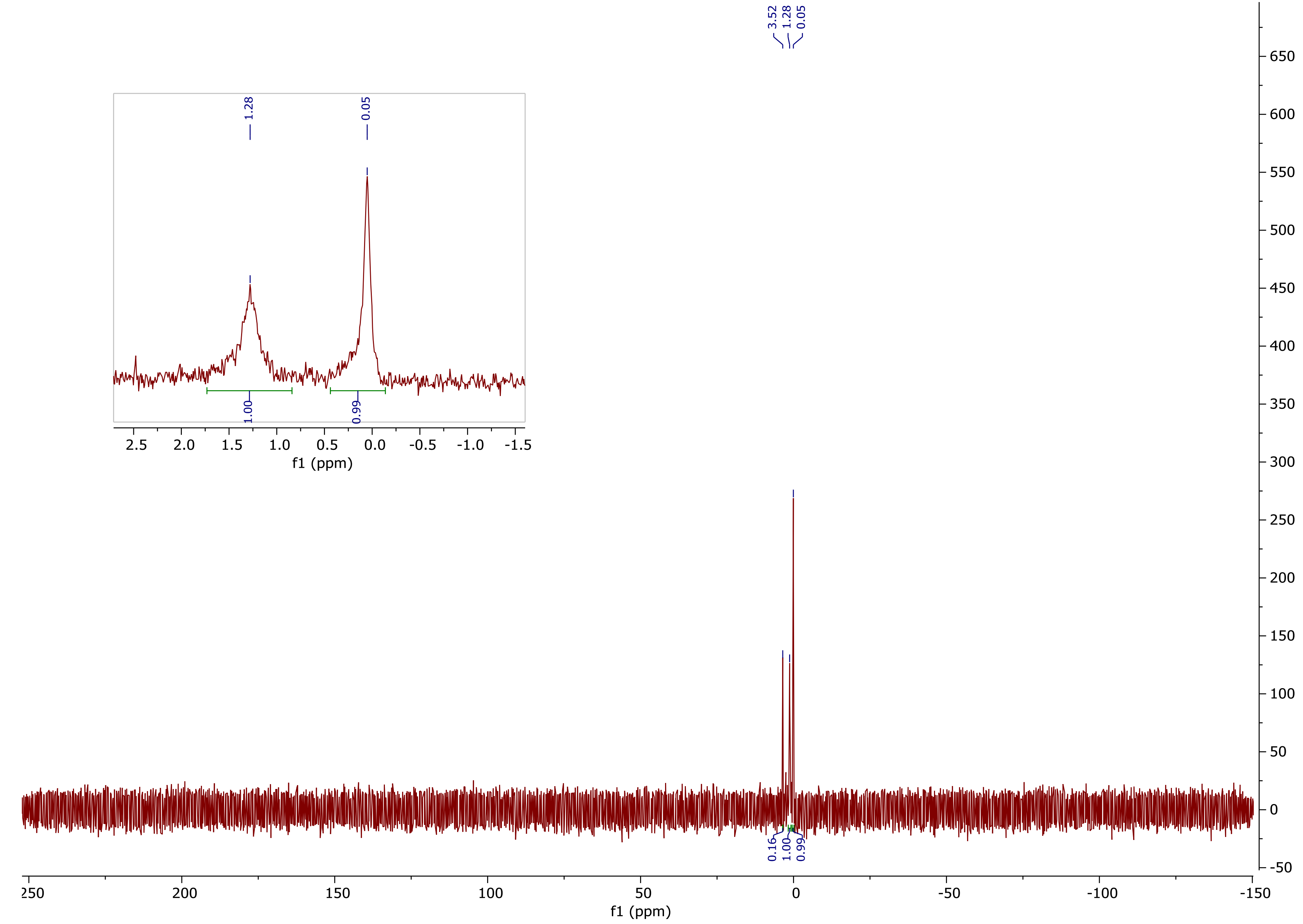

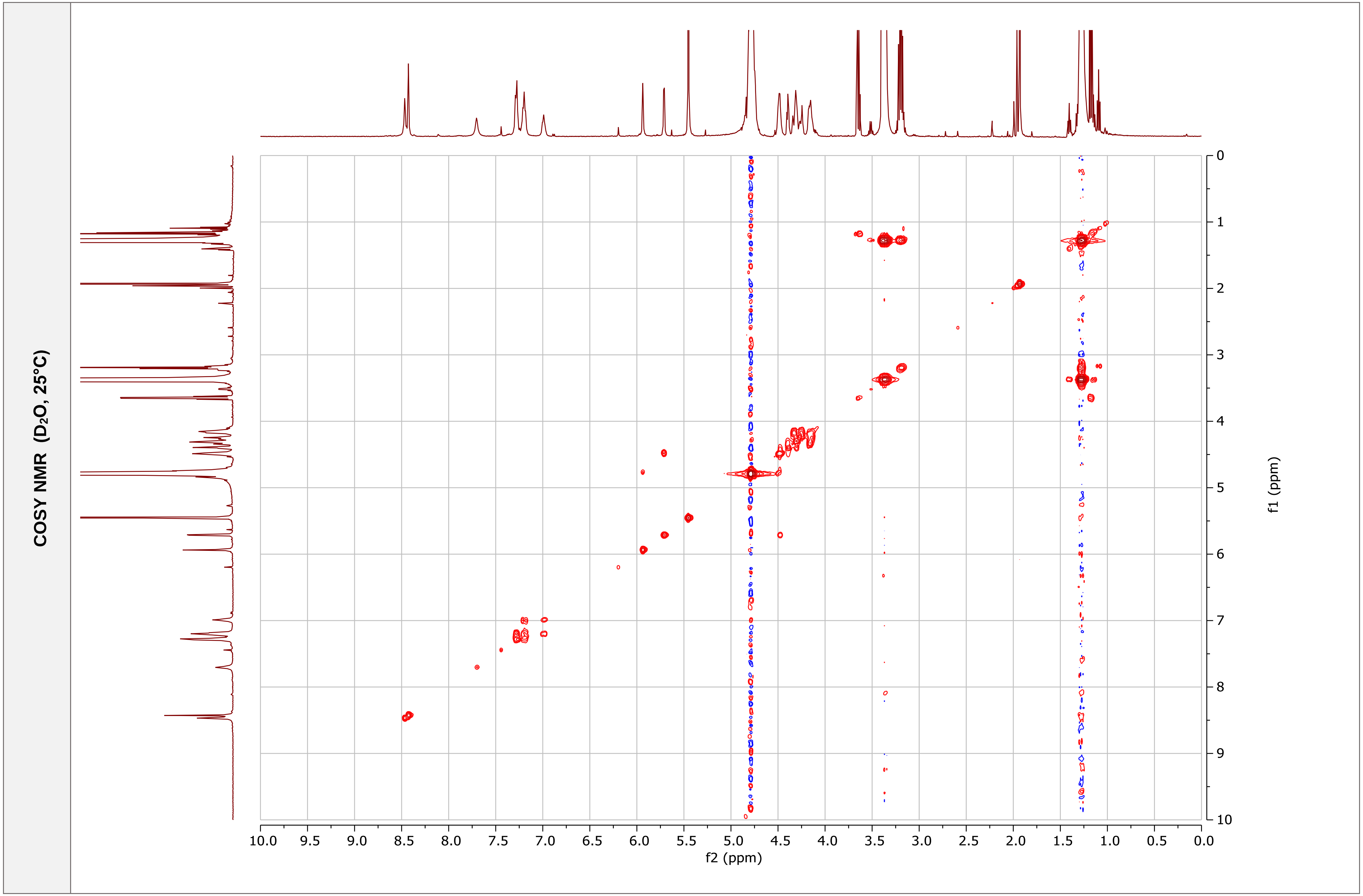

COSY NMR (D$_2$O, 25°C)
f2 (ppm)
f1 (ppm)
10.0 9.5 9.0 8.5 8.0 7.5 7.0 6.5 6.0 5.5 5.0 4.5 4.0 3.5 3.0 2.5 2.0 1.5 1.0 0.5 0.0
0 1 2 3 4 5 6 7 8 9 10

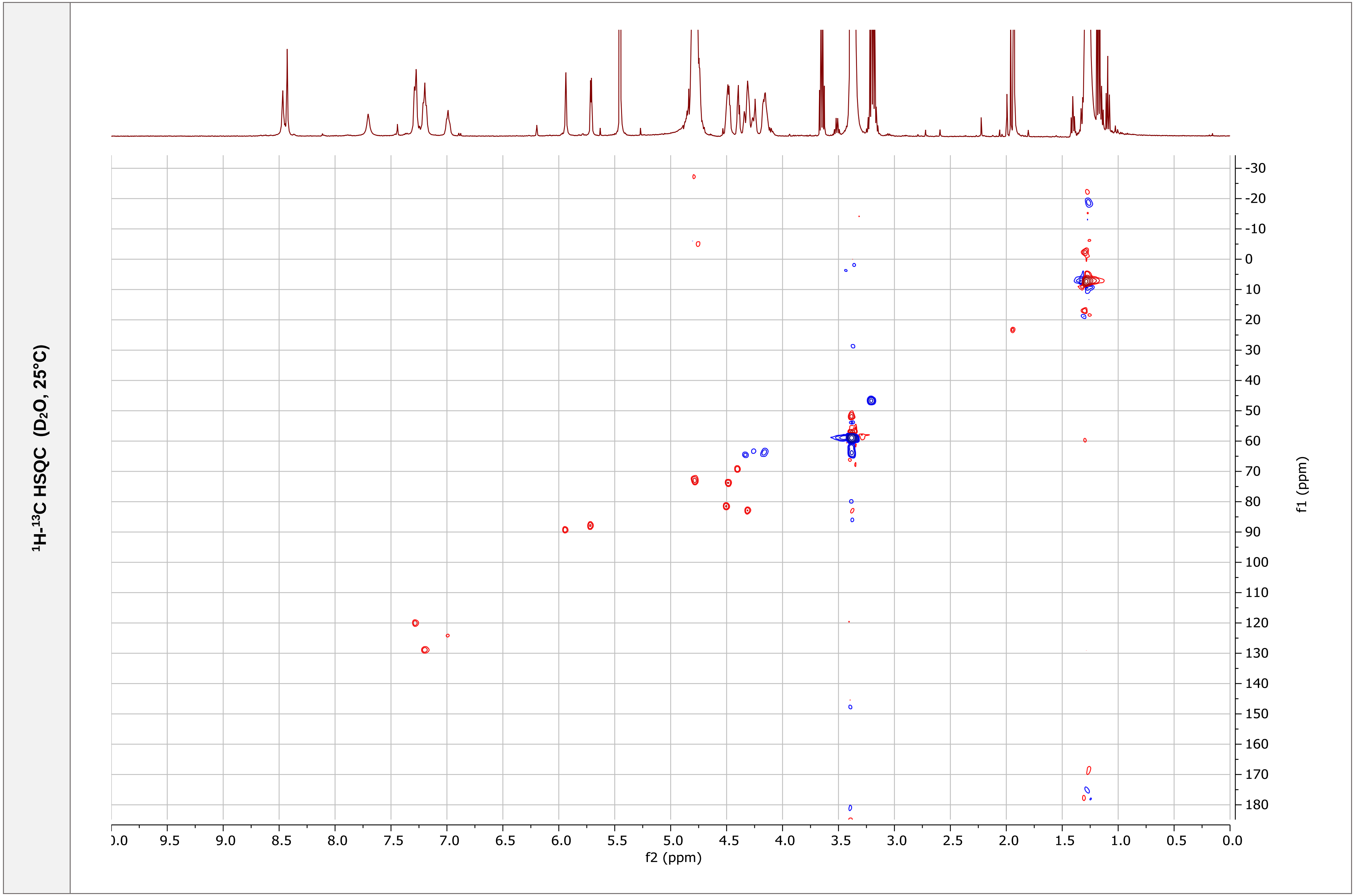

^1H-^{13}C HSQC (D$_2$O, 25°C)
f2 (ppm)
f1 (ppm)

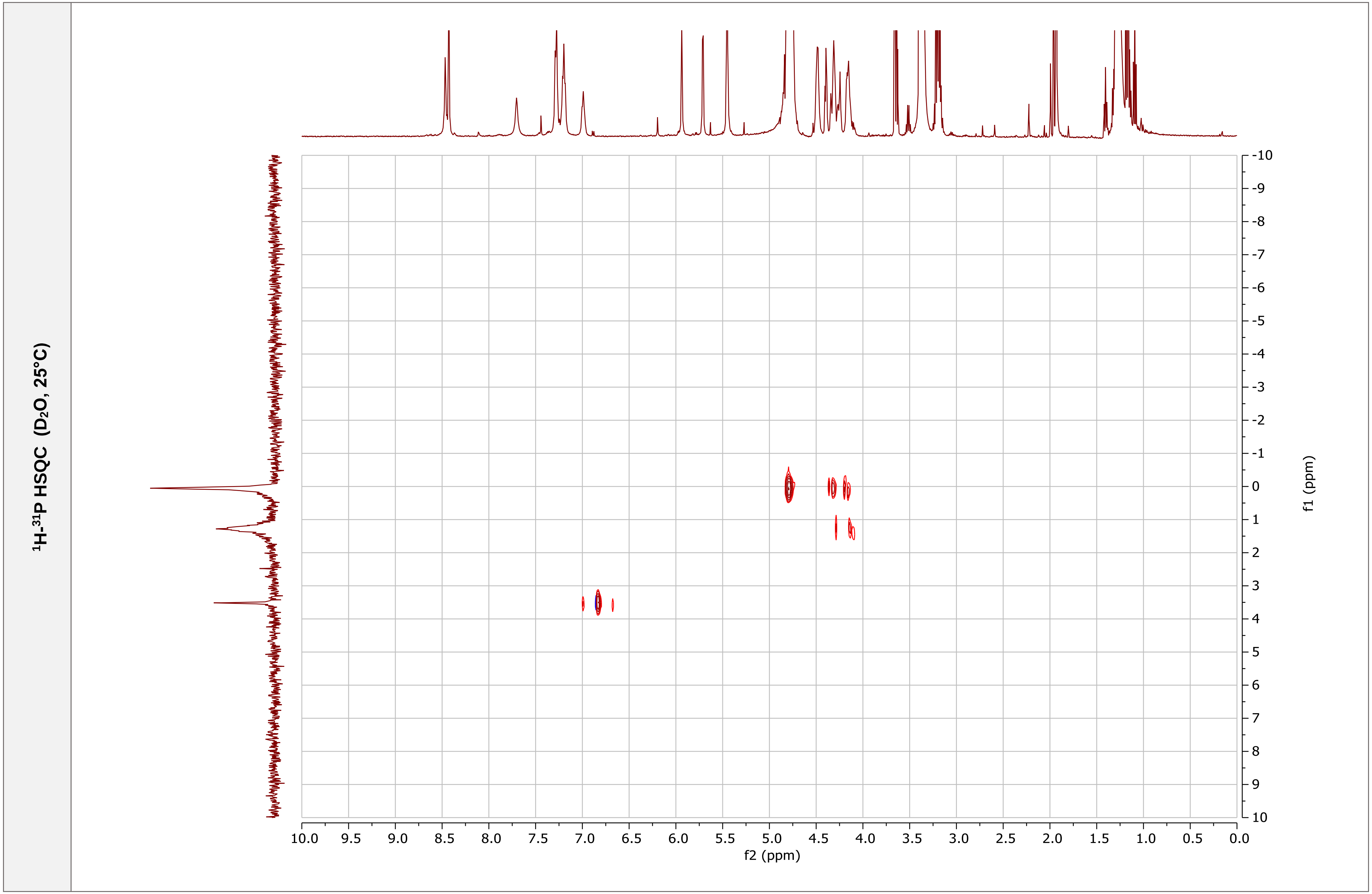

^1H-^{31}P HSQC (D$_2$O, 25°C)
f2 (ppm)
f1 (ppm)

(17) $U^{g6}AU$	
**Chemical structure**	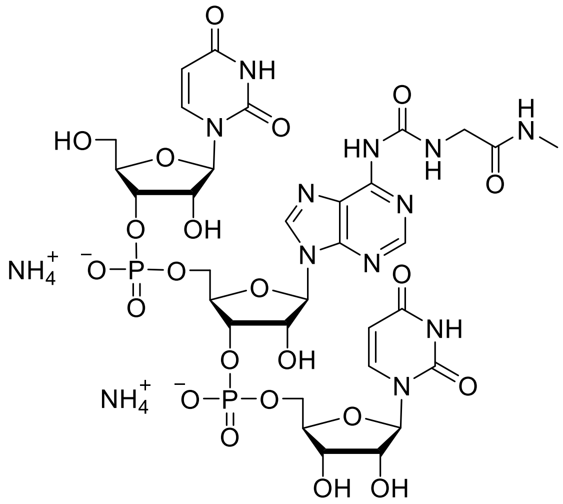
**RP HPLC** Abs. @ 254 nm	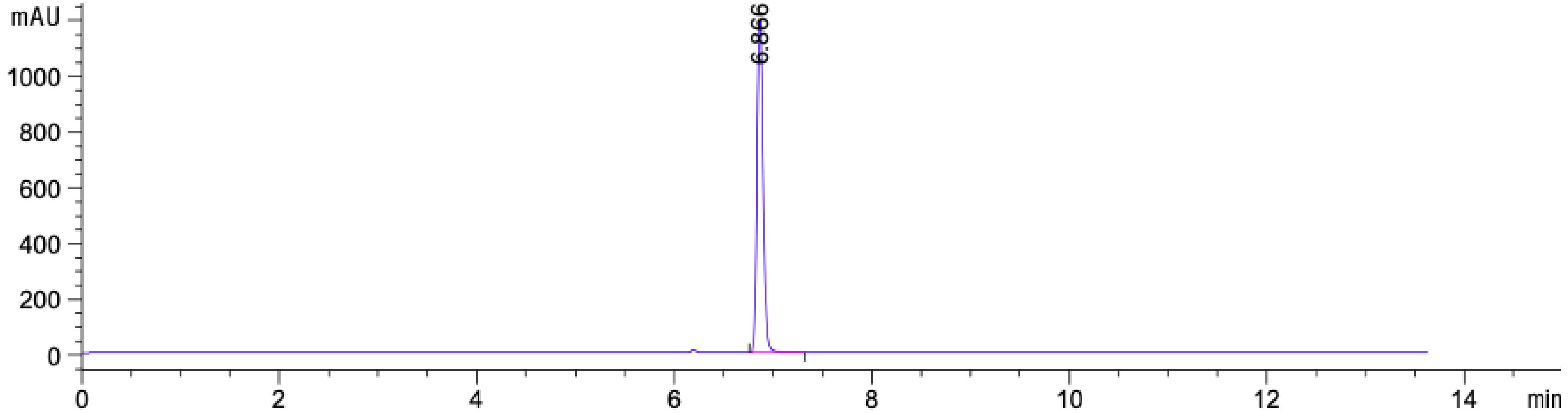

MS (-) ESI

(Calc. [M-H]$^-$ $C_{32}H_{40}N_{11}O_{22}P_2^-$ 992.18301)

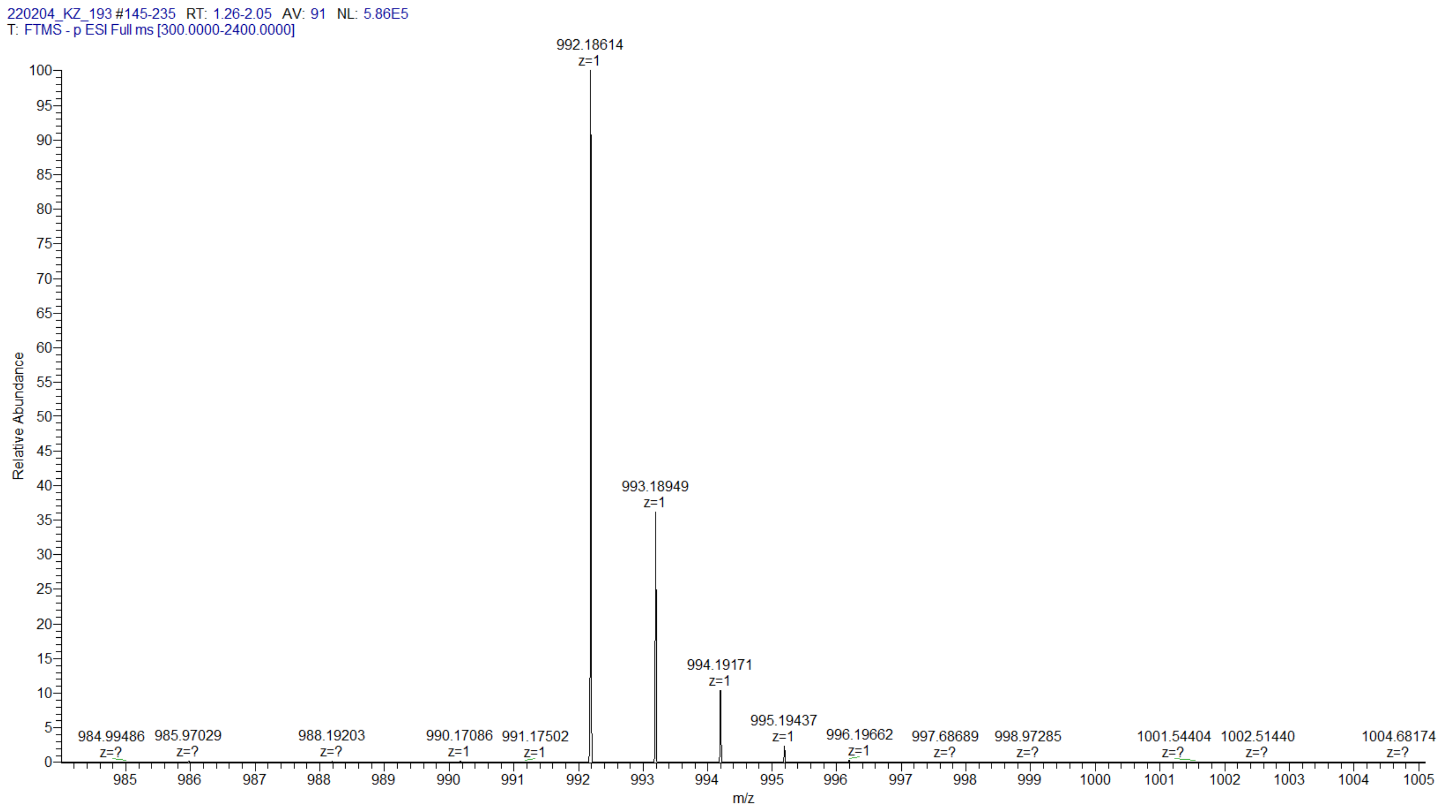

^{1}H NMR (500 MHz, D_2O, 25°C)

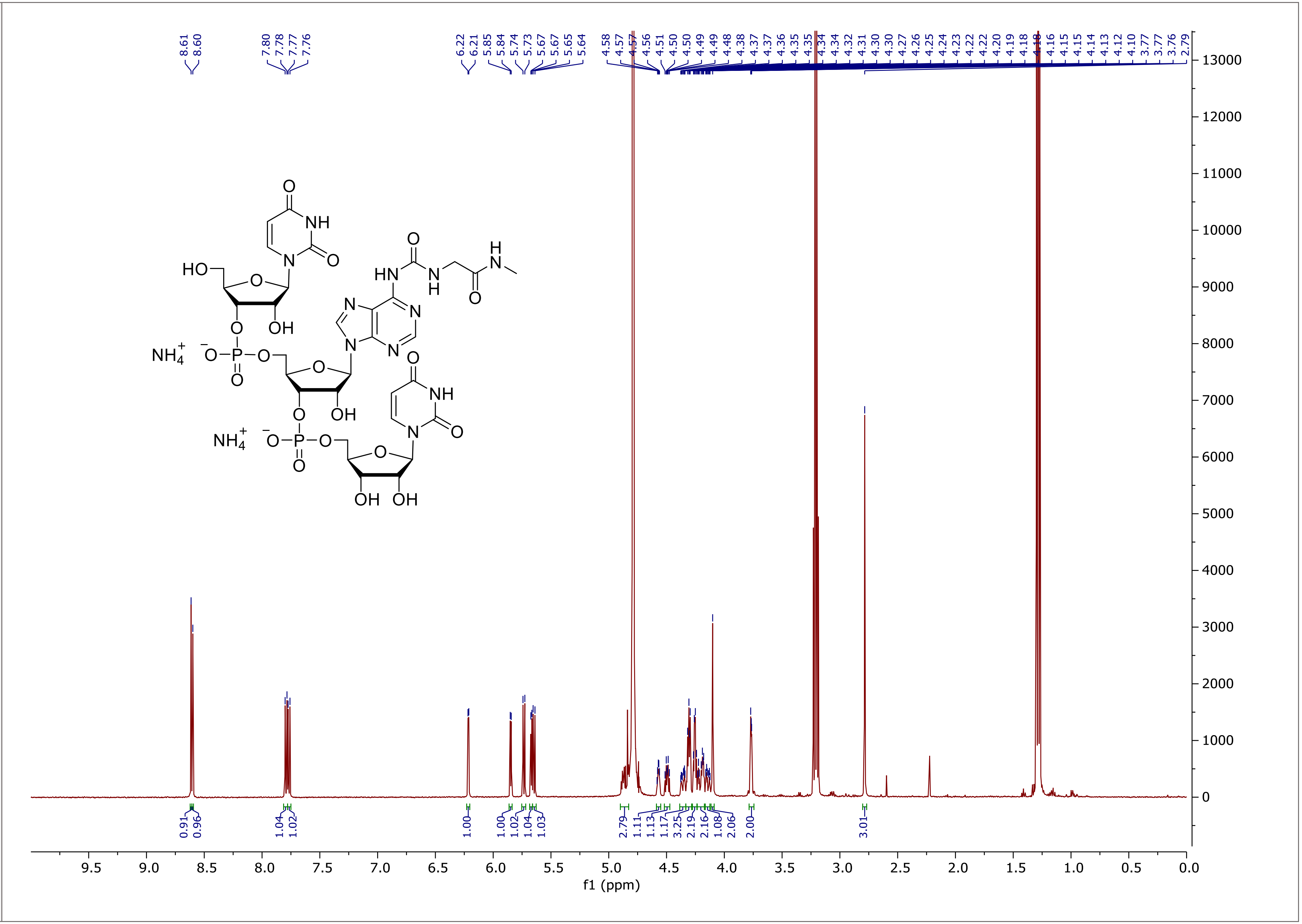

^{31}P NMR (202.5 MHz, D_2O, 25°C)

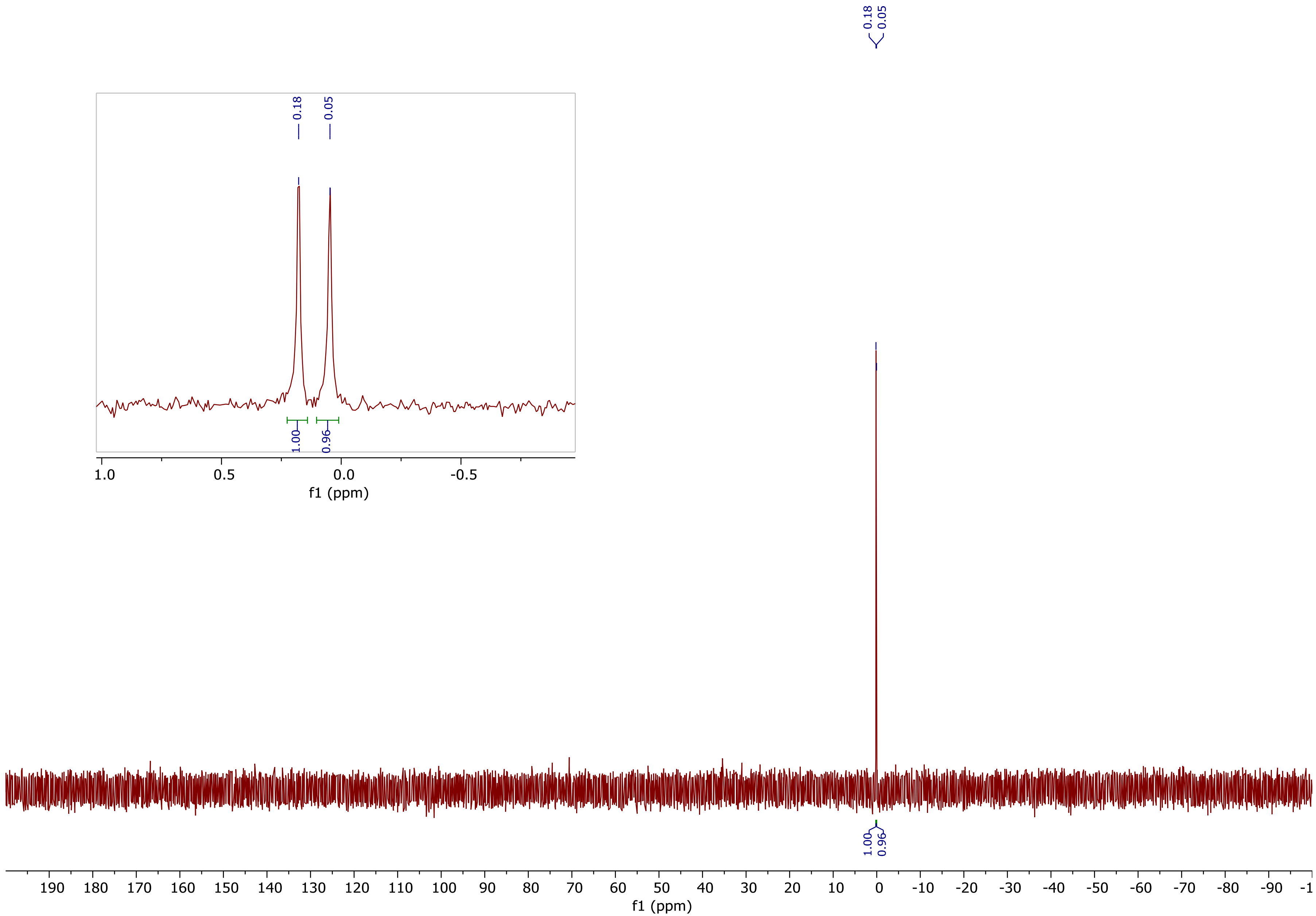

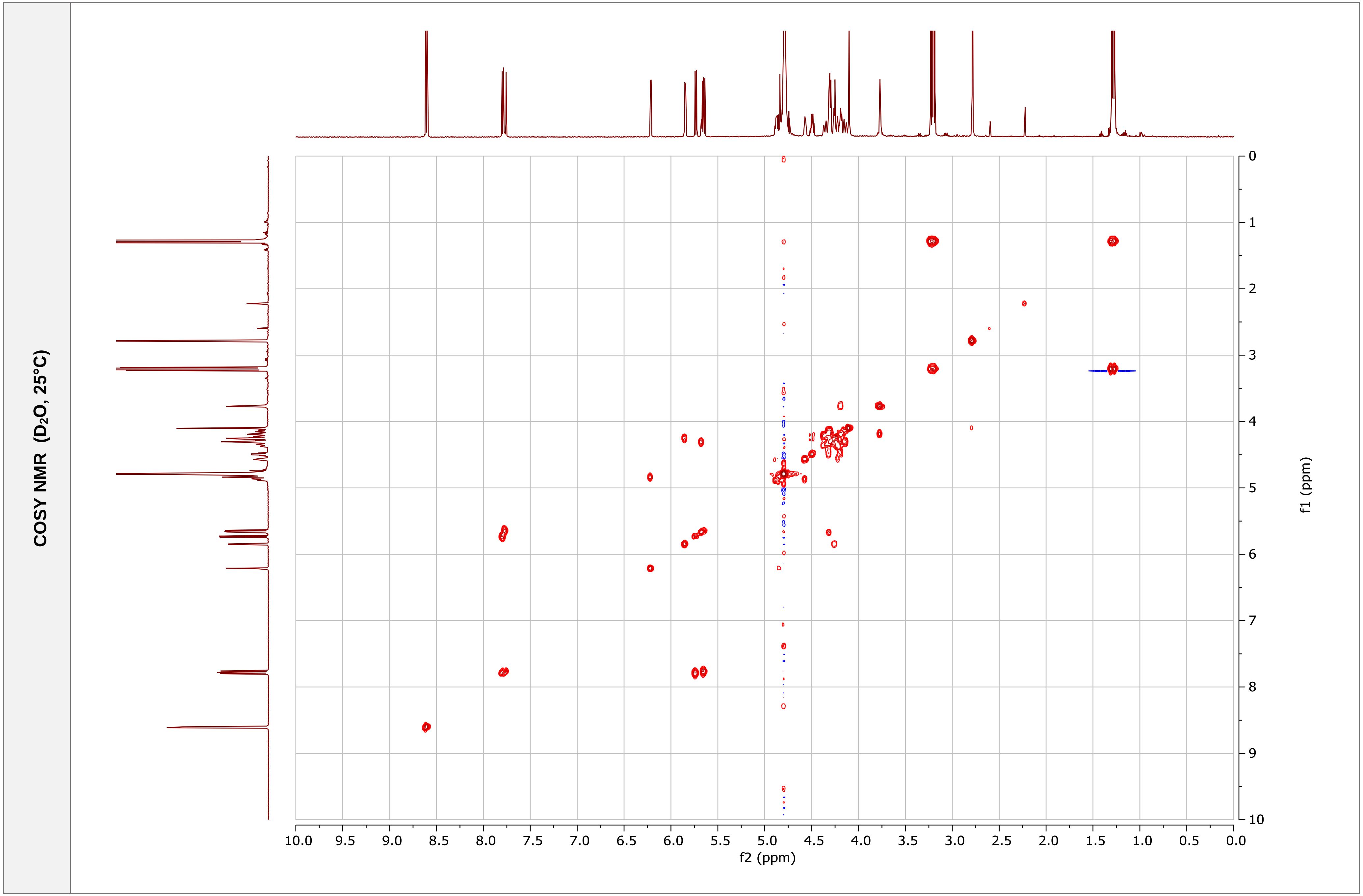
COSY NMR (D2O, 25°C)
f2 (ppm)
f1 (ppm)
10.0
9.5
9.0
8.5
8.0
7.5
7.0
6.5
6.0
5.5
5.0
4.5
4.0
3.5
3.0
2.5
2.0
1.5
1.0
0.5
0.0
0
1
2
3
4
5
6
7
8
9
10

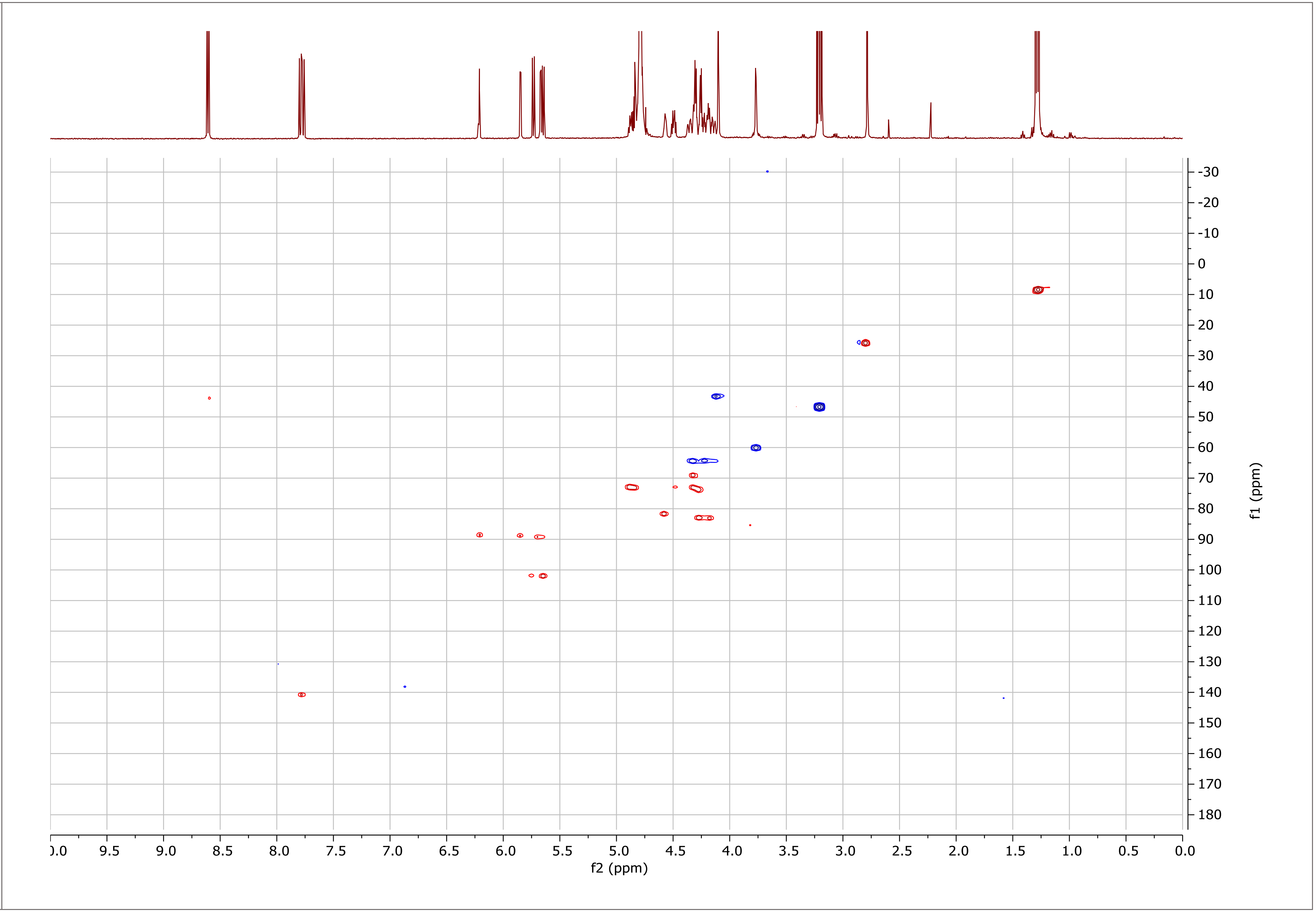

^1H-^{13}C HSQC (D$_2$O, 25°C)

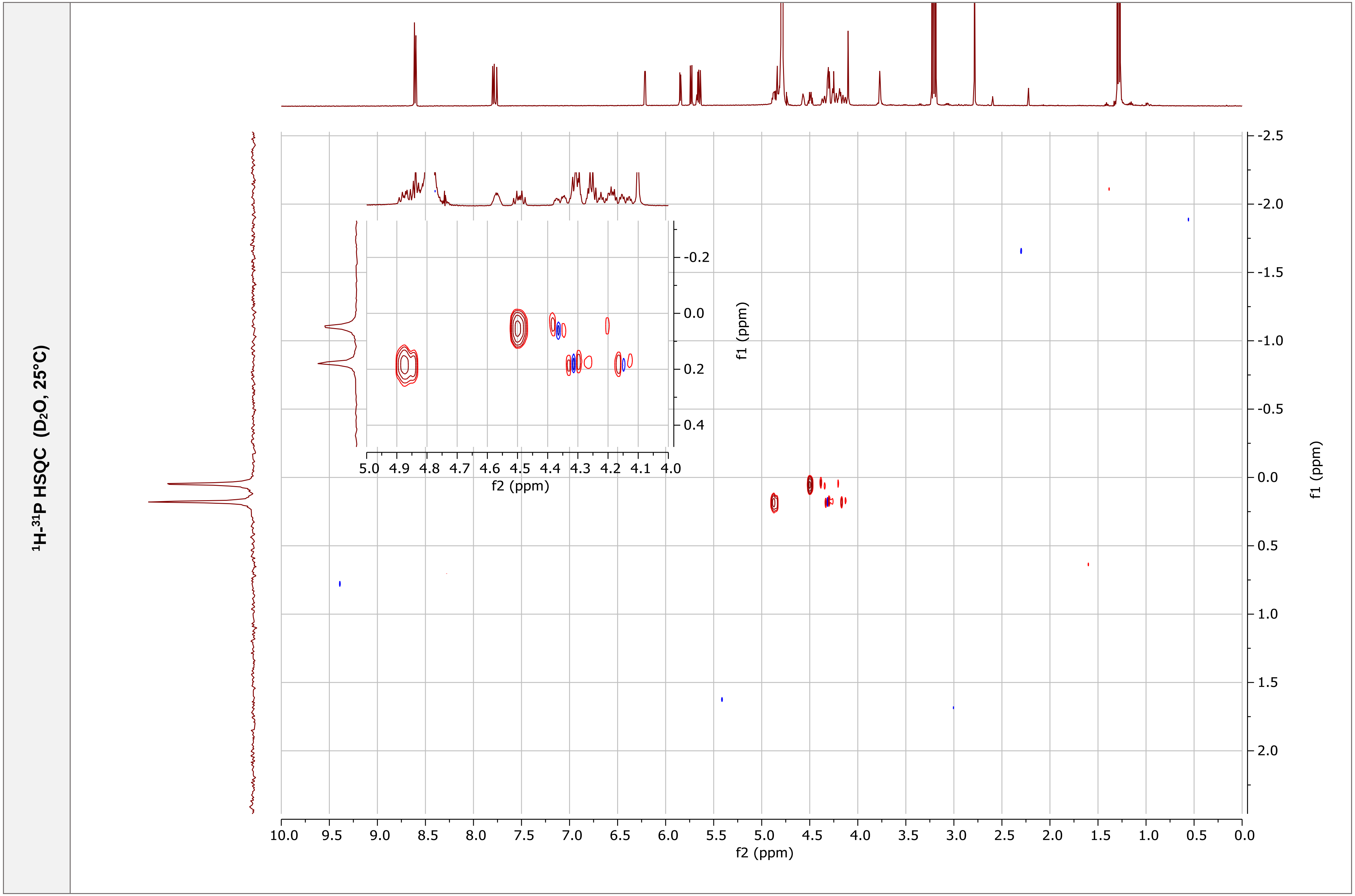

^{1}H-^{31}P HSQC (D$_2$O, 25°C)
f2 (ppm)
f1 (ppm)

(18) U^{g6m6}AU	
**Chemical structure**	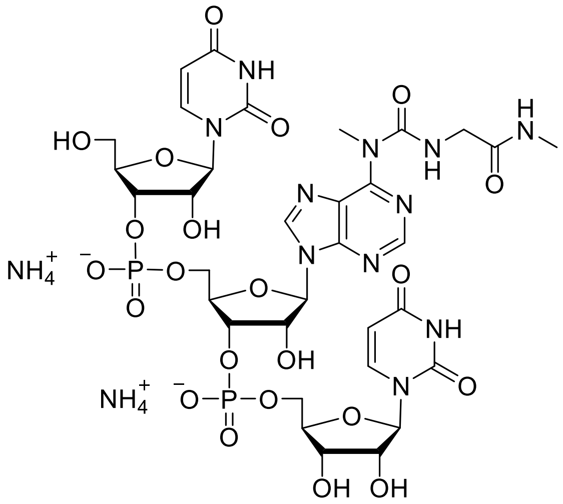
**RP HPLC** Abs. @ 254 nm	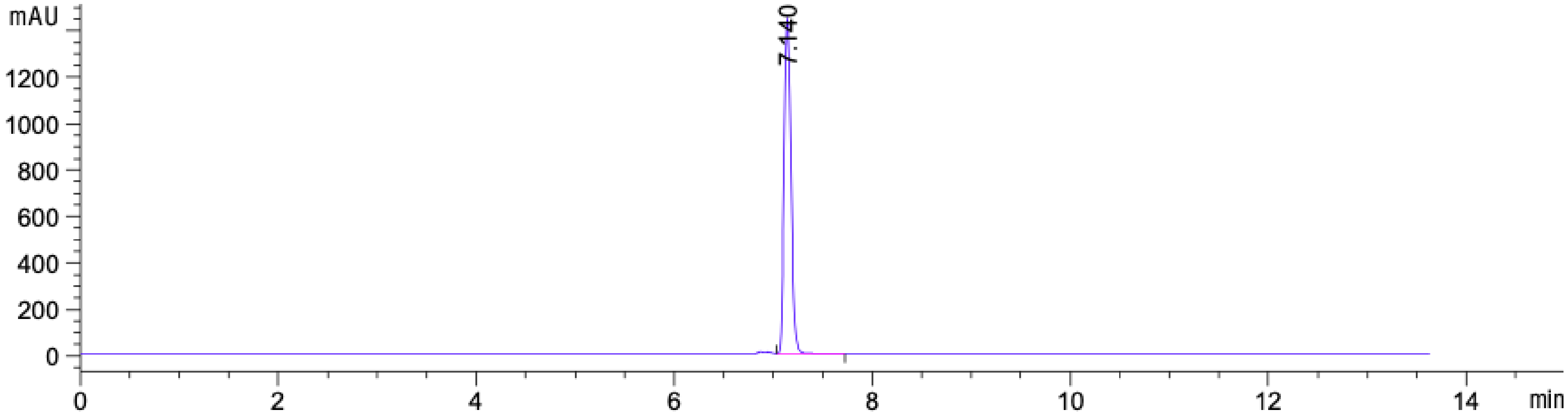

MS (-) ESI

(Calc. $[M-H]^-$ $C_{33}H_{42}N_{11}O_{22}P_2^-$ 1006.19866)

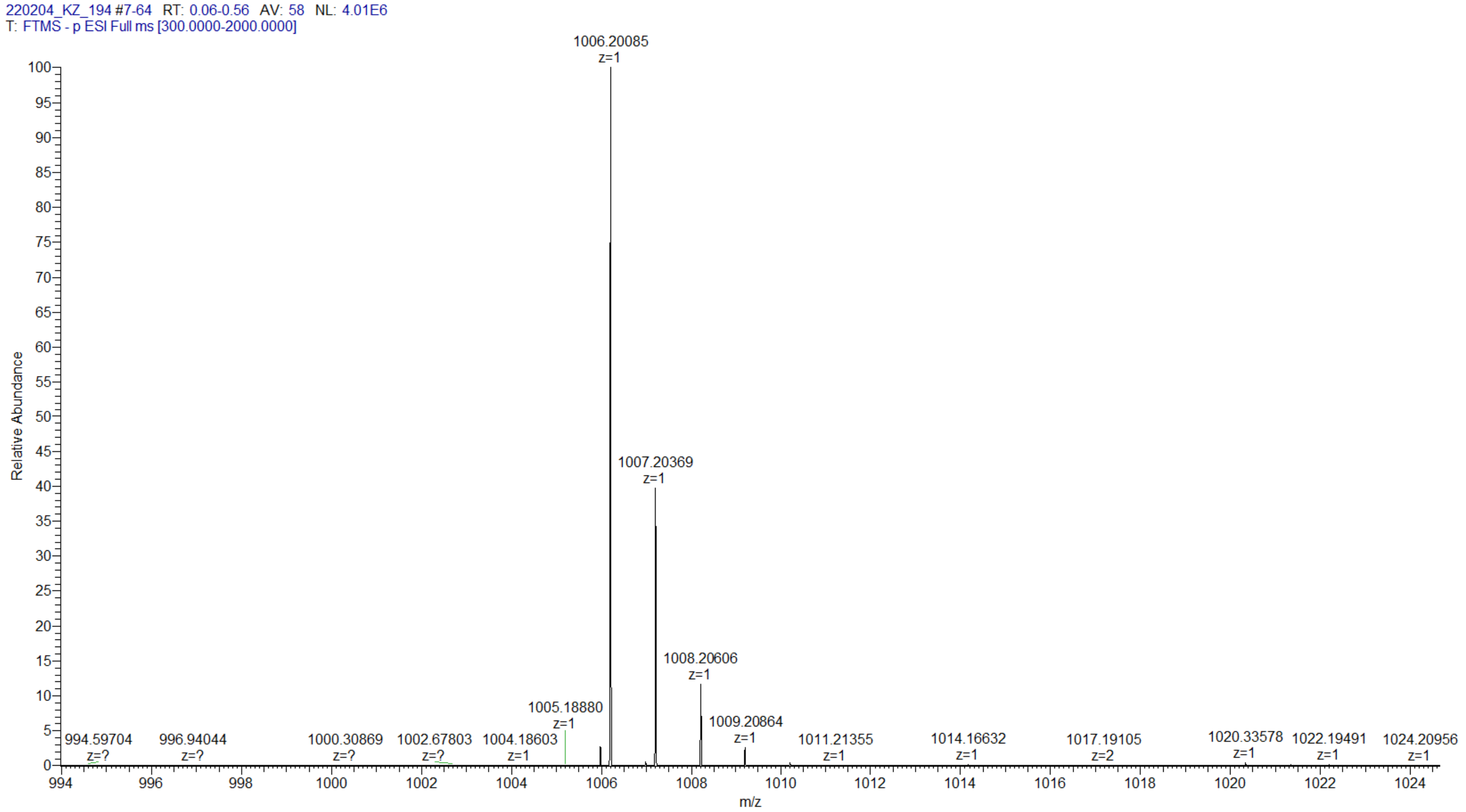

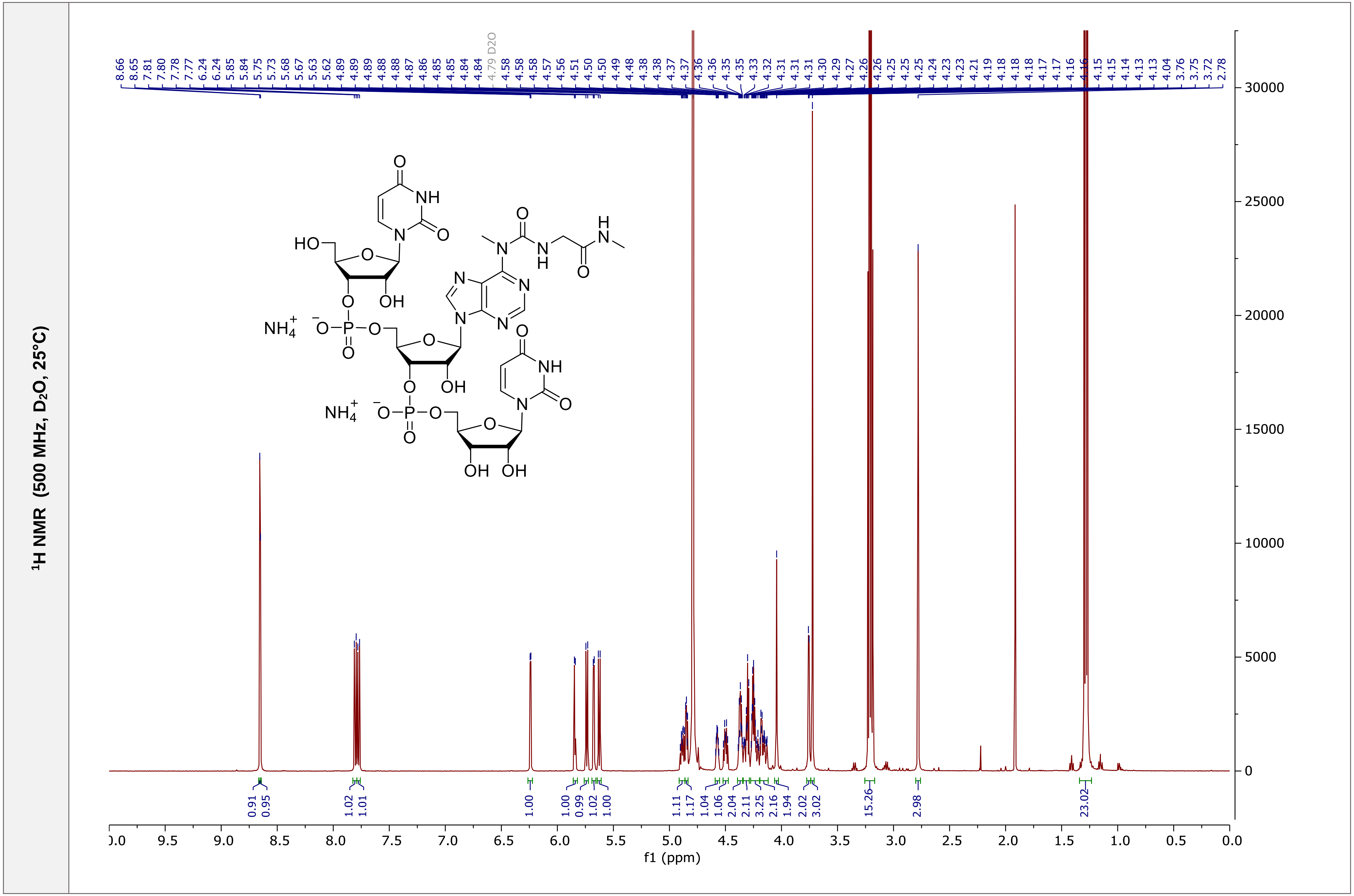

1H NMR (500 MHz, D2O, 25°C)
f1 (ppm)

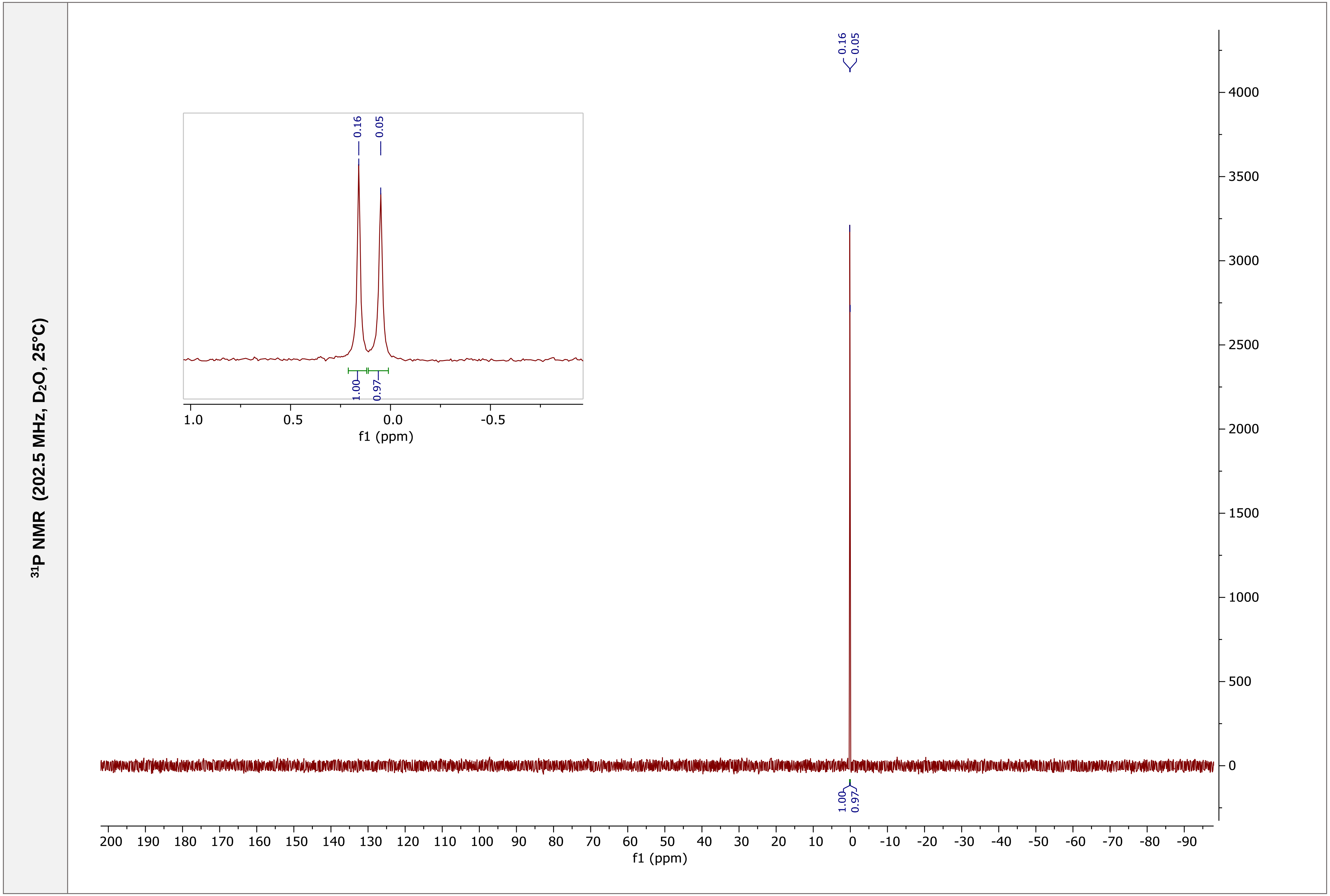

^{31}P NMR (202.5 MHz, D_2O, 25°C)
0.16
0.05
1.00
0.97
f1 (ppm)

COSY NMR (D_2O, 25°C)

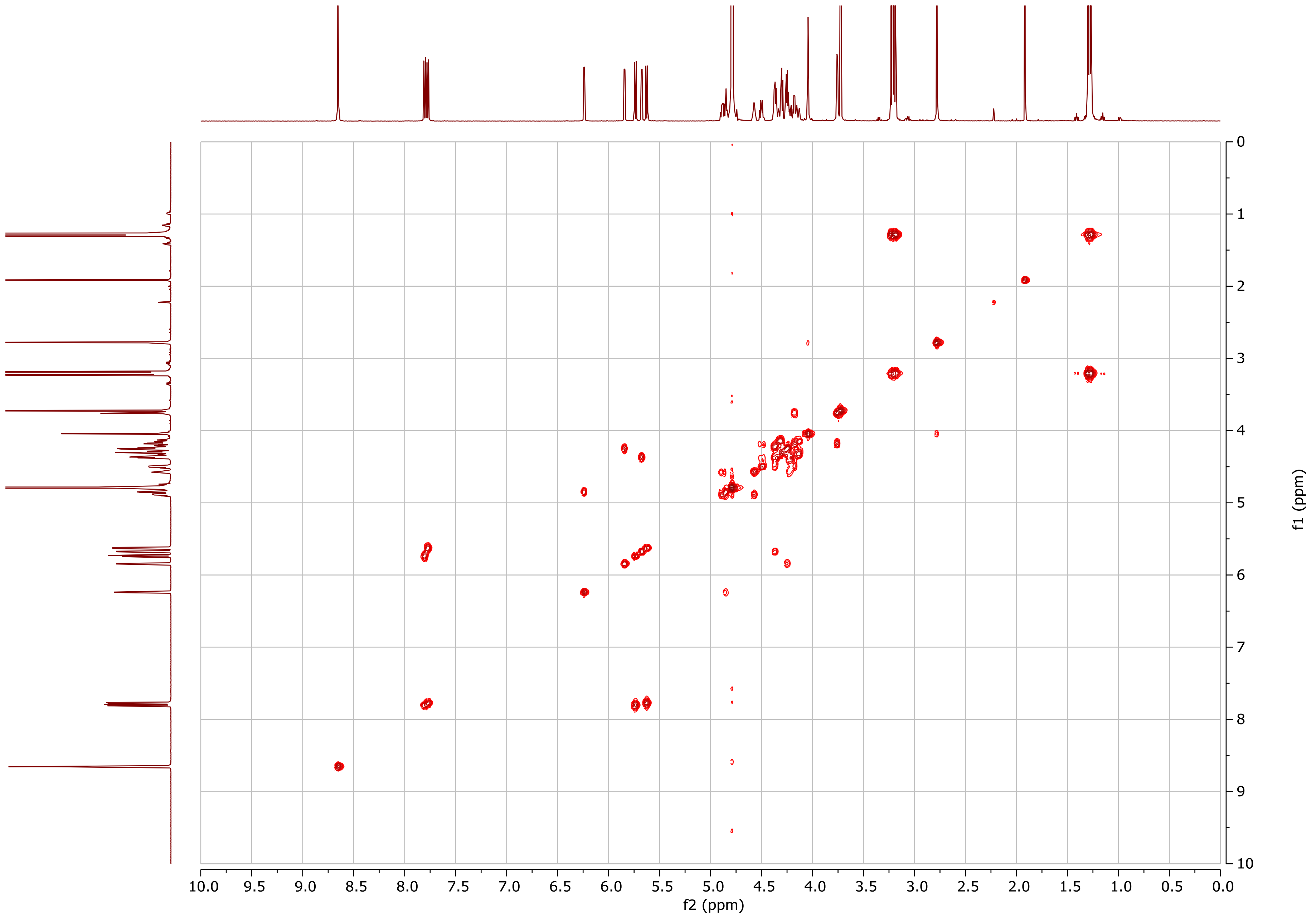

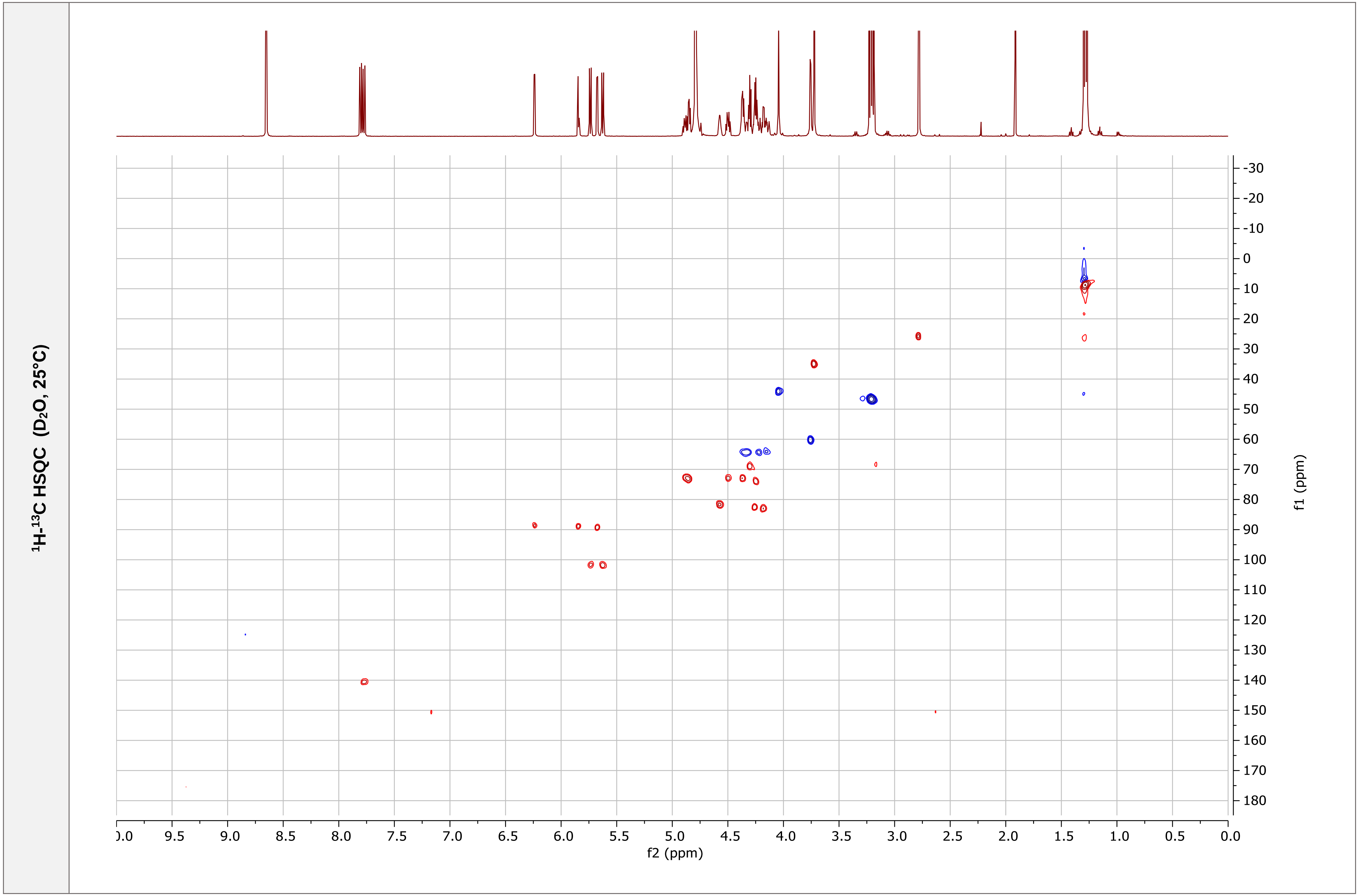

^{1}H-^{13}C HSQC (D$_2$O, 25°C)
f2 (ppm)
f1 (ppm)

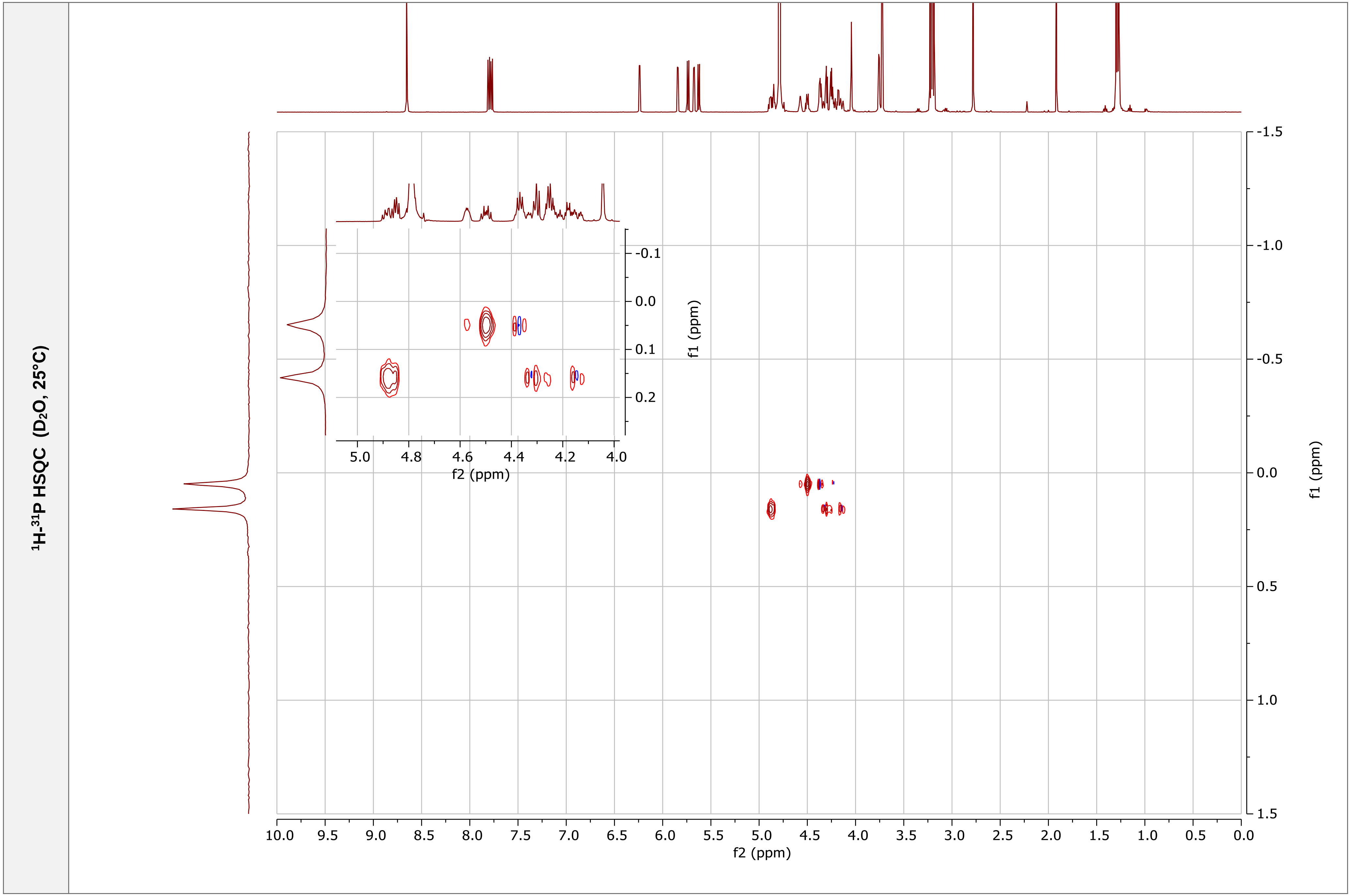

¹H-³¹P HSQC (D₂O, 25°C)
f2 (ppm)
f1 (ppm)
10.0 9.5 9.0 8.5 8.0 7.5 7.0 6.5 6.0 5.5 5.0 4.5 4.0 3.5 3.0 2.5 2.0 1.5 1.0 0.5 0.0
-1.5 -1.0 -0.5 0.0 0.5 1.0 1.5
5.0 4.8 4.6 4.4 4.2 4.0
-0.1 0.0 0.1 0.2

(19) p^{m3}CpG	
**Chemical structure**	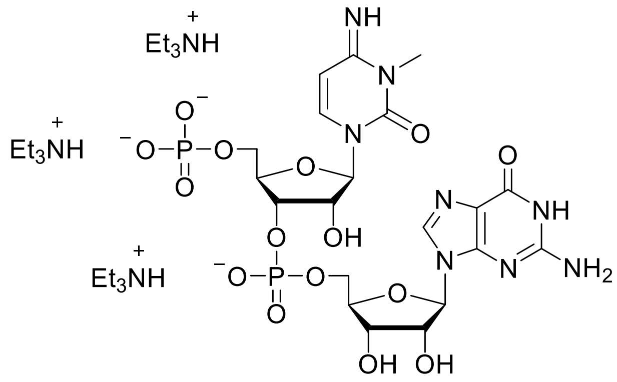
**RP HPLC** Abs. @ 254 nm	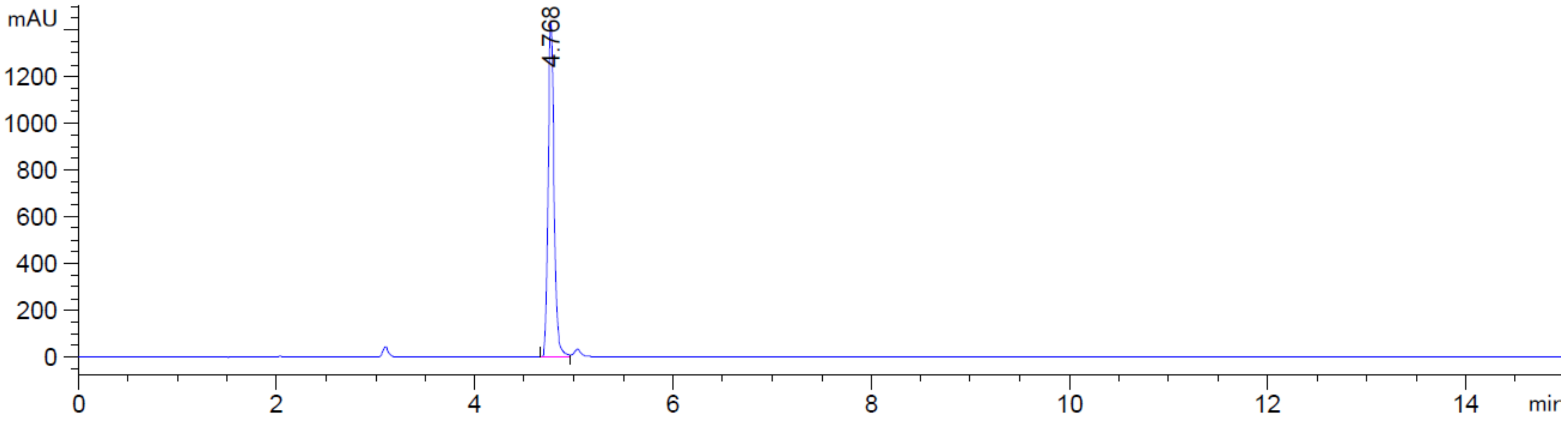

MS (-) ESI
(Calc. $[M-H]^-$ $C_{20}H_{27}N_8O_{15}P_2^-$ 681.10766)

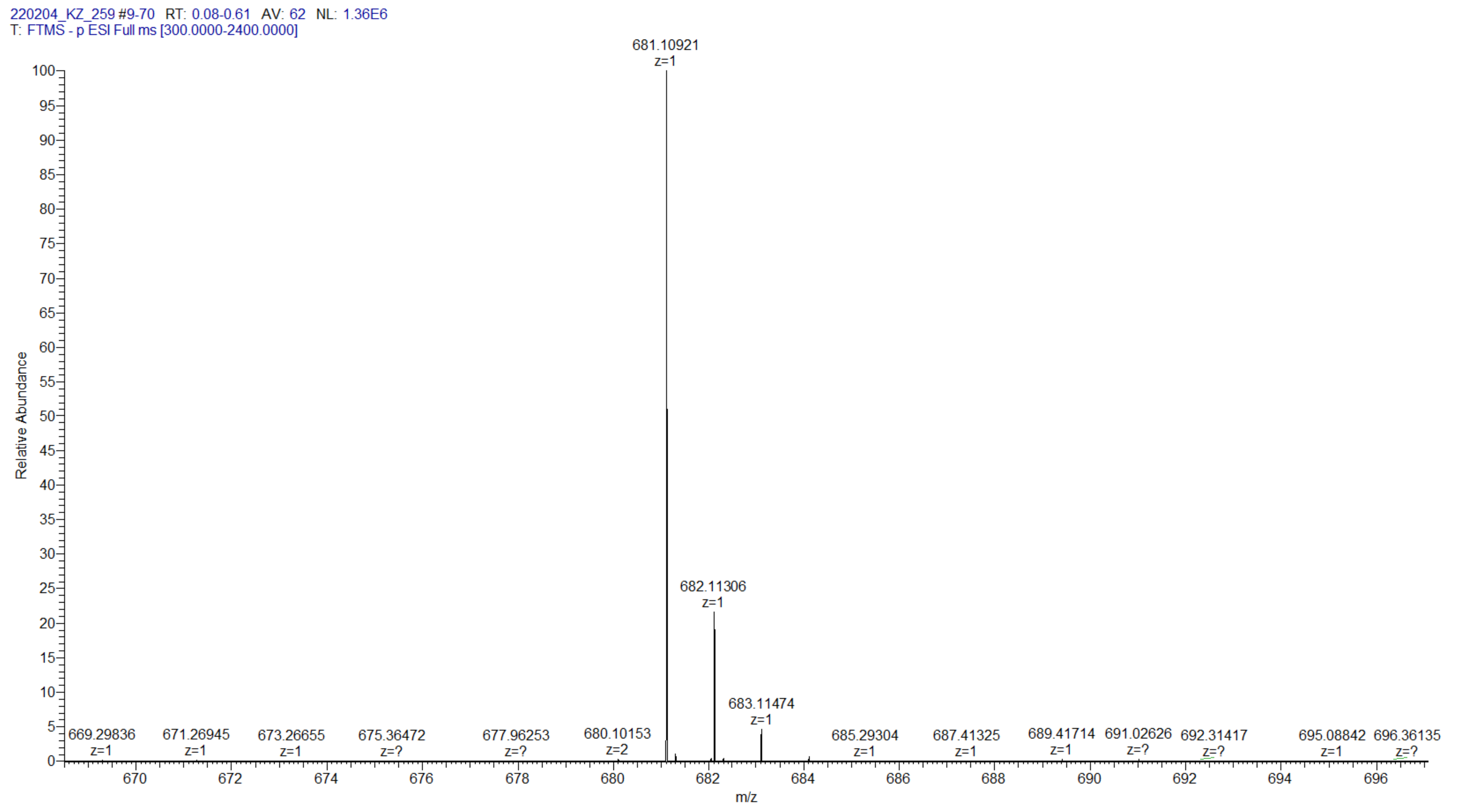

^{1}H NMR (500 MHz, D_2O, 25°C)

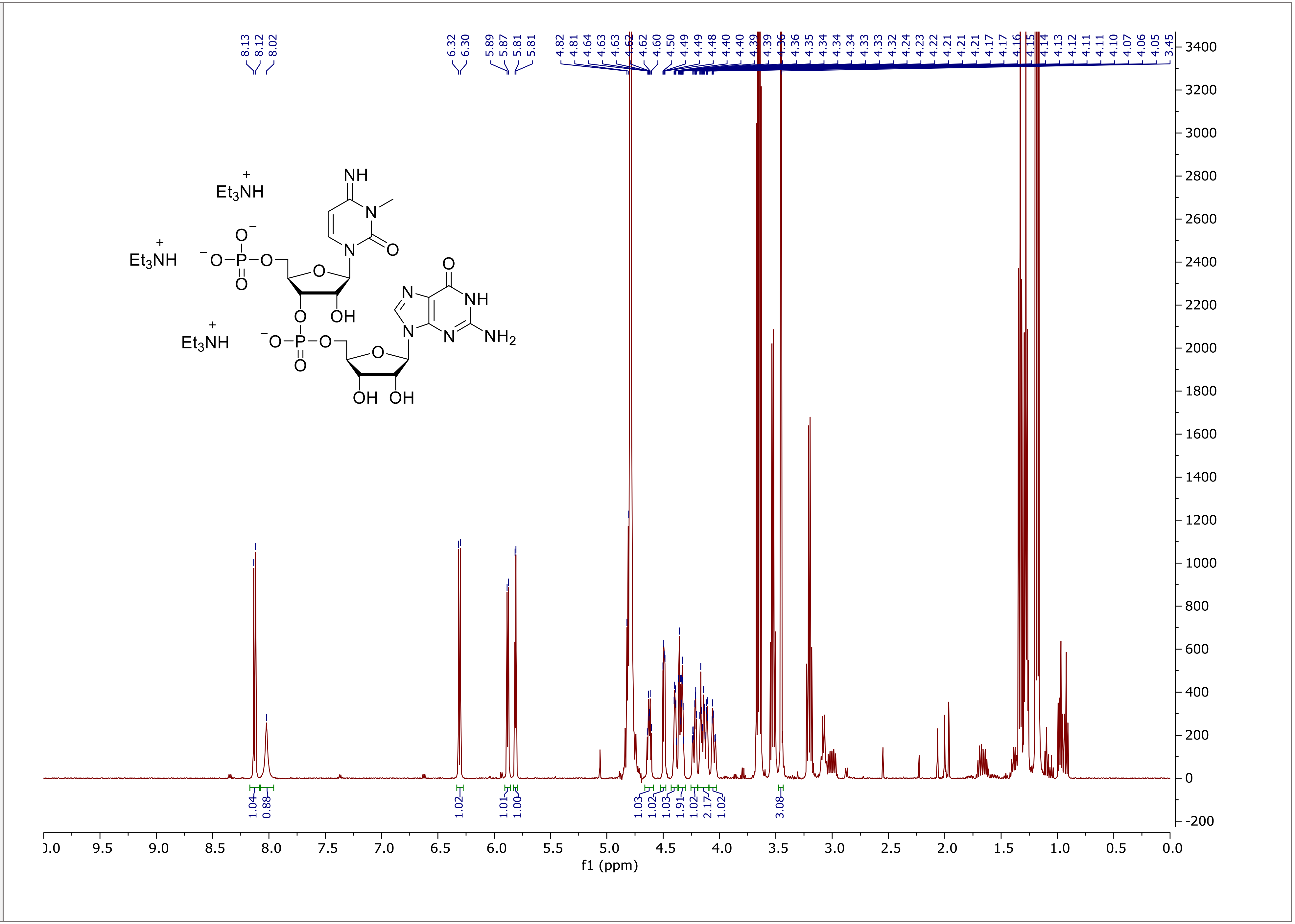

^{31}P NMR (202.5 MHz, D_2O, 25°C)

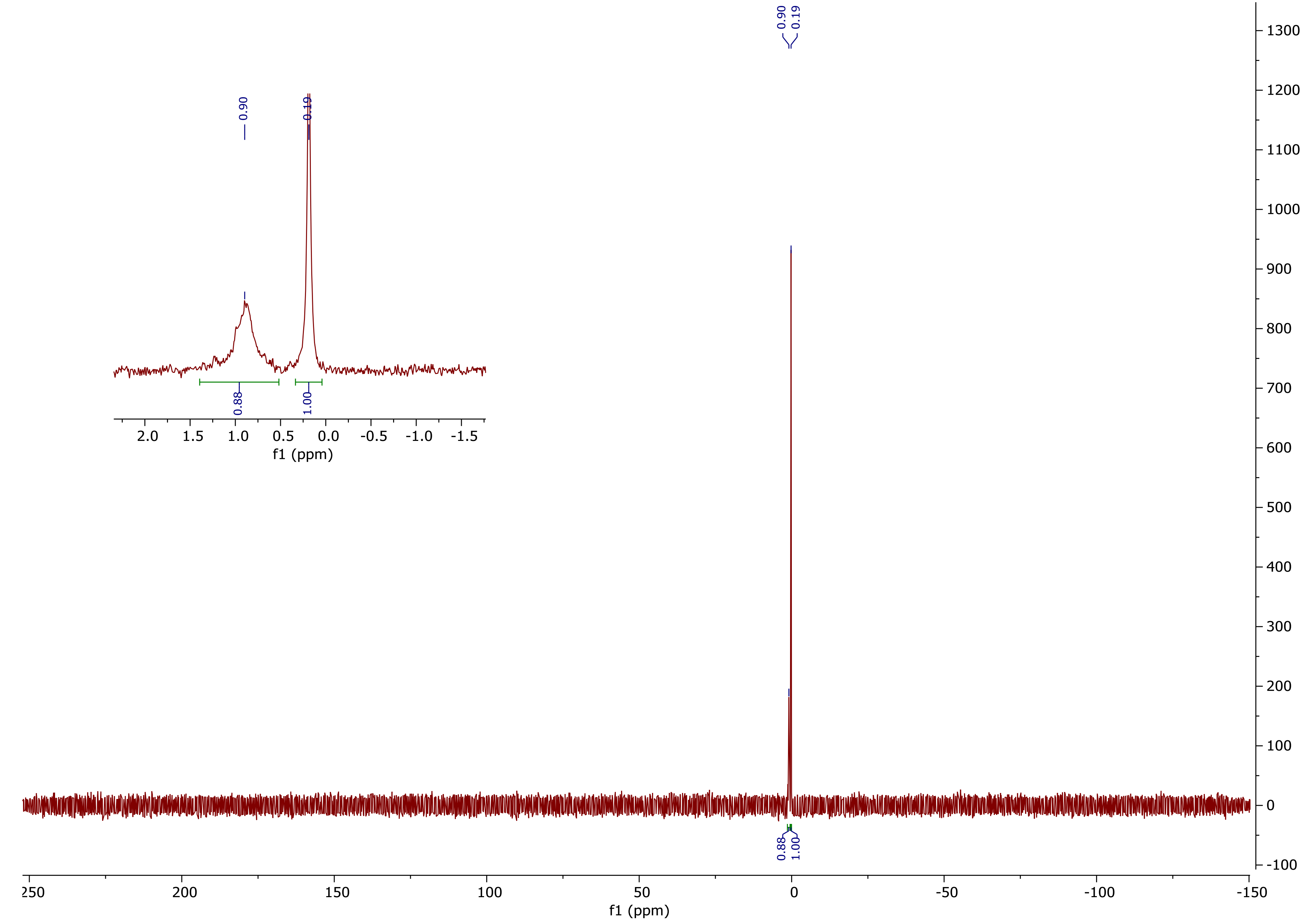

COSY NMR (D$_2$O, 25°C)

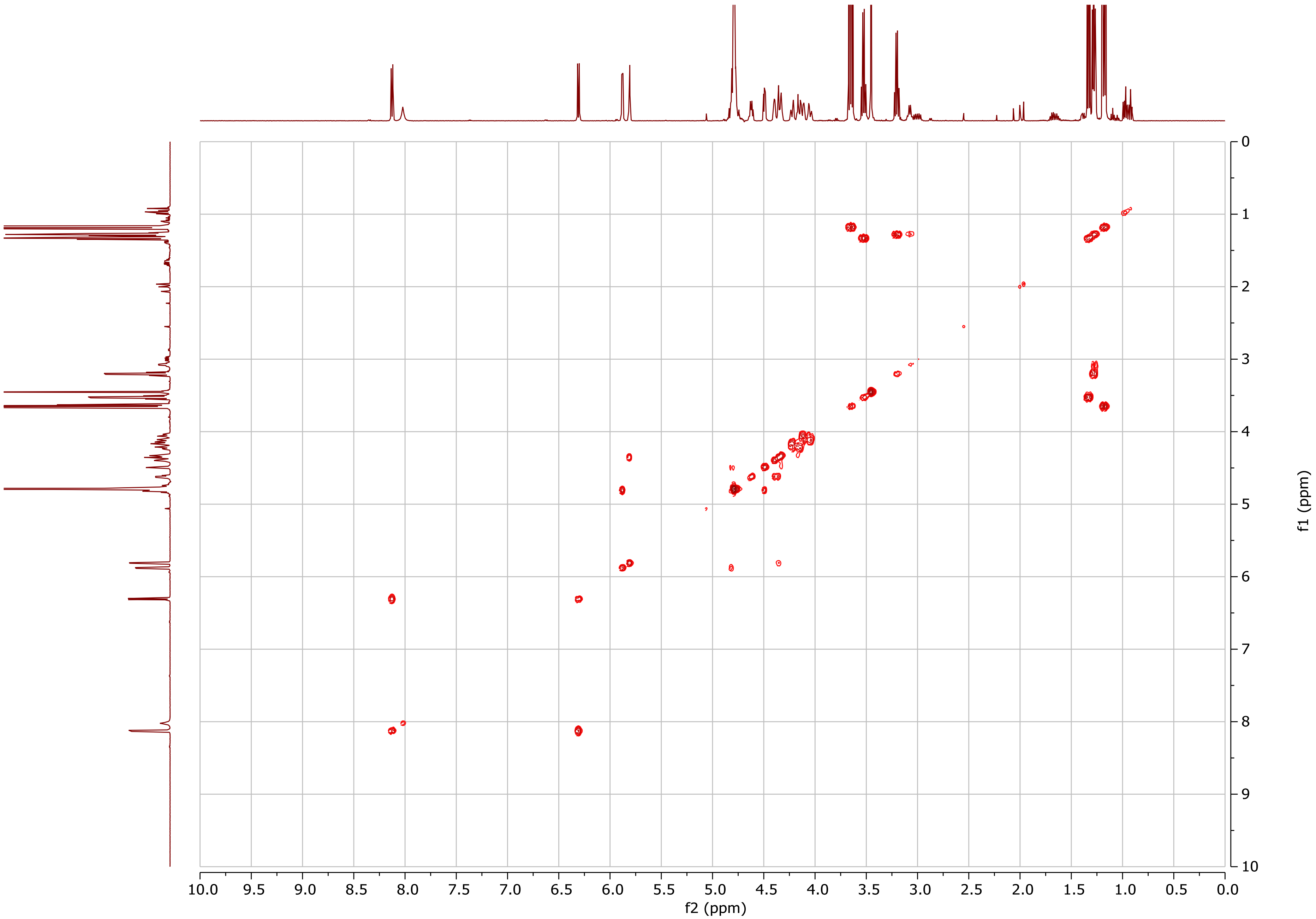

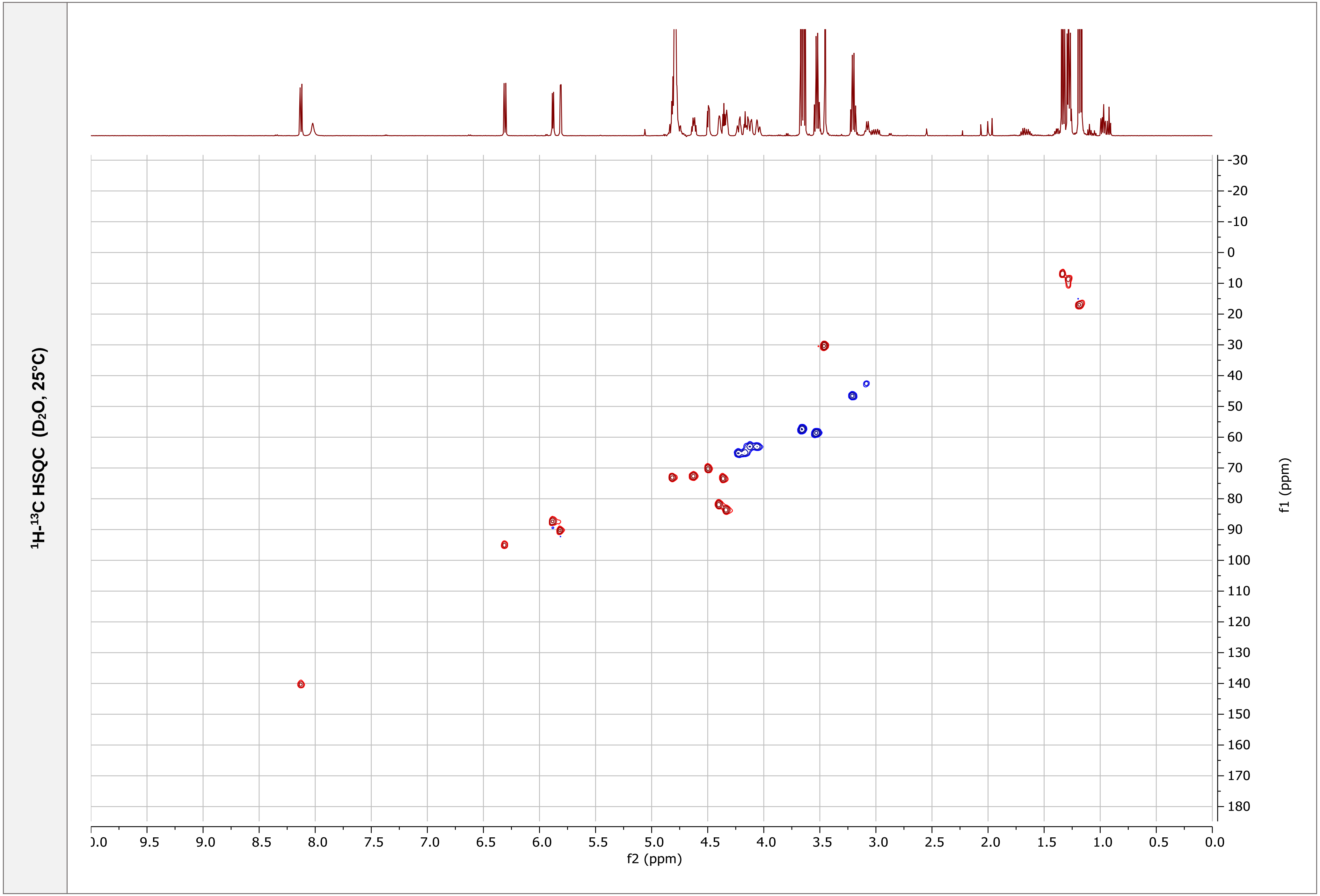

^{1}H-^{13}C HSQC (D$_2$O, 25°C)
f2 (ppm)
f1 (ppm)

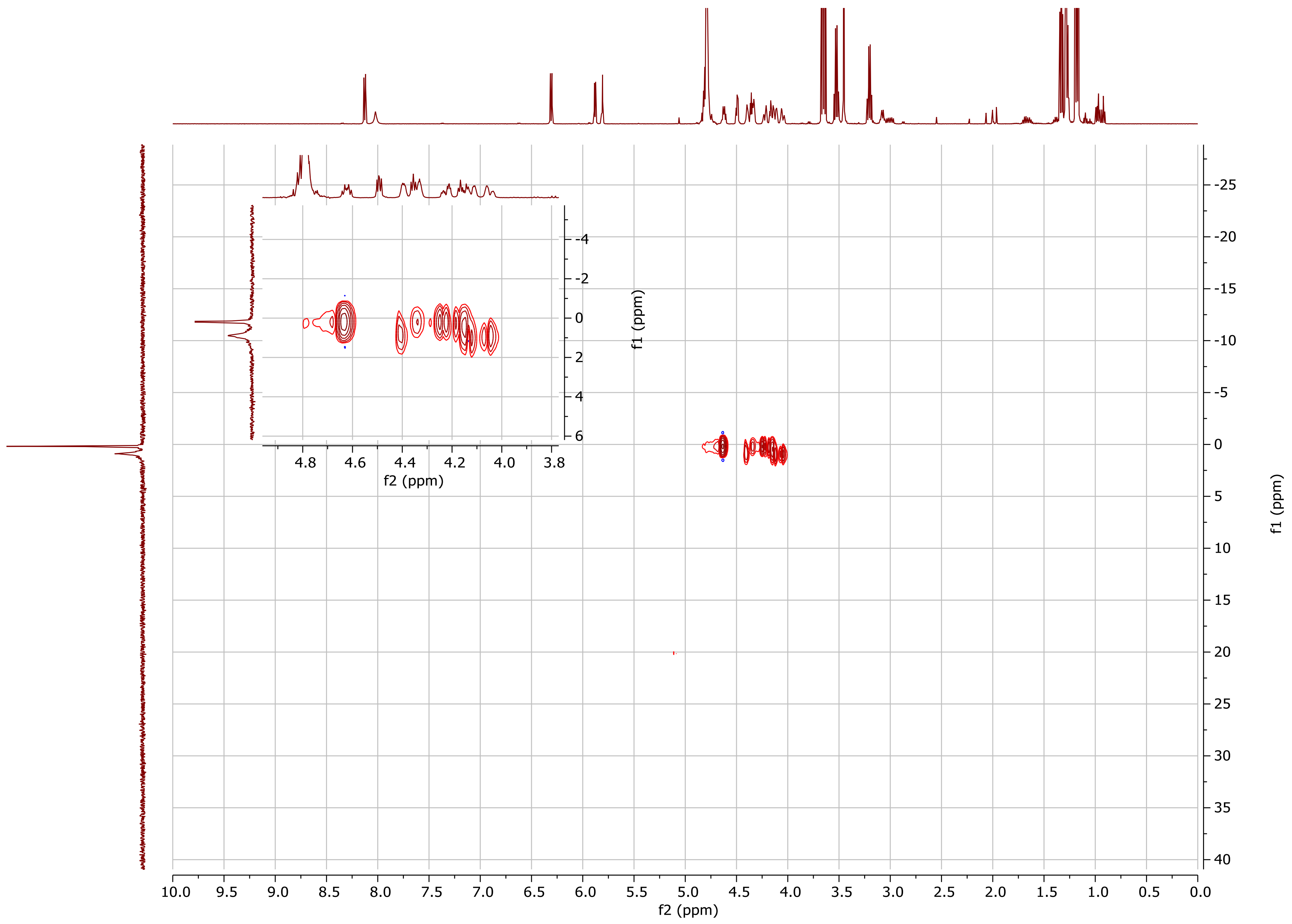

^{1}H-^{31}P HSQC (D_2O, 25°C)

(20) p^{m4}CpG

**Chemical structure**	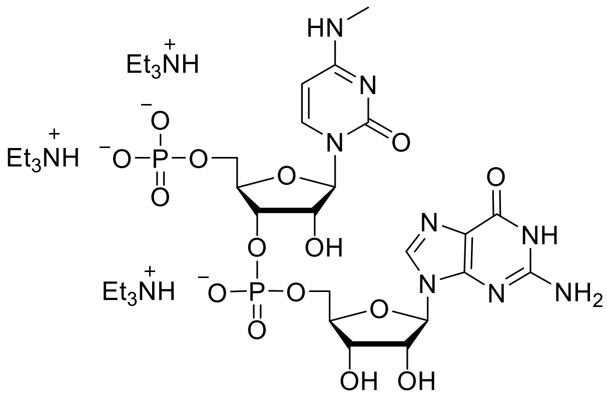
**RP HPLC** Abs. @ 254 nm	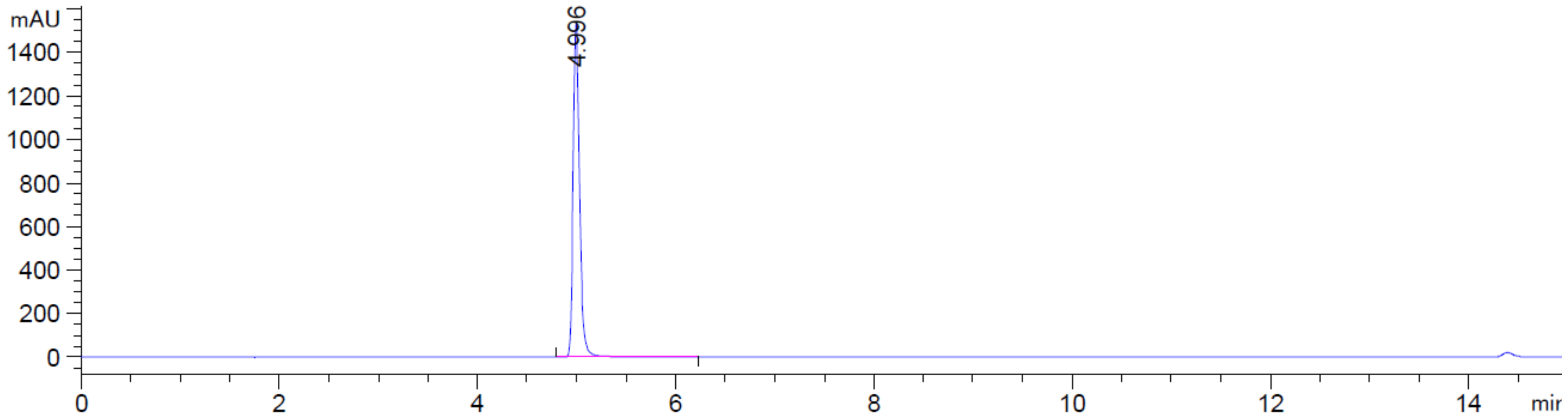

MS (-) ESI

(Calc. [M-H]$^-$ $C_{20}H_{27}N_8O_{15}P_2^-$ 681.10766)

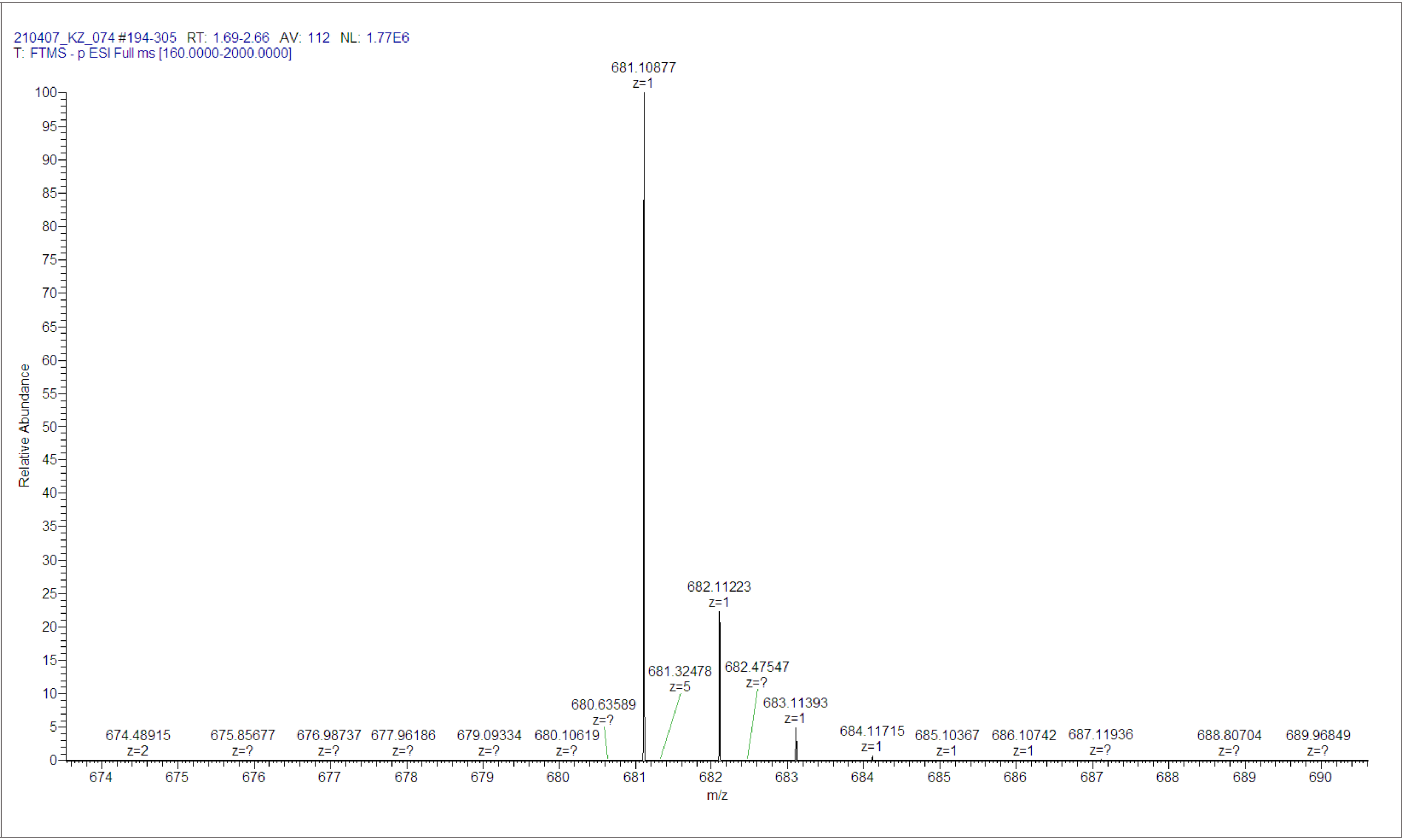

^1H NMR (500 MHz, D_2O, 25°C)

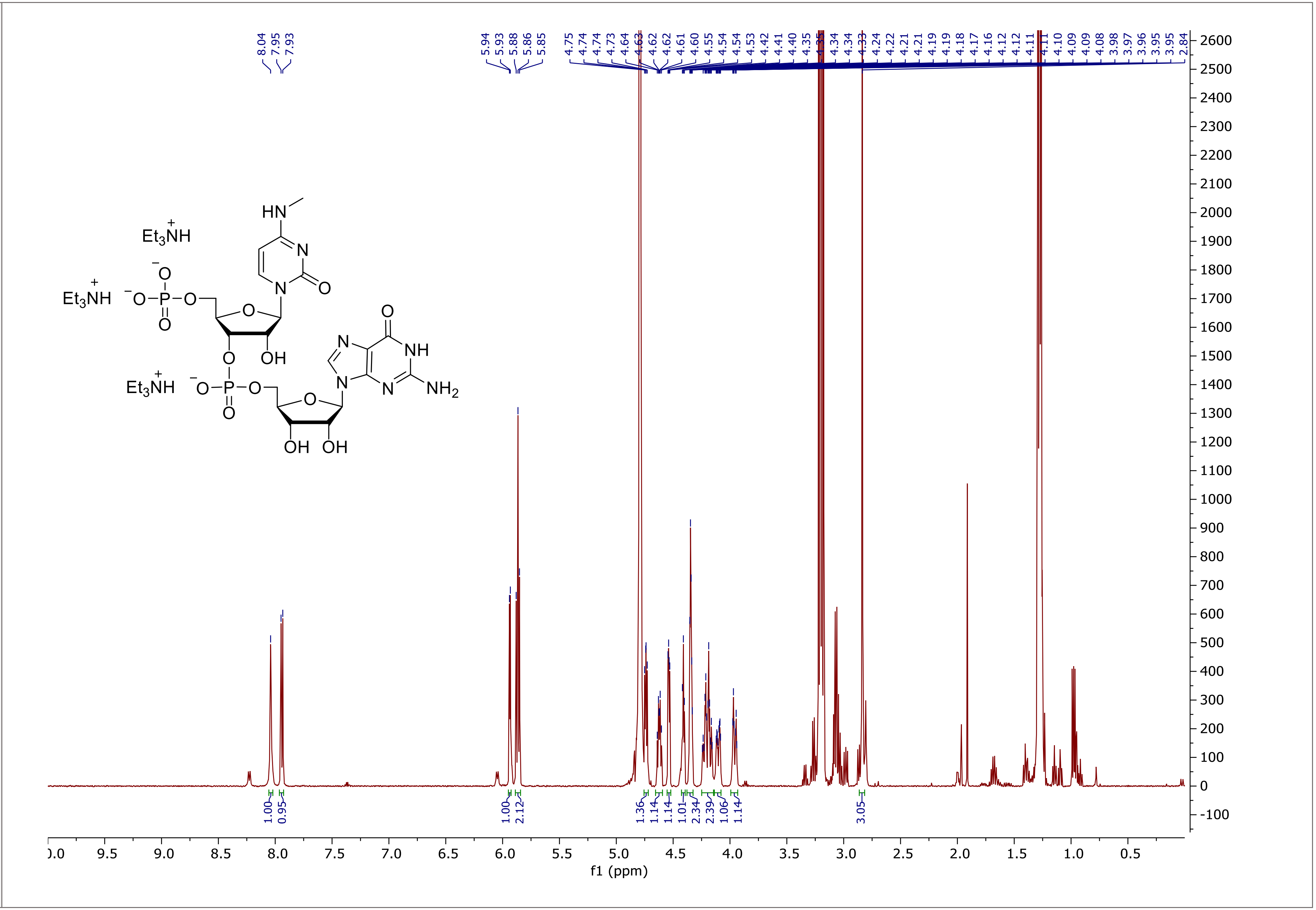

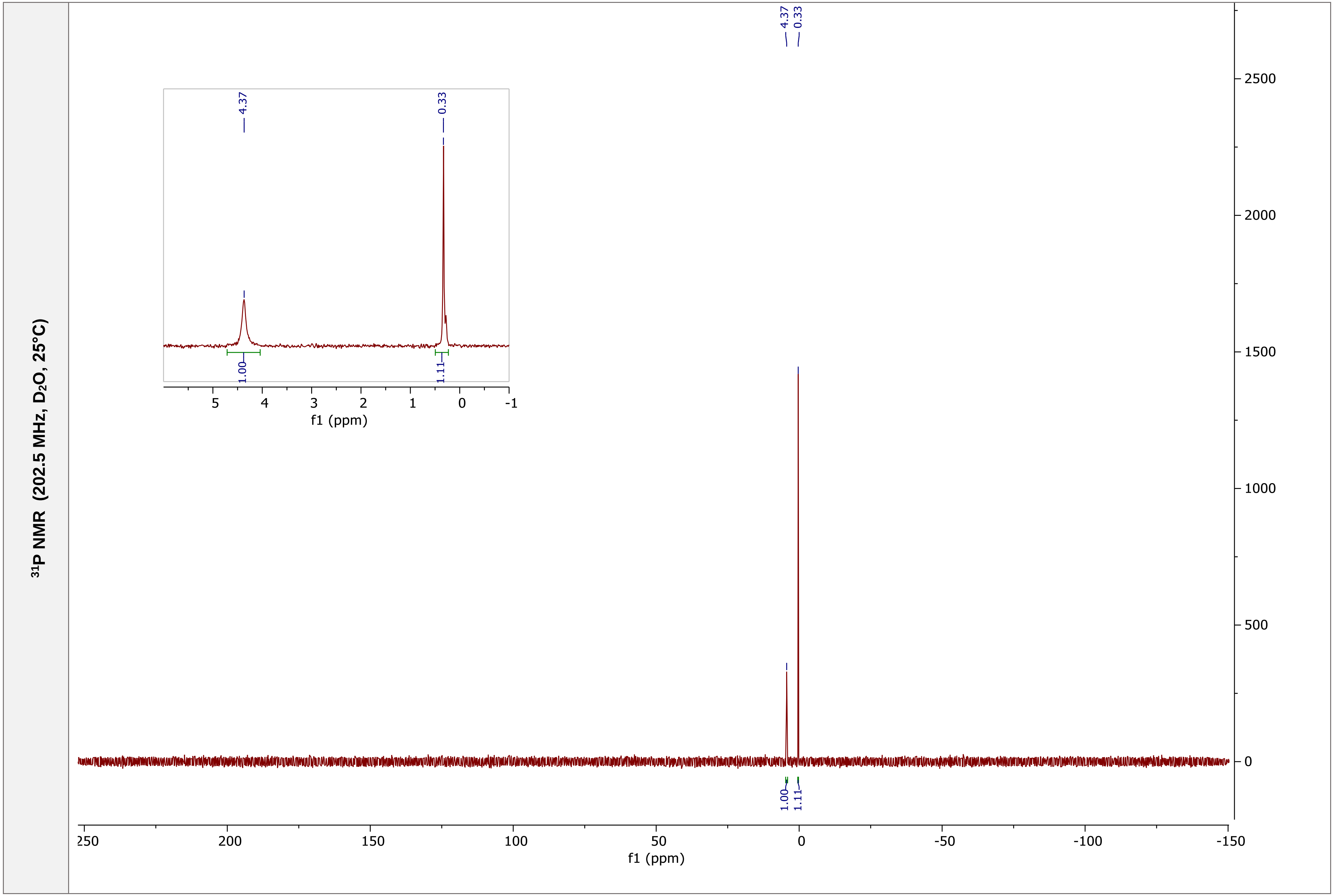

^{31}P NMR (202.5 MHz, D$_2$O, 25°C)
4.37
0.33
1.00
1.11
f1 (ppm)

COSY NMR (D$_2$O, 25°C)
f2 (ppm)
f1 (ppm)
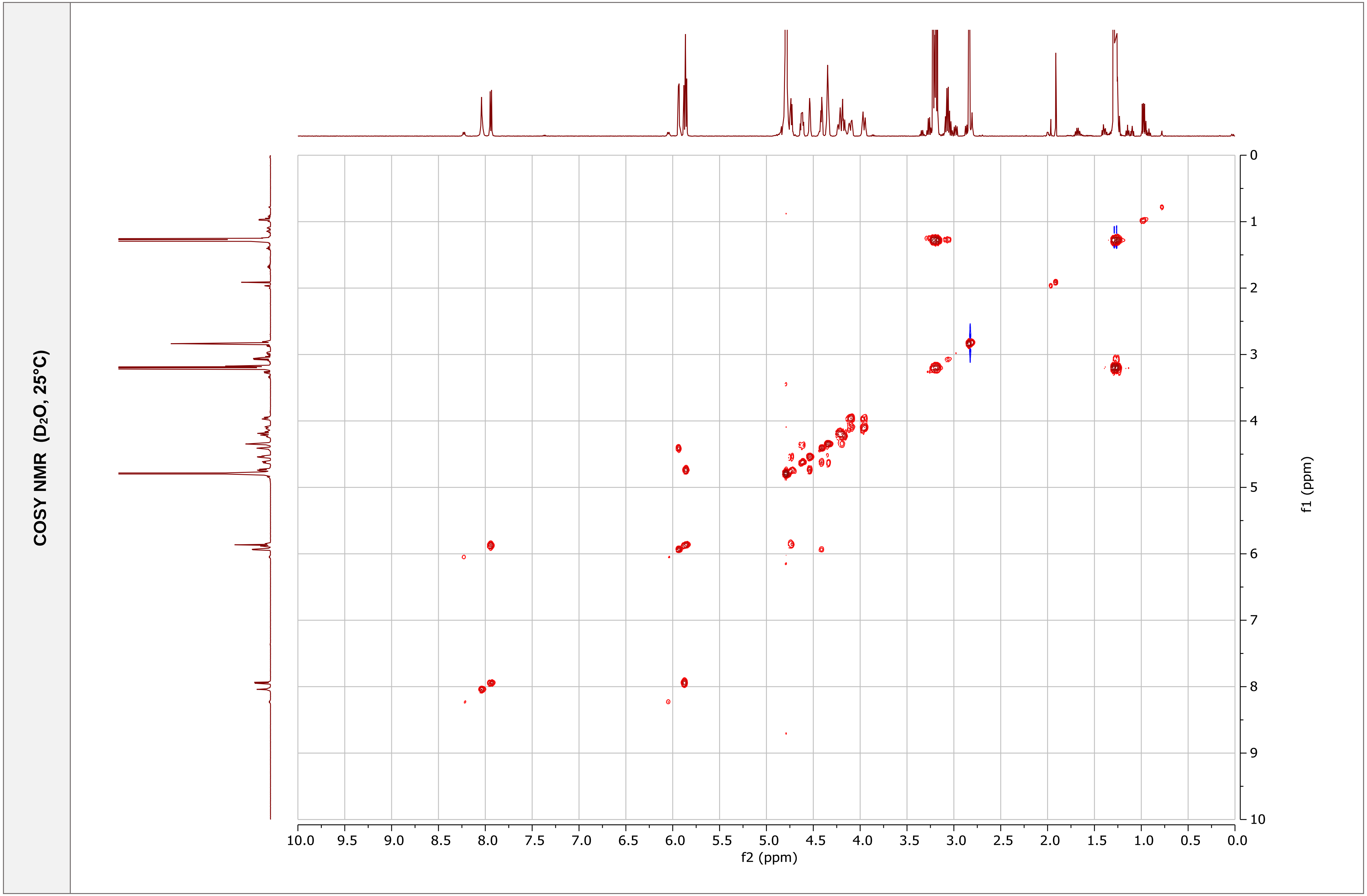

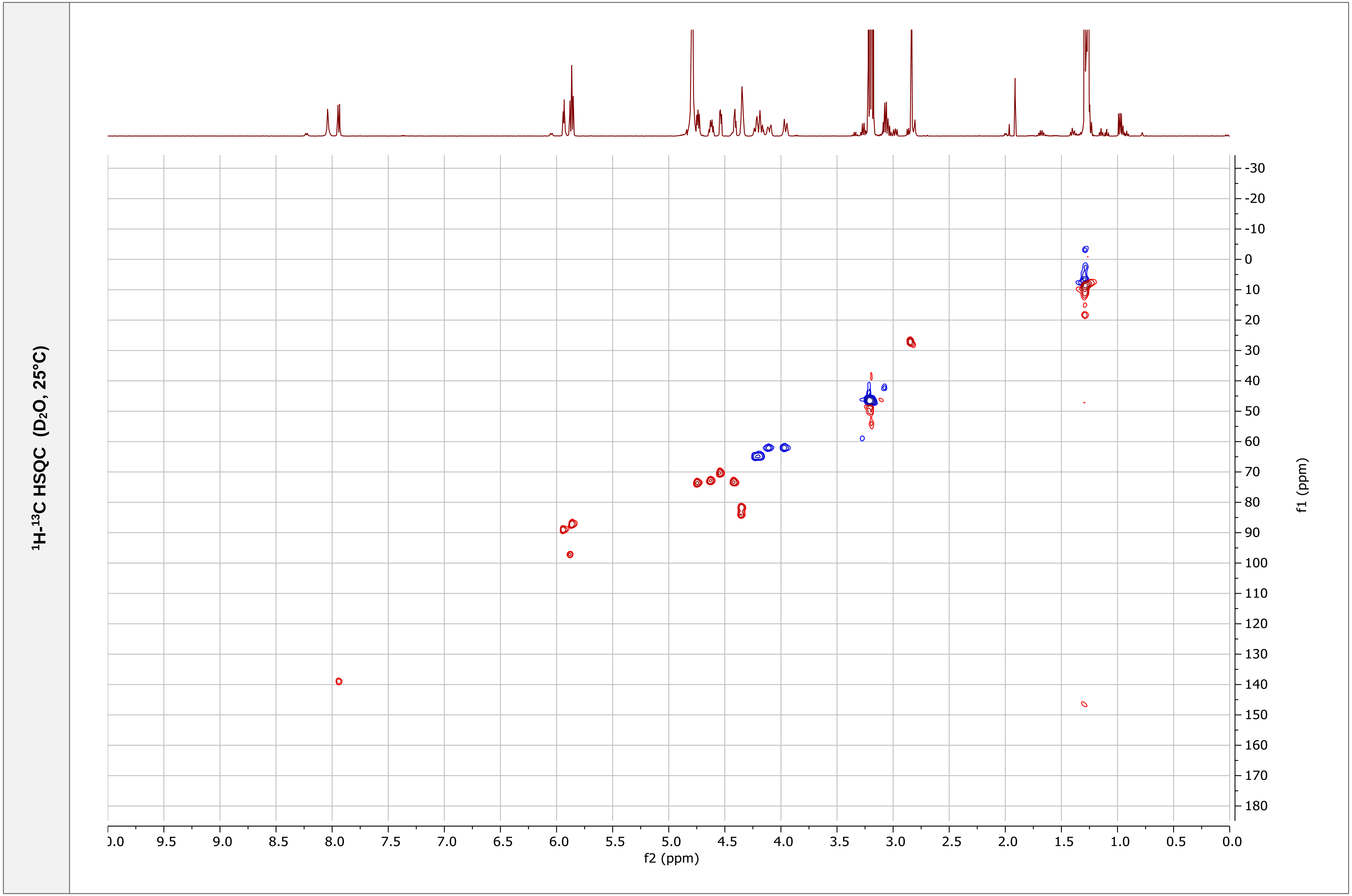

^1H-^{13}C HSQC (D$_2$O, 25°C)
f2 (ppm)
f1 (ppm)

^{1}H-^{31}P HSQC (D_2O, 25°C)

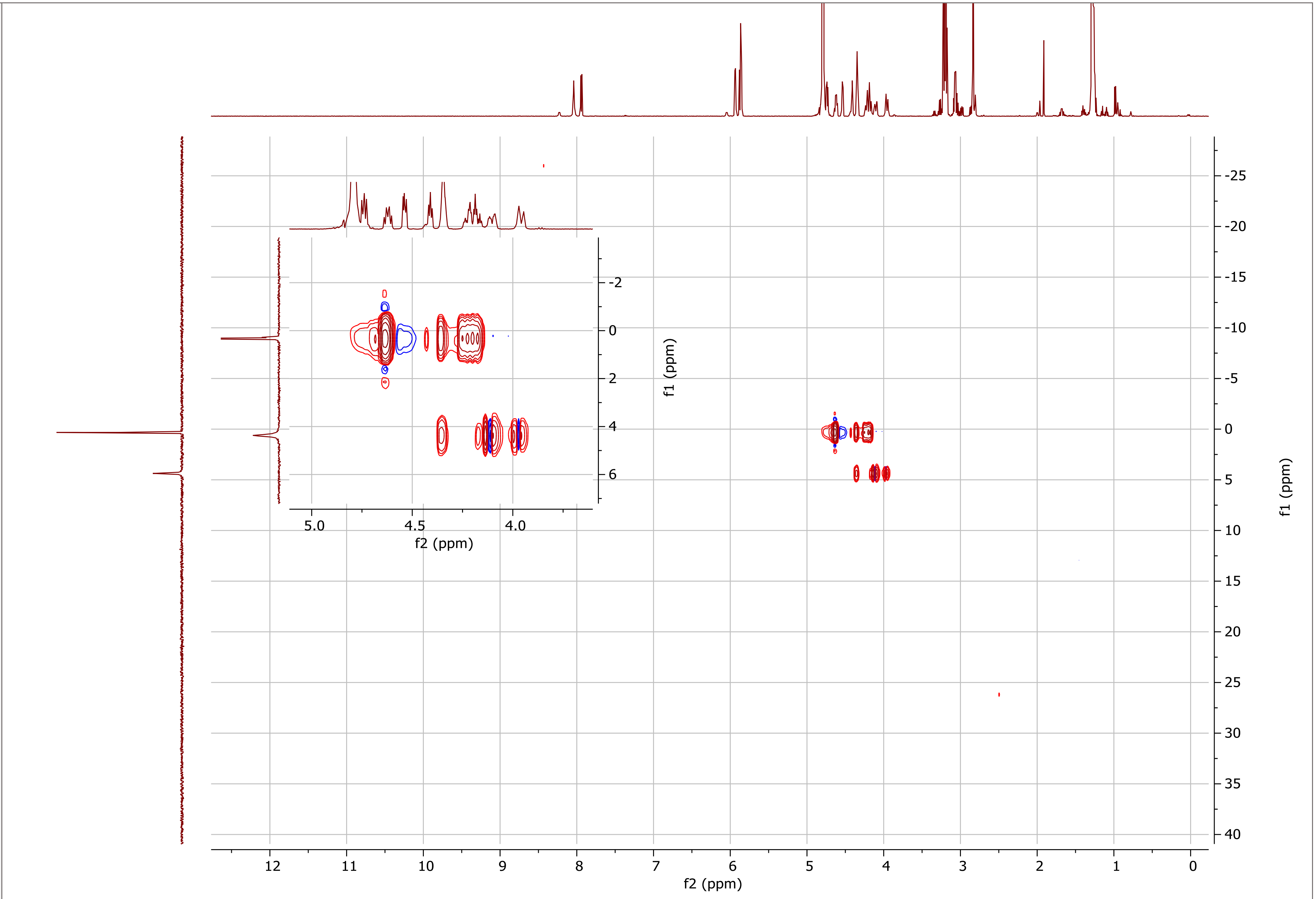

(21) p^{m3,4}CpG	
**Chemical structure**	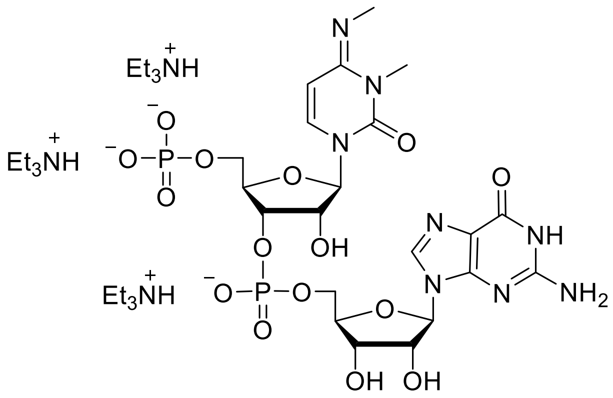
**RP HPLC** Abs. @ 254 nm	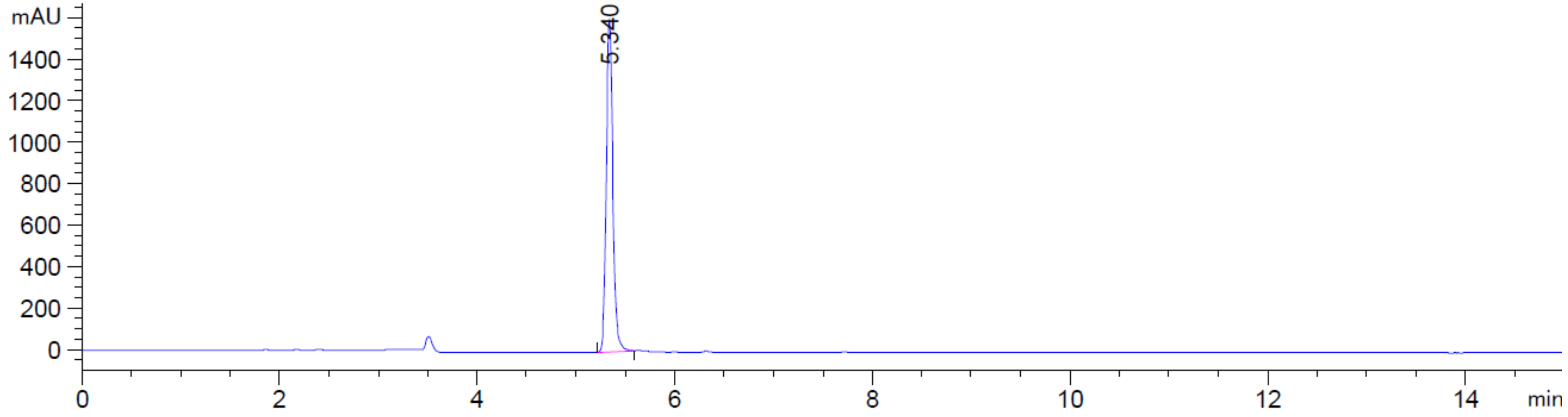

MS (-) ESI
(Calc. $[M-H]^- C_{21}H_{29}N_8O_{15}P_2^-$ 695.12331)

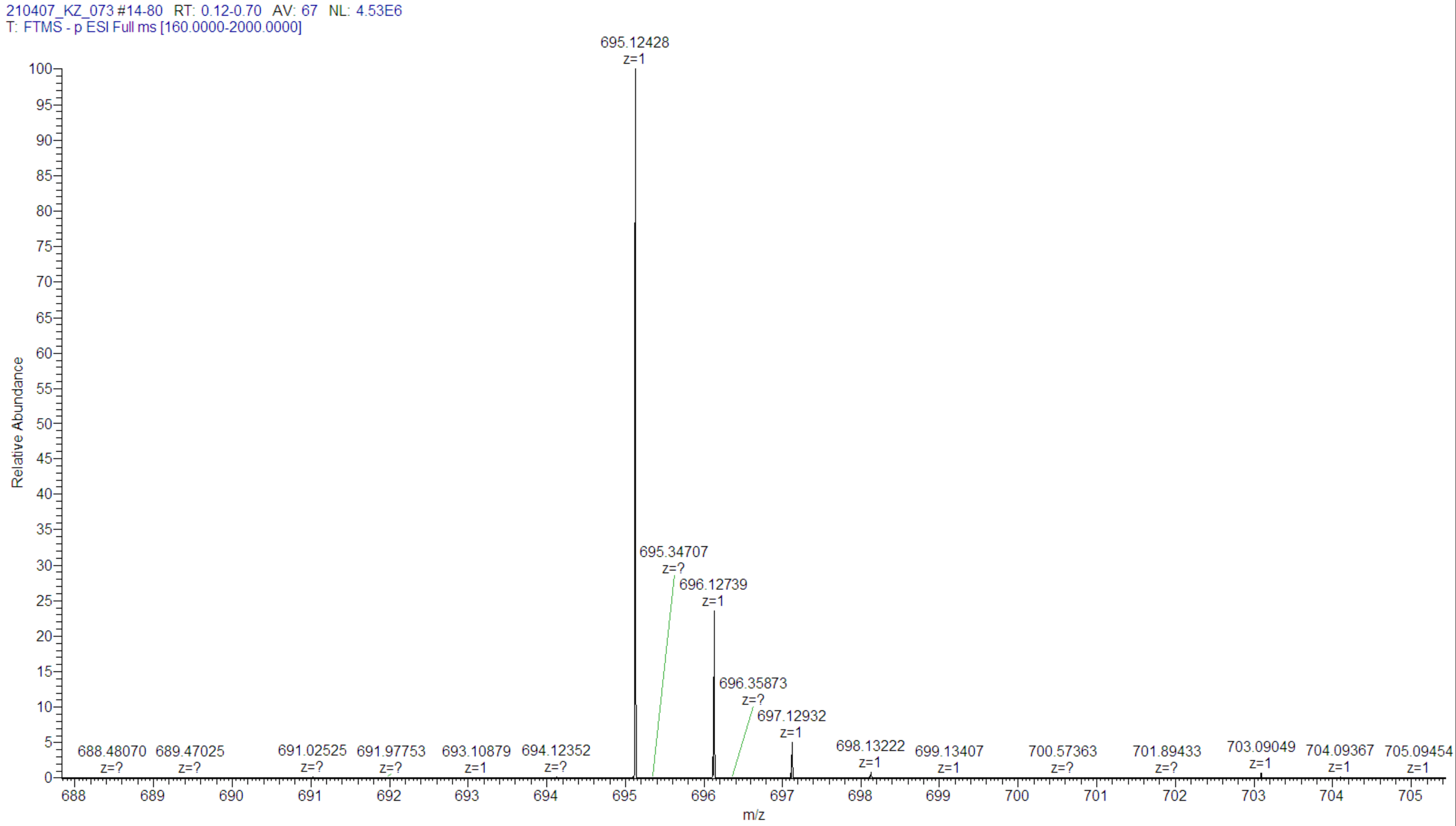

^{1}H NMR (500 MHz, D_2O, 25°C)

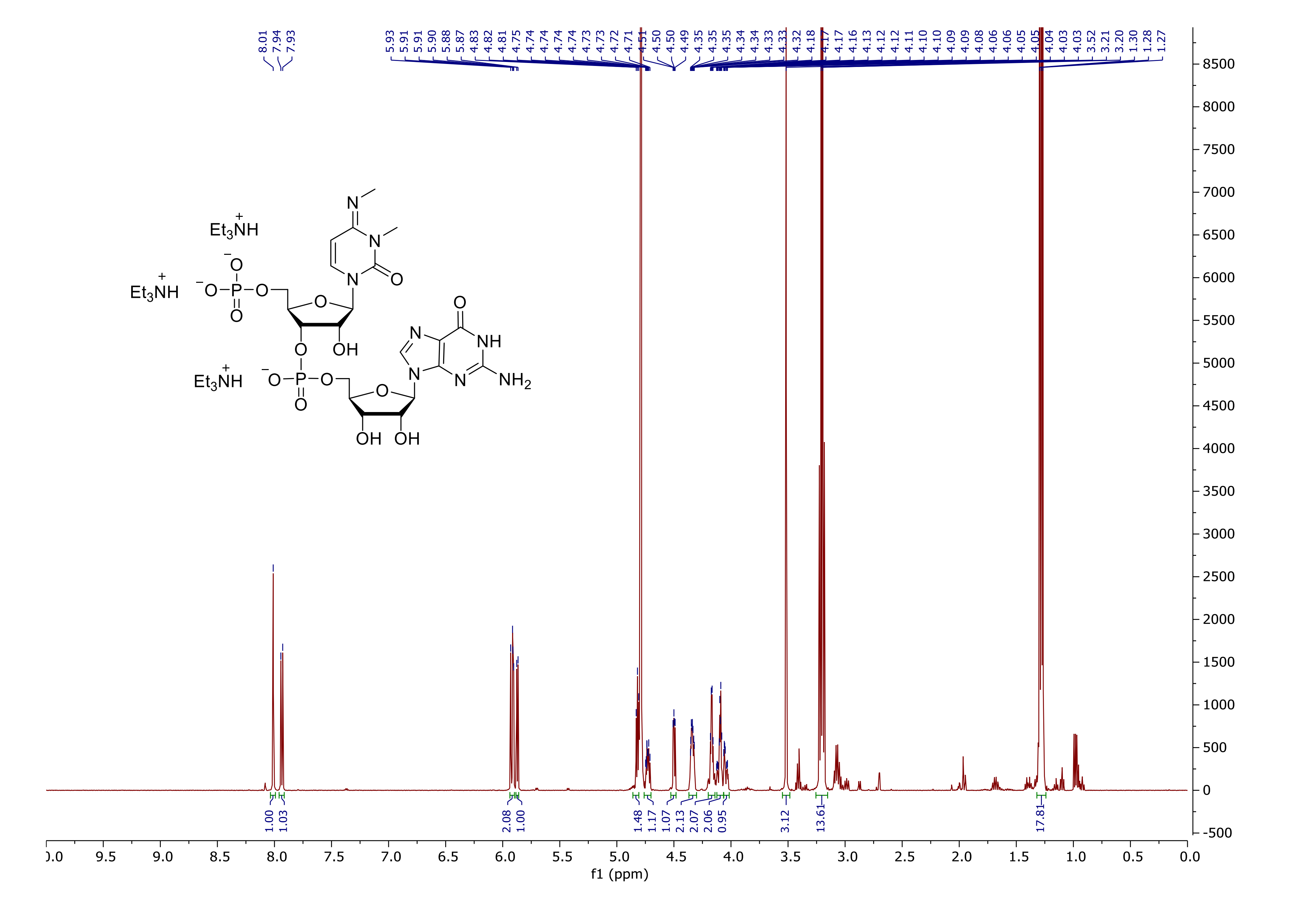

^{31}P NMR (202.5 MHz, D_2O, 25°C)

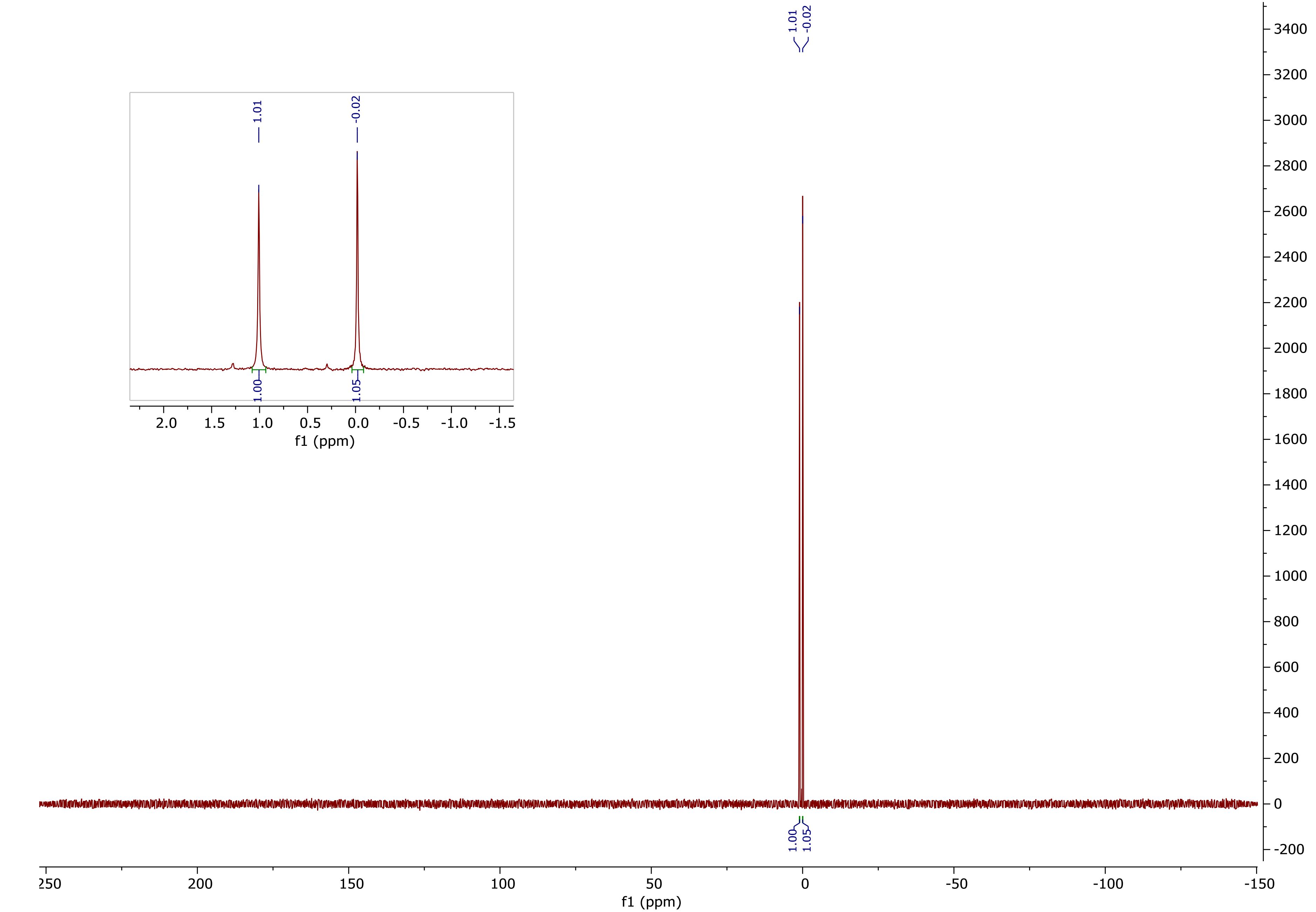

COSY NMR (D_2O, 25°C)

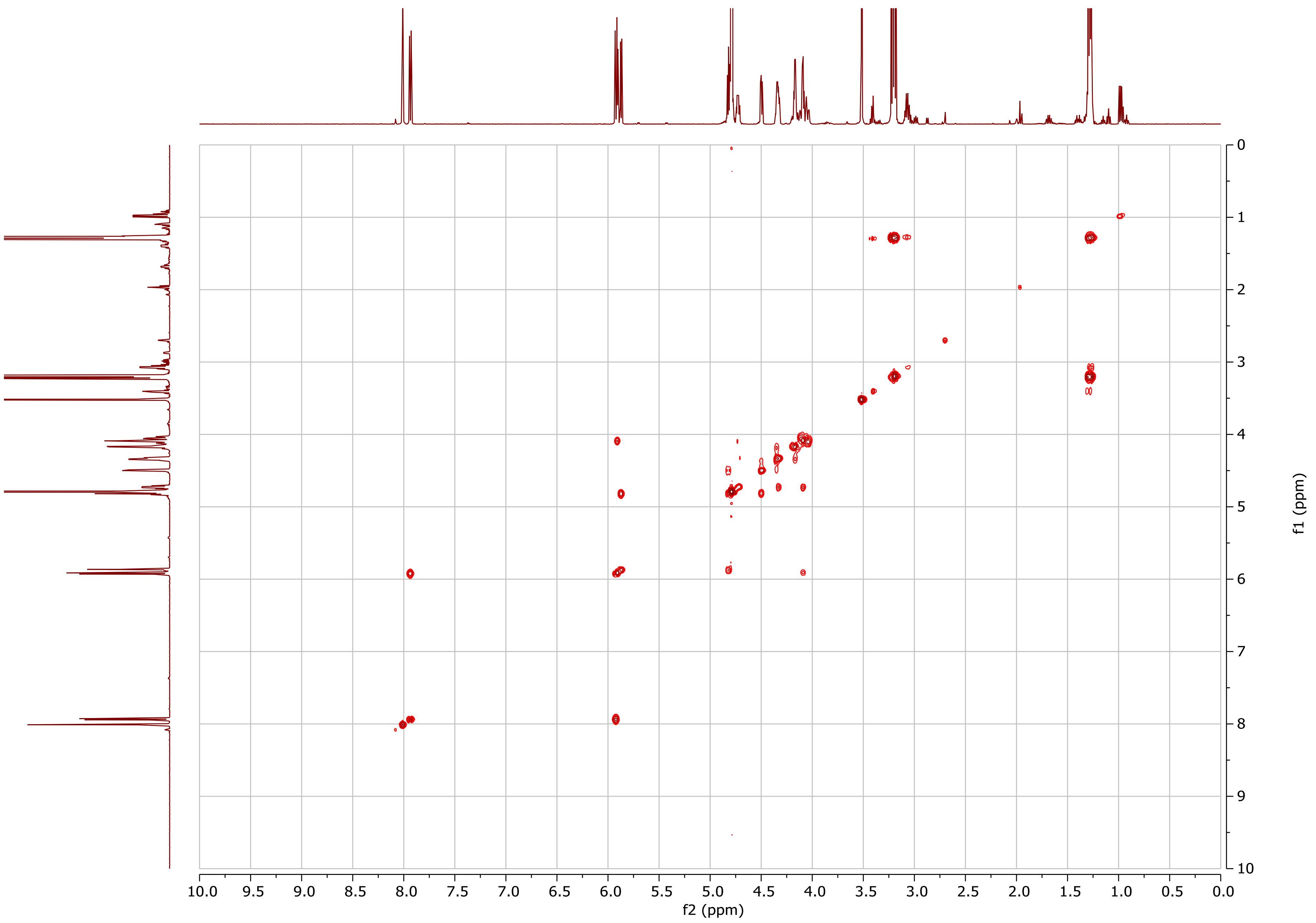

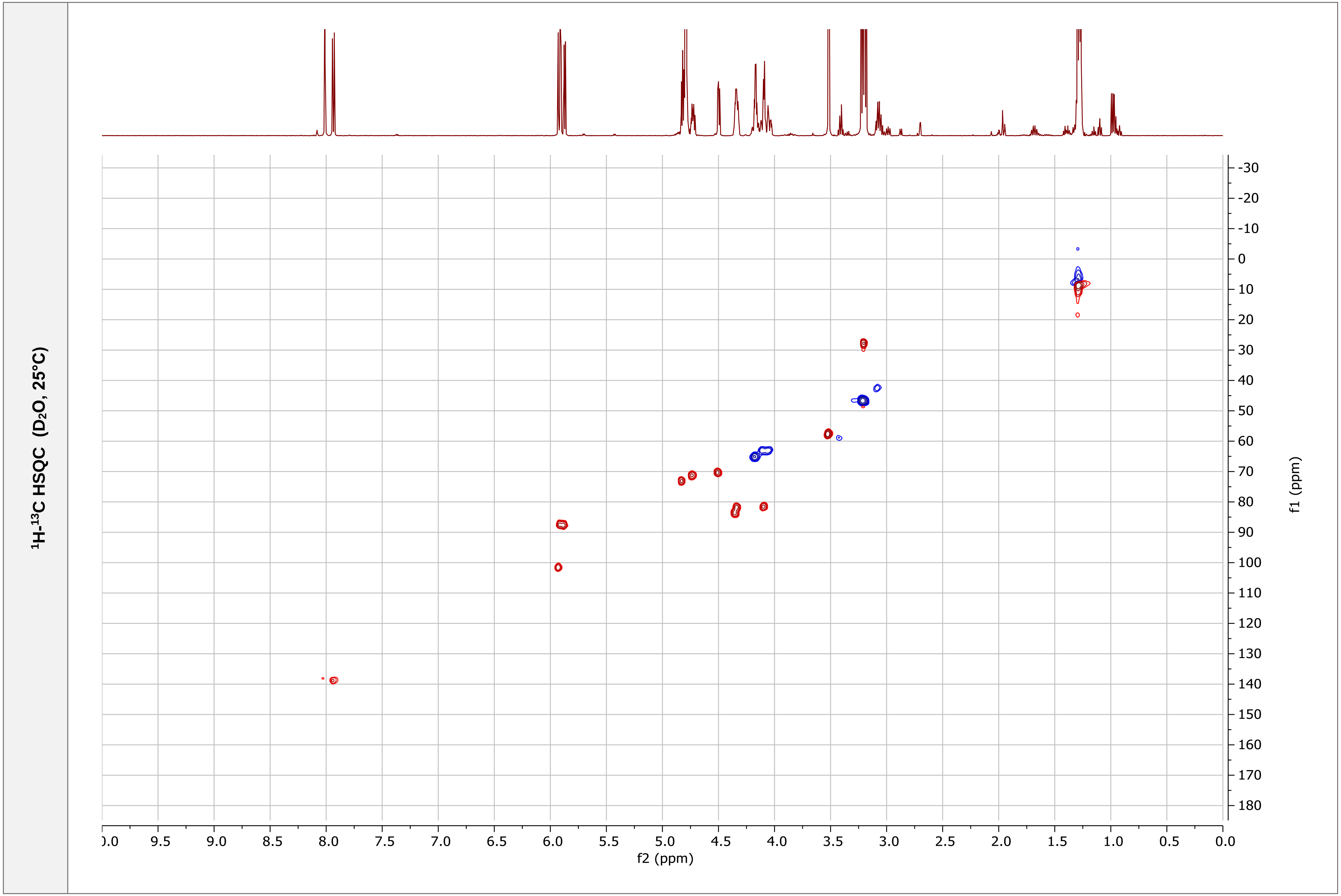

^{1}H-^{13}C HSQC (D$_2$O, 25°C)
f2 (ppm)
f1 (ppm)

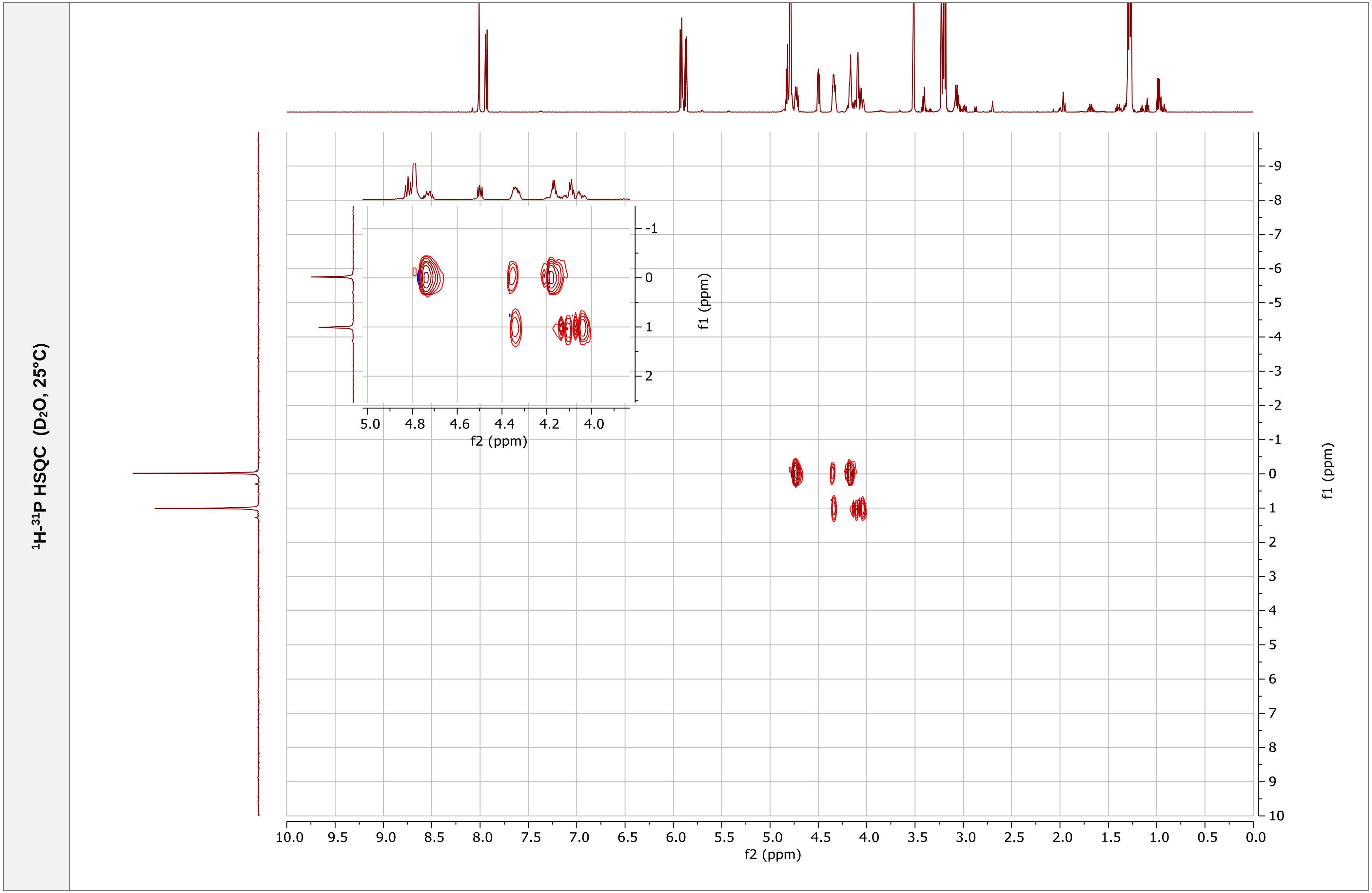
^1H-^{31}P HSQC (D$_2$O, 25°C)
f2 (ppm)
f1 (ppm)

(22) $p^{m3}U_{m}pG$	
**Chemical structure**	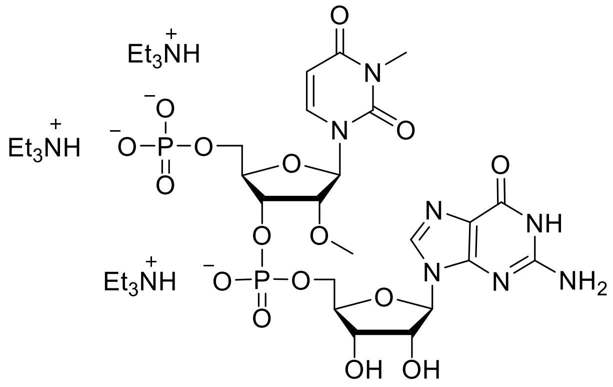
**RP HPLC** Abs. @ 254 nm	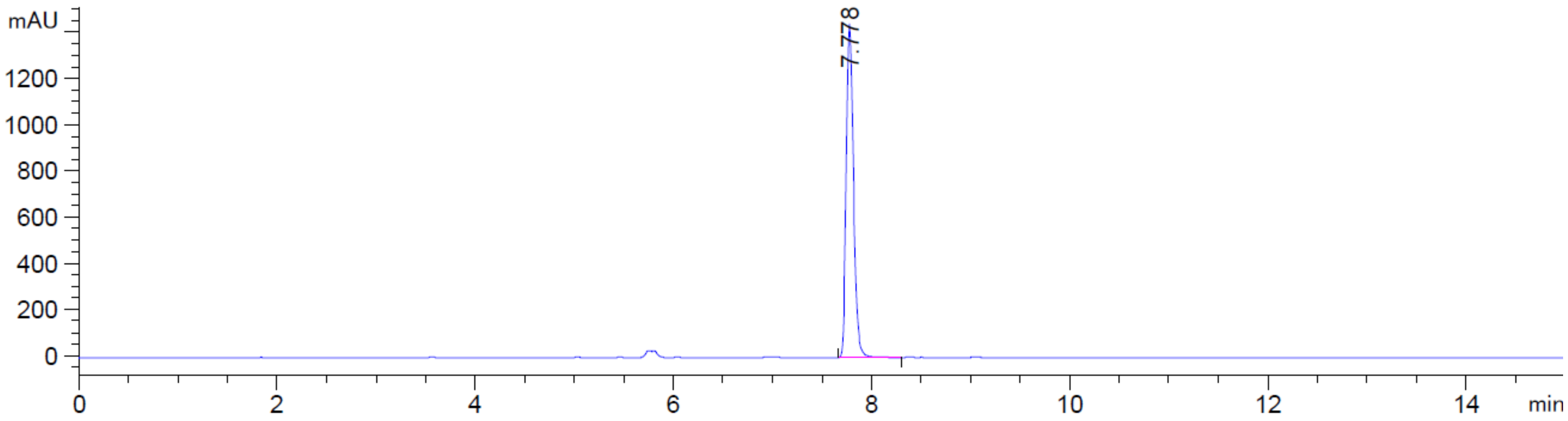

MS (-) ESI

(Calc. $[M-H]^-$ $C_{21}H_{28}N_7O_{16}P_2^-$ 696.10732)

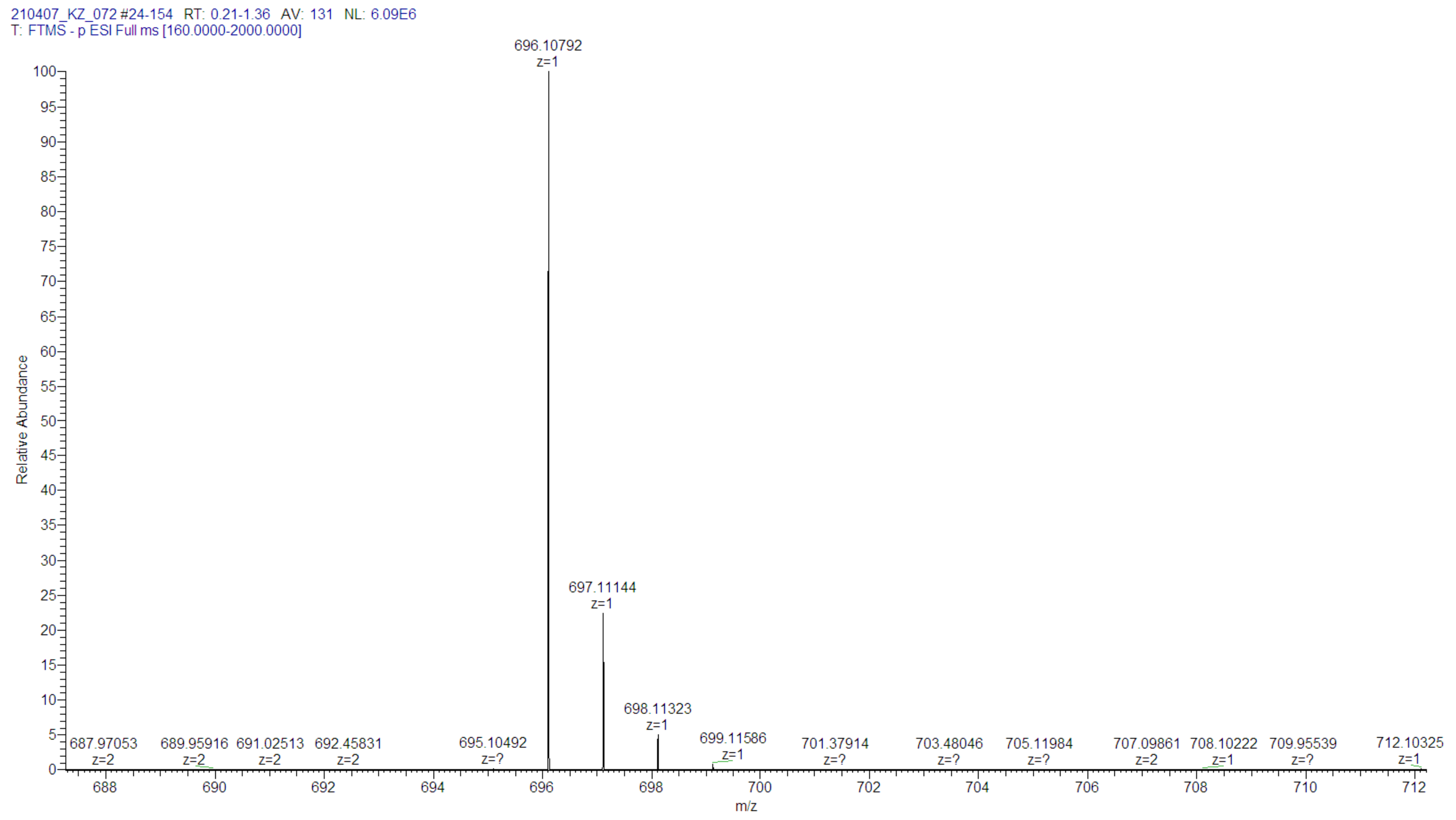

^{1}H NMR (500 MHz, D_2O, 25°C)

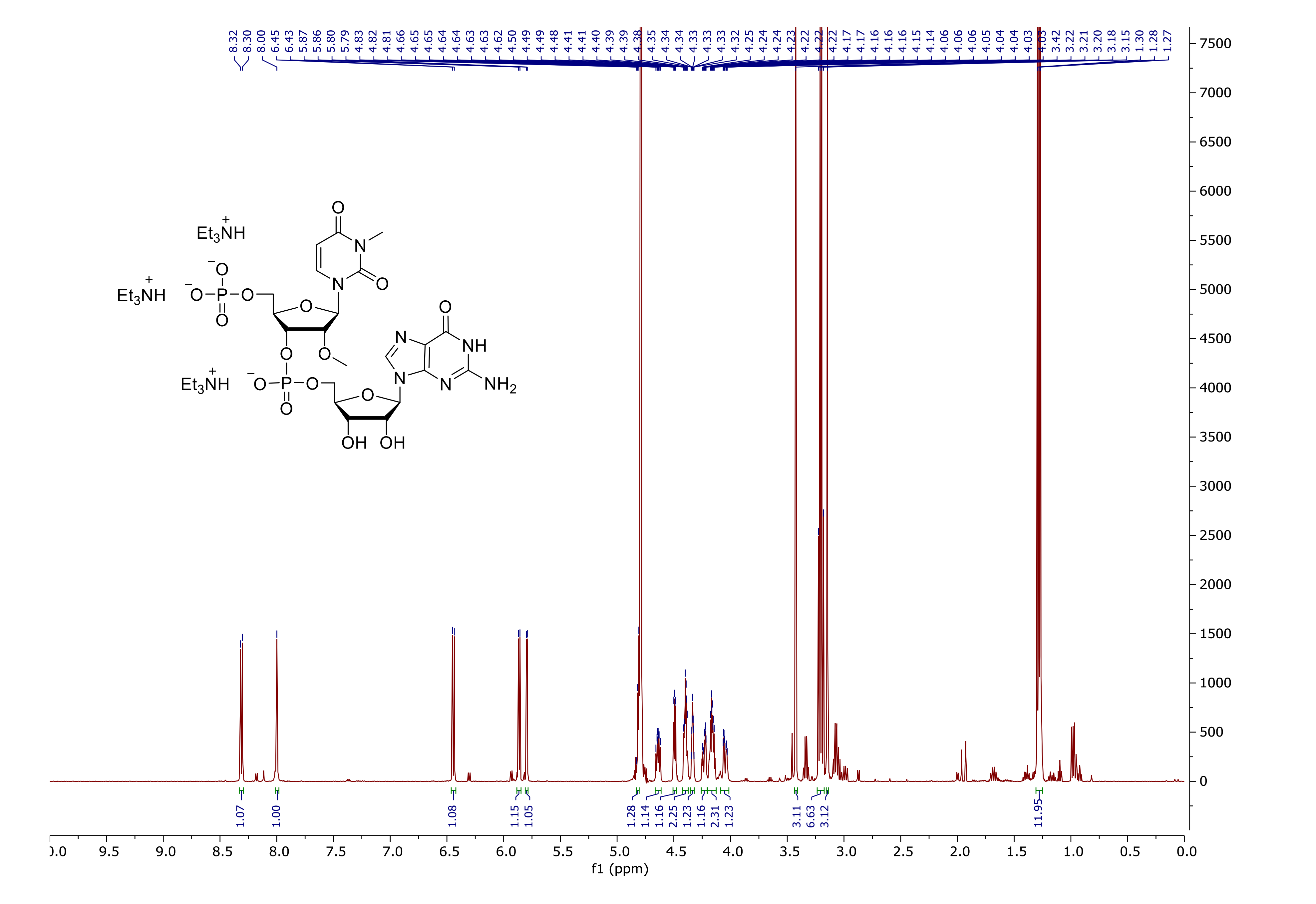

^{31}P NMR (202.5 MHz, D_2O, 25°C)

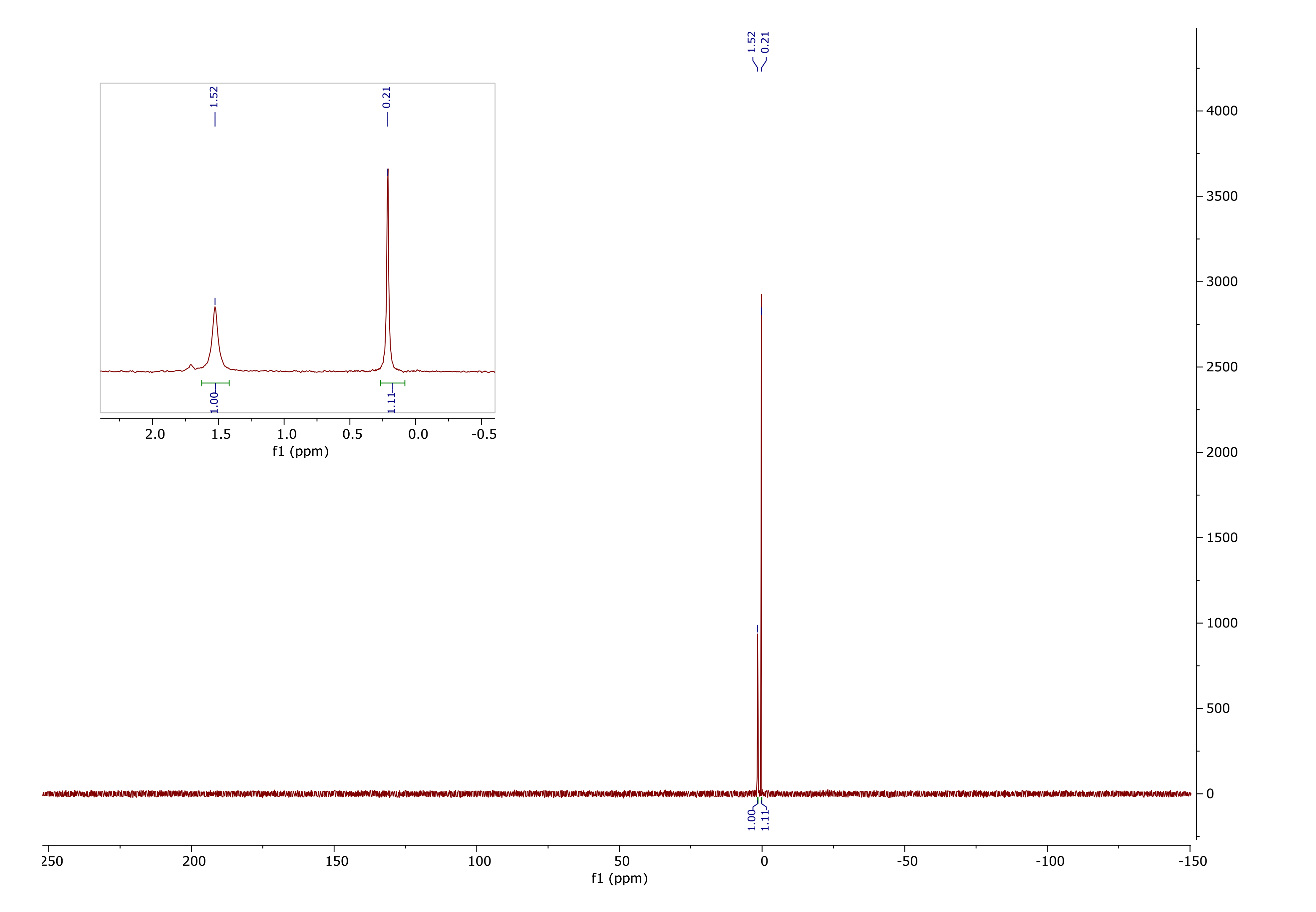

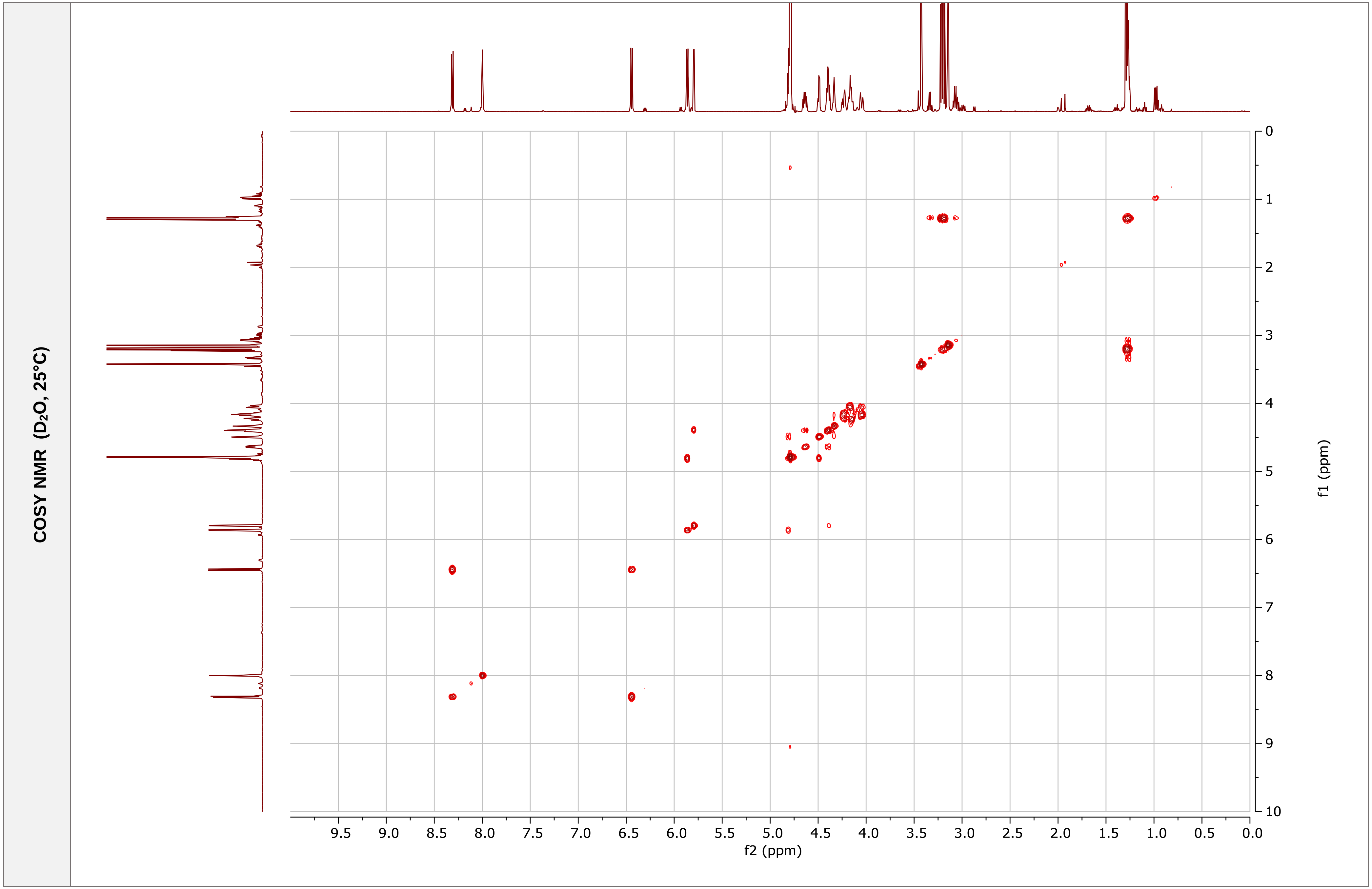
COSY NMR (D2O, 25°C)
f2 (ppm)
f1 (ppm)

^{1}H-^{13}C HSQC (D$_2$O, 25°C)

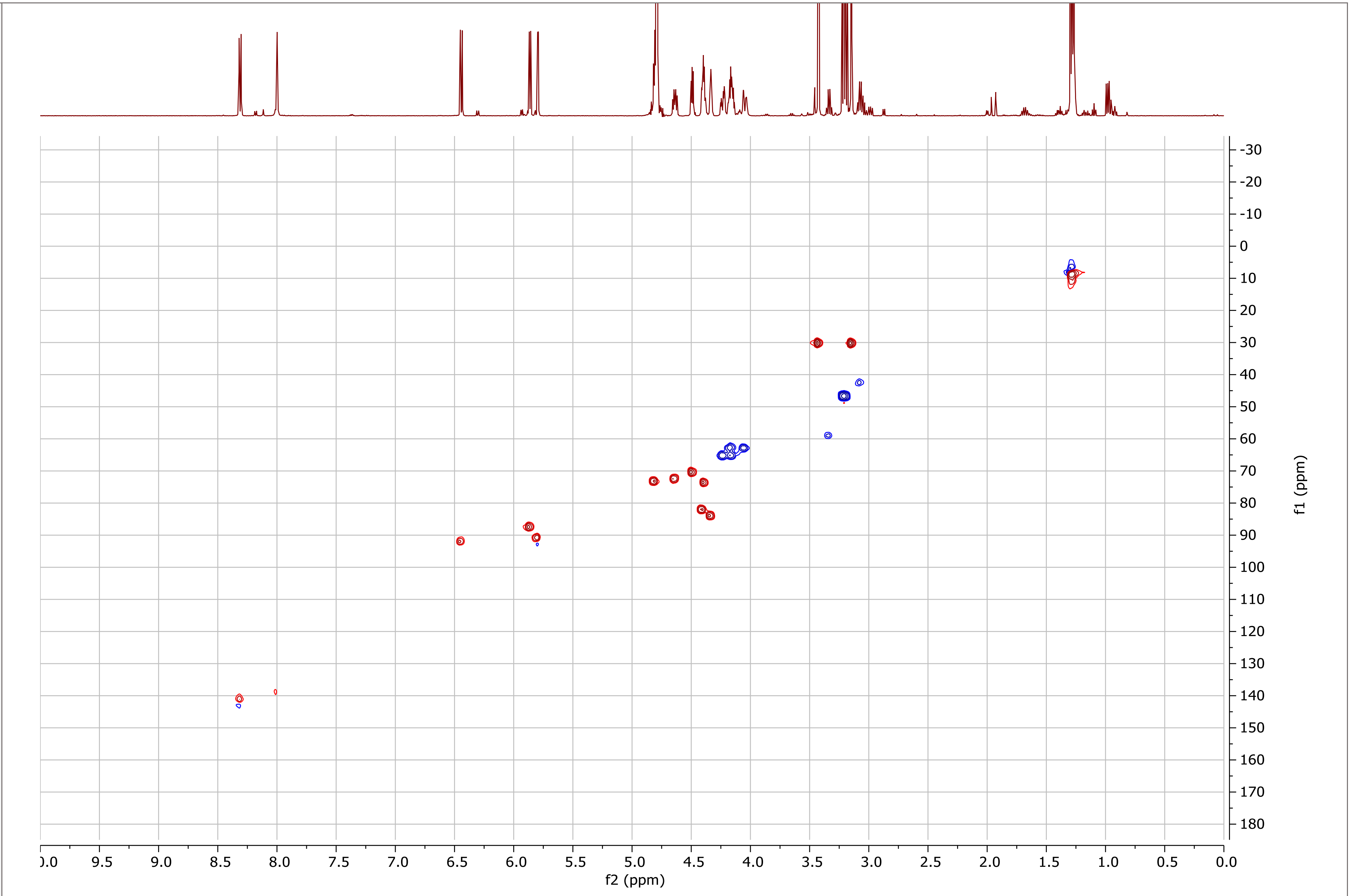

^{1}H-^{31}P HSQC (D_2O, 25°C)

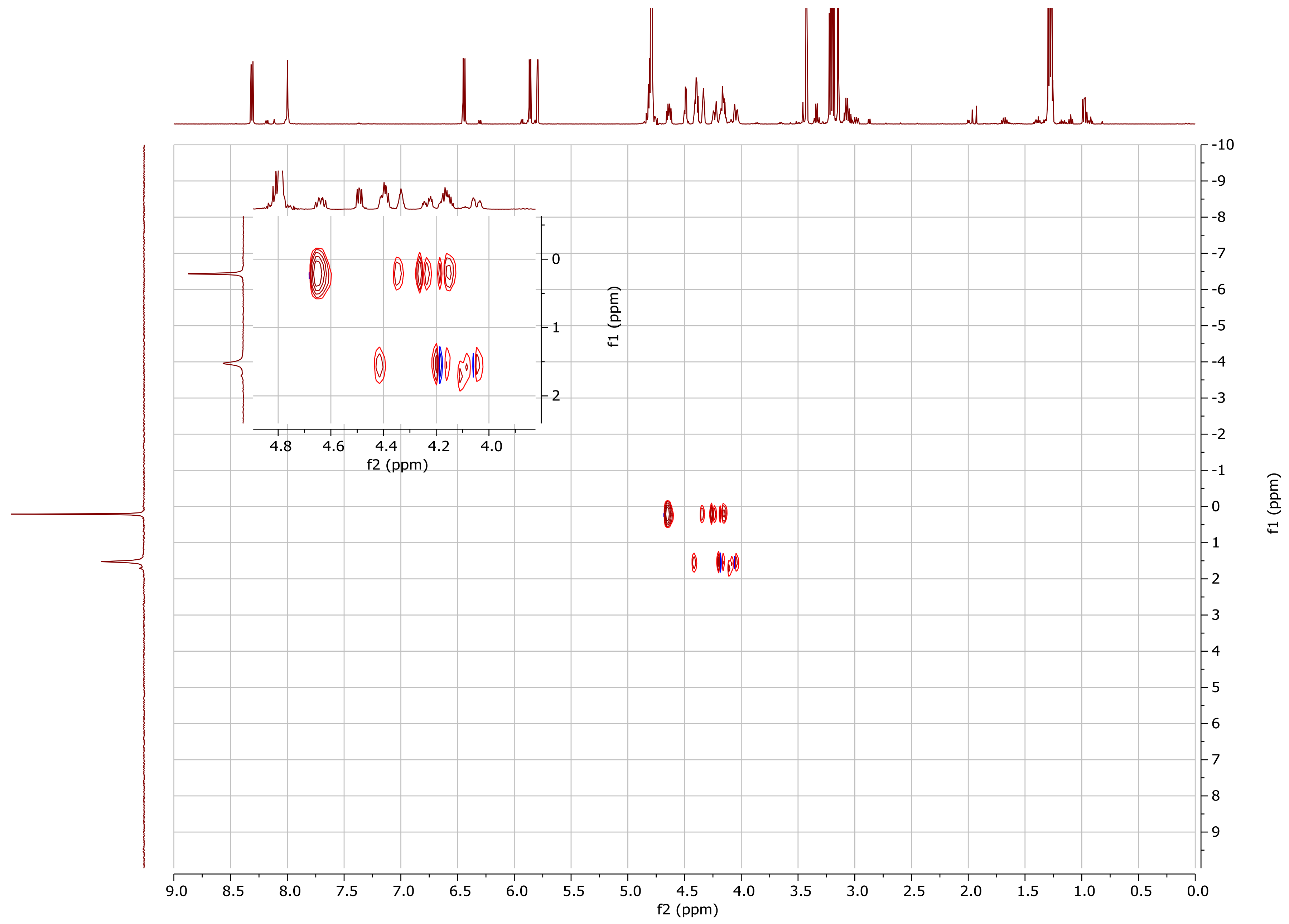

(23) G^{2nBn3}TC	
**Chemical structure**	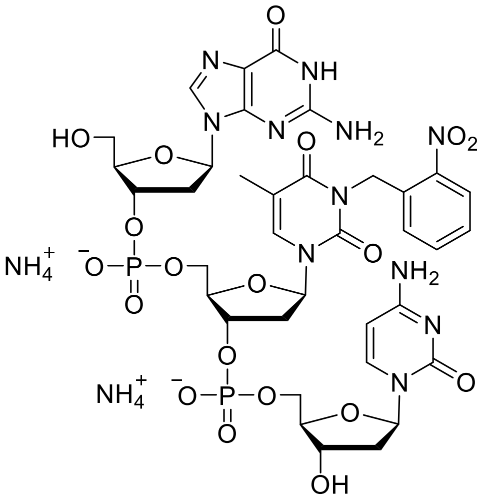
**RP HPLC** Abs. @ 254 nm	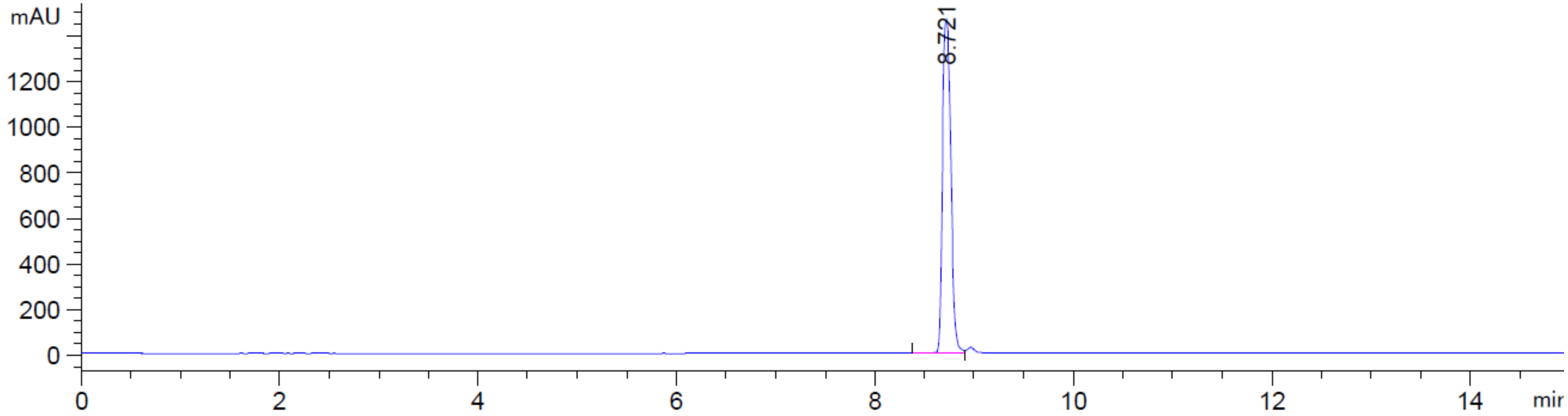

MS (-) ESI

(Calc. $[M-H]^-$ $C_{36}H_{42}N_{11}O_{19}P_2^-$ 994.21391)

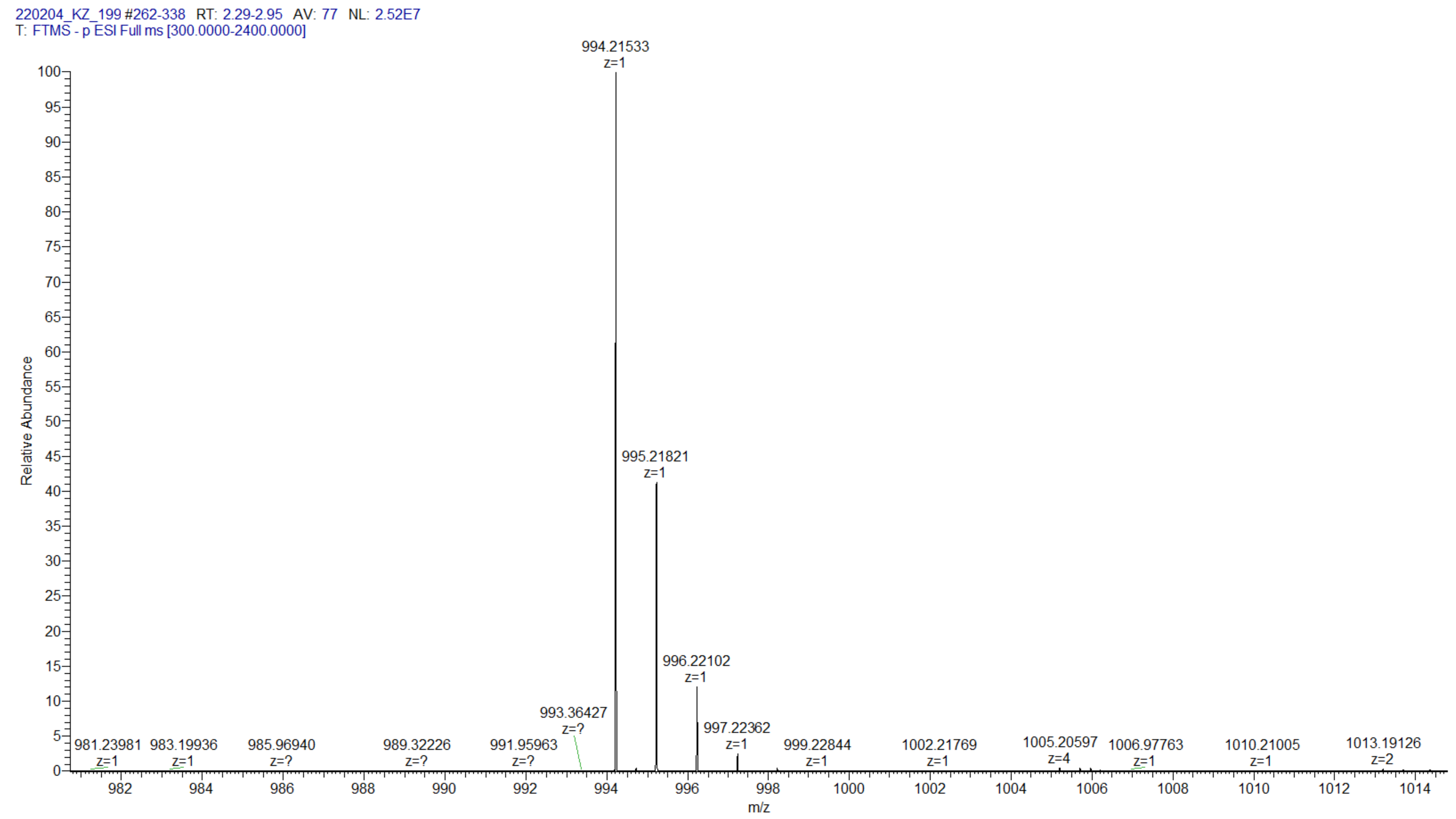

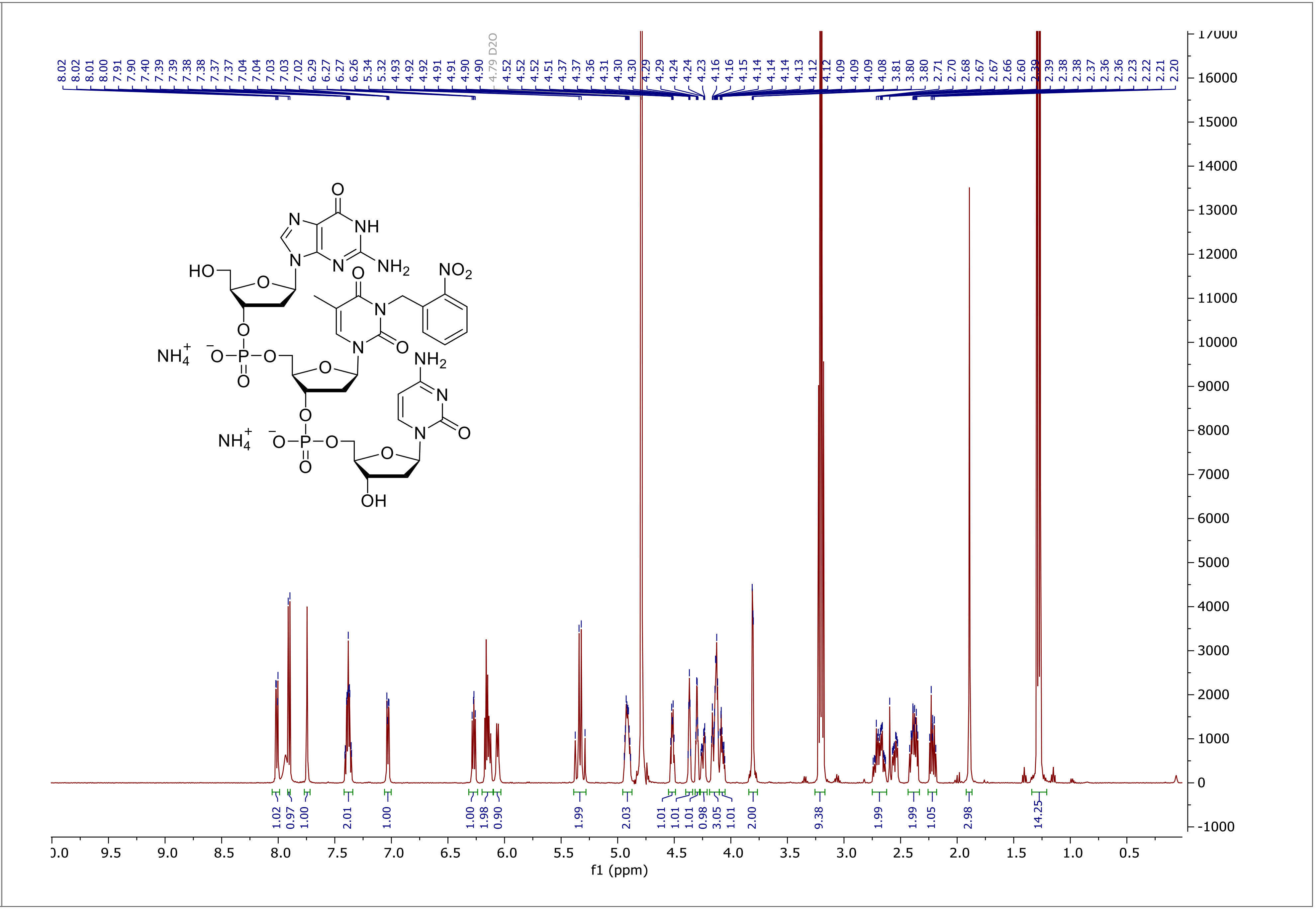

^1H NMR (500 MHz, D_2O, 25°C)

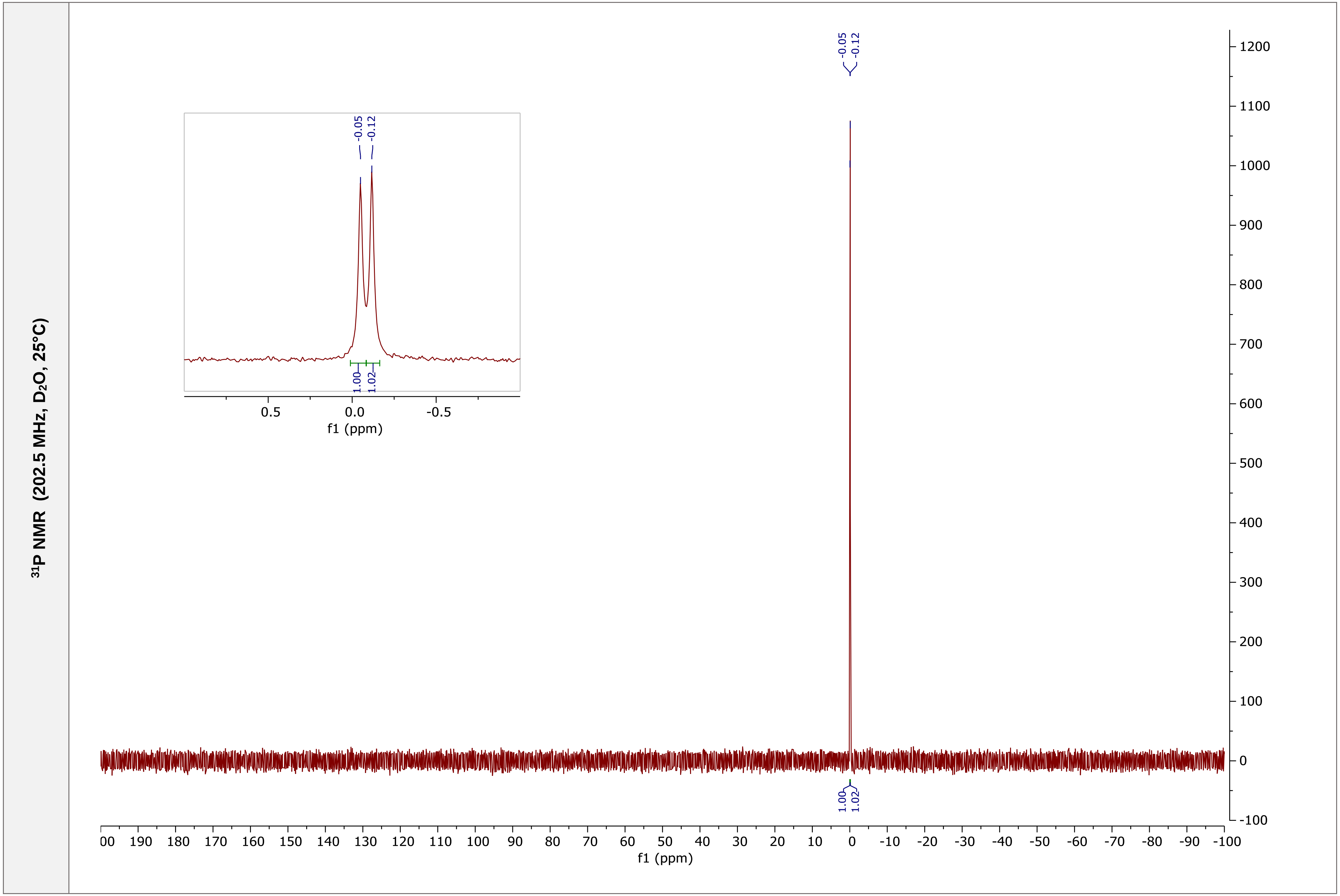

^{31}P NMR (202.5 MHz, D_2O, 25°C)
-0.05
-0.12
1.00
1.02
0.5
0.0
-0.5
f1 (ppm)
f1 (ppm)

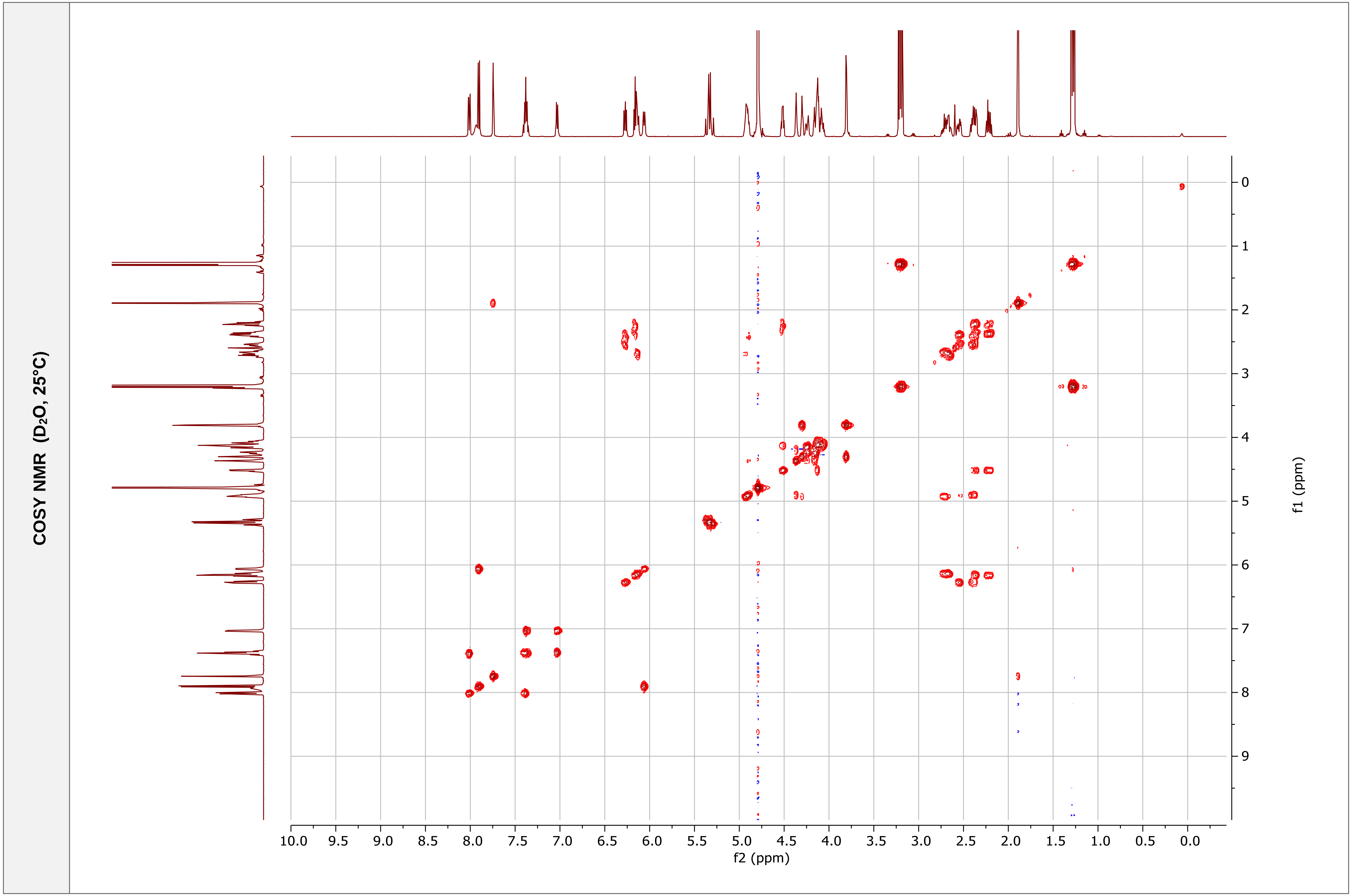
COSY NMR (D2O, 25°C)
f2 (ppm)
f1 (ppm)
10.0 9.5 9.0 8.5 8.0 7.5 7.0 6.5 6.0 5.5 5.0 4.5 4.0 3.5 3.0 2.5 2.0 1.5 1.0 0.5 0.0
0 1 2 3 4 5 6 7 8 9

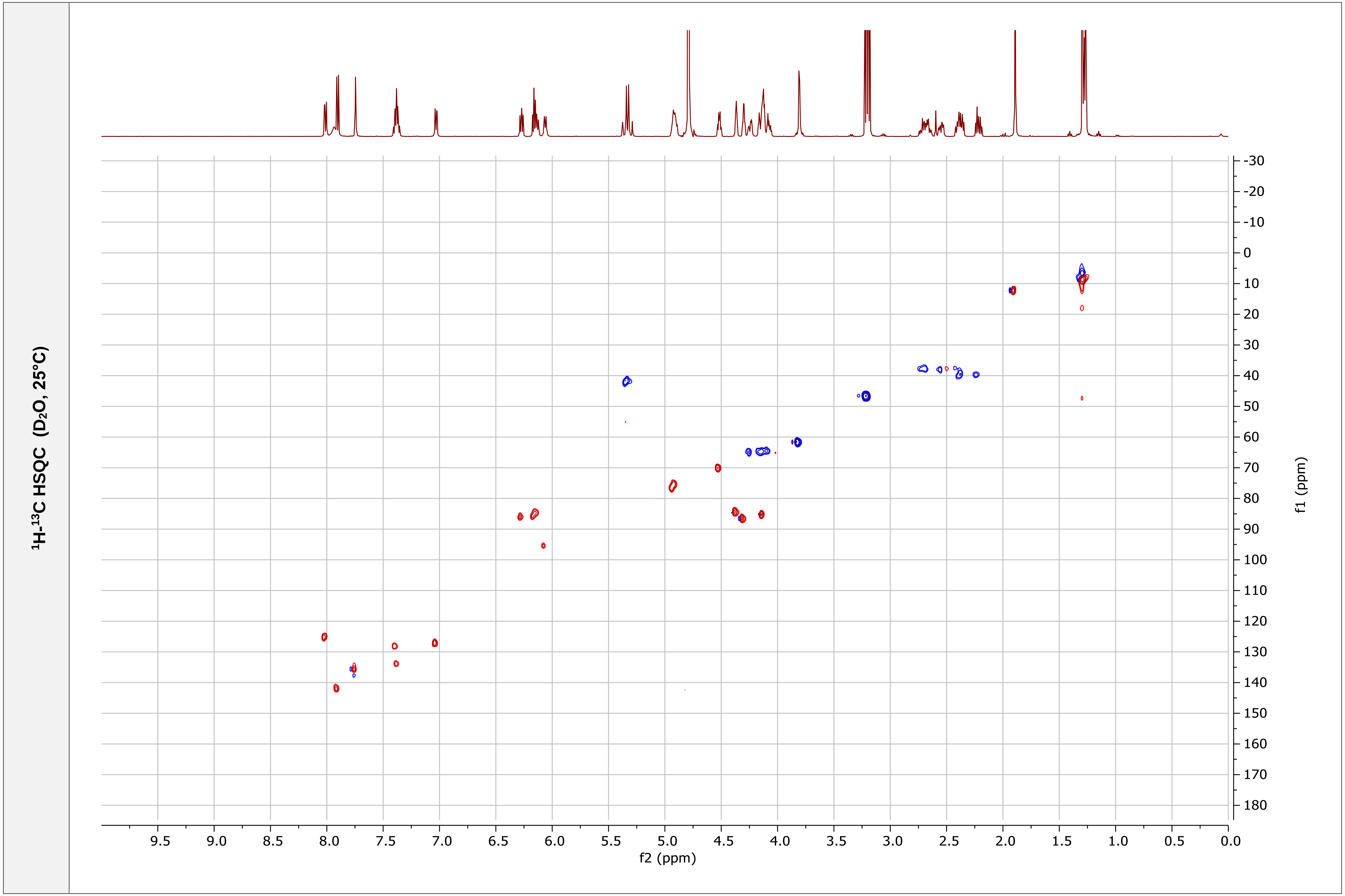
^{1}H-^{13}C HSQC (D$_2$O, 25°C)
f2 (ppm)
f1 (ppm)

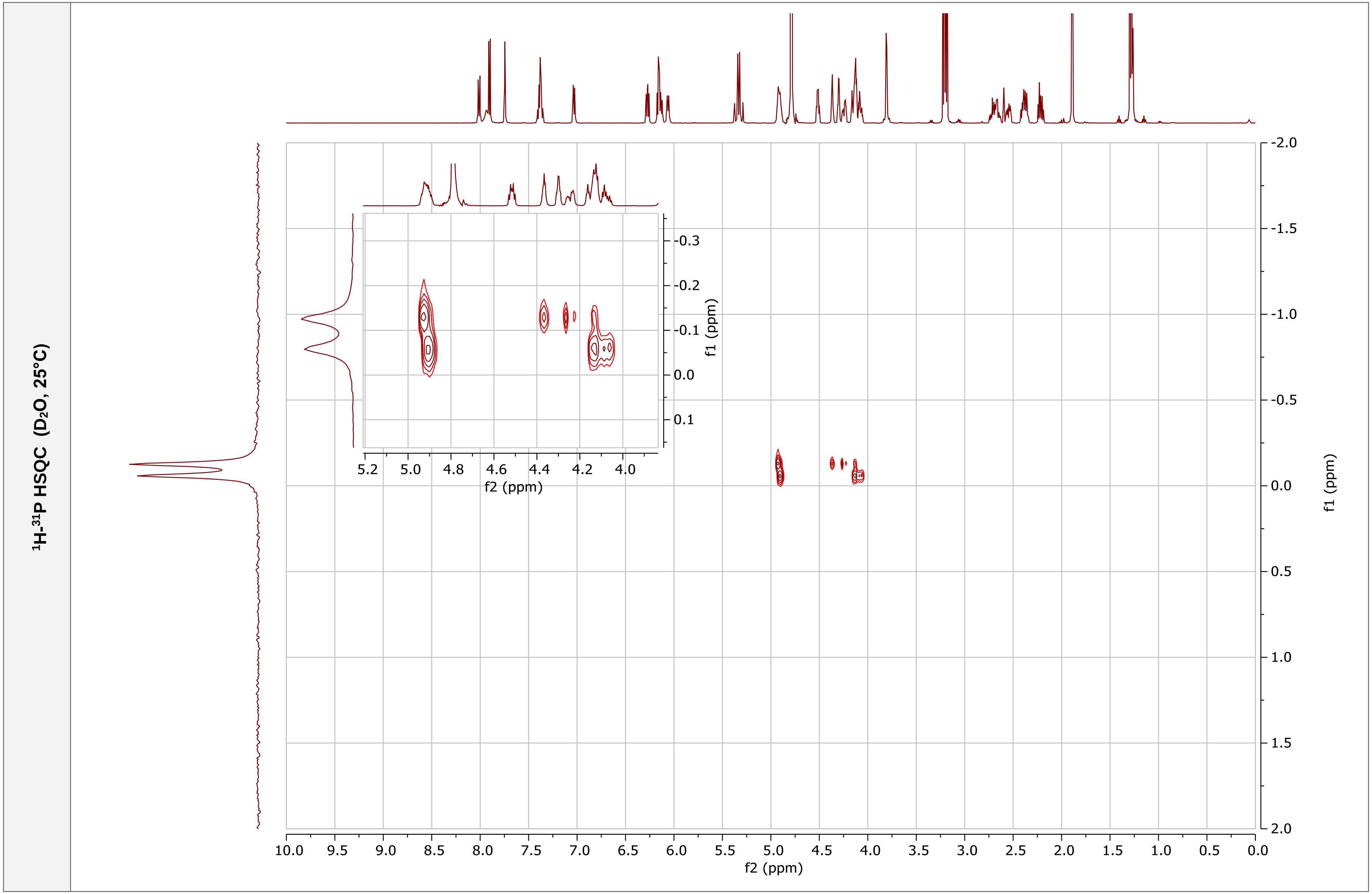

^1H-^{31}P HSQC (D$_2$O, 25°C)
f2 (ppm)
f1 (ppm)

(24) $U^{m2}GU$

Chemical structure

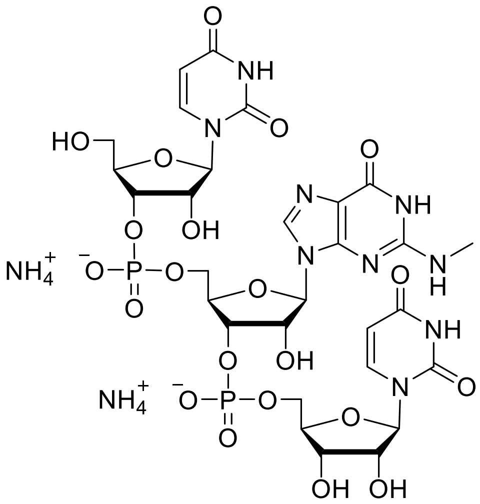

RP HPLC
Abs. @ 254 nm

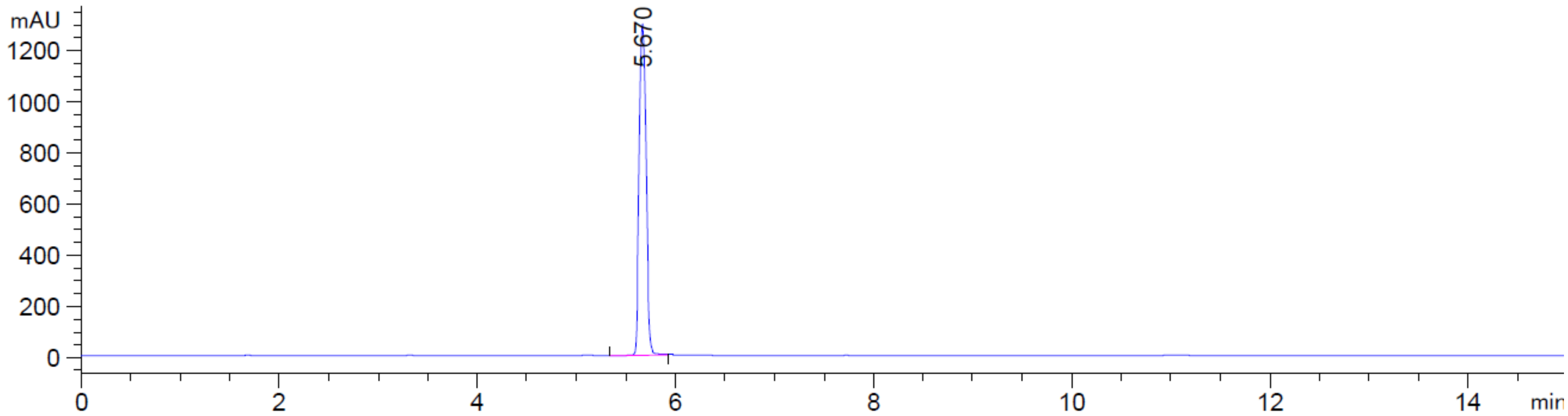

MS (-) ESI

(Calc. [M-H]$^-$ $C_{29}H_{36}N_9O_{21}P_2^-$ 908.15065)

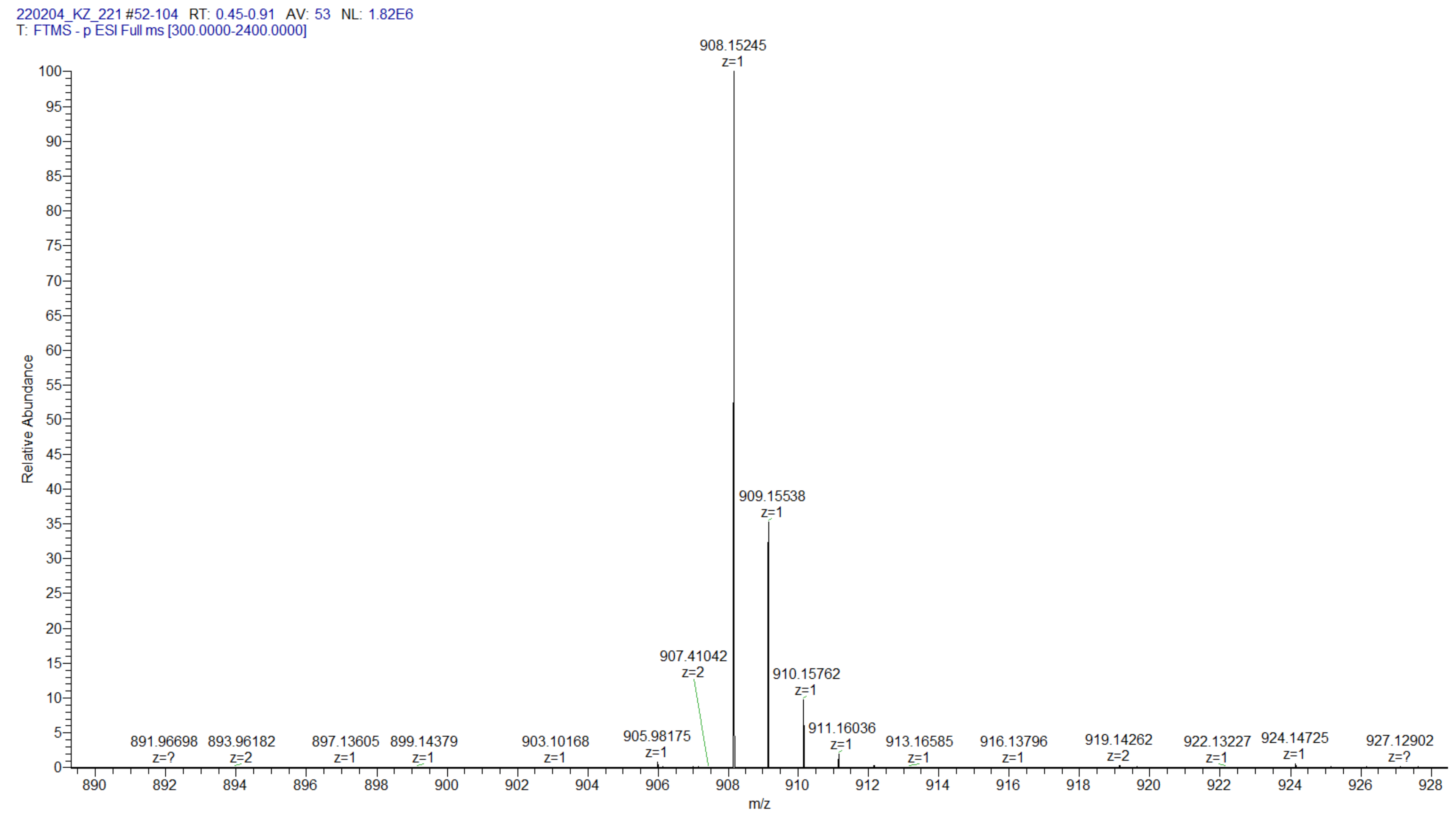

^1H NMR (500 MHz, D_2O, 25°C)

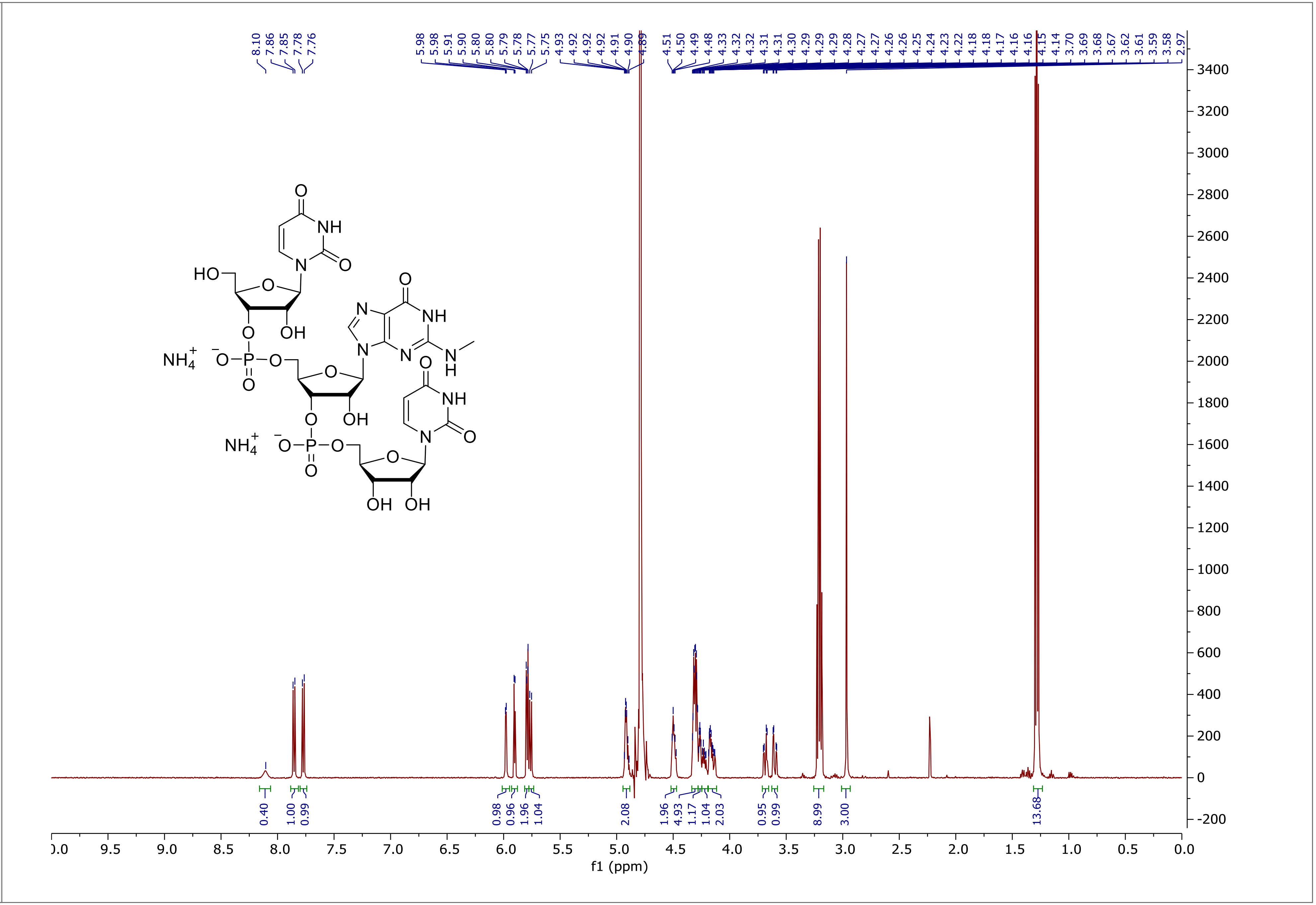

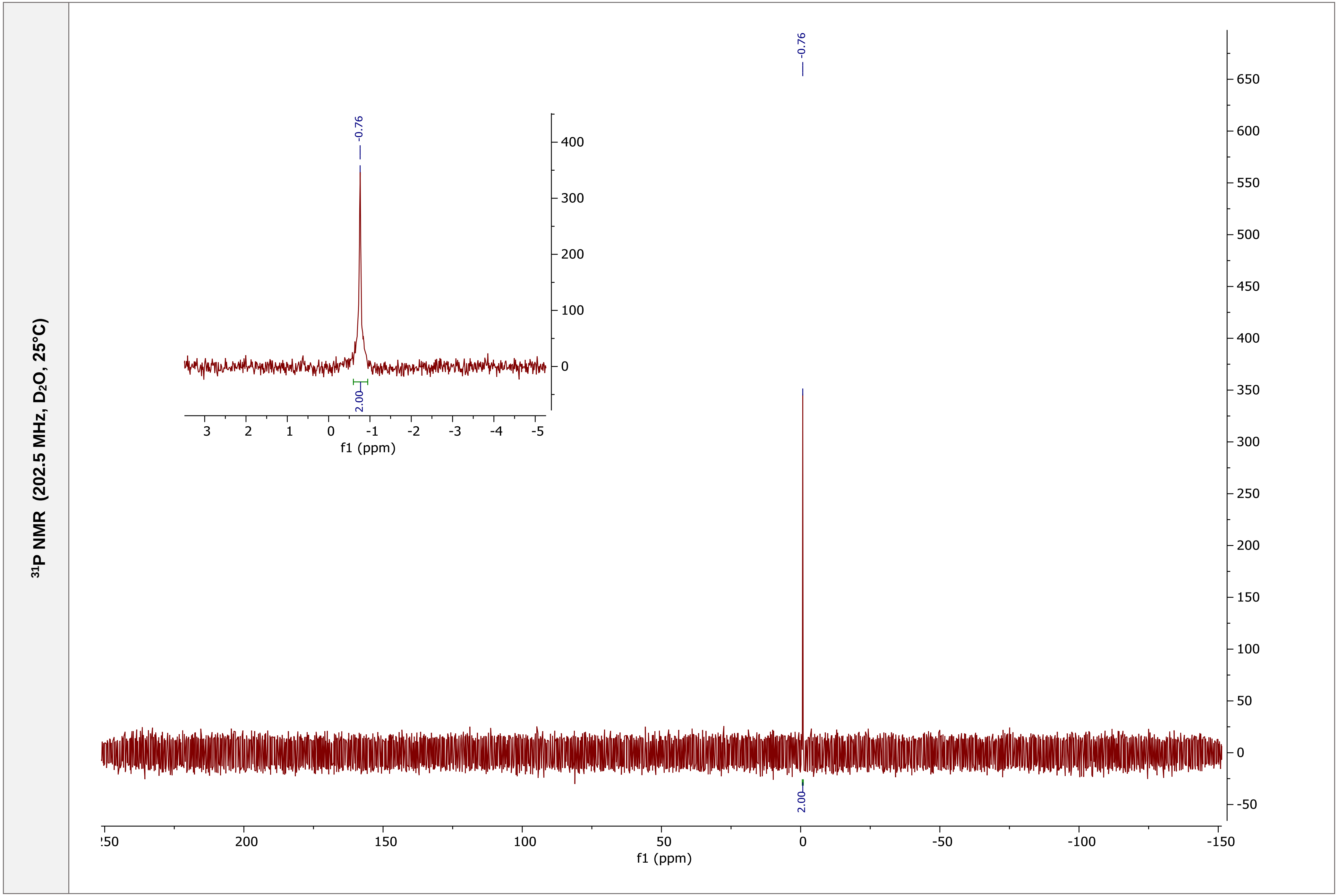

^{31}P NMR (202.5 MHz, D$_2$O, 25°C)
-0.76
2.00
f1 (ppm)

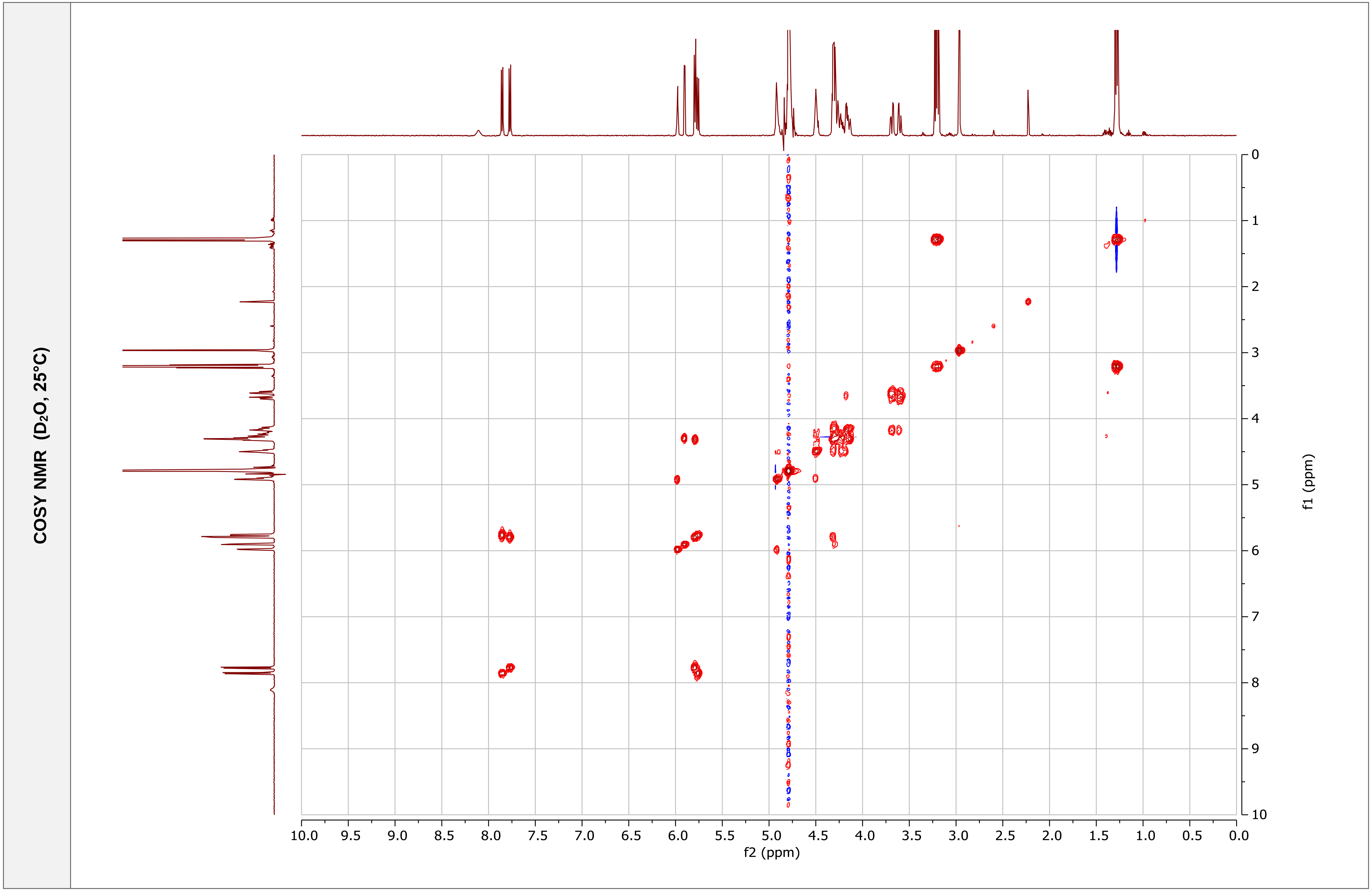
COSY NMR (D2O, 25°C)
f2 (ppm)
f1 (ppm)

^1H-^{13}C HSQC (D$_2$O, 25°C)

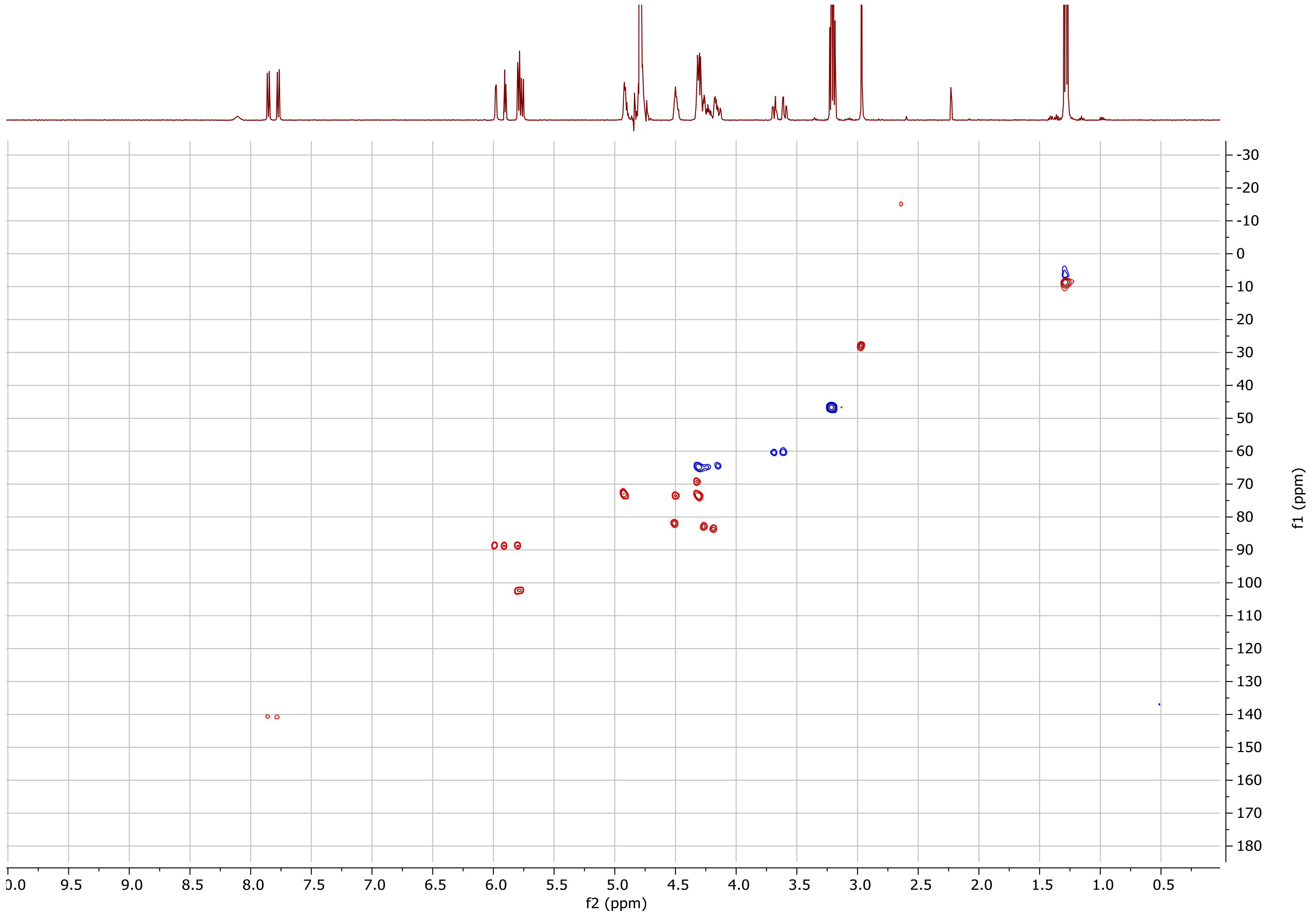

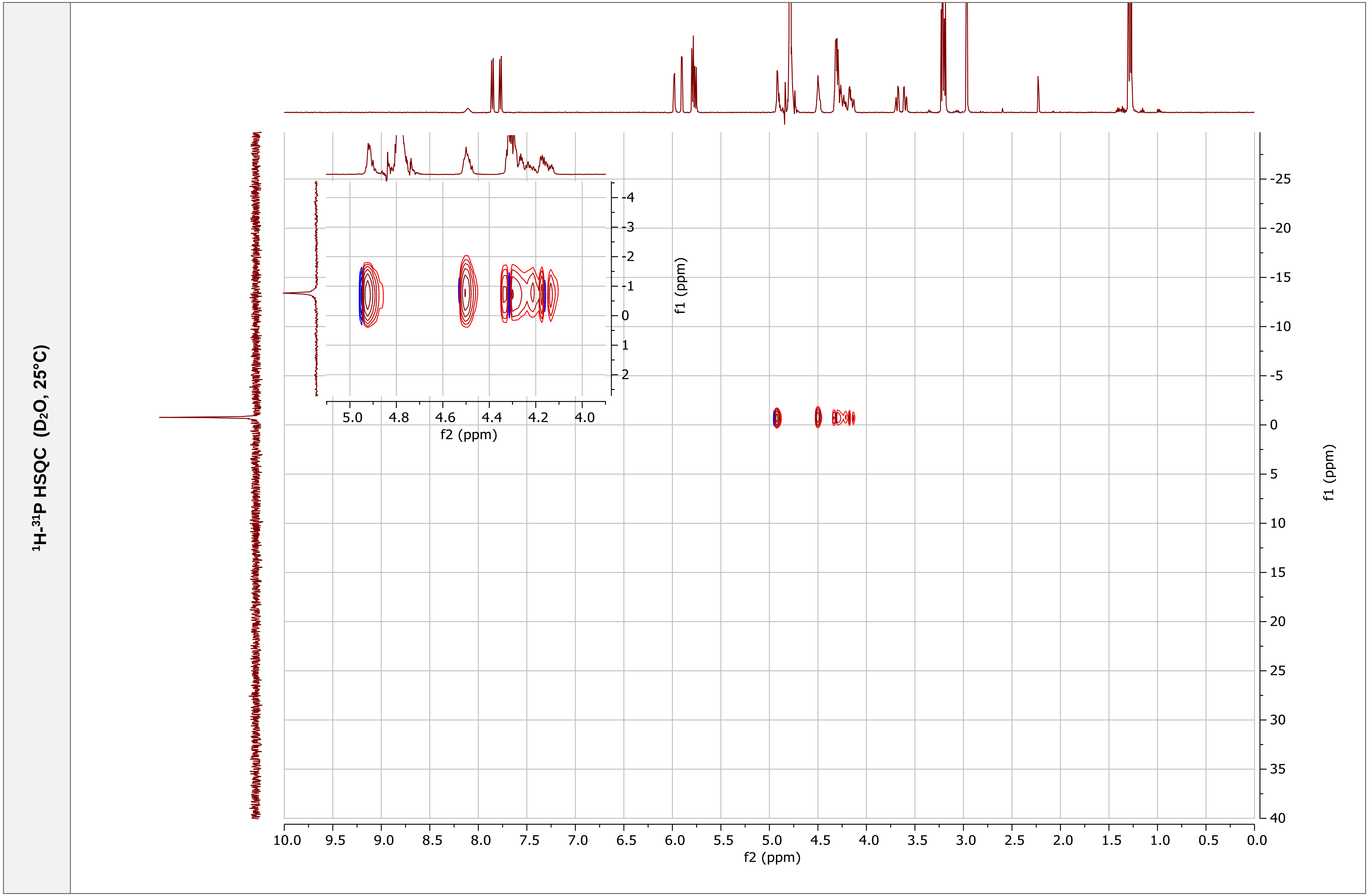

1H-31P HSQC (D2O, 25°C)
f2 (ppm)
f1 (ppm)
10.0 9.5 9.0 8.5 8.0 7.5 7.0 6.5 6.0 5.5 5.0 4.5 4.0 3.5 3.0 2.5 2.0 1.5 1.0 0.5 0.0
-25 -20 -15 -10 -5 0 5 10 15 20 25 30 35 40
5.0 4.8 4.6 4.4 4.2 4.0
-4 -3 -2 -1 0 1 2

(25) U^{m1}GU

Chemical structure

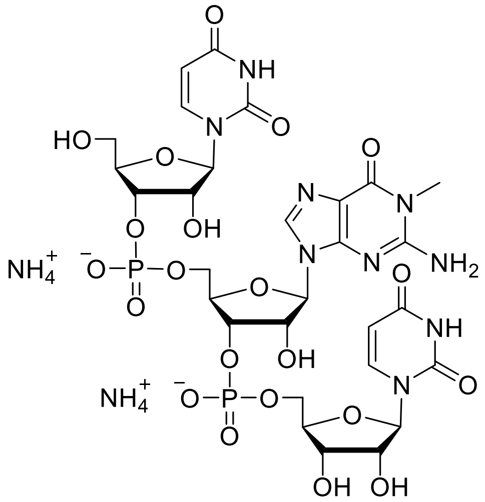

RP HPLC
Abs. @ 254 nm

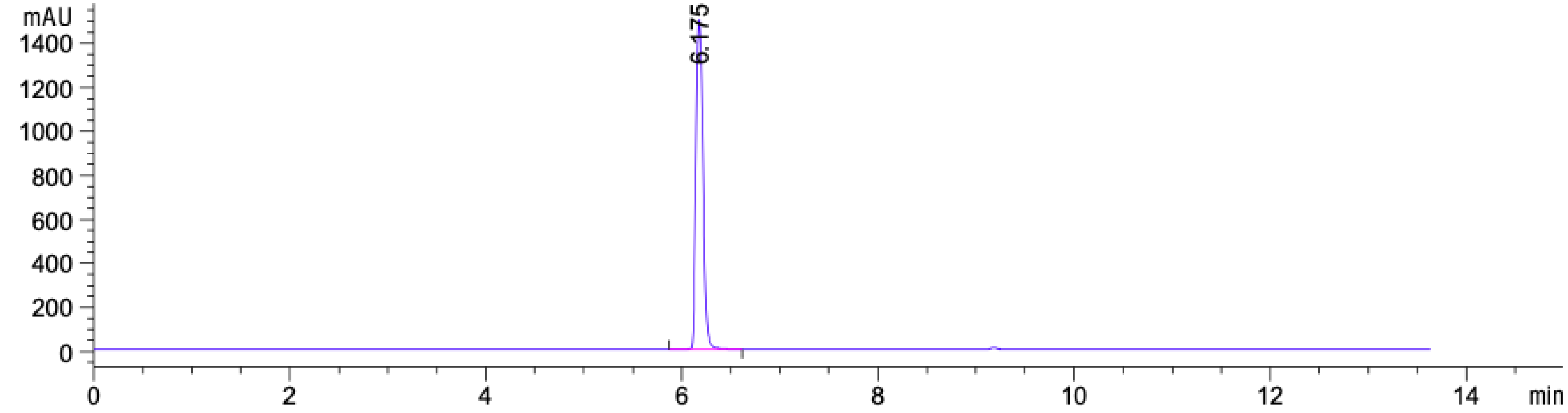

MS (-) ESI

(Calc. [M-H]⁻ $C_{29}H_{36}N_9O_{21}P_2^-$ 908.15065)

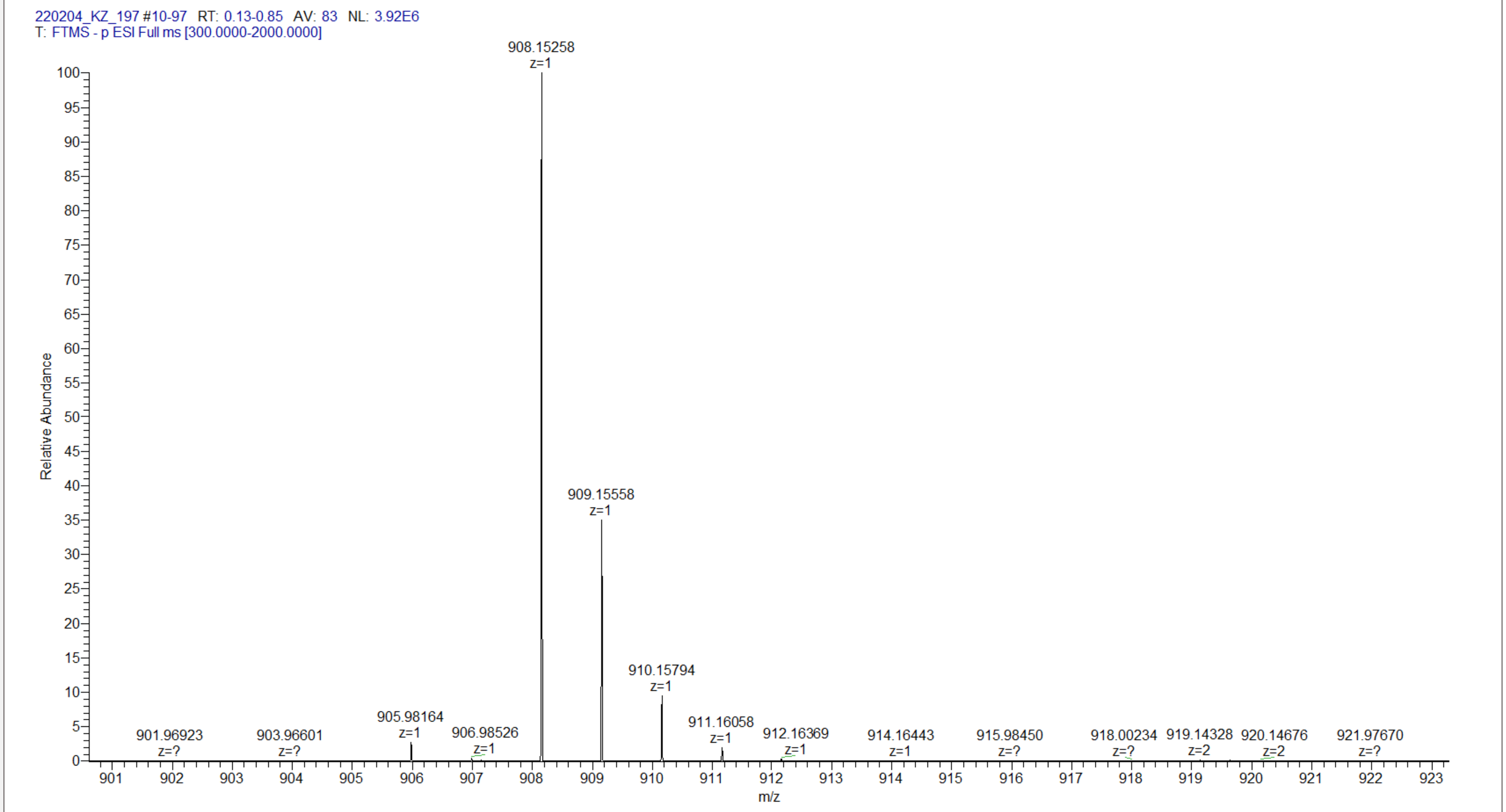

^1H NMR (500 MHz, D_2O, 25°C)

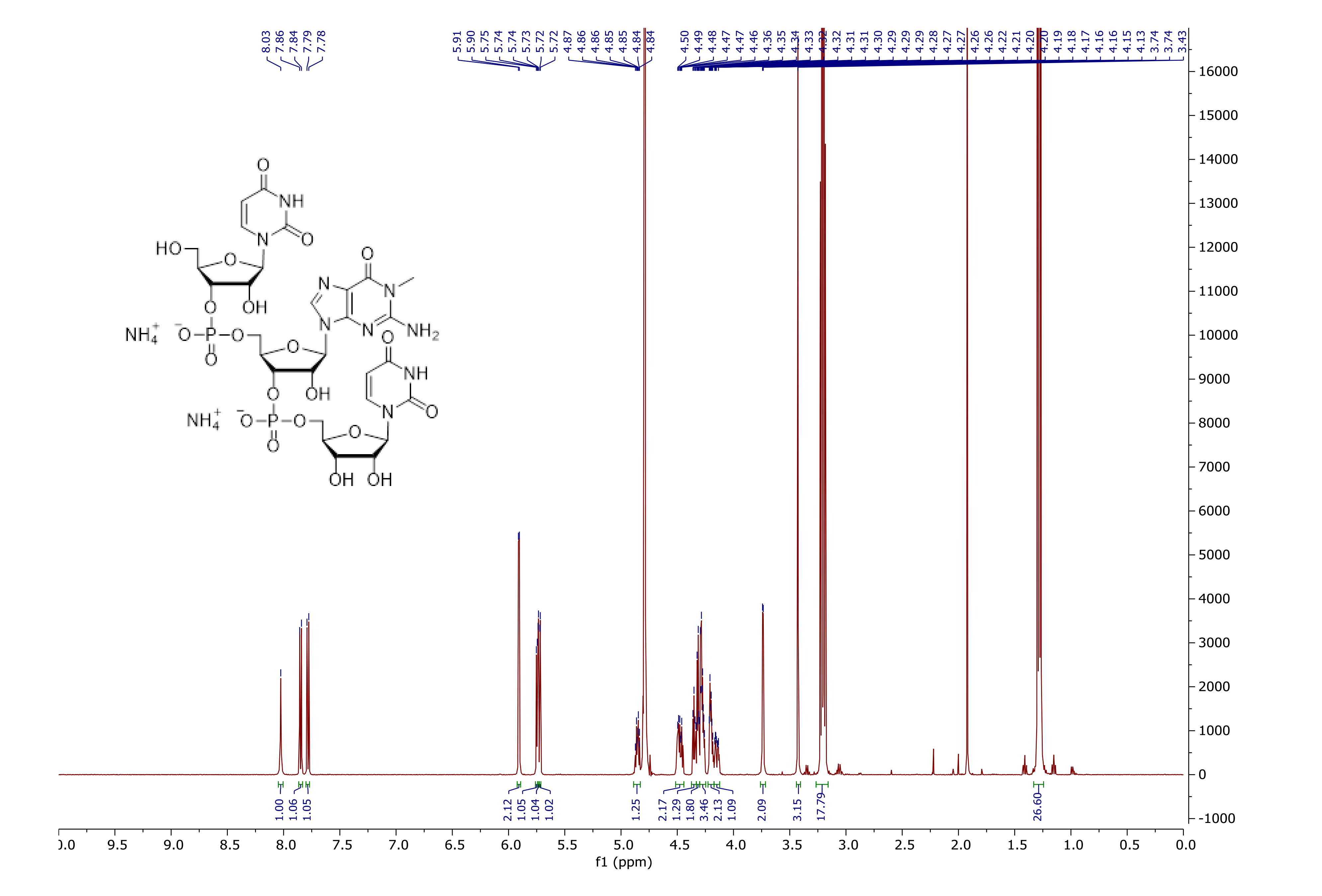

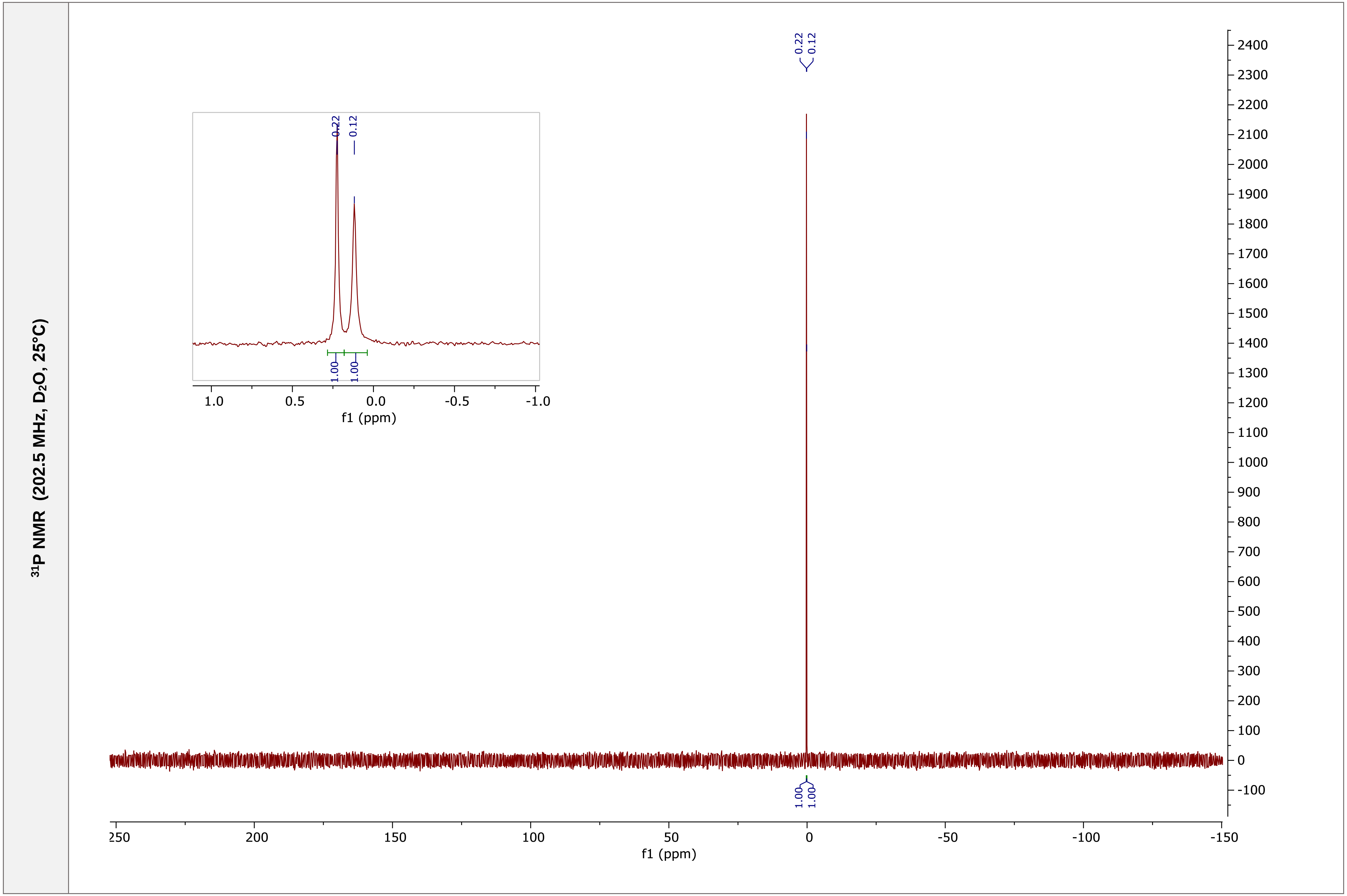

^{31}P NMR (202.5 MHz, D_2O, 25°C)
0.22
0.12
1.00
1.00
1.0
0.5
0.0
-0.5
-1.0
f1 (ppm)
2400
2300
2200
2100
2000
1900
1800
1700
1600
1500
1400
1300
1200
1100
1000
900
800
700
600
500
400
300
200
100
0
-100
250
200
150
100
50
0
-50
-100
-150
f1 (ppm)

COSY NMR (D$_2$O, 25°C)

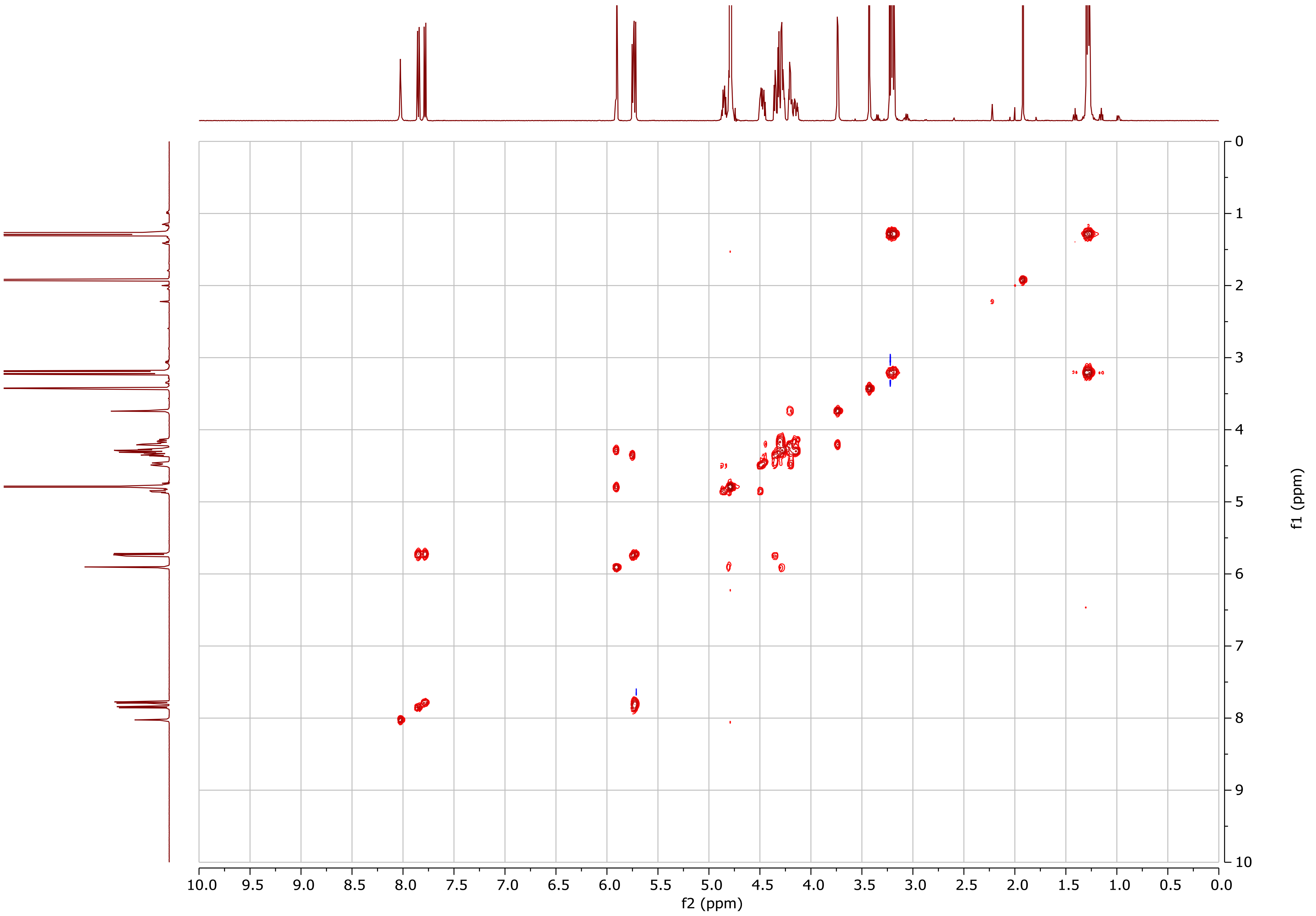

^{1}H-^{13}C HSQC (D$_2$O, 25°C)

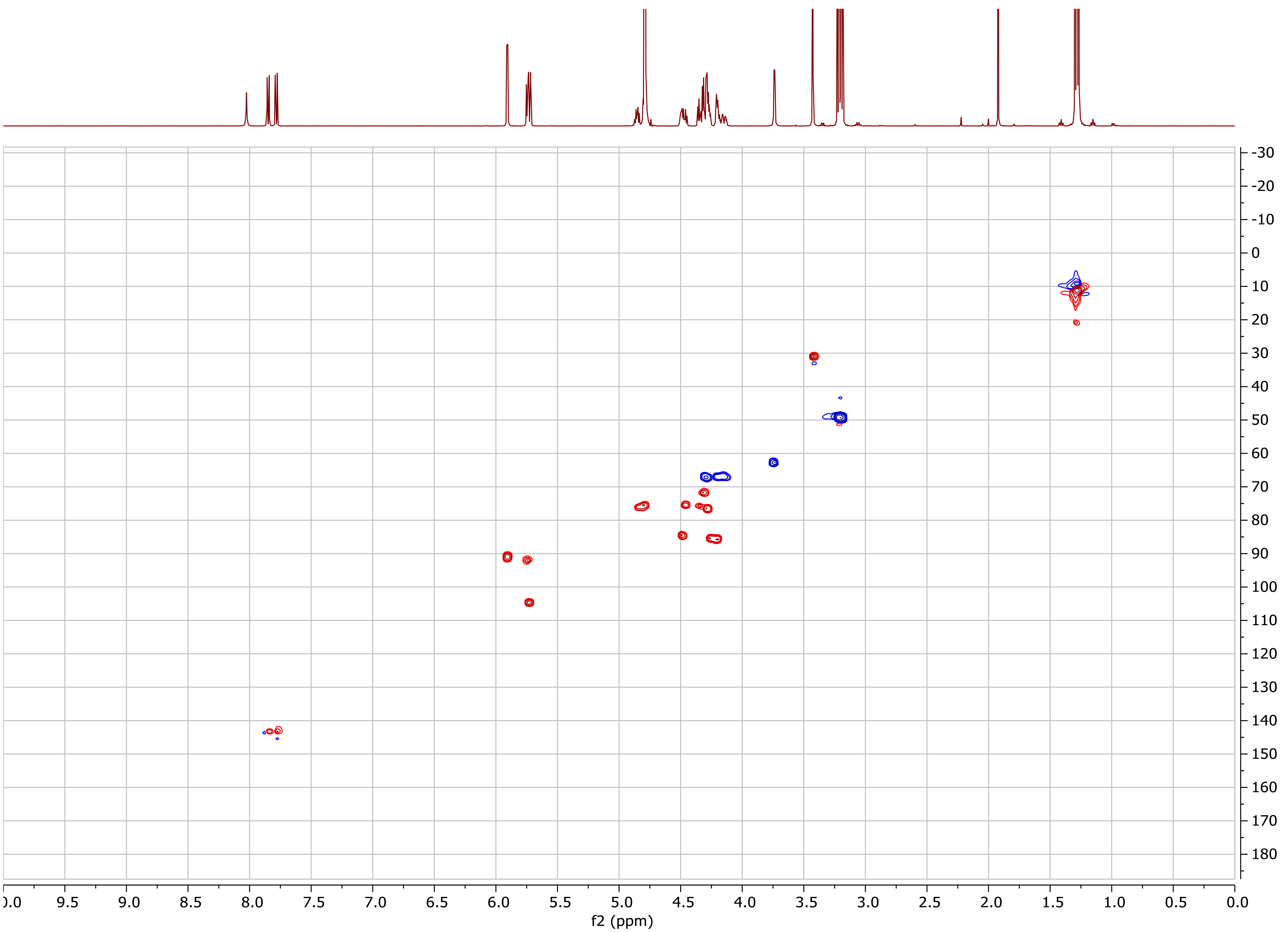

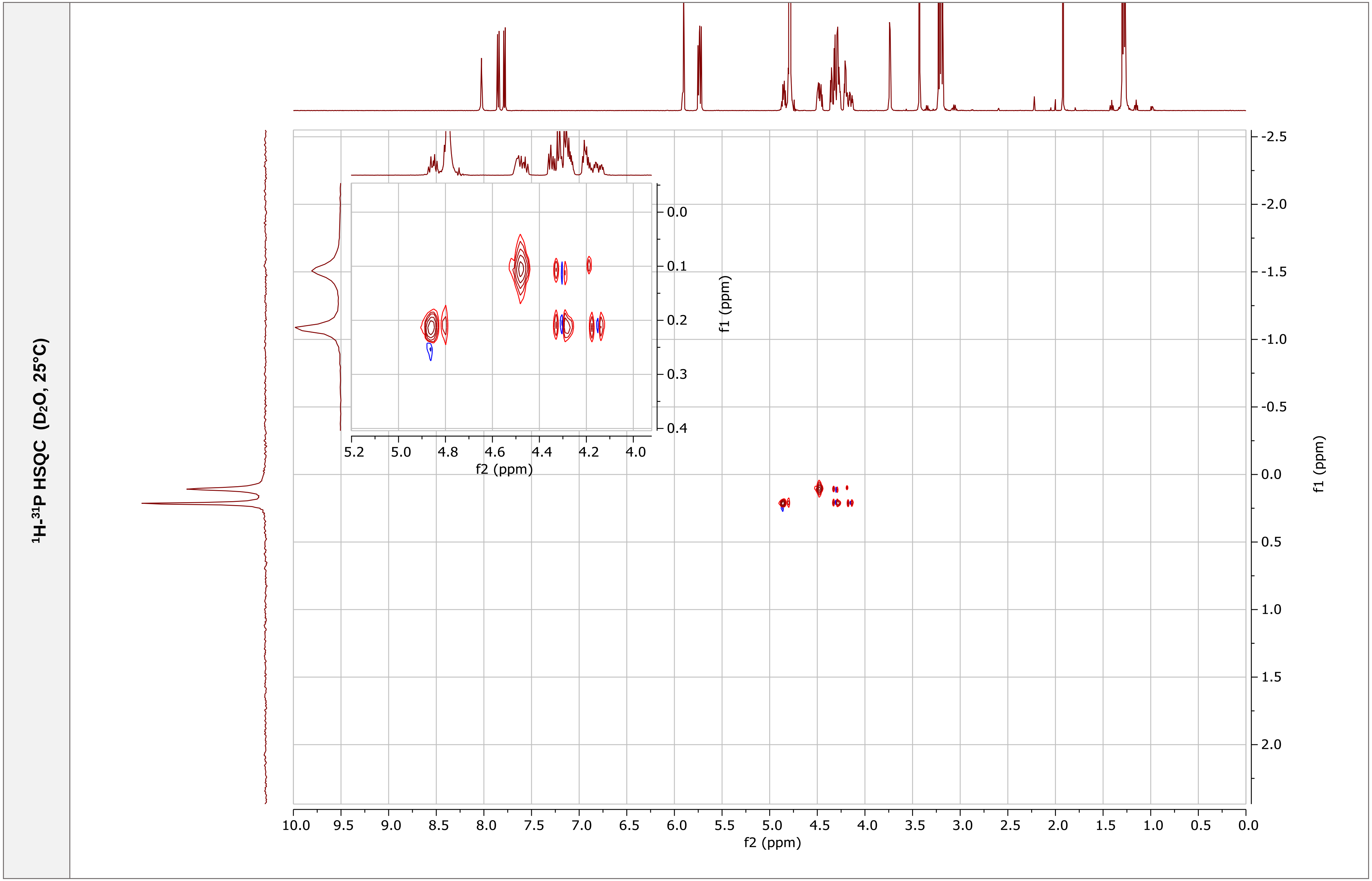

^{1}H-^{31}P HSQC (D$_2$O, 25°C)
f2 (ppm)
f1 (ppm)

(26) $m^7Gppp^{m6}A_mpG_mpG$

Chemical structure

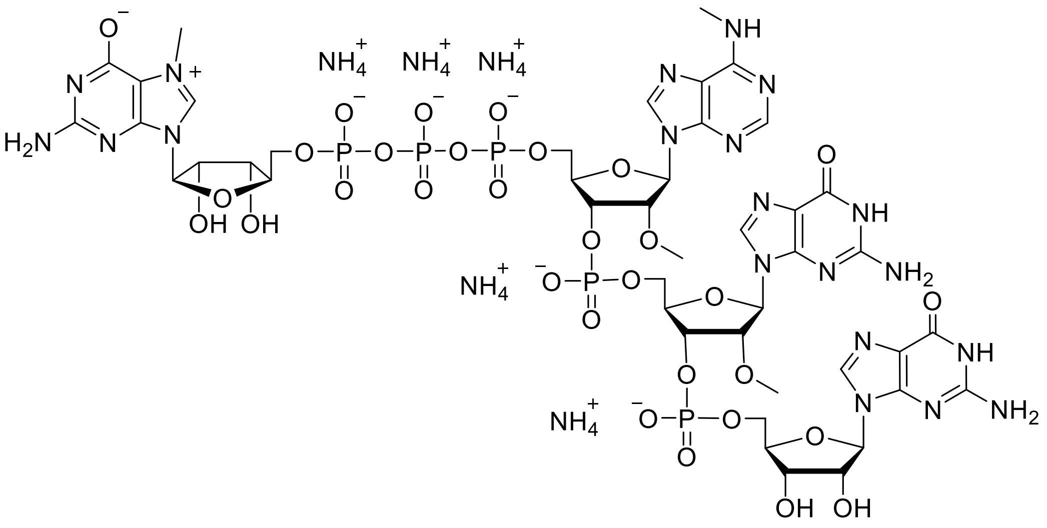

RP HPLC
Abs. @ 254 nm

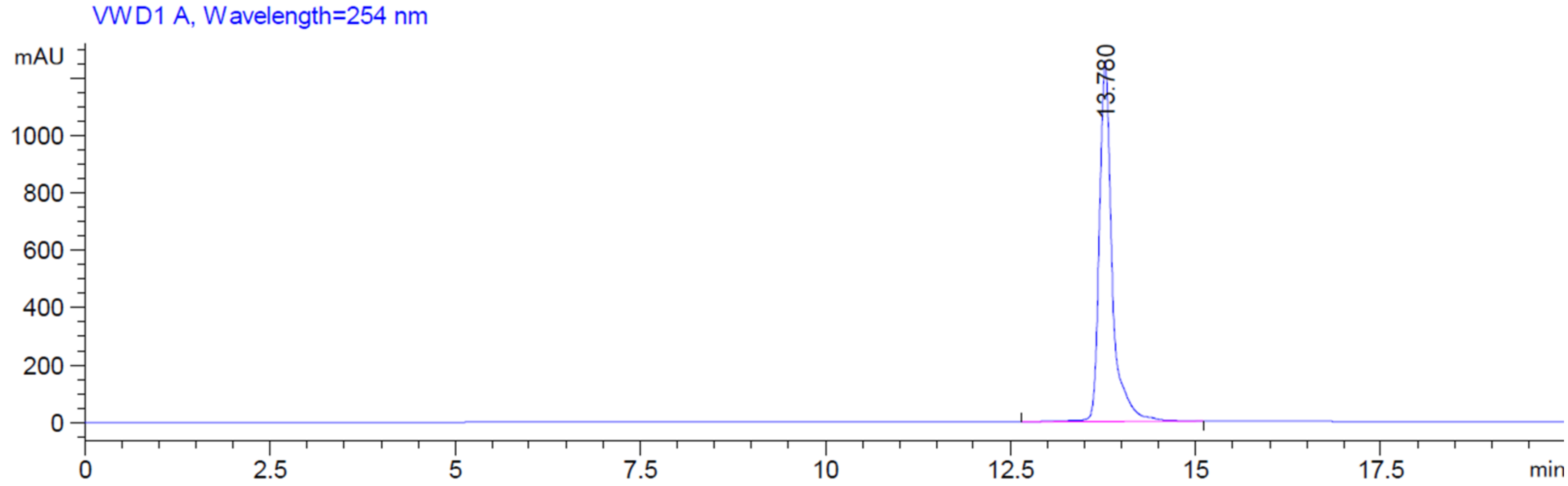

MS (-) ESI

(Calc. $[M-H]^-$ $C_{44}H_{58}N_{20}O_{31}P_5^-$ 1517.22704)

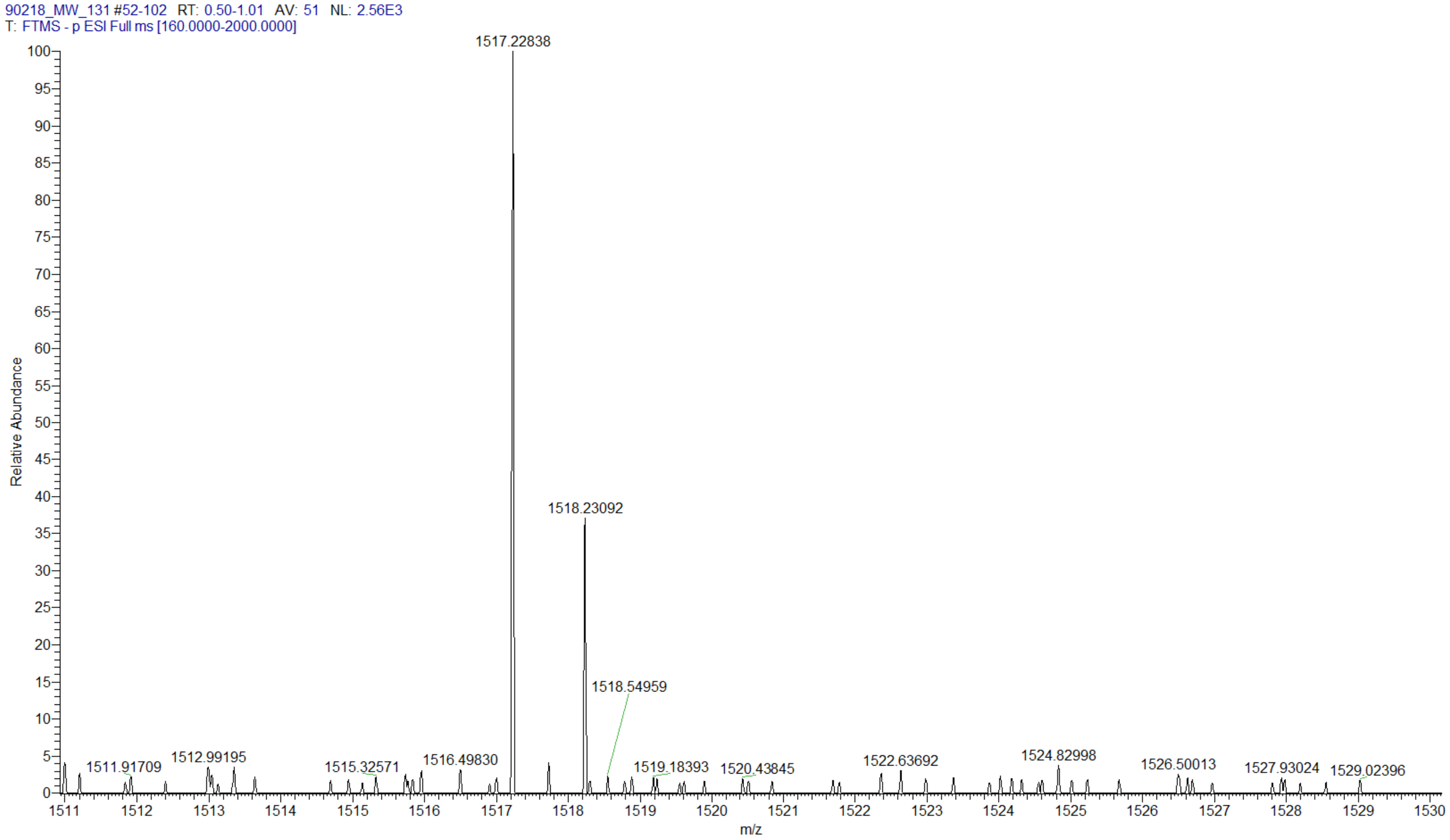

$(27)\ m^{7}Gppp^{m6,6}A_{m}pA_{m}pC_{m}p^{m3}U^{m}pA$	
**Chemical structure**	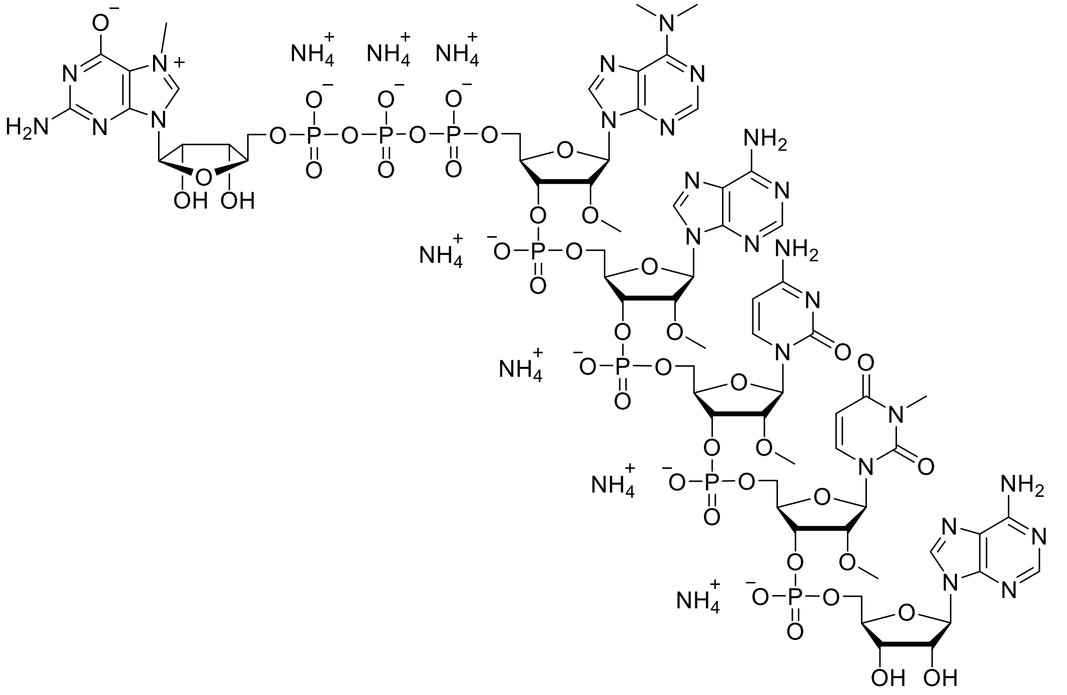
**RP HPLC** Abs. @ 254 nm	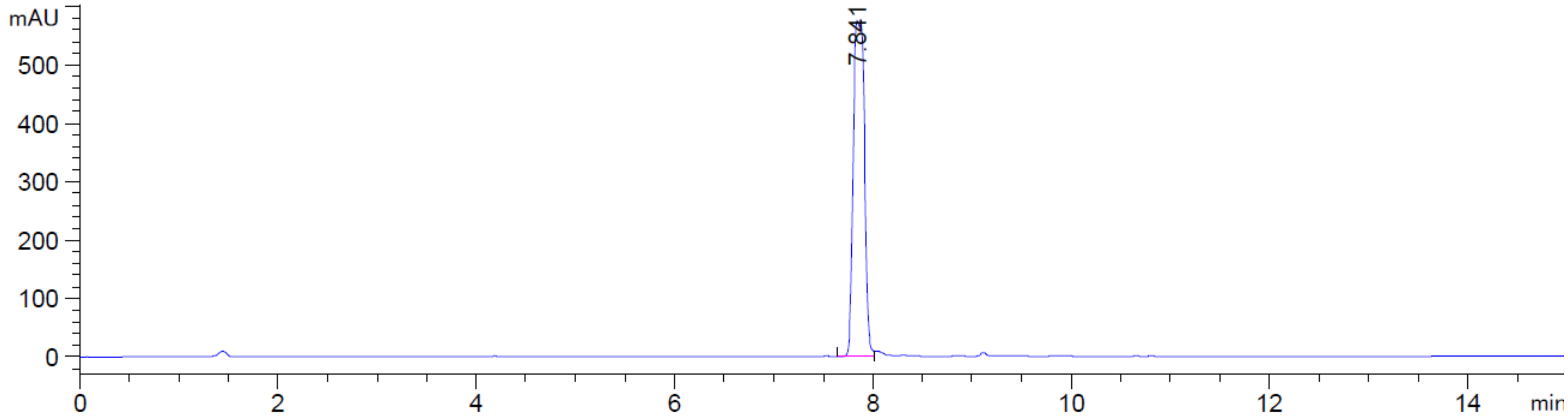

MS (-) ESI

(Calc. $[M-H]^-$ $C_{66}H_{89}N_{25}O_{44}P_7^-$ 2152.36640)

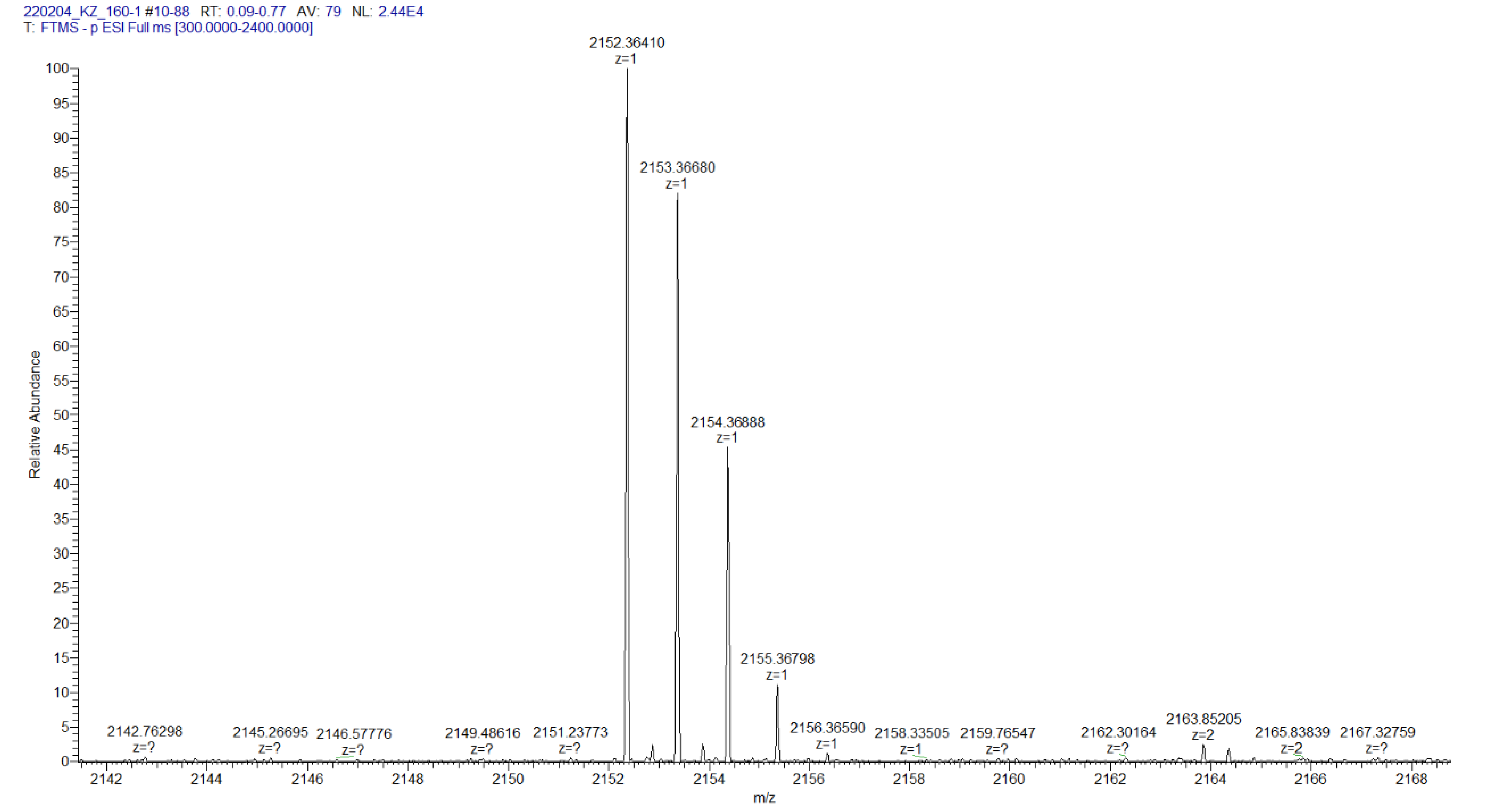

Supplement: Supplementary file 1 — jo2c01390_si_001.pdf [file jo2c01390_si_001.pdf]
